# Multidisciplinary Exploration of Computed‐Tomographic and Ancient‐DNA Data of an Iron Age Skull From Latvia With Multiple Lytic Bone Lesions: Differential Diagnosis Between Metastatic Carcinoma, Multiple Myeloma and Skeletal Tuberculosis

**DOI:** 10.1002/ece3.74058

**Published:** 2026-07-23

**Authors:** Alise Akermane‐Pokšāne, Jānis Ķimsis, Elīna Pētersone‐Gordina, Antonija Vilcāne, Guntis Gerhards, Mārtiņš Pēterfelds, Renāte Ranka

**Affiliations:** ^1^ Latvian Biomedical Research and Study Centre Riga Latvia; ^2^ Faculty of Humanities, Institute of Latvian History University of Latvia Riga Latvia; ^3^ Department of Radiology Riga Stradiņš University Riga Latvia

**Keywords:** aDNA, computed tomography, differential diagnosis, Iron Age Latvia

## Abstract

Multiple lytic lesions of the cranial vault are among the most diagnostically ambiguous findings in paleopathology, since at least three pathological processes (metastatic carcinoma, multiple myeloma and disseminated tuberculosis) can produce morphologically convergent “punched‐out” foci, while diagenetic erosion can mimic all three. Differential diagnosis based on macroscopic observation alone is therefore frequently inconclusive. The recent debate about whether multiple myeloma should be regarded as an ancient disease at all further reinforces the need for adjunct diagnostic streams. We hypothesised that, in a single archaeological individual presenting with multiple lytic cranial lesions, the integration of high‐resolution computed tomography (CT, including assessment of the postcranial skeleton) with shotgun ancient DNA (aDNA) analysis can constrain the differential diagnosis between metastatic carcinoma, multiple myeloma and tuberculosis even when overall aDNA preservation is limited. We analysed the cranium and the first cervical vertebra (atlas, C1) of a 40–50‐year‐old female from a disturbed grave at the Čunkāni‐Dreņģeri cemetery, Latvia, dated to the 10th CEntury CE, by subjecting it to direct visual paleopathological examination, computed tomographic and aDNA analysis. CT and aDNA streams support different leading diagnoses: CT favours metastatic carcinoma based on irregular margins and lesion‐size variability, whereas aDNA provides a weak signal compatible with tuberculosis. Hyperdiploid multiple myeloma is effectively excluded. The combined cranial‐plus‐cervical pattern is consistent with hematogenously disseminated systemic disease, and the partial diagenetic component requires histological and X‐ray fluorescence spectrometry authentication of lesion margins. The discordance between CT and aDNA streams demonstrates that single‐stream paleopathological assessment can be misleading, and that targeted *M. tuberculosis* in‐solution capture should be the next step.

## Graphical abstract

In this study we used a combined computed tomographic and archaeogenetic approach to analyse a skull and vertebra with lytic lesions from a disturbed grave in the Čunkāni‐Dreņģeri cemetery in Latvia, dated to 10th century CE. Both methodologies were used to evaluate a proposed differential diagnosis of multiple myeloma, metastatic carcinoma and bone tuberculosis. This study underlines the importance of employing a multifaceted approach including direct observations of archaeological bone tissue as possibly less subjected to damage caused by burial environment in comparison to biomolecules such as DNA, which makes it more descriptive in cases of poor DNA preservation.

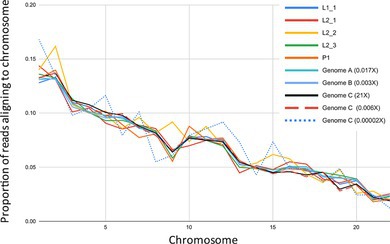

## 1 Introduction

### 1.1 Lytic Cranial Lesions in Paleopathology: Convergent Morphology, Divergent Aetiologies

Multiple lytic lesions of the cranial vault constitute one of the longest‐running diagnostic challenges in paleopathology. At least three discrete pathological processes (metastatic carcinoma, multiple myeloma and disseminated tuberculosis) can each produce sharply demarcated, ‘punched‐out’ osteolytic foci that, in the absence of soft tissue and laboratory testing, are morphologically convergent (Ortner 2003; Aufderheide and Rodríguez‐Martín 1998; Roberts and Manchester 2005). To these primary candidates must be added Langerhans cell histiocytosis, hyperparathyroidism, treponematosis, leprosy, Paget's disease, intraosseous benign neoplasms and parasitic disease, most of which can be excluded on the grounds of distribution, demographic profile or accompanying features, but each of which has been invoked, at least once, in published archaeological cases (Schultz 2003; Hackett 1976).

Besides the proposed causative agents of the observed lesions, which could have caused bone changes during the lifetime of the analysed individual, the possibility of the observed changes being a pseudopathology cannot be overlooked. Diagenetic processes, including microbial bioerosion (Hackett 1981; Turner‐Walker and Jans 2008), sediment infiltration (Fojtová et al. 2023), root etching, and the differential dissolution of bone tissue in acidic soils (Oghenemavwe et al. 2022), can produce defects that are macroscopically indistinguishable from true antemortem disease (Schmidt and Grosskopf 2025). The acidic, partly moist soils of the Eastern Baltic (Pētersone‐Gordina et al. 2018; Kazarina et al. 2021) constitute a diagenetic environment in which pseudopathological mimicry could be expected, but as the pseudopathological nature of these lesions could be assessed by methods unavailable to us like histological examination, X‐ray fluorescence spectrometry (Fojtová et al. 2023) and others, it will remain a future research prospect.

### 1.2 The State of the Art for the Three Principal Differential Diagnoses

#### 1.2.1 Multiple Myeloma

Multiple myeloma (MM) was first reported in archaeological context by Morse et al. (1974) and was for several decades treated as a well attested ancient disease (Kyle and Steensma 2011). Rothschild et al. (1998) formalised dry bone criteria distinguishing MM from metastatic carcinoma: MM lesions are described as sharply defined, spheroidal, with smooth margins and uniformly effaced trabeculae, in contrast to the irregular trabecular preservation and resorption fronts of metastatic carcinoma. Strouhal (1991) and Marks and Hamilton (2007) provided complementary differential frameworks. However, Riccomi et al. (2019) systematically reviewed published MM cases and concluded that most do not satisfy these criteria when re‐examined; Rothschild (2023) went further and proposed that MM may not be a true ancient disease at all, with the great majority of pre‐industrial cases more parsimoniously interpreted as metastatic carcinoma. Recent reports therefore tend to adopt the language of ‘probable’ rather than ‘confirmed’ MM (Dittmar et al. 2020; Phillips 2024). The genetic architecture of modern MM is well‐characterised: hyperdiploid MM (~50%–60% of cases) exhibits trisomies of chromosomes 3, 5, 7, 9, 11, 15, 19 and 21; non‐hyperdiploid MM is defined by *t*(14q32) translocations with partner chromosomes 4, 6, 11, 16 and 20 (Smadja et al. 2001; Prideaux et al. 2014). To our knowledge, no published paleopathological MM case has been tested against this genetic architecture using aDNA analysis.

#### 1.2.2 Metastatic Carcinoma

In contrast to MM, metastatic carcinoma as an ancient pathological condition is not currently disputed (Hunt et al. 2018; Marks and Hamilton 2007). The earliest documented case dates to ~1200 BC in ancient Nubia (Binder et al. 2014); subsequent reports span Liechtenstein (Cooper and Goritschnig 2024), Hungary (Antónia and László 2009; Merczi et al. 2014), Italy (Minozzi et al. 2018; Biehler‐Gomez et al. 2025), Egypt (Wahba et al. 2021; Panzer et al. 2025), Sudan (Binder et al. 2014), Russia (Schultz et al. 2007) and China (He et al. 2022). In adult women aged 40–50, breast adenocarcinoma is the leading primary source of calvarial metastases, followed by lung, thyroid and renal carcinoma (Marks and Hamilton 2007). Metastatic cranial lesions typically vary in size, with irregular or ‘moth‐eaten’ margins, resorption fronts; clinical CT analysis reports perilesional sclerosis in 38.9% of metastases versus 13.2% of MM cases, and high‐density areas in 85.2% versus 19% (Mutlu et al. 2021). Schlott et al. (2007) attempted PCR‐based detection of tumour‐related genes in a Scythian individual with metastatic skeletal lesions (Schultz et al. 2007), but to our knowledge no shotgun aDNA based oncogenetic study has been published for an archaeological metastatic cancer case.

#### 1.2.3 Skeletal Tuberculosis

Since calvarial perforating tuberculosis (Volkmann type I; Raut et al. 2004); can produce sharply gouged, full‐thickness lytic lesions without periosteal reaction, a morphology that, in dry bone, is indistinguishable from the ‘punched‐out’ pattern of MM or metastases makes tuberculosis the most relevant infectious agent. Calvarial TB constitutes < 1% of skeletal TB cases (Roberts and Buikstra 2003), but disseminated forms with cranial and craniocervical involvement are documented (Lifeso 1987; Govender et al. 2003). *Mycobacterium tuberculosis* aDNA has been authenticated in early‐medieval Europe (Bouwman et al. 2012; Bos et al. 2014; Donoghue et al. 2015) and also in Iron Age Lithuania at Marvelė (Faerman and Jankauskas 2000), establishing both ecological plausibility and a methodological precedent for the present study. Recent improvements in pathogen aDNA recovery and targeted in‐solution capture (Vågene et al. 2022), have made screening for *M. tuberculosis* complex more feasible in archaeological material.

### 1.3 The Rationale for a Multidisciplinary Approach

Although Hunt et al. (2018) systematically reviewed published archaeological cancer cases, of the more than 200 cases catalogued only a handful integrate macroscopic observation with high‐resolution radiological imaging; fewer still integrate radiology with aDNA. The Bianucci et al. (2021) study of a third‐century Roman bust illustrates the value of imaging in tightening differential diagnosis even in the absence of bone but adds no molecular dimension. Schlott et al. (2007) demonstrated that aDNA from archaeological cancer cases can be assayed by PCR but limited the analysis to a few candidate genes and did not couple it to imaging. Panzer et al. (2025) systematically CT screened 45 Egyptian mummies for bone and soft‐tissue tumours but did not perform genetic testing. To our knowledge, no published paleopathological study integrates volumetric CT (cranium and postcranial skeleton) with deep shotgun aDNA on the same archaeological individual for the explicit purpose of differential diagnosis between MM, metastatic carcinoma and tuberculosis.

This is the methodological gap the present study addresses. The two streams are complementary in three ways. First, they target different biological substrates: bone tissue (CT) and biomolecules (aDNA) degrade differently in the burial environment (Rollo et al. 2002), potentially compensating for the poorer data quality. Second, they provide independent kinds of evidence: CT yields lesion morphology, distribution and full skeletal context; aDNA yields chromosomal architecture (relevant to MM hyperdiploidy), variant calling (relevant to genetic predisposition) and pathogen identification (relevant to TB).

### 1.4 Specific Hypotheses and Falsifiable Predictions

#### 1.4.1 Prediction Set A: Multiple Myeloma

If the lesions reflect MM, we predict:

- Sharply demarcated, uniformly small (< 3 cm), spherical lesions with smooth margins and uniformly effaced trabeculae on CT (Rothschild et al. 1998)
- On shotgun aDNA, in hyperdiploid MM, detectable read‐depth deviations on chromosomes 3, 5, 7, 9, 11, 15, 19, 21 above the noise level established by control genomes (Smadja et al. 2001; Prideaux et al. 2014)
- Postcranially, lytic foci predominantly in the vertebral bodies, ribs, sternum, pelvis and proximal long bones, where red marrow is most active (Kyle and Rajkumar 2008; Angtuaco et al. 2004)
- Absence of pathogen‐specific aDNA signal

#### 1.4.2 Prediction Set B: Metastatic Carcinoma

If the lesions reflect metastatic breast carcinoma, we predict:

- Heterogeneously sized lesions (mm to multiple cm), with irregular margins, resorption fronts, and at least some perilesional density variation on CT (Mutlu et al. 2021)
- On aDNA, no characteristic aneuploidy pattern; possible pathogenic variants in BRCA1/2, PALB2, or other breast‐cancer‐associated loci, given low coverage
- Postcranially, lytic‐blastic foci preferentially in the axial skeleton with unilateral patchy distribution rather than uniform red‐marrow distribution (Marks and Hamilton 2007)
- Absence of pathogen‐specific aDNA signal

#### 1.4.3 Prediction Set C: Disseminated Tuberculosis

If the lesions reflect disseminated tuberculosis, we predict:

- Sharply punched‐out, fully transcortical lytic lesions without periosteal reaction or sclerosis on CT, distributed in the calvarium with possible craniocervical (atlanto‐axial) involvement (Volkmann type I; Raut et al. 2004; Lifeso 1987)
- Chromosomal aneuploidies absent
- Postcranially, possible Pott's disease of the vertebral column or extraspinal foci
- On aDNA, sequences alignable to *M. tuberculosis* complex with characteristic post‐mortem damage profiles (Skoglund et al. 2014), and dispersion across the reference genome rather than concentration in conserved regions (Feuerborn et al. 2020)

### 1.5 Aim of the Present Study

The aim of this study is to test the three hypotheses against integrated CT and aDNA data from a single Iron Age (10th century CE) Latvian individual with multiple lytic cranial lesions. This study demonstrates the methodological synergy of CT and aDNA in paleopathology; reports the first paleopathological application, to our knowledge, of read‐depth‐based screening for MM‐characteristic chromosomal aneuploidies; provides the first molecular evaluation of *M. tuberculosis* in an Iron Age Eastern Baltic individual with cranial lesions; and documents the diagenetic background against which any antemortem diagnosis must be authenticated.

## 2 Materials and Methods

### 2.1 Archaeological Context

The individual analysed in this study was recovered from a disturbed grave containing a partially preserved skull, mandible and a single vertebra (C1) at the Čunkāni‐Dreņģeri cemetery, situated in southwestern Latvia in the historical region of Zemgale (the territory of the Iron Age Semigallian cultural areal). The cemetery has been the subject of ongoing bioarchaeological investigation by the Institute of Latvian History; the broader context of diet, mobility and demography is described in Pētersone‐Gordina et al. (2023). Location of this burial in the cemetery and the associated grave goods together place the individual in the 10th century CE (Atgāzis 1994). The remains were curated in the Bioarchaeological Repository of the Institute of Latvian History, University of Latvia. Because the burial is more than 100 years old, no formal ethics permission was required under the Latvian law “On the Protection of the Body of Deceased Human Beings and the Use of Human Tissues and Organs in Medicine”, section 4, paragraph 4 (1992).

### 2.2 Osteological Estimation of Biological Sex and Age at Death

#### 2.2.1 Sex Estimation

Sex was estimated from cranial morphological traits scored on a five point ordinal scale according to Buikstra and Ubelaker (1994): the nuchal crest, mastoid process, supra‐orbital margin, glabella and mental eminence (the last as preserved on the mandible). The supra‐orbital margin and glabella were scored 1 (gracile), the mastoid process 2, and the nuchal crest 1, yielding an overall score consistent with female sex. We acknowledge that the cranium alone shows substantially less sexual dimorphism than the pelvis (Walker 2008; Buikstra and Ubelaker 1994); the morphological estimate is therefore reported as “probable female” and is subsequently corroborated by genetic XX assignment from shotgun sequencing data (Section 3.3). The genetic confirmation reduces the operational uncertainty of the morphological estimate to a level acceptable for the purposes of differential diagnosis.

#### 2.2.2 Age Estimation

Age at death was estimated by combining three independent macroscopic methods, applied to the cranium and the preserved mandibular dentition, with the explicit acknowledgement that single‐method age estimates from the cranium have wide error margins (Lovejoy 1985; Schmitt et al. 2002).

#### 2.2.3 Cranial‐Suture Closure

Ectocranial sutures (coronal, sagittal, lambdoidal) were scored according to Meindl and Lovejoy (1985) on the standard ten‐point composite scale. Closure was advanced (composite score 14–17), giving a point estimate of approximately 39–44 years with a 95% prediction interval of approximately ±10 years. We note explicitly that suture‐closure‐based estimates have been criticised for low precision and reliability (Hershkovitz et al. 1997); we use the result only as one constraint among several.

#### 2.2.4 Dental Attrition

The mandible of the individual is preserved with the majority of the dentition in situ. Occlusal attrition of the molars and premolars was scored according to Brothwell (1981) and Smith (1984). The first and second molars showed moderate attrition with dentine exposure on the cusps and limited dentine pooling, corresponding approximately to Brothwell stage 3–4 (mid‐stage). For populations with subsistence level abrasive diets, as expected in 10th‐century Eastern Baltic agricultural communities (Pētersone‐Gordina et al. 2023), this stage corresponds to an age range of approximately 35–45 years (Brothwell 1981; Lovejoy 1985), with a recognised dependence on diet, food preparation technology and individual occlusal mechanics. The dental attrition estimate is therefore concordant with the suture closure estimate.

#### 2.2.5 Radiological Signs

On CT, cranial sutures appeared closed, dense and sclerotic; bone density was preserved at 500–1000 HU; cranial dimensions (latero‐lateral 15 cm, ventro‐dorsal 17 cm) were within normal adult limits. Radiological age estimation yielded an interval of approximately 30–60 years (broad, but consistent).

Combining the three methods yields a composite age‐at‐death estimate of 40–50 years, with overlapping but non‐identical individual ranges. We retain this estimate for the present study but emphasise that single individual age estimates from cranial methods are inherently imprecise and that the ‘40–50 years’ band should be read as a conservative midpoint with uncertainty of approximately ±10 years on either side.

### 2.3 Morphological and Computed‐Tomographic Analysis

Two complementary visual modalities were used to assess the cranium and the cervical vertebra: direct visual paleopathological examination and computed‐tomographic (CT) analysis. The relative roles of the two modalities are summarised below.

#### 2.3.1 Direct Visual Paleopathological Examination

The morphological description of the cranial and cervical lesions, including their anatomical distribution, gross size, shape (spheroidal, lobulated, irregular), margin character (sharp vs. ill‐defined, smooth vs. irregular), the presence or absence of reactive bone formation at the lesion periphery, and the macroscopic distinction between probable arachnoid foveae and venous lacunae on the one hand and probable pathological foci on the other, was based on direct visual paleopathological examination of the specimen in accordance with standard paleopathological practice (Aufderheide and Rodríguez‐Martín 1998; Ortner 2003; Roberts and Manchester 2005). Lesion margins, surface texture and reactive changes were recorded for each focus. The dry‐bone criteria of Rothschild et al. (1998) for the differentiation of multiple myeloma from metastatic carcinoma, and the criteria reviewed by Roberts and Buikstra (2003) for skeletal tuberculosis, were applied to the observations obtained from this examination.

#### 2.3.2 Computed Tomographic Acquisition and Analysis

##### 2.3.2.1 Imaging Protocol

CT was performed on a GE Revolution scanner (GE Healthcare Technologies Inc.) with a slice thickness of 0.6 mm in helical acquisition mode. Both the cranium and the first cervical vertebra (atlas, C1) were scanned. Volumetric data were reconstructed at 0.6 mm voxel size and reviewed in axial, coronal and sagittal multi‐planar reformations together with three‐dimensional volume‐rendered surface views, in soft tissue and bone windows.

##### 2.3.2.2 Quantitative Description of Lesions

**FIGURE 1:**
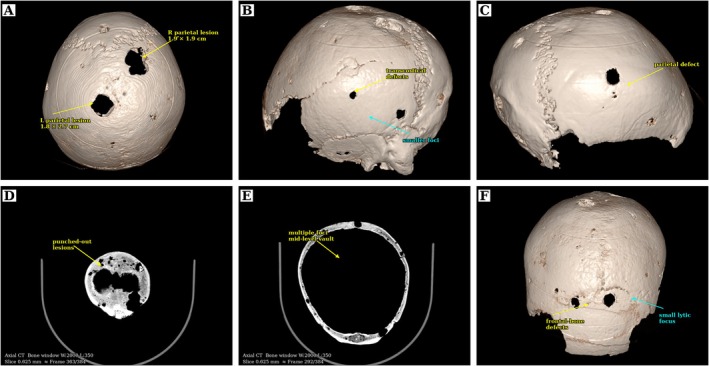
Computed‐tomographic and three‐dimensional volume‐rendered findings of the cranial vault. (A) Three‐dimensional volume‐rendered superior view of the cranium, showing the two largest lytic foci of the parietal vault: a right parietal lesion measuring 1.9 × 1.9 cm and a left parietal lesion measuring 1.8 × 2.7 cm. Both lesions exhibit irregular, non‐sclerotic margins and full‐thickness destruction of the diploë and external/internal tables. The wider opening of each lesion on the outer cortical surface and the substantial size variability across the cranium are morphological features more consistent with metastatic carcinoma than with multiple myeloma per the Rothschild–Hershkovitz–Dutour (1998) framework. (B) Three‐dimensional volume‐rendered right‐lateral view, demonstrating the parietal transcortical defects in profile. (C) Three‐dimensional volume‐rendered left‐lateral view, demonstrating bilaterality of the lesion distribution. (D) Axial computed‐tomographic section through the upper parietal vault (≈frame 363/384, approximately 4 cm above the supra‐orbital plane). Multiple punched‐out lytic lesions are visible distributed around the cranial cross‐section. The lesions are sharply demarcated, fully transcortical, without surrounding sclerosis or reactive bone formation. Bone window: W 2000 HU; L 350 HU; slice thickness 0.625 mm. (E) Axial computed‐tomographic section through the mid‐level cranial vault (≈frame 292/384, approximately 7 cm above the supra‐orbital plane), showing multiple smaller punched‐out foci in the parietal and occipital cortices. Same imaging parameters as in (D). (F) Three‐dimensional volume‐rendered frontal view, showing three well‐defined lytic defects in the frontal bone, demonstrating involvement of the *os frontale* in addition to the *os parietale* and *os occipitale*.

Each candidate lytic focus was measured in two perpendicular dimensions on axial slices and characterised by (1) margin geometry (sharply demarcated vs. irregular vs. bevelled); (2) presence or absence of perilesional sclerosis or reactive bone formation; (3) involvement of the inner versus outer cortical table; (4) origin in cancellous (diploë) versus cortical bone; (5) presence or absence of a button sequestrum. The position and number of foci were recorded per anatomical region. Axial and 3D‐rendered images were prepared at the locations described in Figure 1.

##### 2.3.2.3 Indication of Section Levels

The views shown in Figure 1 are explicitly indicated as follows: three‐dimensional volume‐rendered reconstructions are presented in panel A (superior view), panel B (right‐lateral view), panel C (left‐lateral view) and panel F (frontal view); axial CT sections are shown in panel D (upper parietal vault, ≈frame 363/384, approximately 4 cm above the supra‐orbital plane; bone window W 2000 HU, L 350 HU, slice thickness 0.625 mm) and panel E (mid‐level cranial vault, ≈frame 292/384, approximately 7 cm above the supra‐orbital plane; same imaging parameters).

##### 2.3.2.4 Atlas (C1) Examination

In addition to the cranial vault, the first cervical vertebra was systematically examined because the upper cervical spine is a recognised but under‐reported site of disseminated TB (Lifeso 1987; Govender et al. 2003) and metastatic disease (Constans et al. 1983; Fornasier and Horne 1975). The atlas was identified anatomically by its closed ring without a vertebral body, paired transverse foramina, concave superior articular facets and anterior/posterior tubercles (Standring 2021). Each lateral mass and arch was examined separately for focal lytic defects, diffuse cortical loss and reactive change.

### 2.4 Sampling for Ancient DNA Analysis

#### 2.4.1 Sample Selection Rationale

Five samples were generated representing the whole spectrum of bone preservation observed macroscopically and on CT: one positive control (P1, petrous portion of the temporal bone, the optimal substrate for endogenous‐DNA recovery (Pinhasi et al. 2015; Margaryan et al. 2018)); one hard‐cortex control sampled in proximity to one of the smaller lesions (L1_1, occipital bone, intact cortex); and three samples from the eroded parietal region adjacent to one of the larger lytic foci (L2_1, L2_2, L2_3), the latter two from regions described as “chalky” on macroscopic inspection. To minimise the damage, sampling was done in the vicinity of just two of the observed lesions. The contrast in bone density between sample groups (hard vs. chalky) was deliberate, in order to allow assessment of how bone preservation interacts with both human aDNA recovery and microbial colonisation (Section 3.5).

#### 2.4.2 Bone Preprocessing and Powdering

All sampling was performed in dedicated clean‐room facilities for aDNA at the Latvian Biomedical Research and Study Centre, following standard precautions (Fulton and Shapiro 2019). Each sampled area was first mechanically cleaned using a single‐use brush, then washed sequentially in 6% sodium hypochlorite, double‐distilled water, and 70% ethanol, and air dried. Bone powder (50–80 mg) was obtained by drilling with a Dremel rotary tool at low speed, using sterilised drill bits.

#### 2.4.3 DNA Extraction and Library Construction

DNA was extracted following a modified Dabney et al. (2013) based Velsko et al. (2020) silica‐binding protocol with three sequential incubations (15 min at 50°C; overnight at 37°C; overnight at 54°C); supernatants of the two overnight incubations were pooled and used for DNA extraction. NGS libraries were constructed using the QIAseq Ultralow Input Kit (Qiagen, Cat. No. 180497) with the included polymerase replaced by AmpliTaq Gold 360 Master Mix (ThermoFisher Scientific, Cat. No. 4398881), to improve performance on damaged templates (Gansauge and Meyer 2013). Library concentrations were measured by Qubit dsDNA HS Assay Kit (Life Technologies); fragment size distribution by Agilent 2100 Bioanalyzer with the High‐Sensitivity DNA Kit; adapter‐dimer spikes were removed with the Nucleomag NGS Clean‐Up and Size Select Kit (Macherey‐Nagel) where present. Two negative controls (extraction blank, library construction blank) were processed in parallel and sequenced on Illumina MiSeq (v2 500‐cycle kit, paired‐end). Test libraries were converted to MGI compatibility (MGIEasy Universal Library Conversion Kit) and sequenced from both ends on a DNBSEQ‐G400 instrument (DNBSEQ‐G400RS High‐throughput Sequencing Set V2.0, FCL PE100) using default parameters.

### 2.5 Bioinformatic Analysis

#### 2.5.1 Read Pre‐Processing

Sequencing reads were trimmed and merged with ClipAndMerge v1.7.8 (Peltzer et al. 2016), specifying the standard Illumina TruSeq adapter sequences, a minimum length of 25 bp and a base‐quality cutoff of Q20.

#### 2.5.2 Human‐Genome Alignment

Reads were aligned to a combined GRCh37 nuclear genome and rCRS mitochondrial reference using bwa aln (parameters ‐l 1024 ‐n 0.01 ‐o 2; Oliva et al. 2021). Reads shorter than 33 bp or with mapping quality < 30 were excluded; PCR duplicates were removed (Picard MarkDuplicates; Broad Institute 2025). Sex was assigned using the Ry_compute.py script (Skoglund et al. 2013). Autosomal polyploidy was screened by comparing per‐chromosome read‐depth ratios across the test samples, two unrelated Iron Age genomes (A, B) processed by the same pipeline, and one modern high‐coverage genome (C) downsampled to comparable depth (Section 3.3). Variant calling was performed using bcftools (Danecek et al. 2021), restricted to positions overlapping between the petrous control P1 and the test samples (samples L1_1, L2_1, L2_2 and L2_3) and supported by ≥ 5 deduplicated reads in at least one non P1 library.

#### 2.5.3 Authentication and Contamination Assessment

aDNA authenticity was assessed by two complementary methods: (1) characterisation of damage‐associated transition frequencies at the read termini using mapDamage 2.0 (Jónsson et al. 2013); and (2) post hoc filtering of reads with PMD (post‐mortem damage) score < 2 using PMDtools (Skoglund et al. 2014). Negative controls were assessed for human aligning read proportions and damage profiles in parallel. Keeping only damaged sequences significantly lessens the overall quality of the alignment in exchange for decreased modern contamination risks. In case of extremely low read counts (< 50), samples were excluded from further analysis.

#### 2.5.4 Pathogen Screening: Metagenomic and Direct Alignment

Two complementary approaches were used. (1) Metagenomic profiling: deduplicated, end‐trimmed reads (5 bp from each end; Gordon 2010; Shen et al. 2016) were classified using Kraken2 (Wood and Salzberg 2014) with a standard prebuilt database, and species‐level abundances refined with Bracken (Lu et al. 2017). Reads classified as *Delftia*, a frequent reagent contaminant (Salter et al. 2014), were removed; contamination was further mitigated using microDecon (McKnight et al. 2019); results were visualised with Pavian (Breitwieser and Salzberg 2020) and MicrobiomeAnalyst (Dhariwal et al. 2017; Chong et al. 2020). (2) Direct alignment: reads were aligned to the *M. tuberculosis* H37Rv reference (AL123456.3) using bwa aln with the same parameters as for the human alignment; PMD‐filtered with PMDscore ≥ 3 (Skoglund et al. 2014); the highly conserved 16S rRNA region (1,471,846–1,473,382 bp) was excluded to reduce cross‐mapping with environmental mycobacteria (Walsh et al. 2019). Where metagenomic profiling implicated other potentially relevant taxa, direct alignment to species specific references (e.g., *Salmonella enterica* ) was performed under the same parameters.

### 2.6 Hypothesis‐Testing Framework

In line with the explicit hypotheses formulated in Section 1.4, each diagnosis was scored on four evidence channels: cranial CT morphology; C1 atlas CT morphology; pathogenic changes in human genomic alignment data and microbial pathogen aDNA. A diagnosis was classified as supported if at least three of four channels were consistent with it and no channel actively contradicted it; weakened if one or more channels did not provide any evidence consistent or contradictory; excluded if the contradiction occurred on a channel with high diagnostic specificity (e.g., pathogenic changes in human genomic alignment data expressed as read‐depth aneuploidy for hyperdiploid MM).

## 3 Results

### 3.1 Cranial CT Findings

CT of the cranial vault revealed multiple lytic foci of mixed character distributed across the frontal, parietal and occipital bones (Figure 1A–F). Cranial sutures were closed, dense and sclerotic; bone density was preserved at 500–1000 HU; cranial dimensions were 15 cm latero‐lateral and 17 cm ventro‐dorsal, within normal adult limits. Bone thickness was unchanged. A fresh fracture line in the left parietal bone, with sharp contours and no diagenetic infill, was interpreted as post‐excavation damage and is excluded from further analysis. No other signs of acute or remote trauma were identified.

The lytic foci segregated into two morphological classes.

#### 3.1.1 Class 1 (Likely Benign)

Smooth or slightly lobulated foci with openings towards the central skull cavity and no or minimal full‐thickness destruction. These features are consistent with intraosseous arachnoid granulations, venous lacunae, epidermoid bone cysts, or aneurysmal bone cysts (Murphey et al. 2002). The arachnoid granulation interpretation is supported by their predominantly parasagittal distribution adjacent to the superior sagittal sinus.

#### 3.1.2 Class 2 (Likely Malignant)

Foci with full‐thickness bone destruction, irregular outline and wider openings on the outer than the inner table. The largest such foci were bilateral in the parietal bone: 1.8 × 2.7 cm on the left and 1.9 × 1.9 cm on the right (Figure 1A). No reactive bone formation, periosteal new‐bone, perilesional sclerosis, or button sequestrum was identified.

Applied to the Rothschild–Hershkovitz–Dutour (1998) framework, the Class 2 features, namely irregular outline and substantial size variability (millimetres up to approximately 3 cm), favour metastatic carcinoma over multiple myeloma, since MM lesions are characterised as ‘sharply defined, spheroidal, with smooth margins and uniformly effaced trabeculae’ and are usually uniformly small (~20 mm; upper limit < 30 mm; Rothschild et al. 1998; Riccomi et al. 2019). The wider openings on the outer table, with full‐thickness destruction, are also more characteristic of metastatic spread than of MM, which tends to involve the diploë more uniformly. The absence of any sclerotic component is, however, somewhat less typical of breast metastases, which often elicit a mixed lytic‐blastic response (Mutlu et al. 2021), and is more aligned with MM or with the perforating form of calvarial TB (Volkmann type I; Raut et al. 2004).

#### 3.1.3 Provisional CT‐Based Interpretation

The Class 2 lesions are most consistent with metastatic carcinoma; multiple myeloma is a less probable but non‐excluded alternative; calvarial perforating tuberculosis remains compatible on morphological grounds alone.

### 3.2 Atlas (C1) CT Findings

**FIGURE 2:**
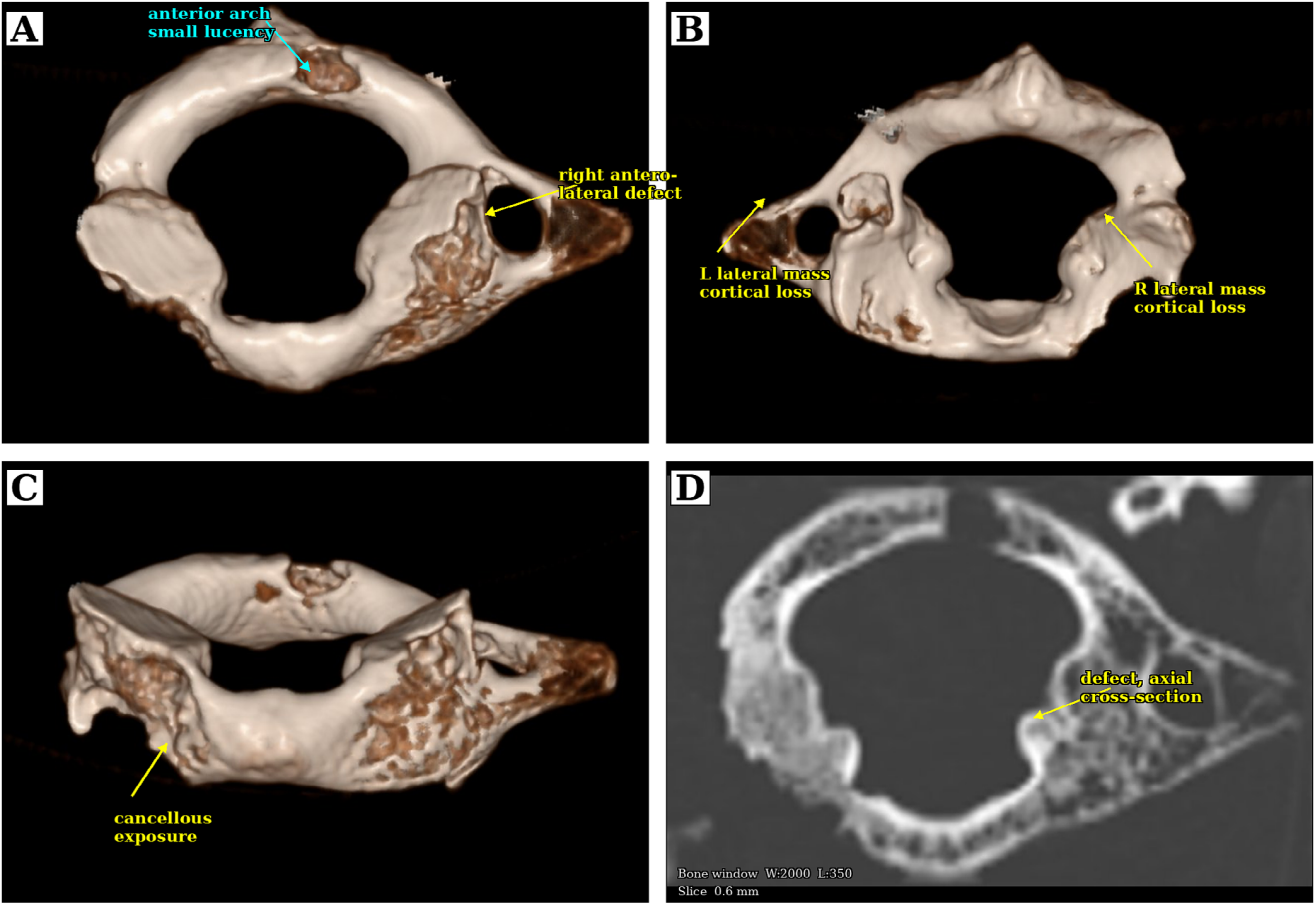
Computed‐tomographic findings of the first cervical vertebra (atlas, C1). (A) Three‐dimensional volume‐rendered superior view of the C1 atlas. The lower arrow indicates a discrete, sharply demarcated, fully transcortical defect in the right anterolateral region of the lateral mass, immediately medial to the right transverse foramen. The upper arrow indicates a second, smaller lucency on the anterior arch, consistent with multifocal involvement of C1. (B) Three‐dimensional volume‐rendered inferior view of the same vertebra, showing bilateral cortical loss with cancellous exposure on the left (L) and right (R) lateral masses. Brown‐ and orange‐rendered regions correspond to areas of lower bone density. The biological interpretation of these areas is ambiguous (see Section 3.5): The lateral masses of the atlas have intrinsically thin cortex (Doherty and Heggeness 1994; Panjabi et al. 1991) and are disproportionately vulnerable to diagenetic loss in acidic, moist soils such as those of the Eastern Baltic. (C) Oblique inferior 3D volume‐rendered view, providing a complementary perspective on the cancellous exposure documented in (B). (D) Axial computed‐tomographic section through the level of the foramina transversaria of C1, showing the right anterolateral defect in cross‐section. Image acquired at 0.6 mm slice thickness; bone window (W: 2000 HU; L: 350 HU). The defect appears as a focal cortical interruption with non‐sclerotic margins, morphologically corresponding to the punched‐out pattern of the Class 2 cranial lesions (see Section 3.1).

CT of the first cervical vertebra revealed two principal abnormalities (Figure 2).

A discrete, sharply demarcated, fully transcortical defect in the right anterolateral region of the lateral mass, immediately medial to the right transverse foramen. The defect appears approximately circular with relatively non‐sclerotic margins. Its morphology (rounded, well‐defined, non‐sclerotic, fully transcortical, with no peripheral remodelling) corresponds closely to the ‘punched‐out’ pattern of the Class 2 cranial lesions (Section 3.1). A second smaller lucency is visible on the anterior arch in the same view, suggesting multifocality on C1 itself.

Bilateral cortical loss with cancellous exposure on the lateral masses and the inferior aspect, rendered in lower density on volumetric reconstructions. Differentiation between antemortem disease and diagenetic loss is biologically ambiguous: the lateral masses of the atlas have intrinsically thin cortex (Doherty and Heggeness 1994; Panjabi et al. 1991) and are disproportionately vulnerable to diagenetic loss, which in acidic, moist soils can produce a lesion‐like appearance (Schmidt and Grosskopf 2025).

#### 3.2.1 Diagnostic Significance

If antemortem, the C1 finding establishes that the disease process is not confined to the calvarium but has extended to the upper cervical spine. Hematogenously disseminated systemic disease is the most parsimonious explanation. Tuberculous spondylitis of C1 (Pott's disease, atlas form), although accounting for only 0.3%–1% of all cases of tuberculous spondylitis (Lifeso 1987; Govender et al. 2003), classically produces destruction of the lateral masses or anterior arch with sharp non‐reactive margins; the morphology of the right anterolateral defect fits this pattern. Atlas metastases account for < 1% of spinal metastases (Constans et al. 1983; Fornasier and Horne 1975) but are documented; in adult women, breast carcinoma is the most likely primary. Multiple myeloma less commonly involves the atlas since the atlas lacks a true vertebral body and the lateral masses contain only a limited volume of haematopoietic marrow (Kyle and Rajkumar 2008).

### 3.3 Human aDNA Preservation, Sex Confirmation and Chromosomal‐Aneuploidy Screen

#### 3.3.1 Read Counts and Preservation

A mean of ~186 million reads were generated per sample. Human DNA preservation was poor with the human DNA proportion being negligible (< 0.1%) for most samples, with L1_1 as the only exception (~3.9%). The petrous control (P1) showed a sequencing‐saturation score > 0.9, indicating that further sequencing could not substantially improve recovery. After PMD filtering, mean depth across samples was 0.0013× and mean coverage 0.117% (Table 1). All five test samples were successfully authenticated by both mapDamage 2.0 transition frequency analysis and PMDtools filtering.

**TABLE 1:** Sequencing and human alignment metrics of the processed libraries.

Sample	Trimmed reads	Quality filtered reads before deduplication	Duplicates	Deduplicated quality filtered reads	Sequencing saturation	Human DNA proportion (%)	Transition frequency at the first relative position		Number of mtDNA reference aligned reads	Average sequencing depth (X), before PMD filtering	Average coverage (%), before PMD filtering	Reads with PMDscore ≥ 3	Proportion of human reads with PMDscore ≥ 3 (%)	Average sequencing depth (X) after PMD filtering	Average coverage (%) after PMD filtering
							5′C>T (%)	3′G>A (%)							
P1	325,270,973	1,762,804	1,687,710	75,094	0.957	0.023	5.44	3.32	23	0.0038	0.2084	4613	6.14	0.00016	0.01358
L1_1	186,932,153	9,243,830	1,887,188	7,356,642	0.204	3.935	0.71	0.70	605	0.2613	20.9959	149,332	2.03	0.00527	0.49406
L2_1	157,741,889	60,968	10,545	50,423	0.173	0.032	7.58	6.15	49	0.0017	0.1632	4565	9.05	0.00014	0.01361
L2_2	21,179,118	15,260	8391	6869	0.550	0.032	9.39	4.29	6	0.0002	0.0193	851	12.39	0.00003	0.00253
L2_3	239,653,266	230,629	56,938	173,691	0.247	0.072	7.45	4.55	64	0.0063	0.5929	17,398	10.02	0.00066	0.06296
B‐ex	1,727,725	726,555	574,534	152,021	0.791	8.799	0.40	0.40	91	0.0054	0.4556	1048	0.69	0.00004	0.00332
B‐lib	1,724,481	18,132	16,278	1854	0.898	0.108	0	0.51	76	0.0001	0.0058	13	0.70	0.00000	0.00004

#### 3.3.2 Sex Confirmation

XX chromosomal sex was confidently assigned for sample L2_3 (> 10,000 authenticated reads); samples P1, L2_1, and L2_2 were consistent with XX assignment but did not meet the high‐confidence threshold; sample L1_1 as not assigned despite a higher read count, due to an ambiguous Ry value (0.0325) likely reflecting low‐level contamination or technical noise. The genetic XX assignment is concordant with the morphological estimate of female sex (Section 2.2) (Table 2).

**TABLE 2:** Sex assignment results for the processed libraries.

Sample	Number of quality filtered human reads	Sum of Y and X aligning reads	Number of Y alighing reads	Ry value	Standard error	95% Confidence interval	Assignment
P1	4613	236	6	0.0254	0.0102	0.0053–0.0455	Consistent with XX but not XY
L1_1	149,332	6594	214	0.0325	0.0022	0.0282–0.0367	Not Assigned
L2_1	4565	226	5	0.0221	0.0098	0.0029–0.0413	Consistent with XX but not XY
L2_2	851	37	0	0	0	0.0–0.0	Consistent with XX
L2_3	17,398	824	7	0.0085	0.0032	0.0022–0.0148	XX

#### 3.3.3 Chromosomal‐Aneuploidy Screen for Hyperdiploid MM

**FIGURE 3:**
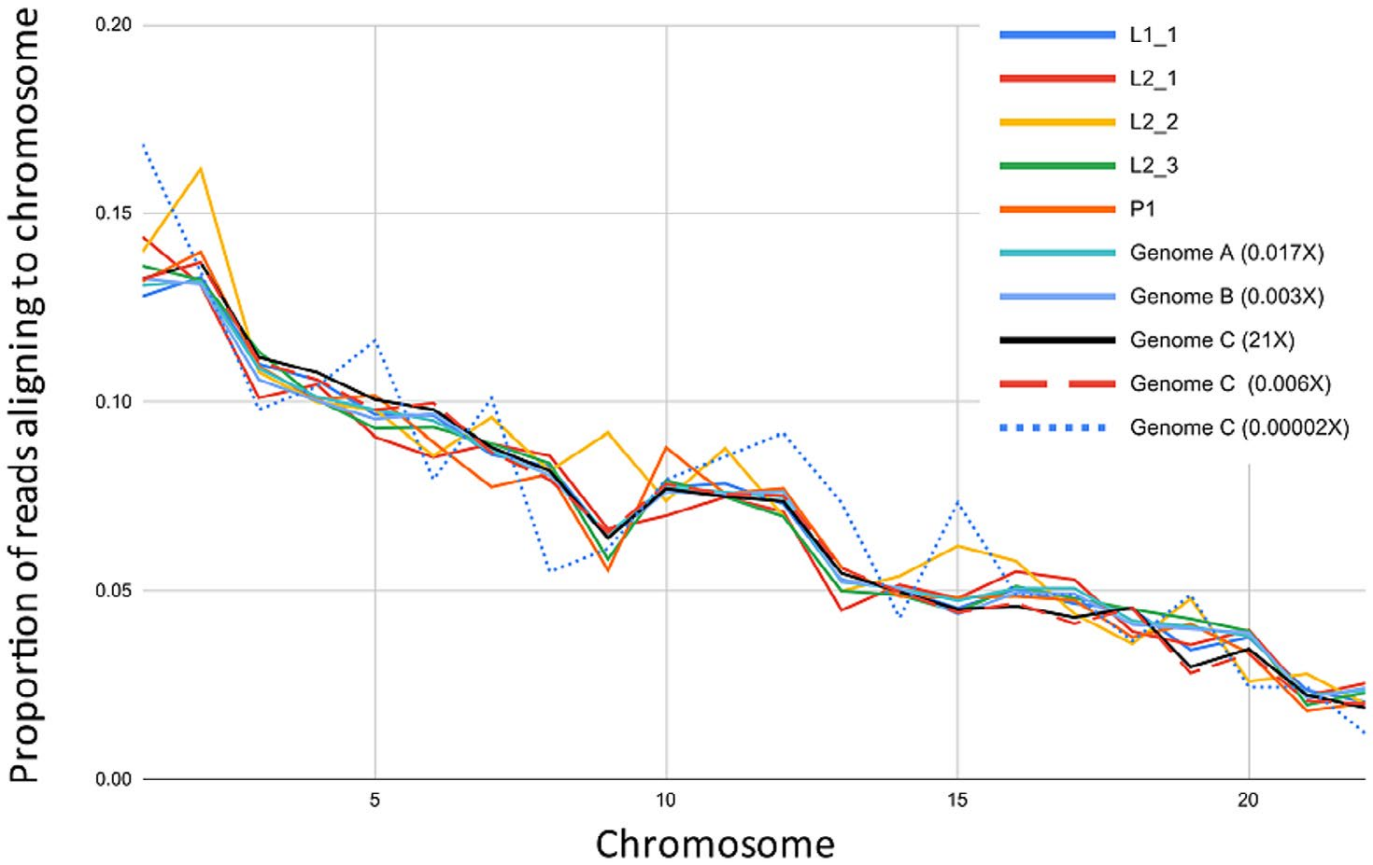
Per‐chromosome normalised read‐depth ratios. Normalised read proportions across all chromosomes for the test samples (P1, L1_1, L2_1, L2_2, L2_3), the Iron Age controls A and B, and the down‐sampled modern genome C. For normalisation, chromosomes 3, 5, 7, 9, 11, 15, 19 and 21 (relevant to hyperdiploid MM) were excluded from the denominator. All samples follow the same trend, including modern genome C. The dip at chromosome 9 and rise at chromosome 19 are consistent across controls, identifying these as reference‐mapping artefacts.

Per‐chromosome normalised read‐depth ratios were compared across the test samples, the unrelated Iron Age controls A and B, and the down‐sampled modern genome C (Figure 3). Hyperdiploid MM is expected to manifest as elevated read‐depth on any combination of chromosomes 3, 5, 7, 9, 11, 15, 19 and 21 (Smadja et al. 2001; Prideaux et al. 2014). No such pattern was detected. A modest decrease at chromosome 9 and an increase at chromosome 19 was observed in all samples including the controls and the down‐sampled modern genome, identifying it as a reference‐mapping artefact rather than a biological signal. Sample L2_2 showed an idiosyncratic pattern attributable to its extremely low read count, as can be seen when Genome C is downsampled to a comparable depth (Figure 3).

This result excludes hyperdiploid MM, the most common MM subtype (~50%–60% of cases). Non‐hyperdiploid MM, defined by *t*(14q32) translocations with partner chromosomes 4, 6, 11, 16 and 20, cannot be excluded with the current low‐coverage data, since translocation detection requires sufficient depth across IGH‐locus breakpoints. We classify hyperdiploid MM as excluded and non‐hyperdiploid MM as not testable but morphologically unlikely given the CT findings (Section 3.1).

#### 3.3.4 Screening for BRCA1/2, PALB2, and Other Potentially Oncogenic Variants

Based on the damaged human sequence alignments, 82 genome positions could be identified which overlapped with petrous bone positive control P1 and were covered by 5 or more sequencing reads from at least one of the analysed test libraries. After visual inspection of reads supporting called variants, no two or more nucleotide repeat sequences were identified, allowing us to exclude imprecise mapping to repeat regions of the reference. No positions overlapping all five of the samples were found (Table 3).

**TABLE 3:** The filtered list of identified variants found both in the positive control and at least one test sample. Numbers represent the number of reads which contain the REF or ALT allele.

Chromosome	Position	Reference alele	Alternative alele	P1 (REF|ALT)	L1_1 (REF|ALT)	L2_1 (REF|ALT)	L2_2 (REF|ALT)	L2_3 (REF|ALT)	Gene
1	91852821	G	A	0|2	0|12	0|8	0|0	0|3	HFM1
1	91852,879	G	A	0|2	0|23	0|11	0|0	1|4	HFM1
1	91852901	C	T	0|2	0|22	0|7	0|0	1|3	HFM1
1	121484779	G	A	0|4	9|5	0|0	1|0	3|4	Intergenic space
1	121484835	A	T	0|1	10|2	0|0	1|0	3|1	Intergenic space
1	121484847	T	G	0|1	12|1	0|0	0|0	2|0	Intergenic space
1	121484852	G	A	0|1	11|1	0|0	0|0	2|1	Intergenic space
1	121484855	T	G	0|1	11|0	0|0	0|0	2|1	Intergenic space
1	121485001	G	A	0|1	0|21	0|1	0|0	0|4	Intergenic space
1	121485038	T	G	1|0	9|0	0|1	0|0	1|0	Intergenic space
1	121485071	C	A	0|1	1|4	0|1	0|0	0|1	Intergenic space
1	121485074	T	C	0|1	5|0	1|0	0|0	1|0	Intergenic space
1	121485076	C	A	0|1	5|0	1|0	0|0	1|0	Intergenic space
1	121485241	G	C	2|0	22|5	2|3	0|0	3|4	Intergenic space
1	121485246	G	A	0|2	6|21	3|0	0|0	2|4	Intergenic space
1	121485248	A	G	0|2	20|7	3|0	0|0	6|0	Intergenic space
1	121485251	A	T	0|2	0|25	0|2	0|0	2|3	Intergenic space
1	121485277	T	G	0|1	36|0	2|0	0|0	8|0	Intergenic space
1	121485290	C	T	2|0	16|19	1|1	0|0	3|6	Intergenic space
1	121485379	C	T	2|2	1|20	0|0	0|0	1|2	Intergenic space
6	58776745	C	T	0|1	0|5	0|0	0|0	0|0	OCT4‐NANOG‐H3K27ac‐H3K4me1 hESC enhancer chr6:58776447–58777002
6	58776782	A	T	0|1	0|5	0|0	0|0	0|1	OCT4‐NANOG‐H3K27ac‐H3K4me1 hESC enhancer chr6:58776447–58777002
6	58776802	T	G	0|1	3|3	0|0	0|0	0|1	OCT4‐NANOG‐H3K27ac‐H3K4me1 hESC enhancer chr6:58776447–58777002
6	58776820	C	T	0|1	5|0	0|0	0|0	1|0	OCT4‐NANOG‐H3K27ac‐H3K4me1 hESC enhancer chr6:58776447–58777002
6	58777500	C	T	0|2	7|0	0|0	0|0	1|0	OCT4‐NANOG‐H3K27ac‐H3K4me1 hESC enhancer chr6:58777003–58777558
6	58777512	G	A	2|0	9|0	0|0	0|0	0|1	OCT4‐NANOG‐H3K27ac‐H3K4me1 hESC enhancer chr6:58777003–58777558
6	58777520	G	A	2|0	8|1	0|0	0|0	0|1	OCT4‐NANOG‐H3K27ac‐H3K4me1 hESC enhancer chr6:58777003–58777558
6	58777546	G	A	0|2	0|12	0|0	0|0	0|3	OCT4‐NANOG‐H3K27ac‐H3K4me1 hESC enhancer chr6:58777003–58777558
6	58777573	C	T	0|1	0|9	0|0	0|0	0|2	OCT4‐NANOG‐H3K27ac‐H3K4me1 hESC enhancer chr6:58777559–58778114
6	58778207	G	A	0|2	0|5	0|0	0|0	0|0	OCT4‐NANOG‐H3K27ac‐H3K4me1 hESC enhancer chr6:58778115–58778671
6	58778649	C	T	0|3	3|4	0|0	0|0	0|1	OCT4‐NANOG‐H3K27ac‐H3K4me1 hESC enhancer chr6:58778115–58778671
6	58778703	G	A	3|0	5|0	0|0	0|0	0|1	OCT4‐NANOG‐H3K27ac‐H3K4me1 hESC enhancer chr6:58778672–58779227
7	61969297	C	T	1|0	5|0	0|1	0|0	1|0	OCT4‐NANOG‐H3K27ac‐H3K4me1 hESC enhancer chr7:61968737–61969310
8	70602390	C	T	0|1	4|14	0|7	0|0	0|14	SLCO5A1
8	70602423	G	A	0|1	13|6	4|3	0|0	5|9	SLCO5A1
8	70602432	G	A	0|1	0|17	0|7	0|0	0|12	SLCO5A1
8	70602433	T	C	1|0	5|12	3|4	0|0	7|5	SLCO5A1
10	42385161	T	G	0|1	1|4	0|0	0|0	1|3	OCT4‐NANOG‐H3K27ac‐H3K4me1 hESC enhancer chr10:42385081–42386032 OR fragment chr10:42384956–42385701
10	42385182	C	T	1|2	5|0	0|0	0|0	4|0	OCT4‐NANOG‐H3K27ac‐H3K4me1 hESC enhancer chr10:42385081–42386032 OR fragment chr10:42384956–42385702
10	42385191	A	G	0|3	1|4	0|0	0|0	2|3	OCT4‐NANOG‐H3K27ac‐H3K4me1 hESC enhancer chr10:42385081–42386032 OR fragment chr10:42384956–42385703
10	42385236	A	T	1|2	4|5	0|0	0|1	3|2	OCT4‐NANOG‐H3K27ac‐H3K4me1 hESC enhancer chr10:42385081–42386032 OR fragment chr10:42384956–42385704
10	42385243	C	T	3|0	9|0	0|0	0|1	5|0	OCT4‐NANOG‐H3K27ac‐H3K4me1 hESC enhancer chr10:42385081–42386032 OR fragment chr10:42384956–42385705
10	42388371	G	C	1|0	5|0	0|0	0|0	0|2	OCT4‐NANOG‐H3K27ac‐H3K4me1 hESC enhancer chr10:42387937–42388888
10	42388379	T	A	1|0	5|0	0|0	0|0	0|2	OCT4‐NANOG‐H3K27ac‐H3K4me1 hESC enhancer chr10:42387937–42388889
10	42396152	C	T	0|1	0|10	0|0	0|0	0|2	OCT4‐NANOG‐H3K27ac‐H3K4me1 hESC enhancer chr10:42395555–42396506
10	42396207	A	C	0|1	0|9	0|0	0|0	0|1	OCT4‐NANOG‐H3K27ac‐H3K4me1 hESC enhancer chr10:42395555–42396506
10	42396817	G	A	0|2	0|9	0|0	0|0	0|0	OCT4‐NANOG‐H3K27ac‐H3K4me1 hESC enhancer chr10:42396507–42397457
10	42396840	A	G	0|2	0|11	0|0	0|0	0|0	OCT4‐NANOG‐H3K27ac‐H3K4me1 hESC enhancer chr10:42396507–42397457
10	42396862	T	C	0|2	2|13	0|0	0|0	0|0	OCT4‐NANOG‐H3K27ac‐H3K4me1 hESC enhancer chr10:42396507–42397457
10	42396883	A	C	0|2	0|17	0|0	0|0	0|0	OCT4‐NANOG‐H3K27ac‐H3K4me1 hESC enhancer chr10:42396507–42397457
10	42396909	TGG	TG	0|2	0|17	0|0	0|0	0|0	OCT4‐NANOG‐H3K27ac‐H3K4me1 hESC enhancer chr10:42396507–42397457
10	42396916	G	A	2|0	3|11	0|0	0|0	0|0	OCT4‐NANOG‐H3K27ac‐H3K4me1 hESC enhancer chr10:42396507–42397457
10	42396932	A	G	0|2	0|6	0|0	0|0	0|0	OCT4‐NANOG‐H3K27ac‐H3K4me1 hESC enhancer chr10:42396507–42397457
10	42599603	A	G	0|0	0|14	0|0	0|0	0|0	OCT4‐NANOG‐H3K27ac‐H3K4me1 hESC enhancer chr10:42599577–42600112
10	42599664	C	T	0|2	3|16	0|0	0|0	0|1	OCT4‐NANOG‐H3K27ac‐H3K4me1 hESC enhancer chr10:42599577–42600112
10	42599680	C	A	0|2	7|10	0|0	0|0	1|0	OCT4‐NANOG‐H3K27ac‐H3K4me1 hESC enhancer chr10:42599577–42600112
10	42599691	G	A	1|1	18|0	0|0	0|0	0|1	OCT4‐NANOG‐H3K27ac‐H3K4me1 hESC enhancer chr10:42599577–42600112
10	42599701	A	T	2|0	18|0	0|0	0|0	0|1	OCT4‐NANOG‐H3K27ac‐H3K4me1 hESC enhancer chr10:42599577–42600112
10	42599702	T	A	1|1	3|15	0|0	0|0	0|1	OCT4‐NANOG‐H3K27ac‐H3K4me1 hESC enhancer chr10:42599577–42600112
10	42599708	G	A	0|1	0|18	0|0	0|0	0|1	OCT4‐NANOG‐H3K27ac‐H3K4me1 hESC enhancer chr10:42599577–42600112
11	51579594	C	T	0|1	0|6	0|1	0|0	0|1	OCT4‐NANOG‐H3K27ac‐H3K4me1 hESC enhancer chr11:51579187–51579881
11	51579611	C	T	1|0	19|0	1|0	0|0	0|1	OCT4‐NANOG‐H3K27ac‐H3K4me1 hESC enhancer chr11:51579187–51579881
11	51579629	C	T	1|0	20|0	1|0	0|0	0|1	OCT4‐NANOG‐H3K27ac‐H3K4me1 hESC enhancer chr11:51579187–51579881
11	51579641	G	A	1|0	20|0	0|1	0|0	1|0	OCT4‐NANOG‐H3K27ac‐H3K4me1 hESC enhancer chr11:51579187–51579881
11	51579645	C	A	0|1	20|0	1|0	0|0	1|0	OCT4‐NANOG‐H3K27ac‐H3K4me1 hESC enhancer chr11:51579187–51579881
11	51579658	T	C	0|1	0|20	0|1	0|0	0|1	OCT4‐NANOG‐H3K27ac‐H3K4me1 hESC enhancer chr11:51579187–51579881
11	51579679	TG	T	0|1	0|17	0|1	0|0	0|1	OCT4‐NANOG‐H3K27ac‐H3K4me1 hESC enhancer chr11:51579187–51579881
17	22253128	C	T	0|1	0|8	0|0	0|0	0|0	OCT4‐NANOG‐H3K27ac‐H3K4me1 hESC enhancer chr17:22252628–22253215
17	22253134	C	T	0|1	0|11	0|0	0|0	0|1	OCT4‐NANOG‐H3K27ac‐H3K4me1 hESC enhancer chr17:22252628–22253215
17	22253139	G	T	0|1	5|4	0|0	0|0	0|0	OCT4‐NANOG‐H3K27ac‐H3K4me1 hESC enhancer chr17:22252628–22253215
17	22253207	T	C	1|0	8|8	0|0	0|0	0|1	OCT4‐NANOG‐H3K27ac‐H3K4me1 hESC enhancer chr17:22252628–22253215
17	22253220	T	C	0|1	12|3	0|0	0|0	1|0	OCT4‐NANOG‐H3K27ac‐H3K4me1 hESC enhancer chr17:22253216–22253802
17	22253231	C	T	0|1	0|8	0|0	0|0	0|0	OCT4‐NANOG‐H3K27ac‐H3K4me1 hESC enhancer chr17:22253216–22253802
18	109289	T	C	0|1	6|1	0|0	0|0	0|0	ROCK1P1 AND OCT4‐NANOG‐H3K27ac hESC enhancer chr18:108681–109468
18	109313	A	G	0|1	9|0	0|0	0|0	0|0	ROCK1P1 AND OCT4‐NANOG‐H3K27ac hESC enhancer chr18:108681–109468
18	109351	G	A	0|1	7|2	0|0	0|0	0|0	ROCK1P1 AND OCT4‐NANOG‐H3K27ac hESC enhancer chr18:108681–109468
18	109366	C	A	0|1	4|1	0|0	0|0	0|0	ROCK1P1 AND OCT4‐NANOG‐H3K27ac hESC enhancer chr18:108681–109468
19	27732052	T	C	0|3	0|12	0|0	0|0	0|0	Intergenic space
19	27732053	A	C	1|2	12|0	0|0	0|0	0|0	Intergenic space
19	27732058	A	C	1|2	10|2	0|0	0|0	0|0	Intergenic space
19	27732124	G	A	0|2	0|13	0|0	0|0	0|0	Intergenic space
19	27732126	T	C	0|1	3|7	0|0	0|0	0|0	Intergenic space

Of the genes known to have alleles that confer a predisposition to cancer (e.g., *BRCA1/2*) none were covered by the sequenced reads of our samples due to the poor aDNA preservation (Tables 3 and 4). This result does not fully exclude metastatic carcinoma as a possible diagnosis due to the low‐coverage of human genome, but as we lacked genomic data to identify any variants of BRCA1/2 and PALB2 the hypothesis of the observed bone lesions being caused by metastatic carcinoma cannot be ruled out.

**TABLE 4:** Exonic mutations identified in this study.

Chromosome	Position	Reference allele	Alternative allele	Region type	Gene	Exonic function	Comment
7	117374997	C	T	Exonic	CTTNBP2	Synonymous SNV	Cortactin binding protein 2, dendritic‐spine maintenance (Shih et al. 2022). No published cancer association
17	38183215	C	T	Exonic	MED24	Nonsynonymous SNV	MED24 transcription cofactor; the published MM‐risk variant rs2302777‐A is at chr17:38179492, > 3700 bp upstream and is a different mutation (Weinhold et al. 2013). Our variant does not support an MM diagnosis

### 3.4 Microbiome Metagenomics and the Diagenetic Background

Of the ~186 million reads per sample, between 6.94% and 12.32% (genus level; Table S1) and 6.17% to 11.76% (species level; Table S2) could be classified to existing microbial references.

**FIGURE 4:**
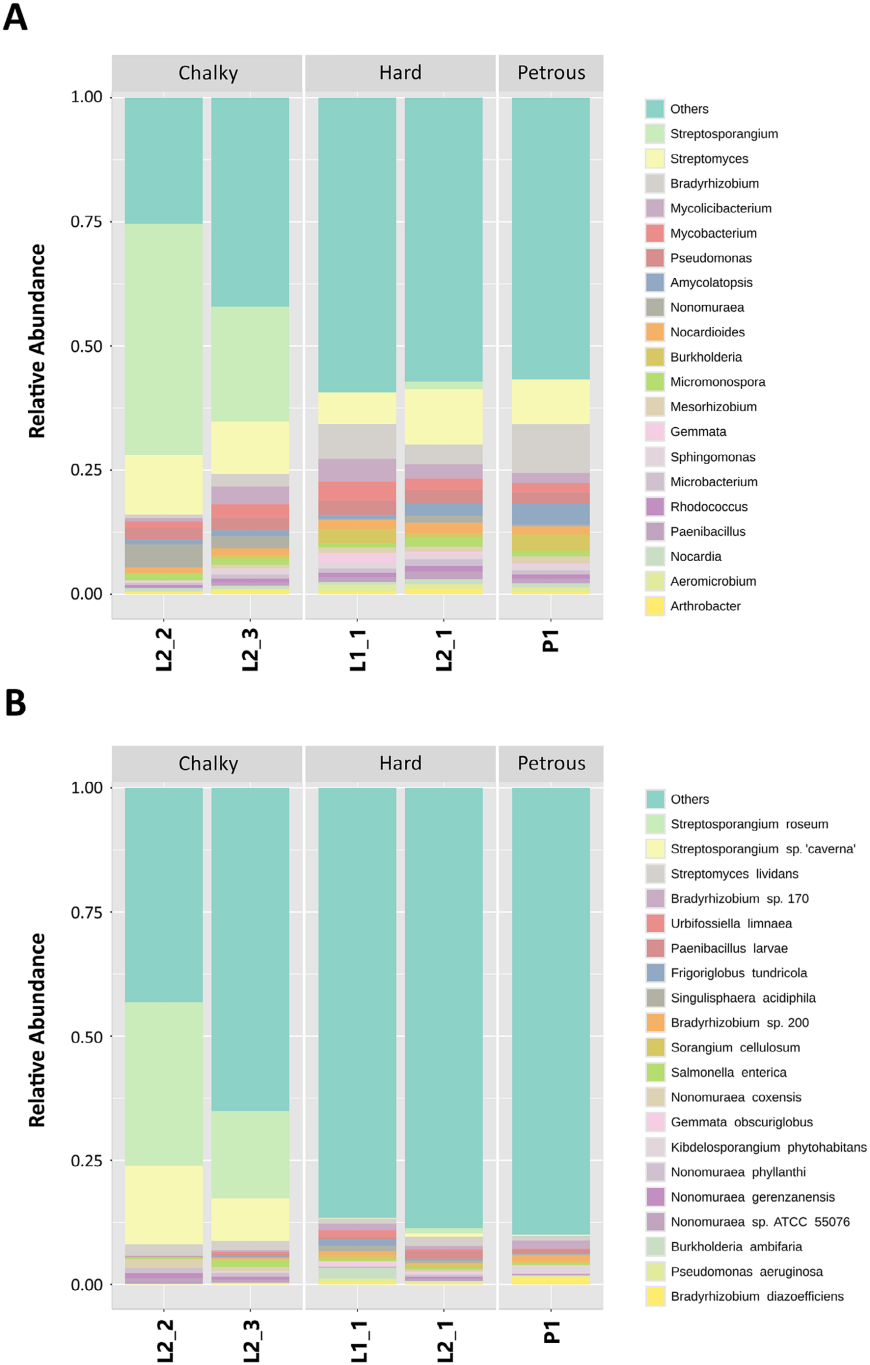
Microbiome composition by bone density. (A) Relative genus‐level abundance, top 20 represented genera. (B) Relative species‐level abundance, top 20 represented species. Samples grouped by macroscopic bone texture: Chalky (L2_2, L2_3), hard (L1_1, L2_1), petrous (P1). *Streptosporangium* (and *S. roseum* ) shows elevated abundance in chalky‐textured samples.

The principal pattern observed was an elevated abundance of *Streptosporangium* in the chalky‐textured samples (L2_2 and L2_3) at both genus and species levels (Figure 4). *Streptosporangium* is a soil‐dwelling, sporangium‐forming actinobacterial genus (Stackebrandt et al. 1994; Nolan et al. 2010), and *S. roseum* sequences with damage patterns characteristic of ancient DNA have previously been reported in Latvian archaeological human samples (Ķimsis et al. 2023). The pattern would be consistent with preferential colonisation of low‐density bone tissue by soil microorganisms and accords with experimental and archaeological observations linking elevated *Streptosporangium* abundance to bioeroded bone (Eriksen et al. 2020), but we emphasise that this remains an association rather than a demonstrated causal relationship.

Interpretation of this finding is problematic as the colonisation may have occurred before the loss of bone density (microbial bioerosion) or after it (microbial colonisation). In either case, the observed pattern would be consistent with pseudopathology concerns raised in Section 1.1. As the mentioned directionality cannot be resolved from abundance data alone (Eriksen et al. 2020), and the abundance of a soil actinobacterium does not by itself demonstrate bone destruction, the confirmation therefore requires histological assessment of microbial focal destruction in the same tissue (Schultz 2001; Hackett 1981; Turner‐Walker and Jans 2008). Two further metagenomic findings are worth noting. First, the *Mycobacterium* genus was strongly represented in metagenomic abundance plots (Figure 4), but the proportion of reads aligning specifically to *M. tuberculosis* by Kraken2 was modest (0.15%–0.28%; 1968–28,583 reads per sample; Table S2). This is consistent with high environmental *Mycobacterium* genus diversity in soils (Walsh et al. 2019) and motivates the more stringent direct‐alignment analysis of Section 3.5. Second, *Salmonella enterica* was among the most represented species (1842–246,323 reads per sample; Table 5), but stringent alignment retained only 119 reads (20 after PMD filtering), insufficient for further analysis. Salmonellosis is plausible in 10th‐century conditions but does not explain the lytic lesions; we report this finding but an in‐depth analysis of such diagnosis is outside the scope of this study.

**TABLE 5:** Sequencing and *Salmonella enterica* alignment metrics of the processed libraries.

Sample	Trimmed reads	Quality filtered reads before deduplication	Deduplicated quality filtered reads	Sequencing saturation	Proportion of *Salmonella enterica* aligning reads (%)	Reads with PMDscore ≥ 3	Proportion of damaged reads (%)
P1	325,270,973	46	10	0.783	0.000003	—	—
L1_1	186,932,153	71	47	0.338	0.000025	—	—
L2_1	157,741,889	44	30	0.318	0.000019	—	—
L2_2	21,179,118	0	0	—	—	—	—
L2_3	239,653,266	163	119	0.270	0.000050	20	16.807
B‐ex	1,727,725	84	19	0.774	0.001100	—	—
B‐lib	1,724,481	43	5	0.884	0.000290	—	—

### 3.5 Direct Alignment to *Mycobacterium tuberculosis*

Direct alignment to the H37Rv reference (AL123456.3) under conservative parameters (mapping quality ≥ 30) yielded 373–4839 reads per sample. After PMD filtering (PMDscore ≥ 3), 68–624 damaged reads per sample remained (Table 6). Visual inspection identified an excess of reads mapping to the 16S rRNA coding region (1,471,846–1,473,382 bp); after exclusion of this highly conserved region (which is also prone to cross‐mapping among environmental mycobacteria), sequencing‐depth metrics normalised across all samples (Table 6).

**TABLE 6:** Sequencing and *Mycobacterium tuberculosis* alignment metrics of the processed libraries.

Sample	Trimmed reads	Quality filtered reads before deduplication	Deduplicated quality filtered reads	Sequencing saturation	Proportion MTB aligning reads (%)	Reads with PMDscore ≥ 3	Proportion of damaged MTB reads (%)	Average sequencing depth (X) with16s rRNA region included	Average coverage (%) with16s rRNA region included	Reads after removing 16s rRNA coding region	Average sequencing depth (X) without 16s rRNA coding regions	Average coverage (%) without 16s rRNA coding region included
P1	325,270,973	11,685	449	0.962	0.0001	68	15.14	0.0014	0.069	18	0.00029	0.02761
L1_1	186,932,153	4947	2964	0.401	0.0016	269	9.08	0.0061	0.213	99	0.00178	0.1421
L2_1	157,741,889	6046	2674	0.558	0.0017	288	10.77	0.0066	0.130	47	0.00073	0.06068
L2_2	21,179,118	3183	373	0.883	0.0018	78	20.91	0.0014	0.035	3	0.00003	0.00331
L2_3	239,653,266	17,919	4839	0.730	0.0020	624	12.90	0.0147	0.230	141	0.00251	0.1516
B‐ex	1,727,725	83	28	0.663	0.0016	—	—	—	—	—	—	—
B‐lib	1,724,481	23	2	0.913	0.0001	—	—	—	—	—	—	—

#### 3.5.1 Authenticity of the Residual Signal as Assessed by Using the Defined Criteria

##### 3.5.1.1 Damage Proportion

The proportion of damaged reads in the *M. tuberculosis* alignment (9.08%–20.91%) was higher than in the human alignment (2.03%–12.39%; Tables 1 and 6), the opposite of what would be expected from modern contamination.

##### 3.5.1.2 Read Dispersion

After exclusion of the 16S region, residual reads were dispersed across the H37Rv genome rather than concentrated in conserved loci, consistent with authentic endogenous origin (Margaryan et al. 2018).

##### 3.5.1.3 Specific‐Gene Alignment

One read in sample L2_3 aligned to rpoB, an *M. tuberculosis* ‐complex‐specific gene used in clinical and ancient identification (Bos et al. 2014).

##### 3.5.1.4 Comparability With Confirmed Cases

Final coverage and depth are comparable to those of the shotgun data from confirmed *M. tuberculosis* ‐positive samples reported by Vågene et al. (2022) for South American pre‐contact populations. With an exception of L1_1 sequencing saturation value 40.1%, sequencing saturation of other samples (Table 6) suggests that further untargeted sequencing could not substantially improve the signal, showing the principal limitation to be preservation, not sequencing effort.

##### 3.5.1.5 Interpretation

The microbial pathogen aDNA channel provides weak but authentic‐looking support for tuberculosis. This is the first molecular line of evidence pointing to the possibility of TB infection in this individual; on its own it is insufficient for a confirmed diagnosis but, combined with the morphology of the C1 lesion, it is non‐trivial.

##### 3.5.1.6 Caveats

First, the data do not exclude the possibility that residual reads derive from post‐mortem colonisation by environmental mycobacteria with sufficient genomic similarity to *M. tuberculosis* to pass conservative alignment filters. In case of poor aDNA preservation, targeted in‐solution capture using *M. tuberculosis* ‐specific probes could provide a definitive resolution. Second, the absence of postcranial samples (apart from C1) limits inference about the form of TB.

### 3.6 Integrated Assessment of the Differential Diagnosis

The framework defined in Section 2.6 was applied to our results (Table 7).

**TABLE 7:** Assessment of the suggested diagnoses based on formulated hypotheses.

	A. Multiple myeloma	Evidence	B. Metastatic carcinoma	Evidence	C. Disseminated tuberculosis	Evidence
Cranial CT morphology	Sharply demarcated, uniformly small (< 3 cm), spherical lesions; smooth margins; uniformly effaced trabeculae (Rothschild et al. 1998)	Does not fit the exact characteristics, but cannot be excluded	Heterogeneously sized lesions (mm to multiple cm); irregular margins; resorption fronts; some perilesional density variation (Mutlu et al. 2021)	Consistent	Sharply punched‐out, fully transcortical lytic lesions; no periosteal reaction or sclerosis; possible craniocervical (atlanto‐axial) involvement (Volkmann type I; Raut et al. 2004; Lifeso 1987)	Does not fit the exact characteristics, but cannot be excluded
Postcranial distribution	Lytic foci predominantly in vertebral bodies, ribs, sternum, pelvis and proximal long bones (red marrow distribution; Kyle and Rajkumar 2008; Angtuaco et al. 2004)	Consistent, insufficient data	Lytic‐blastic foci in the axial skeleton with unilateral patchy distribution (Marks and Hamilton 2007)	Consistent	Possible Pott's disease of the vertebral column or extraspinal foci	Consistent, insufficient data
Pathogenic changes in human genomic alignment data	In hyperdiploid MM, detectable read‐depth deviations on chromosomes 3, 5, 7, 9, 11, 15, 19, 21 above the noise level established by control genomes (Smadja et al. 2001; Prideaux et al. 2014)	Contradicts	No characteristic aneuploidy pattern. Possible pathogenic variants in BRCA1/2, PALB2, or other breast‐cancer‐associated loci, given low coverage	Data absent	Chromosomal aneuploidies absent	Consistent
Microbial pathogen aDNA	Absence of pathogen‐specific aDNA signal	Consistent	Absence of pathogen‐specific aDNA signal	Consistent	Sequences alignable to *M. tuberculosis* complex with characteristic post‐mortem damage profiles; dispersion across the reference genome rather than concentration in conserved regions; damage proportions consistent with or exceeding host human DNA (Skoglund et al. 2014)	Consistent, data weak
Overall classification	Hyperdiploid—excluded; non‐hyperdiploid—weakened		Weakened		Supported	

While the most evidence supports disseminated tuberculosis as the leading cause of the observed lesions, a combination of considered diagnoses or pseudopathology cannot be fully excluded without additional evidence.

## 4 Discussion

### 4.1 Discordance Between CT and aDNA Streams

Our findings show discordance between the two principal evidence streams: CT favours metastatic carcinoma, while aDNA, through the combination of hyperdiploid‐MM exclusion and weak but authentic‐looking *M. tuberculosis* alignment, is more compatible with disseminated tuberculosis. Such discordance reflects three structural features of the case.

First, morphological convergence is genuine: calvarial perforating TB (Volkmann type I) and lytic metastases produce overlapping macroscopic appearances. Second, low aDNA preservation imposes informational asymmetries: chromosomal aneuploidies can be detected and excluded at low coverage, but translocations and SNVs cannot. Pathogen aDNA can be detected even at very low coverage but requires additional authentication. The two streams therefore have different sensitivity profiles for different diagnoses.

Third, the diagnoses are not mutually exclusive: metastatic carcinoma in a 40–50‐year‐old female and disseminated tuberculosis can co‐occur in the same individual. The C1 lesion morphology and the residual MTB‐like aDNA signal are compatible with at least coexisting TB; the cranial morphology and demographic profile are compatible with coexisting metastatic disease. We cannot exclude this possibility based on the present evidence.

### 4.2 Implications for the Multiple‐Myeloma Debate

Rothschild (2023) has argued that pre‐industrial MM cases may not represent true MM, and that on re‐examination most are better interpreted as metastatic carcinoma. The present study is consistent with that argument in two ways. First, the CT morphology of the cranial lesions, particularly the irregular margins and substantial size variability, fits Rothschild–Hershkovitz–Dutour (Rothschild et al. 1998) criteria for metastatic carcinoma rather than MM. Second, the genetic exclusion of hyperdiploid MM removes the most common MM subtype from consideration. The case therefore adds to the empirical basis for re‐examining the MM‐versus‐metastasis distinction in pre‐industrial populations.

To our knowledge, this is the first paleopathological case in which read‐depth‐based screening for MM‐characteristic chromosomal aneuploidies has been performed on shotgun aDNA data. The methodology is not novel in clinical genetics (Smadja et al. 2001; Prideaux et al. 2014) but its translation to paleopathology illustrates how the MM‐versus‐metastasis distinction can be approached molecularly even when full genetic analysis is precluded by preservation.

### 4.3 Tuberculosis in the Iron Age Eastern Baltic

Faerman and Jankauskas (2000) reported TB aDNA from Iron Age Lithuania at Marvelė; Bouwman et al. (2012), Bos et al. (2014) and Donoghue et al. (2015) have established the broader aDNA data context for TB in early‐medieval Europe. The Eastern Baltic of the 9th–11th centuries, a time of dense proto‐urban settlement at sites such as Daugmale, Grobiņa and Salaspils Laukskola, with active long‐distance trade with Scandinavia and the Volga–Byzantine route (Vasks 2018), is ecologically plausible for tuberculosis. The present case, if confirmed by targeted in‐solution capture, would represent the first molecular evaluation of TB in an Iron Age Latvian individual with multiple lytic cranial lesions and would be regionally informative even if the overall diagnosis remained ambiguous.

### 4.4 The Contribution of Cervical‐Spine Assessment

Inclusion of the C1 atlas in CT analysis was, in retrospect, the single most informative methodological extension. The right anterolateral C1 defect (discrete, sharply demarcated, fully transcortical, with no perilesional reaction) is morphologically the closest match to the Class 2 cranial lesions and demonstrates that the disease process, if antemortem, is not confined to the calvarium. The absence of available postcranial vertebral material limits further inference; future studies of similarly multifocal cranial archaeological cases should systematically include cervical and thoracolumbar vertebrae in CT analysis. The strength of the contribution from a single additional vertebra (albeit a small one) argues for routine extension of CT differential‐diagnostic analysis to the postcranial skeleton wherever possible.

### 4.5 Possible Indications of Pseudopathology

The high abundance of *Streptosporangium* in the metagenome results (Section 3.4) could be an indication that the observed lesions are pseudopathological. The abundant representation of *Streptosporangium* in metagenomic profiles would be in line with evidence of *Streptosporangium* being present in tested and post‐mortem environmentally affected bone tissue presented by Eriksen et al. (2020). As this study lacks a histological and X‐ray fluorescence spectrometric testing pseudopathological origin of the observed lesions cannot be properly supported or rejected, but based on our results, such analyses would be an important future research prospect.

### 4.6 Limitations

#### 4.6.1 Preservation

Mean human DNA coverage of 0.0013× is below the threshold at which variants predisposing to cancer (BRCA1/2, PALB2, etc.) can be confidently called; non‐hyperdiploid MM cannot be excluded; SNV oncogenetic analysis is therefore underpowered.

#### 4.6.2 Methodological Limitations

Pseudopathology still remains to be one of the possible explanations for the observed lesions, and a lack of histological or X‐ray fluorescence spectrometry does not allow us to prove or reject it.

#### 4.6.3 Lack of Targeted Enrichment

Shotgun‐only sequencing leaves the *M. tuberculosis* alignment at the threshold of conviction. Targeted in‐solution capture should be considered for validation.

#### 4.6.4 Postcranial Material

Beyond the C1 atlas, postcranial elements were not available for analysis. Vertebral‐body involvement is critical for distinguishing MM from metastases and for confirming Pott's disease for TB.

### 4.7 Recommendations for Future Work

- Targeted *M. tuberculosis* in‐solution capture from the existing aDNA extracts, with subsequent genomic reconstruction. A positive result with confirmed authenticity would establish disseminated TB; a negative result would consolidate metastatic carcinoma as the leading diagnosis. In‐solution capture of breast‐cancer‐predisposition loci (BRCA1/2, PALB2) could also be performed for higher‐coverage oncogenetic interrogation
- Histological and X‐ray fluorescence spectrometry authentication of cranial and C1 lesion margins per the Schultz (2001), Hackett (1981) and Turner‐Walker and Jans (2008) protocols, with explicit search for Wedl tunnels and microbial focal destruction
- Mycolic‐acid analysis of bone lipids per Lee et al. (2012) to provide an aDNA‐independent biomarker line

## 5 Conclusions

Integrating high‐resolution CT with deep shotgun aDNA sequencing in a single 10th‐century Latvian individual presenting with multiple lytic cranial lesions yields a partly‐resolved differential diagnosis: hyperdiploid multiple myeloma is excluded by chromosomal‐aneuploidy screening; cranial CT morphology favours metastatic carcinoma; the combination of C1 morphology and a weak but authentic‐looking *M. tuberculosis* aDNA signal supports disseminated tuberculosis as a serious alternative; pseudopathology could not be confirmed and requires histological study. Metastatic carcinoma and disseminated tuberculosis emerge as the leading non‐mutually exclusive hypotheses, with different evidence profiles on different channels.

The principal methodological contribution of the study is the demonstration that, even at low overall preservation, the integration of CT and aDNA can constrain the differential diagnosis of multifocal lytic lesions in archaeological individuals, and that single‐stream assessment can be misleading.

## Author Contributions

**Alise Akermane‐Pokšāne:** data curation (equal), formal analysis (equal), funding acquisition (equal), investigation (equal), methodology (equal), project administration (equal), visualization (equal), writing – original draft (equal), writing – review and editing (equal). **Jānis Ķimsis:** data curation (equal), formal analysis (equal), investigation (equal), methodology (equal), visualization (equal), writing – original draft (equal), writing – review and editing (equal). **Elīna Pētersone‐Gordina:** resources (equal), writing – review and editing (equal). **Antonija Vilcāne:** resources (equal), writing – review and editing (equal). **Guntis Gerhards:** funding acquisition (equal), resources (equal), writing – review and editing (equal). **Mārtiņš Pēterfelds:** formal analysis (equal), investigation (equal), methodology (equal), visualization (equal), writing – review and editing (equal). **Renāte Ranka:** conceptualization (equal), funding acquisition (equal), methodology (equal), resources (equal), supervision (equal), writing – original draft (equal), writing – review and editing (equal).

## Funding

The genetic research of this study was funded within the framework of the European Union's Recovery and Resilience Mechanism project No. 5.2.1.1.i.0/2/24/I/CFLA/001 ‘Consolidation of the Latvian Institute of Organic Synthesis and the Latvian Biomedical Research and Study Centre’. The study was also supported by the Latvian Council of Science, project No. lzp‐2022/1‐0059. The funding bodies did not participate in the designing of the study; sample collection, analysis, data interpretation and creating the manuscript.

## Conflicts of Interest

The authors declare no conflicts of interest.

## Supporting information


**Table S1:** Kraken2 metagenomic alignment genus level results.

**Table S2:** Kraken2 metagenomic alignment species level results.

## Data Availability

Raw sequencing reads have been deposited in the European Nucleotide Archive under project accession PRJEB94441 (https://www.ebi.ac.uk/ena/browser/view/PRJEB94441).
