# Supplementary material for: Multidisciplinary Exploration of Computed‐Tomographic and Ancient‐DNA Data of an Iron Age Skull From Latvia With Multiple Lytic Bone Lesions: Differential Diagnosis Between Metastatic Carcinoma, Multiple Myeloma and Skeletal Tuberculosis
Source: Ecol Evol. 2026 Jul 23;16(7):e74058. doi: 10.1002/ece3.74058 (PMC13395577; doi:10.1002/ece3.74058)
Supplement: Supplementary file 1 — Table S1: Kraken2 metagenomic alignment genus level results. [file ECE3-16-e74058-s001.docx]

**Table S1** Kraken2 metagenomic alignment genus level results.

| **Sample name** | **P1** | **L1_1** | **L2_1** | **L2_2** | **L2_3** |
| --- | --- | --- | --- | --- | --- |
| **Deduplicated input reads** | **84369632** | **152006844** | **129149404** | **5964256** | **189847984** |
| **Total assigned reads on genera level** | **8574933** | **10542690** | **11729028** | **734792** | **22072365** |
| **Total assigned read proportion (%)** | **10.16** | **6.94** | **9.08** | **12.32** | **11.63** |
| Azorhizobium | 3593 | 2575 | 2802 | 19 | 4069 |
| Cellvibrio | 584 | 1441 | 916 | 93 | 1050 |
| Dictyoglomus | 38 | 147 | 84 | 0 | 101 |
| Methylophilus | 0 | 0 | 0 | 0 | 0 |
| Pelobacter | 1231 | 2718 | 2822 | 14 | 2618 |
| Phenylobacterium | 10367 | 21214 | 13012 | 198 | 19908 |
| Shewanella | 3573 | 7184 | 7113 | 544 | 7910 |
| Myxococcus | 9118 | 19144 | 29189 | 355 | 22665 |
| Stigmatella | 3216 | 5802 | 5749 | 115 | 8234 |
| Cystobacter | 3495 | 6002 | 5747 | 105 | 7939 |
| Melittangium | 2526 | 4433 | 4623 | 32 | 5648 |
| Archangium | 9264 | 18693 | 19693 | 274 | 22797 |
| Chondromyces | 3382 | 6111 | 8473 | 77 | 6998 |
| Nannocystis | 6146 | 11495 | 11538 | 268 | 13580 |
| Polyangium | 5911 | 13700 | 15288 | 202 | 13903 |
| Vitreoscilla | 725 | 886 | 930 | 17 | 1442 |
| Lysobacter | 57735 | 62855 | 64892 | 1079 | 65118 |
| Simonsiella | 4 | 19 | 13 | 0 | 90 |
| Caulobacter | 22855 | 38578 | 30836 | 0 | 42338 |
| Hyphomicrobium | 31140 | 14846 | 15589 | 267 | 14927 |
| Hyphomonas | 2684 | 3083 | 2887 | 72 | 4140 |
| Leptothrix | 2364 | 3085 | 3056 | 88 | 5030 |
| Stella | 6561 | 5386 | 6444 | 311 | 10071 |
| Gallionella | 0 | 0 | 0 | 0 | 0 |
| Ancylobacter | 15595 | 10407 | 11215 | 290 | 16934 |
| Runella | 427 | 816 | 715 | 5 | 789 |
| Spirosoma | 2043 | 6992 | 5687 | 44 | 6702 |
| Gemmata | 19662 | 199360 | 77114 | 520 | 94649 |
| Planctomyces | 10945 | 50645 | 27550 | 392 | 42380 |
| Pirellula | 1931 | 6262 | 5636 | 71 | 8757 |
| Isosphaera | 1063 | 5371 | 3234 | 40 | 5060 |
| Methylocystis | 11997 | 11023 | 10089 | 205 | 15207 |
| Borrelia | 74 | 85 | 69 | 0 | 123 |
| Spirochaeta | 521 | 1035 | 1120 | 8 | 1307 |
| Treponema | 864 | 2221 | 1438 | 0 | 1995 |
| Leptospira | 404 | 963 | 783 | 12 | 992 |
| Leptospirillum | 0 | 0 | 0 | 0 | 0 |
| Aquaspirillum | 321 | 623 | 537 | 9 | 974 |
| Azospirillum | 28796 | 34034 | 34392 | 710 | 51834 |
| Campylobacter | 736 | 1169 | 1055 | 12 | 1466 |
| Helicobacter | 368 | 1064 | 676 | 19 | 1949 |
| Achromobacter | 29892 | 35116 | 42166 | 853 | 58901 |
| Alteromonas | 734 | 978 | 1103 | 63 | 1422 |
| Brucella | 11859 | 9095 | 9434 | 64 | 10889 |
| Flavobacterium | 2700 | 81864 | 12109 | 58 | 10801 |
| Francisella | 244 | 249 | 407 | 14 | 311 |
| Paracoccus | 27438 | 31320 | 31981 | 259 | 45535 |
| Thermus | 3169 | 4972 | 6542 | 0 | 7749 |
| Xanthobacter | 11824 | 10346 | 10238 | 340 | 14617 |
| Comamonas | 13278 | 21135 | 20725 | 355 | 29322 |
| Pseudomonas | 192597 | 297316 | 288320 | 19046 | 555318 |
| Xanthomonas | 46051 | 99037 | 62580 | 0 | 61871 |
| Azotobacter | 2548 | 3840 | 3977 | 120 | 5112 |
| Agrobacterium | 20576 | 21564 | 17721 | 563 | 26473 |
| Bradyrhizobium | 843134 | 732797 | 471529 | 5391 | 560957 |
| Rhizobium | 73905 | 93783 | 73744 | 1403 | 115040 |
| Methylobacillus | 0 | 0 | 0 | 0 | 0 |
| Methylobacterium | 56583 | 57859 | 59752 | 1332 | 82670 |
| Methylococcus | 4471 | 7576 | 6796 | 150 | 8554 |
| Methylomonas | 2627 | 4991 | 4300 | 201 | 5365 |
| Methylosinus | 4753 | 4547 | 4157 | 78 | 6130 |
| Methylobacter | 621 | 1120 | 911 | 26 | 1447 |
| Acetobacter | 2246 | 2958 | 4337 | 36 | 4112 |
| Gluconobacter | 1356 | 2126 | 1654 | 116 | 2890 |
| Legionella | 1181 | 1690 | 1289 | 64 | 1751 |
| Fluoribacter | 29 | 37 | 33 | 1 | 21 |
| Tatlockia | 74 | 44 | 43 | 1 | 52 |
| Acinetobacter | 6321 | 5849 | 4348 | 135 | 4952 |
| Neisseria | 3562 | 5195 | 4544 | 137 | 8719 |
| Thermomicrobium | 692 | 1179 | 1469 | 18 | 1708 |
| Alcaligenes | 2765 | 2554 | 2128 | 0 | 3194 |
| Bordetella | 26593 | 48672 | 49919 | 528 | 117725 |
| Acidiphilium | 3536 | 4455 | 5112 | 18 | 6016 |
| Ochrobactrum | 0 | 6 | 10 | 0 | 2 |
| Beijerinckia | 971 | 896 | 775 | 3 | 1438 |
| Chromobacterium | 6307 | 9337 | 9440 | 263 | 14054 |
| Eikenella | 244 | 449 | 454 | 3 | 672 |
| Zymomonas | 81 | 169 | 962 | 43 | 299 |
| Citrobacter | 4637 | 9243 | 7509 | 343 | 8571 |
| Enterobacter | 22172 | 40095 | 34951 | 399 | 34306 |
| Erwinia | 1400 | 7501 | 2825 | 73 | 3463 |
| Escherichia | 14171 | 21354 | 16498 | 808 | 18133 |
| Hafnia | 0 | 0 | 0 | 0 | 0 |
| Klebsiella | 0 | 0 | 0 | 0 | 0 |
| Kluyvera | 615 | 757 | 608 | 49 | 637 |
| Morganella | 427 | 950 | 822 | 22 | 1269 |
| Proteus | 0 | 0 | 0 | 0 | 0 |
| Providencia | 319 | 1033 | 1145 | 73 | 980 |
| Salmonella | 29417 | 48995 | 36084 | 2705 | 249994 |
| Serratia | 9583 | 11975 | 10765 | 175 | 13871 |
| Shigella | 2867 | 3549 | 4266 | 32 | 2496 |
| Xenorhabdus | 340 | 489 | 1178 | 6 | 1215 |
| Yersinia | 6410 | 5240 | 4476 | 99 | 3934 |
| Edwardsiella | 1017 | 2458 | 1978 | 10 | 2310 |
| Aeromonas | 17673 | 22911 | 25249 | 916 | 30388 |
| Photobacterium | 940 | 1611 | 1470 | 105 | 2039 |
| Vibrio | 5165 | 9883 | 9455 | 530 | 10325 |
| Plesiomonas | 238 | 304 | 335 | 2 | 284 |
| Pasteurella | 1177 | 1984 | 2648 | 36 | 971 |
| Anaplasma | 58 | 135 | 164 | 9 | 158 |
| Bartonella | 679 | 896 | 601 | 17 | 819 |
| Coxiella | 195 | 591 | 437 | 3 | 462 |
| Rickettsia | 0 | 0 | 0 | 0 | 0 |
| Symbiopectobacterium | 0 | 0 | 0 | 0 | 0 |
| Chlamydia | 159 | 290 | 214 | 13 | 225 |
| Bacteroides | 1568 | 2896 | 2412 | 14 | 3344 |
| Butyrivibrio | 0 | 0 | 0 | 0 | 0 |
| Fibrobacter | 0 | 0 | 0 | 0 | 0 |
| Porphyromonas | 320 | 949 | 774 | 20 | 880 |
| Prevotella | 1056 | 2553 | 2027 | 0 | 2663 |
| Roseburia | 0 | 0 | 0 | 0 | 0 |
| Wolinella | 28 | 69 | 81 | 1 | 97 |
| Oxalobacter | 0 | 0 | 0 | 0 | 0 |
| Syntrophomonas | 20 | 80 | 153 | 0 | 184 |
| Pectinatus | 0 | 0 | 0 | 0 | 0 |
| Dichelobacter | 42 | 76 | 62 | 0 | 58 |
| Desulfovibrio | 3384 | 6620 | 6839 | 157 | 8878 |
| Desulfuromonas | 2491 | 5126 | 5357 | 49 | 6544 |
| Desulfobulbus | 891 | 2053 | 2023 | 40 | 2822 |
| Desulfococcus | 490 | 1023 | 962 | 58 | 1496 |
| Desulfomicrobium | 863 | 1904 | 1801 | 20 | 1773 |
| Acidaminococcus | 0 | 0 | 0 | 0 | 0 |
| Megasphaera | 899 | 1836 | 1499 | 33 | 1913 |
| Nitrobacter | 7796 | 4699 | 4023 | 272 | 6167 |
| Nitrosomonas | 714 | 2033 | 1110 | 31 | 1894 |
| Thiobacillus | 1756 | 2492 | 2629 | 86 | 4517 |
| Thiomicrospira | 132 | 310 | 254 | 56 | 336 |
| Hydrogenobacter | 0 | 0 | 0 | 0 | 0 |
| Ehrlichia | 0 | 0 | 0 | 0 | 0 |
| Wolbachia | 0 | 0 | 0 | 0 | 0 |
| Bdellovibrio | 402 | 1055 | 707 | 22 | 1233 |
| Herbaspirillum | 7382 | 9085 | 9333 | 261 | 16657 |
| Selenomonas | 1625 | 3494 | 3472 | 61 | 4986 |
| Cytophaga | 31 | 183 | 139 | 0 | 173 |
| Saprospira | 0 | 0 | 0 | 0 | 0 |
| Weeksella | 0 | 0 | 0 | 0 | 0 |
| Capnocytophaga | 375 | 1153 | 585 | 0 | 800 |
| Beggiatoa | 0 | 0 | 0 | 0 | 0 |
| Thiothrix | 603 | 1272 | 936 | 64 | 1370 |
| Afipia | 19812 | 13621 | 11011 | 129 | 11854 |
| Erythrobacter | 9689 | 11596 | 10813 | 329 | 16630 |
| Ectothiorhodospira | 1008 | 1528 | 1494 | 27 | 1882 |
| Thiocapsa | 1334 | 2225 | 2019 | 26 | 2970 |
| Rhodobacter | 4066 | 5666 | 5770 | 141 | 7647 |
| Rhodomicrobium | 2547 | 1777 | 1821 | 24 | 2150 |
| Rhodopseudomonas | 38070 | 25784 | 23489 | 616 | 34242 |
| Rhodospirillum | 4274 | 4500 | 4531 | 159 | 7235 |
| Chlorobium | 0 | 0 | 0 | 0 | 0 |
| Pelodictyon | 315 | 910 | 757 | 13 | 912 |
| Prosthecochloris | 452 | 1380 | 1268 | 4 | 1877 |
| Chloroflexus | 630 | 1998 | 1531 | 31 | 2331 |
| Porphyrobacter | 2444 | 3051 | 2938 | 73 | 4761 |
| Microcystis | 277 | 1376 | 882 | 30 | 1258 |
| Synechococcus | 7286 | 15009 | 14469 | 317 | 18994 |
| Synechocystis | 71 | 242 | 252 | 0 | 366 |
| Pseudanabaena | 107 | 363 | 264 | 11 | 342 |
| Oscillatoria | 0 | 0 | 0 | 0 | 0 |
| Anabaena | 676 | 2214 | 1189 | 0 | 1986 |
| Nostoc | 1937 | 8774 | 5076 | 97 | 7987 |
| Calothrix | 1231 | 3417 | 2111 | 33 | 3378 |
| Fischerella | 224 | 646 | 359 | 4 | 1060 |
| Phormidium | 82 | 321 | 225 | 8 | 255 |
| Scytonema | 398 | 1454 | 897 | 34 | 1484 |
| Nitrosococcus | 374 | 1280 | 857 | 3 | 1079 |
| Nitrospira | 9459 | 17684 | 18170 | 113 | 17261 |
| Piscirickettsia | 56 | 74 | 63 | 40 | 99 |
| Peptostreptococcus | 4 | 6 | 8 | 0 | 6 |
| Ruminococcus | 154 | 435 | 498 | 3 | 520 |
| Sarcina | 39 | 17 | 20 | 0 | 21 |
| Micrococcus | 5606 | 5692 | 8530 | 693 | 13612 |
| Deinococcus | 14285 | 25701 | 27372 | 541 | 37500 |
| Streptococcus | 2212 | 3070 | 9022 | 0 | 2614 |
| Enterococcus | 4228 | 2033 | 1778 | 55 | 1609 |
| Lactococcus | 0 | 0 | 0 | 0 | 0 |
| Planococcus | 1465 | 1966 | 1445 | 0 | 1804 |
| Atopobium | 113 | 409 | 254 | 1 | 361 |
| Bacillus | 51978 | 49492 | 42346 | 947 | 47226 |
| Clostridium | 2609 | 2385 | 2362 | 2496 | 2955 |
| Desulfotomaculum | 90 | 104 | 207 | 1 | 318 |
| Sporosarcina | 209 | 1051 | 537 | 129 | 880 |
| Clavibacter | 8677 | 10515 | 17190 | 420 | 18854 |
| Lactobacillus | 674 | 887 | 1594 | 5 | 1612 |
| Listeria | 7375 | 3770 | 2221 | 642 | 6346 |
| Renibacterium | 182 | 249 | 298 | 11 | 348 |
| Actinomyces | 11779 | 14898 | 20766 | 887 | 29354 |
| Arthrobacter | 37683 | 61772 | 126271 | 3220 | 177474 |
| Bifidobacterium | 7816 | 11840 | 14465 | 713 | 19036 |
| Brevibacterium | 15299 | 17551 | 25131 | 848 | 32992 |
| Cellulomonas | 40204 | 45546 | 66701 | 1916 | 79963 |
| Corynebacterium | 43162 | 46017 | 69154 | 2462 | 89681 |
| Eubacterium | 133 | 563 | 629 | 4 | 602 |
| Thermodesulfobacterium | 66 | 140 | 115 | 0 | 125 |
| Propionibacterium | 3403 | 4383 | 6553 | 202 | 7709 |
| Thermoanaerobacter | 159 | 192 | 171 | 8 | 173 |
| Mycobacterium | 164186 | 401907 | 280219 | 8489 | 608827 |
| Amycolatopsis | 361026 | 89154 | 308882 | 6599 | 253620 |
| Nocardia | 69965 | 59023 | 114504 | 4398 | 139901 |
| Rhodococcus | 76017 | 92999 | 124714 | 4285 | 173637 |
| Saccharopolyspora | 53200 | 22368 | 57388 | 2525 | 69404 |
| Nocardioides | 131475 | 190529 | 240225 | 8108 | 281290 |
| Pseudonocardia | 65564 | 50604 | 124598 | 3152 | 131710 |
| Actinopolyspora | 2434 | 1430 | 3166 | 107 | 3766 |
| Saccharomonospora | 29965 | 11279 | 33128 | 1001 | 31871 |
| Frankia | 16634 | 15888 | 27960 | 1530 | 42892 |
| Geodermatophilus | 3692 | 4751 | 6473 | 185 | 8639 |
| Dermatophilus | 307 | 231 | 469 | 7 | 734 |
| Actinoplanes | 47783 | 31787 | 86819 | 3304 | 99912 |
| Micromonospora | 97053 | 75556 | 213754 | 10642 | 267372 |
| Streptomyces | 766502 | 669441 | 1304601 | 88325 | 2332955 |
| Actinomadura | 37027 | 27816 | 62266 | 8517 | 160505 |
| Streptosporangium | 16113 | 11811 | 177794 | 342044 | 5105965 |
| Nocardiopsis | 12271 | 10881 | 21345 | 3523 | 57446 |
| Thermomonospora | 9188 | 6423 | 16927 | 3983 | 65371 |
| Thermoactinomyces | 31 | 200 | 151 | 6 | 205 |
| Kibdelosporangium | 111732 | 14967 | 32524 | 313 | 43397 |
| Curtobacterium | 12980 | 15505 | 21504 | 700 | 26525 |
| Tropheryma | 9 | 24 | 51 | 4 | 117 |
| Aeromicrobium | 77866 | 142022 | 109812 | 1111 | 60999 |
| Pimelobacter | 7559 | 10602 | 12815 | 409 | 14251 |
| Gordonia | 34937 | 37055 | 54273 | 1780 | 74194 |
| Sphaerobacter | 2903 | 4090 | 5286 | 60 | 6319 |
| Tsukamurella | 8784 | 9123 | 13508 | 382 | 17650 |
| Kitasatospora | 19328 | 16767 | 28652 | 2500 | 51450 |
| Saccharothrix | 104204 | 28201 | 63783 | 1794 | 50486 |
| Sporolactobacillus | 73 | 136 | 123 | 0 | 91 |
| Ureaplasma | 1 | 8 | 8 | 0 | 14 |
| Spiroplasma | 0 | 0 | 0 | 0 | 0 |
| Acholeplasma | 21 | 26 | 41 | 0 | 41 |
| Desulfobacter | 0 | 0 | 0 | 0 | 0 |
| Desulfosarcina | 1820 | 3954 | 3887 | 36 | 5053 |
| Halanaerobium | 20 | 39 | 41 | 0 | 180 |
| Thermotoga | 140 | 316 | 432 | 0 | 654 |
| Haliscomenobacter | 187 | 472 | 311 | 1 | 390 |
| Flexistipes | 0 | 0 | 0 | 0 | 0 |
| Desulfomonile | 260 | 624 | 506 | 0 | 875 |
| Xylella | 265 | 634 | 451 | 21 | 450 |
| Sporomusa | 103 | 176 | 226 | 4 | 172 |
| Thermosipho | 23 | 172 | 113 | 0 | 126 |
| Fervidobacterium | 53 | 99 | 89 | 0 | 157 |
| Teredinibacter | 0 | 0 | 0 | 0 | 0 |
| Roseobacter | 986 | 1278 | 910 | 10 | 1366 |
| Aquifex | 15 | 63 | 54 | 5 | 77 |
| Cardiobacterium | 683 | 924 | 932 | 4 | 980 |
| Hirschia | 68 | 74 | 36 | 1 | 60 |
| Symbiobacterium | 1202 | 2114 | 2450 | 61 | 3110 |
| Verrucomicrobium | 2890 | 7342 | 6189 | 39 | 7647 |
| Marinobacter | 5815 | 7967 | 7946 | 281 | 8577 |
| Halomonas | 20368 | 29353 | 30121 | 769 | 40869 |
| Carnobacterium | 0 | 0 | 0 | 0 | 0 |
| Acidovorax | 22965 | 34298 | 35115 | 204 | 52897 |
| Azoarcus | 6572 | 9382 | 10029 | 98 | 15398 |
| Dactylococcopsis | 46 | 113 | 200 | 0 | 97 |
| Magnetospirillum | 6376 | 6738 | 6743 | 132 | 10869 |
| Suttonella | 36 | 66 | 58 | 4 | 75 |
| Sphingomonas | 99601 | 124237 | 104141 | 1730 | 192058 |
| Thiocystis | 1033 | 1906 | 1589 | 58 | 1908 |
| Sulfobacillus | 1 | 10 | 2 | 0 | 11 |
| Acidothermus | 694 | 1101 | 1500 | 222 | 3604 |
| Lachnospira | 37 | 19 | 27 | 0 | 66 |
| Rhodoferax | 8717 | 13985 | 13357 | 224 | 20493 |
| Rubrivivax | 2683 | 3933 | 4309 | 157 | 6706 |
| Gloeothece | 48 | 197 | 157 | 2 | 207 |
| Lyngbya | 0 | 2 | 2 | 2 | 0 |
| Phyllobacterium | 44537 | 25234 | 14130 | 154 | 25039 |
| Sinorhizobium | 20314 | 22086 | 18621 | 277 | 26065 |
| Acetohalobium | 8 | 32 | 43 | 0 | 50 |
| Arcobacter | 0 | 0 | 0 | 0 | 0 |
| Chelatococcus | 7770 | 6393 | 6745 | 233 | 9211 |
| Desulfobacula | 61 | 147 | 96 | 0 | 104 |
| Colwellia | 0 | 0 | 0 | 0 | 0 |
| Geobacter | 5261 | 11365 | 10260 | 155 | 13414 |
| Petrotoga | 15 | 32 | 27 | 0 | 39 |
| Ornithobacterium | 69 | 107 | 89 | 1 | 110 |
| Marinomonas | 0 | 0 | 0 | 0 | 0 |
| Thermodesulfovibrio | 51 | 90 | 113 | 0 | 145 |
| Sphingobacterium | 995 | 2139 | 1611 | 27 | 2171 |
| Hydrogenovibrio | 68 | 268 | 233 | 11 | 201 |
| Thermoanaerobacterium | 91 | 124 | 172 | 0 | 132 |
| Planobispora | 13 | 2 | 1 | 0 | 2 |
| Alicyclobacillus | 2228 | 5033 | 4717 | 118 | 6396 |
| Amphibacillus | 3 | 4 | 16 | 0 | 6 |
| Microlunatus | 9886 | 12331 | 19462 | 738 | 26858 |
| Rhodoplanes | 23368 | 15118 | 16498 | 444 | 40646 |
| Syntrophobacter | 781 | 1601 | 1647 | 21 | 2014 |
| Rhodothermus | 1949 | 5006 | 4814 | 43 | 6113 |
| Taylorella | 18 | 25 | 59 | 4 | 155 |
| Janthinobacterium | 6824 | 11155 | 10193 | 119 | 59171 |
| Helcococcus | 12 | 11 | 18 | 0 | 6 |
| Aminobacter | 23857 | 15849 | 16683 | 178 | 19134 |
| Burkholderia | 284511 | 309882 | 104598 | 0 | 164387 |
| Thiomonas | 2557 | 4460 | 4109 | 66 | 5802 |
| Iodobacter | 133 | 485 | 269 | 2 | 659 |
| Sebaldella | 53 | 24 | 45 | 0 | 39 |
| Rothia | 2366 | 3524 | 5194 | 423 | 8502 |
| Kingella | 448 | 798 | 599 | 32 | 1118 |
| Halothermothrix | 42 | 56 | 70 | 1 | 74 |
| Phascolarctobacterium | 0 | 0 | 0 | 0 | 0 |
| Coprococcus | 0 | 0 | 0 | 0 | 0 |
| Candidatus Kinetoplastibacterium | 0 | 0 | 0 | 0 | 0 |
| Thauera | 14172 | 18188 | 21768 | 171 | 32593 |
| Gloeobacter | 2170 | 4857 | 4255 | 41 | 5872 |
| Zymobacter | 155 | 351 | 222 | 1 | 297 |
| Coriobacterium | 251 | 370 | 497 | 2 | 536 |
| Agromyces | 32990 | 32555 | 56530 | 782 | 63306 |
| Microbacterium | 71542 | 92735 | 166731 | 3534 | 176668 |
| Rathayibacter | 10746 | 13336 | 25553 | 542 | 23207 |
| Candidatus Phytoplasma | 17 | 33 | 59 | 2 | 61 |
| Acetobacterium | 155 | 427 | 324 | 1 | 563 |
| Melissococcus | 1 | 11 | 3 | 0 | 6 |
| Acidobacterium | 1249 | 3941 | 2783 | 30 | 3544 |
| Kineococcus | 2307 | 2729 | 3679 | 277 | 6560 |
| Exiguobacterium | 812 | 1900 | 1676 | 23 | 2347 |
| Neorickettsia | 29 | 105 | 45 | 0 | 72 |
| Rhodovulum | 2648 | 3196 | 3108 | 90 | 4253 |
| Liberibacter | 285 | 168 | 164 | 31 | 457 |
| Rahnella | 3510 | 2237 | 1285 | 61 | 1747 |
| Cycloclasticus | 79 | 87 | 113 | 7 | 79 |
| Variovorax | 58846 | 79866 | 73761 | 812 | 104341 |
| Riemerella | 0 | 0 | 0 | 0 | 0 |
| Simkania | 73 | 68 | 79 | 0 | 87 |
| Sphaerotilus | 5127 | 7138 | 8199 | 91 | 11487 |
| Dactylosporangium | 25501 | 20043 | 54238 | 5422 | 99785 |
| Nitrosospira | 1185 | 14192 | 2139 | 41 | 3393 |
| Roseococcus | 1951 | 2159 | 2116 | 22 | 3200 |
| Arthrospira | 0 | 11 | 46 | 0 | 13 |
| Acetivibrio | 89 | 557 | 185 | 4 | 188 |
| Dermabacter | 1653 | 2374 | 3031 | 82 | 2941 |
| Desulfitobacterium | 310 | 435 | 381 | 14 | 894 |
| Ideonella | 2292 | 2522 | 3057 | 14 | 4990 |
| Dietzia | 9376 | 8790 | 13949 | 533 | 16651 |
| Blastococcus | 7026 | 8279 | 10989 | 414 | 14011 |
| Sorangium | 26414 | 65407 | 84030 | 968 | 62903 |
| Methylomicrobium | 1135 | 1935 | 1688 | 6 | 2152 |
| Actinokineospora | 27490 | 5058 | 24334 | 524 | 14725 |
| Dialister | 162 | 655 | 367 | 8 | 638 |
| Methylophaga | 0 | 0 | 0 | 0 | 0 |
| Stenotrophomonas | 31460 | 36549 | 38120 | 520 | 49938 |
| Sutterella | 1024 | 1458 | 1333 | 24 | 2518 |
| Actinosynnema | 21322 | 8173 | 16337 | 633 | 18351 |
| Ewingella | 0 | 0 | 0 | 0 | 0 |
| Brevundimonas | 22714 | 29662 | 27342 | 0 | 35806 |
| Lawsonia | 2 | 9 | 4 | 0 | 23 |
| Chromohalobacter | 575 | 783 | 853 | 0 | 1063 |
| Rubrobacter | 9487 | 14130 | 18499 | 291 | 23391 |
| Halobacteroides | 54 | 23 | 55 | 0 | 60 |
| Ammonifex | 186 | 373 | 452 | 0 | 705 |
| Kutzneria | 56688 | 15719 | 50938 | 1811 | 53023 |
| Brachybacterium | 13699 | 14856 | 21521 | 787 | 27331 |
| Jonesia | 0 | 0 | 0 | 0 | 0 |
| Syntrophus | 192 | 430 | 425 | 6 | 569 |
| Tolumonas | 71 | 158 | 241 | 5 | 163 |
| Caldicellulosiruptor | 0 | 0 | 0 | 0 | 0 |
| Ferrimonas | 604 | 989 | 1028 | 43 | 1299 |
| Polynucleobacter | 1158 | 2864 | 1711 | 66 | 2620 |
| Paenibacillus | 77873 | 101407 | 188585 | 637 | 137298 |
| Moorella | 598 | 1284 | 1322 | 38 | 1912 |
| Microcoleus | 0 | 0 | 0 | 0 | 0 |
| Pleurocapsa | 78 | 375 | 169 | 0 | 223 |
| Aquabacter | 3067 | 2001 | 2359 | 27 | 3212 |
| Desulfonema | 205 | 552 | 409 | 8 | 890 |
| Desulfohalobium | 149 | 389 | 333 | 0 | 412 |
| Geovibrio | 98 | 264 | 205 | 2 | 222 |
| Pseudobutyrivibrio | 19 | 19 | 6 | 0 | 15 |
| Entomoplasma | 0 | 1 | 6 | 0 | 10 |
| Mesoplasma | 0 | 0 | 0 | 0 | 0 |
| Oenococcus | 25 | 86 | 81 | 0 | 136 |
| Agrococcus | 8322 | 11342 | 14496 | 253 | 16792 |
| Devosia | 20846 | 18883 | 15507 | 222 | 22398 |
| Leptolyngbya | 1766 | 7953 | 6214 | 85 | 6767 |
| Hydrogenophaga | 18714 | 26199 | 28373 | 330 | 40577 |
| Lautropia | 746 | 1076 | 1357 | 17 | 1731 |
| Microbulbifer | 3184 | 4691 | 4603 | 84 | 5635 |
| Marinobacterium | 1444 | 2121 | 1789 | 65 | 2548 |
| Ralstonia | 16588 | 28286 | 19908 | 0 | 27159 |
| Candidatus Arthromitus | 0 | 0 | 0 | 0 | 0 |
| Psychroserpens | 44 | 52 | 32 | 1 | 31 |
| Acetomicrobium | 73 | 86 | 85 | 0 | 93 |
| Acidocella | 1031 | 1120 | 1032 | 28 | 1597 |
| Syntrophobotulus | 68 | 95 | 105 | 0 | 116 |
| Wigglesworthia | 18 | 11 | 21 | 0 | 28 |
| Salinivibrio | 214 | 387 | 264 | 16 | 283 |
| Dehalobacterium | 0 | 0 | 0 | 0 | 0 |
| Tetragenococcus | 27 | 66 | 80 | 0 | 192 |
| Polaromonas | 3649 | 7283 | 6148 | 228 | 17648 |
| Pseudoalteromonas | 950 | 2638 | 1813 | 202 | 2372 |
| Desulfocapsa | 0 | 0 | 0 | 0 | 0 |
| Pantoea | 4089 | 13654 | 6747 | 197 | 9046 |
| Intrasporangium | 2571 | 2716 | 4438 | 92 | 4645 |
| Catellatospora | 8235 | 6461 | 43513 | 421 | 23666 |
| Friedmanniella | 2671 | 3642 | 6271 | 206 | 9497 |
| Thiodictyon | 1676 | 3910 | 3302 | 74 | 4795 |
| Janibacter | 7129 | 8369 | 12278 | 28 | 15072 |
| Nakamurella | 7185 | 7286 | 13093 | 557 | 16204 |
| Deferribacter | 33 | 31 | 46 | 1 | 60 |
| Acidimicrobium | 885 | 1309 | 2219 | 28 | 2196 |
| Octadecabacter | 931 | 1075 | 911 | 29 | 1411 |
| Xylophilus | 2010 | 2354 | 2470 | 72 | 4544 |
| Chroococcidiopsis | 394 | 1625 | 1435 | 1 | 1630 |
| Planktothrix | 0 | 0 | 0 | 0 | 0 |
| Aneurinibacillus | 0 | 0 | 0 | 0 | 0 |
| Brevibacillus | 2887 | 6925 | 5735 | 105 | 7248 |
| Leucobacter | 9916 | 11889 | 16754 | 734 | 22798 |
| Cylindrospermum | 47 | 833 | 248 | 0 | 203 |
| Dehalobacter | 44 | 347 | 193 | 0 | 201 |
| Couchioplanes | 5335 | 3906 | 11235 | 329 | 10040 |
| Microvirgula | 750 | 1302 | 1287 | 44 | 1716 |
| Kocuria | 10308 | 10546 | 15122 | 138 | 21195 |
| Nesterenkonia | 736 | 739 | 1133 | 68 | 2054 |
| Dermacoccus | 3088 | 3667 | 5175 | 73 | 6057 |
| Kytococcus | 1417 | 2041 | 3221 | 63 | 3936 |
| Sulfurospirillum | 0 | 0 | 0 | 0 | 0 |
| Vogesella | 1009 | 1583 | 1526 | 19 | 2531 |
| Moritella | 63 | 185 | 112 | 15 | 166 |
| Sagittula | 2453 | 2414 | 2475 | 49 | 4055 |
| Rickettsiella | 0 | 0 | 0 | 0 | 0 |
| Blastochloris | 9713 | 6264 | 6870 | 204 | 9559 |
| Skermania | 1395 | 1673 | 2577 | 95 | 3053 |
| Chryseobacterium | 1689 | 4605 | 4075 | 13 | 4945 |
| Empedobacter | 0 | 0 | 0 | 0 | 0 |
| Bergeyella | 12 | 207 | 16 | 0 | 37 |
| Persicobacter | 0 | 0 | 0 | 0 | 0 |
| Alcanivorax | 2846 | 3519 | 3589 | 90 | 4405 |
| Sulfitobacter | 7962 | 9758 | 8801 | 151 | 13245 |
| Desulfobacca | 267 | 456 | 501 | 9 | 662 |
| Sanguibacter | 3025 | 3767 | 5764 | 152 | 6237 |
| Dehalococcoides | 140 | 321 | 348 | 0 | 570 |
| Geitlerinema | 459 | 832 | 827 | 5 | 955 |
| Desulfurobacterium | 2 | 44 | 35 | 1 | 45 |
| Mitsuaria | 2072 | 3196 | 2852 | 39 | 4533 |
| Euhalothece | 16 | 22 | 47 | 0 | 82 |
| Actinoalloteichus | 13556 | 5645 | 15558 | 713 | 20413 |
| Meiothermus | 794 | 1385 | 1753 | 17 | 2563 |
| Psychromonas | 99 | 113 | 47 | 5 | 69 |
| Mesorhizobium | 125389 | 127413 | 116279 | 1419 | 166794 |
| Cyclobacterium | 0 | 0 | 0 | 0 | 0 |
| Coprothermobacter | 22 | 132 | 130 | 11 | 99 |
| Orientia | 21 | 21 | 20 | 0 | 23 |
| Cryobacterium | 3830 | 5004 | 6989 | 253 | 9575 |
| Macrococcus | 78 | 387 | 168 | 1 | 247 |
| Frateuria | 4010 | 4527 | 4762 | 97 | 5279 |
| Hydrogenophilus | 1128 | 880 | 978 | 0 | 1226 |
| Brenneria | 653 | 1185 | 1163 | 31 | 1634 |
| Waddlia | 80 | 99 | 72 | 1 | 106 |
| Citromicrobium | 705 | 1098 | 1053 | 17 | 1592 |
| Tessaracoccus | 9269 | 16235 | 15597 | 518 | 21535 |
| Dechloromonas | 3523 | 5697 | 5410 | 56 | 7500 |
| Methylocaldum | 908 | 1464 | 1283 | 16 | 1808 |
| Thermaerobacter | 3589 | 5822 | 7053 | 119 | 8807 |
| Roseovarius | 7258 | 7575 | 7845 | 114 | 10503 |
| Antarctobacter | 0 | 0 | 0 | 0 | 0 |
| Maricaulis | 667 | 649 | 628 | 12 | 980 |
| Gracilibacillus | 61 | 95 | 50 | 0 | 92 |
| Rhodanobacter | 6390 | 7671 | 8266 | 400 | 9573 |
| Duganella | 1387 | 2369 | 2688 | 27 | 10278 |
| Neptunomonas | 0 | 0 | 0 | 0 | 0 |
| Thermocrinis | 100 | 232 | 119 | 0 | 158 |
| Halothece | 66 | 164 | 208 | 0 | 213 |
| Thermobacillus | 770 | 1796 | 1361 | 9 | 1828 |
| Mycetocola | 1369 | 2120 | 2663 | 148 | 3691 |
| Myroides | 0 | 0 | 0 | 0 | 0 |
| Actinobaculum | 189 | 295 | 367 | 46 | 812 |
| Asticcacaulis | 1810 | 2336 | 2042 | 72 | 2940 |
| Cylindrospermopsis | 1 | 48 | 59 | 0 | 66 |
| Allorhizobium | 104 | 316 | 578 | 10 | 496 |
| Desulfosporosinus | 135 | 411 | 303 | 5 | 2907 |
| Chitinophaga | 2188 | 3586 | 2638 | 21 | 3746 |
| Heliorestis | 91 | 170 | 64 | 3 | 94 |
| Denitrobacterium | 287 | 663 | 520 | 0 | 542 |
| Aminomonas | 0 | 0 | 0 | 0 | 0 |
| Thermanaerovibrio | 325 | 626 | 694 | 6 | 815 |
| Aminobacterium | 42 | 82 | 127 | 1 | 231 |
| Methylovorus | 331 | 602 | 450 | 0 | 655 |
| Buttiauxella | 233 | 1055 | 465 | 16 | 13560 |
| Leminorella | 93 | 131 | 162 | 0 | 201 |
| Obesumbacterium | 24 | 61 | 55 | 5 | 72 |
| Pragia | 41 | 110 | 91 | 9 | 101 |
| Tatumella | 0 | 0 | 0 | 0 | 0 |
| Corallococcus | 8606 | 16427 | 14944 | 211 | 23133 |
| Psychroflexus | 7 | 39 | 46 | 3 | 48 |
| Luteimonas | 17763 | 15811 | 16559 | 180 | 20294 |
| Pseudoxanthomonas | 17800 | 18055 | 17662 | 54 | 19764 |
| Leclercia | 2095 | 3301 | 3396 | 8 | 3776 |
| Thermobifida | 4850 | 4450 | 9131 | 1587 | 26679 |
| Nonomuraea | 34815 | 21512 | 167463 | 33906 | 547236 |
| Succinivibrio | 35 | 74 | 31 | 0 | 61 |
| Slackia | 0 | 0 | 0 | 0 | 0 |
| Eggerthella | 1639 | 2786 | 3217 | 85 | 3686 |
| Cryptobacterium | 13 | 50 | 47 | 0 | 113 |
| Hippea | 32 | 30 | 76 | 0 | 50 |
| Sodalis | 811 | 1222 | 1207 | 71 | 1760 |
| Pedobacter | 1014 | 3180 | 3549 | 38 | 5075 |
| Beutenbergia | 2320 | 2632 | 3945 | 119 | 4239 |
| Allochromatium | 2365 | 3320 | 3038 | 36 | 3552 |
| Thermochromatium | 650 | 1034 | 1036 | 13 | 1257 |
| Marichromatium | 1495 | 1990 | 2133 | 26 | 2366 |
| Halorhodospira | 1121 | 2144 | 1860 | 19 | 1990 |
| Bosea | 37051 | 33725 | 30771 | 570 | 41962 |
| Marmoricola | 3143 | 4325 | 5996 | 163 | 6588 |
| Modestobacter | 5841 | 7290 | 11450 | 432 | 15469 |
| Ferribacterium | 2097 | 2705 | 2567 | 63 | 4804 |
| Ignavigranum | 7 | 107 | 12 | 0 | 13 |
| Glaciecola | 23 | 61 | 86 | 0 | 64 |
| Gluconacetobacter | 938 | 1306 | 1204 | 15 | 1712 |
| Hymenobacter | 11004 | 23207 | 20950 | 341 | 29323 |
| Asaia | 381 | 726 | 743 | 0 | 994 |
| Aquabacterium | 4208 | 5665 | 5655 | 0 | 8495 |
| Ketogulonicigenium | 820 | 1254 | 1047 | 44 | 1600 |
| Pandoraea | 11476 | 16941 | 16924 | 260 | 23725 |
| Roseateles | 3608 | 4763 | 4719 | 76 | 6665 |
| Roseivivax | 3007 | 2972 | 3346 | 167 | 4403 |
| Frigoribacterium | 2352 | 3622 | 4429 | 205 | 5607 |
| Ruegeria | 2644 | 3006 | 2908 | 40 | 3937 |
| Richelia | 20 | 77 | 39 | 0 | 60 |
| Tetrasphaera | 2255 | 2346 | 3569 | 123 | 4625 |
| Chloroherpeton | 0 | 0 | 0 | 0 | 0 |
| Collinsella | 1165 | 1734 | 2097 | 54 | 2276 |
| Stanieria | 47 | 141 | 99 | 0 | 88 |
| Gloeocapsa | 0 | 0 | 0 | 0 | 0 |
| Cyanobacterium | 0 | 0 | 0 | 0 | 0 |
| Streptomonospora | 4945 | 4468 | 8583 | 1302 | 23679 |
| Cellulophaga | 107 | 213 | 135 | 0 | 146 |
| Pelistega | 2 | 24 | 17 | 5 | 14 |
| Cupriavidus | 50734 | 66766 | 76463 | 1120 | 109894 |
| Ensifer | 16220 | 15321 | 13302 | 236 | 18624 |
| Thioalkalivibrio | 6182 | 8899 | 9015 | 136 | 10358 |
| Desulfotalea | 14 | 92 | 82 | 8 | 174 |
| Halothiobacillus | 891 | 1062 | 941 | 44 | 1114 |
| Bisgaardia | 10 | 6 | 6 | 0 | 7 |
| Anabaenopsis | 132 | 1067 | 182 | 1 | 415 |
| Leifsonia | 9863 | 9689 | 13072 | 410 | 18732 |
| Agreia | 891 | 970 | 1254 | 66 | 1656 |
| Muricauda | 417 | 718 | 508 | 1 | 789 |
| Tolypothrix | 0 | 0 | 0 | 0 | 0 |
| Defluviicoccus | 1876 | 2393 | 1919 | 6 | 3013 |
| Ignatzschineria | 34 | 42 | 56 | 0 | 58 |
| Zobellia | 32 | 87 | 52 | 1 | 67 |
| Neochlamydia | 44 | 233 | 86 | 1 | 629 |
| Candidatus Carsonella | 0 | 0 | 0 | 0 | 0 |
| Tepidimonas | 1749 | 2097 | 2168 | 2 | 3509 |
| Alkaliphilus | 204 | 82 | 100 | 2 | 64 |
| Trichlorobacter | 462 | 1543 | 1245 | 3 | 1638 |
| Micropruina | 1558 | 2134 | 2967 | 15 | 3628 |
| Actinopolymorpha | 3567 | 3364 | 6165 | 387 | 9572 |
| Denitrovibrio | 38 | 59 | 97 | 0 | 214 |
| Bulleidia | 22 | 55 | 94 | 0 | 93 |
| Filomicrobium | 1751 | 1028 | 1016 | 31 | 968 |
| Acidithiobacillus | 2017 | 4003 | 3553 | 57 | 4718 |
| Subtercola | 1650 | 1979 | 2535 | 68 | 3218 |
| Methylocella | 3578 | 3427 | 2961 | 78 | 4934 |
| Dyadobacter | 1352 | 3173 | 2585 | 28 | 3036 |
| Roseiflexus | 1659 | 3545 | 2988 | 66 | 3670 |
| Pectobacterium | 1716 | 2536 | 2244 | 48 | 2820 |
| Roseomonas | 21183 | 20657 | 21741 | 521 | 32194 |
| Ornithinimicrobium | 9239 | 11990 | 18175 | 972 | 27069 |
| Syntrophothermus | 95 | 366 | 241 | 0 | 311 |
| Geobacillus | 1421 | 3446 | 2870 | 0 | 3427 |
| Oceanimonas | 376 | 564 | 519 | 2 | 681 |
| Carboxydothermus | 66 | 297 | 199 | 0 | 183 |
| Limnothrix | 0 | 7 | 3 | 0 | 13 |
| Alkalilimnicola | 993 | 1291 | 1620 | 63 | 2034 |
| Olsenella | 1305 | 1821 | 2251 | 23 | 2807 |
| Candidatus Xiphinematobacter | 285 | 794 | 516 | 5 | 658 |
| Idiomarina | 0 | 0 | 0 | 0 | 0 |
| Catenibacterium | 17 | 8 | 11 | 0 | 15 |
| Thalassomonas | 308 | 513 | 435 | 25 | 770 |
| Quatrionicoccus | 703 | 1375 | 1275 | 33 | 1783 |
| Denitromonas | 25 | 14 | 19 | 0 | 7 |
| Thermacetogenium | 279 | 714 | 757 | 2 | 1096 |
| Oleiphilus | 0 | 0 | 0 | 0 | 0 |
| Thermomonas | 8702 | 9570 | 8457 | 112 | 9074 |
| Prauserella | 6133 | 2162 | 7184 | 206 | 6619 |
| Salegentibacter | 141 | 150 | 190 | 22 | 166 |
| Bacteriovorax | 35 | 54 | 70 | 2 | 106 |
| Thermosynechococcus | 0 | 0 | 0 | 0 | 0 |
| Salinibacter | 1125 | 2530 | 2358 | 21 | 3026 |
| Azospira | 5027 | 7294 | 8404 | 0 | 14742 |
| Thermobispora | 3284 | 2832 | 9026 | 3912 | 68237 |
| Massilia | 25420 | 47482 | 50585 | 495 | 94293 |
| Blastomonas | 1099 | 1520 | 1364 | 0 | 2146 |
| Anoxybacillus | 538 | 1470 | 1164 | 3 | 1627 |
| Roseibium | 6585 | 5940 | 5076 | 32 | 6828 |
| Starkeya | 10574 | 7918 | 7624 | 190 | 11627 |
| Stappia | 5629 | 5042 | 4479 | 165 | 6404 |
| Pigmentiphaga | 2850 | 3716 | 4087 | 101 | 6301 |
| Aequorivita | 198 | 285 | 216 | 51 | 279 |
| Kozakia | 394 | 713 | 513 | 4 | 854 |
| Georgenia | 7371 | 9225 | 13719 | 478 | 17927 |
| Gallibacterium | 0 | 0 | 0 | 0 | 0 |
| Thioflavicoccus | 1590 | 2113 | 2267 | 66 | 2598 |
| Dysgonomonas | 4 | 28 | 17 | 0 | 43 |
| Reichenbachiella | 51 | 138 | 162 | 1 | 122 |
| Jeotgalibacillus | 8 | 45 | 65 | 0 | 50 |
| Sterolibacterium | 1303 | 1656 | 2027 | 51 | 3186 |
| Cellulosimicrobium | 9955 | 11151 | 15798 | 529 | 19570 |
| Hahella | 604 | 828 | 884 | 84 | 1024 |
| Cedecea | 667 | 1312 | 1362 | 32 | 1728 |
| Megamonas | 22 | 49 | 54 | 3 | 49 |
| Moellerella | 1 | 59 | 46 | 3 | 41 |
| Nodularia | 128 | 398 | 255 | 0 | 424 |
| Roseibacterium | 721 | 697 | 776 | 7 | 1037 |
| Raoultella | 1754 | 2974 | 2957 | 81 | 3028 |
| Ureibacillus | 0 | 0 | 0 | 0 | 0 |
| Marinitoga | 0 | 17 | 17 | 0 | 22 |
| Anaeromyxobacter | 20665 | 38260 | 42130 | 580 | 48857 |
| Haliangium | 5193 | 8058 | 8636 | 107 | 9279 |
| Magnetococcus | 161 | 365 | 262 | 9 | 372 |
| Oerskovia | 1656 | 2157 | 3472 | 120 | 4277 |
| Lentzea | 66670 | 20722 | 48542 | 658 | 29518 |
| Sphingobium | 35751 | 42772 | 36304 | 483 | 58220 |
| Novosphingobium | 18082 | 23281 | 20859 | 0 | 32379 |
| Sphingopyxis | 22387 | 31364 | 24064 | 479 | 37300 |
| Kaistia | 5289 | 4851 | 4361 | 122 | 6092 |
| Cyanobium | 1915 | 2737 | 2816 | 63 | 3879 |
| Ilyobacter | 15 | 16 | 5 | 0 | 17 |
| Laribacter | 1038 | 1361 | 1982 | 14 | 2853 |
| Hydrogenobaculum | 25 | 0 | 65 | 10 | 78 |
| Salinispora | 1361 | 1374 | 4068 | 216 | 6055 |
| Sneathia | 1 | 3 | 1 | 0 | 13 |
| Thalassospira | 1638 | 1842 | 1593 | 39 | 2579 |
| Nordella | 9581 | 5949 | 7940 | 235 | 8094 |
| Citricoccus | 2142 | 3063 | 4715 | 63 | 4845 |
| Halomicronema | 249 | 556 | 464 | 7 | 677 |
| Caloranaerobacter | 75 | 31 | 68 | 2 | 67 |
| Tistrella | 4635 | 4676 | 5538 | 77 | 8659 |
| Thermovibrio | 56 | 146 | 164 | 0 | 236 |
| Gemmatimonas | 6953 | 24103 | 17906 | 239 | 70516 |
| Allobaculum | 106 | 184 | 181 | 10 | 191 |
| Ramlibacter | 7830 | 10850 | 10662 | 338 | 16916 |
| Lentibacillus | 51 | 123 | 94 | 1 | 172 |
| Opitutus | 5317 | 11798 | 11674 | 147 | 12271 |
| Carboxydocella | 80 | 135 | 155 | 0 | 153 |
| Cetobacterium | 0 | 8 | 7 | 0 | 9 |
| Salinisphaera | 1299 | 1586 | 1579 | 30 | 1760 |
| Aurantimonas | 3318 | 3343 | 3020 | 55 | 4403 |
| Kribbella | 23245 | 17876 | 89940 | 855 | 62672 |
| Oceanobacillus | 0 | 0 | 0 | 0 | 0 |
| Persephonella | 74 | 77 | 75 | 0 | 152 |
| Xylanimonas | 5493 | 6098 | 8823 | 232 | 11063 |
| Marinithermus | 993 | 1549 | 1625 | 7 | 2132 |
| Microvirga | 17660 | 16254 | 15192 | 367 | 21762 |
| Acetoanaerobium | 13 | 18 | 24 | 0 | 12 |
| Caldithrix | 127 | 546 | 378 | 18 | 636 |
| Thalassolituus | 0 | 0 | 0 | 0 | 0 |
| Enterovibrio | 9 | 17 | 13 | 0 | 20 |
| Jannaschia | 2737 | 2887 | 3218 | 82 | 4026 |
| Gelria | 563 | 821 | 1069 | 5 | 1170 |
| Dorea | 82 | 171 | 100 | 0 | 115 |
| Plantibacter | 2196 | 5054 | 6796 | 108 | 6282 |
| Sedimentibacter | 74 | 36 | 41 | 0 | 45 |
| Leisingera | 4114 | 5120 | 4883 | 171 | 6632 |
| Caminibacter | 28 | 57 | 51 | 0 | 81 |
| Turicibacter | 25 | 46 | 69 | 0 | 49 |
| Conexibacter | 32931 | 27184 | 66226 | 492 | 74518 |
| Marinilactibacillus | 10 | 12 | 22 | 0 | 12 |
| Thermoleophilum | 5969 | 4313 | 11111 | 75 | 12851 |
| Alysiella | 38 | 109 | 100 | 0 | 132 |
| Tannerella | 362 | 686 | 656 | 8 | 1073 |
| Caldimonas | 4737 | 7455 | 7335 | 105 | 11206 |
| Terasakiella | 0 | 0 | 0 | 0 | 0 |
| Parascardovia | 101 | 283 | 240 | 24 | 584 |
| Candidatus Pelagibacter | 0 | 0 | 0 | 0 | 0 |
| Candidatus Baumannia | 12 | 16 | 29 | 0 | 18 |
| Paraliobacillus | 11 | 20 | 24 | 0 | 14 |
| Alicycliphilus | 7932 | 8052 | 9343 | 119 | 13134 |
| Sulfurimonas | 0 | 0 | 0 | 0 | 0 |
| Collimonas | 3110 | 4856 | 4339 | 41 | 7524 |
| Thermanaeromonas | 84 | 140 | 174 | 0 | 183 |
| Candidatus Blochmannia | 87 | 96 | 70 | 0 | 94 |
| Dickeya | 2268 | 3371 | 2986 | 145 | 5571 |
| Cobetia | 1695 | 2332 | 2454 | 0 | 3202 |
| Skermanella | 11510 | 13731 | 13292 | 288 | 21292 |
| Gemmobacter | 1558 | 1541 | 1823 | 27 | 2615 |
| Labrys | 3133 | 2823 | 2690 | 45 | 3912 |
| Candidatus Nardonella | 18 | 9 | 21 | 1 | 10 |
| Parvularcula | 494 | 852 | 687 | 7 | 1088 |
| Oceanithermus | 1131 | 2148 | 2365 | 25 | 2986 |
| Morococcus | 56 | 172 | 152 | 0 | 154 |
| Rhizobacter | 5748 | 9459 | 8104 | 62 | 13455 |
| Sulfurihydrogenibium | 0 | 0 | 0 | 0 | 0 |
| Tepidibacter | 0 | 0 | 0 | 0 | 0 |
| Histophilus | 18 | 24 | 30 | 0 | 9 |
| Schlegelella | 1988 | 2674 | 2610 | 0 | 4374 |
| Croceibacter | 19 | 54 | 34 | 0 | 40 |
| Faecalibacterium | 1510 | 3218 | 3468 | 102 | 4639 |
| Chamaesiphon | 131 | 435 | 320 | 3 | 1074 |
| Desulfatibacillum | 307 | 644 | 633 | 13 | 853 |
| Ottowia | 2262 | 3033 | 3092 | 13 | 4657 |
| Kordia | 0 | 0 | 0 | 0 | 0 |
| Candidatus Competibacter | 4 | 14 | 22 | 0 | 41 |
| Hydrogenimonas | 162 | 319 | 298 | 1 | 297 |
| Pyxidicoccus | 3509 | 7900 | 6335 | 73 | 7482 |
| Oceanisphaera | 119 | 146 | 145 | 100 | 196 |
| Formosa | 89 | 171 | 130 | 1 | 134 |
| Thermodesulfobium | 18 | 96 | 79 | 3 | 79 |
| Pannonibacter | 6292 | 5285 | 5050 | 58 | 7844 |
| Streptacidiphilus | 6399 | 5720 | 10393 | 1073 | 20898 |
| Reinekea | 87 | 229 | 182 | 14 | 262 |
| Chitinibacter | 227 | 647 | 595 | 6 | 668 |
| Dyella | 6774 | 7332 | 7381 | 272 | 9056 |
| Saccharospirillum | 268 | 688 | 410 | 13 | 480 |
| Belliella | 0 | 0 | 0 | 0 | 0 |
| Hylemonella | 841 | 1103 | 1061 | 27 | 1728 |
| Ectothiorhodosinus | 100 | 257 | 169 | 2 | 259 |
| Anaerolinea | 210 | 462 | 542 | 5 | 759 |
| Caldilinea | 756 | 2049 | 1620 | 22 | 2043 |
| Candidatus Portiera | 0 | 0 | 0 | 0 | 0 |
| Salinibacterium | 3341 | 5938 | 6371 | 260 | 8919 |
| Diaphorobacter | 34842 | 16991 | 17434 | 0 | 24822 |
| Pseudorhodobacter | 231 | 253 | 218 | 14 | 378 |
| Alistipes | 3513 | 7395 | 6523 | 107 | 9018 |
| Akkermansia | 973 | 2433 | 1802 | 35 | 2531 |
| Chitinimonas | 3759 | 4765 | 5333 | 78 | 7304 |
| Thermodesulfatator | 51 | 160 | 72 | 2 | 148 |
| Crinalium | 69 | 144 | 103 | 0 | 338 |
| Luteibacter | 7790 | 10116 | 9987 | 159 | 11240 |
| Anaerotruncus | 323 | 642 | 592 | 18 | 693 |
| Gillisia | 5 | 33 | 28 | 15 | 27 |
| Nitratireductor | 5458 | 5316 | 5034 | 104 | 6542 |
| Grimontia | 181 | 238 | 242 | 0 | 210 |
| Algoriphagus | 179 | 378 | 331 | 10 | 350 |
| Mucispirillum | 85 | 112 | 71 | 0 | 82 |
| Marvinbryantia | 76 | 247 | 219 | 27 | 481 |
| Caldanaerobacter | 39 | 164 | 140 | 7 | 177 |
| Oceanicola | 576 | 610 | 801 | 9 | 1095 |
| Robiginitalea | 177 | 396 | 385 | 3 | 368 |
| Maribacter | 0 | 0 | 0 | 0 | 0 |
| Mahella | 35 | 161 | 111 | 3 | 161 |
| Ethanoligenens | 274 | 649 | 491 | 3 | 665 |
| Aquicella | 222 | 359 | 500 | 1 | 611 |
| Isoptericola | 4240 | 4632 | 7412 | 213 | 7733 |
| Chlorobaculum | 1177 | 3112 | 2752 | 20 | 3973 |
| Parvibaculum | 3948 | 3978 | 3757 | 39 | 5752 |
| Gulosibacter | 1763 | 2166 | 2922 | 143 | 3921 |
| Lentisphaera | 28 | 100 | 86 | 2 | 102 |
| Kerstersia | 559 | 586 | 666 | 0 | 910 |
| Pseudovibrio | 0 | 0 | 0 | 0 | 0 |
| Agarivorans | 76 | 162 | 139 | 16 | 194 |
| Algibacter | 0 | 0 | 0 | 0 | 0 |
| Thermobaculum | 370 | 666 | 717 | 36 | 1149 |
| Salipiger | 4933 | 5149 | 5721 | 59 | 7617 |
| Crocosphaera | 38 | 164 | 101 | 1 | 89 |
| Trichormus | 30 | 326 | 125 | 0 | 126 |
| Blastopirellula | 1393 | 4227 | 3030 | 93 | 5270 |
| Rhodopirellula | 1266 | 4030 | 2688 | 22 | 4769 |
| Sulfurovum | 43 | 322 | 268 | 1 | 292 |
| Serinicoccus | 5544 | 6531 | 9512 | 260 | 11694 |
| Thalassobacter | 0 | 0 | 0 | 0 | 0 |
| Arsenicicoccus | 2054 | 2339 | 3794 | 153 | 4926 |
| Nitrincola | 0 | 0 | 0 | 0 | 0 |
| Owenweeksia | 50 | 56 | 57 | 0 | 39 |
| Chondrocystis | 34 | 194 | 157 | 1 | 177 |
| Nitratiruptor | 19 | 123 | 88 | 0 | 200 |
| Nitratifractor | 137 | 321 | 239 | 2 | 343 |
| Candidatus Cardinium | 11 | 74 | 45 | 0 | 46 |
| Alkalibacter | 25 | 91 | 101 | 4 | 104 |
| Hoeflea | 1967 | 1938 | 1800 | 17 | 2504 |
| Thermincola | 47 | 174 | 114 | 3 | 153 |
| Aquiflexum | 109 | 87 | 126 | 2 | 95 |
| Candidatus Amoebophilus | 11 | 35 | 26 | 0 | 50 |
| Jiangella | 12784 | 11058 | 22020 | 785 | 23988 |
| Candidatus Protochlamydia | 0 | 0 | 0 | 0 | 0 |
| Odoribacter | 61 | 68 | 71 | 0 | 51 |
| Leeuwenhoekiella | 32 | 69 | 78 | 0 | 118 |
| Bizionia | 6 | 24 | 18 | 0 | 36 |
| Stackebrandtia | 2834 | 2093 | 6292 | 197 | 7340 |
| Thioclava | 1375 | 1694 | 1651 | 12 | 2281 |
| Sulfuricurvum | 1 | 27 | 53 | 0 | 66 |
| Alkaliflexus | 51 | 84 | 56 | 0 | 105 |
| Segniliparus | 618 | 735 | 1071 | 85 | 1973 |
| Kordiimonas | 120 | 342 | 170 | 3 | 373 |
| Salinimonas | 207 | 615 | 352 | 7 | 404 |
| Pontibacillus | 23 | 58 | 64 | 0 | 89 |
| Advenella | 390 | 629 | 543 | 26 | 764 |
| Thermosediminibacter | 88 | 181 | 163 | 0 | 187 |
| Subdoligranulum | 0 | 0 | 0 | 0 | 0 |
| Laceyella | 129 | 342 | 359 | 0 | 484 |
| Gramella | 293 | 617 | 360 | 4 | 511 |
| Candidatus Rhabdochlamydia | 11 | 151 | 68 | 0 | 86 |
| Martelella | 8372 | 7231 | 7312 | 227 | 10555 |
| Proteiniphilum | 84 | 161 | 117 | 13 | 179 |
| Candidatus Hepatoplasma | 5 | 9 | 8 | 0 | 16 |
| Tateyamaria | 0 | 0 | 0 | 0 | 0 |
| Uruburuella | 456 | 538 | 420 | 7 | 624 |
| Phaeobacter | 2889 | 3148 | 2912 | 51 | 4305 |
| Herminiimonas | 256 | 405 | 327 | 0 | 455 |
| Endozoicomonas | 0 | 0 | 0 | 0 | 0 |
| Pusillimonas | 1600 | 2338 | 2206 | 103 | 3489 |
| Petrimonas | 50 | 146 | 187 | 5 | 161 |
| Dinoroseobacter | 1095 | 1228 | 1485 | 36 | 1859 |
| Denitratisoma | 2474 | 3978 | 4571 | 44 | 6850 |
| Emticicia | 0 | 0 | 0 | 0 | 0 |
| Sideroxydans | 565 | 956 | 836 | 31 | 1437 |
| Methylibium | 3175 | 4150 | 4147 | 73 | 7502 |
| Saccharophagus | 39 | 72 | 45 | 3 | 77 |
| Paucibacter | 750 | 1031 | 1015 | 7 | 1738 |
| Leadbetterella | 95 | 88 | 70 | 1 | 116 |
| Neoasaia | 505 | 682 | 575 | 53 | 1014 |
| Dokdonella | 3013 | 3666 | 4106 | 75 | 4161 |
| Pontibacter | 1433 | 3155 | 2642 | 40 | 3848 |
| Shinella | 16248 | 17869 | 18059 | 416 | 25550 |
| Dokdonia | 56 | 83 | 54 | 1 | 106 |
| Cohnella | 1213 | 2766 | 2616 | 13 | 3461 |
| Fluviicola | 18 | 56 | 44 | 0 | 47 |
| Larkinella | 369 | 1246 | 925 | 1 | 1269 |
| Truepera | 861 | 1545 | 1748 | 33 | 2585 |
| Conchiformibius | 63 | 59 | 100 | 1 | 108 |
| Sphingosinicella | 8989 | 11241 | 10752 | 191 | 23670 |
| Thalassobius | 0 | 0 | 0 | 0 | 0 |
| Thermovirga | 18 | 117 | 109 | 11 | 205 |
| Olleya | 29 | 65 | 41 | 0 | 53 |
| Candidatus Karelsulcia | 28 | 48 | 23 | 1 | 63 |
| Microcella | 4267 | 6389 | 7259 | 182 | 8340 |
| Turneriella | 266 | 512 | 398 | 9 | 512 |
| Polymorphospora | 4713 | 3480 | 11281 | 628 | 15121 |
| Candidatus Contubernalis | 14 | 79 | 68 | 2 | 92 |
| Criblamydia | 54 | 340 | 21 | 0 | 18 |
| Costertonia | 23 | 66 | 27 | 0 | 31 |
| Paludibacter | 49 | 76 | 59 | 1 | 69 |
| Zobellella | 890 | 1041 | 1232 | 27 | 1643 |
| Sedimenticola | 325 | 504 | 502 | 15 | 612 |
| Salimicrobium | 0 | 0 | 0 | 0 | 0 |
| Simplicispira | 889 | 1479 | 1515 | 5 | 2193 |
| Pseudochrobactrum | 0 | 0 | 0 | 0 | 0 |
| Niastella | 1250 | 1188 | 838 | 2 | 1276 |
| Lutibacter | 65 | 38 | 47 | 4 | 49 |
| Castellaniella | 3020 | 2518 | 3660 | 32 | 4667 |
| Marinimicrobium | 436 | 605 | 563 | 1 | 561 |
| Methylotenera | 0 | 0 | 0 | 0 | 0 |
| Desulfoglaeba | 512 | 1434 | 1013 | 5 | 1344 |
| Altererythrobacter | 2589 | 3502 | 3103 | 30 | 4971 |
| Nonlabens | 0 | 0 | 0 | 0 | 0 |
| Verminephrobacter | 1223 | 1349 | 1486 | 9 | 2195 |
| Granulibacter | 588 | 985 | 848 | 26 | 1191 |
| Aureispira | 0 | 0 | 0 | 0 | 0 |
| Pelosinus | 92 | 172 | 153 | 17 | 243 |
| Haematobacter | 1623 | 2062 | 1587 | 16 | 2366 |
| Phycicoccus | 4472 | 5489 | 8023 | 264 | 10226 |
| Rivularia | 10 | 155 | 104 | 0 | 155 |
| Parabacteroides | 467 | 1095 | 771 | 21 | 1283 |
| Natranaerobius | 37 | 73 | 39 | 0 | 53 |
| Olivibacter | 147 | 243 | 217 | 0 | 350 |
| Halotalea | 774 | 1280 | 1490 | 15 | 1847 |
| Mariprofundus | 183 | 420 | 537 | 2 | 539 |
| Methyloversatilis | 1225 | 1683 | 1531 | 20 | 3046 |
| Caldalkalibacillus | 71 | 145 | 121 | 2 | 186 |
| Galbibacter | 8 | 28 | 33 | 0 | 35 |
| Gilvibacter | 0 | 0 | 0 | 0 | 0 |
| Niabella | 599 | 849 | 585 | 2 | 716 |
| Candidatus Kuenenia | 155 | 376 | 408 | 12 | 607 |
| Brasilonema | 116 | 250 | 164 | 0 | 208 |
| Maribius | 0 | 0 | 0 | 0 | 0 |
| Marixanthomonas | 19 | 27 | 22 | 1 | 25 |
| Edaphobacter | 3867 | 9495 | 8075 | 79 | 8856 |
| Sediminicola | 21 | 36 | 16 | 3 | 21 |
| Geosporobacter | 44 | 62 | 43 | 2 | 73 |
| Nisaea | 2149 | 1925 | 2165 | 19 | 3389 |
| Actinocatenispora | 7779 | 5655 | 13765 | 570 | 15201 |
| Aquincola | 3025 | 3874 | 4036 | 40 | 6263 |
| Geoalkalibacter | 1048 | 2740 | 2645 | 19 | 3400 |
| Terriglobus | 3375 | 9737 | 7943 | 85 | 9748 |
| Tamlana | 55 | 45 | 40 | 0 | 53 |
| Desulfurispirillum | 0 | 0 | 0 | 0 | 0 |
| Congregibacter | 466 | 671 | 691 | 12 | 887 |
| Candidatus Endoriftia | 462 | 647 | 607 | 10 | 1007 |
| Fulvivirga | 163 | 338 | 227 | 7 | 314 |
| Barnesiella | 71 | 453 | 419 | 0 | 557 |
| Flavisolibacter | 654 | 1084 | 950 | 4 | 1171 |
| Sphaerochaeta | 232 | 423 | 400 | 7 | 522 |
| Paludibacterium | 1663 | 2551 | 2484 | 14 | 3598 |
| Lysinibacillus | 0 | 0 | 0 | 0 | 0 |
| Deefgea | 130 | 791 | 539 | 22 | 546 |
| Undibacterium | 510 | 1030 | 790 | 0 | 1107 |
| Candidatus Riesia | 12 | 7 | 5 | 1 | 8 |
| Salinicola | 937 | 996 | 1123 | 16 | 1445 |
| Aquisalimonas | 1091 | 1370 | 1431 | 15 | 2010 |
| Aquitalea | 877 | 1419 | 1468 | 13 | 2056 |
| Candidatus Ishikawaella | 2 | 12 | 10 | 0 | 5 |
| Candidatus Midichloria | 16 | 26 | 65 | 0 | 50 |
| Celerinatantimonas | 29 | 135 | 33 | 19 | 72 |
| Thermosulfidibacter | 51 | 145 | 118 | 7 | 240 |
| Solimonas | 2676 | 3577 | 3710 | 43 | 3894 |
| Cronobacter | 1245 | 1914 | 1975 | 7 | 2583 |
| Aureimonas | 14092 | 14183 | 13807 | 387 | 18834 |
| Catenulispora | 4677 | 3876 | 7481 | 461 | 10697 |
| Ferrovum | 277 | 610 | 384 | 9 | 656 |
| Zunongwangia | 113 | 166 | 186 | 8 | 143 |
| Litorivicinus | 191 | 222 | 273 | 11 | 347 |
| Mucilaginibacter | 1813 | 3662 | 3248 | 19 | 4377 |
| Elusimicrobium | 45 | 124 | 43 | 3 | 65 |
| Humibacter | 962 | 1485 | 2219 | 53 | 2458 |
| Desulfurivibrio | 296 | 715 | 708 | 3 | 766 |
| Tumebacillus | 875 | 3172 | 2408 | 78 | 2428 |
| Granulosicoccus | 379 | 415 | 452 | 5 | 762 |
| Coraliomargarita | 117 | 541 | 314 | 0 | 386 |
| Mesoflavibacter | 17 | 39 | 18 | 1 | 33 |
| Caldimicrobium | 16 | 31 | 48 | 0 | 83 |
| Flagellimonas | 37 | 76 | 66 | 7 | 138 |
| Adlercreutzia | 779 | 1159 | 1505 | 31 | 1852 |
| Frondihabitans | 2409 | 3449 | 3850 | 97 | 5269 |
| Simiduia | 0 | 0 | 0 | 0 | 0 |
| Phytobacter | 0 | 0 | 0 | 0 | 0 |
| Candidatus Nitrotoga | 545 | 1191 | 952 | 10 | 1853 |
| Desulfarculus | 865 | 1964 | 2335 | 43 | 2712 |
| Rhodocytophaga | 0 | 0 | 0 | 0 | 0 |
| Saccharibacillus | 495 | 859 | 959 | 7 | 1082 |
| Candidatus Cloacimonas | 23 | 99 | 62 | 1 | 162 |
| Elioraea | 2258 | 2472 | 2404 | 112 | 4122 |
| Chloracidobacterium | 2325 | 8553 | 6447 | 75 | 7923 |
| Actinotalea | 2113 | 2487 | 3473 | 117 | 4960 |
| Terribacillus | 32 | 46 | 43 | 0 | 77 |
| Oscillibacter | 204 | 466 | 385 | 16 | 677 |
| Singulisphaera | 19665 | 107917 | 42027 | 271 | 38033 |
| Candidatus Neoehrlichia | 1 | 13 | 5 | 0 | 19 |
| Iamia | 16079 | 12483 | 29851 | 295 | 24211 |
| Steroidobacter | 15305 | 12595 | 6347 | 24 | 3982 |
| Candidatus Desulforudis | 408 | 666 | 808 | 8 | 1144 |
| Devriesea | 100 | 177 | 296 | 5 | 362 |
| Candidatus Purcelliella | 10 | 5 | 14 | 0 | 8 |
| Bibersteinia | 119 | 2592 | 983 | 0 | 1746 |
| Labrenzia | 2343 | 2323 | 2031 | 47 | 2856 |
| Sediminibacillus | 39 | 56 | 65 | 0 | 57 |
| Nosocomiicoccus | 2 | 15 | 7 | 0 | 12 |
| Arenimonas | 1510 | 2364 | 2345 | 20 | 1818 |
| Viridibacillus | 11 | 40 | 25 | 0 | 17 |
| Desulfoluna | 354 | 1000 | 759 | 2 | 906 |
| Tepidanaerobacter | 22 | 57 | 45 | 0 | 41 |
| Kushneria | 677 | 1208 | 1077 | 19 | 1301 |
| Rhodovastum | 3072 | 3680 | 3353 | 55 | 4808 |
| Sediminibacterium | 194 | 215 | 207 | 16 | 158 |
| Cloacibacillus | 0 | 0 | 0 | 0 | 0 |
| Sneathiella | 241 | 405 | 268 | 21 | 341 |
| Candidatus Azobacteroides | 5 | 9 | 5 | 0 | 13 |
| Aliivibrio | 47 | 48 | 94 | 29 | 156 |
| Methylacidiphilum | 160 | 416 | 272 | 0 | 290 |
| Amphritea | 84 | 250 | 204 | 37 | 260 |
| Microterricola | 2621 | 3181 | 4216 | 79 | 4504 |
| Luteolibacter | 2880 | 10063 | 6783 | 69 | 8637 |
| Mariniflexile | 22 | 80 | 86 | 1 | 91 |
| Aquiluna | 217 | 380 | 493 | 33 | 824 |
| Rhodoluna | 0 | 0 | 0 | 0 | 0 |
| Calditerrivibrio | 29 | 57 | 51 | 0 | 102 |
| Aromatoleum | 6508 | 8876 | 8655 | 182 | 13144 |
| Pseudolabrys | 26660 | 11076 | 12052 | 410 | 21979 |
| Fructobacillus | 8 | 12 | 7 | 15 | 11 |
| Salinimicrobium | 44 | 61 | 104 | 0 | 148 |
| Zhouia | 51 | 158 | 37 | 0 | 51 |
| Pseudogulbenkiania | 1168 | 1767 | 1738 | 74 | 2650 |
| Candidatus Hamiltonella | 12 | 58 | 69 | 1 | 81 |
| Candidatus Hodgkinia | 47 | 34 | 22 | 2 | 52 |
| Butyricimonas | 189 | 243 | 227 | 17 | 201 |
| Paraprevotella | 126 | 143 | 248 | 0 | 349 |
| Demequina | 357 | 670 | 954 | 25 | 1159 |
| Butyricicoccus | 0 | 2 | 1 | 0 | 2 |
| Chitiniphilus | 1069 | 1842 | 1651 | 26 | 2638 |
| Sinomonas | 2865 | 3245 | 4958 | 83 | 6109 |
| Bermanella | 11 | 36 | 35 | 9 | 70 |
| Maritalea | 353 | 237 | 233 | 1 | 364 |
| Ruania | 4446 | 4236 | 7581 | 336 | 10436 |
| Spongiibacter | 397 | 762 | 655 | 19 | 931 |
| Pyramidobacter | 431 | 547 | 818 | 4 | 831 |
| Inhella | 890 | 1144 | 1221 | 15 | 1787 |
| Gordonibacter | 922 | 1596 | 2016 | 11 | 2683 |
| Solibacillus | 0 | 0 | 0 | 0 | 0 |
| Rummeliibacillus | 12 | 70 | 48 | 0 | 83 |
| Filimonas | 0 | 0 | 0 | 0 | 0 |
| Lacibacter | 0 | 0 | 0 | 0 | 0 |
| Kosmotoga | 37 | 108 | 56 | 18 | 94 |
| Paenisporosarcina | 37 | 204 | 175 | 0 | 210 |
| Wenxinia | 3 | 16 | 34 | 1 | 41 |
| Candidatus Thioglobus | 17 | 75 | 28 | 4 | 31 |
| Cohaesibacter | 0 | 0 | 0 | 0 | 0 |
| Candidatus Koribacter | 2385 | 5501 | 4215 | 95 | 4774 |
| Paraoerskovia | 1257 | 1603 | 2256 | 53 | 2296 |
| Limnohabitans | 446 | 1957 | 651 | 35 | 1097 |
| Phycisphaera | 1664 | 3746 | 3096 | 64 | 4469 |
| Geminocystis | 57 | 174 | 98 | 3 | 135 |
| Dehalogenimonas | 378 | 893 | 1054 | 6 | 1364 |
| Mycobacteroides | 8323 | 12212 | 15220 | 0 | 24683 |
| Plantactinospora | 9542 | 6701 | 20395 | 1097 | 27099 |
| Alloactinosynnema | 23905 | 4436 | 23694 | 455 | 15278 |
| Ilumatobacter | 4937 | 2886 | 5294 | 66 | 4603 |
| Kinneretia | 4514 | 5606 | 5713 | 35 | 9345 |
| Caldisericum | 62 | 153 | 155 | 1 | 258 |
| Hoyosella | 818 | 1179 | 1233 | 54 | 1952 |
| Basfia | 12 | 21 | 32 | 0 | 62 |
| Cellulosilyticum | 18 | 16 | 37 | 0 | 39 |
| Anaerobacillus | 15 | 45 | 119 | 3 | 34 |
| Tomitella | 3002 | 3428 | 5213 | 220 | 6571 |
| Luteipulveratus | 2417 | 2153 | 3900 | 146 | 4283 |
| Gallaecimonas | 53 | 102 | 100 | 16 | 154 |
| Dolichospermum | 310 | 1093 | 816 | 41 | 1699 |
| Sphaerospermopsis | 0 | 0 | 0 | 0 | 0 |
| Luteimicrobium | 2379 | 2474 | 3425 | 77 | 3746 |
| Methylovulum | 138 | 473 | 269 | 6 | 374 |
| Muriicola | 28 | 61 | 100 | 0 | 73 |
| Candidatus Puniceispirillum | 147 | 128 | 84 | 0 | 260 |
| Ignavibacterium | 0 | 0 | 0 | 0 | 0 |
| Fibrisoma | 12 | 7 | 3 | 0 | 0 |
| Fibrella | 692 | 1498 | 1254 | 21 | 2009 |
| Marivirga | 50 | 322 | 81 | 0 | 155 |
| Celeribacter | 2545 | 3370 | 3690 | 62 | 4569 |
| Herbiconiux | 2846 | 3390 | 4243 | 63 | 4931 |
| Jeongeupia | 1841 | 2854 | 2754 | 37 | 4364 |
| Psychrosphaera | 12 | 98 | 81 | 61 | 103 |
| Phytohabitans | 14759 | 9610 | 29609 | 805 | 31551 |
| Euzebya | 2296 | 2834 | 4310 | 138 | 4499 |
| Yimella | 1019 | 1205 | 1964 | 137 | 2776 |
| Phocaeicola | 0 | 0 | 0 | 0 | 0 |
| Solitalea | 104 | 201 | 225 | 0 | 254 |
| Sulfuricella | 544 | 1102 | 1048 | 14 | 1812 |
| Gilvimarinus | 138 | 224 | 181 | 15 | 255 |
| Granulicella | 5018 | 13205 | 11953 | 90 | 12091 |
| Flavonifractor | 515 | 985 | 1312 | 15 | 1501 |
| Miniimonas | 3046 | 3219 | 5366 | 234 | 7231 |
| Ponticoccus | 839 | 1111 | 1130 | 0 | 1368 |
| Acidiferrobacter | 1088 | 2133 | 1763 | 35 | 2277 |
| Christensenella | 564 | 1149 | 1183 | 4 | 1782 |
| Polymorphum | 3857 | 3499 | 3228 | 71 | 4952 |
| Flavihumibacter | 537 | 1046 | 749 | 16 | 944 |
| Ferruginibacter | 0 | 0 | 0 | 0 | 0 |
| Fimbriimonas | 864 | 2365 | 2097 | 14 | 2787 |
| Caldinitratiruptor | 1520 | 2711 | 3461 | 59 | 4205 |
| Candidatus Moranella | 29 | 28 | 30 | 1 | 43 |
| Sulfuritalea | 1284 | 1853 | 1894 | 75 | 3832 |
| Aeribacillus | 0 | 0 | 0 | 0 | 0 |
| Sandaracinus | 8480 | 16452 | 15912 | 210 | 17469 |
| Chitinolyticbacter | 1002 | 1266 | 1178 | 24 | 2214 |
| Trueperella | 1247 | 2094 | 2631 | 91 | 2828 |
| Mycolicibacter | 11962 | 20998 | 17941 | 1016 | 32968 |
| Candidatus Steffania | 21 | 6 | 14 | 0 | 19 |
| Chthonomonas | 160 | 488 | 618 | 11 | 729 |
| Commensalibacter | 0 | 25 | 39 | 0 | 76 |
| Candidatus Schneideria | 0 | 7 | 8 | 0 | 5 |
| Lonsdalea | 0 | 0 | 0 | 0 | 0 |
| Pelagibacterium | 3116 | 2182 | 2101 | 46 | 2538 |
| Oceanicoccus | 41 | 121 | 57 | 9 | 77 |
| Propioniciclava | 4035 | 5586 | 8197 | 324 | 9658 |
| Paenalcaligenes | 35 | 93 | 109 | 69 | 229 |
| Kyrpidia | 517 | 1052 | 915 | 33 | 1072 |
| Melioribacter | 96 | 260 | 197 | 1 | 181 |
| Allokutzneria | 16418 | 4372 | 14706 | 431 | 12332 |
| Paralcaligenes | 551 | 681 | 682 | 35 | 1247 |
| Moorena | 341 | 1065 | 563 | 8 | 885 |
| Methylovirgula | 3442 | 2878 | 2643 | 11 | 3573 |
| Auritidibacter | 181 | 270 | 349 | 54 | 831 |
| Murdochiella | 75 | 223 | 203 | 1 | 334 |
| Imtechella | 1 | 25 | 21 | 0 | 16 |
| Catenovulum | 22 | 65 | 43 | 18 | 106 |
| Thermotomaculum | 53 | 144 | 163 | 0 | 121 |
| Candidatus Profftia | 5 | 35 | 7 | 0 | 17 |
| Magnetospira | 830 | 683 | 745 | 7 | 1099 |
| Mesotoga | 133 | 151 | 147 | 0 | 278 |
| Austwickia | 601 | 724 | 1083 | 45 | 1349 |
| Gloeomargarita | 81 | 160 | 169 | 1 | 190 |
| Alkalitalea | 26 | 48 | 55 | 0 | 60 |
| Snodgrassella | 16 | 38 | 28 | 0 | 25 |
| Gryllotalpicola | 1690 | 1769 | 2348 | 36 | 2456 |
| Siansivirga | 16 | 17 | 15 | 0 | 14 |
| Flavivirga | 6 | 23 | 22 | 2 | 31 |
| Roseicitreum | 0 | 0 | 0 | 0 | 0 |
| Halioglobus | 486 | 722 | 733 | 21 | 810 |
| Metallibacterium | 75 | 239 | 152 | 5 | 265 |
| Neokomagataea | 112 | 197 | 75 | 0 | 120 |
| Sphingomicrobium | 1524 | 1875 | 1493 | 20 | 2673 |
| Glaciimonas | 183 | 380 | 282 | 0 | 508 |
| Marisediminicola | 876 | 1740 | 1742 | 72 | 2298 |
| Ferrovibrio | 3245 | 2361 | 2413 | 45 | 3881 |
| Parasphingopyxis | 1368 | 1800 | 1299 | 18 | 3010 |
| Gayadomonas | 0 | 0 | 0 | 0 | 0 |
| Otariodibacter | 0 | 10 | 6 | 1 | 12 |
| Oceanotoga | 68 | 85 | 74 | 0 | 30 |
| Candidatus Endolissoclinum | 48 | 121 | 57 | 13 | 312 |
| Thiohalobacter | 1506 | 2221 | 1943 | 28 | 2789 |
| Kroppenstedtia | 310 | 712 | 514 | 28 | 838 |
| Auraticoccus | 1530 | 2284 | 3734 | 155 | 5150 |
| Euzebyella | 68 | 57 | 74 | 0 | 50 |
| Croceicoccus | 5137 | 6504 | 5736 | 129 | 8135 |
| Methylogaea | 851 | 1688 | 1340 | 17 | 1629 |
| Candidatus Nasuia | 17 | 26 | 20 | 0 | 79 |
| Candidatus Annandia | 0 | 0 | 0 | 0 | 0 |
| Lelliottia | 444 | 948 | 828 | 25 | 1277 |
| Pluralibacter | 685 | 1248 | 1356 | 9 | 1992 |
| Kosakonia | 1234 | 2254 | 2139 | 32 | 3332 |
| Candidatus Saccharimonas | 0 | 0 | 0 | 0 | 0 |
| Chryseoglobus | 1151 | 1603 | 2014 | 41 | 2082 |
| Shimwellia | 0 | 0 | 0 | 0 | 0 |
| Frischella | 3 | 22 | 15 | 0 | 10 |
| Spiribacter | 3558 | 4862 | 4825 | 84 | 6345 |
| Noviherbaspirillum | 1091 | 2249 | 1878 | 5 | 3239 |
| Polycladomyces | 120 | 374 | 336 | 3 | 350 |
| Roseimicrobium | 2131 | 5248 | 4581 | 25 | 4156 |
| Coprobacter | 53 | 67 | 73 | 0 | 52 |
| Keratinibaculum | 315 | 41 | 100 | 2 | 97 |
| Rufibacter | 328 | 893 | 832 | 3 | 1491 |
| Candidatus Profftella | 0 | 0 | 0 | 0 | 0 |
| Telmatocola | 1117 | 6372 | 3297 | 132 | 5890 |
| Intestinimonas | 376 | 584 | 734 | 6 | 912 |
| Tardiphaga | 13426 | 9995 | 8349 | 259 | 11386 |
| Allobacillus | 8 | 48 | 20 | 1 | 65 |
| Bythopirellula | 1472 | 2765 | 1874 | 14 | 3373 |
| Breoghania | 2978 | 2510 | 2424 | 85 | 3467 |
| Litorilituus | 50 | 18 | 16 | 4 | 20 |
| Endomicrobium | 64 | 86 | 70 | 3 | 95 |
| Glycocaulis | 570 | 680 | 653 | 29 | 773 |
| Actibacterium | 1165 | 1225 | 1021 | 0 | 1443 |
| Chryseolinea | 1313 | 2060 | 1432 | 6 | 1857 |
| Cruoricaptor | 55 | 180 | 115 | 0 | 109 |
| Diaminobutyricimonas | 854 | 1302 | 1624 | 29 | 1786 |
| Falsirhodobacter | 651 | 712 | 660 | 31 | 1102 |
| Fretibacterium | 0 | 0 | 0 | 0 | 0 |
| Jatrophihabitans | 6743 | 6043 | 11839 | 319 | 14828 |
| Komagataeibacter | 3870 | 4698 | 3781 | 81 | 5976 |
| Mameliella | 973 | 1064 | 952 | 13 | 1547 |
| Oceanirhabdus | 16 | 24 | 34 | 0 | 28 |
| Pontimonas | 0 | 0 | 0 | 0 | 0 |
| Salisediminibacterium | 139 | 385 | 316 | 0 | 558 |
| Sinomicrobium | 65 | 127 | 92 | 2 | 118 |
| Sphingorhabdus | 1374 | 1801 | 1475 | 0 | 2601 |
| Zhongshania | 89 | 117 | 119 | 9 | 171 |
| Candidatus Symbiobacter | 0 | 0 | 0 | 0 | 0 |
| Ferriphaselus | 412 | 637 | 524 | 21 | 1280 |
| Tabrizicola | 859 | 1014 | 924 | 2 | 1183 |
| Gynuella | 119 | 182 | 192 | 11 | 274 |
| Candidatus Atelocyanobacterium | 9 | 39 | 19 | 1 | 18 |
| Tahibacter | 1869 | 2417 | 2271 | 31 | 2746 |
| Jeotgalibaca | 0 | 0 | 0 | 0 | 0 |
| Draconibacterium | 75 | 146 | 180 | 1 | 178 |
| Basilea | 0 | 0 | 0 | 0 | 0 |
| Peptoclostridium | 38 | 76 | 84 | 0 | 99 |
| Methyloceanibacter | 5707 | 4191 | 4148 | 47 | 4914 |
| Methyloligella | 3773 | 2535 | 2897 | 58 | 3152 |
| Oxynema | 168 | 549 | 447 | 6 | 571 |
| Effusibacillus | 47 | 195 | 156 | 1 | 178 |
| Pseudocitrobacter | 91 | 206 | 138 | 3 | 194 |
| Terrisporobacter | 35 | 30 | 11 | 0 | 11 |
| Gottschalkia | 122 | 46 | 58 | 21 | 44 |
| Lachnoclostridium | 377 | 945 | 834 | 37 | 1132 |
| Polymorphobacter | 2142 | 2737 | 2687 | 9 | 3813 |
| Ruminiclostridium | 34 | 113 | 36 | 1 | 62 |
| Candidatus Nucleicultrix | 43 | 72 | 54 | 0 | 63 |
| Aquisphaera | 8663 | 45643 | 22802 | 295 | 33290 |
| Defluviitoga | 15 | 33 | 57 | 0 | 38 |
| Mongoliitalea | 53 | 66 | 61 | 0 | 99 |
| Wenyingzhuangia | 0 | 0 | 0 | 0 | 0 |
| Thalassotalea | 91 | 149 | 124 | 10 | 184 |
| Vespertiliibacter | 0 | 1 | 5 | 0 | 1 |
| Candidatus Paracaedibacter | 73 | 85 | 48 | 0 | 78 |
| Flaviflexus | 1294 | 1586 | 2417 | 84 | 3026 |
| Thermosulfurimonas | 150 | 314 | 375 | 2 | 472 |
| Pseudoduganella | 10399 | 15949 | 15149 | 167 | 30943 |
| Vulgatibacter | 2540 | 4140 | 5176 | 48 | 5244 |
| Labilithrix | 3299 | 6950 | 6813 | 48 | 6569 |
| Pseudohongiella | 74 | 206 | 203 | 22 | 293 |
| Neorhizobium | 6146 | 6153 | 5415 | 180 | 8428 |
| Bacterioplanes | 0 | 0 | 0 | 0 | 0 |
| Candidatus Phaeomarinobacter | 743 | 660 | 565 | 2 | 811 |
| Methylacidimicrobium | 1219 | 2831 | 2150 | 28 | 2809 |
| Candidatus Ruthia | 29 | 41 | 31 | 0 | 68 |
| Niveispirillum | 1120 | 1352 | 1336 | 17 | 2291 |
| Nitrospirillum | 2663 | 3012 | 2986 | 95 | 4090 |
| Mumia | 3602 | 4886 | 5556 | 244 | 5693 |
| Candidatus Babela | 88 | 225 | 85 | 0 | 91 |
| Rouxiella | 336 | 506 | 379 | 23 | 701 |
| Hartmannibacter | 3067 | 2968 | 2504 | 35 | 3449 |
| Sabulilitoribacter | 1 | 24 | 19 | 0 | 14 |
| Paradevosia | 3342 | 2395 | 2325 | 75 | 2754 |
| Faecalitalea | 15 | 68 | 35 | 0 | 22 |
| Rubripirellula | 931 | 2867 | 1891 | 52 | 3129 |
| Roseimaritima | 2117 | 5883 | 3629 | 49 | 6201 |
| Oricola | 1402 | 1381 | 1403 | 46 | 2163 |
| Parasaccharibacter | 86 | 155 | 168 | 0 | 261 |
| Thiolapillus | 187 | 509 | 368 | 53 | 516 |
| Hydrogenibacillus | 929 | 1614 | 1708 | 19 | 2113 |
| Pararhodospirillum | 599 | 1010 | 1104 | 16 | 1797 |
| Pararhizobium | 3960 | 4139 | 3916 | 59 | 5568 |
| Salinispira | 79 | 143 | 126 | 11 | 158 |
| Paraglaciecola | 0 | 0 | 0 | 0 | 0 |
| Phreatobacter | 19349 | 11833 | 12342 | 271 | 16874 |
| Mageeibacillus | 8 | 41 | 196 | 0 | 69 |
| Pseudothermotoga | 0 | 0 | 0 | 0 | 0 |
| Aestuariispira | 525 | 615 | 523 | 3 | 837 |
| Aquihabitans | 17001 | 13062 | 29708 | 282 | 24229 |
| Ciceribacter | 1446 | 1398 | 1494 | 15 | 2160 |
| Siccibacter | 0 | 0 | 0 | 0 | 0 |
| Frederiksenia | 0 | 17 | 17 | 9 | 11 |
| Gimesia | 4136 | 17508 | 10061 | 209 | 17389 |
| Glaciihabitans | 681 | 1462 | 1291 | 122 | 1943 |
| Homoserinibacter | 1192 | 1733 | 2178 | 66 | 2524 |
| Hungatella | 0 | 0 | 0 | 0 | 0 |
| Lentilitoribacter | 72 | 87 | 81 | 0 | 131 |
| Marinicauda | 2056 | 2587 | 2842 | 100 | 3959 |
| Melaminivora | 2489 | 3445 | 3778 | 21 | 5363 |
| Nibribacter | 147 | 289 | 233 | 4 | 363 |
| Paludibaculum | 4415 | 11275 | 8065 | 121 | 10088 |
| Pelolinea | 195 | 507 | 436 | 2 | 469 |
| Permianibacter | 375 | 579 | 567 | 1 | 684 |
| Planctopirus | 754 | 3193 | 1705 | 42 | 2812 |
| Pseudopedobacter | 44 | 98 | 79 | 4 | 117 |
| Rhizorhabdus | 10678 | 10927 | 9992 | 200 | 19752 |
| Rubinisphaera | 824 | 2978 | 1991 | 16 | 3279 |
| Seonamhaeicola | 0 | 0 | 0 | 0 | 0 |
| Swingsia | 29 | 19 | 15 | 0 | 43 |
| Variibacter | 6464 | 3136 | 3174 | 94 | 5073 |
| Halobacteriovorax | 16 | 64 | 33 | 2 | 71 |
| Actinotignum | 0 | 0 | 0 | 0 | 0 |
| Cereibacter | 3991 | 5314 | 5609 | 95 | 7203 |
| Crenobacter | 1508 | 2168 | 2227 | 107 | 4783 |
| Aliiroseovarius | 766 | 789 | 747 | 6 | 962 |
| Herbinix | 18 | 36 | 30 | 2 | 45 |
| Thermogutta | 540 | 1670 | 1395 | 12 | 2337 |
| Wenzhouxiangella | 1883 | 3159 | 3035 | 48 | 3950 |
| Limnochorda | 1110 | 2247 | 2658 | 38 | 3427 |
| Novibacillus | 142 | 433 | 282 | 2 | 427 |
| Candidatus Methylopumilus | 87 | 209 | 181 | 7 | 237 |
| Pseudooceanicola | 1222 | 1313 | 1400 | 15 | 2016 |
| Candidatus Tachikawaea | 4 | 4 | 7 | 0 | 6 |
| Sulfurifustis | 3306 | 5397 | 4980 | 61 | 6777 |
| Aurantimicrobium | 107 | 342 | 266 | 16 | 442 |
| Sediminicoccus | 1775 | 1829 | 1739 | 19 | 2639 |
| Gemmatirosa | 12364 | 40788 | 29330 | 285 | 92575 |
| Faecalibaculum | 121 | 138 | 139 | 1 | 286 |
| Pseudorhodoplanes | 15984 | 4379 | 4570 | 60 | 8153 |
| Caproiciproducens | 181 | 334 | 433 | 7 | 630 |
| Woeseia | 983 | 1004 | 899 | 4 | 1097 |
| Pyruvatibacter | 1451 | 1310 | 1245 | 0 | 1601 |
| Occallatibacter | 1864 | 4841 | 3898 | 23 | 4471 |
| Glutamicibacter | 3904 | 5336 | 7794 | 356 | 12288 |
| Paeniglutamicibacter | 912 | 1273 | 1785 | 86 | 2690 |
| Paenarthrobacter | 3263 | 4812 | 8835 | 138 | 12131 |
| Pseudarthrobacter | 10371 | 34878 | 37340 | 790 | 59111 |
| Sulfuricaulis | 1844 | 2422 | 2374 | 38 | 3380 |
| Chania | 120 | 127 | 112 | 2 | 163 |
| Egibacter | 2281 | 2972 | 4431 | 93 | 4686 |
| Lacimicrobium | 108 | 186 | 142 | 9 | 256 |
| Egicoccus | 2565 | 3100 | 4496 | 181 | 5111 |
| Ichthyobacterium | 0 | 0 | 0 | 0 | 0 |
| Cnuibacter | 1672 | 1730 | 2754 | 43 | 3193 |
| Pseudohalocynthiibacter | 165 | 810 | 435 | 4 | 604 |
| Methylomagnum | 996 | 1754 | 1475 | 56 | 2324 |
| Paludisphaera | 8027 | 37151 | 19026 | 370 | 29269 |
| Hankyongella | 1489 | 1465 | 1393 | 15 | 2053 |
| Acidihalobacter | 2511 | 3846 | 3601 | 45 | 4354 |
| Anoxybacter | 30 | 55 | 61 | 0 | 79 |
| Arachidicoccus | 0 | 0 | 0 | 0 | 0 |
| Sellimonas | 110 | 97 | 123 | 0 | 72 |
| Athalassotoga | 32 | 57 | 54 | 2 | 79 |
| Niveibacterium | 1216 | 1451 | 1742 | 17 | 2574 |
| Hathewaya | 54 | 17 | 18 | 1 | 19 |
| Dissulfurimicrobium | 0 | 0 | 0 | 0 | 0 |
| Anseongella | 133 | 355 | 266 | 2 | 361 |
| Jiella | 2931 | 2387 | 2371 | 58 | 3442 |
| Frigidibacter | 1453 | 1611 | 2132 | 3 | 2764 |
| Apibacter | 36 | 65 | 70 | 0 | 81 |
| Sulfuriferula | 1209 | 2400 | 2173 | 63 | 3447 |
| Bradymonas | 502 | 1363 | 1156 | 14 | 1598 |
| Izhakiella | 0 | 0 | 0 | 0 | 12 |
| Marinagarivorans | 56 | 88 | 56 | 2 | 88 |
| Aquisalinus | 772 | 850 | 714 | 41 | 1123 |
| Candidatus Fokinia | 39 | 24 | 13 | 0 | 27 |
| Haematospirillum | 123 | 300 | 213 | 0 | 266 |
| Candidatus Methylospira | 367 | 687 | 538 | 19 | 841 |
| Mycoavidus | 152 | 497 | 398 | 3 | 570 |
| Paraburkholderia | 37380 | 52776 | 51403 | 692 | 70792 |
| Caballeronia | 10468 | 14191 | 13801 | 187 | 20195 |
| Youhaiella | 2243 | 2152 | 1756 | 27 | 2472 |
| Anaerocolumna | 21 | 189 | 72 | 0 | 80 |
| Pontivivens | 1201 | 1376 | 1588 | 15 | 1744 |
| Paraclostridium | 34 | 24 | 9 | 0 | 16 |
| Brevirhabdus | 934 | 1549 | 1475 | 6 | 2130 |
| Qipengyuania | 6092 | 8000 | 7366 | 136 | 11666 |
| Alkalimarinus | 229 | 180 | 169 | 57 | 203 |
| Sulfuriflexus | 196 | 359 | 388 | 4 | 417 |
| Pseudobacter | 0 | 0 | 0 | 0 | 0 |
| Sinimarinibacterium | 800 | 1303 | 1065 | 7 | 1018 |
| Mycolicibacterium | 170495 | 492651 | 335173 | 4964 | 785017 |
| Allofrancisella | 17 | 23 | 19 | 1 | 17 |
| Clostridioides | 1013 | 176 | 1597 | 4 | 1993 |
| Kovacikia | 419 | 1744 | 1093 | 2 | 1347 |
| Panacibacter | 0 | 0 | 0 | 0 | 0 |
| Pajaroellobacter | 67 | 141 | 161 | 2 | 157 |
| Salipaludibacillus | 4 | 78 | 15 | 0 | 19 |
| Serpentinicella | 4 | 17 | 6 | 0 | 14 |
| Pistricoccus | 0 | 0 | 0 | 0 | 0 |
| Candidatus Desulfofervidus | 60 | 117 | 165 | 0 | 220 |
| Atlantibacter | 0 | 0 | 0 | 0 | 0 |
| Pseudorhizobium | 1869 | 2275 | 2227 | 23 | 3298 |
| Acuticoccus | 3390 | 3863 | 4154 | 122 | 5999 |
| Ruthenibacterium | 0 | 0 | 0 | 0 | 0 |
| Candidatus Doolittlea | 9 | 20 | 25 | 1 | 48 |
| Candidatus Hoaglandella | 4 | 11 | 12 | 0 | 17 |
| Candidatus Mikella | 5 | 7 | 16 | 0 | 13 |
| Candidatus Gullanella | 9 | 8 | 2 | 0 | 4 |
| Parageobacillus | 0 | 0 | 0 | 0 | 0 |
| Raineyella | 1201 | 1614 | 2386 | 101 | 2602 |
| Sediminispirochaeta | 52 | 105 | 83 | 0 | 139 |
| Oryzomicrobium | 892 | 1209 | 1308 | 28 | 2123 |
| Acidipropionibacterium | 3517 | 5157 | 7586 | 424 | 12732 |
| Cutibacterium | 5809 | 219 | 912 | 0 | 5150 |
| Rugosibacter | 318 | 1024 | 843 | 2 | 1222 |
| Roseitalea | 1818 | 2032 | 1913 | 31 | 2581 |
| Acutalibacter | 119 | 243 | 285 | 5 | 319 |
| Flintibacter | 141 | 282 | 308 | 6 | 423 |
| Longicatena | 0 | 0 | 0 | 0 | 0 |
| Muribaculum | 89 | 359 | 269 | 13 | 366 |
| Turicimonas | 0 | 0 | 0 | 0 | 0 |
| Orrella | 1756 | 2421 | 2533 | 48 | 3607 |
| Kiritimatiella | 1886 | 4943 | 3909 | 11 | 5156 |
| Pseudactinotalea | 1171 | 1487 | 2077 | 41 | 2254 |
| Emergencia | 100 | 188 | 117 | 2 | 154 |
| Raoultibacter | 303 | 600 | 500 | 0 | 872 |
| Candidatus Fukatsuia | 0 | 0 | 0 | 0 | 0 |
| Floricoccus | 0 | 0 | 0 | 0 | 0 |
| Ndongobacter | 102 | 149 | 155 | 3 | 390 |
| Immundisolibacter | 1861 | 2806 | 2653 | 31 | 3513 |
| Fuerstiella | 656 | 2775 | 1698 | 21 | 2708 |
| Bernardetia | 48 | 36 | 67 | 0 | 52 |
| Sulfuritortus | 1780 | 2621 | 2834 | 107 | 4132 |
| Candidatus Walczuchella | 11 | 6 | 7 | 0 | 25 |
| Abyssicoccus | 0 | 0 | 0 | 0 | 0 |
| Silicimonas | 994 | 1245 | 1185 | 27 | 1701 |
| Rodentibacter | 0 | 0 | 0 | 0 | 0 |
| Lacunisphaera | 1730 | 4270 | 4397 | 31 | 4178 |
| Nissabacter | 345 | 631 | 555 | 2 | 737 |
| Salinivirga | 26 | 41 | 36 | 0 | 37 |
| Candidatus Nitrosoglobus | 16 | 38 | 37 | 4 | 25 |
| Phoenicibacter | 0 | 0 | 0 | 0 | 0 |
| Candidatus Promineofilum | 1477 | 3667 | 3304 | 35 | 4537 |
| Luteitalea | 46970 | 120942 | 80232 | 501 | 82992 |
| Faecalicatena | 23 | 22 | 49 | 0 | 37 |
| Mariniblastus | 504 | 1689 | 1170 | 15 | 1783 |
| Arabiibacter | 478 | 861 | 935 | 30 | 1417 |
| Thaumasiovibrio | 9 | 68 | 55 | 1 | 89 |
| Candidatus Vallotia | 142 | 185 | 223 | 1 | 309 |
| Brevefilum | 85 | 245 | 234 | 18 | 385 |
| Koinonema | 165 | 645 | 344 | 4 | 673 |
| Silvanigrella | 12 | 136 | 27 | 1 | 54 |
| Ketobacter | 0 | 0 | 0 | 0 | 0 |
| Ereboglobus | 870 | 2425 | 1859 | 22 | 2059 |
| Thermosulfuriphilus | 0 | 0 | 0 | 0 | 0 |
| Sulfurivermis | 1495 | 1789 | 2028 | 17 | 1921 |
| Oceanispirochaeta | 20 | 26 | 31 | 2 | 50 |
| Guyparkeria | 1222 | 1714 | 1765 | 9 | 1855 |
| Pseudodesulfovibrio | 3883 | 6896 | 7240 | 158 | 9659 |
| Agarilytica | 55 | 55 | 57 | 3 | 184 |
| Hyphococcus | 460 | 403 | 387 | 27 | 505 |
| Anaerotignum | 0 | 0 | 0 | 0 | 0 |
| Monoglobus | 8 | 66 | 41 | 0 | 24 |
| Candidatus Nanopelagicus | 82 | 69 | 71 | 5 | 155 |
| Thiomicrorhabdus | 138 | 325 | 288 | 31 | 330 |
| Allobranchiibius | 1383 | 1828 | 2656 | 127 | 3909 |
| Chenggangzhangella | 4620 | 3805 | 3565 | 138 | 5910 |
| Paraphotobacterium | 4 | 9 | 14 | 0 | 3 |
| Candidatus Fonsibacter | 3 | 15 | 17 | 0 | 95 |
| Changchengzhania | 23 | 32 | 17 | 0 | 34 |
| Tuwongella | 1107 | 6929 | 3780 | 52 | 6630 |
| Metakosakonia | 0 | 0 | 0 | 0 | 43 |
| Pseudescherichia | 160 | 193 | 286 | 2 | 466 |
| Antarcticibacterium | 0 | 0 | 0 | 0 | 0 |
| Aminipila | 55 | 292 | 159 | 0 | 212 |
| Labilibaculum | 34 | 53 | 45 | 2 | 65 |
| Bacterioplanoides | 49 | 87 | 60 | 4 | 82 |
| Psychromicrobium | 246 | 280 | 468 | 14 | 655 |
| Epidermidibacterium | 2377 | 3081 | 5920 | 300 | 6608 |
| Parolsenella | 618 | 1393 | 1571 | 9 | 1837 |
| Tritonibacter | 632 | 891 | 633 | 0 | 948 |
| Methylomusa | 89 | 130 | 122 | 0 | 194 |
| Candidatus Nanosynbacter | 102 | 134 | 89 | 0 | 276 |
| Glaesserella | 64 | 63 | 76 | 1 | 125 |
| Mixta | 883 | 1263 | 1315 | 36 | 1797 |
| Candidatus Phycorickettsia | 18 | 13 | 19 | 0 | 28 |
| Mycolicibacillus | 2967 | 4878 | 5104 | 173 | 8134 |
| Miniphocaeibacter | 0 | 0 | 0 | 0 | 0 |
| Limnobaculum | 34 | 25 | 37 | 2 | 60 |
| Arcticibacterium | 18 | 24 | 26 | 0 | 43 |
| Ruficoccus | 426 | 1165 | 1043 | 8 | 1153 |
| Limimaricola | 55 | 32 | 54 | 0 | 65 |
| Yoonia | 511 | 470 | 463 | 1 | 546 |
| Ahniella | 897 | 1392 | 1129 | 0 | 1155 |
| Ferrigenium | 709 | 1222 | 1220 | 9 | 2076 |
| Candidatus Bipolaricaulis | 385 | 694 | 709 | 4 | 900 |
| Methylorubrum | 15619 | 14433 | 14132 | 433 | 18769 |
| Desulfofarcimen | 26 | 82 | 87 | 0 | 165 |
| Candidatus Syntrophocurvum | 4 | 10 | 21 | 0 | 20 |
| Thermoleptolyngbya | 186 | 754 | 796 | 2 | 969 |
| Thermoclostridium | 59 | 108 | 75 | 1 | 125 |
| Pseudoclostridium | 34 | 69 | 70 | 1 | 51 |
| Mediterraneibacter | 5388 | 321 | 2018 | 0 | 4161 |
| Halarcobacter | 2 | 22 | 9 | 3 | 16 |
| Indioceanicola | 2010 | 2088 | 2110 | 4 | 3234 |
| Sedimentisphaera | 245 | 835 | 342 | 17 | 370 |
| Cyclonatronum | 175 | 647 | 310 | 15 | 659 |
| Serpentinimonas | 1747 | 2053 | 2460 | 46 | 3821 |
| Duncaniella | 33 | 88 | 107 | 0 | 107 |
| Schaalia | 2401 | 3199 | 4615 | 197 | 6228 |
| Rippkaea | 44 | 99 | 99 | 0 | 169 |
| Parashewanella | 22 | 49 | 35 | 11 | 70 |
| Mycetohabitans | 479 | 842 | 728 | 2 | 1162 |
| Caproicibacter | 122 | 328 | 222 | 2 | 297 |
| Nibricoccus | 951 | 2760 | 2179 | 12 | 2694 |
| Desulfocurvibacter | 668 | 1098 | 1362 | 7 | 1488 |
| Dysosmobacter | 642 | 1284 | 1580 | 18 | 1820 |
| Aquella | 25 | 37 | 19 | 2 | 24 |
| Limnospira | 0 | 0 | 0 | 0 | 0 |
| Baekduia | 18436 | 15984 | 37724 | 308 | 41121 |
| Candidatus Cytomitobacter | 10 | 36 | 46 | 0 | 36 |
| Candidatus Sneabacter | 49 | 8 | 16 | 0 | 22 |
| Candidatus Nesciobacter | 6 | 7 | 4 | 0 | 12 |
| Candidatus Deianiraea | 3 | 14 | 4 | 0 | 14 |
| Petrocella | 4 | 27 | 24 | 1 | 19 |
| Acidibrevibacterium | 1798 | 2020 | 1759 | 20 | 3115 |
| Acidisarcina | 1218 | 3656 | 2717 | 7 | 3397 |
| Pseudopuniceibacterium | 714 | 589 | 624 | 3 | 975 |
| Pradoshia | 5 | 30 | 18 | 0 | 3 |
| Thermanaerosceptrum | 33 | 202 | 167 | 1 | 150 |
| Geomonas | 4883 | 10298 | 10207 | 149 | 12959 |
| Candidatus Hydrogenosomobacter | 9 | 20 | 35 | 0 | 31 |
| Georhizobium | 1871 | 1778 | 1526 | 57 | 2300 |
| Saliniradius | 65 | 146 | 223 | 2 | 177 |
| Leptodesmis | 60 | 352 | 337 | 1 | 576 |
| Tellurirhabdus | 563 | 1296 | 1028 | 38 | 1604 |
| Peribacillus | 1428 | 7349 | 2571 | 33 | 3497 |
| Cytobacillus | 0 | 0 | 0 | 0 | 0 |
| Mesobacillus | 135 | 273 | 362 | 1 | 443 |
| Sphingosinithalassobacter | 2243 | 2518 | 2248 | 38 | 4315 |
| Aquirufa | 165 | 198 | 212 | 0 | 271 |
| Croceibacterium | 615 | 625 | 616 | 15 | 1224 |
| Aquisediminimonas | 0 | 0 | 0 | 0 | 0 |
| Maribellus | 51 | 124 | 46 | 0 | 67 |
| Kineobactrum | 632 | 801 | 766 | 9 | 1229 |
| Faecalibacillus | 0 | 0 | 0 | 0 | 0 |
| Pulveribacter | 909 | 1160 | 1165 | 3 | 1783 |
| Intestinibaculum | 30 | 30 | 35 | 0 | 38 |
| Protaetiibacter | 3376 | 5348 | 6575 | 293 | 7898 |
| Rhodophyticola | 0 | 0 | 0 | 0 | 0 |
| Tautonia | 7963 | 42793 | 19658 | 248 | 27919 |
| Ephemeroptericola | 105 | 107 | 127 | 3 | 239 |
| Tepidiforma | 2872 | 4635 | 5843 | 47 | 6021 |
| Paroceanicella | 2337 | 2777 | 2763 | 35 | 4162 |
| Salicibibacter | 169 | 366 | 354 | 40 | 690 |
| Tardibacter | 2111 | 2798 | 1749 | 56 | 3607 |
| Suicoccus | 0 | 0 | 0 | 0 | 0 |
| Staphylospora | 308 | 561 | 622 | 12 | 979 |
| Anaerohalosphaera | 235 | 632 | 488 | 2 | 712 |
| Limihaloglobus | 84 | 266 | 321 | 1 | 394 |
| Lacipirellula | 59347 | 39518 | 32755 | 821 | 33931 |
| Gudongella | 53 | 83 | 119 | 20 | 112 |
| Ktedonosporobacter | 345 | 673 | 954 | 2 | 1211 |
| Paraflavitalea | 1898 | 2316 | 952 | 16 | 853 |
| Fluviispira | 49 | 40 | 57 | 1 | 52 |
| Urechidicola | 38 | 23 | 23 | 0 | 41 |
| Hypericibacter | 10009 | 10141 | 11847 | 184 | 24274 |
| Casimicrobium | 1783 | 2641 | 2735 | 11 | 4222 |
| Aureibaculum | 22 | 20 | 14 | 0 | 20 |
| Aerosticca | 3612 | 5005 | 5572 | 70 | 6172 |
| Pseudolysobacter | 1269 | 1852 | 1506 | 6 | 1798 |
| Parasphingorhabdus | 0 | 0 | 0 | 0 | 0 |
| Novosphingopyxis | 1175 | 1177 | 1054 | 2 | 1678 |
| Salaquimonas | 838 | 754 | 632 | 0 | 759 |
| Crateriforma | 1009 | 2257 | 1691 | 16 | 2933 |
| Bremerella | 3046 | 9806 | 6684 | 115 | 11236 |
| Alienimonas | 2783 | 8233 | 5556 | 75 | 8418 |
| Amniculibacterium | 13 | 58 | 39 | 0 | 63 |
| Candidatus Chazhemtobacterium | 20 | 51 | 43 | 2 | 116 |
| Massilistercora | 311 | 310 | 393 | 7 | 941 |
| Lacrimispora | 51 | 142 | 201 | 1 | 283 |
| Enterocloster | 453 | 841 | 773 | 4 | 1240 |
| Lichenihabitans | 2473 | 2750 | 2236 | 92 | 3297 |
| Limnoglobus | 7475 | 55664 | 26522 | 138 | 43091 |
| Candidatus Vesicomyosocius | 19 | 24 | 37 | 0 | 21 |
| Parasedimentitalea | 0 | 0 | 0 | 0 | 0 |
| Candidatus Reidiella | 0 | 0 | 0 | 0 | 0 |
| Pauljensenia | 525 | 571 | 1097 | 48 | 1265 |
| Limosilactobacillus | 524 | 1611 | 1265 | 20 | 1108 |
| Peptacetobacter | 11 | 15 | 42 | 7 | 41 |
| Zhaonella | 38 | 113 | 132 | 2 | 176 |
| Amedibacterium | 20 | 21 | 83 | 0 | 61 |
| Pseudoleptotrichia | 49 | 11 | 19 | 0 | 12 |
| Entomomonas | 0 | 0 | 0 | 0 | 0 |
| Paraconexibacter | 8552 | 6442 | 15533 | 136 | 17639 |
| Faecalibacter | 10 | 8 | 12 | 0 | 14 |
| Persicimonas | 1687 | 3562 | 3680 | 18 | 4809 |
| Boudabousia | 53 | 46 | 76 | 16 | 138 |
| Fannyhessea | 0 | 0 | 0 | 0 | 0 |
| Acetilactobacillus | 0 | 0 | 0 | 0 | 0 |
| Amylolactobacillus | 27 | 35 | 26 | 0 | 18 |
| Apilactobacillus | 21 | 31 | 39 | 0 | 67 |
| Bombilactobacillus | 13 | 27 | 33 | 1 | 23 |
| Companilactobacillus | 41 | 62 | 68 | 1 | 84 |
| Furfurilactobacillus | 17 | 60 | 45 | 1 | 32 |
| Lapidilactobacillus | 0 | 8 | 5 | 0 | 3 |
| Latilactobacillus | 0 | 0 | 0 | 0 | 0 |
| Levilactobacillus | 0 | 0 | 0 | 0 | 0 |
| Ligilactobacillus | 0 | 0 | 0 | 0 | 0 |
| Liquorilactobacillus | 61 | 56 | 78 | 11 | 98 |
| Loigolactobacillus | 27 | 111 | 83 | 0 | 225 |
| Paucilactobacillus | 18 | 39 | 40 | 2 | 28 |
| Schleiferilactobacillus | 95 | 193 | 187 | 0 | 206 |
| Secundilactobacillus | 77 | 105 | 64 | 0 | 70 |
| Terricaulis | 1557 | 2141 | 1605 | 10 | 2261 |
| Spartinivicinus | 30 | 39 | 33 | 13 | 40 |
| Biomaibacter | 48 | 107 | 162 | 1 | 129 |
| Sulfurimicrobium | 968 | 1672 | 1687 | 37 | 3019 |
| Desulfolutivibrio | 587 | 1388 | 1148 | 13 | 1725 |
| Frigoriglobus | 10868 | 109308 | 43698 | 470 | 53609 |
| Tepiditoga | 41 | 10 | 5 | 0 | 14 |
| Profundibacter | 205 | 420 | 265 | 1 | 414 |
| Thiosulfativibrio | 14 | 40 | 27 | 4 | 39 |
| Thiosulfatimonas | 27 | 111 | 74 | 6 | 83 |
| Kaistella | 0 | 0 | 0 | 0 | 0 |
| Radiobacillus | 51 | 45 | 37 | 0 | 36 |
| Koleobacter | 0 | 0 | 0 | 0 | 0 |
| Candidatus Velamenicoccus | 0 | 0 | 0 | 0 | 0 |
| Actinomarinicola | 8838 | 6651 | 14998 | 63 | 12014 |
| Malacoplasma | 22 | 30 | 43 | 1 | 36 |
| Oecophyllibacter | 188 | 268 | 248 | 4 | 356 |
| Thiospirochaeta | 12 | 13 | 14 | 0 | 16 |
| Schnuerera | 538 | 34 | 97 | 49 | 66 |
| Maridesulfovibrio | 190 | 165 | 173 | 4 | 254 |
| Pelagovum | 1180 | 1454 | 1657 | 40 | 2220 |
| Aureliella | 470 | 1836 | 1123 | 23 | 1806 |
| Nitrosophilus | 71 | 94 | 64 | 0 | 113 |
| Polystyrenella | 251 | 1113 | 610 | 3 | 1083 |
| Lignipirellula | 3478 | 11186 | 8458 | 170 | 13998 |
| Calycomorphotria | 625 | 2728 | 1484 | 13 | 2482 |
| Maioricimonas | 3273 | 12655 | 8255 | 105 | 12920 |
| Caulifigura | 6325 | 14850 | 9642 | 73 | 11485 |
| Rosistilla | 2829 | 8747 | 5993 | 62 | 10016 |
| Symmachiella | 1647 | 5864 | 3665 | 46 | 6046 |
| Aeoliella | 2685 | 4779 | 3432 | 29 | 5569 |
| Botrimarina | 6450 | 11397 | 8540 | 99 | 12662 |
| Pirellulimonas | 7258 | 13133 | 10066 | 132 | 15055 |
| Thalassoglobus | 320 | 940 | 630 | 1 | 966 |
| Stieleria | 3413 | 10178 | 7126 | 158 | 12169 |
| Candidatus Nitrosacidococcus | 28 | 62 | 59 | 0 | 43 |
| Priestia | 171 | 1479 | 1094 | 0 | 907 |
| Aurantiacibacter | 1548 | 2163 | 1764 | 42 | 3170 |
| Paraurantiacibacter | 532 | 603 | 555 | 5 | 824 |
| Tsuneonella | 2656 | 3133 | 2863 | 70 | 4431 |
| Arachnia | 1236 | 1845 | 2701 | 100 | 3923 |
| Dongshaea | 118 | 170 | 165 | 4 | 248 |
| Poriferisphaera | 108 | 498 | 236 | 3 | 378 |
| Usitatibacter | 8709 | 11156 | 11332 | 290 | 21238 |
| Lederbergia | 11 | 19 | 15 | 0 | 10 |
| Lichenicola | 1288 | 1696 | 1756 | 7 | 2221 |
| Pedococcus | 1814 | 2561 | 4209 | 163 | 5388 |
| Yinghuangia | 3620 | 3097 | 5547 | 541 | 11106 |
| Anatilimnocola | 3372 | 11852 | 9461 | 189 | 14152 |
| Urbifossiella | 12109 | 112947 | 46608 | 486 | 65058 |
| Natranaerofaba | 71 | 36 | 66 | 0 | 66 |
| Humisphaera | 3105 | 10240 | 7207 | 100 | 10537 |
| Fluviibacter | 0 | 0 | 0 | 0 | 0 |
| Jinshanibacter | 0 | 0 | 0 | 0 | 0 |
| Entomobacter | 17 | 36 | 24 | 0 | 41 |
| Sporofaciens | 0 | 0 | 1 | 0 | 0 |
| Dissulfurispira | 90 | 364 | 200 | 0 | 642 |
| Pseudocnuella | 0 | 0 | 0 | 0 | 0 |
| Syntrophotalea | 749 | 1555 | 1491 | 24 | 1861 |
| Luteithermobacter | 0 | 0 | 0 | 0 | 0 |
| Paremcibacter | 0 | 0 | 0 | 0 | 0 |
| Jejubacter | 0 | 0 | 0 | 0 | 0 |
| Berryella | 1409 | 3096 | 3246 | 23 | 2689 |
| Sodaliphilus | 213 | 442 | 404 | 5 | 667 |
| Qingshengfaniella | 0 | 0 | 0 | 0 | 0 |
| Weizmannia | 0 | 0 | 0 | 0 | 0 |
| Candidatus Azoamicus | 0 | 2 | 3 | 2 | 6 |
| Fuscovulum | 741 | 814 | 765 | 33 | 1269 |
| Chlamydiifrater | 37 | 17 | 19 | 0 | 39 |
| Methylotuvimicrobium | 421 | 1018 | 622 | 33 | 1122 |
| Allosphingosinicella | 1563 | 2416 | 1638 | 99 | 7055 |
| Aquirhabdus | 29 | 155 | 84 | 2 | 170 |
| Caenibius | 2370 | 3247 | 2366 | 21 | 3471 |
| Croceimicrobium | 8 | 62 | 85 | 0 | 181 |
| Occultella | 2083 | 2263 | 3649 | 93 | 4170 |
| Caldichromatium | 591 | 995 | 955 | 13 | 1216 |
| Heliomicrobium | 417 | 991 | 1030 | 0 | 1157 |
| Candidatus Mycosynbacter | 14 | 43 | 44 | 0 | 73 |
| Pukyongia | 34 | 74 | 33 | 1 | 36 |
| Pukyongiella | 923 | 1179 | 1203 | 18 | 2099 |
| Pikeienuella | 1730 | 2028 | 1936 | 5 | 2755 |
| Caproicibacterium | 174 | 256 | 244 | 2 | 286 |
| Entomospira | 17 | 22 | 23 | 1 | 57 |
| Flocculibacter | 16 | 29 | 23 | 4 | 28 |
| Phnomibacter | 0 | 0 | 0 | 0 | 0 |
| Evansella | 46 | 84 | 79 | 0 | 140 |
| Gottfriedia | 0 | 0 | 0 | 0 | 0 |
| Heyndrickxia | 84 | 89 | 117 | 0 | 98 |
| Rossellomorea | 0 | 0 | 0 | 0 | 0 |
| Sutcliffiella | 36 | 83 | 65 | 0 | 76 |
| Alkalicella | 49 | 19 | 121 | 0 | 31 |
| Kaustia | 4532 | 3446 | 3659 | 61 | 5401 |
| Natronoglycomyces | 465 | 587 | 1328 | 41 | 1745 |
| Candidatus Formimonas | 0 | 0 | 0 | 0 | 0 |
| Candidatus Pseudothioglobus | 0 | 36 | 17 | 0 | 36 |
| Solibaculum | 112 | 420 | 415 | 1 | 495 |
| Miltoncostaea | 12867 | 13289 | 22524 | 245 | 32452 |
| Breznakiella | 100 | 166 | 211 | 1 | 232 |
| Pacificitalea | 806 | 885 | 960 | 13 | 1173 |
| Thermophilibacter | 580 | 692 | 961 | 6 | 1071 |
| Parafannyhessea | 621 | 1272 | 1308 | 19 | 1237 |
| Aequoribacter | 66 | 119 | 139 | 2 | 172 |
| Atribacter | 56 | 89 | 96 | 9 | 118 |
| Cognaticolwellia | 0 | 0 | 0 | 0 | 0 |
| Peteryoungia | 0 | 0 | 0 | 0 | 0 |
| Falsihalocynthiibacter | 0 | 0 | 0 | 0 | 0 |
| Pseudosulfitobacter | 1769 | 2254 | 2231 | 144 | 2670 |
| Anthocerotibacter | 156 | 441 | 563 | 0 | 839 |
| Mangrovivirga | 34 | 58 | 60 | 0 | 85 |
| Adhaeretor | 1900 | 3389 | 2415 | 46 | 3714 |
| Pseudobdellovibrio | 42 | 56 | 60 | 1 | 69 |
| Carbonactinospora | 1 | 2 | 5 | 0 | 12 |
| Desulfoscipio | 145 | 323 | 265 | 2 | 361 |
| Methyloradius | 31 | 160 | 89 | 5 | 136 |
| Terrihabitans | 2401 | 1711 | 1274 | 10 | 2127 |
| Thermostichus | 67 | 112 | 83 | 14 | 121 |
| Parasynechococcus | 162 | 261 | 337 | 0 | 292 |
| Sandaracinobacteroides | 992 | 1475 | 1394 | 23 | 2297 |
| Citrifermentans | 1393 | 3556 | 3334 | 27 | 3877 |
| Musicola | 154 | 277 | 239 | 9 | 251 |
| Pseudoprevotella | 19 | 47 | 56 | 1 | 102 |
| Gephyromycinifex | 829 | 928 | 1586 | 162 | 2656 |
| Allocoleopsis | 0 | 0 | 0 | 0 | 0 |
| Desulfosediminicola | 81 | 302 | 339 | 6 | 532 |
| Novisyntrophococcus | 36 | 100 | 24 | 0 | 86 |
| Pengzhenrongella | 2341 | 2942 | 4150 | 205 | 5828 |
| Nanchangia | 475 | 571 | 710 | 31 | 1029 |
| Nitrogeniibacter | 2353 | 3328 | 3284 | 49 | 4902 |
| Parazoarcus | 2019 | 2902 | 3363 | 35 | 4812 |
| Pseudazoarcus | 1632 | 2044 | 2194 | 21 | 3204 |
| Pseudothauera | 621 | 1079 | 1057 | 11 | 1808 |
| Vescimonas | 461 | 1131 | 1118 | 14 | 1235 |
| Pusillibacter | 184 | 262 | 348 | 1 | 367 |
| Candidatus Viadribacter | 843 | 1100 | 812 | 14 | 1393 |
| Flagellatimonas | 1070 | 1377 | 1239 | 13 | 1607 |
| Venatorbacter | 60 | 233 | 259 | 9 | 287 |
| Halalkalibacterium (ex Joshi et al. 2022) | 58 | 85 | 64 | 1 | 155 |
| Pseudalkalibacillus | 0 | 0 | 2 | 0 | 2 |
| Alkalihalophilus | 0 | 0 | 0 | 0 | 0 |
| Rhodocaloribacter | 2148 | 5160 | 5037 | 54 | 5567 |
| Aegicerativicinus | 21 | 25 | 23 | 0 | 34 |
| Allosaccharopolyspora | 4431 | 1884 | 5151 | 161 | 5049 |
| Saccharobesus | 26 | 35 | 42 | 6 | 34 |
| Zophobihabitans | 5 | 18 | 20 | 0 | 23 |
| Desulfomarina | 55 | 99 | 112 | 0 | 129 |
| Metamycoplasma | 40 | 95 | 76 | 0 | 50 |
| Candidatus Uabimicrobium | 81 | 339 | 134 | 22 | 282 |
| Paralysiella | 63 | 193 | 167 | 4 | 287 |
| Mergibacter | 10 | 22 | 26 | 1 | 17 |
| Iocasia | 10 | 50 | 46 | 0 | 70 |
| Candidatus Absconditicoccus | 34 | 57 | 53 | 0 | 79 |
| Candidatus Gromoviella | 23 | 24 | 38 | 1 | 49 |
| Stutzerimonas | 12536 | 17174 | 16488 | 236 | 18911 |
| Halopseudomonas | 2286 | 2631 | 2809 | 102 | 3765 |
| Denitrificimonas | 109 | 1445 | 8543 | 15486 | 2632 |
| Mariniplasma | 0 | 29 | 36 | 0 | 48 |
| Alteracholeplasma | 13 | 192 | 30 | 0 | 60 |
| Paracholeplasma | 22 | 39 | 29 | 0 | 59 |
| Desulforapulum | 78 | 227 | 264 | 1 | 465 |
| Desulfosudis | 301 | 872 | 823 | 3 | 1207 |
| Paraneptunicella | 58 | 143 | 60 | 6 | 77 |
| Oceanidesulfovibrio | 786 | 1423 | 1359 | 17 | 2088 |
| Candidatus Minimicrobia | 6 | 41 | 28 | 0 | 49 |
| Constantimarinum | 33 | 136 | 45 | 0 | 59 |
| Tichowtungia | 165 | 436 | 542 | 0 | 645 |
| Oleidesulfovibrio | 281 | 708 | 817 | 4 | 767 |
| Geotalea | 1006 | 2213 | 1684 | 28 | 2452 |
| Megalodesulfovibrio | 517 | 1029 | 823 | 2 | 1045 |
| Solidesulfovibrio | 2067 | 3561 | 3792 | 81 | 4807 |
| Paradesulfovibrio (ex Waite et al. 2020) | 113 | 190 | 181 | 0 | 225 |
| Aquibium | 7789 | 7157 | 7525 | 190 | 9917 |
| Sulfuriroseicoccus | 346 | 1052 | 742 | 29 | 1064 |
| Aggregatimonas | 47 | 55 | 82 | 0 | 90 |
| Mucisphaera | 905 | 2100 | 1903 | 21 | 2366 |
| Spiractinospora | 1303 | 1531 | 3018 | 334 | 6747 |
| Sulfidibacter | 1406 | 3395 | 3296 | 28 | 3332 |
| Desulforamulus | 0 | 0 | 0 | 0 | 0 |
| Leptothermofonsia | 176 | 516 | 365 | 4 | 646 |
| Novacetimonas | 0 | 0 | 0 | 0 | 0 |
| Williamsoniiplasma | 7 | 26 | 16 | 6 | 25 |
| Yangia | 1043 | 1327 | 1484 | 11 | 2040 |
| Natronosporangium | 2586 | 2054 | 5962 | 179 | 6873 |
| Mangrovibacillus | 0 | 0 | 0 | 0 | 0 |
| Phototrophicus | 0 | 0 | 0 | 0 | 0 |
| Candidatus Sulfidibacterium | 77 | 82 | 123 | 0 | 187 |
| Mesomycoplasma | 0 | 0 | 0 | 0 | 0 |
| Acididesulfobacillus | 134 | 313 | 242 | 23 | 522 |
| Candidatus Ichthyocystis | 0 | 0 | 0 | 0 | 0 |
| Periweissella | 11 | 48 | 82 | 0 | 53 |
| Allomeiothermus | 605 | 1291 | 1220 | 16 | 1581 |
| Gymnodinialimonas | 668 | 802 | 814 | 80 | 1003 |
| Tannockella | 10 | 4 | 3 | 4 | 2 |
| Sideroxyarcus | 680 | 1191 | 1036 | 7 | 1896 |
| Pseudemcibacter | 0 | 0 | 0 | 0 | 0 |
| Wujia | 15 | 56 | 72 | 0 | 65 |
| Simiaoa | 0 | 0 | 0 | 0 | 0 |
| Qiania | 98 | 164 | 126 | 7 | 191 |
| Wansuia | 3 | 127 | 99 | 1 | 161 |
| Paenalkalicoccus | 92 | 125 | 94 | 0 | 64 |
| Wielerella | 40 | 157 | 44 | 6 | 73 |
| Pontibrevibacter | 610 | 699 | 593 | 13 | 911 |
| Microvenator | 264 | 797 | 723 | 15 | 835 |
| Neotabrizicola | 984 | 1263 | 1330 | 3 | 1684 |
| Salidesulfovibrio | 347 | 756 | 808 | 14 | 1019 |
| Gracilinema | 18 | 95 | 90 | 0 | 122 |
| Leadbettera | 66 | 180 | 240 | 0 | 231 |
| Alloacidobacterium | 1397 | 4319 | 3296 | 28 | 3864 |
| Psychrodesulfovibrio | 234 | 530 | 528 | 3 | 643 |
| Formicincola | 0 | 0 | 0 | 0 | 0 |
| Candidatus Chromulinivorax | 37 | 100 | 29 | 2 | 16 |
| Candidatus Arcticimaribacter | 0 | 0 | 0 | 0 | 0 |
| Abyssalbus | 26 | 39 | 37 | 2 | 40 |
| Brucepastera | 9 | 75 | 57 | 0 | 58 |
| Marinilongibacter | 44 | 90 | 91 | 0 | 131 |
| Parvicella | 36 | 22 | 18 | 0 | 36 |
| Candidatus Comchoanobacter | 22 | 23 | 26 | 0 | 34 |
| Xianfuyuplasma | 0 | 0 | 0 | 0 | 0 |
| Capillimicrobium | 13590 | 11241 | 29701 | 231 | 32065 |
| Nicolia | 2 | 14 | 26 | 0 | 9 |
| Prescottella | 3497 | 3351 | 5731 | 201 | 6224 |
| Xiamenia | 218 | 707 | 554 | 19 | 777 |
| Horticoccus | 1423 | 3184 | 2529 | 16 | 3261 |
| Rasiella | 16 | 20 | 23 | 4 | 25 |
| Acidilutibacter | 147 | 20 | 77 | 2 | 34 |
| Leptogranulimonas | 120 | 227 | 247 | 2 | 292 |
| Mycoplasmoides | 0 | 0 | 0 | 0 | 0 |
| Actinacidiphila | 3354 | 2567 | 5866 | 367 | 8248 |
| Peterkaempfera | 2599 | 2192 | 4748 | 569 | 11715 |
| Streptantibioticus | 2631 | 2114 | 3933 | 343 | 7877 |
| Anaeropeptidivorans | 11 | 14 | 43 | 8 | 34 |
| Desulfolithobacter | 0 | 0 | 0 | 0 | 0 |
| Alloalcanivorax | 712 | 1107 | 1130 | 100 | 1473 |
| Isoalcanivorax | 539 | 973 | 995 | 16 | 1359 |
| Thermospira | 24 | 115 | 93 | 0 | 139 |
| Solicola | 3314 | 5090 | 5760 | 159 | 5365 |
| Paracrocinitomix | 36 | 29 | 21 | 0 | 29 |
| Anaeromicropila | 21 | 13 | 14 | 0 | 3 |
| Thomasclavelia | 0 | 0 | 0 | 0 | 0 |
| Duffyella | 269 | 344 | 279 | 45 | 517 |
| Aristophania | 10 | 33 | 31 | 0 | 29 |
| Aminithiophilus | 420 | 725 | 1076 | 8 | 1076 |
| Abyssibius | 0 | 0 | 0 | 0 | 0 |
| Deferrimonas | 829 | 1663 | 1686 | 27 | 2275 |
