# Supplementary material for: Multidisciplinary Exploration of Computed‐Tomographic and Ancient‐DNA Data of an Iron Age Skull From Latvia With Multiple Lytic Bone Lesions: Differential Diagnosis Between Metastatic Carcinoma, Multiple Myeloma and Skeletal Tuberculosis
Source: Ecol Evol. 2026 Jul 23;16(7):e74058. doi: 10.1002/ece3.74058 (PMC13395577; doi:10.1002/ece3.74058)
Supplement: Supplementary file 2 — Table S2: Kraken2 metagenomic alignment species level results. [file ECE3-16-e74058-s002.docx]

**Table S2** Kraken2 metagenomic alignment species level results.

| **Sample name** | **P1** | **L1_1** | **L2_1** | **L2_2** | **L2_3** |
| --- | --- | --- | --- | --- | --- |
| **Deduplicated input reads** | **84369632** | **152006844** | **129149404** | **5964256** | **189847984** |
| **Total assigned reads on species level** | **7401962** | **9373577** | **10435950** | **701593** | **19734526** |
| **Total assigned read proportion (%)** | **8.77** | **6.17** | **8.08** | **11.76** | **10.40** |
| **Assigned MTB read proportion (%)** | **0.17** | **0.22** | **0.18** | **0.28** | **0.15** |
| Azorhizobium caulinodans | 3133 | 2059 | 2190 | 0 | 2695 |
| Cellulomonas gilvus | 1954 | 1997 | 2867 | 138 | 3329 |
| Dictyoglomus thermophilum | 0 | 95 | 27 | 0 | 52 |
| Methylophilus methylotrophus | 0 | 0 | 0 | 0 | 0 |
| Syntrophotalea carbinolica | 248 | 499 | 389 | 13 | 428 |
| Shewanella putrefaciens | 92 | 363 | 369 | 19 | 599 |
| Myxococcus xanthus | 3176 | 7770 | 20051 | 167 | 8520 |
| Corallococcus macrosporus | 2719 | 5437 | 3298 | 56 | 7540 |
| Stigmatella aurantiaca | 3104 | 5678 | 5602 | 106 | 7903 |
| Cystobacter fuscus | 3467 | 5971 | 5710 | 102 | 7856 |
| Archangium gephyra | 2928 | 6315 | 5760 | 46 | 6537 |
| Chondromyces crocatus | 3382 | 6111 | 8473 | 77 | 6998 |
| Sorangium cellulosum | 25887 | 61253 | 77153 | 926 | 61330 |
| Vitreoscilla stercoraria | 17 | 59 | 27 | 0 | 33 |
| Vitreoscilla filiformis | 504 | 566 | 597 | 0 | 769 |
| Lysobacter enzymogenes | 5597 | 6532 | 6014 | 126 | 7910 |
| Simonsiella muelleri | 4 | 19 | 13 | 0 | 90 |
| Stella humosa | 6505 | 5323 | 6370 | 307 | 9905 |
| Cyclobacterium marinum | 6 | 54 | 34 | 0 | 43 |
| Runella slithyformis | 197 | 335 | 297 | 5 | 303 |
| Gemmata obscuriglobus | 8967 | 82378 | 32722 | 191 | 42234 |
| Rubinisphaera brasiliensis | 824 | 2978 | 1991 | 16 | 3279 |
| Planctopirus limnophila | 273 | 1407 | 681 | 18 | 1057 |
| Gimesia maris | 420 | 1962 | 1072 | 0 | 1848 |
| Pirellula staleyi | 1505 | 4901 | 4605 | 57 | 7003 |
| Isosphaera pallida | 1063 | 5371 | 3234 | 40 | 5060 |
| Methylocystis parvus | 1688 | 1749 | 1302 | 62 | 2062 |
| Borrelia hermsii | 8 | 12 | 13 | 0 | 10 |
| Borrelia parkeri | 1 | 0 | 11 | 0 | 6 |
| Borrelia anserina | 1 | 8 | 1 | 0 | 4 |
| Borrelia coriaceae | 0 | 6 | 4 | 0 | 3 |
| Spirochaeta thermophila | 346 | 759 | 838 | 7 | 1009 |
| Brachyspira hyodysenteriae | 3 | 24 | 14 | 0 | 7 |
| Treponema phagedenis | 33 | 69 | 67 | 1 | 58 |
| Treponema succinifaciens | 65 | 125 | 59 | 0 | 109 |
| Leptospira biflexa | 12 | 31 | 22 | 0 | 50 |
| Leptospira interrogans | 36 | 114 | 137 | 0 | 120 |
| Leptospira borgpetersenii | 0 | 0 | 0 | 0 | 0 |
| Leptospirillum ferrooxidans | 0 | 0 | 0 | 0 | 0 |
| Azospirillum brasilense | 5112 | 6661 | 6710 | 88 | 8941 |
| Azospirillum lipoferum | 1415 | 1549 | 1543 | 58 | 2163 |
| Campylobacter coli | 54 | 16 | 54 | 0 | 26 |
| Campylobacter fetus | 14 | 27 | 22 | 0 | 54 |
| Campylobacter jejuni | 93 | 107 | 146 | 7 | 83 |
| Campylobacter hyointestinalis | 2 | 8 | 6 | 0 | 15 |
| Campylobacter concisus | 51 | 155 | 126 | 1 | 133 |
| Campylobacter curvus | 23 | 218 | 102 | 0 | 214 |
| Campylobacter lari | 23 | 10 | 11 | 0 | 26 |
| Campylobacter mucosalis | 10 | 18 | 22 | 0 | 21 |
| Campylobacter rectus | 89 | 51 | 55 | 2 | 76 |
| Campylobacter showae | 134 | 94 | 160 | 0 | 157 |
| Campylobacter sputorum | 3 | 8 | 7 | 0 | 15 |
| Helicobacter acinonychis | 1 | 10 | 11 | 0 | 19 |
| Helicobacter cinaedi | 1 | 15 | 10 | 0 | 40 |
| Helicobacter felis | 10 | 9 | 24 | 0 | 22 |
| Helicobacter mustelae | 2 | 27 | 16 | 5 | 63 |
| Pseudoalteromonas carrageenovora | 0 | 0 | 0 | 0 | 0 |
| Brucella abortus | 1600 | 523 | 1703 | 0 | 1359 |
| Brucella ovis | 135 | 19 | 35 | 0 | 42 |
| Elizabethkingia meningoseptica | 0 | 0 | 0 | 0 | 0 |
| Chryseobacterium balustinum | 10 | 122 | 66 | 0 | 91 |
| Empedobacter brevis | 4 | 46 | 35 | 1 | 33 |
| Chryseobacterium gleum | 138 | 134 | 111 | 0 | 143 |
| Chryseobacterium indoltheticum | 0 | 0 | 0 | 0 | 0 |
| Myroides odoratus | 22 | 47 | 32 | 1 | 27 |
| Sphingobacterium thalpophilum | 56 | 233 | 199 | 0 | 268 |
| Francisella tularensis | 163 | 86 | 236 | 13 | 223 |
| Paracoccus denitrificans | 1194 | 1030 | 940 | 18 | 822 |
| Paracoccus sp. (in: a-proteobacteria) | 0 | 4 | 0 | 0 | 1 |
| Meiothermus ruber | 362 | 647 | 752 | 6 | 1063 |
| Xanthobacter autotrophicus | 2271 | 2120 | 2059 | 70 | 2655 |
| Xanthobacter flavus | 0 | 606 | 0 | 0 | 616 |
| Comamonas testosteroni | 1507 | 2802 | 2534 | 0 | 2802 |
| Pseudomonas aeruginosa | 31729 | 62553 | 46489 | 786 | 41481 |
| Pseudoalteromonas atlantica | 0 | 8 | 2 | 0 | 2 |
| Burkholderia cepacia | 0 | 0 | 0 | 0 | 0 |
| Pseudomonas fluorescens | 5458 | 7046 | 6620 | 292 | 13370 |
| Pseudomonas fragi | 0 | 0 | 0 | 0 | 0 |
| Pseudomonas mendocina | 0 | 0 | 0 | 0 | 0 |
| Pseudomonas oleovorans | 0 | 0 | 0 | 0 | 0 |
| Pseudomonas putida | 8249 | 12231 | 13231 | 215 | 19067 |
| Ralstonia solanacearum | 5001 | 18406 | 8391 | 64 | 10619 |
| Pseudomonas sp. | 0 | 69 | 85 | 0 | 13 |
| Stutzerimonas stutzeri | 1372 | 2871 | 1652 | 0 | 0 |
| Pseudomonas syringae | 2423 | 3439 | 3884 | 21 | 16255 |
| Burkholderia glumae | 2061 | 1781 | 2576 | 85 | 9169 |
| Xanthomonas translucens | 4009 | 3485 | 4001 | 53 | 4518 |
| Xanthomonas citri | 5353 | 5571 | 19494 | 0 | 14377 |
| Xanthomonas oryzae | 933 | 1333 | 1364 | 7 | 1189 |
| Azotobacter chroococcum | 1350 | 1835 | 1685 | 26 | 1945 |
| Azotobacter vinelandii | 474 | 717 | 883 | 36 | 1102 |
| Agrobacterium tumefaciens | 8352 | 10292 | 7191 | 162 | 9508 |
| Agrobacterium rhizogenes | 1084 | 1085 | 1106 | 106 | 1327 |
| Agrobacterium vitis | 1956 | 2179 | 2005 | 79 | 3320 |
| Bradyrhizobium japonicum | 11290 | 10828 | 7493 | 20 | 7242 |
| Bradyrhizobium sp. | 2961 | 3725 | 2578 | 24 | 3230 |
| Sinorhizobium fredii | 7873 | 9658 | 8212 | 136 | 10940 |
| Mesorhizobium loti | 3643 | 4197 | 5052 | 3 | 3554 |
| Sinorhizobium meliloti | 5388 | 4614 | 3870 | 45 | 5111 |
| Rhizobium leguminosarum | 16563 | 25647 | 15493 | 326 | 14581 |
| Rhizobium phaseoli | 2104 | 1927 | 1919 | 0 | 1519 |
| Rhizobium tropici | 1579 | 1512 | 1410 | 30 | 1654 |
| Neorhizobium galegae | 3652 | 3637 | 3079 | 84 | 4637 |
| Methylobacillus flagellatus | 0 | 0 | 0 | 0 | 0 |
| Methylorubrum extorquens | 4343 | 3343 | 2578 | 49 | 1206 |
| Methylobacterium sp. | 28 | 27 | 21 | 7 | 30 |
| Methylobacterium organophilum | 2593 | 2888 | 2467 | 27 | 3476 |
| Methylococcus capsulatus | 879 | 1203 | 1373 | 7 | 1286 |
| Methylomonas methanica | 0 | 0 | 0 | 0 | 0 |
| Methylosinus trichosporium | 1990 | 2073 | 1872 | 10 | 2356 |
| Acetobacter aceti | 970 | 1377 | 984 | 1 | 1779 |
| Novacetimonas hansenii | 0 | 0 | 0 | 0 | 0 |
| Acetobacter pasteurianus | 156 | 192 | 2508 | 15 | 216 |
| Gluconobacter oxydans | 674 | 1067 | 882 | 87 | 1410 |
| Legionella pneumophila | 0 | 0 | 0 | 0 | 0 |
| Legionella hackeliae | 56 | 45 | 37 | 7 | 38 |
| Legionella longbeachae | 76 | 51 | 21 | 3 | 28 |
| Tatlockia micdadei | 74 | 44 | 43 | 1 | 52 |
| Legionella spiritensis | 122 | 161 | 130 | 0 | 205 |
| Legionella israelensis | 18 | 107 | 53 | 4 | 154 |
| Legionella jordanis | 25 | 79 | 76 | 0 | 51 |
| Fluoribacter dumoffii | 29 | 37 | 33 | 1 | 21 |
| Acinetobacter calcoaceticus | 0 | 0 | 0 | 0 | 0 |
| Moraxella bovis | 5 | 38 | 62 | 0 | 46 |
| Moraxella nonliquefaciens | 15 | 16 | 56 | 15 | 52 |
| Moraxella catarrhalis | 6 | 29 | 21 | 0 | 27 |
| Neisseria cinerea | 4 | 26 | 20 | 0 | 32 |
| Neisseria flavescens | 10 | 14 | 52 | 0 | 39 |
| Neisseria lactamica | 221 | 302 | 264 | 0 | 279 |
| Neisseria meningitidis | 1043 | 825 | 667 | 13 | 2579 |
| Neisseria animalis | 95 | 96 | 104 | 19 | 141 |
| Neisseria canis | 60 | 72 | 44 | 0 | 114 |
| Neisseria macacae | 0 | 0 | 0 | 0 | 0 |
| Thermomicrobium roseum | 692 | 1178 | 1466 | 18 | 1705 |
| Bordetella bronchiseptica | 2273 | 1771 | 1940 | 57 | 3009 |
| Bordetella parapertussis | 882 | 442 | 15561 | 0 | 456 |
| Bordetella pertussis | 1387 | 22316 | 4804 | 0 | 74206 |
| Bordetella avium | 0 | 0 | 0 | 0 | 0 |
| Acidiphilium cryptum | 1040 | 1439 | 1702 | 6 | 2159 |
| Brucella anthropi | 0 | 0 | 0 | 0 | 0 |
| Beijerinckia indica | 0 | 0 | 0 | 0 | 0 |
| Chromobacterium violaceum | 902 | 1379 | 1350 | 18 | 1977 |
| Iodobacter fluviatilis | 70 | 353 | 177 | 2 | 424 |
| Eikenella corrodens | 156 | 339 | 352 | 0 | 546 |
| Zymomonas mobilis | 81 | 169 | 962 | 43 | 299 |
| Citrobacter koseri | 159 | 224 | 264 | 0 | 251 |
| Citrobacter freundii | 1452 | 3973 | 2043 | 217 | 2077 |
| Pantoea agglomerans | 305 | 7375 | 942 | 0 | 818 |
| Enterobacter cloacae | 2538 | 3204 | 3756 | 0 | 3527 |
| Pantoea ananatis | 264 | 430 | 369 | 21 | 420 |
| Pectobacterium carotovorum | 0 | 0 | 0 | 0 | 0 |
| Dickeya chrysanthemi | 193 | 275 | 289 | 3 | 299 |
| Shimwellia blattae | 0 | 0 | 0 | 0 | 0 |
| Escherichia fergusonii | 0 | 0 | 0 | 0 | 0 |
| Atlantibacter hermannii | 0 | 0 | 0 | 0 | 0 |
| Pseudescherichia vulneris | 160 | 193 | 286 | 2 | 466 |
| Hafnia alvei | 0 | 0 | 0 | 0 | 0 |
| Klebsiella oxytoca | 620 | 631 | 870 | 5 | 893 |
| Klebsiella pneumoniae | 19691 | 25092 | 19117 | 1721 | 12980 |
| Raoultella planticola | 412 | 643 | 336 | 3 | 395 |
| Raoultella terrigena | 0 | 0 | 0 | 0 | 0 |
| Morganella morganii | 371 | 887 | 748 | 18 | 1103 |
| Proteus mirabilis | 0 | 0 | 0 | 0 | 0 |
| Providencia rettgeri | 0 | 0 | 0 | 0 | 0 |
| Providencia stuartii | 28 | 80 | 195 | 0 | 232 |
| Salmonella sp. | 0 | 322 | 0 | 0 | 58 |
| Serratia odorifera | 248 | 378 | 290 | 8 | 327 |
| Shigella boydii | 0 | 0 | 0 | 0 | 0 |
| Shigella flexneri | 593 | 1744 | 2928 | 1 | 680 |
| Shigella sonnei | 51 | 369 | 238 | 0 | 105 |
| Xenorhabdus nematophila | 0 | 0 | 0 | 0 | 0 |
| Yersinia enterocolitica | 185 | 188 | 506 | 15 | 157 |
| Yersinia intermedia | 13 | 207 | 47 | 4 | 89 |
| Yersinia pestis | 5802 | 2960 | 3103 | 0 | 2300 |
| Yersinia bercovieri | 11 | 33 | 13 | 0 | 20 |
| Edwardsiella tarda | 0 | 0 | 0 | 0 | 0 |
| Arsenophonus nasoniae | 2 | 17 | 22 | 3 | 25 |
| Aeromonas hydrophila | 2593 | 3320 | 3339 | 123 | 3579 |
| Aeromonas salmonicida | 0 | 0 | 0 | 0 | 0 |
| Aeromonas sobria | 24 | 157 | 0 | 0 | 0 |
| Aeromonas caviae | 1795 | 2414 | 2700 | 0 | 1413 |
| Aeromonas jandaei | 398 | 452 | 478 | 2 | 559 |
| Aeromonas media | 1234 | 1298 | 1530 | 25 | 766 |
| Aeromonas schubertii | 402 | 671 | 728 | 21 | 732 |
| Aeromonas veronii | 796 | 934 | 827 | 0 | 0 |
| Aeromonas allosaccharophila | 0 | 0 | 0 | 0 | 0 |
| Vibrio alginolyticus | 25 | 222 | 234 | 0 | 190 |
| Aliivibrio fischeri | 40 | 31 | 40 | 26 | 107 |
| Vibrio harveyi | 69 | 370 | 139 | 32 | 173 |
| Vibrio parahaemolyticus | 1338 | 304 | 1500 | 3 | 64 |
| Vibrio vulnificus | 0 | 0 | 0 | 0 | 0 |
| Grimontia hollisae | 102 | 116 | 149 | 0 | 92 |
| Vibrio mimicus | 42 | 126 | 162 | 23 | 141 |
| Vibrio cincinnatiensis | 0 | 0 | 0 | 0 | 0 |
| Vibrio fluvialis | 183 | 310 | 221 | 0 | 437 |
| Vibrio campbellii | 82 | 128 | 99 | 0 | 197 |
| Vibrio gazogenes | 0 | 0 | 0 | 0 | 0 |
| Vibrio mediterranei | 14 | 33 | 15 | 0 | 30 |
| Vibrio natriegens | 62 | 102 | 77 | 0 | 125 |
| Plesiomonas shigelloides | 238 | 304 | 335 | 2 | 284 |
| Aggregatibacter actinomycetemcomitans | 39 | 131 | 89 | 6 | 211 |
| Actinobacillus pleuropneumoniae | 13 | 92 | 21 | 8 | 35 |
| Actinobacillus suis | 24 | 13 | 25 | 0 | 31 |
| Actinobacillus equuli | 1 | 16 | 15 | 0 | 15 |
| Actinobacillus lignieresii | 2 | 5 | 11 | 0 | 8 |
| Haemophilus influenzae | 54 | 71 | 63 | 1 | 54 |
| Avibacterium paragallinarum | 4 | 64 | 77 | 0 | 59 |
| [Haemophilus] ducreyi | 0 | 17 | 23 | 0 | 18 |
| Histophilus somni | 18 | 24 | 30 | 0 | 9 |
| Glaesserella parasuis | 62 | 39 | 61 | 1 | 101 |
| Aggregatibacter segnis | 0 | 0 | 0 | 0 | 0 |
| Pasteurella multocida | 961 | 1790 | 2431 | 25 | 507 |
| Gallibacterium anatis | 0 | 0 | 0 | 0 | 0 |
| Pasteurella canis | 32 | 17 | 12 | 0 | 29 |
| Pasteurella dagmatis | 13 | 7 | 9 | 0 | 9 |
| Avibacterium volantium | 0 | 0 | 0 | 0 | 0 |
| Anaplasma centrale | 11 | 21 | 22 | 0 | 36 |
| Anaplasma marginale | 12 | 24 | 37 | 0 | 10 |
| Bartonella bacilliformis | 9 | 17 | 26 | 0 | 48 |
| Coxiella burnetii | 87 | 415 | 285 | 1 | 293 |
| Ehrlichia ruminantium | 0 | 0 | 0 | 0 | 0 |
| Rickettsia conorii | 0 | 0 | 0 | 0 | 0 |
| Rickettsia prowazekii | 0 | 1 | 2 | 11 | 2 |
| Rickettsia rickettsii | 0 | 0 | 47 | 0 | 62 |
| Orientia tsutsugamushi | 21 | 21 | 20 | 0 | 23 |
| Rickettsia typhi | 3 | 5 | 4 | 0 | 8 |
| Rickettsia akari | 0 | 1 | 7 | 0 | 7 |
| Rickettsia australis | 0 | 5 | 4 | 0 | 5 |
| Rickettsia canadensis | 0 | 16 | 7 | 0 | 4 |
| Bartonella quintana | 5 | 98 | 36 | 0 | 42 |
| Bartonella elizabethae | 12 | 25 | 30 | 0 | 18 |
| Chlamydia trachomatis | 1 | 48 | 16 | 0 | 16 |
| Bacteroides fragilis | 0 | 0 | 0 | 0 | 0 |
| Bacteroides thetaiotaomicron | 53 | 212 | 156 | 4 | 194 |
| Bacteroides uniformis | 402 | 160 | 150 | 0 | 193 |
| Phocaeicola vulgatus | 34 | 67 | 53 | 5 | 33 |
| Parabacteroides distasonis | 0 | 0 | 0 | 0 | 0 |
| Campylobacter gracilis | 89 | 105 | 102 | 1 | 131 |
| Sebaldella termitidis | 53 | 24 | 45 | 0 | 39 |
| Butyrivibrio fibrisolvens | 17 | 86 | 43 | 0 | 65 |
| Fibrobacter succinogenes | 0 | 0 | 0 | 0 | 0 |
| Prevotella ruminicola | 0 | 0 | 0 | 0 | 0 |
| Wolinella succinogenes | 28 | 69 | 81 | 1 | 97 |
| Oxalobacter formigenes | 39 | 246 | 226 | 15 | 317 |
| Fusobacterium gonidiaformans | 0 | 0 | 0 | 0 | 0 |
| Fusobacterium mortiferum | 9 | 4 | 6 | 0 | 7 |
| Faecalibacterium prausnitzii | 786 | 1946 | 2070 | 45 | 2578 |
| Fusobacterium varium | 0 | 0 | 0 | 0 | 0 |
| Fusobacterium necrophorum | 1 | 9 | 5 | 0 | 15 |
| Fusobacterium periodonticum | 2 | 0 | 0 | 0 | 1 |
| Fusobacterium ulcerans | 4 | 18 | 7 | 0 | 10 |
| Syntrophomonas wolfei | 20 | 80 | 153 | 0 | 184 |
| Pectinatus frisingensis | 0 | 0 | 0 | 0 | 0 |
| Dichelobacter nodosus | 42 | 76 | 62 | 0 | 58 |
| Desulfocurvibacter africanus | 668 | 1098 | 1362 | 7 | 1488 |
| Desulfovibrio desulfuricans | 446 | 568 | 626 | 21 | 1111 |
| Megalodesulfovibrio gigas | 483 | 991 | 779 | 0 | 946 |
| Maridesulfovibrio salexigens | 172 | 85 | 120 | 1 | 134 |
| Desulfovibrio vulgaris | 1048 | 1911 | 2048 | 2 | 1827 |
| Desulfobulbus propionicus | 429 | 817 | 1091 | 27 | 1374 |
| Desulfococcus multivorans | 490 | 1023 | 962 | 58 | 1496 |
| Desulfomicrobium baculatum | 0 | 0 | 0 | 0 | 0 |
| Desulfovibrio piger | 325 | 688 | 783 | 31 | 1270 |
| Acidaminococcus fermentans | 0 | 0 | 0 | 0 | 0 |
| Megasphaera elsdenii | 321 | 819 | 634 | 30 | 924 |
| Nitrobacter hamburgensis | 5034 | 2348 | 1740 | 181 | 2191 |
| Nitrobacter winogradskyi | 0 | 0 | 0 | 0 | 0 |
| Nitrosomonas europaea | 142 | 587 | 312 | 0 | 447 |
| Nitrosomonas eutropha | 295 | 748 | 406 | 20 | 773 |
| Acidithiobacillus ferrooxidans | 592 | 823 | 661 | 1 | 780 |
| Starkeya novella | 4632 | 3024 | 2981 | 38 | 3870 |
| Thiobacillus sp. | 0 | 1 | 0 | 0 | 0 |
| Thiomonas intermedia | 601 | 941 | 884 | 11 | 999 |
| Halothiobacillus neapolitanus | 0 | 0 | 0 | 0 | 0 |
| Acidithiobacillus thiooxidans | 140 | 153 | 182 | 0 | 184 |
| Hydrogenobacter thermophilus | 0 | 0 | 0 | 0 | 0 |
| Ehrlichia canis | 2 | 254 | 12 | 0 | 44 |
| Ehrlichia chaffeensis | 4 | 5 | 2 | 0 | 4 |
| Anaplasma phagocytophilum | 5 | 16 | 16 | 8 | 18 |
| Anaplasma platys | 4 | 25 | 29 | 0 | 36 |
| Neorickettsia risticii | 0 | 20 | 13 | 0 | 8 |
| Neorickettsia sennetsu | 3 | 44 | 5 | 0 | 25 |
| Francisella persica | 4 | 6 | 4 | 0 | 7 |
| Wolbachia pipientis | 32 | 10 | 11 | 0 | 1 |
| Bdellovibrio bacteriovorus | 0 | 0 | 0 | 0 | 0 |
| Bacteriovorax stolpii | 35 | 54 | 70 | 2 | 106 |
| Herbaspirillum seropedicae | 901 | 1546 | 1617 | 9 | 2322 |
| Selenomonas ruminantium | 87 | 274 | 135 | 2 | 381 |
| Cellulophaga lytica | 31 | 21 | 39 | 0 | 37 |
| Pedobacter heparinus | 0 | 0 | 0 | 0 | 0 |
| Cytophaga hutchinsonii | 31 | 183 | 139 | 0 | 173 |
| Flavobacterium johnsoniae | 147 | 320 | 300 | 0 | 297 |
| Solitalea canadensis | 74 | 118 | 113 | 0 | 103 |
| Bernardetia litoralis | 48 | 36 | 67 | 0 | 52 |
| Marivirga tractuosa | 50 | 322 | 81 | 0 | 155 |
| Saprospira grandis | 0 | 0 | 0 | 0 | 0 |
| Sphingobacterium mizutaii | 0 | 0 | 0 | 0 | 0 |
| Weeksella virosa | 0 | 0 | 0 | 0 | 0 |
| Capnocytophaga ochracea | 24 | 110 | 84 | 0 | 72 |
| Allochromatium vinosum | 1055 | 1524 | 1357 | 7 | 1543 |
| Thermochromatium tepidum | 650 | 1034 | 1036 | 13 | 1257 |
| Halorhodospira halochloris | 204 | 374 | 316 | 2 | 338 |
| Halorhodospira halophila | 861 | 1706 | 1469 | 13 | 1485 |
| Rhodobacter capsulatus | 717 | 1337 | 1064 | 10 | 1214 |
| Cereibacter sphaeroides | 2765 | 3386 | 3773 | 67 | 4342 |
| Rhodomicrobium vannielii | 2463 | 1684 | 1711 | 17 | 1901 |
| Fuscovulum blasticum | 0 | 0 | 0 | 0 | 0 |
| Rhodopseudomonas palustris | 21493 | 14273 | 12741 | 182 | 17301 |
| Blastochloris viridis | 3859 | 2519 | 2512 | 58 | 3467 |
| Pararhodospirillum photometricum | 599 | 1010 | 1104 | 16 | 1797 |
| Rhodospirillum rubrum | 1292 | 1426 | 1307 | 74 | 2278 |
| Chlorobium limicola | 105 | 302 | 267 | 4 | 349 |
| Chlorobium phaeovibrioides | 92 | 194 | 113 | 1 | 173 |
| Chlorobium phaeobacteroides | 0 | 0 | 0 | 0 | 0 |
| Chlorobaculum tepidum | 247 | 617 | 695 | 0 | 1137 |
| Pelodictyon luteolum | 232 | 672 | 548 | 4 | 656 |
| Prosthecochloris aestuarii | 34 | 250 | 319 | 1 | 311 |
| Chloroflexus aurantiacus | 283 | 1084 | 630 | 21 | 1144 |
| Microcystis aeruginosa | 197 | 1214 | 731 | 24 | 967 |
| Synechocystis sp. PCC 6714 | 15 | 80 | 87 | 0 | 107 |
| Planktothrix agardhii | 0 | 0 | 0 | 0 | 0 |
| Trichormus azollae | 30 | 184 | 92 | 0 | 88 |
| Anabaena cylindrica | 33 | 165 | 104 | 0 | 148 |
| Dolichospermum flos-aquae | 48 | 239 | 132 | 41 | 414 |
| Nostoc commune | 38 | 79 | 86 | 0 | 96 |
| Leptolyngbya boryana | 50 | 270 | 200 | 1 | 259 |
| Tolypothrix sp. PCC 7601 | 0 | 289 | 244 | 0 | 397 |
| Nitrosococcus oceani | 61 | 250 | 187 | 1 | 317 |
| Nitrosospira multiformis | 313 | 733 | 498 | 22 | 919 |
| Piscirickettsia salmonis | 56 | 74 | 63 | 40 | 99 |
| Leuconostoc gelidum | 0 | 0 | 0 | 0 | 0 |
| Leuconostoc mesenteroides | 6 | 23 | 30 | 16 | 10 |
| Leuconostoc lactis | 0 | 20 | 31 | 0 | 36 |
| Oenococcus oeni | 7 | 10 | 14 | 0 | 25 |
| Weissella paramesenteroides | 9 | 12 | 11 | 0 | 14 |
| Leuconostoc carnosum | 0 | 4 | 5 | 0 | 12 |
| Pediococcus acidilactici | 29 | 106 | 48 | 2 | 53 |
| Pediococcus pentosaceus | 0 | 0 | 0 | 0 | 0 |
| Peptoniphilus asaccharolyticus | 3 | 0 | 0 | 0 | 0 |
| Ruminococcus albus | 9 | 34 | 63 | 1 | 84 |
| Kocuria varians | 518 | 650 | 855 | 38 | 989 |
| Staphylococcus aureus | 243 | 8832 | 0 | 101 | 0 |
| Staphylococcus hyicus | 3 | 9 | 8 | 0 | 15 |
| Staphylococcus warneri | 0 | 0 | 0 | 0 | 0 |
| Staphylococcus gallinarum | 0 | 0 | 0 | 0 | 0 |
| Staphylococcus muscae | 0 | 0 | 0 | 0 | 0 |
| Staphylococcus schleiferi | 0 | 0 | 0 | 0 | 0 |
| Deinococcus radiodurans | 591 | 950 | 1084 | 0 | 1127 |
| Streptococcus suis | 148 | 180 | 317 | 3 | 190 |
| Streptococcus mutans | 0 | 0 | 0 | 0 | 0 |
| Streptococcus sobrinus | 0 | 0 | 0 | 0 | 0 |
| Streptococcus pyogenes | 963 | 34 | 56 | 0 | 409 |
| Blautia hansenii | 0 | 0 | 0 | 0 | 0 |
| Streptococcus acidominimus | 0 | 3 | 1 | 0 | 9 |
| Streptococcus anginosus | 0 | 0 | 0 | 0 | 0 |
| Streptococcus canis | 0 | 19 | 17 | 0 | 15 |
| Streptococcus dysgalactiae | 19 | 33 | 6 | 0 | 4 |
| Streptococcus equinus | 0 | 0 | 0 | 0 | 0 |
| Streptococcus equi | 33 | 33 | 99 | 0 | 72 |
| Streptococcus intermedius | 70 | 15 | 29 | 0 | 5 |
| Streptococcus porcinus | 1 | 17 | 4 | 0 | 10 |
| Streptococcus ratti | 12 | 21 | 44 | 0 | 18 |
| Streptococcus vestibularis | 0 | 0 | 0 | 0 | 0 |
| Streptococcus ferus | 0 | 0 | 0 | 0 | 0 |
| Streptococcus iniae | 0 | 5 | 8 | 2 | 5 |
| Streptococcus parauberis | 17 | 5 | 16 | 0 | 9 |
| Streptococcus uberis | 0 | 6 | 12 | 0 | 10 |
| Enterococcus faecium | 2562 | 130 | 183 | 0 | 0 |
| Enterococcus gallinarum | 0 | 0 | 0 | 0 | 0 |
| Enterococcus hirae | 12 | 155 | 110 | 6 | 58 |
| Lactococcus lactis | 0 | 0 | 0 | 0 | 0 |
| Lactococcus garvieae | 39 | 168 | 123 | 3 | 50 |
| Lactococcus piscium | 0 | 7 | 4 | 0 | 53 |
| Lactococcus raffinolactis | 0 | 0 | 0 | 0 | 0 |
| Planococcus kocurii | 2 | 16 | 38 | 0 | 38 |
| Aerococcus urinae | 19 | 25 | 38 | 0 | 33 |
| Bacillus amyloliquefaciens | 2573 | 1138 | 4935 | 119 | 3652 |
| Bacillus anthracis | 18581 | 3916 | 9081 | 43 | 10928 |
| Brevibacillus brevis | 220 | 567 | 473 | 2 | 626 |
| [Bacillus] caldolyticus | 59 | 36 | 171 | 0 | 164 |
| Bacillus cereus | 20455 | 19108 | 12011 | 32 | 11264 |
| Weizmannia coagulans | 0 | 0 | 0 | 0 | 0 |
| Cytobacillus firmus | 9 | 51 | 64 | 0 | 163 |
| Paenibacillus lautus | 136 | 336 | 246 | 1 | 644 |
| Bacillus licheniformis | 117 | 703 | 371 | 0 | 293 |
| Priestia megaterium | 0 | 0 | 0 | 0 | 0 |
| Bacillus mycoides | 1331 | 803 | 204 | 69 | 176 |
| Paenibacillus polymyxa | 455 | 1089 | 1381 | 91 | 2160 |
| Peribacillus psychrosaccharolyticus | 9 | 98 | 57 | 10 | 78 |
| Bacillus pumilus | 0 | 0 | 0 | 0 | 0 |
| Evansella cellulosilytica | 36 | 15 | 17 | 0 | 28 |
| Lysinibacillus sphaericus | 12 | 19 | 40 | 0 | 35 |
| Bacillus subtilis | 1977 | 4793 | 6747 | 512 | 6552 |
| Bacillus thuringiensis | 0 | 0 | 0 | 0 | 0 |
| Amphibacillus xylanus | 3 | 4 | 16 | 0 | 6 |
| Alicyclobacillus acidoterrestris | 0 | 0 | 0 | 0 | 0 |
| Bacillus atrophaeus | 12 | 1710 | 33 | 0 | 132 |
| Bacillus badius | 47 | 116 | 79 | 1 | 126 |
| Alicyclobacillus cycloheptanicus | 303 | 796 | 616 | 12 | 737 |
| Geobacillus kaustophilus | 23 | 164 | 46 | 0 | 169 |
| Paenibacillus larvae | 60457 | 62790 | 154413 | 0 | 83076 |
| Brevibacillus laterosporus | 115 | 579 | 192 | 24 | 137 |
| Lederbergia lenta | 11 | 19 | 15 | 0 | 10 |
| Bacillus methanolicus | 0 | 0 | 0 | 0 | 0 |
| Paenibacillus pabuli | 0 | 0 | 0 | 0 | 0 |
| Virgibacillus pantothenticus | 0 | 0 | 0 | 0 | 30 |
| Sporosarcina pasteurii | 4 | 8 | 27 | 3 | 28 |
| Sporosarcina psychrophila | 0 | 172 | 75 | 0 | 48 |
| Peribacillus simplex | 755 | 4697 | 1502 | 17 | 1918 |
| Bacillus smithii | 29 | 153 | 121 | 0 | 95 |
| Virgibacillus halodenitrificans | 0 | 0 | 0 | 0 | 0 |
| Clostridium acetobutylicum | 11 | 12 | 16 | 0 | 8 |
| Paraclostridium bifermentans | 34 | 24 | 9 | 0 | 16 |
| Clostridium botulinum | 0 | 0 | 0 | 0 | 0 |
| Clostridium butyricum | 0 | 0 | 0 | 0 | 0 |
| Clostridium cellulovorans | 16 | 28 | 59 | 0 | 43 |
| Clostridium cochlearium | 178 | 14 | 27 | 3 | 94 |
| Clostridioides difficile | 976 | 156 | 1591 | 4 | 1960 |
| Clostridium formicaceticum | 31 | 16 | 40 | 1 | 40 |
| Hathewaya histolytica | 54 | 17 | 18 | 1 | 19 |
| Clostridium septicum | 18 | 4 | 11 | 0 | 5 |
| Clostridium sporogenes | 126 | 27 | 85 | 12 | 45 |
| Thermoclostridium stercorarium | 59 | 108 | 75 | 1 | 125 |
| Acetoanaerobium sticklandii | 13 | 18 | 24 | 0 | 12 |
| Acetivibrio thermocellus | 7 | 482 | 45 | 2 | 67 |
| Thermoanaerobacterium thermosaccharolyticum | 30 | 75 | 110 | 0 | 63 |
| Clostridium tyrobutyricum | 0 | 0 | 0 | 0 | 0 |
| Clostridium beijerinckii | 0 | 0 | 0 | 0 | 0 |
| Ruminiclostridium cellulolyticum | 17 | 93 | 18 | 0 | 46 |
| [Clostridium] innocuum | 0 | 0 | 0 | 0 | 0 |
| Moorella thermoacetica | 0 | 0 | 0 | 0 | 0 |
| Clostridium cadaveris | 29 | 31 | 16 | 3 | 12 |
| Enterocloster clostridioformis | 0 | 0 | 0 | 0 | 0 |
| Clostridium kluyveri | 23 | 32 | 34 | 0 | 66 |
| Clostridium ljungdahlii | 0 | 0 | 0 | 0 | 2 |
| Clostridium novyi | 48 | 6 | 7 | 1 | 4 |
| Thomasclavelia ramosa | 0 | 0 | 0 | 0 | 0 |
| Clostridium scatologenes | 25 | 24 | 12 | 0 | 15 |
| Gottschalkia acidurici | 122 | 46 | 58 | 21 | 44 |
| Clostridium baratii | 22 | 18 | 10 | 2 | 9 |
| Desulfosporosinus orientis | 0 | 0 | 0 | 0 | 0 |
| Desulforamulus ruminis | 0 | 0 | 0 | 0 | 0 |
| Desulfotomaculum nigrificans | 90 | 104 | 207 | 1 | 318 |
| Halobacillus halophilus | 0 | 0 | 0 | 0 | 0 |
| Sporosarcina ureae | 0 | 0 | 0 | 0 | 0 |
| Leifsonia xyli | 968 | 1357 | 1644 | 70 | 2358 |
| Lactobacillus acidophilus | 102 | 137 | 575 | 0 | 421 |
| Levilactobacillus brevis | 8 | 119 | 76 | 0 | 178 |
| Lentilactobacillus buchneri | 15 | 41 | 49 | 0 | 58 |
| Lacticaseibacillus casei | 29 | 93 | 57 | 4 | 86 |
| Lentilactobacillus hilgardii | 1 | 43 | 10 | 4 | 22 |
| Lactiplantibacillus pentosus | 93 | 77 | 126 | 0 | 117 |
| Lactobacillus sp. | 0 | 0 | 0 | 0 | 0 |
| Lactobacillus gasseri | 0 | 21 | 33 | 0 | 22 |
| Lacticaseibacillus paracasei | 122 | 234 | 186 | 19 | 242 |
| Latilactobacillus sakei | 0 | 0 | 0 | 0 | 0 |
| Lactobacillus acetotolerans | 4 | 10 | 14 | 0 | 18 |
| Ligilactobacillus agilis | 56 | 65 | 51 | 0 | 52 |
| Companilactobacillus alimentarius | 9 | 7 | 5 | 0 | 4 |
| Amylolactobacillus amylophilus | 27 | 35 | 26 | 0 | 18 |
| Lactobacillus amylovorus | 47 | 57 | 60 | 0 | 168 |
| Ligilactobacillus animalis | 0 | 0 | 0 | 0 | 0 |
| Loigolactobacillus bifermentans | 3 | 11 | 15 | 0 | 19 |
| Loigolactobacillus coryniformis | 0 | 67 | 39 | 0 | 120 |
| Companilactobacillus farciminis | 10 | 10 | 20 | 0 | 17 |
| Limosilactobacillus fermentum | 0 | 0 | 0 | 0 | 0 |
| Fructilactobacillus fructivorans | 1 | 9 | 33 | 0 | 7 |
| Liquorilactobacillus mali | 11 | 23 | 26 | 0 | 27 |
| Ligilactobacillus murinus | 0 | 0 | 0 | 0 | 0 |
| Ligilactobacillus ruminis | 93 | 100 | 131 | 0 | 123 |
| Ligilactobacillus salivarius | 0 | 0 | 0 | 0 | 0 |
| Fructilactobacillus sanfranciscensis | 0 | 0 | 0 | 0 | 0 |
| Weissella viridescens | 0 | 0 | 0 | 0 | 0 |
| Limosilactobacillus oris | 44 | 95 | 91 | 0 | 90 |
| Limosilactobacillus vaginalis | 7 | 16 | 32 | 0 | 39 |
| Listeria ivanovii | 1 | 16 | 22 | 10 | 263 |
| Listeria monocytogenes | 7282 | 3467 | 2001 | 631 | 5754 |
| Listeria seeligeri | 1 | 18 | 5 | 0 | 11 |
| Listeria grayi | 18 | 33 | 25 | 0 | 53 |
| Listeria welshimeri | 0 | 10 | 10 | 0 | 5 |
| Renibacterium salmoninarum | 182 | 249 | 298 | 11 | 348 |
| Erysipelothrix rhusiopathiae | 0 | 11 | 18 | 25 | 24 |
| Schaalia odontolytica | 536 | 611 | 1132 | 34 | 889 |
| Trueperella pyogenes | 481 | 795 | 1167 | 45 | 885 |
| Arthrobacter sp. | 47 | 60 | 89 | 0 | 99 |
| Bifidobacterium bifidum | 290 | 743 | 635 | 28 | 1072 |
| Bifidobacterium angulatum | 245 | 398 | 387 | 31 | 669 |
| Bifidobacterium asteroides | 0 | 0 | 0 | 0 | 0 |
| Bifidobacterium breve | 0 | 0 | 0 | 0 | 0 |
| Bifidobacterium catenulatum | 122 | 215 | 284 | 3 | 420 |
| Bifidobacterium coryneforme | 4 | 45 | 65 | 0 | 38 |
| Bifidobacterium dentium | 223 | 285 | 311 | 2 | 340 |
| Bifidobacterium indicum | 88 | 46 | 83 | 6 | 62 |
| Bifidobacterium pseudolongum | 571 | 1043 | 1135 | 20 | 1375 |
| Brevibacterium linens | 818 | 1123 | 1534 | 37 | 2075 |
| Cellulomonas fimi | 4092 | 5120 | 7221 | 83 | 8008 |
| Cellulosimicrobium cellulans | 5802 | 6803 | 9930 | 408 | 11530 |
| Cellulomonas flavigena | 1636 | 1795 | 2665 | 57 | 3286 |
| Corynebacterium diphtheriae | 2091 | 424 | 3046 | 1436 | 3534 |
| Corynebacterium glutamicum | 1594 | 1285 | 1583 | 16 | 2684 |
| Corynebacterium pseudotuberculosis | 0 | 0 | 0 | 0 | 0 |
| Corynebacterium callunae | 0 | 0 | 0 | 0 | 0 |
| Corynebacterium renale | 198 | 294 | 267 | 17 | 391 |
| Corynebacterium xerosis | 0 | 0 | 0 | 0 | 0 |
| Corynebacterium variabile | 758 | 707 | 1049 | 24 | 893 |
| Peptoclostridium acidaminophilum | 38 | 76 | 84 | 0 | 99 |
| Eubacterium limosum | 50 | 206 | 222 | 4 | 301 |
| Thermodesulfobacterium commune | 23 | 34 | 42 | 0 | 57 |
| Propionibacterium freudenreichii | 1450 | 1744 | 3154 | 80 | 3260 |
| Acidipropionibacterium acidipropionici | 1161 | 1590 | 2774 | 125 | 4583 |
| Acidipropionibacterium jensenii | 1458 | 2419 | 3028 | 208 | 5032 |
| Arachnia propionica | 590 | 947 | 1471 | 37 | 2010 |
| Mycobacterium avium | 5110 | 8874 | 6897 | 112 | 13547 |
| Mycolicibacterium fortuitum | 2714 | 7851 | 5253 | 71 | 12470 |
| Mycobacterium intracellulare | 9823 | 11231 | 10329 | 118 | 16576 |
| Mycobacterium kansasii | 1596 | 44346 | 8914 | 29 | 34123 |
| Mycolicibacterium phlei | 3060 | 10571 | 6959 | 117 | 16860 |
| Mycolicibacterium smegmatis | 3974 | 14425 | 9011 | 103 | 19873 |
| Mycobacterium tuberculosis | 12599 | 20981 | 18640 | 1968 | 28583 |
| Mycobacterium cookii | 1723 | 4527 | 3229 | 22 | 6993 |
| Mycobacterium gordonae | 1628 | 2942 | 2238 | 65 | 4638 |
| Mycobacterium malmoense | 1902 | 3175 | 2356 | 72 | 4110 |
| Mycobacterium marinum | 1155 | 3084 | 2344 | 63 | 4234 |
| Mycobacterium simiae | 1239 | 2443 | 1867 | 0 | 3477 |
| Mycolicibacter terrae | 1695 | 2848 | 2252 | 72 | 3829 |
| Mycobacterium xenopi | 1305 | 4016 | 2598 | 24 | 5300 |
| Mycolicibacterium aurum | 2589 | 7302 | 4912 | 37 | 12149 |
| Mycolicibacterium chitae | 2181 | 5221 | 4206 | 110 | 8102 |
| Mycolicibacterium fallax | 1981 | 3502 | 3155 | 123 | 6247 |
| Mycolicibacterium gadium | 3073 | 13165 | 7902 | 83 | 22886 |
| Mycolicibacterium neoaurum | 2860 | 7345 | 5405 | 88 | 11595 |
| Mycolicibacterium senegalense | 1089 | 2728 | 2107 | 5 | 4451 |
| Mycolicibacterium thermoresistibile | 2580 | 6596 | 4545 | 39 | 9697 |
| Mycolicibacterium aichiense | 1971 | 4243 | 3174 | 56 | 6956 |
| Mycolicibacterium chubuense | 5176 | 14442 | 10993 | 47 | 20840 |
| Mycobacterium diernhoferi | 1875 | 4065 | 3076 | 36 | 6596 |
| Mycolicibacterium farcinogenes | 1953 | 4818 | 4101 | 151 | 8930 |
| Mycolicibacterium gilvum | 2633 | 7086 | 4821 | 154 | 11491 |
| Mycobacterium ulcerans | 276 | 703 | 458 | 62 | 1262 |
| Mycolicibacterium vaccae | 2447 | 6738 | 4831 | 105 | 11081 |
| Amycolatopsis methanolica | 11811 | 3634 | 12520 | 146 | 8556 |
| Nocardia otitidiscaviarum | 2225 | 2048 | 3536 | 165 | 4325 |
| Nocardia asteroides | 12472 | 10705 | 20311 | 847 | 25981 |
| Rhodococcus fascians | 1877 | 2833 | 3964 | 132 | 5602 |
| Rhodococcus rhodochrous | 2261 | 2164 | 3061 | 83 | 3987 |
| Rhodococcus ruber | 2140 | 2629 | 3909 | 221 | 5361 |
| Rhodococcus erythropolis | 1298 | 2095 | 2619 | 0 | 2637 |
| Saccharopolyspora erythraea | 16295 | 6842 | 16979 | 779 | 21047 |
| Saccharomonospora viridis | 3096 | 1061 | 3393 | 107 | 3655 |
| Frankia sp. ArI3 | 1938 | 2426 | 3382 | 209 | 5993 |
| Frankia alni | 2569 | 2396 | 4597 | 232 | 6692 |
| Geodermatophilus obscurus | 3332 | 4346 | 5993 | 156 | 7563 |
| Dermatophilus congolensis | 307 | 231 | 469 | 7 | 734 |
| Actinoplanes missouriensis | 3720 | 3003 | 8556 | 249 | 6636 |
| Actinoplanes teichomyceticus | 3898 | 2979 | 7695 | 217 | 8092 |
| Micromonospora chalcea | 1775 | 1614 | 4745 | 170 | 6670 |
| Micromonospora echinospora | 3207 | 2159 | 6541 | 367 | 10063 |
| Micromonospora viridifaciens | 3210 | 3126 | 7217 | 514 | 10805 |
| Streptomyces actuosus | 2165 | 1929 | 3615 | 151 | 4648 |
| Streptomyces albidoflavus | 5631 | 5268 | 10221 | 265 | 10111 |
| Streptomyces albus | 6161 | 5988 | 11341 | 1037 | 23376 |
| Streptomyces ambofaciens | 2047 | 1762 | 3291 | 137 | 3800 |
| Streptomyces antibioticus | 2029 | 1937 | 3163 | 246 | 6540 |
| Streptomyces anulatus | 2846 | 2794 | 4925 | 549 | 20770 |
| Streptomyces atratus | 2455 | 1924 | 4285 | 649 | 11435 |
| Kitasatospora aureofaciens | 2132 | 1729 | 3114 | 269 | 6113 |
| Streptomyces clavuligerus | 2351 | 1927 | 3912 | 239 | 7322 |
| Streptomyces coelicolor | 1752 | 1260 | 2644 | 362 | 6211 |
| Streptomyces cyaneus | 0 | 0 | 1 | 0 | 0 |
| Streptomyces exfoliatus | 1981 | 2187 | 3887 | 164 | 5583 |
| Streptomyces fradiae | 1792 | 2922 | 3660 | 81 | 4133 |
| Streptomyces glaucescens | 1893 | 1637 | 3054 | 246 | 6052 |
| Streptomyces globisporus | 588 | 845 | 2298 | 47 | 4056 |
| Streptomyces griseus | 1950 | 2008 | 3475 | 316 | 7772 |
| Streptomyces hygroscopicus | 8714 | 6039 | 11199 | 1656 | 19877 |
| Streptomyces lavendulae | 2119 | 2000 | 4035 | 311 | 6396 |
| Streptomyces lincolnensis | 2704 | 2050 | 4349 | 285 | 6633 |
| Streptomyces lividans | 69933 | 93589 | 197714 | 16374 | 378440 |
| Streptomyces microflavus | 1194 | 1543 | 3143 | 257 | 4878 |
| Streptomyces nigrescens | 1736 | 1915 | 2739 | 88 | 4244 |
| Streptomyces phaeochromogenes | 0 | 0 | 2 | 0 | 8 |
| Streptomyces rimosus | 2888 | 4442 | 6122 | 913 | 8927 |
| Streptomyces rochei | 359 | 619 | 1222 | 0 | 2175 |
| Streptomyces scabiei | 1827 | 2090 | 2000 | 331 | 5504 |
| Streptomyces tendae | 1269 | 1400 | 2569 | 249 | 4624 |
| Streptomyces violaceoruber | 641 | 1135 | 1145 | 92 | 3371 |
| Streptomyces albireticuli | 1185 | 1037 | 1650 | 361 | 7109 |
| Streptomyces peucetius | 3740 | 3696 | 6904 | 833 | 16608 |
| Streptomyces vinaceus | 2071 | 1660 | 3891 | 222 | 5461 |
| Streptomyces kanamyceticus | 1743 | 1587 | 3196 | 121 | 4508 |
| Streptomyces chartreusis | 3756 | 3512 | 6346 | 258 | 8878 |
| Streptomyces noursei | 3816 | 3586 | 6033 | 279 | 8487 |
| Actinomadura madurae | 7824 | 4542 | 12031 | 1297 | 25964 |
| Streptosporangium roseum | 7210 | 6047 | 112299 | 230938 | 3445251 |
| Thermobispora bispora | 3256 | 2801 | 8989 | 3909 | 68154 |
| Nocardiopsis dassonvillei | 2430 | 1799 | 4580 | 1177 | 14879 |
| Thermomonospora curvata | 3197 | 2407 | 6065 | 1455 | 25591 |
| Thermobifida fusca | 801 | 792 | 1919 | 391 | 5459 |
| Thermoactinomyces vulgaris | 31 | 200 | 151 | 6 | 205 |
| Microbacterium testaceum | 1002 | 1152 | 7574 | 42 | 4878 |
| Curtobacterium flaccumfaciens | 1962 | 2639 | 4185 | 3 | 3282 |
| Tropheryma whipplei | 9 | 24 | 51 | 4 | 117 |
| Aeromicrobium erythreum | 9961 | 18992 | 14366 | 54 | 6527 |
| Pimelobacter simplex | 7282 | 10291 | 12446 | 387 | 13424 |
| Gordonia bronchialis | 1334 | 1532 | 2057 | 35 | 2655 |
| Gordonia terrae | 1713 | 1235 | 2850 | 61 | 2003 |
| Sphaerobacter thermophilus | 2903 | 4090 | 5286 | 60 | 6319 |
| Tsukamurella paurometabola | 2527 | 2732 | 4489 | 131 | 5734 |
| Kitasatospora setae | 3065 | 2911 | 5289 | 249 | 8164 |
| Pseudonocardia autotrophica | 5558 | 3782 | 8583 | 351 | 8771 |
| Mycoplasmopsis arginini | 2 | 1 | 1 | 2 | 37 |
| Mycoplasma capricolum | 48 | 5 | 9 | 0 | 11 |
| Mycoplasmoides gallisepticum | 33 | 90 | 26 | 0 | 40 |
| Mycoplasmoides genitalium | 7 | 9 | 3 | 0 | 7 |
| Metamycoplasma hominis | 14 | 14 | 20 | 0 | 6 |
| Mesomycoplasma hyopneumoniae | 0 | 3 | 8 | 0 | 8 |
| Mesomycoplasma hyorhinis | 0 | 0 | 0 | 0 | 0 |
| Mycoplasma mycoides | 10 | 63 | 27 | 2 | 23 |
| Mycoplasmoides pneumoniae | 6 | 4 | 5 | 0 | 8 |
| Mycoplasma leachii | 0 | 10 | 0 | 0 | 14 |
| Mycoplasmopsis pulmonis | 1 | 1 | 2 | 0 | 4 |
| Mycoplasmopsis synoviae | 0 | 10 | 14 | 0 | 8 |
| Mycoplasmopsis agalactiae | 0 | 0 | 0 | 0 | 0 |
| Metamycoplasma arthritidis | 7 | 3 | 3 | 0 | 3 |
| Mycoplasmopsis bovigenitalium | 2 | 3 | 1 | 0 | 5 |
| Mycoplasmopsis californica | 0 | 6 | 8 | 0 | 5 |
| Mycoplasmopsis fermentans | 2 | 0 | 8 | 0 | 13 |
| Malacoplasma iowae | 0 | 9 | 3 | 1 | 6 |
| Mesomycoplasma mobile | 1 | 2 | 3 | 0 | 1 |
| Mesomycoplasma neurolyticum | 0 | 0 | 0 | 0 | 0 |
| Metamycoplasma orale | 5 | 18 | 3 | 0 | 3 |
| Metamycoplasma salivarium | 0 | 1 | 4 | 0 | 0 |
| Mesomycoplasma flocculare | 0 | 1 | 3 | 8 | 3 |
| Ureaplasma urealyticum | 0 | 0 | 4 | 0 | 8 |
| Spiroplasma citri | 0 | 0 | 0 | 0 | 0 |
| Spiroplasma melliferum | 0 | 10 | 4 | 0 | 5 |
| Spiroplasma monobiae | 0 | 1 | 0 | 0 | 0 |
| Spiroplasma apis | 1 | 4 | 4 | 1 | 2 |
| Spiroplasma poulsonii | 1 | 3 | 3 | 0 | 8 |
| Spiroplasma clarkii | 0 | 32 | 4 | 0 | 3 |
| Spiroplasma gladiatoris | 0 | 0 | 0 | 0 | 0 |
| Spiroplasma taiwanense | 0 | 0 | 0 | 0 | 0 |
| Acholeplasma laidlawii | 8 | 17 | 23 | 0 | 23 |
| Mesoplasma florum | 0 | 2 | 7 | 0 | 0 |
| Desulfobacter hydrogenophilus | 0 | 0 | 0 | 0 | 0 |
| Desulfobacter postgatei | 69 | 193 | 203 | 10 | 170 |
| Desulforapulum autotrophicum | 78 | 227 | 264 | 1 | 465 |
| Thermoanaerobacter kivui | 14 | 25 | 41 | 2 | 26 |
| Halanaerobium praevalens | 17 | 16 | 15 | 0 | 59 |
| Thermotoga maritima | 0 | 18 | 160 | 0 | 0 |
| Thermotoga neapolitana | 0 | 0 | 3 | 0 | 10 |
| Haliscomenobacter hydrossis | 187 | 472 | 311 | 1 | 390 |
| Flexistipes sinusarabici | 0 | 0 | 0 | 0 | 0 |
| unidentified bacterial endosymbiont | 381 | 752 | 481 | 28 | 454 |
| Desulfomonile tiedjei | 260 | 624 | 506 | 0 | 875 |
| Bathymodiolus thermophilus thioautotrophic gill symbiont | 21 | 25 | 33 | 0 | 35 |
| Xylella fastidiosa | 174 | 469 | 289 | 20 | 282 |
| Sporomusa termitida | 103 | 176 | 226 | 4 | 172 |
| Thermosipho africanus | 8 | 36 | 17 | 0 | 10 |
| Fervidobacterium islandicum | 0 | 18 | 49 | 0 | 97 |
| Fervidobacterium nodosum | 17 | 6 | 10 | 0 | 6 |
| Teredinibacter turnerae | 0 | 0 | 0 | 0 | 0 |
| Roseobacter denitrificans | 0 | 0 | 0 | 0 | 0 |
| Hirschia baltica | 68 | 74 | 36 | 1 | 60 |
| Symbiobacterium thermophilum | 1202 | 2114 | 2450 | 61 | 3110 |
| Verrucomicrobium spinosum | 1272 | 3527 | 2840 | 11 | 3636 |
| Vagococcus fluvialis | 51 | 3 | 22 | 4 | 19 |
| Marinobacter nauticus | 0 | 0 | 0 | 0 | 0 |
| Halomonas elongata | 544 | 679 | 711 | 3 | 841 |
| Carnobacterium divergens | 13 | 16 | 11 | 0 | 26 |
| Carnobacterium maltaromaticum | 18 | 132 | 28 | 0 | 45 |
| Burkholderia mallei | 677 | 1044 | 1867 | 80 | 4198 |
| Sphingobium yanoikuyae | 3114 | 2848 | 1637 | 0 | 1483 |
| Bifidobacterium animalis | 1675 | 2213 | 2449 | 320 | 3129 |
| Bifidobacterium pseudocatenulatum | 138 | 201 | 288 | 18 | 319 |
| Corynebacterium flavescens | 129 | 231 | 268 | 15 | 313 |
| Lysinibacillus fusiformis | 119 | 49 | 58 | 0 | 35 |
| Latilactobacillus curvatus | 51 | 35 | 74 | 0 | 59 |
| Mycobacterium celatum | 12 | 16 | 7 | 0 | 29 |
| Mycolicibacterium confluentis | 2178 | 4972 | 3933 | 16 | 7484 |
| Acidothermus cellulolyticus | 694 | 1101 | 1500 | 222 | 3604 |
| Rubrivivax gelatinosus | 1633 | 2757 | 2915 | 73 | 3574 |
| Nostoc sp. PCC 7524 | 25 | 106 | 69 | 0 | 177 |
| Nitrospirillum amazonense | 2579 | 2919 | 2876 | 88 | 3841 |
| Campylobacter upsaliensis | 4 | 9 | 6 | 0 | 10 |
| Legionella anisa | 63 | 55 | 35 | 0 | 32 |
| Legionella cherrii | 39 | 27 | 40 | 3 | 47 |
| Legionella sainthelensi | 15 | 68 | 37 | 2 | 31 |
| Neisseria weaveri | 50 | 84 | 88 | 0 | 107 |
| Burkholderia gladioli | 4346 | 4707 | 5568 | 43 | 7374 |
| Ralstonia syzygii | 66 | 334 | 224 | 2 | 268 |
| Agrobacterium rubi | 615 | 701 | 634 | 0 | 939 |
| Mesorhizobium huakuii | 2326 | 1997 | 2155 | 7 | 1648 |
| Pseudoalteromonas espejiana | 2 | 17 | 4 | 1 | 9 |
| Alteromonas macleodii | 121 | 274 | 218 | 8 | 192 |
| Pseudoalteromonas nigrifaciens | 6 | 9 | 5 | 0 | 5 |
| Francisella philomiragia | 0 | 9 | 15 | 0 | 9 |
| Bacteroides eggerthii | 115 | 169 | 101 | 0 | 197 |
| Bacteroides heparinolyticus | 29 | 84 | 74 | 0 | 106 |
| Bacteroides ovatus | 0 | 0 | 0 | 0 | 0 |
| Odoribacter splanchnicus | 61 | 68 | 71 | 0 | 51 |
| Bacteroides zoogleoformans | 132 | 245 | 203 | 2 | 125 |
| Porphyromonas asaccharolytica | 51 | 107 | 147 | 1 | 156 |
| Prevotella veroralis | 1 | 15 | 14 | 0 | 10 |
| Cronobacter sakazakii | 0 | 0 | 0 | 0 | 0 |
| Serratia proteamaculans | 0 | 0 | 0 | 0 | 0 |
| Yersinia kristensenii | 29 | 50 | 28 | 6 | 57 |
| Vibrio pelagius | 0 | 0 | 0 | 0 | 0 |
| Vibrio metschnikovii | 30 | 64 | 40 | 1 | 38 |
| Vibrio nigripulchritudo | 44 | 62 | 59 | 1 | 89 |
| Leptospira noguchii | 32 | 96 | 35 | 1 | 79 |
| Leptospira santarosai | 0 | 0 | 0 | 0 | 0 |
| Leptospira weilii | 19 | 49 | 67 | 0 | 46 |
| Acetohalobium arabaticum | 8 | 32 | 43 | 0 | 50 |
| Capnocytophaga cynodegmi | 1 | 12 | 21 | 0 | 10 |
| Aliarcobacter butzleri | 0 | 0 | 0 | 0 | 0 |
| Aliarcobacter cryaerophilus | 0 | 0 | 0 | 0 | 0 |
| Arcobacter nitrofigilis | 5 | 2 | 5 | 1 | 4 |
| Aliarcobacter skirrowii | 1 | 4 | 9 | 0 | 2 |
| Sphingomonas sp. | 0 | 0 | 0 | 0 | 0 |
| Desulfobacula toluolica | 61 | 147 | 96 | 0 | 104 |
| Malacoplasma penetrans | 22 | 20 | 40 | 0 | 30 |
| Colwellia psychrerythraea | 13 | 133 | 24 | 38 | 18 |
| Geobacter metallireducens | 688 | 1434 | 1259 | 13 | 1490 |
| Ornithobacterium rhinotracheale | 69 | 107 | 89 | 1 | 110 |
| Cobetia marina | 18 | 164 | 312 | 0 | 490 |
| Thermodesulfovibrio yellowstonii | 51 | 90 | 113 | 0 | 145 |
| Arcanobacterium haemolyticum | 0 | 0 | 0 | 0 | 0 |
| Anaerotignum propionicum | 0 | 0 | 0 | 0 | 0 |
| Clavibacter michiganensis | 5681 | 6660 | 11406 | 257 | 12293 |
| Komagataeibacter xylinus | 1478 | 1880 | 1693 | 28 | 2515 |
| Burkholderia pseudomallei | 13112 | 10736 | 8111 | 903 | 36391 |
| Sphingobacterium multivorum | 109 | 171 | 189 | 0 | 212 |
| Hydrogenovibrio marinus | 33 | 49 | 76 | 0 | 35 |
| Streptomyces nitrosporeus | 1363 | 1215 | 2612 | 332 | 7136 |
| Campylobacter helveticus | 6 | 30 | 14 | 0 | 127 |
| Salmonella enterica | 28119 | 43541 | 33160 | 1842 | 246323 |
| Mycoplasmopsis bovis | 21 | 13 | 5 | 2 | 14 |
| Streptantibioticus cattleyicolor | 2631 | 2114 | 3933 | 343 | 7877 |
| Mycobacterium haemophilum | 1196 | 1989 | 1433 | 48 | 2482 |
| Mycolicibacter hiberniae | 1183 | 2016 | 1802 | 156 | 3911 |
| Paenarthrobacter nicotinovorans | 424 | 305 | 575 | 13 | 880 |
| Caldicellulosiruptor naganoensis | 0 | 0 | 0 | 0 | 0 |
| Thermoanaerobacterium xylanolyticum | 37 | 21 | 14 | 0 | 32 |
| Clostridium argentinense | 51 | 29 | 31 | 1 | 28 |
| [Clostridium] scindens | 87 | 281 | 172 | 1 | 333 |
| Thomasclavelia spiroformis | 0 | 2 | 25 | 0 | 12 |
| Cellulosilyticum lentocellum | 9 | 14 | 27 | 0 | 35 |
| Lacrimispora sphenoides | 14 | 76 | 99 | 1 | 123 |
| Lacrimispora xylanolytica | 15 | 29 | 49 | 0 | 37 |
| Staphylococcus arlettae | 0 | 0 | 0 | 0 | 0 |
| Staphylococcus auricularis | 0 | 0 | 0 | 0 | 0 |
| Staphylococcus cohnii | 0 | 0 | 0 | 0 | 0 |
| Staphylococcus kloosii | 2 | 0 | 4 | 0 | 6 |
| Streptococcus alactolyticus | 0 | 0 | 0 | 0 | 0 |
| Microlunatus phosphovorus | 1594 | 2424 | 3992 | 136 | 6065 |
| Helicobacter canis | 4 | 12 | 31 | 4 | 23 |
| Legionella oakridgensis | 50 | 77 | 38 | 0 | 53 |
| Methylorubrum zatmanii | 623 | 854 | 1064 | 0 | 943 |
| Moraxella ovis | 6 | 20 | 13 | 0 | 34 |
| Pseudomonas savastanoi | 205 | 312 | 472 | 18 | 506 |
| Pseudomonas tolaasii | 149 | 1353 | 307 | 165 | 604 |
| Xanthomonas albilineans | 674 | 965 | 732 | 14 | 828 |
| Bradyrhizobium elkanii | 9137 | 7888 | 5228 | 35 | 3835 |
| Rhizobium etli | 3209 | 2885 | 2742 | 93 | 2865 |
| Brucella melitensis | 881 | 1954 | 354 | 0 | 1414 |
| Brucella suis | 231 | 256 | 724 | 0 | 460 |
| Pectobacterium atrosepticum | 104 | 257 | 101 | 0 | 137 |
| Yersinia aldovae | 12 | 38 | 43 | 0 | 57 |
| Yersinia frederiksenii | 91 | 63 | 48 | 0 | 179 |
| Yersinia rohdei | 22 | 56 | 61 | 3 | 114 |
| Yersinia ruckeri | 54 | 146 | 81 | 15 | 194 |
| Aeromonas enteropelogenes | 471 | 540 | 633 | 6 | 800 |
| Vibrio splendidus | 42 | 343 | 44 | 2 | 104 |
| Vibrio tubiashii | 42 | 122 | 74 | 4 | 74 |
| Mycoplasma haemofelis | 1 | 3 | 7 | 0 | 16 |
| Leptospira kirschneri | 74 | 26 | 22 | 3 | 24 |
| Turneriella parva | 266 | 512 | 398 | 9 | 512 |
| Borreliella afzelii | 0 | 9 | 6 | 0 | 38 |
| Borreliella garinii | 0 | 0 | 0 | 0 | 0 |
| Borrelia crocidurae | 5 | 0 | 0 | 0 | 9 |
| Thermoleophilum album | 5776 | 4096 | 10853 | 59 | 12271 |
| Syntrophotalea acetylenica | 229 | 720 | 695 | 4 | 812 |
| Pelobacter propionicus | 1231 | 2718 | 2822 | 14 | 2618 |
| Lawsonia intracellularis | 2 | 9 | 4 | 0 | 23 |
| Rhodothermus marinus | 1893 | 4944 | 4740 | 39 | 5947 |
| Mycoplasmopsis bovirhinis | 1 | 2 | 16 | 0 | 0 |
| Metamycoplasma canadense | 2 | 1 | 7 | 0 | 9 |
| Mycoplasmopsis canis | 1 | 4 | 11 | 0 | 16 |
| Mycoplasmopsis gallinacea | 0 | 2 | 12 | 0 | 10 |
| Metamycoplasma hyosynoviae | 2 | 3 | 11 | 0 | 2 |
| Mycoplasmopsis meleagridis | 11 | 9 | 5 | 0 | 15 |
| Mesomycoplasma ovipneumoniae | 0 | 2 | 6 | 11 | 70 |
| Halomonas meridiana | 502 | 915 | 765 | 46 | 1098 |
| Halomonas subglaciescola | 272 | 491 | 427 | 24 | 558 |
| Taylorella equigenitalis | 18 | 20 | 40 | 4 | 106 |
| Janthinobacterium lividum | 849 | 1825 | 1493 | 0 | 8918 |
| Caldicellulosiruptor bescii | 0 | 8 | 4 | 0 | 9 |
| Halothermothrix orenii | 42 | 56 | 70 | 1 | 74 |
| Amycolatopsis orientalis | 18965 | 2555 | 9395 | 155 | 7794 |
| Methylobacterium radiotolerans | 0 | 0 | 0 | 0 | 0 |
| Achromobacter denitrificans | 2878 | 3450 | 4387 | 80 | 5769 |
| Helicobacter hepaticus | 51 | 2 | 6 | 0 | 8 |
| Synechococcus elongatus | 258 | 568 | 510 | 4 | 667 |
| Synechococcus sp. PCC 7002 | 0 | 83 | 43 | 0 | 88 |
| Synechococcus sp. WH 8020 | 91 | 102 | 73 | 1 | 98 |
| Deinococcus radiophilus | 0 | 0 | 0 | 0 | 0 |
| Rhodococcus globerulus | 705 | 1383 | 1480 | 62 | 9072 |
| Phascolarctobacterium faecium | 85 | 152 | 110 | 0 | 170 |
| Blautia producta | 44 | 76 | 67 | 0 | 156 |
| Anaerococcus vaginalis | 0 | 0 | 0 | 0 | 0 |
| [Ruminococcus] gnavus | 5324 | 245 | 1960 | 0 | 4099 |
| [Ruminococcus] torques | 4 | 45 | 29 | 0 | 40 |
| Streptococcus milleri | 0 | 0 | 0 | 0 | 0 |
| Coprococcus eutactus | 24 | 19 | 17 | 0 | 195 |
| Bartonella grahamii | 0 | 6 | 4 | 0 | 4 |
| Bartonella taylorii | 1 | 12 | 12 | 0 | 15 |
| Bartonella vinsonii | 31 | 22 | 14 | 0 | 20 |
| Sphingopyxis macrogoltabida | 1534 | 2116 | 1362 | 0 | 824 |
| Sphingopyxis terrae | 904 | 1325 | 994 | 0 | 1024 |
| Candidatus Kinetoplastibacterium crithidii | 0 | 0 | 0 | 0 | 0 |
| Acidithiobacillus caldus | 281 | 689 | 793 | 2 | 1060 |
| Yersinia mollaretii | 18 | 57 | 55 | 5 | 59 |
| Pseudomonas viridiflava | 624 | 1321 | 804 | 26 | 1386 |
| Thermostichus lividus | 67 | 112 | 83 | 14 | 121 |
| Gloeobacter violaceus | 651 | 1333 | 1144 | 5 | 1726 |
| Zymobacter palmae | 155 | 351 | 222 | 1 | 297 |
| Acidobacterium capsulatum | 1249 | 3941 | 2783 | 30 | 3544 |
| Coriobacterium glomerans | 251 | 370 | 497 | 2 | 536 |
| Curtobacterium luteum | 923 | 1040 | 1401 | 5 | 1104 |
| Rathayibacter rathayi | 479 | 632 | 753 | 25 | 1095 |
| Rathayibacter tritici | 626 | 755 | 978 | 51 | 1527 |
| Brevibacterium casei | 0 | 0 | 0 | 0 | 0 |
| Streptomyces galilaeus | 1450 | 1943 | 4733 | 67 | 5762 |
| Streptomyces murinus | 2423 | 2555 | 3847 | 165 | 5936 |
| Streptomyces avermitilis | 1897 | 1812 | 3290 | 268 | 6177 |
| Bifidobacterium thermophilum | 0 | 0 | 0 | 0 | 0 |
| Amycolatopsis mediterranei | 32752 | 8710 | 29676 | 297 | 27403 |
| Saccharopolyspora gregorii | 6348 | 2562 | 6666 | 190 | 6659 |
| Microbacterium liquefaciens | 564 | 915 | 1153 | 28 | 1153 |
| Mycoplasmopsis felis | 7 | 14 | 8 | 0 | 15 |
| Sutcliffiella cohnii | 0 | 0 | 0 | 0 | 0 |
| Aeribacillus pallidus | 0 | 0 | 0 | 0 | 0 |
| Geobacillus thermocatenulatus | 0 | 0 | 0 | 0 | 0 |
| Geobacillus thermodenitrificans | 148 | 59 | 63 | 2 | 158 |
| Geobacillus thermoleovorans | 0 | 0 | 0 | 0 | 0 |
| Kyrpidia tusciae | 172 | 530 | 377 | 0 | 388 |
| Enterococcus avium | 0 | 0 | 0 | 0 | 0 |
| Acetobacterium woodii | 25 | 42 | 86 | 0 | 57 |
| Lactobacillus johnsonii | 22 | 15 | 19 | 0 | 143 |
| Lentilactobacillus kefiri | 17 | 29 | 5 | 0 | 19 |
| Leuconostoc citreum | 0 | 5 | 0 | 0 | 12 |
| Leuconostoc pseudomesenteroides | 0 | 0 | 0 | 0 | 0 |
| Melissococcus plutonius | 1 | 11 | 3 | 0 | 6 |
| Rickettsia amblyommatis | 1 | 12 | 14 | 0 | 76 |
| Rickettsia bellii | 0 | 9 | 31 | 0 | 19 |
| Rickettsia montanensis | 0 | 0 | 0 | 0 | 0 |
| Rickettsia rhipicephali | 0 | 4 | 0 | 0 | 1 |
| Neorickettsia helminthoeca | 3 | 18 | 11 | 0 | 12 |
| Gluconacetobacter diazotrophicus | 854 | 1213 | 1094 | 8 | 1463 |
| Paracoccus alcaliphilus | 631 | 563 | 824 | 0 | 576 |
| Paracoccus aminophilus | 541 | 658 | 683 | 12 | 889 |
| Paracoccus aminovorans | 539 | 811 | 558 | 10 | 535 |
| Rhodospirillum centenum | 2788 | 2855 | 2965 | 69 | 4377 |
| Candidatus Liberibacter africanus | 36 | 32 | 30 | 2 | 26 |
| Candidatus Liberibacter asiaticus | 49 | 32 | 86 | 26 | 372 |
| Leptothrix cholodnii | 1831 | 2489 | 2349 | 46 | 3440 |
| Moraxella cuniculi | 7 | 23 | 39 | 7 | 62 |
| Variovorax paradoxus | 5657 | 11563 | 7795 | 0 | 351 |
| Riemerella anatipestifer | 0 | 0 | 0 | 0 | 0 |
| Sphingobacterium faecium | 62 | 20 | 16 | 0 | 11 |
| Pelodictyon phaeoclathratiforme | 83 | 238 | 208 | 9 | 255 |
| Geobacter sulfurreducens | 1118 | 2478 | 2340 | 26 | 2850 |
| Streptomyces mobaraensis | 3207 | 4092 | 6385 | 646 | 17635 |
| Heliomicrobium modesticaldum | 417 | 991 | 1030 | 0 | 1157 |
| Citrobacter amalonaticus | 0 | 0 | 0 | 0 | 0 |
| Dactylosporangium aurantiacum | 7488 | 5662 | 16857 | 735 | 17160 |
| Corynebacterium kutscheri | 62 | 34 | 29 | 1 | 32 |
| Bifidobacterium choerinum | 717 | 1016 | 1166 | 28 | 1602 |
| Planobispora rosea | 13 | 2 | 1 | 0 | 2 |
| Candidatus Phytoplasma luffae | 0 | 1 | 1 | 0 | 4 |
| Coprothermobacter proteolyticus | 22 | 132 | 130 | 11 | 99 |
| Limosilactobacillus pontis | 0 | 0 | 0 | 0 | 0 |
| Rickettsia helvetica | 0 | 0 | 1 | 0 | 0 |
| Rickettsia japonica | 0 | 9 | 0 | 0 | 1 |
| Rickettsia massiliae | 0 | 3 | 4 | 0 | 4 |
| Rickettsia parkeri | 1 | 6 | 3 | 0 | 0 |
| Rickettsia sibirica | 0 | 0 | 0 | 0 | 3 |
| Ehrlichia muris | 33 | 13 | 16 | 0 | 8 |
| Nitrosospira briensis | 244 | 944 | 388 | 0 | 636 |
| Rhodovulum sulfidophilum | 1288 | 1349 | 1335 | 9 | 1618 |
| Bordetella holmesii | 555 | 787 | 740 | 25 | 966 |
| Helicobacter heilmannii | 15 | 40 | 50 | 0 | 36 |
| Helicobacter pullorum | 3 | 6 | 16 | 0 | 3 |
| Clostridium saccharoperbutylacetonicum | 50 | 45 | 14 | 0 | 10 |
| Pseudomonas cichorii | 0 | 0 | 0 | 0 | 0 |
| Corynebacterium bovis | 682 | 709 | 1182 | 14 | 1133 |
| Mycolicibacterium pulveris | 3438 | 12043 | 7462 | 81 | 20628 |
| Mycolicibacterium rhodesiae | 4123 | 15577 | 8802 | 77 | 25231 |
| Streptomyces subrutilus | 1948 | 1913 | 3747 | 210 | 6362 |
| Gordonia amarae | 1311 | 1417 | 2042 | 53 | 2485 |
| Gordonia rubripertincta | 1815 | 1669 | 2669 | 57 | 3135 |
| Clostridium felsineum | 50 | 47 | 73 | 5 | 54 |
| Clostridium intestinale | 0 | 0 | 0 | 0 | 0 |
| Desulfitobacterium dehalogenans | 71 | 87 | 67 | 0 | 97 |
| Brucella canis | 2948 | 0 | 202 | 0 | 62 |
| Thiobacillus denitrificans | 1672 | 2398 | 2519 | 79 | 4268 |
| Ideonella dechloratans | 1147 | 1240 | 1536 | 0 | 1576 |
| Paraburkholderia xenovorans | 1872 | 2574 | 2642 | 13 | 3038 |
| Porphyromonas cangingivalis | 26 | 46 | 29 | 3 | 51 |
| Nocardia brasiliensis | 7179 | 6056 | 12389 | 428 | 14446 |
| Nocardia farcinica | 5038 | 3719 | 8199 | 340 | 9964 |
| Nocardia nova | 1899 | 2103 | 3185 | 163 | 4505 |
| Pseudonocardia petroleophila | 5468 | 3628 | 11808 | 301 | 10328 |
| Nocardia seriolae | 2149 | 2079 | 3387 | 76 | 3908 |
| Helicobacter bilis | 4 | 5 | 4 | 0 | 6 |
| Polaromonas vacuolata | 91 | 303 | 179 | 3 | 390 |
| Laceyella sacchari | 129 | 342 | 359 | 0 | 484 |
| Marichromatium purpuratum | 1439 | 1928 | 2059 | 22 | 2200 |
| Candidatus Phytoplasma mali | 13 | 2 | 0 | 0 | 1 |
| Rhodococcus opacus | 7732 | 16243 | 15223 | 468 | 25256 |
| Arthrobacter agilis | 1633 | 1831 | 2948 | 243 | 3573 |
| Rothia kristinae | 920 | 1639 | 2463 | 268 | 3789 |
| Sinomonas atrocyanea | 1571 | 1855 | 2756 | 36 | 2955 |
| Arthrobacter crystallopoietes | 1194 | 1727 | 2651 | 87 | 3703 |
| Glutamicibacter nicotianae | 0 | 0 | 0 | 0 | 0 |
| Glutamicibacter protophormiae | 846 | 1143 | 1930 | 41 | 2607 |
| Paenarthrobacter ureafaciens | 1011 | 2069 | 2917 | 54 | 3649 |
| Corynebacterium jeikeium | 605 | 704 | 1628 | 0 | 0 |
| Photobacterium damselae | 0 | 0 | 0 | 0 | 0 |
| Streptomyces pristinaespiralis | 1790 | 1704 | 3237 | 114 | 4592 |
| Corynebacterium vitaeruminis | 523 | 647 | 962 | 53 | 1786 |
| Gluconobacter frateurii | 134 | 197 | 154 | 4 | 363 |
| Rhodococcus coprophilus | 1216 | 1262 | 1685 | 108 | 2228 |
| Shewanella algae | 298 | 441 | 380 | 11 | 323 |
| Pseudothermotoga elfii | 5 | 41 | 0 | 0 | 0 |
| Bartonella henselae | 16 | 16 | 15 | 0 | 13 |
| Heyndrickxia oleronia | 80 | 65 | 92 | 0 | 53 |
| Alteracholeplasma palmae | 13 | 192 | 30 | 0 | 60 |
| Skermania piniformis | 1378 | 1654 | 2554 | 93 | 3003 |
| Streptomyces laurentii | 2 | 0 | 2 | 0 | 3 |
| Faecalitalea cylindroides | 15 | 68 | 35 | 0 | 22 |
| Lachnospira eligens | 37 | 19 | 27 | 0 | 66 |
| Dorea formicigenerans | 6 | 34 | 37 | 0 | 27 |
| Eubacterium ventriosum | 11 | 20 | 10 | 0 | 24 |
| Mesorhizobium ciceri | 4666 | 4855 | 4467 | 2 | 4366 |
| Paulownia witches'-broom phytoplasma | 0 | 0 | 4 | 1 | 5 |
| Mycolicibacterium austroafricanum | 10279 | 28998 | 21155 | 309 | 51210 |
| Mycolicibacterium duvalii | 2024 | 5980 | 4398 | 69 | 9462 |
| Mycolicibacterium moriokaense | 3971 | 14506 | 8659 | 116 | 24697 |
| Mycolicibacterium parafortuitum | 2641 | 6782 | 4800 | 29 | 11007 |
| Mycolicibacterium poriferae | 2449 | 7709 | 5258 | 87 | 12291 |
| Mycolicibacterium tokaiense | 2060 | 4884 | 4043 | 69 | 6932 |
| Hydrogenovibrio crunogenus | 6 | 39 | 25 | 10 | 36 |
| Sulfurimonas denitrificans | 1 | 5 | 2 | 0 | 2 |
| Blattabacterium sp. (Mastotermes darwiniensis) | 1 | 5 | 4 | 0 | 2 |
| Methylomicrobium album | 671 | 1151 | 1115 | 0 | 1337 |
| Veillonella dispar | 0 | 0 | 0 | 0 | 0 |
| Corynebacterium glucuronolyticum | 0 | 0 | 0 | 0 | 0 |
| Dialister pneumosintes | 13 | 9 | 15 | 0 | 20 |
| Methylobacterium mesophilicum | 745 | 1051 | 921 | 0 | 613 |
| Erythrobacter litoralis | 1108 | 1860 | 1570 | 69 | 2572 |
| Helcococcus kunzii | 12 | 11 | 18 | 0 | 6 |
| Afipia carboxidovorans | 3614 | 1252 | 1663 | 0 | 1307 |
| Aliivibrio salmonicida | 6 | 17 | 53 | 2 | 48 |
| Streptomyces nodosus | 1614 | 1350 | 2778 | 247 | 5466 |
| Stenotrophomonas maltophilia | 9819 | 10030 | 9502 | 0 | 3928 |
| Blautia obeum | 32 | 109 | 106 | 1 | 307 |
| Leptotrichia buccalis | 1 | 5 | 12 | 0 | 7 |
| Sutterella wadsworthensis | 134 | 368 | 245 | 4 | 366 |
| Actinosynnema mirum | 6641 | 2271 | 5723 | 316 | 7803 |
| Xenorhabdus bovienii | 120 | 108 | 80 | 0 | 74 |
| Xenorhabdus poinarii | 7 | 110 | 35 | 1 | 32 |
| Saccharomonospora azurea | 5248 | 1847 | 5905 | 139 | 5135 |
| Saccharomonospora cyanea | 6158 | 2201 | 5944 | 214 | 5977 |
| Saccharomonospora glauca | 4033 | 1420 | 4927 | 161 | 3927 |
| Exiguobacterium acetylicum | 0 | 0 | 0 | 0 | 0 |
| Ewingella americana | 0 | 0 | 0 | 0 | 0 |
| Brevundimonas vesicularis | 0 | 0 | 0 | 0 | 0 |
| Burkholderia plantarii | 1217 | 1514 | 1933 | 24 | 2507 |
| Parazoarcus communis | 1713 | 2560 | 2958 | 11 | 3903 |
| Ureaplasma diversum | 1 | 3 | 3 | 0 | 2 |
| Actinosynnema pretiosum | 14680 | 5901 | 10614 | 317 | 10548 |
| Streptomyces sampsonii | 593 | 629 | 1905 | 8 | 1788 |
| Nitrospira moscoviensis | 3745 | 6929 | 6670 | 30 | 6274 |
| Rubrobacter radiotolerans | 969 | 1623 | 2247 | 46 | 2804 |
| Halobacteroides halobius | 54 | 23 | 55 | 0 | 60 |
| Roseobacter litoralis | 234 | 470 | 329 | 3 | 398 |
| Streptomyces collinus | 1914 | 2081 | 2954 | 123 | 3733 |
| Corynebacterium argentoratense | 98 | 168 | 159 | 11 | 262 |
| Ammonifex degensii | 186 | 373 | 452 | 0 | 705 |
| Mammaliicoccus lentus | 0 | 0 | 0 | 0 | 0 |
| Rickettsia felis | 0 | 0 | 0 | 0 | 2 |
| Streptomyces chromofuscus | 1824 | 1724 | 2666 | 168 | 4189 |
| Cereibacter azotoformans | 737 | 1380 | 1187 | 0 | 1403 |
| Pseudomonas alcaligenes | 0 | 0 | 0 | 0 | 0 |
| Butyrivibrio proteoclasticus | 0 | 0 | 0 | 0 | 0 |
| Mycobacterium branderi | 2408 | 6004 | 4108 | 170 | 10090 |
| Kutzneria albida | 18117 | 4754 | 14910 | 826 | 20927 |
| Pseudoalteromonas luteoviolacea | 41 | 151 | 54 | 7 | 102 |
| Pseudoalteromonas rubra | 137 | 368 | 242 | 22 | 275 |
| Pseudoalteromonas tetraodonis | 0 | 72 | 43 | 0 | 10 |
| Pseudoalteromonas undina | 3 | 62 | 23 | 0 | 37 |
| Shewanella benthica | 40 | 39 | 50 | 1 | 35 |
| Pseudoalteromonas piscicida | 14 | 81 | 124 | 14 | 180 |
| Paenarthrobacter aurescens | 774 | 933 | 2277 | 14 | 2657 |
| Brachybacterium faecium | 836 | 1078 | 1708 | 28 | 1607 |
| Jonesia denitrificans | 0 | 0 | 0 | 0 | 0 |
| Corynebacterium amycolatum | 347 | 485 | 1029 | 70 | 1434 |
| Prescottella equi | 3492 | 3344 | 5724 | 201 | 6207 |
| Corynebacterium propinquum | 0 | 0 | 0 | 0 | 0 |
| Corynebacterium striatum | 0 | 0 | 0 | 0 | 0 |
| Corynebacterium urealyticum | 749 | 775 | 1175 | 33 | 1281 |
| Tolumonas auensis | 71 | 158 | 241 | 5 | 163 |
| Caldicellulosiruptor saccharolyticus | 0 | 118 | 5 | 0 | 12 |
| Enterococcus dispar | 0 | 0 | 0 | 0 | 0 |
| Mycobacterium conspicuum | 2288 | 4196 | 3145 | 94 | 6472 |
| Ferrimonas balearica | 304 | 458 | 563 | 39 | 670 |
| Paenibacillus alvei | 58 | 301 | 181 | 0 | 359 |
| Paenibacillus durus | 191 | 798 | 481 | 18 | 708 |
| Paenibacillus macerans | 0 | 0 | 0 | 0 | 0 |
| Bradyrhizobium oligotrophicum | 4895 | 3658 | 3001 | 0 | 3425 |
| Borrelia recurrentis | 3 | 0 | 0 | 0 | 0 |
| Nitrosomonas ureae | 191 | 508 | 262 | 11 | 420 |
| Sulfurospirillum barnesii | 3 | 11 | 8 | 0 | 14 |
| Desulfovibrio fairfieldensis | 303 | 477 | 579 | 4 | 741 |
| Microcystis viridis | 12 | 25 | 49 | 0 | 115 |
| Halomonas venusta | 62 | 245 | 181 | 10 | 156 |
| Legionella adelaidensis | 17 | 28 | 52 | 5 | 35 |
| Legionella geestiana | 57 | 168 | 129 | 0 | 192 |
| Legionella lansingensis | 28 | 67 | 57 | 3 | 46 |
| Capnocytophaga haemolytica | 61 | 67 | 54 | 0 | 139 |
| Mesomycoplasma conjunctivae | 1 | 1 | 2 | 0 | 2 |
| Metamycoplasma alkalescens | 6 | 42 | 10 | 0 | 12 |
| Streptomyces griseoviridis | 2761 | 1906 | 3986 | 169 | 5913 |
| Schnuerera ultunensis | 538 | 34 | 97 | 49 | 66 |
| Helicobacter cholecystus | 0 | 7 | 5 | 0 | 1 |
| Psychrobacter urativorans | 0 | 0 | 0 | 0 | 0 |
| Desulfonema magnum | 170 | 459 | 304 | 1 | 511 |
| Desulfonema limicola | 34 | 93 | 104 | 7 | 378 |
| Vibrio scophthalmi | 18 | 47 | 49 | 10 | 49 |
| Desulfohalobium retbaense | 149 | 389 | 333 | 0 | 412 |
| Butyrivibrio crossotus | 2 | 19 | 19 | 0 | 18 |
| Granulicatella adiacens | 0 | 0 | 0 | 0 | 0 |
| Staphylococcus chromogenes | 12 | 15 | 27 | 1 | 9 |
| Staphylococcus felis | 21 | 6 | 8 | 0 | 7 |
| Actinomadura verrucosospora | 6344 | 5034 | 11204 | 1703 | 30211 |
| [Ruminococcus] lactaris | 59 | 30 | 28 | 0 | 21 |
| Anabaena sp. 90 | 179 | 541 | 336 | 0 | 446 |
| Weissella hellenica | 8 | 6 | 1 | 3 | 3 |
| Pseudomonas avellanae | 296 | 371 | 403 | 0 | 564 |
| Mycolicibacterium hassiacum | 3130 | 8285 | 6557 | 109 | 14504 |
| Thermoanaerobacter wiegelii | 14 | 0 | 0 | 0 | 3 |
| Spirochaeta africana | 174 | 276 | 281 | 1 | 298 |
| Sphingobium chlorophenolicum | 871 | 673 | 461 | 0 | 531 |
| Parabacteroides merdae | 161 | 141 | 172 | 12 | 204 |
| Bacteroides stercoris | 106 | 126 | 184 | 0 | 170 |
| Thermosipho melanesiensis | 12 | 19 | 31 | 0 | 47 |
| Pseudomonas agarici | 0 | 0 | 0 | 0 | 0 |
| Pseudomonas nitroreducens | 273 | 382 | 480 | 0 | 485 |
| Clostridium chauvoei | 20 | 14 | 10 | 0 | 7 |
| Tsukamurella pulmonis | 1821 | 1828 | 2400 | 86 | 3354 |
| Hydrogenophaga pseudoflava | 940 | 1509 | 1646 | 5 | 2304 |
| Borrelia miyamotoi | 9 | 16 | 5 | 0 | 12 |
| Limosilactobacillus panis | 0 | 0 | 0 | 0 | 0 |
| Rickettsia peacockii | 0 | 3 | 4 | 0 | 0 |
| Lautropia mirabilis | 574 | 883 | 1127 | 4 | 1216 |
| Bacteroides caccae | 7 | 79 | 23 | 0 | 70 |
| Lacticaseibacillus rhamnosus | 27 | 199 | 78 | 3 | 80 |
| Streptomyces olivaceus | 2047 | 1550 | 3448 | 291 | 7270 |
| Bibersteinia trehalosi | 0 | 2375 | 725 | 0 | 1166 |
| Streptomyces tubercidicus | 1144 | 1180 | 2138 | 80 | 3178 |
| Streptomyces aurantiacus | 5396 | 3778 | 8354 | 824 | 14351 |
| Streptomyces lydicus | 5941 | 5406 | 10385 | 992 | 18709 |
| Spiroplasma kunkelii | 0 | 0 | 0 | 0 | 3 |
| Spiroplasma phoeniceum | 9 | 0 | 15 | 0 | 0 |
| Micromonospora aurantiaca | 6406 | 4990 | 13466 | 611 | 20671 |
| Micromonospora carbonacea | 3703 | 2978 | 8227 | 364 | 11553 |
| Micromonospora echinaurantiaca | 4206 | 3016 | 9264 | 257 | 9322 |
| Micromonospora echinofusca | 3424 | 2796 | 7927 | 535 | 9872 |
| Micromonospora inositola | 4153 | 3071 | 9106 | 323 | 10070 |
| Micromonospora purpureochromogenes | 4061 | 2495 | 8976 | 244 | 7886 |
| Micromonospora rosaria | 0 | 0 | 0 | 0 | 4 |
| Micromonospora sagamiensis | 2441 | 1790 | 5868 | 305 | 7037 |
| Pseudomonas amygdali | 553 | 838 | 611 | 1 | 884 |
| Pseudomonas azotoformans | 249 | 526 | 763 | 0 | 1963 |
| Pseudomonas corrugata | 190 | 395 | 445 | 15 | 652 |
| Pseudomonas fulva | 2115 | 2411 | 2716 | 5 | 4607 |
| Pseudomonas synxantha | 404 | 687 | 348 | 0 | 29268 |
| Pseudomonas taetrolens | 138 | 283 | 317 | 1 | 394 |
| Pseudomonas oryzihabitans | 555 | 1022 | 1207 | 0 | 1315 |
| Serratia fonticola | 210 | 558 | 382 | 8 | 555 |
| Vibrio cyclitrophicus | 80 | 97 | 149 | 6 | 199 |
| Guyparkeria halophila | 1166 | 1652 | 1691 | 5 | 1689 |
| Dactylosporangium roseum | 3085 | 2917 | 7132 | 3159 | 45855 |
| Mycoplasmopsis pullorum | 0 | 0 | 0 | 0 | 0 |
| Microbulbifer hydrolyticus | 350 | 438 | 441 | 15 | 532 |
| Marinobacterium georgiense | 0 | 0 | 0 | 0 | 0 |
| Xanthomonas fragariae | 627 | 737 | 571 | 31 | 578 |
| Novosphingobium aromaticivorans | 718 | 920 | 589 | 0 | 486 |
| Candidatus Arthromitus sp. SFB-mouse | 10 | 1 | 0 | 0 | 20 |
| Paenibacillus thiaminolyticus | 356 | 733 | 683 | 11 | 1060 |
| Rubrobacter xylanophilus | 3355 | 4701 | 6096 | 117 | 8015 |
| Desulfitobacterium hafniense | 63 | 193 | 144 | 10 | 568 |
| Sporosarcina sp. | 0 | 1 | 0 | 0 | 0 |
| Mycoplasma crocodyli | 2 | 5 | 10 | 0 | 3 |
| Rhizobium sullae | 1370 | 1745 | 1388 | 48 | 1622 |
| Pseudomonas fuscovaginae | 0 | 0 | 0 | 0 | 0 |
| Vibrio diabolicus | 0 | 0 | 0 | 0 | 0 |
| Marinobacter sp. | 0 | 0 | 0 | 0 | 0 |
| Actinobacillus indolicus | 3 | 19 | 10 | 0 | 14 |
| Brevibacillus agri | 315 | 1002 | 674 | 4 | 850 |
| Actinobacillus delphinicola | 0 | 3 | 3 | 0 | 6 |
| Ureibacillus thermosphaericus | 0 | 0 | 0 | 0 | 0 |
| Syntrophobotulus glycolicus | 68 | 95 | 105 | 0 | 116 |
| Streptomyces griseocarneus | 3124 | 2197 | 4119 | 258 | 6521 |
| Wigglesworthia glossinidia | 18 | 11 | 21 | 0 | 28 |
| Kluyvera ascorbata | 292 | 299 | 255 | 18 | 276 |
| Mycoplasma cottewii | 41 | 3 | 10 | 0 | 7 |
| Mycoplasma yeatsii | 0 | 0 | 0 | 0 | 9 |
| Salinivibrio costicola | 12 | 26 | 51 | 0 | 53 |
| Dehalobacterium formicoaceticum | 0 | 0 | 0 | 0 | 0 |
| Pediococcus damnosus | 18 | 24 | 10 | 0 | 13 |
| Lapidilactobacillus dextrinicus | 0 | 8 | 5 | 0 | 3 |
| Tetragenococcus halophilus | 1 | 26 | 13 | 0 | 41 |
| Allomeiothermus silvanus | 466 | 1135 | 1035 | 5 | 1167 |
| Acinetobacter venetianus | 1 | 15 | 19 | 10 | 8 |
| Prevotella dentalis | 204 | 467 | 363 | 2 | 426 |
| Streptomyces flavovirens | 1 | 2 | 8 | 0 | 34 |
| Vibrio tapetis | 32 | 58 | 51 | 1 | 229 |
| Brachyspira pilosicoli | 24 | 12 | 7 | 0 | 14 |
| Acetobacterium wieringae | 28 | 175 | 134 | 0 | 154 |
| Bartonella alsatica | 4 | 9 | 11 | 0 | 12 |
| Actinomyces howellii | 655 | 719 | 1110 | 59 | 1546 |
| Schaalia meyeri | 0 | 0 | 0 | 0 | 0 |
| Actinomyces slackii | 366 | 596 | 775 | 35 | 1380 |
| Tatumella citrea | 37 | 121 | 52 | 0 | 55 |
| Staphylococcus delphini | 0 | 0 | 0 | 0 | 0 |
| Enterococcus durans | 34 | 188 | 32 | 0 | 62 |
| Intrasporangium calvum | 2405 | 2530 | 4216 | 79 | 4149 |
| Dactylosporangium fulvum | 6479 | 4119 | 12128 | 806 | 17721 |
| Dactylosporangium matsuzakiense | 3423 | 3232 | 8181 | 351 | 9513 |
| Dactylosporangium vinaceum | 4801 | 3863 | 9642 | 352 | 8871 |
| Mycobacterium heidelbergense | 1974 | 2848 | 2145 | 48 | 4047 |
| Hyphomicrobium denitrificans | 8655 | 4314 | 4203 | 31 | 4038 |
| Pseudomonas asplenii | 215 | 270 | 226 | 0 | 384 |
| Pseudomonas citronellolis | 2002 | 2783 | 2930 | 153 | 5008 |
| Pseudomonas coronafaciens | 52 | 110 | 230 | 10 | 621 |
| Pseudomonas ficuserectae | 94 | 64 | 104 | 0 | 114 |
| Pseudomonas resinovorans | 458 | 800 | 692 | 0 | 696 |
| Treponema paraluiscuniculi | 6 | 3 | 4 | 0 | 9 |
| Nocardiopsis alba | 1281 | 1198 | 2290 | 296 | 6693 |
| Eubacterium callanderi | 40 | 119 | 97 | 0 | 75 |
| Fructilactobacillus lindneri | 38 | 14 | 4 | 0 | 11 |
| Streptomyces rutgersensis | 1303 | 1385 | 3055 | 228 | 5208 |
| Nakamurella multipartita | 2823 | 2871 | 5329 | 291 | 6157 |
| Mycolicibacterium mageritense | 3749 | 10803 | 6980 | 162 | 17427 |
| Thalassobius gelatinovorus | 0 | 0 | 0 | 0 | 0 |
| Thermobifida alba | 2132 | 1936 | 3403 | 523 | 9546 |
| Mycoplasmopsis edwardii | 0 | 0 | 44 | 0 | 39 |
| Acidimicrobium ferrooxidans | 857 | 1278 | 2182 | 25 | 2113 |
| Octadecabacter arcticus | 215 | 261 | 210 | 3 | 319 |
| Peptoniphilus ivorii | 126 | 152 | 249 | 3 | 224 |
| Raoultella ornithinolytica | 541 | 1182 | 1139 | 64 | 1455 |
| Limnospira fusiformis | 0 | 0 | 14 | 0 | 0 |
| Chroococcidiopsis thermalis | 0 | 0 | 0 | 0 | 0 |
| Streptomyces venezuelae | 13331 | 12933 | 23333 | 1635 | 40741 |
| Salmonella bongori | 136 | 2947 | 89 | 769 | 144 |
| Brevibacillus choshinensis | 0 | 0 | 0 | 0 | 0 |
| Brevibacillus formosus | 25 | 99 | 107 | 0 | 105 |
| Brevibacillus parabrevis | 248 | 478 | 411 | 1 | 734 |
| Deinococcus proteolyticus | 416 | 769 | 841 | 1 | 1172 |
| Flavobacterium branchiophilum | 42 | 73 | 21 | 1 | 32 |
| Caldicellulosiruptor owensensis | 0 | 0 | 0 | 0 | 0 |
| Sediminispirochaeta smaragdinae | 52 | 105 | 83 | 0 | 139 |
| Pectobacterium wasabiae | 85 | 179 | 93 | 0 | 65 |
| Pantoea cypripedii | 107 | 144 | 143 | 36 | 220 |
| Erwinia persicina | 100 | 408 | 719 | 18 | 374 |
| Erwinia rhapontici | 136 | 339 | 249 | 8 | 551 |
| Brenneria rubrifaciens | 163 | 199 | 253 | 0 | 368 |
| Bradyrhizobium sp. NC92 | 2263 | 2105 | 1568 | 62 | 1825 |
| Janthinobacterium agaricidamnosum | 1132 | 2198 | 1869 | 28 | 11397 |
| Magnetospirillum gryphiswaldense | 1471 | 1643 | 1336 | 17 | 2598 |
| Dehalobacter restrictus | 44 | 292 | 75 | 0 | 150 |
| Vibrio anguillarum | 121 | 551 | 198 | 4 | 227 |
| Mycoplasmopsis caviae | 1 | 5 | 1 | 0 | 11 |
| Moorella glycerini | 163 | 388 | 309 | 14 | 516 |
| Sphingobium chungbukense | 0 | 0 | 0 | 0 | 0 |
| Bartonella clarridgeiae | 1 | 6 | 2 | 0 | 13 |
| Couchioplanes caeruleus | 5142 | 3689 | 10977 | 313 | 9460 |
| Xanthomonas arboricola | 1487 | 1739 | 1638 | 7 | 1915 |
| Xanthomonas cucurbitae | 567 | 811 | 674 | 5 | 810 |
| Xanthomonas hortorum | 1344 | 1523 | 1273 | 52 | 1537 |
| Xanthomonas hyacinthi | 928 | 891 | 970 | 10 | 1030 |
| Xanthomonas sacchari | 2996 | 3298 | 3825 | 127 | 4060 |
| Xanthomonas vasicola | 0 | 0 | 0 | 0 | 0 |
| Xanthomonas vesicatoria | 1213 | 1187 | 1299 | 0 | 908 |
| Xanthomonas theicola | 845 | 664 | 831 | 0 | 707 |
| Rhizobium gallicum | 1710 | 2463 | 1719 | 0 | 1928 |
| Psychrobacter sp. | 57 | 8 | 12 | 0 | 14 |
| Shewanella frigidimarina | 2 | 23 | 20 | 8 | 29 |
| Helicobacter bizzozeronii | 0 | 34 | 33 | 0 | 26 |
| Thermus oshimai | 0 | 0 | 0 | 0 | 0 |
| Psychroflexus torquis | 4 | 21 | 29 | 2 | 22 |
| Lacticaseibacillus zeae | 0 | 0 | 0 | 0 | 0 |
| Microbacterium esteraromaticum | 1211 | 1614 | 2625 | 66 | 2832 |
| Pseudodesulfovibrio profundus | 184 | 396 | 369 | 31 | 644 |
| Mycoplasma suis | 0 | 5 | 0 | 0 | 2 |
| Microvirgula aerodenitrificans | 711 | 1258 | 1235 | 41 | 1600 |
| Pseudothermotoga hypogea | 0 | 0 | 0 | 0 | 0 |
| Deinococcus radiopugnans | 323 | 821 | 699 | 0 | 973 |
| Tsukamurella tyrosinosolvens | 2468 | 2433 | 3390 | 87 | 4498 |
| Citrobacter braakii | 0 | 0 | 0 | 0 | 0 |
| Borreliella turdi | 0 | 1 | 0 | 0 | 0 |
| Burkholderia thailandensis | 3299 | 3039 | 5305 | 15 | 5935 |
| Colwellia sp. MT41 | 11 | 7 | 27 | 10 | 25 |
| Desulfofarcimen acetoxidans | 26 | 82 | 87 | 0 | 165 |
| Rahnella inusitata | 0 | 0 | 0 | 0 | 0 |
| Oleidesulfovibrio alaskensis | 281 | 708 | 817 | 4 | 767 |
| Streptomyces platensis | 1088 | 897 | 1511 | 238 | 3526 |
| Streptomyces thermocarboxydus | 1385 | 1234 | 2034 | 91 | 3311 |
| Streptococcus macedonicus | 0 | 0 | 0 | 0 | 0 |
| Thauera aromatica | 2408 | 3417 | 3950 | 63 | 6178 |
| Actinotignum schaalii | 0 | 0 | 0 | 0 | 0 |
| Planktothrix rubescens | 10 | 18 | 11 | 2 | 31 |
| Cellulophaga algicola | 15 | 22 | 19 | 0 | 24 |
| Desulforamulus reducens | 70 | 209 | 110 | 0 | 80 |
| Rathayibacter iranicus | 563 | 724 | 988 | 48 | 1285 |
| Candidatus Phytoplasma australiense | 0 | 9 | 22 | 0 | 12 |
| Alcanivorax borkumensis | 0 | 182 | 98 | 0 | 60 |
| Pantoea dispersa | 702 | 970 | 915 | 30 | 1150 |
| Paenibacillus glucanolyticus | 73 | 434 | 511 | 4 | 379 |
| Paenibacillus peoriae | 235 | 309 | 291 | 3 | 472 |
| Cyanobium gracile | 854 | 1184 | 1111 | 23 | 1771 |
| Synechococcus sp. WH 8101 | 101 | 367 | 252 | 1 | 350 |
| Citrifermentans bremense | 788 | 1806 | 1730 | 11 | 2059 |
| Sulfitobacter pontiacus | 0 | 0 | 0 | 0 | 0 |
| Shewanella violacea | 4 | 101 | 61 | 0 | 88 |
| Shewanella amazonensis | 55 | 100 | 141 | 1 | 272 |
| Shewanella sp. MR-4 | 61 | 31 | 31 | 0 | 42 |
| Latilactobacillus graminis | 0 | 32 | 0 | 0 | 6 |
| Lactiplantibacillus paraplantarum | 0 | 0 | 0 | 0 | 0 |
| Paraburkholderia graminis | 902 | 1363 | 1226 | 34 | 1539 |
| Burkholderia pyrrocinia | 0 | 0 | 0 | 0 | 0 |
| Burkholderia vietnamiensis | 0 | 0 | 0 | 0 | 0 |
| Phaeobacter gallaeciensis | 0 | 0 | 0 | 0 | 0 |
| Desulfobacca acetoxidans | 267 | 456 | 501 | 9 | 662 |
| Saccharopolyspora spinosa | 3619 | 1358 | 4605 | 299 | 5730 |
| Sanguibacter keddieii | 1497 | 1825 | 3122 | 57 | 3027 |
| Shewanella woodyi | 54 | 64 | 30 | 0 | 27 |
| Staphylococcus succinus | 0 | 0 | 0 | 0 | 0 |
| Dehalococcoides mccartyi | 140 | 321 | 286 | 0 | 452 |
| Paenibacillus mucilaginosus | 1039 | 1756 | 1736 | 46 | 2282 |
| Paracholeplasma brassicae | 22 | 39 | 29 | 0 | 59 |
| Enterobacter asburiae | 1463 | 1942 | 2125 | 21 | 1984 |
| Lelliottia amnigena | 0 | 0 | 0 | 0 | 0 |
| Pluralibacter gergoviae | 449 | 843 | 897 | 0 | 1216 |
| Kluyvera intermedia | 297 | 288 | 185 | 31 | 198 |
| Serratia ficaria | 214 | 148 | 183 | 0 | 168 |
| Serratia rubidaea | 603 | 524 | 507 | 1 | 889 |
| Borreliella valaisiana | 0 | 2 | 0 | 0 | 2 |
| Acidiphilium multivorum | 2466 | 2948 | 3335 | 12 | 3746 |
| Shewanella baltica | 210 | 365 | 348 | 1 | 387 |
| Zobellia galactanivorans | 32 | 87 | 52 | 1 | 67 |
| Aquifex aeolicus | 15 | 63 | 54 | 5 | 77 |
| Desulfovibrio sulfodismutans | 404 | 1027 | 1008 | 21 | 1200 |
| Sodalis glossinidius | 102 | 181 | 197 | 2 | 293 |
| Sideroxydans lithotrophicus | 565 | 956 | 836 | 31 | 1437 |
| Bacillus pseudomycoides | 81 | 134 | 49 | 0 | 64 |
| Desulfurobacterium thermolithotrophum | 2 | 44 | 35 | 1 | 45 |
| Synechococcus sp. CC9311 | 19 | 69 | 46 | 0 | 57 |
| Borreliella bissettiae | 0 | 11 | 9 | 0 | 0 |
| Corynebacterium ulcerans | 75 | 673 | 115 | 3 | 226 |
| Halothece sp. PCC 7418 | 66 | 164 | 208 | 0 | 213 |
| Mycoplasma wenyonii | 0 | 0 | 0 | 0 | 4 |
| Sulfurospirillum deleyianum | 1 | 19 | 17 | 2 | 6 |
| Desulfocapsa sulfexigens | 0 | 0 | 0 | 0 | 0 |
| Hydrogenophaga taeniospiralis | 634 | 1307 | 1036 | 0 | 1019 |
| Erwinia tracheiphila | 26 | 98 | 100 | 1 | 129 |
| Pseudomonas knackmussii | 2033 | 2540 | 2871 | 36 | 3099 |
| Komagataeibacter oboediens | 231 | 460 | 385 | 6 | 422 |
| Acetobacter pomorum | 61 | 77 | 14 | 0 | 155 |
| Lachnoclostridium phytofermentans | 10 | 77 | 21 | 0 | 11 |
| Pantoea stewartii | 139 | 278 | 189 | 1 | 265 |
| Streptomyces nojiriensis | 1607 | 1662 | 3289 | 159 | 5973 |
| Streptomyces luteoverticillatus | 2163 | 1817 | 3702 | 266 | 6569 |
| Sulfurospirillum multivorans | 8 | 39 | 108 | 0 | 112 |
| Streptomyces aureoverticillatus | 2518 | 2023 | 4355 | 409 | 7967 |
| Streptomyces gardneri | 2074 | 1840 | 3011 | 230 | 4171 |
| Legionella waltersii | 55 | 51 | 38 | 4 | 53 |
| Mycolicibacterium alvei | 1638 | 3999 | 2946 | 100 | 7516 |
| Streptomyces cavourensis | 1881 | 1848 | 2753 | 132 | 6059 |
| Streptomyces cinereoruber | 889 | 720 | 1576 | 45 | 2524 |
| Streptomyces alboflavus | 2169 | 2217 | 3909 | 151 | 4992 |
| Streptomyces calvus | 1631 | 1655 | 2712 | 210 | 4590 |
| Streptomyces cinnabarinus | 1859 | 1725 | 3381 | 313 | 5427 |
| Streptomyces filamentosus | 815 | 871 | 1304 | 139 | 3278 |
| Streptomyces finlayi | 1229 | 1284 | 2539 | 210 | 5520 |
| Streptomyces griseorubiginosus | 3347 | 2584 | 5370 | 214 | 6699 |
| Streptomyces hawaiiensis | 1528 | 1295 | 2627 | 148 | 3382 |
| Streptomyces lateritius | 47 | 29 | 117 | 1 | 135 |
| Streptomyces prasinus | 1557 | 1401 | 2587 | 320 | 6644 |
| Streptomyces californicus | 1566 | 1367 | 2648 | 197 | 4897 |
| Streptomyces tanashiensis | 1884 | 1889 | 3355 | 172 | 6303 |
| Streptomyces vinaceusdrappus | 946 | 860 | 1949 | 13 | 1553 |
| Streptomyces violascens | 148 | 704 | 173 | 0 | 1110 |
| Streptomyces xanthophaeus | 2019 | 1929 | 4167 | 317 | 9562 |
| Streptomyces viridosporus | 1695 | 1403 | 2444 | 107 | 3605 |
| Edwardsiella ictaluri | 93 | 241 | 159 | 1 | 196 |
| Citrobacter farmeri | 0 | 0 | 0 | 0 | 0 |
| Citrobacter rodentium | 712 | 767 | 756 | 30 | 725 |
| Citrobacter sedlakii | 31 | 236 | 189 | 0 | 92 |
| Streptomyces libani | 868 | 839 | 1140 | 41 | 1740 |
| Kitasatospora albolonga | 1316 | 1133 | 2001 | 301 | 4622 |
| Streptomyces antimycoticus | 1879 | 1195 | 2327 | 130 | 3006 |
| Streptomyces cyaneogriseus | 1631 | 1378 | 2197 | 148 | 3893 |
| Streptomyces fungicidicus | 589 | 834 | 1713 | 103 | 2446 |
| Streptomyces globosus | 1944 | 2291 | 3972 | 250 | 6657 |
| Streptomyces griseochromogenes | 2319 | 1671 | 3700 | 308 | 9110 |
| Streptomyces katrae | 2264 | 1873 | 3615 | 141 | 6495 |
| Streptomyces lusitanus | 3851 | 3352 | 6189 | 285 | 9037 |
| Streptomyces mirabilis | 1640 | 2501 | 5369 | 283 | 14205 |
| Streptomyces natalensis | 0 | 1 | 2 | 0 | 2 |
| Streptomyces olivoreticuli | 3064 | 2000 | 4300 | 272 | 7110 |
| Streptomyces pactum | 1774 | 1268 | 2751 | 142 | 3387 |
| Streptomyces spectabilis | 3872 | 3075 | 5866 | 355 | 9784 |
| Streptomyces tuirus | 843 | 1215 | 2048 | 157 | 2986 |
| Streptomyces violaceusniger | 2291 | 1182 | 2978 | 382 | 6476 |
| Cupriavidus basilensis | 5357 | 6587 | 7036 | 31 | 10101 |
| Enterobacter cancerogenus | 0 | 0 | 0 | 0 | 0 |
| Pectobacterium cacticida | 79 | 92 | 124 | 2 | 348 |
| Musicola paradisiaca | 154 | 277 | 239 | 9 | 251 |
| Pseudomonas sp. VLB120 | 235 | 798 | 188 | 0 | 212 |
| Microbacterium schleiferi | 568 | 1513 | 1624 | 57 | 1351 |
| Caulobacter henricii | 0 | 0 | 0 | 0 | 0 |
| Petrotoga mobilis | 15 | 32 | 27 | 0 | 39 |
| Moritella yayanosii | 5 | 91 | 29 | 2 | 54 |
| Caulobacter mirabilis | 1959 | 3151 | 2206 | 34 | 2751 |
| Selenomonas sputigena | 395 | 1079 | 1115 | 2 | 1591 |
| Candidatus Phytoplasma solani | 0 | 1 | 3 | 0 | 8 |
| Azotobacter salinestris | 529 | 1070 | 1151 | 42 | 1484 |
| Macrococcus caseolyticus | 5 | 60 | 44 | 0 | 90 |
| Macrococcus equipercicus | 32 | 90 | 71 | 1 | 101 |
| Staphylococcus condimenti | 8 | 23 | 0 | 0 | 7 |
| Staphylococcus piscifermentans | 0 | 5 | 4 | 0 | 1 |
| Acinetobacter variabilis | 0 | 0 | 0 | 0 | 0 |
| Acinetobacter dispersus | 0 | 0 | 0 | 0 | 0 |
| Pseudomonas plecoglossicida | 537 | 649 | 726 | 0 | 874 |
| Nodularia spumigena | 48 | 200 | 136 | 0 | 257 |
| Shewanella oneidensis | 18 | 85 | 46 | 0 | 45 |
| Shewanella pealeana | 25 | 36 | 46 | 12 | 52 |
| Limnothrix rosea | 0 | 7 | 3 | 0 | 13 |
| Mammaliicoccus vitulinus | 0 | 0 | 0 | 0 | 0 |
| Mesorhizobium amorphae | 2690 | 2276 | 1927 | 44 | 2261 |
| Enterococcus raffinosus | 27 | 41 | 61 | 0 | 70 |
| Waddlia chondrophila | 80 | 99 | 72 | 1 | 106 |
| Bacillus mojavensis | 8 | 69 | 43 | 0 | 101 |
| Bacillus vallismortis | 9 | 163 | 79 | 1 | 87 |
| Achromobacter ruhlandii | 447 | 729 | 804 | 0 | 1569 |
| Aeromonas encheleia | 0 | 0 | 0 | 0 | 0 |
| Streptomyces seoulensis | 3425 | 2624 | 4475 | 234 | 7522 |
| Thiocystis violascens | 977 | 1843 | 1515 | 54 | 1742 |
| Thermaerobacter marianensis | 1084 | 1800 | 2299 | 54 | 2636 |
| Roseovarius tolerans | 598 | 529 | 665 | 15 | 813 |
| Antarctobacter heliothermus | 0 | 0 | 0 | 0 | 0 |
| Photobacterium profundum | 7 | 70 | 28 | 9 | 21 |
| Brevundimonas subvibrioides | 819 | 1653 | 1373 | 52 | 1620 |
| Maricaulis maris | 662 | 642 | 621 | 12 | 963 |
| Brevundimonas mediterranea | 815 | 831 | 703 | 3 | 810 |
| Sulfitobacter guttiformis | 1 | 2 | 2 | 0 | 0 |
| Collinsella aerofaciens | 616 | 1010 | 1259 | 45 | 1231 |
| Entomoplasma freundtii | 0 | 0 | 6 | 0 | 4 |
| Staphylococcus coagulans | 0 | 2 | 2 | 0 | 0 |
| Stutzerimonas balearica | 0 | 0 | 0 | 0 | 0 |
| Paraburkholderia caribensis | 1935 | 2678 | 2569 | 31 | 4059 |
| Pseudonocardia alaniniphila | 227 | 193 | 467 | 0 | 418 |
| Streptomyces autolyticus | 1419 | 735 | 1991 | 215 | 1982 |
| Saccharomonospora xinjiangensis | 3370 | 1510 | 3884 | 119 | 4528 |
| Micropruina glycogenica | 1365 | 1917 | 2709 | 0 | 3048 |
| Pseudomonas mandelii | 0 | 0 | 0 | 0 | 0 |
| Campylobacter lanienae | 0 | 23 | 3 | 1 | 7 |
| Castellaniella defragrans | 2326 | 1741 | 2739 | 0 | 2597 |
| Thermocrinis ruber | 15 | 98 | 22 | 0 | 50 |
| Mannheimia haemolytica | 42 | 62 | 47 | 12 | 62 |
| Aromatoleum bremense | 1795 | 2427 | 2742 | 62 | 3681 |
| Aromatoleum petrolei | 2129 | 2419 | 2632 | 36 | 3821 |
| Hoylesella enoeca | 31 | 84 | 45 | 9 | 222 |
| Vibrio rumoiensis | 0 | 0 | 0 | 0 | 0 |
| Ferribacterium limneticum | 1774 | 2344 | 2139 | 37 | 3843 |
| Nostoc sp. ATCC 53789 | 215 | 906 | 472 | 29 | 766 |
| Campylobacter hominis | 0 | 0 | 0 | 0 | 0 |
| Cellulophaga baltica | 0 | 109 | 23 | 0 | 20 |
| Mycoplasmopsis gallopavonis | 0 | 0 | 6 | 0 | 3 |
| Pseudomonas orientalis | 736 | 1177 | 1740 | 7 | 2196 |
| Pseudomonas veronii | 0 | 0 | 0 | 0 | 0 |
| Myroides odoratimimus | 0 | 0 | 0 | 0 | 0 |
| Solibacillus silvestris | 0 | 0 | 0 | 0 | 0 |
| Streptococcus constellatus | 0 | 0 | 0 | 0 | 0 |
| Cellulomonas iranensis | 1667 | 1842 | 2921 | 123 | 3190 |
| Pseudomonas sp. K-62 | 1 | 28 | 24 | 0 | 19 |
| Helicobacter typhlonius | 39 | 34 | 18 | 0 | 20 |
| Sphingobium herbicidovorans | 589 | 732 | 765 | 0 | 658 |
| Cylindrospermopsis raciborskii | 1 | 22 | 42 | 0 | 49 |
| Wolbachia endosymbiont of Drosophila simulans | 0 | 2 | 3 | 0 | 7 |
| Prevotella bryantii | 8 | 18 | 27 | 0 | 21 |
| Wolbachia endosymbiont of Onchocerca volvulus | 0 | 1 | 13 | 0 | 7 |
| Pseudoalteromonas distincta | 13 | 8 | 6 | 0 | 6 |
| Bifidobacterium subtile | 271 | 489 | 489 | 37 | 796 |
| Parascardovia denticolens | 101 | 283 | 240 | 24 | 584 |
| Pseudomonas mosselii | 0 | 0 | 0 | 0 | 0 |
| Mycobacterium canettii | 1880 | 11311 | 4971 | 1143 | 7180 |
| Pectobacterium odoriferum | 0 | 0 | 0 | 0 | 0 |
| Bifidobacterium pullorum | 492 | 524 | 654 | 54 | 942 |
| Pseudomonas migulae | 166 | 194 | 179 | 222 | 321 |
| Asticcacaulis excentricus | 0 | 0 | 0 | 0 | 0 |
| Desulfosporosinus meridiei | 20 | 64 | 45 | 1 | 652 |
| Paenibacillus chitinolyticus | 226 | 568 | 393 | 2 | 672 |
| Denitrobacterium detoxificans | 287 | 663 | 520 | 0 | 542 |
| Alkalihalobacillus clausii | 0 | 0 | 0 | 0 | 0 |
| Sutcliffiella horikoshii | 36 | 68 | 60 | 0 | 62 |
| Alkalihalophilus pseudofirmus | 0 | 0 | 0 | 0 | 0 |
| Erwinia pyrifoliae | 13 | 47 | 60 | 6 | 248 |
| Thioflavicoccus mobilis | 1590 | 2113 | 2267 | 66 | 2598 |
| Haliangium ochraceum | 5193 | 8058 | 8636 | 107 | 9279 |
| Herbaspirillum rubrisubalbicans | 1213 | 1135 | 1250 | 3 | 2300 |
| Wolbachia endosymbiont of Litomosoides sigmodontis | 0 | 3 | 4 | 0 | 0 |
| Streptomyces cyanogenus | 1971 | 1245 | 2516 | 224 | 4215 |
| Acidovorax avenae | 2777 | 4123 | 3992 | 64 | 5034 |
| Acidovorax cattleyae | 377 | 689 | 757 | 10 | 975 |
| Acidovorax citrulli | 755 | 1047 | 1172 | 2 | 2553 |
| Hylemonella gracilis | 502 | 723 | 611 | 0 | 718 |
| Treponema brennaborense | 129 | 388 | 207 | 0 | 354 |
| Hyphomonas neptunium | 534 | 600 | 576 | 36 | 904 |
| Parageobacillus caldoxylosilyticus | 19 | 167 | 121 | 4 | 160 |
| Aminomonas paucivorans | 0 | 0 | 0 | 0 | 0 |
| Entomoplasma melaleucae | 0 | 1 | 0 | 0 | 6 |
| Thermanaerovibrio acidaminovorans | 270 | 421 | 454 | 6 | 555 |
| Aminobacterium colombiense | 42 | 82 | 127 | 1 | 231 |
| Frateuria aurantia | 498 | 724 | 729 | 12 | 656 |
| Fannyhessea vaginae | 0 | 0 | 0 | 0 | 0 |
| Streptococcus pluranimalium | 24 | 25 | 7 | 0 | 22 |
| Paracoccus pantotrophus | 0 | 0 | 0 | 0 | 0 |
| Microbacterium oxydans | 2084 | 2503 | 4501 | 147 | 4281 |
| Cupriavidus gilardii | 2106 | 2842 | 3164 | 15 | 4525 |
| Cupriavidus pauculus | 6127 | 8150 | 9021 | 104 | 12657 |
| Pseudanabaena sp. PCC 7367 | 39 | 201 | 133 | 0 | 177 |
| Liquorilactobacillus nagelii | 13 | 21 | 42 | 0 | 56 |
| Buttiauxella agrestis | 0 | 0 | 0 | 0 | 0 |
| Obesumbacterium proteus | 24 | 61 | 55 | 5 | 72 |
| Pragia fontium | 41 | 110 | 91 | 9 | 101 |
| Tatumella ptyseos | 0 | 0 | 0 | 0 | 0 |
| Buttiauxella ferragutiae | 116 | 211 | 54 | 8 | 6688 |
| Serratia grimesii | 58 | 137 | 84 | 0 | 127 |
| Serratia plymuthica | 0 | 0 | 0 | 0 | 0 |
| Sulfitobacter mediterraneus | 0 | 0 | 0 | 0 | 0 |
| Aminobacter aminovorans | 1107 | 1063 | 1014 | 42 | 941 |
| Aminobacter niigataensis | 217 | 84 | 115 | 0 | 110 |
| Streptomyces armeniacus | 3149 | 2577 | 4493 | 330 | 7170 |
| Archangium violaceum | 6164 | 12184 | 13704 | 214 | 15745 |
| Melittangium boletus | 2521 | 4426 | 4616 | 32 | 5631 |
| Myxococcus stipitatus | 1382 | 2973 | 2564 | 52 | 3661 |
| Companilactobacillus paralimentarius | 1 | 1 | 3 | 0 | 1 |
| Chlamydia psittaci | 8 | 61 | 56 | 0 | 81 |
| Chlamydia abortus | 3 | 22 | 5 | 0 | 1 |
| Chlamydia felis | 0 | 6 | 4 | 0 | 5 |
| Chlamydia caviae | 9 | 2 | 19 | 13 | 9 |
| Chlamydia pneumoniae | 0 | 23 | 3 | 0 | 8 |
| Chlamydia suis | 47 | 23 | 16 | 0 | 24 |
| Chlamydia muridarum | 1 | 16 | 10 | 0 | 9 |
| Simkania negevensis | 73 | 68 | 79 | 0 | 87 |
| Desulfosarcina ovata | 692 | 1268 | 1123 | 6 | 1432 |
| Bradyrhizobium genosp. B | 3149 | 2493 | 1822 | 0 | 1442 |
| Bradyrhizobium genosp. L | 6416 | 5709 | 4150 | 14 | 4255 |
| Leclercia adecarboxylata | 646 | 1437 | 1090 | 0 | 385 |
| Streptomyces tsukubensis | 3583 | 3145 | 5376 | 499 | 12571 |
| Lactobacillus amylolyticus | 0 | 0 | 0 | 0 | 0 |
| Succinivibrio dextrinosolvens | 35 | 74 | 31 | 0 | 61 |
| Clostridium aceticum | 15 | 23 | 18 | 0 | 31 |
| Clostridium autoethanogenum | 0 | 4 | 4 | 0 | 7 |
| Lacrimispora saccharolytica | 22 | 35 | 52 | 0 | 123 |
| Pseudoclostridium thermosuccinogenes | 34 | 69 | 70 | 1 | 51 |
| Gordonia alkanivorans | 1183 | 1114 | 1796 | 192 | 2721 |
| Slackia heliotrinireducens | 0 | 0 | 0 | 0 | 0 |
| Eggerthella lenta | 773 | 1464 | 1760 | 22 | 1649 |
| Gemella sanguinis | 0 | 0 | 0 | 0 | 0 |
| Magnetospirillum magneticum | 1885 | 1621 | 1694 | 30 | 2514 |
| Cryptobacterium curtum | 13 | 50 | 47 | 0 | 113 |
| Microbacterium chocolatum | 699 | 1458 | 2049 | 31 | 2015 |
| Brachyspira intermedia | 6 | 9 | 26 | 0 | 13 |
| Brachyspira murdochii | 0 | 0 | 0 | 0 | 0 |
| Hippea maritima | 32 | 30 | 76 | 0 | 50 |
| Lysobacter antibioticus | 2322 | 2606 | 2503 | 27 | 1855 |
| Taylorella asinigenitalis | 0 | 5 | 19 | 0 | 48 |
| Gordonia polyisoprenivorans | 2604 | 3089 | 4274 | 134 | 5571 |
| Beutenbergia cavernae | 1960 | 2227 | 3465 | 90 | 3163 |
| Desulfotalea psychrophila | 14 | 92 | 82 | 8 | 174 |
| Pseudarthrobacter chlorophenolicus | 236 | 800 | 1343 | 67 | 2538 |
| Rothia nasimurium | 0 | 0 | 0 | 0 | 0 |
| Mannheimia granulomatis | 9 | 40 | 43 | 5 | 59 |
| Thauera sp. MZ1T | 4058 | 4493 | 5598 | 35 | 8191 |
| Salisediminibacterium selenitireducens | 84 | 156 | 244 | 0 | 387 |
| Mycolicibacterium monacense | 2905 | 8576 | 6383 | 10 | 13840 |
| Achromobacter xylosoxidans | 10210 | 11635 | 13528 | 244 | 17649 |
| Bartonella tribocorum | 42 | 19 | 16 | 0 | 30 |
| Thermacetogenium phaeum | 279 | 714 | 757 | 2 | 1096 |
| Mycolicibacterium brumae | 1734 | 3466 | 3194 | 58 | 5303 |
| Chlamydia pecorum | 0 | 17 | 11 | 0 | 17 |
| Idiomarina abyssalis | 11 | 53 | 55 | 1 | 82 |
| endosymbiont of Acanthamoeba sp. UWC8 | 2 | 16 | 35 | 0 | 36 |
| Syntrophothermus lipocalidus | 95 | 366 | 241 | 0 | 311 |
| Microbulbifer elongatus | 237 | 365 | 344 | 1 | 377 |
| Pseudomonas thivervalensis | 156 | 319 | 311 | 19 | 404 |
| Saccharophagus degradans | 39 | 72 | 45 | 3 | 77 |
| Mogibacterium pumilum | 0 | 0 | 0 | 0 | 0 |
| Mesomycoplasma dispar | 15 | 18 | 0 | 1 | 5 |
| Priestia flexa | 0 | 0 | 0 | 0 | 0 |
| Halalkalibacterium halodurans | 58 | 85 | 64 | 1 | 155 |
| Pseudomonas cannabina | 80 | 124 | 60 | 0 | 82 |
| Aerococcus christensenii | 4 | 42 | 30 | 3 | 39 |
| Pseudoalteromonas sp. PS1M3 | 21 | 16 | 6 | 27 | 13 |
| Burkholderia multivorans | 0 | 0 | 0 | 0 | 0 |
| Treponema primitia | 102 | 178 | 195 | 1 | 292 |
| Lacticaseibacillus manihotivorans | 19 | 72 | 80 | 0 | 137 |
| Dorea longicatena | 75 | 136 | 63 | 0 | 87 |
| Caulobacter segnis | 0 | 0 | 0 | 0 | 0 |
| Gordonia amicalis | 2074 | 1456 | 2659 | 0 | 2935 |
| Ligilactobacillus acidipiscis | 2 | 54 | 17 | 0 | 16 |
| Ignavigranum ruoffiae | 7 | 107 | 12 | 0 | 13 |
| Peptacetobacter hiranonis | 11 | 15 | 42 | 7 | 41 |
| [Clostridium] hylemonae | 106 | 191 | 171 | 0 | 360 |
| Ruegeria pomeroyi | 799 | 722 | 677 | 11 | 1034 |
| Neoehrlichia mikurensis | 1 | 13 | 5 | 0 | 19 |
| Aromatoleum toluolicum | 0 | 835 | 0 | 0 | 0 |
| Oligella ureolytica | 42 | 43 | 110 | 120 | 127 |
| Sphingomonas sp. A1 | 0 | 0 | 73 | 0 | 0 |
| Moritella marina | 22 | 14 | 22 | 1 | 17 |
| Candidatus Paracaedibacter acanthamoebae | 72 | 78 | 45 | 0 | 53 |
| Candidatus Portiera aleyrodidarum | 0 | 0 | 0 | 0 | 0 |
| Asaia bogorensis | 325 | 663 | 669 | 0 | 828 |
| Vibrio aerogenes | 29 | 81 | 117 | 1 | 76 |
| Thiomicrospira aerophila | 35 | 33 | 30 | 2 | 44 |
| Metamycoplasma anseris | 0 | 2 | 4 | 0 | 5 |
| Metamycoplasma cloacale | 1 | 1 | 8 | 0 | 0 |
| Streptomyces malaysiensis | 1457 | 1054 | 2171 | 18 | 2794 |
| Herbaspirillum frisingense | 1019 | 1314 | 1268 | 78 | 2391 |
| Paraburkholderia tropica | 1227 | 1590 | 1802 | 17 | 2588 |
| [Brevibacterium] flavum | 2124 | 0 | 0 | 0 | 1450 |
| Mycoplasmoides fastidiosum | 0 | 0 | 0 | 0 | 0 |
| Nostoc linckia | 98 | 526 | 222 | 7 | 293 |
| Ketogulonicigenium vulgare | 451 | 639 | 536 | 34 | 786 |
| Ketogulonicigenium robustum | 284 | 522 | 400 | 3 | 565 |
| Pandoraea apista | 0 | 0 | 0 | 0 | 0 |
| Pandoraea norimbergensis | 764 | 1071 | 1029 | 19 | 1193 |
| Pandoraea pnomenusa | 1994 | 3318 | 2970 | 0 | 3745 |
| Pandoraea pulmonicola | 823 | 1058 | 1239 | 0 | 1546 |
| Pandoraea sputorum | 569 | 872 | 869 | 40 | 1271 |
| Fervidobacterium pennivorans | 36 | 56 | 24 | 0 | 44 |
| Thermotoga petrophila | 1 | 50 | 28 | 0 | 123 |
| Nonomuraea gerenzanensis | 8590 | 5417 | 43844 | 7423 | 125494 |
| Shewanella japonica | 2 | 233 | 29 | 0 | 22 |
| Shewanella sp. ANA-3 | 83 | 325 | 270 | 94 | 217 |
| Ramlibacter tataouinensis | 6803 | 9700 | 9298 | 256 | 13853 |
| Staphylococcus ureilyticus | 0 | 1 | 0 | 0 | 0 |
| Dyadobacter fermentans | 399 | 712 | 679 | 25 | 1084 |
| Bordetella petrii | 3278 | 3484 | 4226 | 67 | 5749 |
| Clostridium gasigenes | 14 | 14 | 14 | 0 | 2 |
| Pseudomonas vancouverensis | 0 | 0 | 0 | 0 | 0 |
| Pseudomonas multiresinivorans | 0 | 0 | 0 | 0 | 0 |
| Burkholderia cenocepacia | 0 | 0 | 0 | 0 | 0 |
| Pseudomonas sp. M1 | 453 | 747 | 726 | 0 | 858 |
| Methylotuvimicrobium buryatense | 245 | 551 | 286 | 33 | 334 |
| Legionella fallonii | 38 | 58 | 48 | 2 | 58 |
| Legionella lytica | 24 | 38 | 20 | 2 | 36 |
| Bacillus spizizenii | 0 | 3 | 4 | 0 | 0 |
| Cupriavidus oxalaticus | 2518 | 2953 | 3581 | 55 | 4227 |
| Flavobacterium psychrophilum | 0 | 0 | 0 | 0 | 0 |
| Thiothrix winogradskyi | 127 | 200 | 187 | 19 | 198 |
| Wolbachia endosymbiont of Brugia pahangi | 0 | 1 | 0 | 0 | 1 |
| Thauera chlorobenzoica | 1166 | 1675 | 1972 | 7 | 2796 |
| Vitreoscilla sp. C1 | 0 | 6 | 42 | 0 | 135 |
| Halobacteriovorax marinus | 13 | 34 | 27 | 2 | 52 |
| Clostridium sp. MD294 | 2 | 13 | 8 | 0 | 22 |
| Acetomicrobium mobile | 73 | 86 | 85 | 0 | 93 |
| Limosilactobacillus mucosae | 13 | 151 | 92 | 0 | 80 |
| Pasteurella skyensis | 15 | 5 | 7 | 0 | 4 |
| Bacillus sp. OxB-1 | 207 | 206 | 134 | 0 | 312 |
| Mycolicibacterium septicum | 1418 | 3923 | 2613 | 106 | 7312 |
| Calothrix sp. PCC 7507 | 157 | 522 | 260 | 13 | 404 |
| Rhodococcus koreensis | 1759 | 2944 | 3203 | 107 | 4307 |
| Austwickia chelonae | 601 | 724 | 1081 | 45 | 1349 |
| Onion yellows phytoplasma | 3 | 0 | 0 | 0 | 0 |
| Chloroherpeton thalassium | 0 | 0 | 0 | 0 | 0 |
| Catenibacterium mitsuokai | 17 | 8 | 11 | 0 | 15 |
| Aequorivita sublithincola | 173 | 194 | 148 | 21 | 155 |
| Sweet potato little leaf phytoplasma | 0 | 0 | 1 | 1 | 0 |
| Candidatus Blochmannia pennsylvanicus | 0 | 0 | 0 | 0 | 10 |
| Burkholderia ubonensis | 1992 | 2658 | 4097 | 0 | 3580 |
| Stanieria cyanosphaera | 5 | 64 | 42 | 0 | 49 |
| Desulfoscipio gibsoniae | 145 | 323 | 265 | 2 | 361 |
| Streptococcus infantarius | 0 | 2 | 0 | 0 | 6 |
| Proteus penneri | 4 | 15 | 12 | 3 | 101 |
| Nostoc sp. PCC 7120 = FACHB-418 | 0 | 567 | 373 | 0 | 344 |
| Saccharothrix espanaensis | 31701 | 8372 | 17276 | 475 | 15483 |
| Saccharothrix syringae | 32918 | 9511 | 22711 | 585 | 16524 |
| Rhodococcus sp. AD45 | 20 | 20 | 23 | 0 | 57 |
| Rhodococcus pyridinivorans | 5159 | 4453 | 5828 | 245 | 7178 |
| Bordetella hinzii | 996 | 1436 | 1486 | 0 | 1486 |
| Pseudomonas frederiksbergensis | 1063 | 17338 | 4640 | 17 | 15869 |
| Acetobacter tropicalis | 265 | 91 | 72 | 0 | 121 |
| Tenacibaculum mesophilum | 1 | 6 | 12 | 2 | 14 |
| Tenacibaculum ovolyticum | 2 | 27 | 23 | 2 | 14 |
| Microbacterium foliorum | 1826 | 2316 | 3539 | 79 | 3921 |
| Vibrio penaeicida | 34 | 62 | 56 | 23 | 70 |
| Serratia sp. ATCC 39006 | 110 | 197 | 119 | 7 | 129 |
| Helicobacter suis | 1 | 12 | 14 | 0 | 16 |
| Chitinophaga filiformis | 0 | 0 | 0 | 0 | 0 |
| Limosilactobacillus frumenti | 9 | 20 | 41 | 0 | 60 |
| Ralstonia mannitolilytica | 1036 | 956 | 1097 | 0 | 0 |
| Methylibium petroleiphilum | 1056 | 1358 | 1263 | 0 | 1535 |
| Aeromonas bestiarum | 167 | 203 | 227 | 0 | 378 |
| Anaerostipes caccae | 30 | 50 | 56 | 0 | 112 |
| Frankia casuarinae | 1885 | 1828 | 3166 | 214 | 5631 |
| Thermoanaerobacter siderophilus | 3 | 0 | 0 | 5 | 19 |
| Cupriavidus necator | 4369 | 6460 | 5942 | 45 | 8913 |
| Ensifer adhaerens | 5467 | 5798 | 4567 | 44 | 5941 |
| Thioalkalivibrio versutus | 765 | 985 | 1024 | 0 | 1098 |
| Acinetobacter bereziniae | 0 | 0 | 0 | 0 | 0 |
| Pseudoalteromonas ulvae | 10 | 31 | 26 | 2 | 17 |
| Tenacibaculum maritimum | 11 | 19 | 12 | 6 | 14 |
| Pseudomonas sp. A2 | 0 | 0 | 0 | 0 | 0 |
| Methylomonas sp. LW13 | 0 | 0 | 0 | 0 | 0 |
| Opitutus terrae | 3079 | 6404 | 5065 | 104 | 5843 |
| Thermanaerovibrio velox | 55 | 204 | 240 | 0 | 259 |
| Thioalkalivibrio paradoxus | 1360 | 1788 | 1744 | 49 | 2015 |
| Bradyrhizobium yuanmingense | 0 | 0 | 1452 | 0 | 4279 |
| Candidatus Blochmannia herculeanus | 22 | 17 | 7 | 0 | 1 |
| Thermoanaerobacter italicus | 62 | 3 | 22 | 0 | 17 |
| Corynebacterium falsenii | 443 | 625 | 910 | 6 | 911 |
| Rickettsia monacensis | 0 | 4 | 1 | 0 | 1 |
| Anabaenopsis circularis | 101 | 1044 | 154 | 0 | 380 |
| Bisgaardia hudsonensis | 10 | 6 | 6 | 0 | 7 |
| Lactobacillus jensenii | 62 | 44 | 2 | 1 | 7 |
| Nocardioides sp. CF8 | 2455 | 5078 | 5970 | 145 | 7558 |
| Sinorhizobium medicae | 1734 | 1738 | 1605 | 0 | 2081 |
| Mycobacterium heckeshornense | 1450 | 4451 | 2976 | 61 | 6171 |
| Mycolicibacterium vanbaalenii | 939 | 12026 | 3462 | 0 | 11306 |
| Synechococcus sp. CC9605 | 68 | 80 | 87 | 0 | 106 |
| Rathayibacter festucae | 1970 | 2298 | 9083 | 63 | 3780 |
| Actinomyces radicidentis | 935 | 1204 | 1808 | 24 | 2045 |
| Muricauda ruestringensis | 28 | 77 | 48 | 0 | 113 |
| Bartonella birtlesii | 1 | 6 | 6 | 0 | 8 |
| Thiothrix unzii | 100 | 251 | 129 | 15 | 308 |
| Thiothrix fructosivorans | 11 | 31 | 44 | 0 | 46 |
| Leptolyngbya sp. PCC 7376 | 34 | 88 | 65 | 0 | 101 |
| Defluviicoccus vanus | 1792 | 2300 | 1809 | 0 | 2764 |
| Mannheimia pernigra | 7 | 40 | 0 | 0 | 0 |
| Chryseobacterium joostei | 10 | 24 | 46 | 0 | 40 |
| Bathymodiolus septemdierum thioautotrophic gill symbiont | 3 | 12 | 16 | 1 | 31 |
| Actinoplanes derwentensis | 5072 | 2890 | 7946 | 245 | 9034 |
| Pediococcus inopinatus | 39 | 13 | 20 | 0 | 23 |
| Candidatus Carsonella ruddii | 0 | 0 | 0 | 0 | 0 |
| Mogibacterium diversum | 5 | 13 | 16 | 1 | 35 |
| Bradyrhizobium sp. ORS 278 | 5670 | 3986 | 3143 | 45 | 3839 |
| Methylobacterium nodulans | 3803 | 3862 | 3572 | 75 | 5050 |
| Streptomyces phaeoluteigriseus | 1997 | 2231 | 4871 | 187 | 7435 |
| Streptomyces auratus | 839 | 722 | 1649 | 170 | 2929 |
| Mycoplasmopsis columbinasalis | 0 | 1 | 5 | 0 | 9 |
| Mycoplasmopsis columbina | 1 | 0 | 1 | 2 | 3 |
| Mycoplasmopsis equigenitalium | 12 | 16 | 8 | 0 | 11 |
| Mycoplasmopsis maculosa | 0 | 1 | 1 | 0 | 0 |
| Halomonas hydrothermalis | 377 | 479 | 358 | 15 | 591 |
| Leuconostoc gasicomitatum | 16 | 0 | 4 | 0 | 2 |
| Bradyrhizobium sp. ORS 285 | 5920 | 4098 | 3366 | 15 | 3919 |
| Coprococcus catus | 19 | 109 | 65 | 0 | 48 |
| Caloranaerobacter azorensis | 75 | 31 | 68 | 2 | 67 |
| Pseudoalteromonas sp. A25 | 3 | 85 | 15 | 2 | 14 |
| Streptomyces coeruleorubidus | 3751 | 3290 | 5088 | 246 | 8974 |
| Paenibacillus azoreducens | 112 | 325 | 195 | 6 | 505 |
| Actinopolymorpha singaporensis | 3539 | 3333 | 6128 | 384 | 9489 |
| Sphingopyxis alaskensis | 0 | 0 | 0 | 0 | 0 |
| Mycobacterium frederiksbergense | 2192 | 5824 | 3897 | 98 | 8447 |
| Denitrovibrio acetiphilus | 38 | 59 | 97 | 0 | 214 |
| Enterococcus rotai | 0 | 11 | 11 | 0 | 28 |
| Pleurocapsa sp. PCC 7327 | 78 | 375 | 169 | 0 | 223 |
| Oscillatoria acuminata | 0 | 0 | 0 | 0 | 0 |
| Arthrospira platensis | 0 | 11 | 46 | 0 | 13 |
| Pseudomonas sp. B10 | 228 | 142 | 91 | 26 | 223 |
| Aerococcus sanguinicola | 41 | 157 | 42 | 0 | 55 |
| Cupriavidus metallidurans | 2143 | 2413 | 3027 | 0 | 2197 |
| Pseudothermotoga thermarum | 4 | 26 | 28 | 0 | 28 |
| Syntrophobacter fumaroxidans | 781 | 1601 | 1647 | 21 | 2014 |
| Microcoleus vaginatus | 0 | 0 | 0 | 0 | 0 |
| Bacillus sonorensis | 0 | 0 | 0 | 0 | 8 |
| Marinomonas mediterranea | 48 | 38 | 77 | 15 | 37 |
| Propionibacterium australiense | 727 | 962 | 1169 | 87 | 1882 |
| Sphingobium cloacae | 1996 | 2688 | 2223 | 16 | 3515 |
| Brucella pinnipedialis | 0 | 52 | 98 | 0 | 261 |
| Brucella ceti | 14 | 144 | 623 | 0 | 146 |
| Treponema sp. OMZ 803 | 0 | 0 | 0 | 0 | 0 |
| Mycobacterium kubicae | 2257 | 4547 | 3154 | 97 | 6940 |
| Roseiflexus castenholzii | 710 | 1635 | 1469 | 42 | 1811 |
| Pseudarthrobacter sulfonivorans | 1691 | 7528 | 6692 | 163 | 10103 |
| Pannonibacter phragmitetus | 5416 | 4303 | 3885 | 0 | 5229 |
| Pseudomonas psychrophila | 0 | 0 | 0 | 0 | 0 |
| Actinoplanes ianthinogenes | 4664 | 3705 | 9253 | 207 | 8556 |
| Arthrobacter keyseri | 3 | 0 | 6 | 0 | 6 |
| Frederiksenia canicola | 0 | 17 | 17 | 9 | 11 |
| Helicobacter canadensis | 6 | 1 | 12 | 0 | 3 |
| Bordetella trematum | 1440 | 1640 | 1490 | 0 | 2228 |
| Wolbachia endosymbiont of Trichogramma pretiosum | 0 | 1 | 3 | 0 | 7 |
| Providencia alcalifaciens | 0 | 0 | 0 | 0 | 0 |
| Mycolicibacterium doricum | 0 | 3732 | 1194 | 0 | 2950 |
| Thermotoga sp. RQ7 | 0 | 13 | 18 | 0 | 39 |
| Thermotoga sp. RQ2 | 0 | 0 | 0 | 0 | 14 |
| Paenibacillus brasilensis | 0 | 0 | 0 | 0 | 0 |
| Stenotrophomonas acidaminiphila | 2535 | 3283 | 3577 | 0 | 714 |
| Pseudoxanthomonas mexicana | 0 | 0 | 0 | 0 | 0 |
| Aerococcus urinaehominis | 13 | 4 | 9 | 0 | 11 |
| Geobacillus subterraneus | 0 | 0 | 0 | 0 | 0 |
| Pseudomonas brenneri | 0 | 0 | 0 | 0 | 0 |
| Amycolatopsis keratiniphila | 17378 | 3502 | 10309 | 267 | 8219 |
| Carboxydothermus hydrogenoformans | 66 | 297 | 199 | 0 | 183 |
| Mesobacillus jeotgali | 77 | 130 | 196 | 1 | 267 |
| Paenibacillus dendritiformis | 521 | 1106 | 998 | 11 | 1495 |
| Schaalia turicensis | 279 | 286 | 435 | 13 | 349 |
| Arcanobacterium phocae | 28 | 88 | 59 | 0 | 117 |
| Kineococcus radiotolerans | 2168 | 2573 | 3494 | 266 | 6146 |
| Desulfomicrobium orale | 288 | 623 | 484 | 2 | 436 |
| Streptomyces alboniger | 1606 | 1520 | 2599 | 208 | 3885 |
| Planktothrix pseudagardhii | 0 | 0 | 0 | 0 | 0 |
| Rhodococcus jostii | 1289 | 2915 | 2357 | 94 | 3561 |
| Exiguobacterium antarcticum | 0 | 0 | 0 | 0 | 0 |
| Citrobacter youngae | 55 | 196 | 219 | 0 | 109 |
| Caldilinea aerophila | 756 | 2049 | 1620 | 22 | 2043 |
| Nitrosococcus halophilus | 109 | 341 | 253 | 0 | 255 |
| Mycobacterium shottsii | 0 | 0 | 0 | 0 | 0 |
| Leisingera methylohalidivorans | 354 | 519 | 415 | 9 | 498 |
| Corynebacterium freneyi | 0 | 0 | 0 | 0 | 0 |
| secondary endosymbiont of Heteropsylla cubana | 0 | 0 | 0 | 0 | 0 |
| Achromobacter sp. | 0 | 0 | 375 | 0 | 324 |
| Paraburkholderia caledonica | 1120 | 1650 | 1371 | 34 | 2100 |
| Mycolicibacterium goodii | 3011 | 7082 | 5016 | 121 | 12172 |
| Actinoplanes sp. SE50/110 | 8632 | 2965 | 10566 | 989 | 20707 |
| Ureaplasma parvum | 0 | 4 | 0 | 0 | 3 |
| Nocardia cyriacigeorgica | 4768 | 4046 | 8908 | 222 | 9172 |
| Helicobacter apodemus | 0 | 4 | 2 | 0 | 7 |
| Idiomarina loihiensis | 130 | 175 | 297 | 22 | 296 |
| Candidatus Phytoplasma ziziphi | 0 | 10 | 0 | 0 | 11 |
| Priestia endophytica | 3 | 0 | 3 | 0 | 6 |
| Paracoccus kondratievae | 0 | 0 | 0 | 0 | 0 |
| Paracoccus methylutens | 110 | 9 | 22 | 0 | 31 |
| Dolichospermum compactum | 113 | 209 | 178 | 0 | 355 |
| Thermocrinis albus | 25 | 28 | 52 | 0 | 41 |
| Mycoplasma haemocanis | 0 | 1 | 0 | 0 | 0 |
| Vibrio lentus | 0 | 22 | 8 | 0 | 21 |
| Leuconostoc kimchii | 0 | 0 | 0 | 0 | 0 |
| Serratia quinivorans | 134 | 243 | 194 | 2 | 214 |
| Thalassomonas viridans | 216 | 249 | 237 | 7 | 395 |
| Azospirillum sp. B510 | 1614 | 2158 | 2082 | 39 | 3546 |
| Pseudoalteromonas flavipulchra | 10 | 14 | 12 | 29 | 0 |
| Nodularia sphaerocarpa | 80 | 197 | 118 | 0 | 167 |
| Clostridium neonatale | 19 | 21 | 15 | 0 | 25 |
| Candidatus Hamiltonella defensa | 12 | 58 | 69 | 1 | 81 |
| Serratia symbiotica | 0 | 0 | 0 | 0 | 0 |
| Quatrionicoccus australiensis | 619 | 1282 | 1165 | 26 | 1534 |
| Blastococcus saxobsidens | 3387 | 3602 | 4932 | 118 | 6221 |
| Helicobacter cetorum | 7 | 16 | 17 | 0 | 37 |
| Mesoplasma lactucae | 0 | 1 | 1 | 0 | 1 |
| Dietzia psychralcaliphila | 0 | 0 | 0 | 0 | 0 |
| Isoptericola variabilis | 2383 | 2551 | 4001 | 111 | 3653 |
| Geovibrio thiophilus | 98 | 264 | 205 | 2 | 222 |
| Mycobacterium lentiflavum | 1765 | 3137 | 2212 | 54 | 4373 |
| Candidatus Mycoplasma haemolamae | 9 | 1 | 0 | 0 | 3 |
| Oleiphilus messinensis | 0 | 0 | 0 | 0 | 0 |
| Anaplasma ovis | 25 | 47 | 58 | 1 | 55 |
| Metamycoplasma phocicerebrale | 0 | 0 | 0 | 0 | 4 |
| Mycoplasmopsis phocirhinis | 0 | 2 | 0 | 0 | 0 |
| [Mycoplasma] phocidae | 0 | 5 | 0 | 0 | 2 |
| Cylindrospermum stagnale | 47 | 833 | 248 | 0 | 203 |
| Desulfitobacterium metallireducens | 66 | 61 | 94 | 1 | 104 |
| Salegentibacter salegens | 21 | 48 | 37 | 20 | 46 |
| Aneurinibacillus thermoaerophilus | 54 | 205 | 114 | 0 | 78 |
| Rathayibacter toxicus | 238 | 377 | 398 | 8 | 566 |
| Mycolicibacterium boenickei | 4196 | 12643 | 8135 | 46 | 19983 |
| Acetobacter orientalis | 100 | 73 | 50 | 0 | 105 |
| Thermosynechococcus vestitus | 0 | 0 | 0 | 0 | 0 |
| Corynebacterium simulans | 0 | 0 | 0 | 0 | 0 |
| Salinibacter ruber | 1013 | 2406 | 2211 | 12 | 2695 |
| Streptomyces griseofuscus | 1187 | 997 | 1966 | 105 | 2673 |
| Streptomyces parvulus | 1981 | 1847 | 2885 | 201 | 4341 |
| Collinsella stercoris | 474 | 597 | 698 | 3 | 772 |
| Pseudomonas sp. SW-3 | 0 | 0 | 0 | 0 | 0 |
| Thiomicrospira cyclica | 15 | 144 | 40 | 3 | 59 |
| Limnospira indica | 0 | 0 | 0 | 0 | 0 |
| Carnobacterium inhibens | 0 | 0 | 0 | 0 | 0 |
| Paraburkholderia phymatum | 710 | 1511 | 1248 | 6 | 1539 |
| Sulfuricurvum kujiense | 1 | 27 | 53 | 0 | 66 |
| Apilactobacillus kunkeei | 0 | 15 | 22 | 0 | 57 |
| Streptococcus urinalis | 4 | 10 | 9 | 0 | 4 |
| Marinitoga piezophila | 0 | 17 | 17 | 0 | 22 |
| Leifsonia shinshuensis | 1684 | 2243 | 2895 | 105 | 3653 |
| Streptococcus lutetiensis | 0 | 0 | 0 | 0 | 0 |
| Shewanella livingstonensis | 10 | 31 | 13 | 2 | 20 |
| Plantibacter flavus | 733 | 1008 | 1234 | 7 | 1460 |
| Vibrio antiquarius | 0 | 16 | 3 | 0 | 4 |
| Leadbettera azotonutricia | 66 | 180 | 240 | 0 | 231 |
| Pseudoalteromonas ruthenica | 1 | 24 | 16 | 0 | 27 |
| Bacteroides coprosuis | 13 | 10 | 8 | 1 | 12 |
| Lactobacillus intestinalis | 21 | 18 | 11 | 0 | 7 |
| Cupriavidus campinensis | 2171 | 2836 | 3539 | 23 | 5052 |
| Pseudopedobacter saltans | 44 | 98 | 79 | 4 | 117 |
| Mycolicibacterium holsaticum | 3253 | 12730 | 7849 | 94 | 21764 |
| Chloroflexus aggregans | 346 | 913 | 901 | 10 | 1186 |
| Metabacillus litoralis | 31 | 219 | 38 | 0 | 29 |
| Pseudoalteromonas issachenkonii | 4 | 20 | 10 | 0 | 24 |
| Lentilactobacillus parabuchneri | 5 | 33 | 65 | 0 | 47 |
| Burkholderia ambifaria | 150 | 209951 | 276 | 0 | 0 |
| Burkholderia dolosa | 33618 | 8148 | 1469 | 0 | 375 |
| Parageobacillus toebii | 14 | 118 | 100 | 0 | 236 |
| Alysiella crassa | 30 | 61 | 54 | 0 | 57 |
| Conchiformibius steedae | 63 | 59 | 100 | 1 | 108 |
| Kozakia baliensis | 394 | 713 | 513 | 4 | 854 |
| Hungatella hathewayi | 0 | 0 | 0 | 0 | 0 |
| Wolbachia endosymbiont of Ostrinia furnacalis | 0 | 12 | 0 | 0 | 0 |
| Turicibacter sanguinis | 2 | 11 | 17 | 0 | 15 |
| Aliiroseovarius crassostreae | 579 | 532 | 532 | 6 | 672 |
| Cellvibrio japonicus | 119 | 256 | 197 | 2 | 291 |
| Staphylococcus lutrae | 8 | 9 | 11 | 0 | 5 |
| Streptomyces yatensis | 2564 | 1523 | 2704 | 208 | 5062 |
| Bartonella bovis | 14 | 3 | 7 | 0 | 12 |
| Bacillus toyonensis | 4 | 15 | 57 | 0 | 53 |
| Weissella soli | 3 | 9 | 10 | 0 | 10 |
| Caulobacter vibrioides | 0 | 0 | 0 | 0 | 0 |
| Chroococcidiopsis sp. CCMEE 29 | 0 | 0 | 0 | 0 | 0 |
| Nitrosospira sp. NRS527 | 276 | 826 | 437 | 2 | 721 |
| Anabaenopsis elenkinii | 30 | 22 | 27 | 1 | 34 |
| Corynebacterium riegelii | 0 | 0 | 0 | 0 | 0 |
| Microbacterium resistens | 1037 | 1214 | 1765 | 60 | 2579 |
| Arthrobacter woluwensis | 684 | 972 | 1527 | 80 | 2730 |
| Helicobacter winghamensis | 2 | 5 | 15 | 0 | 6 |
| Sterolibacterium denitrificans | 1159 | 1494 | 1835 | 39 | 2755 |
| [Mannheimia] succiniciproducens | 12 | 16 | 6 | 0 | 60 |
| Leptotrichia wadei | 8 | 10 | 8 | 0 | 15 |
| Leptotrichia shahii | 2 | 3 | 4 | 0 | 0 |
| Pseudoleptotrichia goodfellowii | 49 | 11 | 19 | 0 | 12 |
| Kushneria marisflavi | 0 | 0 | 0 | 0 | 0 |
| Pseudomonas parafulva | 0 | 0 | 0 | 0 | 0 |
| Chromohalobacter salexigens | 0 | 0 | 0 | 0 | 0 |
| Hahella chejuensis | 265 | 385 | 419 | 58 | 539 |
| Novosphingobium resinovorum | 3393 | 3080 | 3083 | 8 | 3474 |
| Pseudomonas graminis | 0 | 0 | 0 | 0 | 0 |
| Bifidobacterium scardovii | 561 | 576 | 692 | 8 | 960 |
| Cedecea neteri | 371 | 846 | 725 | 7 | 859 |
| Enterobacter hormaechei | 11287 | 15083 | 16574 | 215 | 14806 |
| Leminorella richardii | 93 | 131 | 162 | 0 | 201 |
| Megamonas hypermegale | 1 | 26 | 17 | 3 | 26 |
| Moellerella wisconsensis | 1 | 59 | 46 | 3 | 41 |
| Providencia rustigianii | 38 | 16 | 39 | 1 | 40 |
| Gordonia westfalica | 9 | 1 | 5 | 0 | 9 |
| Collimonas fungivorans | 1166 | 1898 | 1552 | 13 | 2311 |
| Roseibacterium elongatum | 693 | 666 | 739 | 4 | 954 |
| Paenibacillus terrae | 32 | 174 | 98 | 9 | 135 |
| Corynebacterium casei | 0 | 0 | 0 | 0 | 0 |
| Enterococcus gilvus | 0 | 0 | 0 | 0 | 0 |
| Agrobacterium larrymoorei | 580 | 607 | 377 | 25 | 887 |
| Rhizorhabdus wittichii | 3887 | 4191 | 4179 | 83 | 7533 |
| Paenibacillus borealis | 178 | 398 | 254 | 0 | 448 |
| Acidithiobacillus ferrivorans | 349 | 726 | 442 | 34 | 656 |
| Comamonas koreensis | 0 | 0 | 0 | 0 | 0 |
| Nocardioides aquaticus | 2325 | 3057 | 4039 | 139 | 4108 |
| Thermanaeromonas toyohensis | 84 | 140 | 174 | 0 | 183 |
| Micrococcus sp. 28 | 5 | 0 | 24 | 0 | 25 |
| Planococcus antarcticus | 21 | 52 | 58 | 0 | 70 |
| Pseudoalteromonas phenolica | 27 | 30 | 14 | 3 | 59 |
| Anaeromyxobacter dehalogenans | 4683 | 8420 | 10259 | 202 | 11542 |
| Corynebacterium phocae | 156 | 146 | 201 | 2 | 255 |
| Corynebacterium camporealensis | 162 | 248 | 395 | 23 | 539 |
| Corynebacterium singulare | 0 | 0 | 0 | 0 | 0 |
| Paenibacillus naphthalenovorans | 0 | 0 | 0 | 0 | 0 |
| Microbacterium hominis | 4784 | 5619 | 10775 | 199 | 10083 |
| Glutamicibacter creatinolyticus | 521 | 684 | 879 | 6 | 945 |
| Pseudomonas lini | 460 | 1799 | 2518 | 0 | 2541 |
| Acidiferrobacter thiooxydans | 431 | 1003 | 852 | 24 | 1131 |
| Nocardiopsis exhalans | 1761 | 1669 | 3490 | 374 | 6529 |
| Virgibacillus necropolis | 7 | 15 | 18 | 0 | 23 |
| Latilactobacillus fuchuensis | 19 | 13 | 5 | 0 | 26 |
| Blattabacterium punctulatus | 0 | 11 | 23 | 0 | 17 |
| Blattabacterium clevelandi | 0 | 2 | 0 | 0 | 1 |
| Cupriavidus taiwanensis | 6821 | 9469 | 14114 | 191 | 14721 |
| Novosphingobium sp. KA1 | 844 | 877 | 719 | 0 | 803 |
| Weissella koreensis | 1 | 2 | 4 | 0 | 27 |
| Bartonella schoenbuchensis | 0 | 8 | 0 | 0 | 6 |
| Synechococcus sp. MIT S9220 | 65 | 157 | 129 | 1 | 168 |
| Synechococcus sp. WH 8109 | 70 | 83 | 94 | 0 | 127 |
| Roseburia intestinalis | 4 | 47 | 63 | 2 | 64 |
| Thermobaculum terrenum | 370 | 666 | 717 | 36 | 1149 |
| Pseudoalteromonas translucida | 0 | 0 | 0 | 0 | 0 |
| Ilyobacter polytropus | 15 | 16 | 5 | 0 | 17 |
| Wolbachia endosymbiont of Aedes albopictus | 0 | 3 | 8 | 0 | 9 |
| Anaerolinea thermophila | 210 | 462 | 542 | 5 | 759 |
| Candidatus Ishikawaella capsulata | 2 | 12 | 10 | 0 | 5 |
| Marvinbryantia formatexigens | 76 | 247 | 219 | 27 | 481 |
| Laribacter hongkongensis | 732 | 1019 | 1577 | 0 | 1944 |
| Salinispora tropica | 1333 | 1343 | 4031 | 213 | 5972 |
| Caenibius tardaugens | 1658 | 2319 | 1652 | 0 | 2457 |
| Wolbachia endosymbiont of Folsomia candida | 0 | 0 | 0 | 0 | 0 |
| Paraburkholderia terricola | 1340 | 2097 | 1568 | 31 | 1560 |
| Paraburkholderia hospita | 1323 | 2670 | 2371 | 51 | 3361 |
| Anaerotruncus colihominis | 323 | 642 | 592 | 18 | 693 |
| Pseudomonas extremorientalis | 0 | 0 | 0 | 0 | 0 |
| Paenibacillus stellifer | 316 | 506 | 374 | 36 | 867 |
| Mycobacterium lacus | 1861 | 3560 | 2438 | 9 | 5125 |
| Vibrio kanaloae | 7 | 57 | 12 | 0 | 34 |
| Vibrio chagasii | 61 | 85 | 65 | 0 | 84 |
| Mycoplasmopsis anatis | 3 | 0 | 0 | 0 | 2 |
| Mycoplasmopsis citelli | 1 | 2 | 0 | 0 | 3 |
| Mycoplasmopsis columboralis | 0 | 0 | 0 | 0 | 0 |
| Mycoplasmopsis cynos | 0 | 8 | 4 | 0 | 2 |
| Mycoplasmopsis glycophila | 0 | 0 | 0 | 0 | 0 |
| Mesomycoplasma molare | 0 | 0 | 0 | 0 | 0 |
| Paenibacillus agaridevorans | 170 | 409 | 435 | 0 | 508 |
| Tistrella mobilis | 4192 | 4179 | 4949 | 41 | 7336 |
| Photorhabdus akhurstii | 0 | 0 | 0 | 0 | 0 |
| Mycoplasma ovis | 2 | 0 | 0 | 0 | 1 |
| Thermodesulfatator indicus | 51 | 160 | 72 | 2 | 148 |
| Sulfuriferula plumbiphila | 684 | 1460 | 1238 | 40 | 2156 |
| Brevundimonas nasdae | 875 | 945 | 771 | 0 | 716 |
| Paenibacillus kribbensis | 33 | 129 | 106 | 0 | 158 |
| Meiothermus taiwanensis | 0 | 0 | 0 | 0 | 0 |
| Methylocystis rosea | 2371 | 1850 | 1861 | 32 | 2627 |
| Gemmatimonas aurantiaca | 2175 | 7740 | 5373 | 46 | 22607 |
| Streptomyces rectiverticillatus | 2226 | 1898 | 3422 | 315 | 7037 |
| Carnobacterium viridans | 0 | 9 | 10 | 0 | 6 |
| Candidatus Kuenenia stuttgartiensis | 155 | 376 | 408 | 12 | 607 |
| Pseudoalteromonas agarivorans | 26 | 70 | 52 | 0 | 51 |
| Secundilactobacillus malefermentans | 46 | 23 | 4 | 0 | 2 |
| Pseudothermotoga lettingae | 0 | 0 | 0 | 0 | 22 |
| Marinomonas primoryensis | 47 | 86 | 31 | 4 | 74 |
| Leptospirillum ferriphilum | 0 | 0 | 0 | 0 | 0 |
| Carboxydocella thermautotrophica | 80 | 135 | 155 | 0 | 153 |
| Alicycliphilus denitrificans | 6161 | 6068 | 6991 | 0 | 7850 |
| Burkholderia anthina | 1245 | 663 | 1324 | 0 | 0 |
| Pectobacterium brasiliense | 302 | 441 | 320 | 8 | 493 |
| Schaalia cardiffensis | 96 | 224 | 332 | 54 | 808 |
| Desulfosudis oleivorans | 301 | 872 | 823 | 3 | 1207 |
| Pseudoalteromonas prydzensis | 0 | 0 | 0 | 0 | 0 |
| Aromatoleum anaerobium | 31 | 66 | 126 | 0 | 83 |
| Pseudodesulfovibrio aespoeensis | 766 | 1353 | 1367 | 16 | 1630 |
| Agarivorans albus | 14 | 21 | 9 | 0 | 12 |
| Erwinia billingiae | 235 | 4514 | 484 | 0 | 662 |
| Kribbella flavida | 8206 | 6739 | 24705 | 218 | 19016 |
| Oceanobacillus iheyensis | 6 | 6 | 15 | 0 | 32 |
| Clostridium isatidis | 6 | 5 | 10 | 0 | 9 |
| Proteus hauseri | 17 | 7 | 21 | 0 | 10 |
| Thermodesulfobium narugense | 12 | 16 | 38 | 0 | 13 |
| Paracoccus seriniphilus | 0 | 0 | 0 | 0 | 0 |
| Vibrio ruber | 11 | 45 | 105 | 1 | 68 |
| Citrobacter sp. TSA-1 | 0 | 6 | 8 | 0 | 24 |
| Corallococcus coralloides | 2415 | 4919 | 4987 | 42 | 6557 |
| Solidesulfovibrio magneticus | 477 | 1080 | 1054 | 34 | 1371 |
| Pseudobutyrivibrio xylanivorans | 19 | 19 | 6 | 0 | 15 |
| Butyrivibrio hungatei | 20 | 23 | 23 | 0 | 29 |
| Paludibacter propionicigenes | 49 | 76 | 59 | 1 | 69 |
| Ruegeria sp. PR1b | 21 | 28 | 11 | 0 | 17 |
| Mycobacterium parmense | 2050 | 4113 | 2955 | 30 | 6086 |
| Sphingomonas aerolata | 386 | 3020 | 1977 | 0 | 2108 |
| Xylanimonas cellulosilytica | 1747 | 1790 | 2540 | 50 | 2702 |
| Marinithermus hydrothermalis | 993 | 1549 | 1625 | 7 | 2132 |
| Candidatus Baumannia cicadellinicola | 12 | 16 | 29 | 0 | 18 |
| Thioalkalivibrio nitratireducens | 1185 | 1790 | 1812 | 27 | 2374 |
| Sneathia vaginalis | 1 | 3 | 1 | 0 | 13 |
| Oceanithermus profundus | 1131 | 2148 | 2365 | 25 | 2986 |
| Caldithrix abyssi | 127 | 546 | 378 | 18 | 636 |
| Methylocystis sp. SC2 | 932 | 861 | 756 | 0 | 1082 |
| Roseibium aggregatum | 2759 | 2602 | 2018 | 1 | 2578 |
| Acidaminococcus intestini | 80 | 119 | 120 | 0 | 149 |
| Pediococcus claussenii | 23 | 44 | 7 | 0 | 11 |
| Corynebacterium glaucum | 0 | 0 | 0 | 0 | 0 |
| Thalassolituus oleivorans | 59 | 82 | 117 | 11 | 113 |
| Streptomyces koyangensis | 1910 | 1792 | 3001 | 306 | 5168 |
| Cetobacterium somerae | 0 | 8 | 7 | 0 | 9 |
| Pedobacter cryoconitis | 37 | 176 | 142 | 9 | 273 |
| Rossellomorea marisflavi | 101 | 297 | 339 | 7 | 625 |
| Paenibacillus graminis | 87 | 172 | 108 | 3 | 229 |
| Paenibacillus odorifer | 116 | 606 | 150 | 1 | 241 |
| Actinobacillus porcitonsillarum | 1 | 48 | 6 | 2 | 22 |
| Bradyrhizobium paxllaeri | 25761 | 24474 | 13001 | 51 | 12556 |
| Vibrio coralliilyticus | 49 | 145 | 120 | 3 | 149 |
| Vibrio rotiferianus | 8 | 15 | 49 | 4 | 17 |
| Vibrio gallicus | 164 | 49 | 21 | 6 | 27 |
| Maridesulfovibrio hydrothermalis | 18 | 80 | 52 | 3 | 119 |
| Rhodococcus aetherivorans | 4445 | 2921 | 3695 | 137 | 4482 |
| Devosia neptuniae | 1561 | 1279 | 1140 | 22 | 1875 |
| Pseudomonas palleroniana | 0 | 0 | 0 | 0 | 0 |
| Corynebacterium sphenisci | 1082 | 1136 | 1639 | 68 | 2269 |
| Conexibacter woesei | 14061 | 11778 | 29293 | 188 | 31948 |
| Fluviicola taffensis | 18 | 56 | 44 | 0 | 47 |
| Corynebacterium atypicum | 451 | 636 | 876 | 10 | 1040 |
| Shewanella denitrificans | 32 | 59 | 64 | 16 | 53 |
| Chryseobacterium daecheongense | 0 | 0 | 0 | 0 | 0 |
| Qipengyuania flava | 1541 | 1946 | 1678 | 26 | 2712 |
| Rhodoferax ferrireducens | 505 | 999 | 888 | 9 | 1289 |
| Streptomyces niveus | 4341 | 3240 | 7022 | 571 | 13387 |
| Alysiella filiformis | 7 | 48 | 46 | 0 | 74 |
| Sulfurospirillum halorespirans | 7 | 27 | 26 | 5 | 15 |
| Sinorhizobium americanum | 1523 | 1854 | 1495 | 7 | 1865 |
| Haematobacter massiliensis | 1457 | 1875 | 1365 | 3 | 1870 |
| Synechococcus sp. PCC 8807 | 0 | 32 | 18 | 0 | 67 |
| Synechococcus sp. PCC 7336 | 161 | 349 | 324 | 6 | 510 |
| Synechococcus sp. PCC 6312 | 12 | 125 | 102 | 3 | 132 |
| Synechococcus sp. PCC 7117 | 65 | 114 | 30 | 0 | 59 |
| Aeromonas dhakensis | 983 | 1674 | 1606 | 58 | 1664 |
| Nocardioides sp. JS614 | 4030 | 7118 | 8798 | 337 | 9299 |
| Anoxybacillus kamchatkensis | 0 | 29 | 27 | 0 | 78 |
| Actinoplanes friuliensis | 4706 | 3437 | 10641 | 262 | 9946 |
| Deferribacter desulfuricans | 33 | 31 | 46 | 1 | 60 |
| Paraglaciecola mesophila | 0 | 0 | 0 | 0 | 0 |
| Malaciobacter halophilus | 1 | 9 | 3 | 0 | 6 |
| Streptococcus pasteurianus | 0 | 0 | 0 | 0 | 0 |
| Paenibacillus barcinonensis | 0 | 0 | 0 | 0 | 0 |
| Candidatus Pelagibacter ubique | 12 | 10 | 9 | 0 | 21 |
| Anoxybacillus gonensis | 0 | 27 | 44 | 0 | 76 |
| Macrococcus brunensis | 0 | 62 | 14 | 0 | 12 |
| Pseudomonas umsongensis | 1878 | 684 | 518 | 4 | 1187 |
| Pseudomonas koreensis | 466 | 645 | 594 | 38 | 1329 |
| Vibrio pomeroyi | 1 | 46 | 12 | 0 | 49 |
| Arthrobacter koreensis | 334 | 500 | 885 | 60 | 1228 |
| Alkalihalobacillus krulwichiae | 40 | 19 | 39 | 0 | 34 |
| Brevibacterium luteolum | 852 | 1307 | 1801 | 67 | 2428 |
| Microbacterium paraoxydans | 371 | 716 | 973 | 0 | 449 |
| Methylocella silvestris | 1635 | 1593 | 1383 | 39 | 2045 |
| Deinococcus aetherius | 808 | 1378 | 1728 | 120 | 2154 |
| Aromatoleum buckelii | 110 | 129 | 122 | 3 | 161 |
| Pseudomonas trivialis | 230 | 485 | 533 | 3 | 2624 |
| Pseudomonas poae | 182 | 475 | 563 | 9 | 1587 |
| Pseudomonas congelans | 176 | 529 | 255 | 2 | 605 |
| Pseudomonas tremae | 65 | 68 | 75 | 11 | 175 |
| Nocardioides aromaticivorans | 3051 | 4519 | 5478 | 162 | 6378 |
| Planococcus rifietoensis | 175 | 178 | 98 | 0 | 137 |
| Streptomyces sp. KCTC 0041BP | 2 | 0 | 3 | 0 | 4 |
| Maize bushy stunt phytoplasma | 0 | 6 | 0 | 0 | 0 |
| Sulfurimonas autotrophica | 0 | 0 | 0 | 0 | 0 |
| Streptomyces drozdowiczii | 2226 | 1870 | 3473 | 252 | 5558 |
| Acinetobacter baylyi | 0 | 0 | 0 | 0 | 0 |
| Acinetobacter bouvetii | 18 | 152 | 55 | 0 | 66 |
| Corynebacterium aquilae | 283 | 317 | 455 | 2 | 559 |
| Dickeya dadantii | 404 | 602 | 588 | 68 | 543 |
| Dickeya dianthicola | 748 | 584 | 249 | 1 | 2277 |
| Dickeya zeae | 0 | 0 | 0 | 0 | 0 |
| Roseomonas cervicalis | 2064 | 1927 | 2005 | 40 | 2957 |
| Nocardia iowensis | 2921 | 2017 | 5049 | 187 | 6614 |
| Novosphingobium pentaromativorans | 425 | 1190 | 711 | 0 | 332 |
| Pseudoalteromonas mariniglutinosa | 59 | 49 | 44 | 0 | 40 |
| Sulfurovum lithotrophicum | 2 | 58 | 54 | 0 | 38 |
| Kerstersia gyiorum | 0 | 0 | 0 | 0 | 0 |
| Streptomyces sp. FR-008 | 676 | 1010 | 886 | 10 | 949 |
| Roseomonas mucosa | 2374 | 2267 | 2427 | 0 | 1754 |
| Variovorax sp. WDL1 | 5977 | 9797 | 8586 | 181 | 11728 |
| Lyngbya confervoides | 0 | 2 | 2 | 2 | 0 |
| Bermanella marisrubri | 11 | 36 | 35 | 9 | 70 |
| Pseudalkalibacillus hwajinpoensis | 0 | 0 | 2 | 0 | 2 |
| Parvularcula bermudensis | 466 | 821 | 650 | 4 | 1005 |
| Enterobacter kobei | 550 | 1319 | 1229 | 0 | 914 |
| Alkaliphilus metalliredigens | 37 | 24 | 30 | 2 | 20 |
| Amycolatopsis japonica | 8526 | 1765 | 7283 | 117 | 5154 |
| Enterocloster bolteae | 77 | 190 | 225 | 0 | 313 |
| Carnobacterium sp. 17-4 | 0 | 0 | 0 | 0 | 0 |
| Nocardia yamanashiensis | 1980 | 2288 | 3850 | 127 | 4658 |
| Allokutzneria albata | 16168 | 4092 | 14374 | 411 | 11586 |
| Streptomyces sp. EN27 | 1 | 1 | 0 | 0 | 3 |
| Vibrio tasmaniensis | 2 | 32 | 13 | 0 | 17 |
| Vibrio fortis | 24 | 55 | 38 | 0 | 57 |
| Mycolicibacterium madagascariense | 3075 | 7792 | 5582 | 46 | 11184 |
| Mycobacterium sp. JS623 | 7868 | 35279 | 21351 | 121 | 77061 |
| Geobacillus zalihae | 0 | 0 | 0 | 0 | 0 |
| Halomonas campaniensis | 0 | 140 | 86 | 71 | 788 |
| Subdoligranulum variabile | 0 | 0 | 0 | 0 | 0 |
| Alistipes finegoldii | 368 | 916 | 926 | 8 | 1257 |
| Williamsoniiplasma luminosum | 1 | 2 | 4 | 6 | 10 |
| Wolbachia endosymbiont of Bemisia tabaci | 0 | 0 | 0 | 0 | 0 |
| Komagataeibacter rhaeticus | 458 | 219 | 85 | 0 | 289 |
| Williamsoniiplasma somnilux | 5 | 23 | 12 | 0 | 14 |
| Caldimonas thermodepolymerans | 1463 | 2799 | 2683 | 0 | 3288 |
| Gracilinema caldarium | 18 | 95 | 90 | 0 | 122 |
| Erwinia sp. Ejp617 | 12 | 59 | 29 | 0 | 75 |
| Thermomonas brevis | 1767 | 1557 | 1855 | 17 | 1698 |
| Roseovarius mucosus | 415 | 423 | 516 | 27 | 506 |
| Dinoroseobacter shibae | 983 | 1104 | 1338 | 27 | 1528 |
| Mesoplasma chauliocola | 0 | 6 | 4 | 0 | 7 |
| Polaromonas naphthalenivorans | 761 | 1515 | 1098 | 0 | 1756 |
| Stenotrophomonas rhizophila | 2277 | 3082 | 2404 | 23 | 5438 |
| Bifidobacterium longum | 861 | 1470 | 2551 | 29 | 2974 |
| Mycolicibacterium psychrotolerans | 2511 | 8140 | 5470 | 101 | 12400 |
| Spiroplasma alleghenense | 0 | 0 | 1 | 0 | 6 |
| Spiroplasma chinense | 0 | 1 | 4 | 0 | 4 |
| Spiroplasma chrysopicola | 16 | 1 | 0 | 0 | 0 |
| Spiroplasma corruscae | 0 | 2 | 6 | 0 | 3 |
| Spiroplasma culicicola | 0 | 5 | 0 | 0 | 2 |
| Spiroplasma diminutum | 0 | 2 | 9 | 0 | 2 |
| Spiroplasma floricola | 0 | 6 | 1 | 0 | 3 |
| Spiroplasma helicoides | 3 | 1 | 4 | 0 | 0 |
| Spiroplasma litorale | 0 | 1 | 0 | 0 | 4 |
| Spiroplasma sabaudiense | 0 | 0 | 0 | 0 | 0 |
| Spiroplasma syrphidicola | 8 | 0 | 0 | 0 | 0 |
| Spiroplasma turonicum | 1 | 4 | 2 | 0 | 1 |
| Spiroplasma sp. TIUS-1 | 0 | 2 | 2 | 0 | 2 |
| Achromobacter spanius | 3103 | 3698 | 4133 | 120 | 5215 |
| Achromobacter insolitus | 0 | 0 | 0 | 0 | 0 |
| Vagococcus carniphilus | 8 | 6 | 34 | 0 | 7 |
| Desulfatibacillum aliphaticivorans | 307 | 644 | 633 | 13 | 853 |
| Rossellomorea vietnamensis | 0 | 0 | 0 | 0 | 0 |
| Sulfitobacter dubius | 140 | 321 | 242 | 0 | 448 |
| Amycolatopsis pretoriensis | 16204 | 3752 | 14572 | 318 | 9583 |
| Aeromonas simiae | 440 | 791 | 790 | 6 | 728 |
| Aeromicrobium marinum | 8486 | 16448 | 12347 | 77 | 5880 |
| Mesoplasma tabanidae | 0 | 0 | 4 | 0 | 14 |
| Neobacillus drentensis | 20 | 63 | 64 | 0 | 114 |
| Thalassospira xiamenensis | 168 | 266 | 291 | 13 | 380 |
| Mycobacterium saskatchewanense | 2555 | 4317 | 3831 | 107 | 7140 |
| Treponema putidum | 42 | 29 | 27 | 0 | 24 |
| Synechococcus sp. Minos11 | 152 | 380 | 263 | 0 | 272 |
| Synechococcus sp. RS9902 | 7 | 200 | 128 | 13 | 411 |
| Synechococcus sp. RS9907 | 124 | 135 | 188 | 0 | 530 |
| Synechococcus sp. RS9909 | 129 | 389 | 312 | 4 | 295 |
| Mergibacter septicus | 10 | 22 | 26 | 1 | 17 |
| Phaeobacter inhibens | 1088 | 1112 | 981 | 15 | 1443 |
| Helicobacter enhydrae | 12 | 10 | 7 | 0 | 6 |
| [Phormidium] sp. ETS-05 | 165 | 645 | 344 | 4 | 673 |
| Ancylobacter polymorphus | 4903 | 3371 | 3623 | 52 | 5165 |
| Rhodococcus gordoniae | 1090 | 919 | 1428 | 22 | 1365 |
| Halomonas sp. 40 | 0 | 0 | 0 | 0 | 1 |
| Clostridium diolis | 173 | 109 | 180 | 0 | 154 |
| Methylorubrum populi | 4140 | 3808 | 3777 | 69 | 3512 |
| Nostoc piscinale | 64 | 219 | 107 | 4 | 203 |
| Erysipelothrix inopinata | 4 | 6 | 7 | 2 | 11 |
| Candidatus Rhabdochlamydia porcellionis | 11 | 80 | 41 | 0 | 36 |
| Citrifermentans bemidjiense | 605 | 1749 | 1603 | 15 | 1818 |
| Corynebacterium halotolerans | 749 | 712 | 1010 | 54 | 1371 |
| Sulfitobacter indolifex | 293 | 330 | 306 | 1 | 440 |
| Shewanella psychrophila | 23 | 70 | 52 | 0 | 55 |
| Comamonas aquatica | 1459 | 2975 | 2454 | 0 | 1855 |
| Mesoplasma syrphidae | 17 | 4 | 16 | 0 | 3 |
| Actinocatenispora thailandica | 3834 | 2732 | 6455 | 412 | 7741 |
| Methylocella tundrae | 1443 | 1275 | 915 | 0 | 1398 |
| Bacillus tequilensis | 0 | 0 | 0 | 0 | 0 |
| Limosilactobacillus gastricus | 0 | 0 | 0 | 0 | 0 |
| Lactobacillus ultunensis | 1 | 16 | 35 | 0 | 24 |
| Ligilactobacillus saerimneri | 8 | 25 | 21 | 0 | 24 |
| Nocardia arthritidis | 2709 | 1932 | 4984 | 209 | 5708 |
| Thermovibrio ammonificans | 56 | 146 | 164 | 0 | 236 |
| Peribacillus asahii | 0 | 0 | 0 | 0 | 0 |
| Borrelia turcica | 1 | 1 | 2 | 0 | 0 |
| Aster yellows witches'-broom phytoplasma | 0 | 0 | 19 | 0 | 10 |
| Photorhabdus thracensis | 100 | 57 | 59 | 3 | 59 |
| Furfurilactobacillus rossiae | 17 | 60 | 45 | 1 | 32 |
| Pseudodesulfovibrio portus | 533 | 596 | 710 | 8 | 981 |
| Dyella japonica | 1467 | 1340 | 1466 | 88 | 1723 |
| Belliella baltica | 0 | 0 | 0 | 0 | 0 |
| Synechococcus sp. CB0101 | 137 | 372 | 413 | 15 | 444 |
| Rhodoluna limnophila | 63 | 179 | 153 | 19 | 198 |
| Desulfitobacterium dichloroeliminans | 108 | 93 | 74 | 2 | 123 |
| Ectothiorhodosinus mongolicus | 100 | 257 | 169 | 2 | 259 |
| Candidatus Kinetoplastibacterium blastocrithidii | 3 | 7 | 3 | 0 | 8 |
| Alteromonas stellipolaris | 0 | 15 | 20 | 14 | 31 |
| Streptomyces sp. TN58 | 1402 | 1414 | 2678 | 295 | 4807 |
| Candidatus Midichloria mitochondrii | 16 | 26 | 65 | 0 | 50 |
| Pseudoalteromonas sp. SM9913 | 6 | 6 | 16 | 0 | 0 |
| Arsenophonus endosymbiont of Aleurodicus dispersus | 1 | 7 | 11 | 0 | 17 |
| Pseudomonas alkylphenolica | 0 | 0 | 0 | 0 | 0 |
| Pseudomonas psychrotolerans | 1593 | 1983 | 2242 | 31 | 2661 |
| Rickettsia asiatica | 0 | 5 | 2 | 0 | 6 |
| Shewanella donghaensis | 0 | 0 | 0 | 0 | 0 |
| Akkermansia muciniphila | 719 | 1855 | 1361 | 31 | 1833 |
| Secundilactobacillus paracollinoides | 31 | 82 | 59 | 0 | 67 |
| Pseudonocardia dioxanivorans | 10085 | 6172 | 15852 | 510 | 14894 |
| Rhizobium daejeonense | 991 | 911 | 714 | 4 | 588 |
| Rummeliibacillus stabekisii | 0 | 30 | 33 | 0 | 53 |
| Desulfovibrio ferrophilus | 138 | 313 | 320 | 5 | 435 |
| Crinalium epipsammum | 69 | 144 | 103 | 0 | 338 |
| Ichthyobacterium seriolicida | 0 | 0 | 0 | 0 | 0 |
| Luteibacter rhizovicinus | 1296 | 1357 | 1518 | 23 | 1626 |
| endosymbiont 'TC1' of Trimyema compressum | 70 | 18 | 52 | 1 | 46 |
| Klebsiella variicola | 427 | 1006 | 1094 | 46 | 1043 |
| Pseudomonas lurida | 88 | 1641 | 348 | 0 | 6811 |
| Candidatus Paracaedibacter symbiosus | 1 | 7 | 3 | 0 | 25 |
| Hoeflea phototrophica | 949 | 896 | 948 | 5 | 1128 |
| Bradyrhizobium betae | 0 | 0 | 0 | 0 | 0 |
| Nautilia profundicola | 0 | 0 | 0 | 0 | 0 |
| Yoonia vestfoldensis | 511 | 466 | 459 | 1 | 535 |
| Vibrio crassostreae | 14 | 58 | 102 | 0 | 94 |
| Wolbachia endosymbiont of Cimex lectularius | 6 | 5 | 6 | 0 | 2 |
| Bacteroides cellulosilyticus | 0 | 0 | 0 | 0 | 0 |
| Serinicoccus marinus | 993 | 1352 | 1759 | 47 | 1824 |
| Anoxybacillus caldiproteolyticus | 18 | 72 | 61 | 2 | 78 |
| Candidatus Cardinium hertigii | 3 | 4 | 3 | 0 | 7 |
| Pseudoalteromonas aliena | 71 | 183 | 21 | 0 | 26 |
| Cupriavidus pinatubonensis | 795 | 2695 | 1396 | 19 | 3604 |
| Mucispirillum schaedleri | 85 | 112 | 71 | 0 | 82 |
| Paenibacillus xylanilyticus | 50 | 115 | 72 | 0 | 169 |
| Gordonia otitidis | 975 | 1054 | 1622 | 28 | 2007 |
| Cardinium endosymbiont of Encarsia pergandiella | 7 | 5 | 7 | 0 | 14 |
| Streptomyces decoyicus | 1486 | 927 | 2178 | 216 | 5325 |
| Streptomyces asoensis | 4076 | 2100 | 4593 | 71 | 4680 |
| Candidatus Blochmannia vafer | 23 | 15 | 6 | 0 | 8 |
| Candidatus Blochmannia ocreatus | 0 | 1 | 2 | 0 | 1 |
| Candidatus Blochmannia vicinus | 1 | 4 | 6 | 0 | 6 |
| Candidatus Blochmannia chromaiodes | 0 | 0 | 10 | 0 | 13 |
| Pseudomonas syringae group genomosp. 7 | 0 | 0 | 0 | 0 | 0 |
| Uruburuella suis | 288 | 321 | 216 | 5 | 342 |
| Robiginitalea biformata | 177 | 396 | 385 | 3 | 368 |
| Microbulbifer thermotolerans | 221 | 393 | 345 | 2 | 432 |
| Mahella australiensis | 35 | 161 | 111 | 3 | 161 |
| Thiospirochaeta perfilievii | 12 | 13 | 14 | 0 | 16 |
| Paraburkholderia phenoliruptrix | 1382 | 1499 | 1480 | 18 | 1765 |
| Ethanoligenens harbinense | 274 | 649 | 491 | 3 | 665 |
| Owenweeksia hongkongensis | 50 | 56 | 57 | 0 | 39 |
| Paenibacillus antarcticus | 21 | 136 | 91 | 0 | 64 |
| Aquicella lusitana | 96 | 163 | 344 | 1 | 412 |
| Aquicella siphonis | 125 | 196 | 156 | 0 | 199 |
| Streptococcus halichoeri | 18 | 36 | 18 | 0 | 16 |
| Muricauda aquimarina | 98 | 120 | 69 | 1 | 111 |
| Bradyrhizobium canariense | 21921 | 22621 | 14812 | 107 | 14208 |
| Fictibacillus arsenicus | 31 | 46 | 54 | 0 | 79 |
| Leuconostoc garlicum | 0 | 14 | 2 | 0 | 0 |
| Aliarcobacter cibarius | 0 | 2 | 1 | 0 | 2 |
| Atlantibacter subterranea | 0 | 0 | 0 | 0 | 0 |
| Qipengyuania aquimaris | 0 | 0 | 0 | 0 | 0 |
| Psychrobacter arenosus | 9 | 30 | 40 | 5 | 53 |
| Parvibaculum lavamentivorans | 3836 | 3854 | 3610 | 30 | 5421 |
| Glutamicibacter arilaitensis | 345 | 331 | 494 | 65 | 863 |
| Gulosibacter molinativorax | 611 | 875 | 1197 | 54 | 1821 |
| Shewanella decolorationis | 36 | 80 | 61 | 1 | 153 |
| Gluconobacter thailandicus | 47 | 186 | 140 | 0 | 133 |
| Roseomonas gilardii | 2603 | 2818 | 2954 | 66 | 4667 |
| Streptococcus pseudopneumoniae | 0 | 0 | 0 | 0 | 0 |
| Glutamicibacter mysorens | 0 | 0 | 0 | 0 | 0 |
| Corynebacterium resistens | 225 | 251 | 502 | 15 | 674 |
| Mycolicibacterium fluoranthenivorans | 2509 | 9650 | 5607 | 22 | 12195 |
| Shewanella marisflavi | 0 | 0 | 0 | 0 | 0 |
| Microbulbifer agarilyticus | 0 | 0 | 0 | 0 | 0 |
| Bacillus halotolerans | 6 | 43 | 88 | 3 | 101 |
| Campylobacter insulaenigrae | 0 | 3 | 0 | 0 | 7 |
| Cyanobium sp. NS01 | 445 | 713 | 745 | 7 | 816 |
| Paraburkholderia phytofirmans | 1757 | 2351 | 2673 | 32 | 3702 |
| Mycobacterium liflandii | 0 | 222 | 167 | 0 | 356 |
| Micromonospora auratinigra | 3220 | 2394 | 6274 | 264 | 7791 |
| Kangiella koreensis | 39 | 76 | 67 | 2 | 103 |
| Janibacter melonis | 0 | 0 | 0 | 0 | 0 |
| Lysobacter gummosus | 7758 | 12201 | 3019 | 103 | 1925 |
| Yersinia aleksiciae | 10 | 102 | 66 | 1 | 49 |
| Acholeplasma hippikon | 12 | 8 | 17 | 0 | 18 |
| Nostoc edaphicum | 47 | 213 | 151 | 0 | 237 |
| Trichormus variabilis | 0 | 141 | 32 | 0 | 38 |
| Peribacillus muralis | 22 | 133 | 112 | 0 | 95 |
| Kingella potus | 121 | 219 | 189 | 5 | 268 |
| Rhodopirellula baltica | 545 | 1472 | 1094 | 13 | 2161 |
| Vibrio ponticus | 20 | 33 | 49 | 6 | 135 |
| Hydrogenovibrio thermophilus | 28 | 179 | 131 | 1 | 130 |
| Komagataeibacter saccharivorans | 0 | 0 | 0 | 0 | 0 |
| Komagataeibacter nataicola | 322 | 439 | 344 | 9 | 538 |
| Methylovorus glucosotrophus | 122 | 168 | 210 | 0 | 285 |
| Pseudoxanthomonas daejeonensis | 1092 | 1417 | 1135 | 0 | 1072 |
| Kaistella antarctica | 18 | 50 | 39 | 0 | 56 |
| Kaistella jeonii | 8 | 48 | 26 | 0 | 50 |
| Microbulbifer variabilis | 97 | 130 | 153 | 2 | 184 |
| Thalassobacter stenotrophicus | 0 | 0 | 0 | 0 | 0 |
| Sphingopyxis granuli | 2090 | 2410 | 1998 | 36 | 3416 |
| Neisseria bacilliformis | 107 | 295 | 221 | 3 | 283 |
| Levilactobacillus zymae | 95 | 276 | 229 | 0 | 310 |
| Pseudoalteromonas marina | 10 | 67 | 8 | 11 | 7 |
| Lactobacillus kefiranofaciens | 6 | 6 | 3 | 0 | 27 |
| Nitratifractor salsuginis | 137 | 321 | 239 | 2 | 343 |
| Methylobacterium brachiatum | 1800 | 1607 | 1522 | 0 | 1141 |
| Sporolactobacillus terrae | 73 | 136 | 123 | 0 | 91 |
| Methylobacterium aquaticum | 4212 | 3760 | 4104 | 134 | 4269 |
| Salegentibacter mishustinae | 97 | 22 | 54 | 1 | 54 |
| Methylotuvimicrobium alcaliphilum | 176 | 467 | 335 | 0 | 788 |
| Shewanella sediminis | 45 | 70 | 78 | 1 | 80 |
| Shewanella halifaxensis | 0 | 0 | 0 | 0 | 0 |
| Lactiplantibacillus argentoratensis | 0 | 7 | 0 | 0 | 0 |
| Nostoc punctiforme | 46 | 482 | 228 | 3 | 288 |
| Halomonas alkaliphila | 21 | 41 | 48 | 0 | 7 |
| Sphaerochaeta coccoides | 45 | 123 | 110 | 0 | 194 |
| Brevibacterium aurantiacum | 1400 | 1161 | 1761 | 126 | 1929 |
| Exiguobacterium marinum | 0 | 0 | 0 | 0 | 0 |
| Microbacterium oleivorans | 1180 | 1589 | 2619 | 16 | 2028 |
| Microbacterium hydrocarbonoxydans | 567 | 804 | 1317 | 9 | 1064 |
| Chlorobaculum limnaeum | 483 | 1159 | 926 | 11 | 1138 |
| Chlorobaculum parvum | 148 | 513 | 462 | 5 | 623 |
| Limimaricola hongkongensis | 55 | 32 | 54 | 0 | 65 |
| Mesorhizobium sp. WSM1497 | 1182 | 979 | 1148 | 82 | 716 |
| Vibrio neonatus | 5 | 46 | 36 | 38 | 23 |
| Collimonas arenae | 667 | 1369 | 1164 | 0 | 2295 |
| Collimonas pratensis | 887 | 1152 | 1106 | 3 | 1758 |
| Gramella echinicola | 7 | 23 | 25 | 0 | 85 |
| Mesobacillus foraminis | 7 | 75 | 105 | 0 | 91 |
| Microcella alkaliphila | 657 | 1045 | 1191 | 10 | 1391 |
| Acinetobacter colistiniresistens | 17 | 76 | 34 | 1 | 31 |
| Nocardiopsis gilva | 2619 | 1843 | 4346 | 759 | 11565 |
| Aquiflexum balticum | 109 | 87 | 126 | 2 | 95 |
| Prosthecochloris sp. GSB1 | 221 | 547 | 452 | 0 | 677 |
| Candidatus Amoebophilus asiaticus | 11 | 35 | 26 | 0 | 50 |
| Dechloromonas denitrificans | 1258 | 1919 | 1851 | 0 | 2229 |
| Variovorax sp. SRS16 | 3655 | 3606 | 3618 | 56 | 5632 |
| Kosakonia radicincitans | 0 | 0 | 0 | 0 | 0 |
| Pseudoalteromonas sp. Bsw20308 | 31 | 2 | 20 | 8 | 18 |
| Staphylococcus pseudintermedius | 16 | 193 | 59 | 2 | 126 |
| Stackebrandtia nassauensis | 2722 | 1969 | 6145 | 188 | 7009 |
| Leucobacter aridicollis | 400 | 556 | 945 | 44 | 935 |
| Phenylobacterium zucineum | 3669 | 9529 | 4873 | 64 | 7224 |
| Streptomyces lienomycini | 1420 | 1172 | 2102 | 147 | 4161 |
| Priestia koreensis | 18 | 68 | 28 | 0 | 57 |
| Alloalcanivorax dieselolei | 0 | 0 | 0 | 0 | 0 |
| Streptomyces roseochromogenus | 1825 | 1507 | 2903 | 79 | 4281 |
| Streptomyces agglomeratus | 2 | 9 | 13 | 0 | 28 |
| Streptomyces rubrolavendulae | 489 | 693 | 924 | 136 | 2495 |
| Streptomyces sviceus | 731 | 295 | 720 | 112 | 1222 |
| Streptomyces angustmyceticus | 2334 | 2241 | 3983 | 254 | 6544 |
| Micromonospora coriariae | 3042 | 2293 | 7261 | 251 | 7727 |
| Fusobacterium canifelinum | 0 | 3 | 1 | 0 | 0 |
| Azospirillum oryzae | 1400 | 1527 | 1740 | 40 | 2856 |
| Segniliparus rotundus | 618 | 735 | 1071 | 85 | 1973 |
| Bradyrhizobium sp. BTAi1 | 4940 | 3977 | 2501 | 0 | 1351 |
| Beggiatoa leptomitoformis | 0 | 0 | 0 | 0 | 0 |
| Acetivibrio clariflavus | 19 | 33 | 56 | 1 | 47 |
| Pseudomonas argentinensis | 240 | 559 | 445 | 5 | 591 |
| Bacteroides helcogenes | 24 | 46 | 49 | 0 | 65 |
| Xenorhabdus budapestensis | 18 | 37 | 29 | 0 | 17 |
| Tetragenococcus koreensis | 2 | 14 | 42 | 0 | 23 |
| Arthrobacter sp. FB24 | 1326 | 1806 | 3292 | 20 | 9448 |
| Jannaschia sp. CCS1 | 0 | 0 | 0 | 0 | 0 |
| Caminibacter mediatlanticus | 13 | 34 | 35 | 0 | 43 |
| Photorhabdus asymbiotica | 27 | 134 | 44 | 0 | 56 |
| Micromonospora rifamycinica | 3047 | 1968 | 5540 | 144 | 5862 |
| Bacteroides nordii | 48 | 233 | 108 | 2 | 152 |
| Thermosediminibacter oceani | 88 | 181 | 163 | 0 | 187 |
| Ruegeria sp. TM1040 | 214 | 367 | 310 | 7 | 483 |
| Mycoplasma iguanae | 0 | 0 | 0 | 0 | 0 |
| Mycobacterium florentinum | 1428 | 2678 | 2178 | 19 | 4124 |
| Dactylococcopsis salina | 46 | 113 | 200 | 0 | 97 |
| Flavonifractor plautii | 515 | 985 | 1312 | 15 | 1501 |
| Sphingopyxis sp. 113P3 | 1192 | 1713 | 1205 | 112 | 1798 |
| Martelella mediterranea | 2487 | 2261 | 2448 | 136 | 3099 |
| Paucilactobacillus oligofermentans | 16 | 2 | 13 | 1 | 4 |
| Bacillus altitudinis | 194 | 172 | 223 | 2 | 101 |
| Candidatus Hepatoplasma crinochetorum | 5 | 9 | 8 | 0 | 16 |
| Vibrio gigantis | 31 | 32 | 64 | 0 | 26 |
| Polaromonas sp. JS666 | 1073 | 1933 | 1531 | 156 | 3119 |
| Caldalkalibacillus thermarum | 71 | 145 | 121 | 2 | 186 |
| Solidesulfovibrio carbinolicus | 837 | 980 | 1311 | 15 | 1431 |
| Sporosarcina ureilytica | 15 | 18 | 24 | 0 | 32 |
| Frankia inefficax | 5126 | 4419 | 8510 | 348 | 10659 |
| Blattabacterium sp. (Cryptocercus kyebangensis) | 0 | 7 | 1 | 0 | 1 |
| Pseudoalteromonas spongiae | 14 | 39 | 33 | 27 | 24 |
| Micromonospora narathiwatensis | 2683 | 1855 | 5870 | 331 | 6980 |
| Micromonospora siamensis | 2843 | 1831 | 6662 | 261 | 7587 |
| Tateyamaria omphalii | 0 | 0 | 0 | 0 | 0 |
| Francisella orientalis | 48 | 5 | 85 | 0 | 1 |
| Enterobacter ludwigii | 638 | 468 | 741 | 15 | 1376 |
| Microbacterium paludicola | 825 | 885 | 1088 | 14 | 883 |
| Rhodococcus triatomae | 1718 | 1891 | 2618 | 235 | 3636 |
| Serratia ureilytica | 557 | 1434 | 971 | 0 | 660 |
| Glaciecola nitratireducens | 23 | 61 | 86 | 0 | 64 |
| Alkalihalobacillus lehensis | 0 | 0 | 0 | 0 | 0 |
| Vibrio europaeus | 10 | 47 | 32 | 0 | 26 |
| Roseburia hominis | 0 | 0 | 0 | 0 | 0 |
| Spiroplasma platyhelix | 4 | 10 | 6 | 0 | 4 |
| Caldicellulosiruptor acetigenus | 0 | 0 | 0 | 0 | 0 |
| Virgibacillus dokdonensis | 41 | 10 | 23 | 0 | 15 |
| Advenella mimigardefordensis | 194 | 239 | 191 | 3 | 349 |
| Lactobacillus apis | 0 | 2 | 2 | 0 | 3 |
| Schleiferilactobacillus harbinensis | 95 | 193 | 187 | 0 | 206 |
| Sphingomonas sp. IC081 | 0 | 0 | 0 | 0 | 0 |
| Catenulispora acidiphila | 4593 | 3783 | 7371 | 454 | 10448 |
| Micromonospora krabiensis | 3207 | 2947 | 7448 | 307 | 8612 |
| Staphylococcus simiae | 0 | 0 | 0 | 0 | 0 |
| Rhodovulum sp. MB263 | 693 | 767 | 857 | 19 | 1221 |
| Persephonella marina | 74 | 77 | 75 | 0 | 152 |
| Sulfurihydrogenibium azorense | 0 | 0 | 0 | 0 | 0 |
| Candidatus Liberibacter americanus | 4 | 18 | 5 | 0 | 9 |
| Deinococcus maricopensis | 968 | 1420 | 1482 | 19 | 1924 |
| Acaryochloris sp. CCMEE 5410 | 13 | 42 | 16 | 0 | 25 |
| Advenella kashmirensis | 196 | 390 | 306 | 20 | 341 |
| Fervidobacterium changbaicum | 0 | 16 | 4 | 0 | 10 |
| Yangia pacifica | 1015 | 1296 | 1447 | 8 | 1957 |
| Denitratisoma oestradiolicum | 551 | 992 | 971 | 16 | 1779 |
| Paraburkholderia terrae | 1878 | 2617 | 2389 | 11 | 3258 |
| Paraburkholderia ginsengisoli | 938 | 1233 | 1162 | 35 | 2000 |
| Arthrobacter sp. 68b | 11 | 7 | 18 | 0 | 55 |
| Flavobacterium indicum | 16 | 44 | 27 | 0 | 27 |
| Trueperella bialowiezensis | 151 | 335 | 330 | 7 | 402 |
| Pseudomonas entomophila | 1372 | 1459 | 1438 | 14 | 2414 |
| Croceibacter atlanticus | 19 | 54 | 34 | 0 | 40 |
| Dokdonia sp. MED134 | 40 | 7 | 14 | 0 | 8 |
| Polaribacter sp. MED152 | 27 | 18 | 9 | 0 | 12 |
| Maribacter sp. HTCC2170 | 25 | 73 | 17 | 0 | 27 |
| Trichlorobacter lovleyi | 462 | 1543 | 1245 | 3 | 1638 |
| Alteromonas mediterranea | 151 | 72 | 313 | 7 | 499 |
| Pseudoalteromonas tunicata | 15 | 56 | 23 | 0 | 18 |
| Psychromonas sp. CNPT3 | 24 | 22 | 20 | 0 | 13 |
| Pseudoxanthomonas suwonensis | 2374 | 2517 | 2769 | 0 | 1839 |
| Spiroplasma eriocheiris | 4 | 4 | 2 | 0 | 2 |
| Streptococcus gallolyticus | 6 | 8 | 7678 | 0 | 18 |
| Leadbetterella byssophila | 95 | 88 | 70 | 1 | 116 |
| Syntrophus aciditrophicus | 192 | 430 | 425 | 6 | 569 |
| Synechococcus sp. CC9902 | 26 | 84 | 88 | 1 | 123 |
| Deinococcus ficus | 0 | 0 | 0 | 0 | 0 |
| Sulfurimonas paralvinellae | 6 | 33 | 36 | 0 | 33 |
| Streptomyces sp. F2 | 3 | 88 | 70 | 0 | 97 |
| Nostoc sp. PCC 7107 | 33 | 193 | 88 | 2 | 243 |
| Mycolicibacterium rufum | 2709 | 7621 | 5277 | 44 | 12255 |
| Gluconobacter albidus | 245 | 320 | 242 | 2 | 461 |
| Bradyrhizobium sp. WSM471 | 0 | 0 | 7314 | 0 | 7209 |
| Streptomyces sp. F11 | 1 | 5 | 21 | 23 | 554 |
| Streptomyces sp. FQ1 | 0 | 1 | 1 | 0 | 0 |
| Brevibacillus sp. WF146 | 719 | 1166 | 1230 | 6 | 1456 |
| Pseudomonas otitidis | 902 | 1550 | 1546 | 13 | 1820 |
| Streptomyces durmitorensis | 2897 | 2321 | 5177 | 363 | 9415 |
| Formosa agariphila | 1 | 15 | 19 | 0 | 21 |
| Neoasaia chiangmaiensis | 449 | 619 | 501 | 49 | 848 |
| Photobacterium ganghwense | 82 | 70 | 66 | 9 | 64 |
| Cyclobacterium amurskyense | 0 | 0 | 0 | 0 | 0 |
| Maribacter dokdonensis | 1 | 30 | 20 | 0 | 16 |
| Synechococcus sp. JA-3-3Ab | 152 | 614 | 674 | 3 | 742 |
| Synechococcus sp. JA-2-3B'a(2-13) | 293 | 384 | 310 | 5 | 587 |
| Pseudomonas moraviensis | 359 | 678 | 560 | 37 | 947 |
| Pseudoduganella albidiflava | 1535 | 2452 | 2609 | 18 | 5703 |
| Pseudoduganella plicata | 1448 | 2068 | 1870 | 13 | 3717 |
| Pseudoduganella lutea | 1069 | 2073 | 1652 | 0 | 3727 |
| Corynebacterium cyclohexanicum | 1263 | 1583 | 2777 | 88 | 2871 |
| Dietzia kunjamensis | 1103 | 321 | 1519 | 0 | 0 |
| Nonlabens tegetincola | 19 | 10 | 7 | 0 | 58 |
| Dokdonella koreensis | 2874 | 3510 | 3921 | 64 | 3747 |
| Paenibacillus sp. JDR-2 | 0 | 0 | 0 | 0 | 0 |
| Mesoplasma coleopterae | 0 | 0 | 0 | 0 | 0 |
| Spiroplasma tabanidicola | 3 | 2 | 1 | 0 | 2 |
| Bacillus infantis | 82 | 140 | 186 | 0 | 183 |
| Dokdonia donghaensis | 5 | 21 | 17 | 1 | 54 |
| Polaribacter dokdonensis | 0 | 12 | 12 | 0 | 5 |
| Burkholderia sp. M701 | 15 | 52 | 38 | 1 | 46 |
| Neisseria animaloris | 72 | 98 | 77 | 9 | 118 |
| Neisseria zoodegmatis | 21 | 36 | 41 | 0 | 30 |
| Paraglaciecola psychrophila | 61 | 187 | 52 | 4 | 38 |
| Pseudoalteromonas sp. A22 | 0 | 29 | 142 | 0 | 91 |
| Nonlabens dokdonensis | 0 | 0 | 0 | 0 | 0 |
| Deinococcus aquaticus | 0 | 0 | 0 | 0 | 0 |
| Parabacteroides goldsteinii | 0 | 0 | 0 | 0 | 0 |
| Alistipes onderdonkii | 463 | 1190 | 1083 | 12 | 1378 |
| Alistipes shahii | 385 | 695 | 639 | 37 | 1043 |
| Bacteroides intestinalis | 8 | 26 | 46 | 0 | 37 |
| Marinobacter psychrophilus | 49 | 90 | 65 | 12 | 94 |
| Psychrobacter cryohalolentis | 0 | 20 | 38 | 0 | 36 |
| Nonlabens spongiae | 6 | 143 | 42 | 0 | 151 |
| Pseudolabrys taiwanensis | 14500 | 6301 | 6830 | 173 | 12671 |
| Sphingobium indicum | 1108 | 1208 | 1334 | 36 | 1960 |
| Clostridium drakei | 0 | 0 | 0 | 0 | 0 |
| Larkinella insperata | 220 | 900 | 688 | 1 | 1000 |
| Shewanella xiamenensis | 0 | 0 | 0 | 0 | 0 |
| Truepera radiovictrix | 833 | 1514 | 1711 | 30 | 2502 |
| Exiguobacterium sibiricum | 84 | 182 | 80 | 44 | 105 |
| Aquitalea magnusonii | 0 | 0 | 0 | 0 | 0 |
| Pedobacter suwonensis | 94 | 145 | 243 | 1 | 261 |
| Enterocloster asparagiformis | 369 | 562 | 527 | 4 | 894 |
| Providencia heimbachae | 7 | 125 | 33 | 1 | 22 |
| Providencia vermicola | 0 | 0 | 0 | 0 | 0 |
| Saccharopolyspora pogona | 7353 | 2724 | 7919 | 368 | 10195 |
| Rhodococcus qingshengii | 1763 | 1817 | 2625 | 42 | 3955 |
| Psychrobacter arcticus | 13 | 13 | 14 | 4 | 22 |
| Salinivibrio proteolyticus | 61 | 39 | 30 | 0 | 30 |
| Methylobacterium oryzae | 1884 | 2664 | 1984 | 0 | 2711 |
| Sphingosinicella microcystinivorans | 6225 | 6948 | 7282 | 83 | 9806 |
| Sphingobium fuliginis | 0 | 0 | 0 | 0 | 0 |
| Thermovirga lienii | 18 | 117 | 109 | 11 | 205 |
| Candidatus Karelsulcia muelleri | 28 | 48 | 23 | 1 | 63 |
| Pedobacter roseus | 172 | 426 | 441 | 1 | 448 |
| Oenococcus kitaharae | 0 | 11 | 10 | 0 | 16 |
| Gordonia sp. KTR9 | 1125 | 1350 | 1800 | 141 | 3209 |
| Rickettsia endosymbiont of Bemisia tabaci | 15 | 13 | 30 | 0 | 6 |
| Erwinia tasmaniensis | 52 | 198 | 169 | 0 | 262 |
| Polymorphospora rubra | 4513 | 3256 | 11016 | 612 | 14524 |
| Sulfobacillus thermotolerans | 1 | 10 | 2 | 0 | 11 |
| Candidatus Contubernalis alkalaceticum | 14 | 79 | 68 | 2 | 92 |
| Mycobacterium colombiense | 1177 | 2491 | 1960 | 101 | 3993 |
| Pseudoalteromonas viridis | 45 | 154 | 91 | 2 | 285 |
| Desulfosporosinus youngiae | 45 | 69 | 85 | 1 | 1710 |
| Criblamydia sequanensis | 54 | 340 | 21 | 0 | 18 |
| Exiguobacterium mexicanum | 72 | 309 | 281 | 0 | 262 |
| Rhodopseudomonas sp. SK50-23 | 4547 | 2218 | 1828 | 81 | 2278 |
| Actinoalloteichus hymeniacidonis | 4031 | 1647 | 4413 | 68 | 4291 |
| Herbaspirillum hiltneri | 808 | 1042 | 960 | 44 | 1984 |
| Burkholderia oklahomensis | 800 | 1090 | 966 | 21 | 1165 |
| Parashewanella spongiae | 13 | 26 | 21 | 5 | 47 |
| Costertonia aggregata | 23 | 66 | 27 | 0 | 31 |
| Empedobacter falsenii | 0 | 0 | 0 | 0 | 0 |
| Microbacterium terricola | 1239 | 1609 | 2467 | 60 | 2678 |
| Solidesulfovibrio carbinoliphilus | 669 | 1407 | 1315 | 23 | 1755 |
| Geobacter pickeringii | 1225 | 2217 | 2164 | 56 | 2644 |
| Arthrobacter sp. 31.31 | 0 | 0 | 10 | 0 | 15 |
| Arthrobacter sp. J3.37 | 0 | 12 | 8 | 0 | 26 |
| Arthrobacter sp. J3.40 | 0 | 20 | 43 | 0 | 144 |
| Arthrobacter sp. J3.49 | 0 | 7 | 15 | 0 | 191 |
| Zobellella denitrificans | 856 | 1003 | 1188 | 25 | 1544 |
| Streptomyces davaonensis | 2062 | 1586 | 3131 | 131 | 4163 |
| Rhizobium favelukesii | 474 | 492 | 706 | 105 | 121 |
| Streptomyces sp. FR1 | 28 | 44 | 65 | 6 | 118 |
| Alkalilimnicola ehrlichii | 965 | 1260 | 1583 | 60 | 1951 |
| Geotalea uraniireducens | 727 | 1606 | 1217 | 0 | 1589 |
| Xenorhabdus doucetiae | 0 | 0 | 0 | 0 | 0 |
| Xenorhabdus hominickii | 58 | 69 | 63 | 6 | 50 |
| Shewanella sp. W3-18-1 | 0 | 24 | 0 | 0 | 0 |
| Shinella zoogloeoides | 5448 | 4776 | 4936 | 69 | 6698 |
| Bacillus sp. Y1 | 18 | 38 | 60 | 0 | 41 |
| Mycoplasma sp. Ms02 | 0 | 1 | 0 | 0 | 1 |
| Mycoplasma nasistruthionis | 0 | 2 | 0 | 0 | 1 |
| Niastella koreensis | 1166 | 1095 | 728 | 0 | 1027 |
| Amphritea atlantica | 73 | 159 | 131 | 22 | 176 |
| Streptomyces sp. Tu6071 | 803 | 448 | 1101 | 10 | 740 |
| Chitinimonas koreensis | 2836 | 3601 | 4027 | 46 | 5443 |
| Heliorestis convoluta | 91 | 170 | 64 | 3 | 94 |
| Micromonospora chokoriensis | 2638 | 1779 | 5464 | 189 | 5126 |
| Micromonospora coxensis | 3521 | 2701 | 6414 | 436 | 9171 |
| Phocaeicola dorei | 67 | 86 | 91 | 0 | 161 |
| Enterococcus lactis | 0 | 0 | 0 | 0 | 28 |
| Psychromonas ingrahamii | 75 | 90 | 26 | 5 | 55 |
| Roseiflexus sp. RS-1 | 948 | 1910 | 1519 | 24 | 1858 |
| Brevibacterium oceani | 366 | 456 | 744 | 21 | 1044 |
| Acidovorax sp. KKS102 | 0 | 0 | 0 | 0 | 0 |
| Pseudomonas extremaustralis | 0 | 0 | 0 | 0 | 0 |
| Shewanella loihica | 30 | 69 | 89 | 0 | 72 |
| Microbulbifer sp. A4B17 | 86 | 150 | 136 | 5 | 127 |
| Methylotenera mobilis | 28 | 87 | 70 | 1 | 77 |
| Clostridium tagluense | 56 | 46 | 34 | 0 | 49 |
| Streptococcus pseudoporcinus | 13 | 17 | 13 | 0 | 25 |
| Desulfoglaeba alkanexedens | 512 | 1434 | 1013 | 5 | 1344 |
| Altererythrobacter epoxidivorans | 0 | 0 | 0 | 0 | 0 |
| Pseudarthrobacter phenanthrenivorans | 847 | 1384 | 1845 | 57 | 2994 |
| Streptomyces vietnamensis | 2445 | 2134 | 3626 | 178 | 5568 |
| Candidatus Protochlamydia amoebophila | 121 | 220 | 108 | 4 | 108 |
| Spiroplasma cantharicola | 0 | 0 | 0 | 0 | 0 |
| Sphingomonas ginsengisoli An et al. 2013 | 1297 | 1469 | 877 | 0 | 2045 |
| Comamonas thiooxydans | 0 | 0 | 0 | 0 | 0 |
| Streptomyces sp. 44030 | 1 | 2 | 3 | 0 | 4 |
| Streptomyces sp. 44414 | 0 | 2 | 0 | 0 | 0 |
| Verminephrobacter eiseniae | 1023 | 1124 | 1221 | 0 | 1598 |
| Granulibacter bethesdensis | 526 | 917 | 768 | 21 | 1008 |
| Pelosinus fermentans | 50 | 78 | 98 | 17 | 170 |
| Paenibacillus sabinae | 268 | 299 | 322 | 2 | 457 |
| Sulfurospirillum cavolei | 0 | 0 | 0 | 0 | 0 |
| Yersinia similis | 27 | 24 | 28 | 0 | 27 |
| Microbacterium sp. XT11 | 572 | 848 | 1259 | 116 | 1540 |
| Ureibacillus thermophilus | 43 | 27 | 36 | 0 | 54 |
| Flammeovirga yaeyamensis | 3 | 16 | 33 | 0 | 65 |
| Blattabacterium sp. (Blatta orientalis) | 0 | 5 | 10 | 0 | 6 |
| Cupriavidus malaysiensis | 970 | 1079 | 903 | 4 | 1709 |
| Janthinobacterium svalbardensis | 245 | 478 | 484 | 0 | 3643 |
| Oceanidesulfovibrio marinus | 786 | 1423 | 1359 | 17 | 2088 |
| Brucella pseudintermedia | 0 | 0 | 0 | 0 | 0 |
| Gallionella capsiferriformans | 0 | 0 | 0 | 0 | 0 |
| Mycolicibacterium pallens | 3122 | 6946 | 6506 | 0 | 13301 |
| Mycolicibacterium rutilum | 4566 | 15163 | 9761 | 49 | 25982 |
| Aureimonas altamirensis | 1345 | 1473 | 1420 | 12 | 1943 |
| Microbacterium pygmaeum | 774 | 1217 | 1740 | 46 | 2306 |
| Arenimonas daejeonensis | 1121 | 1929 | 1828 | 0 | 659 |
| Gottfriedia acidiceleris | 0 | 0 | 0 | 0 | 0 |
| Sphingobacterium daejeonense | 0 | 0 | 0 | 0 | 0 |
| Bacteroides xylanisolvens | 57 | 134 | 177 | 0 | 221 |
| Isoptericola dokdonensis | 1800 | 2018 | 3336 | 98 | 3914 |
| Corynebacterium sp. L2-79-05 | 0 | 0 | 0 | 0 | 0 |
| Nocardiopsis sp. 90127 | 0 | 0 | 0 | 0 | 7 |
| Paenibacillus sonchi | 79 | 134 | 131 | 24 | 171 |
| Flammeovirga kamogawensis | 0 | 0 | 0 | 0 | 0 |
| Rivularia sp. PCC 7116 | 10 | 155 | 104 | 0 | 155 |
| Campylobacter cuniculorum | 0 | 0 | 0 | 0 | 0 |
| Methylobacterium tardum | 1723 | 1484 | 1609 | 17 | 1470 |
| Aminobacter sp. MSH1 | 7693 | 4768 | 4911 | 75 | 5437 |
| Synechococcus sp. PCC 7003 | 48 | 128 | 100 | 0 | 202 |
| Synechococcus sp. PCC 73109 | 29 | 90 | 15 | 0 | 50 |
| Loigolactobacillus backii | 23 | 33 | 28 | 0 | 85 |
| Janthinobacterium sp. Marseille | 356 | 637 | 476 | 0 | 1229 |
| Paenibacillus cellulosilyticus | 0 | 0 | 0 | 0 | 0 |
| Ensifer mexicanus | 1574 | 2011 | 1700 | 21 | 2310 |
| Natranaerobius thermophilus | 37 | 73 | 39 | 0 | 53 |
| Micrococcus sp. A7 | 0 | 0 | 6 | 0 | 0 |
| Halotalea alkalilenta | 718 | 1218 | 1416 | 11 | 1681 |
| Paradesulfovibrio bizertensis | 113 | 190 | 181 | 0 | 225 |
| Phocaeicola salanitronis | 71 | 185 | 203 | 2 | 327 |
| Thermobacillus composti | 770 | 1796 | 1361 | 9 | 1828 |
| Cyanobacterium aponinum | 23 | 164 | 70 | 0 | 70 |
| Streptomyces bingchenggensis | 21433 | 2498 | 16280 | 0 | 6088 |
| Vibrio sinaloensis | 0 | 0 | 0 | 0 | 0 |
| Rhizobium pseudoryzae | 1649 | 1195 | 1151 | 15 | 1504 |
| Pseudomonas protegens | 904 | 1753 | 3294 | 142 | 2025 |
| Thermocrinis minervae | 60 | 104 | 44 | 0 | 66 |
| Marinomonas arctica | 0 | 0 | 0 | 0 | 0 |
| Cycloclasticus sp. P1 | 0 | 36 | 38 | 5 | 43 |
| Candidatus Ruthia magnifica | 18 | 12 | 5 | 0 | 4 |
| Terribacillus goriensis | 14 | 25 | 28 | 0 | 59 |
| Sphingomonas sediminicola | 992 | 1165 | 896 | 46 | 2625 |
| Moraxella bovoculi | 36 | 97 | 25 | 0 | 78 |
| Mycobacterium seoulense | 1693 | 2731 | 1938 | 45 | 4050 |
| Phocaeicola coprophilus | 0 | 0 | 0 | 0 | 0 |
| Nitratiruptor sp. SB155-2 | 11 | 29 | 53 | 0 | 75 |
| Sulfurovum sp. NBC37-1 | 5 | 31 | 52 | 0 | 79 |
| Parabacteroides johnsonii | 13 | 175 | 95 | 0 | 230 |
| Kocuria turfanensis | 211 | 508 | 971 | 56 | 1254 |
| Roseibium alexandrii | 738 | 728 | 607 | 1 | 799 |
| Algoriphagus machipongonensis | 28 | 45 | 43 | 0 | 48 |
| Mycobacterium crocinum | 1884 | 2715 | 2539 | 97 | 5202 |
| Bartonella australis | 38 | 37 | 41 | 0 | 26 |
| Pontibacter akesuensis | 0 | 0 | 0 | 0 | 0 |
| Candidatus Protochlamydia naegleriophila | 0 | 0 | 0 | 0 | 0 |
| Wenxinia marina | 3 | 16 | 34 | 1 | 41 |
| Echinicola vietnamensis | 49 | 109 | 103 | 12 | 162 |
| Actinocatenispora sera | 3773 | 2730 | 7080 | 144 | 6946 |
| Ehrlichia sp. HF | 2 | 1 | 0 | 0 | 0 |
| Roseovarius sp. TM1035 | 0 | 0 | 0 | 0 | 0 |
| Methylocystis heyeri | 1541 | 1649 | 1434 | 2 | 2085 |
| Alicyclobacillus fastidiosus | 188 | 450 | 360 | 3 | 424 |
| Companilactobacillus crustorum | 2 | 4 | 4 | 0 | 5 |
| Inhella inkyongensis | 511 | 719 | 717 | 0 | 657 |
| Terriglobus roseus | 1291 | 4231 | 3313 | 42 | 3902 |
| Congregibacter litoralis | 466 | 671 | 691 | 12 | 887 |
| Candidatus Endoriftia persephone | 462 | 647 | 607 | 10 | 1007 |
| Porphyromonas crevioricanis | 26 | 103 | 28 | 2 | 29 |
| Pseudoalteromonas arctica | 1 | 11 | 9 | 0 | 18 |
| Chromobacterium haemolyticum | 613 | 865 | 979 | 0 | 1108 |
| Clostridium taeniosporum | 0 | 0 | 0 | 0 | 0 |
| Pseudomonas reinekei | 0 | 0 | 0 | 0 | 0 |
| Phytobacter diazotrophicus | 10 | 129 | 72 | 0 | 184 |
| Coraliomargarita akajimensis | 117 | 541 | 314 | 0 | 386 |
| Thioalkalivibrio sp. K90mix | 742 | 1235 | 1262 | 20 | 1567 |
| Sphingomonas sanxanigenens | 2925 | 3971 | 2643 | 0 | 5367 |
| Barnesiella viscericola | 71 | 453 | 419 | 0 | 557 |
| Mucilaginibacter daejeonensis | 107 | 224 | 214 | 0 | 364 |
| Mucilaginibacter ginsenosidivorans | 0 | 0 | 0 | 0 | 0 |
| Mycobacterium shinjukuense | 1679 | 3645 | 2684 | 28 | 4225 |
| Vibrio rhizosphaerae | 13 | 176 | 89 | 2 | 91 |
| Zunongwangia profunda | 55 | 69 | 56 | 4 | 58 |
| Candidatus Phytoplasma rubi | 0 | 2 | 8 | 0 | 6 |
| Tessaracoccus flavescens | 1590 | 1366 | 2411 | 92 | 2283 |
| Thermoanaerobacter sp. X514 | 27 | 0 | 38 | 0 | 0 |
| Agrococcus jejuensis | 1544 | 2048 | 2765 | 61 | 2848 |
| Paludibacterium yongneupense | 482 | 962 | 831 | 0 | 1200 |
| Streptococcus merionis | 11 | 18 | 10 | 0 | 24 |
| Pontibacter korlensis | 59 | 266 | 155 | 7 | 209 |
| Devosia ginsengisoli | 1633 | 1545 | 1275 | 21 | 1573 |
| Undibacterium parvum | 0 | 0 | 0 | 0 | 0 |
| Candidatus Riesia pediculicola | 1 | 2 | 2 | 1 | 5 |
| Nocardioides daphniae | 1491 | 2180 | 3112 | 113 | 2180 |
| Halobacillus mangrovi | 15 | 56 | 54 | 0 | 86 |
| Virgibacillus sp. SK37 | 0 | 42 | 5 | 0 | 62 |
| Shewanella piezotolerans | 3 | 59 | 46 | 1 | 51 |
| Nonomuraea coxensis | 7467 | 4657 | 38757 | 10967 | 163349 |
| Azospira restricta | 2941 | 4208 | 5005 | 101 | 9076 |
| Micrococcus sp. A1 | 9 | 56 | 18 | 0 | 6 |
| Anaeromyxobacter sp. Fw109-5 | 3920 | 7469 | 8152 | 82 | 9250 |
| Streptomyces sp. HK1 | 0 | 0 | 18 | 0 | 6 |
| Alicyclobacillus acidocaldarius | 1002 | 1963 | 2029 | 72 | 2801 |
| Thiomicrospira microaerophila | 11 | 56 | 53 | 34 | 78 |
| Aquisphaera giovannonii | 8663 | 45643 | 22802 | 295 | 33290 |
| Sphingobium sp. MI1205 | 2233 | 2003 | 1946 | 31 | 2993 |
| Streptomyces xiamenensis | 938 | 953 | 1605 | 118 | 3257 |
| Leptospira kmetyi | 28 | 113 | 116 | 0 | 249 |
| Flavobacterium sp. KI723T1 | 116 | 11 | 72 | 0 | 19 |
| Treponema pedis | 20 | 117 | 25 | 0 | 45 |
| Coprococcus comes | 3 | 302 | 30 | 0 | 32 |
| Gramella forsetii | 48 | 107 | 38 | 0 | 82 |
| Faecalibacterium duncaniae | 261 | 158 | 162 | 0 | 226 |
| Clostridium sp. M62/1 | 111 | 226 | 249 | 5 | 180 |
| Celerinatantimonas diazotrophica | 29 | 135 | 33 | 19 | 72 |
| Priestia aryabhattai | 0 | 0 | 0 | 0 | 0 |
| Thermosulfidibacter takaii | 51 | 145 | 118 | 7 | 240 |
| Microterricola viridarii | 2503 | 3050 | 4061 | 69 | 4156 |
| Mycetohabitans rhizoxinica | 445 | 804 | 684 | 0 | 1063 |
| Candidatus Vesicomyosocius okutanii | 7 | 7 | 10 | 0 | 4 |
| Vibrio rarus | 9 | 5 | 14 | 1 | 15 |
| Cronobacter dublinensis | 0 | 0 | 0 | 0 | 0 |
| Cronobacter muytjensii | 86 | 121 | 149 | 2 | 242 |
| Cronobacter malonaticus | 158 | 172 | 207 | 0 | 163 |
| Caldimonas brevitalea | 1614 | 2797 | 2447 | 41 | 2966 |
| Caldicellulosiruptor hydrothermalis | 1 | 5 | 1 | 0 | 3 |
| Caldicellulosiruptor kronotskyensis | 3 | 11 | 2 | 0 | 4 |
| Paenibacillus donghaensis | 186 | 344 | 258 | 12 | 449 |
| Planococcus donghaensis | 13 | 12 | 23 | 0 | 44 |
| Actinopolyspora erythraea | 2350 | 1337 | 3056 | 100 | 3517 |
| Arthrobacter sp. AK-1 | 25 | 13 | 7 | 1 | 30 |
| Pseudoxanthomonas spadix | 1266 | 1293 | 1105 | 13 | 1085 |
| Rhodanobacter thiooxydans | 1544 | 1755 | 1731 | 59 | 1671 |
| Ferrovum myxofaciens | 277 | 610 | 384 | 9 | 656 |
| Burkholderia sp. KJ006 | 0 | 0 | 0 | 0 | 0 |
| Paenisporosarcina antarctica | 25 | 175 | 147 | 0 | 174 |
| Enterococcus thailandicus | 0 | 0 | 0 | 0 | 0 |
| Neptunomonas japonica | 46 | 55 | 38 | 2 | 60 |
| Blautia wexlerae | 0 | 0 | 0 | 0 | 0 |
| Azoarcus olearius | 1903 | 2552 | 2983 | 37 | 4416 |
| Litorivicinus lipolyticus | 191 | 222 | 273 | 11 | 347 |
| Marinobacter salsuginis | 253 | 395 | 299 | 5 | 425 |
| Yersinia massiliensis | 2 | 41 | 39 | 0 | 106 |
| Brucella pseudogrignonensis | 234 | 366 | 306 | 0 | 787 |
| Nocardioides marinisabuli | 2515 | 3840 | 4936 | 114 | 4714 |
| Jiangella alkaliphila | 6505 | 5578 | 11730 | 405 | 12139 |
| Marinobacterium rhizophilum | 609 | 770 | 706 | 34 | 846 |
| Paraburkholderia bryophila | 983 | 1545 | 1408 | 25 | 1517 |
| Paraburkholderia megapolitana | 914 | 1175 | 1049 | 11 | 1052 |
| Chryseobacterium oranimense | 5 | 39 | 30 | 0 | 48 |
| Ectothiorhodospira haloalkaliphila | 469 | 774 | 716 | 23 | 946 |
| Peribacillus butanolivorans | 0 | 0 | 0 | 0 | 0 |
| Mucilaginibacter paludis | 115 | 232 | 180 | 1 | 218 |
| Elusimicrobium minutum | 45 | 124 | 43 | 3 | 65 |
| Streptomyces deccanensis | 1944 | 1794 | 3663 | 118 | 5020 |
| Sphingomonas insulae | 767 | 1165 | 1131 | 29 | 1445 |
| Thiomonas arsenitoxydans | 586 | 961 | 816 | 0 | 1210 |
| Methylobacterium sp. 4-46 | 3916 | 4268 | 4199 | 185 | 6337 |
| Desulfurivibrio alkaliphilus | 296 | 715 | 708 | 3 | 766 |
| Candidatus Riesia pediculischaeffi | 11 | 5 | 3 | 0 | 2 |
| Sinorhizobium sp. M14 | 568 | 527 | 172 | 0 | 114 |
| Pedobacter steynii | 0 | 0 | 0 | 0 | 0 |
| Nitratireductor kimnyeongensis | 716 | 908 | 786 | 4 | 924 |
| Methylacidiphilum kamchatkense | 2 | 70 | 51 | 0 | 44 |
| Acetobacter ghanensis | 225 | 170 | 167 | 0 | 245 |
| Deinococcus peraridilitoris | 419 | 860 | 1102 | 17 | 1155 |
| Anaerocolumna cellulosilytica | 10 | 123 | 29 | 0 | 18 |
| Aminipila butyrica | 13 | 65 | 45 | 0 | 57 |
| Nocardioides mesophilus | 2807 | 4225 | 6068 | 292 | 8362 |
| Variovorax sp. PBS-H4 | 3613 | 4458 | 4316 | 25 | 5334 |
| Variovorax sp. PBL-H6 | 3921 | 4939 | 4927 | 0 | 6209 |
| Hydrogenophaga sp. PBL-H3 | 1467 | 2656 | 2709 | 0 | 3026 |
| Variovorax sp. PBL-E5 | 3123 | 3399 | 3882 | 81 | 4491 |
| Lysobacter capsici | 6174 | 5820 | 3919 | 171 | 5008 |
| Microbulbifer celer | 0 | 0 | 0 | 0 | 0 |
| Vibrio porteresiae | 25 | 64 | 56 | 1 | 62 |
| Variovorax boronicumulans | 1236 | 1773 | 1690 | 0 | 1336 |
| Lysinibacillus parviboronicapiens | 0 | 118 | 28 | 0 | 32 |
| Granulosicoccus antarcticus | 379 | 415 | 452 | 5 | 762 |
| Megamonas funiformis | 21 | 23 | 37 | 0 | 23 |
| Pseudomonas marincola | 230 | 609 | 429 | 32 | 678 |
| Ruminococcus gauvreauii | 43 | 189 | 261 | 0 | 216 |
| Corynebacterium timonense | 364 | 507 | 757 | 2 | 1137 |
| Xanthomonas perforans | 177 | 1090 | 2411 | 0 | 199 |
| Pedococcus dokdonensis | 1730 | 2468 | 4099 | 156 | 5139 |
| Streptomyces sp. CNQ-509 | 1923 | 1112 | 2281 | 143 | 3155 |
| Brucella microti | 0 | 16 | 17 | 0 | 16 |
| Chelatococcus daeguensis | 3027 | 2193 | 2483 | 88 | 3036 |
| Mycolicibacterium insubricum | 1754 | 3212 | 2982 | 97 | 5111 |
| Rickettsia endosymbiont of Ixodes scapularis | 31 | 19 | 7 | 0 | 4 |
| Rhodoplanes piscinae | 5 | 5 | 11 | 0 | 15 |
| Pseudonocardia sp. AL041005-10 | 2452 | 1140 | 3539 | 4 | 1426 |
| Pandoraea thiooxydans | 880 | 1261 | 1132 | 31 | 1703 |
| Dyella thiooxydans | 1885 | 2108 | 2176 | 15 | 2537 |
| Thermus sp. 4C | 0 | 0 | 9 | 0 | 0 |
| Vibrio celticus | 0 | 0 | 0 | 0 | 0 |
| Vibrio artabrorum | 1 | 12 | 37 | 6 | 37 |
| Adlercreutzia equolifaciens | 407 | 613 | 830 | 21 | 952 |
| Nostoc sphaeroides | 101 | 213 | 157 | 0 | 315 |
| Niabella soli | 259 | 329 | 261 | 1 | 345 |
| Acetobacter senegalensis | 38 | 95 | 44 | 1 | 141 |
| Kocuria flava | 955 | 981 | 1720 | 45 | 1185 |
| Arthrobacter sp. Chr15 | 0 | 2 | 8 | 0 | 5 |
| Anaeromyxobacter sp. K | 2872 | 5569 | 5706 | 49 | 7106 |
| Simiduia agarivorans | 264 | 271 | 243 | 9 | 485 |
| Dechloromonas sp. A34 | 1040 | 2070 | 1839 | 31 | 2223 |
| Ciceribacter selenitireducens | 35 | 0 | 33 | 0 | 156 |
| Nocardioides humi | 3988 | 4547 | 6163 | 186 | 5765 |
| Campylobacter canadensis | 2 | 3 | 4 | 0 | 4 |
| Peribacillus frigoritolerans | 371 | 1585 | 492 | 0 | 399 |
| Croceicoccus marinus | 1244 | 1625 | 1300 | 37 | 1892 |
| Nocardioides dokdonensis | 2354 | 3605 | 4409 | 229 | 5117 |
| Candidatus Magnetococcus massalia | 0 | 8 | 2 | 0 | 9 |
| Rhizobium rhizoryzae | 720 | 766 | 608 | 21 | 787 |
| Amphritea japonica | 11 | 90 | 73 | 14 | 83 |
| Vibrio sp. 23023 | 7 | 0 | 0 | 0 | 0 |
| Candidatus Nitrotoga arctica | 362 | 812 | 698 | 6 | 1427 |
| Desulfarculus baarsii | 781 | 1871 | 2225 | 36 | 2463 |
| Agromyces aureus | 3256 | 3742 | 5812 | 64 | 6401 |
| Lysobacter soli | 4101 | 3343 | 2980 | 7 | 2530 |
| Pseudobdellovibrio exovorus | 42 | 56 | 60 | 1 | 69 |
| Paracoccus aestuarii | 0 | 0 | 0 | 0 | 0 |
| Paraprevotella xylaniphila | 126 | 143 | 248 | 0 | 349 |
| Chthonomonas calidirosea | 160 | 488 | 618 | 11 | 729 |
| Maritalea myrionectae | 353 | 237 | 233 | 1 | 364 |
| Cellvibrio sp. KY-YJ-3 | 133 | 118 | 85 | 46 | 80 |
| Saccharopolyspora gloriosae | 6214 | 1939 | 6008 | 147 | 5712 |
| Nocardia terpenica | 4791 | 3896 | 6794 | 184 | 8296 |
| Xanthomonas euvesicatoria | 4041 | 55722 | 950 | 31 | 937 |
| Candidatus Cloacimonas acidaminovorans | 23 | 99 | 62 | 1 | 162 |
| Tolypothrix tenuis | 21 | 231 | 109 | 0 | 358 |
| Chloracidobacterium thermophilum | 22 | 3112 | 1862 | 0 | 1222 |
| Serratia nematodiphila | 65 | 65 | 73 | 0 | 59 |
| Planococcus glaciei | 102 | 239 | 152 | 0 | 152 |
| Flavobacterium anhuiense | 32 | 234 | 313 | 19 | 148 |
| Sphingobacterium siyangense | 19 | 34 | 44 | 0 | 73 |
| Mycobacterium noviomagense | 1355 | 3841 | 2727 | 65 | 5808 |
| Alkaliphilus oremlandii | 41 | 21 | 14 | 0 | 17 |
| Microbacterium binotii | 491 | 484 | 994 | 28 | 1248 |
| Bordetella flabilis | 1268 | 1659 | 1572 | 34 | 2400 |
| Bordetella genomosp. 6 | 615 | 400 | 430 | 7 | 615 |
| Bordetella bronchialis | 1728 | 1655 | 2213 | 19 | 2865 |
| Bordetella genomosp. 13 | 3529 | 2747 | 3893 | 14 | 4413 |
| Pseudodesulfovibrio tunisiensis | 241 | 609 | 583 | 6 | 983 |
| Streptomyces sp. Mg1 | 1246 | 1332 | 1871 | 176 | 2849 |
| Steroidobacter denitrificans | 15277 | 12564 | 6310 | 21 | 3899 |
| Acinetobacter septicus | 0 | 0 | 0 | 0 | 0 |
| Singulisphaera acidiphila | 19665 | 107917 | 42027 | 271 | 38033 |
| Ilumatobacter coccineus | 4825 | 2762 | 5147 | 57 | 4272 |
| Iamia majanohamensis | 7828 | 5886 | 14383 | 152 | 10825 |
| Liquorilactobacillus hordei | 37 | 11 | 9 | 11 | 15 |
| Mycobacterium stomatepiae | 1569 | 2773 | 2172 | 39 | 4078 |
| Pseudomonas taiwanensis | 214 | 63 | 404 | 0 | 379 |
| Pantoea anthophila | 0 | 0 | 0 | 0 | 0 |
| Pantoea deleyi | 231 | 292 | 272 | 5 | 411 |
| Pantoea eucalypti | 15 | 172 | 173 | 0 | 132 |
| Pantoea vagans | 422 | 705 | 530 | 21 | 734 |
| Gordonibacter pamelaeae | 358 | 781 | 854 | 0 | 829 |
| Candidatus Desulforudis audaxviator | 352 | 603 | 734 | 4 | 978 |
| Devriesea agamarum | 100 | 177 | 296 | 5 | 362 |
| Candidatus Purcelliella pentastirinorum | 10 | 5 | 14 | 0 | 8 |
| Nitrosococcus watsonii | 71 | 422 | 196 | 2 | 240 |
| Vibrio mangrovi | 14 | 60 | 39 | 3 | 49 |
| Halomonas beimenensis | 962 | 1037 | 1068 | 22 | 1549 |
| Rhodopseudomonas boonkerdii | 3355 | 2079 | 1630 | 44 | 1700 |
| Altererythrobacter ishigakiensis | 0 | 0 | 0 | 0 | 0 |
| Streptomyces milbemycinicus | 7511 | 2317 | 8302 | 0 | 3930 |
| Modestobacter marinus | 2933 | 3734 | 6190 | 246 | 7592 |
| Filimonas lacunae | 0 | 0 | 0 | 0 | 0 |
| Streptomyces sp. T12 | 2608 | 4147 | 4804 | 165 | 7754 |
| Calditerrivibrio nitroreducens | 29 | 57 | 51 | 0 | 102 |
| Micromonospora tulbaghiae | 2301 | 1908 | 4598 | 324 | 7599 |
| Nocardia wallacei | 3130 | 2661 | 4859 | 253 | 6585 |
| Mycobacterium lepromatosis | 214 | 367 | 245 | 9 | 582 |
| Myroides profundi | 2 | 9 | 6 | 0 | 6 |
| Acetobacter ascendens | 73 | 218 | 57 | 0 | 118 |
| Paenibacillus sp. Y412MC10 | 226 | 278 | 298 | 0 | 384 |
| Mycobacterium dioxanotrophicus | 3571 | 9318 | 6835 | 89 | 15251 |
| Oscillatoria nigro-viridis | 195 | 449 | 269 | 3 | 472 |
| Thermobifida halotolerans | 1750 | 1536 | 3588 | 660 | 11177 |
| Geoalkalibacter subterraneus | 480 | 1404 | 1202 | 12 | 1757 |
| Bacillus inaquosorum | 0 | 19 | 84 | 0 | 125 |
| Paenibacillus riograndensis | 108 | 155 | 145 | 0 | 176 |
| Sphingobium sp. YBL2 | 395 | 502 | 564 | 0 | 832 |
| Pelosinus sp. UFO1 | 41 | 94 | 55 | 0 | 73 |
| Thalassomonas actiniarum | 32 | 122 | 113 | 4 | 236 |
| Thalassomonas haliotis | 58 | 141 | 84 | 14 | 139 |
| Pseudomonas anuradhapurensis | 103 | 170 | 168 | 4 | 274 |
| Pseudomonas promysalinigenes | 0 | 0 | 0 | 0 | 0 |
| Bacillus sp. S3 | 16 | 136 | 107 | 0 | 100 |
| Mycobacterium riyadhense | 2038 | 5496 | 3473 | 64 | 7070 |
| Dialister succinatiphilus | 148 | 510 | 265 | 8 | 476 |
| Halopseudomonas xinjiangensis | 0 | 0 | 0 | 0 | 0 |
| Stenotrophomonas pavanii | 0 | 0 | 64 | 0 | 62 |
| Serratia sp. SCBI | 20 | 44 | 39 | 0 | 74 |
| Burkholderia latens | 0 | 0 | 0 | 0 | 0 |
| Campylobacter peloridis | 1 | 27 | 2 | 0 | 12 |
| Burkholderia arboris | 0 | 0 | 0 | 0 | 0 |
| Burkholderia seminalis | 0 | 0 | 0 | 0 | 0 |
| Pseudomonas chengduensis | 514 | 665 | 587 | 0 | 706 |
| Nosocomiicoccus ampullae | 2 | 15 | 7 | 0 | 12 |
| Marinobacter sp. BSs20148 | 104 | 138 | 149 | 8 | 218 |
| Marinomonas rhizomae | 19 | 20 | 34 | 13 | 48 |
| Marinomonas foliarum | 4 | 37 | 29 | 13 | 30 |
| Bacillus velezensis | 2290 | 2126 | 2670 | 68 | 3292 |
| Baekduia soli | 8333 | 7230 | 16603 | 170 | 18375 |
| Anseongella ginsenosidimutans | 133 | 355 | 266 | 2 | 361 |
| Arachidicoccus ginsenosidivorans | 35 | 107 | 98 | 0 | 123 |
| Campylobacter subantarcticus | 0 | 3 | 0 | 0 | 1 |
| Kosakonia oryzae | 0 | 0 | 0 | 0 | 0 |
| Hymenobacter tibetensis | 0 | 0 | 0 | 0 | 0 |
| Borreliella finlandensis | 0 | 0 | 0 | 0 | 0 |
| Tepidanaerobacter acetatoxydans | 22 | 57 | 45 | 0 | 41 |
| Dietzia timorensis | 1311 | 1248 | 2071 | 70 | 2609 |
| Deinococcus gobiensis | 741 | 1288 | 1317 | 26 | 1463 |
| Eggerthella sp. YY7918 | 298 | 411 | 419 | 0 | 404 |
| Aurantiacibacter gangjinensis | 0 | 0 | 0 | 0 | 0 |
| Rhodovastum atsumiense | 2960 | 3556 | 3206 | 46 | 4477 |
| Leisingera caerulea | 861 | 1187 | 1114 | 57 | 1259 |
| Halomonas sp. MS1 | 89 | 126 | 145 | 0 | 166 |
| Lactobacillus taiwanensis | 0 | 0 | 0 | 0 | 0 |
| Oceanimonas sp. GK1 | 376 | 564 | 519 | 2 | 681 |
| Candidatus Azobacteroides pseudotrichonymphae | 5 | 9 | 5 | 0 | 13 |
| Methylacidiphilum infernorum | 106 | 174 | 147 | 0 | 150 |
| Mycobacterium heraklionense | 1517 | 2446 | 1981 | 102 | 3710 |
| Vibrio azureus | 3 | 38 | 32 | 4 | 13 |
| Rufibacter tibetensis | 0 | 0 | 0 | 0 | 0 |
| Dictyoglomus turgidum | 38 | 51 | 57 | 0 | 48 |
| Bartonella sp. 1-1C | 2 | 3 | 0 | 0 | 13 |
| Micromonospora endophytica | 2132 | 1656 | 5056 | 320 | 6675 |
| Pseudomonas yamanorum | 0 | 0 | 0 | 0 | 0 |
| Muricauda lutaonensis | 133 | 124 | 106 | 0 | 109 |
| Providencia sneebia | 4 | 14 | 15 | 1 | 7 |
| Shewanella vesiculosa | 7 | 45 | 13 | 4 | 46 |
| Marinobacterium sediminicola | 0 | 0 | 0 | 0 | 0 |
| Mycoplasma anserisalpingitidis | 1 | 5 | 5 | 0 | 4 |
| Vagococcus teuberi | 0 | 0 | 0 | 0 | 0 |
| Sphingobium sp. RSMS | 0 | 0 | 0 | 0 | 0 |
| Paenibacillus urinalis | 75 | 143 | 134 | 0 | 163 |
| Thiocapsa bogorovii | 1334 | 2225 | 2019 | 26 | 2970 |
| Denitrificimonas caeni | 0 | 1228 | 8285 | 15470 | 2052 |
| Dyella terrae | 1016 | 951 | 891 | 76 | 1240 |
| Campylobacter avium | 2 | 4 | 4 | 0 | 3 |
| Saccharopolyspora rosea | 6523 | 2661 | 6920 | 215 | 7026 |
| Tetragenococcus osmophilus | 23 | 26 | 24 | 0 | 127 |
| Paenibacillus xylanexedens | 34 | 175 | 94 | 0 | 135 |
| Azospirillum thiophilum | 2161 | 1973 | 2358 | 50 | 3363 |
| Listeria marthii | 0 | 13 | 22 | 0 | 21 |
| Rhodoluna lacicola | 22 | 130 | 96 | 5 | 154 |
| Prauserella marina | 6047 | 2044 | 7022 | 202 | 6325 |
| Pelagibacterium halotolerans | 1838 | 1156 | 1017 | 19 | 1210 |
| Candidatus Rhodoluna planktonica | 0 | 0 | 0 | 0 | 0 |
| Mesorhizobium australicum | 1557 | 1409 | 1172 | 0 | 1549 |
| Mycoplasma struthionis | 0 | 0 | 0 | 0 | 0 |
| Stappia indica | 3423 | 3159 | 2799 | 121 | 3860 |
| Enterobacter mori | 592 | 583 | 787 | 0 | 653 |
| Roseovarius indicus | 1870 | 1662 | 2016 | 22 | 2335 |
| Saccharopolyspora endophytica | 1 | 0 | 0 | 0 | 2 |
| Butyricimonas virosa | 75 | 50 | 61 | 0 | 72 |
| Aliarcobacter thereius | 0 | 0 | 0 | 0 | 0 |
| Streptomyces silaceus | 0 | 1 | 0 | 0 | 0 |
| Oceanobacillus oncorhynchi | 0 | 0 | 0 | 0 | 0 |
| Paraoerskovia marina | 1252 | 1596 | 2249 | 53 | 2279 |
| Pseudomonas sp. LM8 | 0 | 1 | 0 | 0 | 0 |
| Dietzia lutea | 907 | 898 | 1582 | 10 | 1337 |
| Hafnia paralvei | 0 | 0 | 0 | 0 | 0 |
| Friedmanniella luteola | 2449 | 3393 | 5976 | 189 | 8834 |
| Microlunatus sagamiharensis | 2989 | 3671 | 5616 | 205 | 7044 |
| Hydrogenobaculum sp. HO | 25 | 0 | 65 | 10 | 78 |
| Phycisphaera mikurensis | 1664 | 3746 | 3096 | 64 | 4469 |
| Mycoplasma tauri | 0 | 3 | 1 | 0 | 3 |
| Francisella halioticida | 14 | 3 | 4 | 0 | 2 |
| Geobacillus sp. Y412MC52 | 0 | 29 | 75 | 0 | 70 |
| Aromatoleum aromaticum | 1885 | 2376 | 2293 | 35 | 3739 |
| Kosakonia arachidis | 0 | 0 | 0 | 0 | 0 |
| Mucilaginibacter gossypii | 111 | 117 | 108 | 2 | 123 |
| Vibrio gallaecicus | 37 | 14 | 35 | 2 | 22 |
| Vibrio breoganii | 89 | 132 | 129 | 3 | 129 |
| Streptomyces gilvosporeus | 1787 | 1426 | 2753 | 227 | 5579 |
| Acidovorax carolinensis | 1237 | 1496 | 1517 | 0 | 2125 |
| Candidatus Nitrosacidococcus tergens | 1 | 34 | 46 | 0 | 28 |
| Allochromatium tepidum | 1253 | 1734 | 1607 | 24 | 1843 |
| Pseudomonas toyotomiensis | 0 | 0 | 0 | 0 | 0 |
| Leptotrichia hongkongensis | 0 | 29 | 4 | 0 | 9 |
| Ensifer sp. T173 | 3954 | 2147 | 1660 | 0 | 2308 |
| Phyllobacterium sp. T1018 | 1046 | 1216 | 1020 | 7 | 1314 |
| Phyllobacterium sp. T1293 | 875 | 507 | 521 | 0 | 595 |
| Rhizobium sp. T136 | 1006 | 1090 | 818 | 0 | 573 |
| Polynucleobacter acidiphobus | 36 | 119 | 109 | 2 | 91 |
| Polynucleobacter difficilis | 89 | 137 | 80 | 1 | 111 |
| Bacteroides sp. D2 | 0 | 19 | 10 | 0 | 19 |
| Candidatus Liberibacter solanacearum | 15 | 8 | 19 | 3 | 23 |
| Brachybacterium saurashtrense | 683 | 690 | 1067 | 34 | 507 |
| Propionibacterium acidifaciens | 952 | 1370 | 1868 | 11 | 1755 |
| Pseudomonas benzenivorans | 518 | 1000 | 746 | 29 | 1030 |
| Chryseobacterium piperi | 29 | 30 | 21 | 0 | 21 |
| Shewanella chilikensis | 40 | 77 | 123 | 42 | 103 |
| Mycobacterium mantenii | 1472 | 3333 | 2335 | 20 | 4927 |
| Sphingobacterium psychroaquaticum | 6 | 62 | 38 | 0 | 58 |
| Mameliella alba | 807 | 878 | 730 | 0 | 1051 |
| Bacillus safensis | 41 | 199 | 117 | 1 | 142 |
| Pseudoalteromonas sp. CF6-2 | 0 | 46 | 7 | 0 | 12 |
| Paenibacillus cellulositrophicus | 207 | 621 | 313 | 0 | 512 |
| Ferruginibacter lapsinanis | 223 | 245 | 206 | 0 | 205 |
| Sphingomonas hankookensis | 1142 | 1911 | 1528 | 53 | 2301 |
| Spirosoma rigui | 225 | 778 | 524 | 2 | 624 |
| Roseicitreum antarcticum | 0 | 0 | 0 | 0 | 0 |
| Luteibacter anthropi | 767 | 1240 | 1099 | 2 | 999 |
| Streptomyces sp. SM17 | 292 | 343 | 1016 | 0 | 928 |
| Micrococcus yunnanensis | 0 | 0 | 0 | 0 | 0 |
| Dickeya sp. NCPPB 3274 | 119 | 106 | 98 | 2 | 76 |
| Dickeya poaceiphila | 0 | 0 | 0 | 0 | 0 |
| Methylovirgula ligni | 1915 | 1475 | 1493 | 4 | 1789 |
| Pseudoalteromonas lipolytica | 15 | 29 | 50 | 0 | 4 |
| Allobacillus halotolerans | 8 | 48 | 20 | 1 | 65 |
| Desulfosarcina alkanivorans | 540 | 1196 | 1489 | 17 | 1971 |
| Brucella pituitosa | 119 | 159 | 100 | 0 | 182 |
| Psychrobacter sp. G | 63 | 29 | 23 | 0 | 21 |
| Luteipulveratus mongoliensis | 2278 | 1997 | 3715 | 135 | 3869 |
| Corynebacterium mustelae | 91 | 76 | 142 | 6 | 345 |
| Leeuwenhoekiella palythoae | 8 | 25 | 13 | 0 | 23 |
| Francisella salina | 0 | 83 | 3 | 0 | 1 |
| Francisella uliginis | 1 | 5 | 5 | 0 | 6 |
| Candidatus Planktophila limnetica | 1 | 34 | 59 | 6 | 109 |
| Candidatus Hodgkinia cicadicola | 47 | 34 | 22 | 2 | 52 |
| Pandoraea oxalativorans | 657 | 879 | 976 | 13 | 1325 |
| Bradyrhizobium sp. C-145 | 1728 | 1884 | 1410 | 0 | 1084 |
| Gluconobacter sphaericus | 254 | 355 | 233 | 22 | 520 |
| Corynebacterium maris | 461 | 535 | 790 | 51 | 1274 |
| Sediminibacterium sp. TEGAF015 | 194 | 215 | 207 | 16 | 158 |
| Polynucleobacter necessarius | 0 | 0 | 0 | 0 | 0 |
| Polynucleobacter asymbioticus | 0 | 0 | 0 | 0 | 0 |
| Euhalothece natronophila | 16 | 22 | 47 | 0 | 82 |
| Mycobacterium paraterrae | 1049 | 2720 | 1985 | 31 | 3683 |
| Bacillus cytotoxicus | 18 | 21 | 40 | 2 | 54 |
| Bacillus sp. 3a | 0 | 7 | 0 | 0 | 5 |
| Microbacterium azadirachtae | 1192 | 1264 | 2066 | 15 | 2461 |
| Rhodanobacter glycinis | 1048 | 1206 | 1210 | 53 | 1532 |
| Thermoanaerobacter mathranii | 37 | 147 | 69 | 0 | 106 |
| Tenacibaculum jejuense | 8 | 13 | 34 | 0 | 35 |
| Synechococcus sp. KORDI-49 | 0 | 0 | 0 | 0 | 0 |
| Synechococcus sp. KORDI-52 | 143 | 187 | 199 | 1 | 203 |
| Thiohalobacter thiocyanaticus | 954 | 878 | 817 | 4 | 1108 |
| Pseudomonas chlororaphis | 6182 | 5286 | 5293 | 97 | 10272 |
| Prauserella muralis | 29 | 56 | 88 | 0 | 127 |
| Agromyces flavus | 2657 | 3015 | 4945 | 51 | 4787 |
| Prevotella fusca | 8 | 28 | 16 | 0 | 29 |
| Prevotella scopos | 0 | 15 | 14 | 0 | 4 |
| Alteromonas australica | 18 | 86 | 85 | 0 | 43 |
| Mycobacterium paraseoulense | 1424 | 2577 | 2086 | 25 | 4196 |
| Ignavibacterium album | 0 | 0 | 0 | 0 | 0 |
| Pantoea sp. At-9b | 202 | 196 | 291 | 0 | 256 |
| Mesorhizobium opportunistum | 2408 | 2200 | 1911 | 101 | 1874 |
| Allofrancisella guangzhouensis | 12 | 15 | 6 | 0 | 5 |
| Abyssogena phaseoliformis symbiont | 25 | 6 | 4 | 0 | 16 |
| Brenneria sp. EniD312 | 0 | 428 | 363 | 0 | 0 |
| Malaciobacter mytili | 14 | 9 | 20 | 0 | 20 |
| Parafannyhessea umbonata | 621 | 1272 | 1308 | 19 | 1237 |
| Pseudoalteromonas donghaensis | 0 | 55 | 30 | 0 | 10 |
| Francisella hispaniensis | 3 | 6 | 1 | 1 | 2 |
| Blattabacterium sp. (Blattella germanica) | 0 | 7 | 4 | 0 | 1 |
| Phytohabitans suffuscus | 7791 | 4879 | 15148 | 479 | 16399 |
| Alistipes indistinctus | 213 | 539 | 429 | 1 | 635 |
| Christensenella minuta | 89 | 313 | 270 | 1 | 438 |
| Phascolarctobacterium succinatutens | 39 | 162 | 124 | 0 | 156 |
| Sphingobium sp. SYK-6 | 1489 | 1776 | 1593 | 5 | 2183 |
| Rhodococcus sp. T9N | 0 | 17 | 0 | 0 | 27 |
| Nocardioides panacisoli | 2089 | 3304 | 3925 | 195 | 4232 |
| Brevibacterium sandarakinum | 0 | 0 | 0 | 0 | 0 |
| Microlunatus soli | 2140 | 2240 | 3671 | 77 | 4325 |
| Desulfovibrio sp. G11 | 108 | 370 | 339 | 5 | 590 |
| Saccharomonospora marina | 7669 | 2804 | 8558 | 230 | 7489 |
| Salisediminibacterium beveridgei | 54 | 228 | 72 | 0 | 170 |
| Aeromonas sanarellii | 182 | 256 | 425 | 10 | 418 |
| Vagococcus penaei | 2 | 11 | 8 | 0 | 1 |
| Candidatus Arsenophonus lipoptenae | 2 | 7 | 2 | 1 | 7 |
| Levilactobacillus koreensis | 0 | 0 | 0 | 0 | 0 |
| Bifidobacterium actinocoloniiforme | 64 | 280 | 336 | 2 | 383 |
| Pyramidobacter piscolens | 431 | 547 | 818 | 4 | 831 |
| Sphaerotilus sulfidivorans | 1351 | 1713 | 1789 | 0 | 1607 |
| Olleya aquimaris | 20 | 16 | 12 | 0 | 15 |
| Hoyosella subflava | 790 | 1148 | 1196 | 51 | 1869 |
| Neisseria zalophi | 21 | 69 | 38 | 0 | 88 |
| Pseudomonas seleniipraecipitans | 0 | 0 | 0 | 0 | 0 |
| Mycoplasma sp. Pen4 | 0 | 2 | 4 | 0 | 0 |
| Clostridium sp. DL-VIII | 36 | 16 | 8 | 0 | 10 |
| Pseudodesulfovibrio mercurii | 571 | 1211 | 1272 | 15 | 1670 |
| Tatumella morbirosei | 0 | 0 | 2 | 0 | 0 |
| Marmoricola scoriae | 2200 | 3269 | 4743 | 87 | 3773 |
| Mammaliicoccus stepanovicii | 27 | 10 | 8 | 0 | 15 |
| Paenalcaligenes hominis | 29 | 36 | 64 | 13 | 181 |
| Rhodococcus sp. C1 | 66 | 141 | 233 | 0 | 644 |
| Pseudomonas sp. T8 | 52 | 111 | 239 | 0 | 1169 |
| Paraurantiacibacter namhicola | 532 | 603 | 555 | 5 | 824 |
| Streptomyces sp. M2 | 567 | 927 | 1339 | 80 | 2079 |
| Enterobacter sp. YSU | 0 | 35 | 0 | 0 | 11 |
| Aeromonas rivuli | 301 | 423 | 458 | 18 | 532 |
| Rhizobium pusense | 2425 | 1882 | 1641 | 0 | 375 |
| Micromonospora sp. L5 | 2189 | 1924 | 5553 | 640 | 6968 |
| Pseudoalteromonas sp. 3J6 | 0 | 10 | 5 | 0 | 6 |
| Sphingobacterium hotanense | 61 | 81 | 54 | 0 | 88 |
| Anaerostipes hadrus | 0 | 0 | 0 | 0 | 0 |
| Actinoplanes sp. N902-109 | 4547 | 3601 | 8964 | 385 | 10673 |
| Sulfuricella denitrificans | 532 | 1090 | 1033 | 13 | 1778 |
| Vibrio paracholerae | 0 | 21 | 33 | 0 | 104 |
| endosymbiont of Euscepes postfasciatus | 0 | 0 | 1 | 0 | 1 |
| Cardinium endosymbiont of Sogatella furcifera | 0 | 48 | 21 | 0 | 12 |
| Fibrella aestuarina | 461 | 974 | 868 | 12 | 1540 |
| Kosmotoga olearia | 19 | 71 | 18 | 18 | 32 |
| Chryseobacterium arthrosphaerae | 0 | 0 | 0 | 0 | 0 |
| Pseudomonas cedrina | 131 | 316 | 311 | 12 | 640 |
| Azospirillum sp. TSH100 | 1356 | 1984 | 2041 | 53 | 2946 |
| Mucilaginibacter mallensis | 63 | 232 | 137 | 0 | 172 |
| Basfia succiniciproducens | 0 | 5 | 26 | 0 | 1 |
| Methylocystis bryophila | 1530 | 1596 | 1583 | 0 | 2332 |
| Neisseria sp. KEM232 | 296 | 217 | 239 | 7 | 488 |
| Pandoraea vervacti | 1040 | 1162 | 1059 | 13 | 1531 |
| Pandoraea faecigallinarum | 638 | 916 | 901 | 3 | 1272 |
| Arthrobacter alpinus | 1301 | 1792 | 2229 | 43 | 3007 |
| Halanaerobium hydrogeniformans | 3 | 22 | 25 | 0 | 120 |
| Kushneria phosphatilytica | 251 | 348 | 362 | 7 | 518 |
| Francisella noatunensis | 1 | 1 | 3 | 0 | 2 |
| Candidatus Koribacter versatilis | 2385 | 5501 | 4215 | 95 | 4774 |
| Paracoccus fistulariae | 0 | 0 | 0 | 0 | 0 |
| Pseudomonas sessilinigenes | 46 | 75 | 134 | 0 | 194 |
| Pseudomonas sp. CMR5c | 280 | 447 | 410 | 14 | 715 |
| Pseudomonas sp. R11-23-07 | 40 | 126 | 83 | 0 | 148 |
| Pseudomonas sp. R2-7-07 | 90 | 158 | 57 | 0 | 337 |
| Pseudomonas sp. R4-34-07 | 151 | 254 | 170 | 0 | 231 |
| Pseudomonas sp. R4-35-07 | 13 | 194 | 135 | 6 | 204 |
| Agromyces soli | 2657 | 2640 | 4371 | 63 | 5280 |
| Bacillus siamensis | 12 | 91 | 60 | 0 | 50 |
| Paracoccus stylophorae | 0 | 0 | 0 | 0 | 0 |
| Streptomyces sp. SN-593 | 4133 | 2494 | 5689 | 370 | 7706 |
| Neokomagataea tanensis | 112 | 197 | 75 | 0 | 120 |
| Flavisolibacter ginsenosidimutans | 0 | 0 | 0 | 0 | 0 |
| Pseudobacter ginsenosidimutans | 0 | 0 | 0 | 0 | 0 |
| Variovorax sp. RA8 | 4702 | 5350 | 4779 | 48 | 7113 |
| Fibrisoma limi | 12 | 7 | 3 | 0 | 0 |
| Halarcobacter bivalviorum | 0 | 4 | 3 | 0 | 7 |
| Sulfitobacter sp. BSw21498 | 270 | 260 | 228 | 1 | 275 |
| Vibrio plantisponsor | 12 | 35 | 46 | 2 | 46 |
| Halomonas titanicae | 421 | 511 | 782 | 28 | 976 |
| Azospirillum sp. TSH58 | 1893 | 1879 | 1826 | 9 | 2707 |
| Streptomyces incarnatus | 1829 | 1337 | 2418 | 84 | 3399 |
| Erwinia piriflorinigrans | 0 | 0 | 0 | 0 | 1 |
| Cytobacillus oceanisediminis | 0 | 0 | 0 | 0 | 0 |
| Mixta calida | 0 | 0 | 0 | 0 | 0 |
| Mixta gaviniae | 119 | 297 | 374 | 23 | 431 |
| Rhodanobacter denitrificans | 2273 | 2619 | 3005 | 174 | 3430 |
| Bacillus sp. 1NLA3E | 66 | 56 | 92 | 0 | 68 |
| Tenacibaculum dicentrarchi | 0 | 0 | 0 | 0 | 0 |
| Geminocystis herdmanii | 30 | 107 | 61 | 3 | 73 |
| Candidatus Profftella armatura | 0 | 0 | 0 | 0 | 0 |
| Cryobacterium arcticum | 800 | 872 | 1638 | 24 | 1761 |
| Caldinitratiruptor microaerophilus | 1492 | 2680 | 3424 | 56 | 4122 |
| Fusobacterium sp. oral taxon 203 | 0 | 0 | 5 | 0 | 1 |
| Serratia sp. FGI94 | 75 | 99 | 178 | 0 | 158 |
| Vibrio casei | 21 | 148 | 34 | 3 | 62 |
| Candidatus Babela massiliensis | 88 | 225 | 85 | 0 | 91 |
| Marisediminicola antarctica | 848 | 1709 | 1705 | 69 | 2215 |
| Bacteroides faecis | 52 | 103 | 48 | 0 | 114 |
| Rhodoplanes sp. Z2-YC6860 | 23214 | 14945 | 16288 | 432 | 40183 |
| Actinomyces weissii | 297 | 570 | 593 | 54 | 1106 |
| Aquibium microcysteis | 4256 | 4011 | 4230 | 130 | 5448 |
| Auraticoccus monumenti | 1474 | 2222 | 3660 | 151 | 4984 |
| Candidatus Rickettsiella viridis | 0 | 0 | 0 | 0 | 0 |
| Pseudomonas sp. ATCC 43928 | 165 | 326 | 276 | 27 | 415 |
| Rhodococcus sp. WMMA185 | 906 | 897 | 1649 | 56 | 1957 |
| Photobacterium swingsii | 39 | 52 | 30 | 0 | 36 |
| Agarivorans gilvus | 18 | 45 | 44 | 0 | 66 |
| Phaeobacter sp. LSS9 | 116 | 114 | 136 | 0 | 114 |
| Streptomyces sp. x3 | 0 | 2 | 1 | 0 | 2 |
| Streptomyces sp. W9 | 1 | 5 | 5 | 0 | 10 |
| Chitinolyticbacter meiyuanensis | 1002 | 1266 | 1178 | 24 | 2214 |
| Paenibacillus jilunlii | 45 | 265 | 168 | 1 | 422 |
| Azospirillum ramasamyi | 1901 | 1726 | 1757 | 38 | 2671 |
| Leucobacter denitrificans | 0 | 0 | 0 | 0 | 0 |
| Flavobacterium haoranii | 0 | 0 | 0 | 0 | 0 |
| Paenibacillus uliginis | 38 | 87 | 144 | 0 | 188 |
| Streptococcus lactarius | 0 | 0 | 0 | 0 | 0 |
| Agrococcus carbonis | 928 | 1532 | 1883 | 3 | 1428 |
| Pseudanabaena sp. ABRG5-3 | 15 | 86 | 56 | 0 | 67 |
| Martelella sp. AD-3 | 2039 | 1693 | 1423 | 34 | 2142 |
| Geobacillus sp. C56-T3 | 0 | 34 | 45 | 0 | 43 |
| Tuwongella immobilis | 1107 | 6929 | 3780 | 52 | 6630 |
| Tsuneonella dongtanensis | 742 | 869 | 769 | 0 | 899 |
| Caldisericum exile | 62 | 153 | 155 | 1 | 258 |
| Vibrio atlanticus | 49 | 43 | 22 | 1 | 34 |
| Brucella sp. BO2 | 86 | 71 | 48 | 0 | 84 |
| Vibrio owensii | 34 | 72 | 107 | 0 | 130 |
| Kushneria konosiri | 169 | 405 | 322 | 11 | 364 |
| Corynebacterium hindlerae | 0 | 0 | 0 | 0 | 0 |
| Blochmannia endosymbiont of Camponotus sp. | 6 | 17 | 1 | 0 | 8 |
| Amycolatopsis roodepoortensis | 10636 | 2433 | 7853 | 238 | 7078 |
| Mycobacterium marseillense | 1402 | 2379 | 2054 | 35 | 3357 |
| Streptomyces sp. Y27 | 1 | 0 | 5 | 0 | 7 |
| Novosphingobium sp. PP1Y | 0 | 0 | 0 | 0 | 0 |
| Methylomonas koyamae | 520 | 1015 | 773 | 10 | 823 |
| Pseudomonas arsenicoxydans | 0 | 0 | 0 | 0 | 0 |
| Myroides phaeus | 0 | 20 | 21 | 0 | 29 |
| Clostridium gelidum | 7 | 13 | 11 | 0 | 9 |
| Terrihabitans soli | 2208 | 1494 | 1016 | 0 | 1547 |
| Jeotgalibaca dankookensis | 5 | 38 | 5 | 0 | 19 |
| Aurantimicrobium minutum | 28 | 161 | 77 | 4 | 210 |
| Aliarcobacter trophiarum | 0 | 0 | 2 | 0 | 3 |
| Azospirillum sp. TSA2s | 1682 | 1800 | 2039 | 32 | 2640 |
| Micromonospora zamorensis | 2583 | 1647 | 5743 | 281 | 5917 |
| Frankia sp. QA3 | 2438 | 2274 | 4149 | 281 | 6622 |
| Acinetobacter sp. Tol 5 | 0 | 10 | 6 | 0 | 6 |
| Shewanella dokdonensis | 21 | 99 | 64 | 1 | 104 |
| Actinomyces sp. oral taxon 414 | 542 | 666 | 1021 | 2 | 785 |
| Aggregatibacter sp. oral taxon 513 | 58 | 26 | 12 | 0 | 50 |
| Atopobium sp. oral taxon 416 | 113 | 409 | 254 | 1 | 361 |
| Dietzia sp. oral taxon 368 | 771 | 1054 | 1467 | 184 | 1667 |
| Haemophilus sp. oral taxon 036 | 9 | 25 | 1 | 0 | 14 |
| Leptotrichia sp. oral taxon 218 | 0 | 0 | 0 | 0 | 0 |
| Leptotrichia sp. oral taxon 498 | 0 | 2 | 21 | 0 | 19 |
| Selenomonas sp. oral taxon 478 | 156 | 269 | 396 | 3 | 335 |
| Streptococcus sp. oral taxon 061 | 0 | 0 | 0 | 0 | 0 |
| Streptococcus sp. oral taxon 064 | 0 | 0 | 0 | 0 | 0 |
| Selenomonas sp. oral taxon 136 | 212 | 423 | 305 | 24 | 370 |
| Candidatus Atelocyanobacterium thalassa | 9 | 39 | 19 | 1 | 18 |
| Kroppenstedtia eburnea | 236 | 538 | 382 | 28 | 676 |
| Paenibacillus sp. sptzw28 | 126 | 430 | 408 | 1 | 1147 |
| Alteromonas naphthalenivorans | 13 | 12 | 33 | 0 | 34 |
| Amycolatopsis acidiphila | 17810 | 4619 | 17403 | 320 | 13886 |
| Oceanicoccus sagamiensis | 41 | 121 | 57 | 9 | 77 |
| Ensifer sojae | 1411 | 1622 | 1684 | 16 | 2416 |
| Caldicellulosiruptor obsidiansis | 28 | 48 | 15 | 0 | 23 |
| Hyphomicrobium sp. MC1 | 4576 | 1941 | 1839 | 2 | 1094 |
| Litorilituus sediminis | 50 | 18 | 16 | 4 | 20 |
| cyanobacterium endosymbiont of Epithemia turgida | 1 | 15 | 7 | 0 | 28 |
| Bradyrhizobium lablabi | 27824 | 24318 | 16956 | 157 | 16523 |
| Mycobacterium shigaense | 1672 | 3465 | 2649 | 97 | 4842 |
| Cycloclasticus sp. PY97N | 16 | 11 | 24 | 2 | 6 |
| Pseudarthrobacter equi | 495 | 746 | 1213 | 15 | 1982 |
| Methylobacterium sp. XJLW | 2212 | 1839 | 2451 | 38 | 4379 |
| Microbacterium oryzae | 921 | 1255 | 1868 | 35 | 2073 |
| Methylocystis sp. SB2 | 1477 | 1165 | 925 | 40 | 1318 |
| Sphingomonas sp. MM-1 | 5480 | 4312 | 4175 | 84 | 8286 |
| Oceanobacillus kimchii | 15 | 16 | 9 | 0 | 9 |
| Dolichospermum heterosporum | 84 | 456 | 216 | 0 | 546 |
| Cylindrospermopsis curvispora | 0 | 26 | 17 | 0 | 17 |
| Hymenobacter yonginensis | 616 | 718 | 803 | 38 | 969 |
| Azoarcus sp. KH32C | 2146 | 3066 | 2974 | 12 | 4391 |
| Pseudogulbenkiania sp. NH8B | 1072 | 1662 | 1613 | 66 | 2369 |
| Sulfuritalea hydrogenivorans | 1034 | 1573 | 1562 | 55 | 3086 |
| Coprococcus sp. ART55/1 | 0 | 0 | 0 | 0 | 0 |
| Polaribacter sp. SA4-10 | 10 | 13 | 10 | 0 | 12 |
| Winogradskyella sp. PG-2 | 0 | 0 | 0 | 0 | 0 |
| Winogradskyella sp. PC-19 | 20 | 27 | 18 | 4 | 37 |
| Tenacibaculum sp. SZ-18 | 0 | 0 | 0 | 0 | 0 |
| Gilvibacter sp. SZ-19 | 0 | 0 | 0 | 0 | 0 |
| Methylophaga nitratireducenticrescens | 0 | 0 | 0 | 0 | 0 |
| Methylophaga frappieri | 0 | 0 | 0 | 0 | 0 |
| Mycoplasma sp. Mirounga ES2805-ORL | 0 | 1 | 1 | 0 | 1 |
| Mycoplasma miroungigenitalium | 0 | 0 | 1 | 0 | 3 |
| Mycoplasma miroungirhinis | 1 | 2 | 4 | 0 | 5 |
| Mycoplasma phocoenae | 1 | 2 | 1 | 0 | 3 |
| Shewanella seohaensis | 45 | 23 | 15 | 0 | 44 |
| Clostridium sp. BNL1100 | 14 | 16 | 13 | 0 | 17 |
| Bacillus sp. WP8 | 29 | 14 | 0 | 0 | 7 |
| Caballeronia insecticola | 1143 | 1699 | 1683 | 1 | 2173 |
| Mycolicibacterium litorale | 5553 | 16325 | 11595 | 178 | 26919 |
| Acidovorax radicis | 0 | 0 | 0 | 0 | 0 |
| Weissella ceti | 15 | 46 | 16 | 0 | 26 |
| Bifidobacterium saguini | 45 | 52 | 69 | 1 | 74 |
| Siansivirga zeaxanthinifaciens | 16 | 17 | 15 | 0 | 14 |
| Vibrio jasicida | 0 | 20 | 45 | 0 | 36 |
| Serinicoccus chungangensis | 1334 | 1621 | 2344 | 49 | 2387 |
| Candidatus Puniceispirillum marinum | 147 | 128 | 84 | 0 | 260 |
| Shewanella indica | 27 | 68 | 48 | 0 | 81 |
| Sinorhizobium sp. CCBAU 05631 | 951 | 1214 | 752 | 5 | 1263 |
| Hydrogenophaga sp. PBC | 3403 | 3757 | 4231 | 34 | 5197 |
| Sphingobacterium lactis | 62 | 150 | 93 | 0 | 168 |
| Sphingomonas sp. SH | 39 | 74 | 173 | 0 | 184 |
| Halopseudomonas aestusnigri | 183 | 271 | 323 | 0 | 382 |
| Bradyrhizobium sp. CCBAU 051011 | 35978 | 37393 | 18039 | 33 | 17544 |
| Bradyrhizobium arachidis | 17352 | 15967 | 11520 | 176 | 13053 |
| Cytobacillus kochii | 6 | 12 | 14 | 1 | 17 |
| Cytobacillus gottheilii | 6 | 130 | 60 | 1 | 19 |
| Kibdelosporangium phytohabitans | 111620 | 14843 | 32377 | 304 | 43066 |
| Gemmatirosa kalamazoonensis | 12198 | 40601 | 29108 | 272 | 92079 |
| Psychrobacter sanguinis | 0 | 0 | 0 | 0 | 0 |
| Mucilaginibacter ginsenosidivorax | 0 | 0 | 0 | 0 | 0 |
| Streptomyces sp. SirexAA-E | 1735 | 1355 | 2887 | 236 | 4673 |
| Thermincola potens | 47 | 174 | 114 | 3 | 153 |
| Pseudoduganella umbonata | 1917 | 2380 | 2606 | 13 | 5833 |
| Photobacterium atrarenae | 0 | 0 | 0 | 0 | 0 |
| Paenibacillus sp. IHB B 3084 | 12 | 103 | 83 | 3 | 172 |
| Terriglobus saanensis | 648 | 2317 | 1897 | 5 | 2335 |
| Caballeronia zhejiangensis | 1531 | 2065 | 1762 | 8 | 3196 |
| Pseudoduganella flava | 1909 | 2933 | 2530 | 16 | 3882 |
| Arcobacter defluvii | 0 | 9 | 0 | 0 | 2 |
| Pseudoalteromonas arabiensis | 66 | 6 | 80 | 0 | 3 |
| Celeribacter baekdonensis | 0 | 0 | 0 | 0 | 0 |
| Mycolicibacter sinensis | 2801 | 3871 | 3713 | 270 | 6392 |
| Photobacterium sanguinicancri | 14 | 28 | 43 | 1 | 56 |
| Cupriavidus sp. USMAA2-4 | 1572 | 1671 | 1563 | 6 | 2755 |
| Halarcobacter anaerophilus | 2 | 8 | 4 | 0 | 3 |
| Chryseobacterium sp. StRB126 | 0 | 0 | 0 | 0 | 0 |
| Shinella sp. HZN7 | 3557 | 3673 | 3401 | 75 | 3669 |
| Pseudodesulfovibrio piezophilus | 40 | 120 | 151 | 0 | 168 |
| Enterobacter bugandensis | 232 | 255 | 247 | 5 | 461 |
| Pseudoalteromonas xiamenensis | 16 | 28 | 28 | 1 | 51 |
| Isorropodon fossajaponicum symbiont | 1 | 18 | 17 | 0 | 11 |
| Mageeibacillus indolicus | 8 | 41 | 196 | 0 | 69 |
| Enterobacter soli | 154 | 91 | 93 | 2 | 129 |
| Desulfosporosinus acidiphilus | 45 | 77 | 105 | 3 | 204 |
| Mycobacterium spongiae | 923 | 2402 | 2018 | 191 | 3511 |
| Bacillus sp. BS-02 | 0 | 0 | 0 | 0 | 0 |
| Methylovorus sp. MP688 | 209 | 434 | 239 | 0 | 370 |
| Psychrobacter sp. 4Dc | 14 | 0 | 0 | 0 | 0 |
| Alkalitalea saponilacus | 26 | 48 | 55 | 0 | 60 |
| Microbacterium sediminis | 1539 | 1962 | 2947 | 60 | 3516 |
| Flavobacterium sp. B183 | 104 | 363 | 175 | 0 | 202 |
| Pseudovibrio sp. FO-BEG1 | 331 | 339 | 308 | 14 | 367 |
| Caldanaerobacter subterraneus | 39 | 164 | 140 | 7 | 177 |
| Microbacterium sp. LKL04 | 405 | 584 | 1016 | 0 | 722 |
| Dietzia sp. DQ12-45-1b | 71 | 957 | 1521 | 0 | 997 |
| Pelolinea submarina | 195 | 507 | 436 | 2 | 469 |
| Arcobacter ellisii | 0 | 2 | 1 | 0 | 2 |
| Kangiella geojedonensis | 0 | 0 | 0 | 0 | 0 |
| Salinimonas lutimaris | 64 | 135 | 74 | 4 | 110 |
| Sandaracinus amylolyticus | 8064 | 15987 | 15358 | 177 | 16227 |
| Pseudomonas brassicacearum | 878 | 1439 | 1440 | 10 | 2440 |
| Nonlabens marinus | 5 | 23 | 20 | 0 | 11 |
| Halioglobus japonicus | 0 | 0 | 0 | 0 | 0 |
| Rickettsia endosymbiont of Oedothorax gibbosus | 4 | 28 | 12 | 0 | 16 |
| Wolbachia endosymbiont of Oedothorax gibbosus | 0 | 7 | 2 | 2 | 1 |
| Cardinium endosymbiont of Oedothorax gibbosus | 0 | 16 | 14 | 0 | 11 |
| Bradyrhizobium ottawaense | 12817 | 11168 | 8370 | 61 | 8191 |
| Oxalobacter vibrioformis | 96 | 173 | 176 | 0 | 254 |
| Desulfosarcina sp. BuS5 | 50 | 209 | 187 | 6 | 172 |
| Rhodococcus sp. p52 | 443 | 196 | 472 | 25 | 655 |
| Flavobacterium sp. Sr18 | 0 | 0 | 0 | 0 | 0 |
| Yersinia entomophaga | 0 | 0 | 0 | 0 | 0 |
| Microbacterium amylolyticum | 348 | 679 | 827 | 22 | 1066 |
| Desulfurispirillum indicum | 0 | 0 | 0 | 0 | 0 |
| Marinomonas posidonica | 32 | 24 | 25 | 6 | 35 |
| Micrococcus sp. MG-2010-D12 | 1 | 0 | 1 | 0 | 0 |
| Companilactobacillus futsaii | 9 | 3 | 3 | 0 | 5 |
| Calothrix brevissima | 104 | 536 | 315 | 8 | 502 |
| Granulicella mallensis | 1134 | 3149 | 2649 | 19 | 3280 |
| Granulicella tundricola | 1008 | 2420 | 2074 | 29 | 2212 |
| Allosphingosinicella indica | 1424 | 2260 | 1453 | 88 | 6641 |
| Rhodoluna sp. KAS3 | 38 | 40 | 60 | 0 | 217 |
| Tardiphaga robiniae | 2633 | 2229 | 1806 | 20 | 2405 |
| Amycolatopsis thermalba | 12754 | 3850 | 13279 | 176 | 9670 |
| Arcobacter sp. L | 0 | 5 | 24 | 0 | 5 |
| Massilia oculi | 0 | 742 | 2474 | 0 | 773 |
| Rhizobacter gummiphilus | 2349 | 4522 | 3326 | 0 | 4317 |
| Actinoplanes sp. OR16 | 3853 | 3530 | 8761 | 243 | 8265 |
| Desulfosarcina widdelii | 509 | 1248 | 1050 | 4 | 1393 |
| Paraburkholderia sprentiae | 1040 | 1387 | 1282 | 6 | 1644 |
| Aeromonas rivipollensis | 0 | 0 | 0 | 0 | 0 |
| Leuconostoc sp. C2 | 2 | 0 | 8 | 0 | 0 |
| Mariniblastus fucicola | 504 | 1689 | 1170 | 15 | 1783 |
| Roseimaritima ulvae | 1683 | 4400 | 2745 | 38 | 4720 |
| Ruegeria conchae | 194 | 249 | 212 | 1 | 328 |
| Thermotomaculum hydrothermale | 53 | 144 | 163 | 0 | 121 |
| Lacinutrix sp. 5H-3-7-4 | 3 | 17 | 29 | 0 | 42 |
| Dokdonia sp. 4H-3-7-5 | 0 | 15 | 8 | 0 | 15 |
| Sphaerospermopsis torques-reginae | 106 | 816 | 209 | 0 | 275 |
| Mycoplasma parvum | 0 | 2 | 2 | 0 | 0 |
| Staphylococcus argenteus | 4 | 3 | 26 | 0 | 26 |
| Polaribacter sejongensis | 0 | 19 | 15 | 0 | 13 |
| Staphylococcus agnetis | 2 | 18 | 18 | 1 | 23 |
| Vibrio maritimus | 12 | 57 | 67 | 0 | 167 |
| Polymorphum gilvum | 3635 | 3250 | 2933 | 54 | 4289 |
| Bradyrhizobium daqingense | 2763 | 1829 | 1121 | 0 | 214 |
| Metallibacterium scheffleri | 75 | 239 | 152 | 5 | 265 |
| Candidatus Kinetoplastibacterium desouzaii | 12 | 4 | 2 | 0 | 11 |
| Candidatus Kinetoplastibacterium galatii | 0 | 2 | 15 | 0 | 5 |
| Candidatus Kinetoplastibacterium oncopeltii | 0 | 6 | 4 | 0 | 4 |
| Polaribacter reichenbachii | 3 | 22 | 16 | 0 | 20 |
| Halomonas sp. TD01 | 0 | 0 | 0 | 0 | 0 |
| Candidatus Pelagibacter sp. IMCC9063 | 7 | 12 | 12 | 4 | 35 |
| Occallatibacter riparius | 1864 | 4841 | 3898 | 23 | 4471 |
| Micromonospora maris | 2459 | 1977 | 5370 | 185 | 6341 |
| Gordonia iterans | 1439 | 1553 | 2510 | 45 | 2336 |
| Fimbriimonas ginsengisoli | 864 | 2365 | 2097 | 14 | 2787 |
| Listeria weihenstephanensis | 15 | 55 | 25 | 0 | 126 |
| Defluviitoga tunisiensis | 15 | 33 | 57 | 0 | 38 |
| Pusillimonas sp. T7-7 | 0 | 0 | 0 | 0 | 0 |
| Companilactobacillus ginsenosidimutans | 0 | 10 | 8 | 0 | 8 |
| Jeongeupia sp. HS-3 | 343 | 833 | 685 | 0 | 1064 |
| Novosphingobium sp. THN1 | 0 | 0 | 0 | 0 | 0 |
| Endozoicomonas montiporae | 37 | 101 | 97 | 5 | 114 |
| Psychrobacter sp. DAB_AL43B | 99 | 44 | 19 | 7 | 35 |
| Shewanella aestuarii | 3 | 33 | 30 | 3 | 47 |
| Pseudomonas sp. StFLB209 | 322 | 492 | 570 | 38 | 1221 |
| Sphingomonas sp. KC8 | 970 | 1081 | 482 | 0 | 869 |
| Neptunomonas concharum | 0 | 0 | 0 | 0 | 0 |
| Campylobacter volucris | 0 | 10 | 2 | 0 | 3 |
| Malaciobacter molluscorum | 2 | 1 | 4 | 0 | 2 |
| Campylobacter sp. RM10537 | 8 | 1 | 4 | 0 | 2 |
| Campylobacter sp. RM6914 | 0 | 12 | 16 | 0 | 39 |
| Otariodibacter oris | 0 | 10 | 6 | 1 | 12 |
| Corynebacterium nuruki | 0 | 0 | 0 | 0 | 0 |
| Marinobacter adhaerens | 372 | 314 | 408 | 0 | 368 |
| Thioalkalivibrio sulfidiphilus | 1990 | 2945 | 2987 | 36 | 2888 |
| Rhodococcus sp. WB1 | 369 | 618 | 762 | 14 | 850 |
| Variovorax sp. HW608 | 2788 | 3966 | 3429 | 0 | 4373 |
| Planococcus plakortidis | 165 | 347 | 315 | 0 | 282 |
| Streptomyces xinghaiensis | 2482 | 2002 | 3682 | 238 | 6891 |
| Cupriavidus neocaledonicus | 767 | 862 | 1019 | 35 | 1608 |
| Candidatus Arthromitus sp. SFB-rat-Yit | 0 | 0 | 0 | 0 | 0 |
| Clostridium sp. SY8519 | 39 | 130 | 146 | 12 | 183 |
| Acidiphilium sp. PM | 29 | 68 | 74 | 0 | 110 |
| Sphingomonas lutea | 1293 | 1645 | 1165 | 14 | 2609 |
| Rhodococcus sp. YL-1 | 87 | 269 | 279 | 13 | 391 |
| Nocardia sp. CS682 | 3066 | 2368 | 5638 | 179 | 5601 |
| Candidatus Moranella endobia | 29 | 28 | 30 | 1 | 43 |
| Corynebacterium epidermidicanis | 162 | 191 | 270 | 10 | 386 |
| Thiomonas sp. X19 | 920 | 2054 | 1812 | 24 | 2251 |
| Synechococcus sp. A15-127 | 81 | 374 | 204 | 0 | 289 |
| Synechococcus sp. A15-44 | 156 | 150 | 196 | 2 | 209 |
| Synechococcus sp. A15-60 | 25 | 302 | 120 | 0 | 54 |
| Synechococcus sp. A15-62 | 78 | 77 | 139 | 8 | 158 |
| Synechococcus sp. SYN20 | 16 | 74 | 43 | 0 | 26 |
| Arcobacter venerupis | 0 | 0 | 0 | 0 | 0 |
| Arcobacter cloacae | 0 | 8 | 1 | 0 | 0 |
| Cobetia pacifica | 126 | 271 | 240 | 0 | 383 |
| Cobetia amphilecti | 0 | 0 | 0 | 0 | 0 |
| Methylotenera versatilis | 0 | 0 | 0 | 0 | 0 |
| Morococcus cerebrosus | 56 | 172 | 152 | 0 | 154 |
| Azospirillum baldaniorum | 1423 | 2002 | 1599 | 56 | 2528 |
| Mycolicibacillus koreensis | 1259 | 2297 | 2451 | 67 | 3782 |
| Mycolicibacillus parakoreensis | 1595 | 2456 | 2506 | 96 | 4020 |
| Candidatus Gullanella endobia | 9 | 8 | 2 | 0 | 4 |
| Bacillus sp. NSP9.1 | 120 | 134 | 106 | 0 | 237 |
| Bacillus sp. SB49 | 72 | 86 | 69 | 0 | 109 |
| Caballeronia grimmiae | 2172 | 2684 | 2552 | 73 | 3224 |
| Corynebacterium uterequi | 0 | 0 | 0 | 0 | 0 |
| Microbacterium lemovicicum | 1251 | 1618 | 3039 | 123 | 2945 |
| Vibrio alfacsensis | 105 | 312 | 447 | 8 | 341 |
| Companilactobacillus heilongjiangensis | 2 | 6 | 4 | 1 | 14 |
| Phytohabitans flavus | 6773 | 4512 | 14203 | 310 | 14572 |
| Pantoea rwandensis | 0 | 0 | 0 | 0 | 0 |
| Thiolapillus brandeum | 187 | 509 | 368 | 53 | 516 |
| Acetobacter persici | 115 | 226 | 185 | 0 | 284 |
| Candidatus Steffania adelgidicola | 21 | 6 | 14 | 0 | 19 |
| Rhizobium laguerreae | 523 | 534 | 268 | 3 | 262 |
| Paracoccus zhejiangensis | 0 | 0 | 0 | 0 | 0 |
| Streptomyces hundungensis | 2139 | 1784 | 3364 | 137 | 5736 |
| Serinicoccus profundi | 915 | 1098 | 1889 | 70 | 2135 |
| Malaciobacter pacificus | 0 | 8 | 6 | 0 | 5 |
| Candidatus Schneideria nysicola | 0 | 7 | 8 | 0 | 5 |
| Halomonas socia | 437 | 604 | 614 | 0 | 932 |
| Lonsdalea britannica | 123 | 291 | 295 | 1 | 485 |
| Comamonas serinivorans | 1217 | 1692 | 1743 | 66 | 2374 |
| Persicobacter sp. JZB09 | 0 | 0 | 0 | 0 | 0 |
| Allofrancisella frigidaquae | 0 | 5 | 9 | 1 | 6 |
| Allofrancisella inopinata | 5 | 2 | 3 | 0 | 4 |
| Dickeya solani | 106 | 249 | 460 | 0 | 1021 |
| Nakamurella panacisegetis | 1896 | 1661 | 3407 | 123 | 4332 |
| Alloalcanivorax xenomutans | 373 | 528 | 587 | 90 | 673 |
| Idiomarina piscisalsi | 0 | 0 | 0 | 0 | 0 |
| Pseudonocardia sp. EC080619-01 | 564 | 2571 | 3550 | 492 | 7196 |
| Pseudonocardia sp. EC080625-04 | 2326 | 878 | 3164 | 11 | 1809 |
| Rickettsia philipii | 2 | 0 | 3 | 0 | 0 |
| Variovorax sp. WS11 | 962 | 1168 | 1048 | 0 | 1551 |
| Chromobacterium vaccinii | 954 | 1273 | 1291 | 54 | 1954 |
| Brenneria goodwinii | 261 | 245 | 272 | 5 | 554 |
| Verminephrobacter aporrectodeae | 0 | 1 | 0 | 0 | 0 |
| Streptococcus troglodytae | 0 | 0 | 0 | 0 | 0 |
| Alcanivorax sp. NBRC 101098 | 79 | 31 | 73 | 0 | 141 |
| Flavobacterium collinsii | 0 | 0 | 0 | 0 | 0 |
| Pseudomonas ogarae | 49 | 285 | 139 | 0 | 322 |
| Leptolyngbya sp. BL0902 | 0 | 0 | 0 | 0 | 0 |
| Romboutsia ilealis | 16 | 22 | 18 | 0 | 7 |
| Vibrio sp. EJY3 | 20 | 274 | 4 | 0 | 12 |
| Vibrio quintilis | 52 | 138 | 73 | 13 | 72 |
| Halomonas sp. GFAJ-1 | 0 | 0 | 0 | 0 | 0 |
| Cruoricaptor ignavus | 55 | 180 | 115 | 0 | 109 |
| Mycolicibacter minnesotensis | 853 | 1731 | 1736 | 66 | 2864 |
| Rhizobium grahamii | 191 | 264 | 336 | 1 | 441 |
| Corynebacterium doosanense | 769 | 728 | 1059 | 12 | 676 |
| Telmatocola sphagniphila | 1089 | 6341 | 3260 | 129 | 5807 |
| Magnetococcus marinus | 161 | 357 | 260 | 9 | 363 |
| Chryseobacterium carnipullorum | 17 | 94 | 36 | 0 | 51 |
| Pseudorhizobium banfieldiae | 892 | 1163 | 1019 | 10 | 1759 |
| Polycladomyces abyssicola | 120 | 374 | 336 | 3 | 350 |
| Paenibacillus beijingensis | 245 | 649 | 395 | 0 | 787 |
| Bacillus sp. JS | 0 | 25 | 0 | 0 | 40 |
| Sphaerochaeta associata | 142 | 118 | 189 | 4 | 190 |
| Dehalobacter sp. CF | 0 | 0 | 76 | 0 | 50 |
| Sphaerochaeta globosa | 30 | 89 | 67 | 0 | 93 |
| Sphaerochaeta pleomorpha | 13 | 91 | 33 | 3 | 44 |
| Rhizobium sp. CCGE 510 | 158 | 160 | 190 | 0 | 238 |
| Luteimicrobium xylanilyticum | 2351 | 2443 | 3388 | 74 | 3663 |
| Melioribacter roseus | 96 | 260 | 197 | 1 | 181 |
| Thauera humireducens | 1237 | 1697 | 2093 | 0 | 3373 |
| Gordonia phthalatica | 1488 | 1847 | 2949 | 146 | 3661 |
| Scytonema sp. HK-05 | 125 | 702 | 298 | 30 | 516 |
| Leptospira mayottensis | 5 | 40 | 14 | 0 | 34 |
| Rhizobium bangladeshense | 1097 | 1105 | 975 | 0 | 885 |
| Rhizobium binae | 762 | 893 | 790 | 0 | 751 |
| Rhizobium lentis | 859 | 751 | 714 | 26 | 897 |
| Mycobacterium paraintracellulare | 9606 | 27674 | 21469 | 489 | 50039 |
| Lentilactobacillus curieae | 0 | 0 | 0 | 0 | 0 |
| Massilia putida | 2088 | 3344 | 3365 | 58 | 5328 |
| Kangiella sediminilitoris | 9 | 65 | 63 | 19 | 54 |
| Lysinibacillus varians | 32 | 0 | 0 | 0 | 0 |
| Dehalobacter sp. DCA | 0 | 54 | 40 | 0 | 0 |
| Acinetobacter oleivorans | 1 | 4 | 1 | 1 | 5 |
| Pseudomonas furukawaii | 857 | 1093 | 1103 | 29 | 1312 |
| Brucella sp. 09RB8471 | 215 | 173 | 192 | 0 | 171 |
| Brucella sp. 10RB9215 | 20 | 151 | 151 | 0 | 136 |
| Lactococcus taiwanensis | 0 | 10 | 18 | 0 | 5 |
| Glutamicibacter sp. ZJUTW | 0 | 0 | 0 | 0 | 0 |
| Clostridium sp. C1 | 0 | 47 | 44 | 15 | 217 |
| Moorena producens | 336 | 1058 | 556 | 8 | 868 |
| Thermotoga sp. Cell2 | 0 | 56 | 0 | 0 | 42 |
| Thermotoga sp. 2812B | 62 | 43 | 13 | 0 | 42 |
| Kosakonia sacchari | 0 | 0 | 0 | 0 | 0 |
| Pontimonas salivibrio | 0 | 0 | 0 | 0 | 0 |
| Ruminococcus bicirculans | 42 | 84 | 80 | 0 | 38 |
| Candidatus Nasuia deltocephalinicola | 17 | 26 | 20 | 0 | 79 |
| Flavobacterium sp. HJ-32-4 | 0 | 0 | 0 | 0 | 715 |
| Ruminococcus champanellensis | 55 | 115 | 90 | 2 | 172 |
| Weissella diestrammenae | 20 | 12 | 13 | 0 | 12 |
| Pseudomonas donghuensis | 0 | 0 | 0 | 0 | 0 |
| Cronobacter condimenti | 273 | 363 | 222 | 4 | 647 |
| Imtechella halotolerans | 1 | 25 | 21 | 0 | 16 |
| Candidatus Thiodictyon syntrophicum | 1620 | 3847 | 3228 | 70 | 4629 |
| Draconibacterium orientale | 40 | 87 | 106 | 1 | 95 |
| Mycobacterium sp. MOTT36Y | 379 | 596 | 763 | 0 | 1234 |
| Streptomyces pratensis | 1618 | 1355 | 3002 | 213 | 5202 |
| Calothrix sp. PCC 6303 | 215 | 162 | 106 | 1 | 102 |
| Galbibacter sp. BG1 | 8 | 28 | 33 | 0 | 35 |
| Streptomyces sp. W75 | 0 | 2 | 2 | 0 | 10 |
| Lonsdalea populi | 0 | 0 | 0 | 0 | 0 |
| Geitlerinema sp. PCC 7407 | 459 | 832 | 827 | 5 | 955 |
| Gloeocapsa sp. PCC 7428 | 0 | 0 | 0 | 0 | 0 |
| Chamaesiphon minutus | 131 | 435 | 320 | 3 | 1074 |
| Methylomonas paludis | 63 | 150 | 156 | 2 | 155 |
| Synechococcus sp. PCC 7502 | 16 | 83 | 30 | 1 | 50 |
| Pseudomonas sp. R1-43-08 | 133 | 163 | 88 | 0 | 880 |
| Pseudomonas sp. R2-37-08W | 0 | 0 | 0 | 0 | 0 |
| Pseudomonas sp. R2-60-08W | 50 | 139 | 107 | 0 | 209 |
| Pseudomonas sp. R3-18-08 | 38 | 145 | 198 | 0 | 319 |
| Pseudomonas sp. R3-52-08 | 12 | 95 | 118 | 0 | 224 |
| Pseudomonas sp. R4-39-08 | 100 | 93 | 36 | 11 | 297 |
| Shewanella litorisediminis | 0 | 0 | 0 | 0 | 0 |
| Streptomyces harbinensis | 1954 | 1655 | 3166 | 236 | 6527 |
| [Pseudomonas] zhaodongensis | 0 | 0 | 0 | 0 | 0 |
| Sulfurimonas gotlandica | 11 | 15 | 11 | 0 | 32 |
| Luteimonas granuli | 1661 | 1653 | 1800 | 8 | 1642 |
| Novosphingobium ginsenosidimutans | 0 | 0 | 0 | 0 | 0 |
| Niabella ginsenosidivorans | 339 | 519 | 323 | 1 | 371 |
| Sphingomonas daechungensis | 889 | 1266 | 921 | 10 | 2043 |
| Agrobacterium fabrum | 997 | 879 | 1020 | 27 | 1414 |
| Mucilaginibacter jinjuensis | 0 | 0 | 0 | 0 | 0 |
| Candidatus Vallotia cooleyia | 51 | 88 | 53 | 1 | 70 |
| Candidatus Vallotia tarda | 4 | 27 | 24 | 0 | 46 |
| Candidatus Profftia tarda | 1 | 5 | 3 | 0 | 8 |
| Komagataeibacter medellinensis | 0 | 0 | 0 | 0 | 0 |
| Halomonas huangheensis | 251 | 336 | 364 | 6 | 461 |
| Paenibacillus swuensis | 103 | 169 | 186 | 0 | 219 |
| Spirosoma montaniterrae | 0 | 0 | 0 | 0 | 0 |
| Bacillus xiamenensis | 0 | 0 | 7 | 0 | 0 |
| Bacillus zhangzhouensis | 23 | 53 | 21 | 0 | 15 |
| Maribacter cobaltidurans | 0 | 0 | 0 | 0 | 0 |
| Yersinia sp. KBS0713 | 16 | 36 | 31 | 0 | 15 |
| Flavobacterium sp. KBS0721 | 71 | 1990 | 839 | 13 | 590 |
| Oerskovia sp. KBS0722 | 1628 | 2126 | 3435 | 117 | 4194 |
| Burkholderia sp. KBS0801 | 0 | 0 | 0 | 0 | 0 |
| Mycoplasma feriruminatoris | 0 | 0 | 0 | 0 | 0 |
| Sulfitobacter sp. DFL14 | 2 | 0 | 1 | 0 | 0 |
| Deinococcus puniceus | 0 | 0 | 0 | 0 | 0 |
| Agrobacterium fabacearum | 2329 | 1346 | 1249 | 8 | 1578 |
| Agrobacterium deltaense | 0 | 102 | 24 | 0 | 72 |
| Agrobacterium salinitolerans | 703 | 515 | 490 | 55 | 718 |
| Mesotoga prima | 50 | 59 | 89 | 0 | 124 |
| Rhizobium anhuiense | 713 | 681 | 580 | 21 | 581 |
| Blattabacterium sp. (Blaberus giganteus) | 1 | 4 | 0 | 0 | 1 |
| Streptomyces sp. X335 | 0 | 0 | 16 | 0 | 67 |
| Gloeomargarita lithophora | 81 | 160 | 169 | 1 | 190 |
| Streptomyces sp. AgN23 | 1832 | 1516 | 2409 | 118 | 4122 |
| Ferriphaselus amnicola | 384 | 606 | 487 | 18 | 1197 |
| Pseudoalteromonas shioyasakiensis | 40 | 75 | 34 | 9 | 40 |
| Flammeovirga sp. MY04 | 43 | 31 | 40 | 0 | 26 |
| Aquimarina sp. Aq107 | 13 | 22 | 7 | 0 | 9 |
| Vogesella sp. LIG4 | 469 | 972 | 890 | 12 | 1400 |
| Paucilactobacillus hokkaidonensis | 1 | 18 | 18 | 0 | 10 |
| Neobacillus mesonae | 0 | 0 | 0 | 0 | 0 |
| Vibrio toranzoniae | 1 | 13 | 12 | 0 | 27 |
| Clostridium sp. MT351 | 0 | 0 | 0 | 0 | 2 |
| Snodgrassella alvi | 16 | 38 | 28 | 0 | 25 |
| Gilliamella apicola | 0 | 0 | 0 | 0 | 0 |
| Bradyrhizobium sp. 6(2017) | 5352 | 4955 | 3112 | 0 | 2596 |
| Cloacibacillus porcorum | 0 | 0 | 0 | 0 | 0 |
| secondary endosymbiont of Ctenarytaina eucalypti | 1 | 13 | 12 | 1 | 29 |
| Pectobacterium aroidearum | 135 | 86 | 167 | 0 | 185 |
| Geotalea daltonii | 278 | 607 | 467 | 28 | 863 |
| Pseudomonas sp. Lz4W | 33 | 65 | 82 | 0 | 180 |
| Pseudomonas sp. UW4 | 172 | 258 | 281 | 0 | 518 |
| Burkholderia pseudomultivorans | 0 | 0 | 0 | 0 | 0 |
| Vibrio neocaledonicus | 0 | 10 | 0 | 0 | 0 |
| Celeribacter indicus | 1686 | 2117 | 2672 | 39 | 3142 |
| Halomicronema hongdechloris | 249 | 556 | 464 | 7 | 677 |
| Gemmata massiliana | 5351 | 58977 | 22525 | 94 | 27125 |
| Deinococcus metallilatus | 456 | 1165 | 1204 | 0 | 1193 |
| Spirosoma aerolatum | 117 | 233 | 250 | 1 | 374 |
| Stutzerimonas kunmingensis | 190 | 297 | 223 | 5 | 370 |
| Tumebacillus algifaecis | 403 | 1334 | 636 | 9 | 730 |
| Planococcus halocryophilus | 0 | 32 | 22 | 0 | 23 |
| Virgibacillus natechei | 3 | 32 | 5 | 1 | 21 |
| Clostridium bornimense | 28 | 19 | 16 | 0 | 25 |
| Parabacteroides faecis | 64 | 187 | 80 | 0 | 114 |
| Octadecabacter antarcticus | 149 | 345 | 179 | 2 | 393 |
| Streptomyces sp. GBA 94-10 4N24 | 457 | 238 | 475 | 191 | 815 |
| Brucella inopinata | 52 | 45 | 58 | 0 | 53 |
| Bombilactobacillus mellifer | 0 | 2 | 0 | 0 | 0 |
| Lactobacillus kullabergensis | 8 | 4 | 3 | 2 | 7 |
| Lactobacillus helsingborgensis | 0 | 4 | 3 | 0 | 8 |
| Apilactobacillus apinorum | 20 | 7 | 0 | 0 | 5 |
| Kordia antarctica | 0 | 0 | 0 | 0 | 0 |
| Actinomadura sp. WMMB 499 | 6423 | 4483 | 9890 | 1219 | 22024 |
| Caldicellulosiruptor changbaiensis | 0 | 41 | 44 | 0 | 17 |
| Corynebacterium humireducens | 462 | 551 | 798 | 36 | 615 |
| Rhizobium sp. Pop5 | 749 | 902 | 901 | 11 | 944 |
| Bradyrhizobium sp. CCGE-LA001 | 2588 | 1496 | 749 | 0 | 0 |
| Dickeya sp. CSL RW240 | 0 | 0 | 0 | 0 | 0 |
| Exiguobacterium sp. ZWU0009 | 12 | 41 | 27 | 0 | 16 |
| Sediminicoccus rosea | 1636 | 1673 | 1554 | 8 | 2225 |
| Streptomyces sp. PVA_94-07 | 167 | 352 | 871 | 0 | 885 |
| Paludibacterium paludis | 722 | 897 | 974 | 7 | 1120 |
| Dickeya sp. DW 0440 | 0 | 0 | 56 | 0 | 0 |
| Streptomyces rapamycinicus | 3443 | 2136 | 4427 | 330 | 5872 |
| Azospirillum humicireducens | 1393 | 1609 | 1500 | 26 | 2486 |
| Sphingomicrobium flavum | 590 | 578 | 453 | 0 | 839 |
| Anaerostipes rhamnosivorans | 22 | 98 | 51 | 0 | 54 |
| Salipiger profundus | 1975 | 1965 | 2173 | 20 | 2926 |
| Salimicrobium jeotgali | 0 | 0 | 0 | 0 | 0 |
| Corynebacterium frankenforstense | 908 | 1003 | 1621 | 57 | 2208 |
| Corynebacterium lactis | 0 | 0 | 0 | 0 | 0 |
| Arsenophonus endosymbiont of Aphis craccivora | 0 | 0 | 0 | 0 | 0 |
| Megasphaera massiliensis | 94 | 211 | 265 | 0 | 198 |
| Acidithiobacillus ferridurans | 199 | 479 | 344 | 0 | 512 |
| Comamonas sp. 7D-2 | 1157 | 3232 | 2822 | 0 | 3189 |
| Chitinophaga solisilvae | 0 | 0 | 3 | 0 | 3 |
| Geobacillus sp. GHH01 | 0 | 0 | 0 | 0 | 0 |
| Endozoicomonas euniceicola | 82 | 241 | 136 | 46 | 142 |
| Gayadomonas joobiniege | 0 | 0 | 0 | 0 | 0 |
| Streptococcus rubneri | 3 | 16 | 10 | 0 | 16 |
| Mucilaginibacter xinganensis | 84 | 144 | 100 | 0 | 149 |
| Pseudorhodoplanes sinuspersici | 15795 | 4167 | 4319 | 45 | 7587 |
| Candidatus Pantoea carbekii | 2 | 14 | 9 | 0 | 16 |
| Mesotoga infera | 82 | 92 | 57 | 0 | 154 |
| Janthinobacterium sp. B9-8 | 111 | 298 | 152 | 6 | 214 |
| Sodalis praecaptivus | 239 | 318 | 316 | 5 | 481 |
| Dokdonia sp. PRO95 | 0 | 29 | 6 | 0 | 15 |
| Dickeya oryzae | 19 | 7 | 19 | 0 | 11 |
| Chryseobacterium lactis | 49 | 73 | 43 | 0 | 66 |
| Chryseobacterium nakagawai | 44 | 17 | 26 | 17 | 20 |
| Campylobacter iguaniorum | 5 | 12 | 5 | 1 | 12 |
| Pseudomonas guangdongensis | 416 | 840 | 731 | 0 | 691 |
| endosymbiont of unidentified scaly snail isolate Monju | 705 | 936 | 972 | 7 | 931 |
| Mycolicibacterium celeriflavum | 3730 | 14456 | 8876 | 65 | 24357 |
| Pseudohongiella spirulinae | 74 | 206 | 203 | 22 | 293 |
| Bacterioplanes sanyensis | 0 | 0 | 0 | 0 | 0 |
| Gramella sp. MAR_2010_147 | 16 | 130 | 18 | 0 | 31 |
| Salipiger abyssi | 1977 | 1865 | 2213 | 10 | 2603 |
| Pseudomonas sp. FGI182 | 57 | 109 | 212 | 0 | 258 |
| Raoultella electrica | 0 | 0 | 0 | 0 | 0 |
| Nostoc sp. 'Peltigera membranacea cyanobiont' N6 | 133 | 127 | 122 | 0 | 210 |
| Streptomyces sp. 769 | 1360 | 1168 | 2114 | 266 | 5484 |
| Edwardsiella piscicida | 215 | 309 | 836 | 0 | 907 |
| Candidatus Endolissoclinum faulkneri | 48 | 121 | 57 | 13 | 312 |
| Streptomyces sp. ID38640 | 780 | 687 | 1029 | 9 | 1314 |
| Streptomyces sp. PAMC 26508 | 548 | 486 | 788 | 89 | 1253 |
| Psychrosphaera aestuarii | 9 | 75 | 42 | 56 | 58 |
| Frischella perrara | 3 | 22 | 15 | 0 | 10 |
| Melaminivora jejuensis | 354 | 881 | 837 | 0 | 556 |
| Brevirhabdus pacifica | 934 | 1549 | 1475 | 6 | 2130 |
| Liberibacter crescens | 180 | 77 | 20 | 0 | 25 |
| Bradyrhizobium icense | 27774 | 25851 | 13861 | 220 | 14937 |
| Staphylococcus petrasii | 0 | 14 | 0 | 0 | 0 |
| Geobacter sp. DSM 9736 | 379 | 921 | 666 | 3 | 963 |
| Arcobacter suis | 0 | 7 | 4 | 0 | 4 |
| Keratinibaculum paraultunense | 315 | 41 | 100 | 2 | 97 |
| Synechococcus sp. KORDI-100 | 201 | 275 | 252 | 2 | 312 |
| Lactococcus formosensis | 0 | 0 | 0 | 0 | 0 |
| Acidiferrobacter sp. SPIII_3 | 622 | 1091 | 867 | 8 | 1046 |
| Flaviflexus salsibiostraticola | 637 | 820 | 1133 | 52 | 1478 |
| Uruburuella testudinis | 168 | 216 | 204 | 2 | 281 |
| Pseudomonas sp. URMO17WK12:I11 | 662 | 794 | 829 | 0 | 1686 |
| Tessaracoccus defluvii | 1262 | 881 | 2211 | 23 | 2714 |
| Mycolicibacterium sediminis | 3214 | 9088 | 5768 | 63 | 11958 |
| Mycolicibacterium arabiense | 2872 | 8908 | 5668 | 60 | 12618 |
| Crassaminicella profunda | 22 | 15 | 23 | 3 | 12 |
| Brachyspira hampsonii | 0 | 0 | 0 | 0 | 0 |
| Anaerococcus obesiensis | 0 | 0 | 0 | 0 | 0 |
| Alistipes senegalensis | 375 | 688 | 532 | 2 | 641 |
| Nitrosospira lacus | 350 | 11688 | 814 | 17 | 1115 |
| Diaphorobacter aerolatus | 1785 | 3238 | 3235 | 23 | 4703 |
| Ornithinimicrobium flavum | 415 | 575 | 1445 | 27 | 1051 |
| Magnetospira sp. QH-2 | 830 | 683 | 745 | 7 | 1099 |
| Gordonia ajococcus | 0 | 65 | 68 | 0 | 561 |
| Swingsia samuiensis | 29 | 19 | 15 | 0 | 43 |
| Pseudomonas sp. ATCC 13867 | 504 | 754 | 924 | 22 | 1162 |
| Methylogaea oryzae | 851 | 1688 | 1340 | 17 | 1629 |
| Photobacterium gaetbulicola | 0 | 0 | 0 | 0 | 0 |
| Thermodesulfobacterium geofontis | 11 | 55 | 54 | 0 | 50 |
| Parageobacillus genomosp. 1 | 0 | 0 | 0 | 0 | 0 |
| Anabaena sp. CCAP 1446/1C | 0 | 270 | 78 | 0 | 52 |
| Paucilactobacillus nenjiangensis | 0 | 17 | 7 | 1 | 13 |
| Intestinimonas butyriciproducens | 376 | 584 | 734 | 6 | 912 |
| Myxococcus hansupus | 1629 | 3529 | 3004 | 42 | 3899 |
| Rhizobium sp. IE4771 | 356 | 510 | 298 | 3 | 326 |
| Arcanobacterium phocisimile | 7 | 97 | 257 | 0 | 164 |
| Rhodococcus sp. P1Y | 1369 | 1209 | 1956 | 21 | 2364 |
| Candidatus Pseudomonas adelgestsugas | 4 | 10 | 14 | 7 | 23 |
| Candidatus Annandia adelgestsuga | 0 | 0 | 0 | 0 | 0 |
| Planococcus versutus | 2 | 19 | 13 | 0 | 35 |
| Bombilactobacillus bombi | 7 | 3 | 7 | 0 | 8 |
| Mycobacterium orygis | 27 | 45 | 80 | 0 | 50 |
| Nostoc flagelliforme | 276 | 452 | 500 | 0 | 630 |
| Flavobacterium commune | 0 | 0 | 0 | 0 | 0 |
| Paenibacillus sp. HWE-109 | 143 | 315 | 230 | 1 | 364 |
| Isoalcanivorax pacificus | 427 | 849 | 848 | 7 | 1028 |
| Pseudomonas soli | 0 | 0 | 0 | 0 | 0 |
| Salinispira pacifica | 79 | 143 | 126 | 11 | 158 |
| Kiritimatiella glycovorans | 1881 | 4936 | 3902 | 11 | 5139 |
| Salinivirga cyanobacteriivorans | 26 | 41 | 36 | 0 | 37 |
| Deinococcus soli (ex Cha et al. 2016) | 461 | 910 | 1148 | 7 | 1194 |
| Ralstonia pseudosolanacearum | 1353 | 1098 | 1547 | 26 | 1430 |
| Polaribacter sp. SA4-12 | 34 | 23 | 10 | 1 | 11 |
| Rhizobium jaguaris | 1311 | 1628 | 1267 | 39 | 1873 |
| Brevundimonas albigilva | 863 | 1200 | 1000 | 0 | 900 |
| Thermus sp. WG | 2 | 0 | 2 | 0 | 0 |
| Arcobacter aquimarinus | 0 | 5 | 2 | 0 | 4 |
| Sphingobium sp. TKS | 1411 | 2956 | 1844 | 14 | 3148 |
| Streptococcus sp. HSISM1 | 0 | 0 | 0 | 0 | 0 |
| Streptococcus sp. HSISS2 | 1 | 0 | 0 | 0 | 2 |
| Streptococcus sp. HSISS3 | 0 | 4 | 3 | 0 | 0 |
| Blattabacterium sp. (Nauphoeta cinerea) | 2 | 0 | 2 | 0 | 3 |
| Capnocytophaga sp. oral taxon 864 | 16 | 11 | 8 | 0 | 104 |
| Capnocytophaga sp. oral taxon 878 | 17 | 18 | 22 | 0 | 23 |
| Bartonella ancashensis | 20 | 27 | 31 | 0 | 18 |
| Afipia sp. NBIMC_P1-C1 | 0 | 0 | 0 | 0 | 0 |
| Anoxybacter fermentans | 30 | 55 | 61 | 0 | 79 |
| Streptomonospora nanhaiensis | 2356 | 2201 | 4454 | 592 | 10391 |
| Acinetobacter equi | 0 | 6 | 7 | 0 | 9 |
| Chryseobacterium gallinarum | 0 | 0 | 0 | 0 | 0 |
| Lysobacter lycopersici | 2204 | 1573 | 1400 | 43 | 1757 |
| [Pantoea] beijingensis | 69 | 115 | 111 | 1 | 112 |
| Bradyrhizobium sp. CCBAU 21365 | 179 | 685 | 265 | 0 | 365 |
| Bradyrhizobium guangdongense | 2672 | 2414 | 1611 | 0 | 1033 |
| Bradyrhizobium guangzhouense | 3108 | 2692 | 1392 | 0 | 728 |
| Bradyrhizobium sp. CCBAU 51753 | 9901 | 7836 | 5619 | 102 | 5302 |
| Bradyrhizobium sp. CCBAU 51765 | 1827 | 816 | 1020 | 0 | 0 |
| Bradyrhizobium zhanjiangense | 2385 | 2135 | 1737 | 38 | 2385 |
| Bradyrhizobium sp. CCBAU 53338 | 1092 | 1481 | 732 | 0 | 149 |
| Bradyrhizobium sp. CCBAU 53340 | 4410 | 3004 | 1996 | 0 | 1463 |
| Bradyrhizobium sp. CCBAU 53351 | 3133 | 2086 | 1254 | 0 | 293 |
| Bradyrhizobium guangxiense | 2781 | 2251 | 1256 | 0 | 972 |
| Bradyrhizobium sp. CCBAU 53421 | 3967 | 2778 | 1320 | 0 | 0 |
| Nitrospira japonica | 2121 | 3870 | 4115 | 16 | 3687 |
| Sphingomonas psychrotolerans | 948 | 1301 | 723 | 0 | 1271 |
| Serratia sp. FS14 | 6 | 23 | 77 | 0 | 102 |
| Vibrio cortegadensis | 14 | 29 | 19 | 2 | 33 |
| Enterobacter sp. MGH 14 | 0 | 8590 | 0 | 0 | 0 |
| Enterobacter sp. BIDMC 29 | 138 | 51 | 122 | 0 | 242 |
| Cycloclasticus zancles | 63 | 38 | 50 | 0 | 30 |
| Kosmotoga pacifica | 17 | 37 | 37 | 0 | 62 |
| Bordetella pseudohinzii | 764 | 990 | 1011 | 17 | 1357 |
| Brachybacterium ginsengisoli | 939 | 1059 | 1478 | 44 | 1679 |
| Thermogutta terrifontis | 540 | 1670 | 1395 | 12 | 2337 |
| Candidatus Saccharimonas aalborgensis | 0 | 0 | 0 | 0 | 0 |
| Tessaracoccus aquimaris | 1423 | 1490 | 2716 | 103 | 3948 |
| Streptomyces sp. GMY02 | 2377 | 1868 | 4316 | 204 | 6748 |
| Paenibacillus sophorae | 116 | 274 | 221 | 1 | 303 |
| Variibacter gotjawalensis | 6271 | 2919 | 2916 | 78 | 4493 |
| [Enterobacter] lignolyticus | 0 | 0 | 0 | 0 | 0 |
| Paradevosia shaoguanensis | 3314 | 2364 | 2288 | 72 | 2671 |
| Frigidibacter mobilis | 1314 | 1455 | 1947 | 0 | 2350 |
| Pseudorhizobium flavum | 0 | 0 | 0 | 0 | 0 |
| Gordonibacter urolithinfaciens | 425 | 658 | 977 | 8 | 1439 |
| Spiribacter salinus | 443 | 652 | 553 | 25 | 708 |
| Spiribacter curvatus | 420 | 807 | 844 | 24 | 979 |
| Formosa sp. Hel1_33_131 | 5 | 39 | 26 | 1 | 36 |
| Formosa sp. Hel3_A1_48 | 33 | 25 | 11 | 0 | 24 |
| Gillisia sp. Hel1_33_143 | 5 | 33 | 28 | 15 | 27 |
| Nonlabens sp. Hel1_33_55 | 12 | 150 | 28 | 0 | 59 |
| Polaribacter sp. Hel1_33_78 | 8 | 29 | 18 | 0 | 15 |
| Reinekea forsetii | 87 | 229 | 182 | 14 | 262 |
| Calothrix sp. 336/3 | 127 | 128 | 75 | 0 | 134 |
| Paenibacillus lentus | 80 | 493 | 180 | 0 | 173 |
| Pseudomonas sp. JY-Q | 0 | 20 | 49 | 0 | 82 |
| Pseudomonas sp. HN11 | 0 | 0 | 0 | 0 | 0 |
| Candidatus Annandia pinicola | 23 | 1 | 5 | 0 | 1 |
| Halomonas sp. A3H3 | 318 | 404 | 567 | 0 | 556 |
| Pseudoalteromonas piratica | 12 | 21 | 13 | 0 | 205 |
| Vallitalea pronyensis | 32 | 30 | 26 | 0 | 19 |
| Croceicoccus naphthovorans | 732 | 1270 | 604 | 1 | 420 |
| Aureimonas sp. AU20 | 2049 | 2263 | 1980 | 30 | 2508 |
| Synechococcus sp. CBW1002 | 702 | 392 | 599 | 34 | 846 |
| Synechococcus sp. CBW1004 | 520 | 895 | 803 | 33 | 1307 |
| Synechococcus sp. CBW1006 | 218 | 587 | 987 | 0 | 941 |
| Synechococcus sp. CBW1108 | 175 | 522 | 447 | 1 | 718 |
| Rhizobium freirei | 0 | 47 | 31 | 0 | 31 |
| Achromobacter pestifer | 1330 | 1513 | 1889 | 19 | 2811 |
| Achromobacter deleyi | 1679 | 1946 | 2339 | 12 | 2604 |
| Neochlamydia sp. S13 | 44 | 233 | 86 | 1 | 629 |
| Streptomyces pluripotens | 1673 | 1292 | 2640 | 386 | 6254 |
| Flavobacterium faecale | 63 | 86 | 55 | 0 | 44 |
| Aliarcobacter lanthieri | 26 | 3 | 16 | 0 | 7 |
| Bradyrhizobium diazoefficiens | 100752 | 41531 | 26059 | 0 | 17206 |
| Hymenobacter sp. APR13 | 865 | 954 | 935 | 27 | 1290 |
| Pseudomonas sp. LS44 | 0 | 0 | 0 | 0 | 0 |
| Richelia sinica | 20 | 77 | 39 | 0 | 60 |
| Arthrobacter sp. QXT-31 | 1021 | 1518 | 4220 | 106 | 8434 |
| Sphingopyxis sp. QXT-31 | 957 | 1333 | 1028 | 10 | 1781 |
| Rhizobium rosettiformans | 0 | 0 | 0 | 0 | 0 |
| Gemmatimonas phototrophica | 1837 | 6974 | 4808 | 79 | 19178 |
| Spirosoma radiotolerans | 52 | 820 | 537 | 0 | 551 |
| Roseivivax marinus | 1364 | 1330 | 1487 | 124 | 1879 |
| Rufibacter sp. DG15C | 0 | 0 | 0 | 0 | 0 |
| Rufibacter radiotolerans | 0 | 0 | 0 | 0 | 0 |
| Kroppenstedtia pulmonis | 73 | 174 | 131 | 0 | 162 |
| Vibrio panuliri | 39 | 106 | 40 | 23 | 72 |
| Sphingomonas sp. ERG5 | 25 | 11 | 24 | 0 | 9 |
| Winogradskyella sediminis | 25 | 33 | 39 | 0 | 27 |
| Muricauda aurantiaca | 9 | 28 | 32 | 0 | 27 |
| Methyloceanibacter caenitepidi | 2425 | 1753 | 1809 | 0 | 1895 |
| Burkholderia mayonis | 810 | 1213 | 1556 | 1 | 1524 |
| Hymenobacter sp. DG25A | 0 | 0 | 0 | 0 | 0 |
| Hymenobacter sp. DG25B | 0 | 0 | 0 | 0 | 0 |
| Hymenobacter qilianensis | 0 | 0 | 0 | 0 | 0 |
| Synechococcus sp. MVIR-18-1 | 8 | 71 | 100 | 28 | 76 |
| Paludisphaera borealis | 7999 | 37120 | 18989 | 367 | 29186 |
| Caldicellulosiruptor morganii | 38 | 16 | 12 | 0 | 16 |
| Candidatus Pelagibacter sp. HIMB1321 | 19 | 15 | 7 | 0 | 13 |
| Sulfitobacter sp. SK011 | 0 | 0 | 0 | 0 | 0 |
| Sulfitobacter sp. SK012 | 146 | 198 | 229 | 4 | 304 |
| Sulfitobacter sp. SK025 | 34 | 115 | 128 | 5 | 280 |
| Agromyces marinus | 2026 | 2190 | 3366 | 57 | 4421 |
| Cupriavidus sp. USMAHM13 | 0 | 0 | 0 | 0 | 0 |
| Mycobacterium paragordonae | 2352 | 4532 | 3486 | 101 | 6727 |
| Achromobacter mucicolens | 751 | 1306 | 1419 | 52 | 2036 |
| Bradyrhizobium sp. WBAH10 | 0 | 0 | 1160 | 0 | 0 |
| Bradyrhizobium sp. WBAH23 | 1126 | 0 | 0 | 0 | 1308 |
| Bradyrhizobium sp. WBAH42 | 0 | 0 | 0 | 0 | 4926 |
| Pseudoalteromonas sp. DL-6 | 1 | 20 | 1 | 0 | 8 |
| Sphingomonas sp. LK11 | 463 | 949 | 544 | 3 | 1078 |
| Vulgatibacter incomptus | 2484 | 4078 | 5102 | 44 | 5078 |
| Labilithrix luteola | 3271 | 6919 | 6776 | 45 | 6486 |
| Pseudomonas oryzae | 675 | 1264 | 1501 | 11 | 1876 |
| Thermosynechococcus sp. NK55a | 15 | 124 | 138 | 0 | 247 |
| Francisella sp. LA112445 | 4 | 4 | 17 | 0 | 11 |
| Leisingera aquaemixtae | 536 | 727 | 750 | 0 | 626 |
| Celeribacter marinus | 111 | 257 | 131 | 0 | 200 |
| Brasilonema sennae | 116 | 250 | 164 | 0 | 208 |
| Exiguobacterium sp. MH3 | 0 | 0 | 0 | 0 | 0 |
| Bythopirellula goksoeyrii | 1472 | 2765 | 1874 | 14 | 3373 |
| Candidatus Competibacter denitrificans | 4 | 14 | 22 | 0 | 41 |
| Synechococcus sp. BIOS-E4-1 | 74 | 159 | 188 | 0 | 227 |
| Synechococcus sp. BIOS-U3-1 | 49 | 247 | 128 | 0 | 171 |
| Synechococcus sp. PROS-U-1 | 91 | 166 | 152 | 0 | 236 |
| Dickeya aquatica | 0 | 91 | 0 | 0 | 0 |
| Clavibacter californiensis | 499 | 665 | 1001 | 31 | 1501 |
| Pseudosulfitobacter pseudonitzschiae | 1155 | 1236 | 1407 | 71 | 1511 |
| Priestia filamentosa | 2 | 58 | 50 | 0 | 38 |
| Bradyrhizobium symbiodeficiens | 3560 | 3402 | 2512 | 0 | 2946 |
| Bradyrhizobium septentrionale | 5721 | 5239 | 3300 | 0 | 1436 |
| Bradyrhizobium sp. 41S5 | 2666 | 1899 | 704 | 0 | 0 |
| Tardiphaga sp. 37S4 | 2960 | 1345 | 481 | 0 | 0 |
| Bradyrhizobium amphicarpaeae | 2096 | 1890 | 1547 | 0 | 1523 |
| Bradyrhizobium cosmicum | 3678 | 2380 | 1988 | 4 | 2041 |
| Bradyrhizobium sp. 1(2017) | 3092 | 2623 | 1953 | 0 | 1801 |
| Brevibacterium sp. Ap13 | 2 | 5 | 2 | 0 | 2 |
| Acinetobacter sp. TGL-Y2 | 3 | 7 | 18 | 0 | 133 |
| Corynebacterium deserti | 107 | 256 | 149 | 3 | 278 |
| Candidatus Endomicrobium trichonymphae | 31 | 68 | 49 | 0 | 55 |
| Endomicrobium proavitum | 32 | 17 | 20 | 3 | 40 |
| Candidatus Tachikawaea gelatinosa | 4 | 4 | 7 | 0 | 6 |
| Thermomonospora amylolytica | 5878 | 3891 | 10715 | 2518 | 39448 |
| Pedobacter sp. | 0 | 1 | 0 | 0 | 0 |
| Hymenobacter sedentarius | 302 | 975 | 810 | 46 | 1156 |
| Pacificitalea manganoxidans | 806 | 885 | 960 | 13 | 1173 |
| Streptomyces ferrugineus | 2658 | 2140 | 3556 | 257 | 6207 |
| Corynebacterium jeddahense | 544 | 945 | 1455 | 0 | 693 |
| Marinobacter sp. LV10R510-11A | 0 | 0 | 0 | 0 | 0 |
| Candidatus Walczuchella monophlebidarum | 11 | 6 | 7 | 0 | 25 |
| Gloeobacter kilaueensis | 676 | 1538 | 1393 | 12 | 1680 |
| Oceanisphaera profunda | 106 | 98 | 102 | 90 | 149 |
| Bordetella genomosp. 9 | 1500 | 1912 | 2276 | 53 | 3304 |
| Bordetella genomosp. 8 | 1448 | 1728 | 2026 | 41 | 3367 |
| Marinobacter similis | 138 | 275 | 247 | 17 | 325 |
| Marinobacter salarius | 414 | 754 | 515 | 5 | 607 |
| Pseudomonas granadensis | 0 | 0 | 0 | 0 | 0 |
| Geosporobacter ferrireducens | 44 | 62 | 43 | 2 | 73 |
| Caminibacter pacificus | 15 | 23 | 16 | 0 | 38 |
| Hyphomicrobium nitrativorans | 6929 | 3336 | 3538 | 77 | 3351 |
| Candidatus Pseudothioglobus singularis | 0 | 36 | 17 | 0 | 36 |
| Methylibium sp. T29 | 0 | 35 | 0 | 0 | 58 |
| Mycolicibacterium anyangense | 1661 | 4537 | 3352 | 131 | 7630 |
| Mannheimia sp. USDA-ARS-USMARC-1261 | 0 | 19 | 0 | 0 | 8 |
| Methylocaldum marinum | 891 | 1445 | 1260 | 14 | 1758 |
| Halopseudomonas salegens | 247 | 295 | 344 | 7 | 431 |
| Vibrio tritonius | 5 | 94 | 23 | 0 | 12 |
| Yoonia maritima | 0 | 4 | 4 | 0 | 11 |
| Streptomyces sp. F12 | 15 | 65 | 40 | 0 | 100 |
| Streptomyces sp. F8 | 64 | 53 | 210 | 9 | 530 |
| Actinacidiphila bryophytorum | 3161 | 2350 | 5608 | 351 | 7668 |
| Candidatus Symbiobacter mobilis | 0 | 0 | 0 | 0 | 0 |
| Chenggangzhangella methanolivorans | 4370 | 3525 | 3233 | 118 | 5164 |
| Bradyrhizobium erythrophlei | 30738 | 27636 | 18899 | 0 | 17899 |
| Thermosipho sp. 1070 | 0 | 0 | 23 | 0 | 34 |
| Streptomyces leeuwenhoekii | 1089 | 1142 | 2388 | 118 | 3116 |
| Citrobacter sp. MGH 55 | 8 | 32 | 36 | 0 | 72 |
| Bacillus gobiensis | 0 | 0 | 0 | 0 | 0 |
| Athalassotoga saccharophila | 32 | 57 | 54 | 2 | 79 |
| Streptomyces dangxiongensis | 1740 | 1302 | 2606 | 361 | 7076 |
| Ectothiorhodospira sp. BSL-9 | 539 | 753 | 777 | 4 | 935 |
| Streptomyces sp. 14R-10 | 20 | 11 | 12 | 0 | 7 |
| Synechococcus sp. BMK-MC-1 | 0 | 0 | 0 | 0 | 0 |
| Synechococcus sp. M16.1 | 12 | 136 | 121 | 0 | 136 |
| Synechococcus sp. MEDNS5 | 149 | 185 | 121 | 9 | 160 |
| Synechococcus sp. NOUM97013 | 47 | 177 | 165 | 3 | 204 |
| Synechococcus sp. PROS-7-1 | 116 | 158 | 160 | 0 | 173 |
| Synechococcus sp. ROS8604 | 66 | 91 | 93 | 0 | 98 |
| Synechococcus sp. TAK9802 | 0 | 0 | 0 | 0 | 0 |
| Vespertiliibacter pulmonis | 0 | 1 | 5 | 0 | 1 |
| Nitrosomonas stercoris | 85 | 185 | 129 | 0 | 248 |
| Xylella taiwanensis | 91 | 165 | 161 | 1 | 168 |
| Gynuella sunshinyii | 119 | 182 | 192 | 11 | 274 |
| Hymenobacter swuensis | 0 | 0 | 0 | 0 | 0 |
| Frondihabitans sp. 762G35 | 982 | 1498 | 1691 | 72 | 2753 |
| Youhaiella tibetensis | 2164 | 2064 | 1653 | 20 | 2239 |
| Campylobacter corcagiensis | 1 | 2 | 2 | 2 | 9 |
| Ochrobactrum sp. LM19 | 0 | 6 | 10 | 0 | 2 |
| Sinorhizobium sp. LM21 | 11 | 1 | 11 | 0 | 4 |
| Aneurinibacillus sp. XH2 | 0 | 127 | 0 | 0 | 32 |
| Paenibacillus kyungheensis | 25 | 59 | 28 | 0 | 24 |
| Sediminicola sp. YIK13 | 21 | 36 | 16 | 3 | 21 |
| Chlamydia avium | 28 | 14 | 6 | 0 | 19 |
| Chlamydia gallinacea | 11 | 19 | 6 | 0 | 7 |
| Cyclonatronum proteinivorum | 175 | 647 | 310 | 15 | 659 |
| Octadecabacter temperatus | 104 | 80 | 122 | 16 | 163 |
| Mixta theicola | 198 | 260 | 245 | 1 | 458 |
| Serpentinimonas raichei | 733 | 703 | 956 | 0 | 1289 |
| Serpentinimonas maccroryi | 453 | 722 | 761 | 8 | 858 |
| Candidatus Phaeomarinobacter ectocarpi | 715 | 629 | 528 | 0 | 728 |
| Myroides sp. ZB35 | 19 | 4 | 0 | 0 | 0 |
| Pseudooceanicola atlanticus | 682 | 759 | 810 | 9 | 1163 |
| Oceanobacillus jeddahense | 5 | 116 | 30 | 0 | 16 |
| Halarcobacter ebronensis | 0 | 9 | 2 | 3 | 5 |
| Paenibacillus yonginensis | 62 | 299 | 212 | 1 | 229 |
| Thermomonas carbonis | 1130 | 2302 | 1476 | 7 | 1437 |
| Klebsiella quasipneumoniae | 1673 | 3740 | 3188 | 51 | 2656 |
| Burkholderia sp. 2002721687 | 0 | 136 | 0 | 0 | 1719 |
| Carbonactinospora thermoautotrophica | 1 | 2 | 5 | 0 | 12 |
| Actinoalloteichus hoggarensis | 3692 | 1480 | 4411 | 172 | 4985 |
| Neisseria arctica | 17 | 41 | 38 | 0 | 73 |
| Alistipes ihumii | 237 | 563 | 508 | 9 | 595 |
| Zhongshania aliphaticivorans | 89 | 117 | 119 | 9 | 171 |
| Corynebacterium pelargi | 0 | 0 | 0 | 0 | 0 |
| Novibacillus thermophilus | 142 | 433 | 282 | 2 | 427 |
| Basilea psittacipulmonis | 0 | 0 | 0 | 0 | 0 |
| Lentibacillus amyloliquefaciens | 18 | 55 | 19 | 0 | 64 |
| Paenibacillus guangzhouensis | 85 | 218 | 156 | 0 | 260 |
| Paludibaculum fermentans | 4415 | 11275 | 8065 | 121 | 10088 |
| Alicyclobacillus dauci | 82 | 197 | 295 | 1 | 335 |
| Heyndrickxia vini | 3 | 23 | 24 | 0 | 44 |
| Streptomyces sp. 604F | 584 | 353 | 418 | 9 | 528 |
| Nonlabens sp. MIC269 | 0 | 4 | 4 | 0 | 45 |
| Arthrobacter sp. D5-1 | 601 | 586 | 8294 | 70 | 2311 |
| Cupriavidus sp. KK10 | 834 | 1092 | 1198 | 4 | 1568 |
| Methylobacterium sp. C1 | 1847 | 1804 | 1872 | 0 | 1720 |
| Vibrio metoecus | 13 | 78 | 72 | 4 | 99 |
| Vibrio ishigakensis | 59 | 127 | 43 | 1 | 67 |
| Vibrio astriarenae | 9 | 50 | 44 | 1 | 57 |
| Hartmannibacter diazotrophicus | 2890 | 2769 | 2268 | 21 | 2919 |
| Kutzneria chonburiensis | 17957 | 5147 | 17004 | 363 | 13515 |
| Lichenicola cladoniae | 1288 | 1696 | 1756 | 7 | 2221 |
| Hymenobacter psoromatis | 432 | 755 | 709 | 3 | 834 |
| Hymenobacter sp. PAMC 26628 | 422 | 999 | 956 | 12 | 1398 |
| Rhodoferax saidenbachensis | 0 | 0 | 0 | 0 | 0 |
| Lacinutrix venerupis | 18 | 20 | 12 | 0 | 6 |
| Gramella flava | 21 | 76 | 73 | 2 | 117 |
| Martelella endophytica | 1185 | 1038 | 1106 | 41 | 1809 |
| Candidatus Sodalis pierantonius | 83 | 150 | 201 | 0 | 271 |
| Corynebacterium sp. ATCC 6931 | 1116 | 645 | 379 | 0 | 1749 |
| Streptomyces sp. MBT27 | 2147 | 2038 | 3851 | 216 | 5919 |
| Anoxybacillus sp. B7M1 | 0 | 0 | 0 | 0 | 88 |
| Flavobacterium gilvum | 40 | 261 | 115 | 0 | 213 |
| Chryseobacterium shandongense | 0 | 0 | 0 | 0 | 0 |
| Burkholderia sp. FERM BP-3421 | 1661 | 1944 | 2554 | 18 | 3155 |
| Arthrobacter sp. PAMC 25486 | 411 | 585 | 895 | 56 | 1364 |
| Pseudomonas capeferrum | 7 | 47 | 49 | 0 | 275 |
| Pseudomonas sp. WCS374 | 21 | 67 | 39 | 176 | 73 |
| Streptomyces sp. M56 | 1921 | 1155 | 3468 | 34 | 3525 |
| Brevibacterium pigmentatum | 0 | 0 | 0 | 0 | 0 |
| Listeria newyorkensis | 1 | 64 | 41 | 1 | 31 |
| Paracoccus mutanolyticus | 642 | 794 | 909 | 0 | 1177 |
| Pseudomonas saudiphocaensis | 0 | 0 | 0 | 0 | 0 |
| Planococcus massiliensis | 46 | 168 | 60 | 0 | 91 |
| Escherichia marmotae | 0 | 0 | 0 | 0 | 0 |
| Aneurinibacillus soli | 0 | 0 | 0 | 0 | 0 |
| Pseudomonas sp. Os17 | 86 | 248 | 143 | 128 | 655 |
| Pseudomonas sp. St29 | 58 | 106 | 136 | 0 | 246 |
| Campylobacter sp. RM16704 | 0 | 0 | 0 | 0 | 0 |
| Prochlorococcus sp. MIT 0604 | 1 | 10 | 0 | 0 | 6 |
| Prochlorococcus sp. MIT 0801 | 1 | 9 | 8 | 0 | 23 |
| Coprobacter secundus | 53 | 67 | 73 | 0 | 52 |
| Arcanobacterium pinnipediorum | 8 | 54 | 37 | 2 | 90 |
| Burkholderia stagnalis | 765 | 913 | 1267 | 20 | 1301 |
| Burkholderia territorii | 0 | 0 | 0 | 0 | 0 |
| Halomonas sp. KO116 | 0 | 0 | 0 | 0 | 0 |
| Blochmannia endosymbiont of Polyrhachis (Hedomyrma) turneri | 0 | 9 | 2 | 0 | 5 |
| Blochmannia endosymbiont of Camponotus (Colobopsis) obliquus | 18 | 6 | 5 | 0 | 8 |
| Siccibacter colletis | 0 | 0 | 0 | 0 | 0 |
| Jeotgalibacillus malaysiensis | 8 | 45 | 65 | 0 | 50 |
| Thermotoga caldifontis | 70 | 75 | 132 | 0 | 305 |
| Thermotoga profunda | 6 | 57 | 76 | 0 | 75 |
| Candidatus Arthromitus sp. SFB-mouse-NL | 0 | 0 | 0 | 0 | 0 |
| Candidatus Nucleicultrix amoebiphila | 43 | 72 | 54 | 0 | 63 |
| Permianibacter aggregans | 375 | 579 | 567 | 1 | 684 |
| Parasaccharibacter apium | 24 | 65 | 52 | 0 | 84 |
| Leptospira sp. GIMC2001 | 9 | 58 | 25 | 3 | 43 |
| Rouxiella chamberiensis | 198 | 333 | 232 | 15 | 453 |
| Pseudoalteromonas sp. NC201 | 3 | 16 | 5 | 0 | 2 |
| Erysipelothrix larvae | 5 | 14 | 11 | 0 | 50 |
| Sphingopyxis fribergensis | 889 | 1616 | 801 | 50 | 601 |
| Microbulbifer sp. ALW1 | 253 | 398 | 244 | 1 | 312 |
| Vibrio coralliirubri | 1 | 9 | 20 | 0 | 17 |
| Sphingomonas sp. HMP9 | 43 | 1619 | 988 | 0 | 492 |
| Corallococcus sp. EGB | 1791 | 2983 | 3122 | 21 | 3840 |
| Bradyrhizobium sp. WD16 | 6619 | 4621 | 4434 | 87 | 5736 |
| Dehalococcoides sp. UCH007 | 0 | 0 | 62 | 0 | 117 |
| Streptomyces sp. ADI95-16 | 1515 | 1046 | 2571 | 153 | 3684 |
| Pseudomonas sp. ZM1 | 0 | 0 | 0 | 2 | 0 |
| Aliamphritea ceti | 97 | 71 | 91 | 0 | 112 |
| Pseudomonas sp. EGD-AKN5 | 3917 | 0 | 0 | 0 | 0 |
| Sulfitobacter sp. CB2047 | 98 | 202 | 231 | 0 | 365 |
| Microbacterium endophyticum | 246 | 559 | 700 | 27 | 902 |
| Lacimicrobium alkaliphilum | 108 | 186 | 142 | 9 | 256 |
| Aquisalinus flavus | 772 | 850 | 714 | 41 | 1123 |
| Bosea vaviloviae | 4598 | 3766 | 3170 | 13 | 3765 |
| Planococcus sp. PAMC 21323 | 0 | 0 | 0 | 0 | 0 |
| Paracoccus tegillarcae | 0 | 0 | 0 | 0 | 0 |
| Polaribacter sp. BM10 | 2 | 14 | 11 | 0 | 21 |
| Acinetobacter seifertii | 0 | 0 | 0 | 0 | 0 |
| Berryella intestinalis | 1104 | 2438 | 2483 | 19 | 1907 |
| Anaerobacillus isosaccharinicus | 15 | 45 | 119 | 3 | 34 |
| Paenibacillus sp. CAA11 | 42 | 212 | 94 | 1 | 276 |
| Pseudomonas sp. DR 5-09 | 161 | 143 | 128 | 31 | 345 |
| Mycolicibacterium sarraceniae | 1062 | 3214 | 2345 | 12 | 4577 |
| Mycolicibacterium helvum | 1524 | 4307 | 3007 | 51 | 6939 |
| Vibrio hyugaensis | 1 | 6 | 10 | 0 | 19 |
| Metabacillus endolithicus | 15 | 15 | 15 | 0 | 19 |
| Streptomyces sp. OUCMDZ-3434 | 596 | 402 | 1290 | 112 | 1090 |
| Streptomyces lunaelactis | 2948 | 3101 | 6535 | 238 | 14965 |
| Candidatus Dehalogenimonas etheniformans | 164 | 374 | 458 | 1 | 633 |
| Paenibacillus sp. FSL P4-0081 | 137 | 280 | 144 | 12 | 335 |
| Paenibacillus sp. FSL R5-0345 | 9 | 40 | 62 | 0 | 102 |
| Paenibacillus sp. FSL R5-0912 | 0 | 0 | 0 | 0 | 0 |
| Paenibacillus sp. FSL R7-0273 | 82 | 294 | 211 | 1 | 322 |
| Paenibacillus sp. FSL R7-0331 | 108 | 175 | 235 | 1 | 287 |
| Paenibacillus sp. FSL H7-0357 | 110 | 264 | 263 | 4 | 367 |
| Paenibacillus sp. FSL H7-0737 | 10 | 61 | 78 | 0 | 48 |
| Pseudooceanicola algae | 0 | 0 | 0 | 0 | 0 |
| Janthinobacterium sp. HH102 | 218 | 698 | 519 | 0 | 4225 |
| Aquitalea aquatilis | 0 | 0 | 0 | 0 | 0 |
| Rhizobium acidisoli | 0 | 0 | 0 | 0 | 0 |
| Rhizobium hidalgonense | 387 | 382 | 420 | 0 | 255 |
| Methylomonas denitrificans | 255 | 455 | 462 | 8 | 609 |
| Sphingobacterium sp. ML3W | 18 | 64 | 34 | 10 | 55 |
| Treponema sp. OMZ 838 | 12 | 19 | 0 | 0 | 14 |
| Sphingomonas morindae | 1023 | 1265 | 976 | 49 | 1606 |
| Francisella frigiditurris | 0 | 24 | 7 | 0 | 5 |
| Frateuria soli | 1842 | 1840 | 1998 | 39 | 2040 |
| Sedimenticola thiotaurini | 325 | 504 | 502 | 15 | 612 |
| Kribbella qitaiheensis | 7117 | 5559 | 26739 | 284 | 17531 |
| Lactobacillus sp. wkB8 | 0 | 20 | 5 | 0 | 8 |
| Mycobacterium sp. EPa45 | 1359 | 3599 | 2787 | 49 | 5853 |
| Marinitoga sp. 1137 | 0 | 0 | 0 | 0 | 0 |
| Streptomyces lydicamycinicus | 953 | 1074 | 1678 | 158 | 2451 |
| Bacillus weihaiensis | 1 | 19 | 23 | 0 | 36 |
| Francisella sp. FSC1006 | 0 | 6 | 7 | 0 | 4 |
| Arachnia rubra | 440 | 668 | 958 | 46 | 1299 |
| Woeseia oceani | 927 | 941 | 825 | 0 | 931 |
| Bradymonas sediminis | 502 | 1363 | 1156 | 14 | 1598 |
| Nesterenkonia pannonica | 0 | 0 | 0 | 0 | 0 |
| Thioclava electrotropha | 628 | 798 | 745 | 2 | 1020 |
| Haematospirillum jordaniae | 123 | 300 | 213 | 0 | 266 |
| Sphingomonas taxi | 539 | 971 | 741 | 0 | 339 |
| Bradyrhizobium vignae | 2886 | 2521 | 1905 | 0 | 2426 |
| Ruthenibacterium lactatiformans | 0 | 0 | 0 | 0 | 0 |
| Thermoanaerobacterium sp. RBIITD | 22 | 27 | 47 | 0 | 36 |
| Mucilaginibacter gotjawali | 0 | 0 | 0 | 0 | 0 |
| Blastomonas fulva | 0 | 0 | 0 | 0 | 0 |
| Aquisediminimonas profunda | 0 | 0 | 0 | 0 | 0 |
| Mycobacterium grossiae | 3741 | 7477 | 6332 | 82 | 11355 |
| Mycoavidus cysteinexigens | 33 | 201 | 141 | 0 | 183 |
| Limnochorda pilosa | 1110 | 2247 | 2658 | 38 | 3427 |
| Enterobacter sp. E20 | 369 | 118 | 385 | 0 | 212 |
| Candidatus Ichthyocystis hellenicum | 0 | 0 | 0 | 0 | 0 |
| Acididesulfobacillus acetoxydans | 134 | 313 | 242 | 23 | 522 |
| Streptomyces sp. CCM_MD2014 | 1274 | 1154 | 1668 | 109 | 2715 |
| Curtobacterium sp. MR_MD2014 | 640 | 883 | 1187 | 26 | 1485 |
| Kangiella profundi | 3 | 210 | 40 | 1 | 37 |
| Citrobacter pasteurii | 60 | 82 | 71 | 0 | 170 |
| Agarivorans aestuarii | 6 | 24 | 46 | 2 | 64 |
| Rhodococcus sp. B7740 | 0 | 0 | 0 | 0 | 0 |
| Aliarcobacter faecis | 0 | 4 | 0 | 0 | 4 |
| Carnobacterium sp. CP1 | 0 | 12 | 20 | 0 | 23 |
| Rugosibacter aromaticivorans | 318 | 1024 | 843 | 2 | 1222 |
| Desulfuromonas sp. AOP6 | 229 | 784 | 783 | 1 | 943 |
| Paenibacillus sp. IHBB 10380 | 11 | 64 | 54 | 0 | 86 |
| Bradyrhizobium sp. UASWS1016 | 0 | 0 | 0 | 0 | 0 |
| Phaeobacter sp. S60 | 0 | 5 | 0 | 0 | 0 |
| Rhodococcus sp. 2G | 97 | 301 | 270 | 0 | 412 |
| Rhizobium sp. ACO-34A | 1222 | 916 | 943 | 28 | 1276 |
| Neptunomonas phycophila | 8 | 52 | 40 | 8 | 59 |
| Citrobacter sp. R56 | 150 | 224 | 212 | 9 | 587 |
| Pseudomonas sp. R32 | 57 | 121 | 112 | 0 | 171 |
| Pseudomonas sp. R76 | 183 | 166 | 196 | 2 | 450 |
| Pseudomonas sp. R84 | 66 | 291 | 248 | 0 | 3264 |
| Pseudomonas sp. S34 | 253 | 703 | 1920 | 67 | 6604 |
| Pseudomonas sp. S35 | 0 | 0 | 0 | 0 | 0 |
| Pseudomonas sp. S49 | 132 | 227 | 242 | 3 | 1080 |
| Proteus terrae | 39 | 93 | 169 | 62 | 128 |
| Polaribacter undariae | 0 | 9 | 5 | 0 | 6 |
| Candidatus Kinetoplastibacterium sorsogonicusi | 2 | 7 | 12 | 0 | 11 |
| Streptomyces sp. PBH53 | 774 | 710 | 1280 | 50 | 1700 |
| Helicobacter ailurogastricus | 34 | 19 | 30 | 0 | 33 |
| Edwardsiella sp. EA181011 | 262 | 841 | 121 | 5 | 33 |
| Falsihalocynthiibacter arcticus | 0 | 0 | 0 | 0 | 0 |
| Wenzhouxiangella marina | 997 | 1964 | 1808 | 38 | 2256 |
| Phaeobacter piscinae | 388 | 528 | 488 | 8 | 874 |
| Sulfurospirillum sp. UCH001 | 2 | 8 | 1 | 0 | 7 |
| Bacillus sp. FJAT-22090 | 11 | 69 | 27 | 0 | 39 |
| Candidatus Methylopumilus planktonicus | 32 | 33 | 62 | 3 | 43 |
| Candidatus Methylopumilus turicensis | 7 | 67 | 29 | 3 | 66 |
| Gudongella oleilytica | 53 | 83 | 119 | 20 | 112 |
| Sphingomicrobium aestuariivivum | 705 | 1042 | 737 | 5 | 1154 |
| Fusobacterium hwasookii | 4 | 18 | 11 | 0 | 9 |
| Myroides sp. A21 | 0 | 13 | 7 | 0 | 8 |
| Pseudomonas cerasi | 78 | 142 | 79 | 2 | 201 |
| Bergeyella cardium | 12 | 207 | 16 | 0 | 37 |
| Lentzea guizhouensis | 33779 | 10133 | 24723 | 411 | 15650 |
| Helicobacter himalayensis | 16 | 13 | 17 | 0 | 18 |
| Pseudohalocynthiibacter aestuariivivens | 165 | 810 | 435 | 4 | 604 |
| Terriglobus albidus | 1296 | 3033 | 2547 | 27 | 3097 |
| Sphingobium phenoxybenzoativorans | 1542 | 1589 | 1373 | 13 | 2329 |
| Coxiella-like endosymbiont | 60 | 31 | 45 | 0 | 26 |
| Massilia sp. YMA4 | 1416 | 2516 | 2528 | 58 | 5291 |
| Planococcus faecalis | 0 | 12 | 6 | 0 | 19 |
| Pseudomonas sp. St290 | 35 | 141 | 107 | 0 | 247 |
| Vibrio sp. qd031 | 0 | 0 | 12 | 0 | 5 |
| Desulfuromonas soudanensis | 829 | 1663 | 1686 | 27 | 2275 |
| Bifidobacterium lemurum | 144 | 295 | 375 | 10 | 552 |
| Yersinia rochesterensis | 0 | 195 | 0 | 14 | 24 |
| Lysobacter maris | 1810 | 1778 | 2053 | 6 | 2002 |
| Pseudomonas sp. LFM046 | 0 | 4 | 8 | 0 | 10 |
| Acinetobacter sp. NCu2D-2 | 20 | 17 | 16 | 7 | 86 |
| Candidatus Filomicrobium marinum | 1751 | 1028 | 1016 | 31 | 968 |
| Euzebya pacifica | 2184 | 2710 | 4163 | 129 | 4168 |
| Chryseobacterium rhizoplanae | 5 | 14 | 6 | 0 | 28 |
| endosymbiont DhMRE of Dentiscutata heterogama | 0 | 0 | 4 | 0 | 1 |
| Novosphingobium sp. P6W | 748 | 2488 | 1090 | 9 | 3694 |
| Actibacterium sp. EMB200-NS6 | 0 | 0 | 0 | 0 | 0 |
| Halomonas sp. HG01 | 498 | 714 | 850 | 5 | 1061 |
| Sphingomonas hengshuiensis | 724 | 1109 | 582 | 30 | 578 |
| Tessaracoccus flavus | 239 | 532 | 1209 | 57 | 884 |
| Halomonas sp. R57-5 | 201 | 201 | 250 | 6 | 271 |
| Pseudomonas sp. MRSN 12121 | 433 | 286 | 391 | 6 | 563 |
| Mycoplasma tullyi | 0 | 1 | 0 | 0 | 1 |
| Niveispirillum cyanobacteriorum | 1120 | 1352 | 1336 | 17 | 2291 |
| Actinoalloteichus sp. GBA129-24 | 3683 | 1134 | 3973 | 114 | 4119 |
| Actinoalloteichus fjordicus | 1843 | 1041 | 2355 | 333 | 6105 |
| Pararhizobium polonicum | 1 | 19 | 35 | 0 | 25 |
| Pseudomonas lactis | 0 | 0 | 0 | 0 | 0 |
| Geminocystis sp. NIES-3708 | 1 | 24 | 12 | 0 | 22 |
| Streptomyces formicae | 4012 | 3120 | 6516 | 251 | 10262 |
| Paenibacillus bovis | 0 | 0 | 0 | 0 | 0 |
| Flavobacterium nitrogenifigens | 32 | 156 | 160 | 0 | 68 |
| Geminocystis sp. NIES-3709 | 26 | 42 | 24 | 0 | 39 |
| Granulicella sp. 5B5 | 1271 | 3250 | 3220 | 23 | 2986 |
| Nostoc sp. 'Lobaria pulmonaria (5183) cyanobiont' | 93 | 545 | 167 | 0 | 344 |
| Psychromicrobium lacuslunae | 246 | 280 | 468 | 14 | 655 |
| Cohnella sp. LGH | 398 | 954 | 723 | 2 | 902 |
| Cnuibacter physcomitrellae | 1672 | 1730 | 2754 | 43 | 3193 |
| Paenibacillus physcomitrellae | 156 | 345 | 165 | 8 | 248 |
| Duffyella gerundensis | 269 | 344 | 279 | 45 | 517 |
| Sulfuricaulis limicola | 1788 | 2359 | 2300 | 34 | 3214 |
| Spongiibacter sp. IMCC21906 | 51 | 140 | 130 | 5 | 215 |
| Hoeflea sp. IMCC20628 | 852 | 855 | 629 | 0 | 879 |
| Candidatus Desulfofervidus auxilii | 60 | 117 | 165 | 0 | 220 |
| Lutibacter profundi | 8 | 11 | 13 | 4 | 11 |
| Sphingomonas sp. JE1 | 19 | 26 | 12 | 0 | 23 |
| Pseudomonas kribbensis | 0 | 0 | 0 | 0 | 0 |
| Empedobacter stercoris | 17 | 26 | 19 | 2 | 20 |
| Hahella sp. KA22 | 338 | 442 | 464 | 26 | 485 |
| Dermabacter vaginalis | 0 | 0 | 0 | 0 | 0 |
| Candidatus Nitrosoglobus terrae | 16 | 38 | 37 | 4 | 25 |
| Tamlana sp. s12 | 32 | 24 | 11 | 0 | 14 |
| Gemmata sp. SH-PL17 | 5260 | 57910 | 21755 | 226 | 25041 |
| Weissella jogaejeotgali | 2 | 11 | 1 | 0 | 10 |
| Hydrogenobacter sp. T-8 | 26 | 94 | 56 | 3 | 59 |
| Planctomyces sp. SH-PL14 | 4424 | 16857 | 10544 | 146 | 15931 |
| Pirellula sp. SH-Sr6A | 426 | 1361 | 1031 | 14 | 1753 |
| Alkalimarinus sediminis | 49 | 47 | 43 | 34 | 49 |
| Acetobacter oryzifermentans | 53 | 164 | 4 | 0 | 30 |
| Citromicrobium sp. JL477 | 649 | 1035 | 979 | 13 | 1426 |
| Zophobihabitans entericus | 5 | 18 | 20 | 0 | 23 |
| Planctomyces sp. SH-PL62 | 6492 | 33756 | 16969 | 242 | 26366 |
| Burkholderia sp. MSMB1588 | 0 | 0 | 0 | 0 | 0 |
| Acinetobacter sp. ACNIH1 | 0 | 0 | 0 | 0 | 0 |
| Aeromonas sp. ASNIH1 | 170 | 213 | 333 | 0 | 736 |
| Aeromonas sp. ASNIH2 | 0 | 218 | 403 | 0 | 2358 |
| Aeromonas sp. ASNIH3 | 74 | 206 | 253 | 367 | 216 |
| Aeromonas sp. ASNIH4 | 0 | 0 | 0 | 0 | 0 |
| Pseudomonas sp. PONIH3 | 506 | 645 | 666 | 0 | 887 |
| Anoxybacillus sp. PDR2 | 0 | 125 | 105 | 0 | 44 |
| Burkholderia sp. MSMB617WGS | 45 | 102 | 192 | 1 | 219 |
| Burkholderia savannae | 233 | 298 | 483 | 6 | 495 |
| Burkholderia sp. LA-2-3-30-S1-D2 | 0 | 0 | 0 | 0 | 0 |
| Burkholderia sp. MSMB0856 | 370 | 560 | 648 | 11 | 815 |
| Cytobacillus solani | 14 | 138 | 105 | 0 | 69 |
| Aureimonas sp. AU12 | 27 | 62 | 27 | 0 | 59 |
| Aureimonas sp. N4 | 0 | 0 | 132 | 0 | 0 |
| Chania multitudinisentens | 120 | 127 | 112 | 2 | 163 |
| Citrobacter portucalensis | 0 | 0 | 0 | 0 | 0 |
| Magnetospirillum sp. ME-1 | 1316 | 1582 | 1756 | 27 | 2391 |
| Pseudonocardia sp. HH130629-09 | 3167 | 1874 | 4645 | 175 | 6091 |
| Streptomyces alfalfae | 3202 | 2221 | 4881 | 271 | 6273 |
| Petrimonas mucosa | 50 | 146 | 187 | 5 | 161 |
| Proteiniphilum saccharofermentans | 48 | 128 | 88 | 11 | 90 |
| Nocardioides ungokensis | 3200 | 4158 | 5747 | 78 | 5715 |
| Treponema sp. OMZ 855 | 0 | 117 | 52 | 0 | 17 |
| Treponema sp. OMZ 857 | 0 | 9 | 24 | 0 | 12 |
| Maribacter sp. 1_2014MBL_MicDiv | 1 | 24 | 12 | 0 | 11 |
| Janthinobacterium sp. 1_2014MBL_MicDiv | 814 | 1155 | 1115 | 13 | 8935 |
| Kosakonia pseudosacchari | 0 | 0 | 0 | 0 | 0 |
| Rouxiella badensis | 138 | 172 | 146 | 8 | 247 |
| Acinetobacter sp. TTH0-4 | 0 | 0 | 0 | 0 | 0 |
| Anabaena sp. WA102 | 331 | 1087 | 350 | 0 | 515 |
| Sphingomonas sp. MEA3-1 | 0 | 0 | 0 | 0 | 0 |
| Exiguobacterium sp. JLM-2 | 0 | 0 | 0 | 0 | 1 |
| Streptomyces yangpuensis | 1109 | 1296 | 2863 | 110 | 4122 |
| Aurantiacibacter atlanticus | 293 | 461 | 309 | 5 | 556 |
| Streptomyces sp. CFMR 7 | 1306 | 937 | 1982 | 157 | 3991 |
| Pseudomonas sp. CCOS 191 | 150 | 221 | 235 | 15 | 305 |
| Edwardsiella sp. LADL05-105 | 0 | 90 | 0 | 0 | 0 |
| Brachybacterium huguangmaarense | 2035 | 2093 | 3148 | 95 | 3627 |
| Sellimonas intestinalis | 110 | 97 | 123 | 0 | 72 |
| Caldimicrobium thiodismutans | 16 | 31 | 48 | 0 | 83 |
| Rhodococcus sp. PBTS 1 | 1676 | 1711 | 2494 | 88 | 2688 |
| Alloactinosynnema sp. L-07 | 23849 | 4373 | 23620 | 451 | 15112 |
| Borrelia venezuelensis | 0 | 0 | 0 | 0 | 14 |
| Staphylococcus schweitzeri | 1 | 4 | 0 | 0 | 5 |
| Campylobacter vulpis | 46 | 10 | 5 | 0 | 20 |
| Coxiella endosymbiont of Dermacentor marginatus | 16 | 32 | 28 | 0 | 33 |
| Lentisphaera profundi | 28 | 100 | 86 | 2 | 102 |
| Mitsuaria sp. 7 | 832 | 1808 | 1205 | 0 | 835 |
| Arsenicicoccus sp. oral taxon 190 | 2026 | 2308 | 3757 | 150 | 4843 |
| Pseudomonas sp. GR 6-02 | 303 | 232 | 150 | 0 | 391 |
| Campylobacter sp. RM16187 | 1 | 12 | 15 | 0 | 17 |
| Campylobacter sp. RM6137 | 0 | 0 | 0 | 0 | 0 |
| Campylobacter sp. RM8964 | 3 | 2 | 0 | 0 | 0 |
| Campylobacter sp. RM12175 | 0 | 0 | 5 | 0 | 12 |
| Campylobacter sp. RM5004 | 1 | 1 | 0 | 0 | 10 |
| Campylobacter sp. RM12651 | 2 | 3 | 1 | 0 | 3 |
| Campylobacter sp. RM16192 | 1 | 4 | 5 | 0 | 11 |
| Leptospirillum sp. Group II 'CF-1' | 215 | 233 | 181 | 0 | 290 |
| Streptomyces sp. Tue 6075 | 1123 | 785 | 1406 | 93 | 3450 |
| Methylophilus sp. TWE2 | 54 | 103 | 85 | 1 | 136 |
| Magnetospirillum sp. XM-1 | 1508 | 1674 | 1697 | 40 | 2784 |
| Streptomyces sp. R527F | 713 | 1506 | 1723 | 61 | 2697 |
| Bacillus glycinifermentans | 108 | 203 | 194 | 1 | 638 |
| Halomonas sp. HL-93 | 0 | 0 | 0 | 0 | 0 |
| Dermabacter jinjuensis | 0 | 0 | 0 | 0 | 0 |
| Aquibium oceanicum | 3415 | 3014 | 3141 | 51 | 4121 |
| Egicoccus halophilus | 2372 | 2883 | 4238 | 165 | 4531 |
| Egibacter rhizosphaerae | 2281 | 2972 | 4431 | 93 | 4686 |
| Rathayibacter tanaceti | 803 | 1053 | 1281 | 0 | 1287 |
| Marinobacter sp. CP1 | 0 | 0 | 0 | 0 | 0 |
| Borreliella mayonii | 2 | 0 | 3 | 0 | 6 |
| Megasphaera hexanoica | 63 | 204 | 90 | 1 | 175 |
| Sulfurifustis variabilis | 3167 | 5241 | 4795 | 50 | 6363 |
| Acetivibrio saccincola | 61 | 42 | 83 | 0 | 74 |
| Massilia sp. NR 4-1 | 732 | 1140 | 1290 | 0 | 3583 |
| Limnohabitans sp. 63ED37-2 | 250 | 1735 | 385 | 19 | 688 |
| Planktothrix tepida | 4 | 98 | 30 | 0 | 55 |
| Flavobacterium kingsejongi | 16 | 87 | 57 | 0 | 76 |
| Akkermansia glycaniphila | 254 | 577 | 441 | 4 | 698 |
| Caulobacter flavus | 1236 | 2503 | 1720 | 0 | 1524 |
| Herbinix luporum | 18 | 36 | 30 | 2 | 45 |
| Mycobacterium sp. YC-RL4 | 2227 | 5739 | 4366 | 115 | 8926 |
| Ferrigenium kumadai | 653 | 1160 | 1146 | 5 | 1910 |
| Chryseobacterium glaciei | 12 | 124 | 55 | 0 | 84 |
| Citrobacter sp. BIDMC107 | 32 | 0 | 0 | 0 | 0 |
| Enterobacter sp. BIDMC100 | 0 | 181 | 215 | 0 | 368 |
| Pseudonocardia sp. EC080610-09 | 875 | 427 | 2241 | 0 | 794 |
| Vitreoscilla massiliensis | 65 | 97 | 79 | 6 | 90 |
| Acidithiobacillus ferriphilus | 98 | 412 | 384 | 13 | 470 |
| Phycicoccus endophyticus | 1545 | 2116 | 3195 | 88 | 3740 |
| Streptomyces spongiicola | 1453 | 1610 | 2573 | 169 | 4605 |
| Pseudonocardia sp. HH130630-07 | 4859 | 2881 | 7462 | 205 | 7168 |
| Paenibacillus sp. 32O-W | 355 | 750 | 752 | 5 | 899 |
| Microbacterium sp. CGR1 | 0 | 0 | 0 | 0 | 0 |
| Bordetella sp. H567 | 1232 | 1596 | 1519 | 42 | 2556 |
| Psychrobacter sp. P11F6 | 0 | 0 | 0 | 0 | 0 |
| Psychrobacter sp. P2G3 | 64 | 19 | 20 | 0 | 8 |
| Psychrobacter sp. P11G3 | 0 | 0 | 0 | 0 | 0 |
| Psychrobacter sp. P11G5 | 34 | 14 | 29 | 3 | 70 |
| Aureimonas populi | 2010 | 2004 | 1869 | 89 | 2649 |
| Citrobacter sp. FDAARGOS_156 | 0 | 46 | 13 | 0 | 0 |
| Faecalibaculum rodentium | 121 | 138 | 139 | 1 | 286 |
| Clostridium sp. MF28 | 6 | 27 | 29 | 0 | 6 |
| Pseudomonas sp. IB20 | 24 | 175 | 53 | 0 | 158 |
| Chelatococcus sp. CO-6 | 4503 | 3932 | 3944 | 126 | 5463 |
| Citrobacter sp. CRE-46 | 0 | 0 | 16 | 0 | 302 |
| Croceicoccus sp. Ery5 | 423 | 704 | 551 | 0 | 682 |
| Rhizobium sp. N113 | 0 | 1176 | 2544 | 0 | 662 |
| Rhizobium sp. N1314 | 0 | 8987 | 0 | 0 | 0 |
| Rhizobium sp. N741 | 0 | 0 | 0 | 0 | 38345 |
| Rhizobium sp. N324 | 1371 | 432 | 288 | 0 | 381 |
| Rhizobium sp. N541 | 0 | 0 | 1723 | 0 | 1093 |
| Arthrobacter sp. ERGS1:01 | 675 | 1057 | 1522 | 44 | 2318 |
| Candidatus Xiphinematobacter sp. Idaho Grape | 285 | 794 | 516 | 5 | 658 |
| Methylovulum psychrotolerans | 138 | 473 | 269 | 6 | 374 |
| Burkholderia sp. IDO3 | 603 | 248 | 669 | 2 | 592 |
| Candidatus Thioglobus autotrophicus | 4 | 38 | 16 | 4 | 23 |
| Hymenobacter monticola | 346 | 930 | 868 | 24 | 1502 |
| Bacillus sp. FJAT-18017 | 68 | 95 | 115 | 0 | 115 |
| Capnocytophaga sp. oral taxon 323 | 23 | 26 | 21 | 0 | 39 |
| Massilia sp. WG5 | 1261 | 1972 | 1947 | 0 | 1506 |
| Pseudoalteromonas sp. R3 | 16 | 89 | 107 | 3 | 118 |
| Pyruvatibacter mobilis | 1258 | 1093 | 987 | 0 | 1021 |
| Thermanaerosceptrum fracticalcis | 33 | 202 | 167 | 1 | 150 |
| Paenibacillus baekrokdamisoli | 39 | 236 | 146 | 1 | 244 |
| Turicibacter sp. H121 | 0 | 0 | 32 | 0 | 0 |
| Microbacterium sp. No. 7 | 2485 | 2565 | 4002 | 137 | 4238 |
| Alteromonas sp. BL110 | 1 | 36 | 32 | 7 | 208 |
| Aquimarina sp. AD1 | 27 | 22 | 19 | 7 | 30 |
| Aquimarina sp. AD10 | 0 | 0 | 0 | 0 | 0 |
| Aquimarina sp. BL5 | 4 | 24 | 16 | 0 | 17 |
| Diaphorobacter ruginosibacter | 794 | 1116 | 1033 | 8 | 1261 |
| Staphylococcus sp. AntiMn-1 | 21 | 0 | 0 | 0 | 0 |
| Candidatus Nitrospira inopinata | 1641 | 3103 | 3336 | 30 | 3464 |
| Pseudodesulfovibrio indicus | 571 | 1132 | 1113 | 50 | 1517 |
| Clostridium thermarum | 34 | 38 | 21 | 0 | 34 |
| Labilibaculum antarcticum | 34 | 53 | 45 | 2 | 65 |
| Mycobacterium vicinigordonae | 1418 | 2645 | 2006 | 93 | 3990 |
| Acetilactobacillus jinshanensis | 0 | 0 | 0 | 0 | 0 |
| Pseudoalteromonas sp. 1_2015MBL_MicDiv | 0 | 8 | 2 | 0 | 7 |
| Psychrobacter sp. AntiMn-1 | 116 | 126 | 23 | 0 | 9 |
| Chryseobacterium sp. IHB B 17019 | 15 | 42 | 38 | 0 | 37 |
| Rhodococcus sp. 008 | 173 | 1352 | 589 | 0 | 771 |
| Candidatus Desulfovibrio trichonymphae | 101 | 219 | 125 | 4 | 168 |
| Streptomyces sp. CdTB01 | 2706 | 2226 | 3934 | 144 | 5525 |
| Anaerocolumna chitinilytica | 5 | 21 | 20 | 0 | 32 |
| Algoriphagus sanaruensis | 35 | 127 | 69 | 0 | 62 |
| Pedobacter sp. PACM 27299 | 38 | 149 | 138 | 1 | 409 |
| Methylomonas sp. DH-1 | 246 | 471 | 553 | 11 | 651 |
| Rhodococcus sp. H-CA8f | 0 | 0 | 0 | 0 | 0 |
| Gulosibacter sediminis | 873 | 980 | 1356 | 67 | 1272 |
| Salegentibacter sp. T436 | 22 | 78 | 98 | 0 | 65 |
| Bdellovibrio sp. SKB1291214 | 26 | 74 | 59 | 0 | 72 |
| Clavibacter phaseoli | 438 | 745 | 1622 | 2 | 909 |
| Serpentinicella alkaliphila | 4 | 17 | 6 | 0 | 14 |
| Oryzomicrobium terrae | 864 | 1178 | 1271 | 25 | 2040 |
| Streptomyces sp. SM18 | 1707 | 1401 | 2797 | 309 | 7788 |
| Pseudomonas sp. Leaf58 | 0 | 0 | 0 | 0 | 0 |
| Aeromicrobium sp. Leaf245 | 10744 | 18785 | 14343 | 92 | 6699 |
| Algibacter alginicilyticus | 0 | 0 | 0 | 0 | 0 |
| Devosia sp. A16 | 5503 | 4328 | 2928 | 62 | 3875 |
| Aeromicrobium choanae | 5120 | 8410 | 7211 | 67 | 4093 |
| Gordonia sp. 1D | 428 | 614 | 1364 | 129 | 2353 |
| Corynebacterium provencense | 435 | 480 | 748 | 39 | 1182 |
| Chitinibacter fontanus | 140 | 162 | 145 | 2 | 265 |
| Agarilytica rhodophyticola | 55 | 55 | 57 | 3 | 184 |
| Shewanella inventionis | 19 | 56 | 54 | 25 | 103 |
| Burkholderia sp. Bp5365 | 1802 | 0 | 0 | 0 | 0 |
| Burkholderia sp. Bp7605 | 916 | 1165 | 1156 | 23 | 1789 |
| Catenovulum sediminis | 9 | 39 | 26 | 9 | 64 |
| Bacillus dafuensis | 0 | 0 | 0 | 0 | 0 |
| Polynucleobacter antarcticus | 0 | 0 | 0 | 0 | 0 |
| Polynucleobacter arcticus | 11 | 41 | 57 | 0 | 56 |
| Polynucleobacter wuianus | 22 | 61 | 44 | 0 | 48 |
| Polynucleobacter sp. MWH-P3-07-1 | 33 | 58 | 42 | 0 | 82 |
| Polynucleobacter tropicus | 0 | 26 | 27 | 0 | 36 |
| Ancylobacter pratisalsi | 2695 | 1799 | 1939 | 66 | 2625 |
| Bordetella sp. N | 1481 | 1534 | 1824 | 33 | 2584 |
| Aeromonas sp. ARM81 | 0 | 0 | 0 | 0 | 0 |
| Spongiibacter taiwanensis | 0 | 0 | 0 | 0 | 0 |
| Citrobacter cronae | 0 | 168 | 119 | 17 | 186 |
| Mycoplasma sp. (ex Biomphalaria glabrata) | 26 | 25 | 42 | 0 | 28 |
| Marinobacter sp. LQ44 | 184 | 412 | 239 | 4 | 301 |
| Roseomonas sp. S08 | 2651 | 2276 | 2387 | 24 | 3198 |
| Fructilactobacillus ixorae | 0 | 48 | 26 | 0 | 27 |
| Dissulfurimicrobium hydrothermale | 0 | 0 | 0 | 0 | 0 |
| Kurthia sp. 11kri321 | 0 | 0 | 0 | 0 | 0 |
| Fluviibacter phosphoraccumulans | 0 | 0 | 0 | 0 | 0 |
| Flavobacterium ammonificans | 17 | 76 | 46 | 0 | 30 |
| Flavobacterium ammoniigenes | 0 | 0 | 0 | 0 | 0 |
| Nostoc sp. NIES-3756 | 19 | 742 | 76 | 0 | 149 |
| Fischerella sp. NIES-3754 | 149 | 414 | 249 | 0 | 650 |
| Leptolyngbya sp. NIES-3755 | 57 | 269 | 151 | 4 | 183 |
| Ensifer alkalisoli | 1675 | 1584 | 1409 | 43 | 1991 |
| Fusobacterium gastrosuis | 1 | 3 | 12 | 0 | 11 |
| Pseudomonas sp. DY-1 | 439 | 659 | 636 | 26 | 969 |
| Paraphotobacterium marinum | 4 | 9 | 14 | 0 | 3 |
| Elizabethkingia bruuniana | 0 | 0 | 0 | 0 | 0 |
| Elizabethkingia ursingii | 0 | 0 | 0 | 0 | 0 |
| Nitratireductor sp. OM-1 | 1383 | 1187 | 972 | 37 | 1446 |
| Celeribacter ethanolicus | 0 | 0 | 0 | 0 | 0 |
| Aeromonas sp. ASNIH5 | 462 | 378 | 299 | 10 | 327 |
| Acinetobacter sp. ACNIH2 | 3 | 18 | 27 | 0 | 31 |
| Achromobacter sp. AONIH1 | 2625 | 3011 | 4360 | 24 | 5546 |
| Serratia sp. SSNIH1 | 108 | 48 | 86 | 0 | 76 |
| Polynucleobacter sp. TSB-Sco08W16 | 41 | 164 | 47 | 1 | 57 |
| Polynucleobacter sp. JS-JIR-II-b4 | 35 | 22 | 15 | 0 | 22 |
| Polynucleobacter sp. JS-JIR-5-A7 | 12 | 24 | 21 | 0 | 75 |
| Polynucleobacter sp. VK25 | 31 | 26 | 38 | 0 | 141 |
| Serinicoccus hydrothermalis | 1387 | 1435 | 2302 | 19 | 2616 |
| Pseudomonas sp. BIOMIG1BAC | 0 | 0 | 0 | 0 | 0 |
| Candidatus Viadribacter manganicus | 815 | 1069 | 775 | 11 | 1310 |
| Streptococcus sp. A12 | 0 | 0 | 0 | 0 | 0 |
| Methylomagnum ishizawai | 690 | 1412 | 1070 | 32 | 1415 |
| Candidatus Formimonas warabiya | 0 | 0 | 0 | 0 | 0 |
| Paraburkholderia caffeinilytica | 974 | 1057 | 1159 | 3 | 1381 |
| Euzebyella marina | 68 | 57 | 74 | 0 | 50 |
| Halomonas sp. hl-4 | 552 | 367 | 726 | 32 | 158 |
| Marinobacter sp. es.042 | 0 | 0 | 0 | 0 | 0 |
| Pseudoalteromonas sp. 13-15 | 0 | 0 | 2 | 0 | 5 |
| Pseudomonas sp. bs2935 | 0 | 317 | 373 | 0 | 1702 |
| cyanobacterium endosymbiont of Rhopalodia gibberula | 0 | 0 | 0 | 0 | 0 |
| Flavobacterium crassostreae | 6 | 64 | 44 | 1 | 45 |
| Thalassotalea crassostreae | 42 | 17 | 20 | 0 | 19 |
| Paenibacillus crassostreae | 50 | 88 | 94 | 0 | 123 |
| Hankyongella ginsenosidimutans | 1427 | 1397 | 1313 | 10 | 1870 |
| Vibrio cidicii | 0 | 0 | 0 | 0 | 0 |
| Azospira sp. I09 | 0 | 0 | 0 | 0 | 0 |
| Bifidobacterium eulemuris | 191 | 204 | 455 | 4 | 663 |
| Acidihalobacter ferrooxydans | 896 | 1192 | 1131 | 24 | 1297 |
| Deinococcus actinosclerus | 864 | 1720 | 1701 | 39 | 2220 |
| Microbulbifer aggregans | 273 | 405 | 492 | 2 | 525 |
| Salinicola tamaricis | 909 | 965 | 1086 | 13 | 1362 |
| Nocardioides rotundus | 2355 | 3688 | 4630 | 361 | 6123 |
| Polaribacter vadi | 0 | 0 | 0 | 0 | 0 |
| Glutamicibacter mishrai | 262 | 249 | 325 | 7 | 592 |
| Sulfitobacter faviae | 888 | 1110 | 835 | 27 | 1197 |
| Methylobacterium indicum | 2096 | 1954 | 2167 | 24 | 3070 |
| Emergencia timonensis | 100 | 188 | 117 | 2 | 154 |
| Acinetobacter vivianii | 24 | 52 | 17 | 0 | 19 |
| Alteromonas sp. Mac1 | 0 | 20 | 0 | 0 | 0 |
| Mesorhizobium erdmanii | 1828 | 1349 | 1292 | 4 | 1595 |
| Mesorhizobium jarvisii | 0 | 7561 | 0 | 0 | 20293 |
| Candidatus Doolittlea endobia | 9 | 20 | 25 | 1 | 48 |
| Candidatus Hoaglandella endobia | 4 | 11 | 12 | 0 | 17 |
| Candidatus Mikella endobia | 5 | 7 | 16 | 0 | 13 |
| Dickeya fangzhongdai | 249 | 901 | 494 | 55 | 657 |
| Chromobacterium rhizoryzae | 453 | 1018 | 1019 | 0 | 1623 |
| Paenibacillus psychroresistens | 240 | 314 | 415 | 0 | 496 |
| Campylobacter geochelonis | 1 | 8 | 6 | 0 | 5 |
| Streptomyces qaidamensis | 1749 | 1750 | 2838 | 204 | 3441 |
| Flavobacterium arcticum | 0 | 0 | 0 | 0 | 0 |
| Arcticibacterium luteifluviistationis | 18 | 24 | 26 | 0 | 43 |
| Leucobacter triazinivorans | 1492 | 1606 | 2259 | 125 | 2423 |
| Acinetobacter lactucae | 0 | 0 | 0 | 0 | 0 |
| Pseudomonas glycinae | 0 | 0 | 0 | 0 | 0 |
| Pseudomonas wadenswilerensis | 387 | 286 | 319 | 9 | 455 |
| Gemella sp. oral taxon 928 | 0 | 0 | 21 | 0 | 0 |
| Leptotrichia sp. oral taxon 847 | 18 | 2 | 3 | 0 | 3 |
| Acinetobacter larvae | 20 | 18 | 24 | 5 | 34 |
| Skermanella mucosa | 2506 | 2935 | 2663 | 29 | 3339 |
| Wenyingzhuangia fucanilytica | 0 | 0 | 0 | 0 | 0 |
| Bosea sp. PAMC 26642 | 3109 | 2896 | 2462 | 35 | 3166 |
| Salipiger sp. CCB-MM3 | 786 | 1101 | 1077 | 13 | 1507 |
| Thermodesulfobium acidiphilum | 5 | 79 | 41 | 3 | 66 |
| Mycobacterium virginiense | 1129 | 2278 | 1688 | 55 | 3407 |
| Burkholderia sp. PAMC 26561 | 457 | 590 | 699 | 0 | 1319 |
| Microbacterium sp. PAMC 28756 | 331 | 350 | 1384 | 0 | 877 |
| Echinicola strongylocentroti | 75 | 152 | 109 | 1 | 162 |
| Frondihabitans sp. PAMC 28766 | 1259 | 1763 | 1937 | 12 | 2018 |
| Variovorax sp. PAMC 28711 | 1182 | 1802 | 1180 | 0 | 1208 |
| Burkholderia sp. PAMC 28687 | 451 | 689 | 524 | 10 | 939 |
| Cupriavidus nantongensis | 2198 | 2074 | 2866 | 6 | 3170 |
| Bacteroides caecimuris | 4 | 34 | 43 | 0 | 508 |
| Blautia pseudococcoides | 10 | 154 | 43 | 3 | 63 |
| Acutalibacter muris | 119 | 243 | 285 | 5 | 319 |
| Longicatena caecimuris | 0 | 0 | 0 | 0 | 0 |
| Muribaculum intestinale | 42 | 109 | 135 | 8 | 148 |
| Turicimonas muris | 0 | 0 | 0 | 0 | 0 |
| Ereboglobus luteus | 870 | 2425 | 1859 | 22 | 2059 |
| Erythrobacter sp. HL-111 | 542 | 913 | 658 | 13 | 996 |
| Cohaesibacter sp. ES.047 | 0 | 0 | 0 | 0 | 0 |
| Leifsonia sp. 509MF | 3193 | 0 | 0 | 0 | 2942 |
| Leifsonia sp. 467MF | 0 | 0 | 0 | 0 | 2841 |
| Leifsonia sp. 21MFCrub1.1 | 1035 | 2607 | 3847 | 118 | 1824 |
| Jiangella sp. DSM 45060 | 6167 | 5355 | 10143 | 371 | 11518 |
| Formosa sp. Hel1_31_208 | 5 | 24 | 18 | 0 | 14 |
| Candidatus Fokinia solitaria | 39 | 24 | 13 | 0 | 27 |
| Flavivirga eckloniae | 6 | 23 | 22 | 2 | 31 |
| Thiomicrospira sp. S5 | 70 | 75 | 130 | 15 | 153 |
| Nocardioides baekrokdamisoli | 1636 | 1825 | 2167 | 18 | 1715 |
| Amycolatopsis albispora | 25855 | 5137 | 21445 | 469 | 15060 |
| Microcella flavibacter | 999 | 2158 | 2081 | 67 | 2325 |
| Enterococcus saigonensis | 0 | 0 | 0 | 0 | 0 |
| Olsenella timonensis | 710 | 953 | 1261 | 27 | 1411 |
| Rhodococcus sp. MTM3W5.2 | 2943 | 2992 | 5439 | 170 | 5561 |
| Rahnella sp. ERMR1:05 | 1976 | 333 | 217 | 2 | 313 |
| Candidatus Promineofilum breve | 1477 | 3667 | 3304 | 35 | 4537 |
| Sphingorhabdus sp. M41 | 0 | 0 | 0 | 0 | 0 |
| Candidatus Chlamydia sanziniae | 22 | 13 | 44 | 0 | 6 |
| Chryseobacterium panacisoli | 6 | 9 | 2 | 0 | 12 |
| Stanieria sp. NIES-3757 | 41 | 77 | 57 | 0 | 39 |
| Echinicola rosea | 105 | 131 | 79 | 0 | 120 |
| Rhodococcus sp. BH4 | 24 | 162 | 243 | 0 | 432 |
| Acinetobacter sp. LoGeW2-3 | 0 | 0 | 0 | 0 | 0 |
| Candidatus Methylospira mobilis | 367 | 687 | 538 | 19 | 841 |
| Herminiimonas arsenitoxidans | 256 | 405 | 327 | 0 | 455 |
| Immundisolibacter cernigliae | 1651 | 2569 | 2372 | 14 | 2882 |
| Streptococcus pantholopis | 4 | 16 | 29 | 0 | 16 |
| Sulfuriflexus mobilis | 196 | 359 | 388 | 4 | 417 |
| Wolbachia endosymbiont of Cruorifilaria tuberocauda | 9 | 5 | 1 | 0 | 2 |
| Wolbachia endosymbiont of Dipetalonema caudispina | 0 | 6 | 3 | 0 | 5 |
| Wolbachia endosymbiont of Dirofilaria (Dirofilaria) immitis | 5 | 6 | 2 | 0 | 14 |
| Streptomyces sp. fd1-xmd | 1758 | 1480 | 2901 | 73 | 4325 |
| Enterobacter roggenkampii | 0 | 0 | 0 | 0 | 0 |
| Campylobacter hepaticus | 0 | 3 | 0 | 0 | 1 |
| Geobacillus sp. JS12 | 0 | 0 | 0 | 0 | 0 |
| Peteryoungia desertarenae | 0 | 0 | 0 | 0 | 0 |
| Sphingomonas naphthae | 1347 | 1908 | 1427 | 0 | 2905 |
| Campylobacter sp. 2014D-0216 | 5 | 2 | 3 | 0 | 1 |
| Chryseobacterium cucumeris | 14 | 42 | 27 | 0 | 9 |
| Panacibacter ginsenosidivorans | 459 | 741 | 612 | 10 | 654 |
| Sphingomonas panacisoli | 0 | 0 | 0 | 0 | 0 |
| Tetrasphaera sp. HKS02 | 1255 | 1227 | 2242 | 43 | 1646 |
| Streptococcus halotolerans | 10 | 12 | 5 | 0 | 13 |
| Nitrosococcus wardiae | 131 | 266 | 219 | 0 | 264 |
| Neisseria musculi | 222 | 165 | 261 | 10 | 469 |
| Colwellia sp. PAMC 20917 | 0 | 0 | 0 | 0 | 0 |
| Abyssicoccus albus | 0 | 0 | 0 | 0 | 0 |
| Skermanella rosea | 2644 | 2621 | 3114 | 29 | 3849 |
| Streptomyces zhihengii | 24 | 21 | 54 | 15 | 379 |
| Polynucleobacter ibericus | 39 | 31 | 19 | 2 | 44 |
| Polynucleobacter sp. UB-Raua-W9 | 0 | 12 | 0 | 0 | 0 |
| Marinobacterium aestuarii | 0 | 0 | 0 | 0 | 0 |
| Streptomyces sp. VN1 | 927 | 1129 | 2653 | 6 | 2001 |
| Edwardsiella anguillarum | 0 | 86 | 0 | 0 | 138 |
| Desulfuromonas sp. DDH964 | 900 | 1708 | 1813 | 27 | 2300 |
| Vibrio japonicus | 7 | 57 | 33 | 11 | 35 |
| Streptococcus marmotae | 0 | 16 | 14 | 0 | 12 |
| Neorhizobium sp. NCHU2750 | 888 | 925 | 752 | 42 | 982 |
| Silicimonas algicola | 966 | 1214 | 1148 | 24 | 1618 |
| Synechococcus sp. NIES-970 | 34 | 109 | 128 | 0 | 165 |
| Acinetobacter sp. MYb10 | 0 | 0 | 0 | 0 | 0 |
| Pseudomonas sp. MYb193 | 69 | 112 | 118 | 3 | 305 |
| Stenotrophomonas sp. MYb57 | 180 | 243 | 250 | 0 | 296 |
| Enterobacter sp. ODB01 | 29 | 26 | 33 | 0 | 0 |
| Streptomyces nigra | 1606 | 1557 | 2818 | 75 | 3611 |
| Desulforamulus ferrireducens | 154 | 182 | 100 | 2 | 116 |
| Lachnoclostridium sp. YL32 | 14 | 38 | 37 | 6 | 75 |
| Fibrella sp. ES10-3-2-2 | 231 | 523 | 385 | 9 | 468 |
| Polynucleobacter duraquae | 15 | 30 | 16 | 6 | 23 |
| secondary endosymbiont of Trabutina mannipara | 0 | 0 | 0 | 0 | 0 |
| Pantoea sp. OXWO6B1 | 2 | 4 | 3 | 0 | 4 |
| Maribacter hydrothermalis | 33 | 15 | 40 | 1 | 28 |
| Bacillus sp. FJAT-14266 | 0 | 0 | 0 | 0 | 850 |
| Streptomyces sp. S8 | 1171 | 929 | 1437 | 151 | 3026 |
| Lacunisphaera limnophila | 1674 | 4208 | 4323 | 27 | 4012 |
| Acinetobacter portensis | 21 | 26 | 6 | 0 | 9 |
| Bacillus sp. FDAARGOS_235 | 12 | 0 | 0 | 0 | 0 |
| Dehalogenimonas formicexedens | 213 | 518 | 596 | 4 | 730 |
| Polaromonas sp. E10S | 0 | 7 | 11 | 0 | 3 |
| Polaromonas sp. E19S | 0 | 0 | 26 | 0 | 0 |
| Polaromonas sp. E3S | 366 | 215 | 527 | 0 | 187 |
| Polaromonas sp. H1N | 288 | 6 | 14 | 0 | 11 |
| Polaromonas sp. H6N | 0 | 0 | 0 | 0 | 0 |
| Polaromonas sp. H8N | 38 | 9 | 6 | 0 | 95 |
| Polaromonas sp. W11N | 0 | 3 | 1 | 0 | 20 |
| Streptomyces sp. RTd22 | 2872 | 1772 | 4040 | 155 | 5043 |
| Agrobacterium sp. 13-2099-1-2 | 102 | 84 | 56 | 0 | 109 |
| Syntrophotalea acetylenivorans | 271 | 335 | 407 | 7 | 620 |
| Acidovorax sp. RAC01 | 891 | 1273 | 1280 | 0 | 1871 |
| Blastomonas sp. RAC04 | 0 | 0 | 0 | 0 | 0 |
| Agrobacterium sp. RAC06 | 306 | 358 | 422 | 5 | 687 |
| Hydrogenophaga sp. RAC07 | 284 | 895 | 686 | 0 | 600 |
| Bosea sp. RAC05 | 4092 | 3438 | 2999 | 0 | 3504 |
| Methyloversatilis sp. RAC08 | 975 | 1403 | 1199 | 0 | 2300 |
| Rhodoferax koreense | 3353 | 5239 | 5509 | 51 | 7750 |
| Phaeobacter porticola | 0 | 0 | 0 | 0 | 0 |
| Brucella sp. 09RB8910 | 0 | 0 | 0 | 0 | 0 |
| Listeria sp. PSOL-1 | 1 | 16 | 11 | 0 | 22 |
| Candidatus Wolbachia massiliensis | 1 | 6 | 6 | 0 | 6 |
| Companilactobacillus allii | 1 | 9 | 10 | 0 | 7 |
| Enterobacter sp. LU1 | 0 | 15 | 20 | 0 | 0 |
| Hafnia sp. CBA7124 | 0 | 25 | 49 | 0 | 34 |
| Campylobacter ornithocola | 0 | 6 | 0 | 0 | 0 |
| Streptomyces sp. ST1015 | 3667 | 2345 | 4639 | 309 | 6440 |
| Capnocytophaga stomatis | 33 | 28 | 16 | 0 | 22 |
| Arcobacter acticola | 0 | 0 | 0 | 0 | 0 |
| Exiguobacterium sp. U13-1 | 0 | 66 | 26 | 0 | 49 |
| Arthrobacter sp. U41 | 889 | 1952 | 2702 | 30 | 4193 |
| Dyella caseinilytica | 722 | 729 | 829 | 6 | 994 |
| Streptomyces sp. SAT1 | 2300 | 1847 | 3504 | 304 | 7964 |
| Hymenobacter nivis | 250 | 692 | 645 | 1 | 797 |
| Rhizorhabdus dicambivorans | 4524 | 4295 | 3379 | 23 | 7371 |
| Urechidicola croceus | 38 | 23 | 23 | 0 | 41 |
| Rhodobacter sp. LPB0142 | 1428 | 1973 | 2017 | 7 | 2288 |
| Tenacibaculum todarodis | 0 | 27 | 17 | 3 | 12 |
| Poseidonibacter parvus | 7 | 0 | 30 | 0 | 14 |
| Ancylobacter sp. TS-1 | 3593 | 2305 | 2427 | 55 | 3486 |
| Arachidicoccus sp. BS20 | 0 | 0 | 0 | 0 | 0 |
| Limihaloglobus sulfuriphilus | 84 | 266 | 321 | 1 | 394 |
| Streptomyces sp. 11-1-2 | 1440 | 1151 | 3174 | 117 | 4310 |
| Actinomyces sp. Chiba101 | 444 | 499 | 844 | 20 | 910 |
| Cyanobium sp. NIES-981 | 531 | 747 | 849 | 25 | 1043 |
| Orrella dioscoreae | 1357 | 1775 | 1917 | 38 | 2625 |
| Roseitalea porphyridii | 1762 | 1970 | 1839 | 27 | 2415 |
| Murdochiella vaginalis | 75 | 223 | 203 | 1 | 334 |
| Ezakiella massiliensis | 0 | 0 | 0 | 0 | 0 |
| Pseudomonas silesiensis | 379 | 525 | 581 | 25 | 1128 |
| Neisseria weixii | 37 | 86 | 124 | 1 | 169 |
| Neisseria chenwenguii | 172 | 316 | 223 | 9 | 701 |
| Mesorhizobium sp. AA22 | 1764 | 1569 | 1432 | 50 | 2166 |
| Sulfurospirillum diekertiae | 21 | 57 | 24 | 1 | 40 |
| Chryseobacterium nepalense | 0 | 0 | 0 | 0 | 0 |
| Streptomyces huasconensis | 673 | 0 | 0 | 0 | 0 |
| Pseudomonas sp. A214 | 167 | 98 | 144 | 0 | 211 |
| Polaribacter sp. KT25b | 17 | 12 | 8 | 0 | 9 |
| Candidatus Aquiluna sp. UB-MaderosW2red | 60 | 107 | 135 | 5 | 391 |
| Pseudomonas sp. Z003-0.4C(8344-21) | 128 | 189 | 155 | 7 | 286 |
| Sphingobium sp. EP60837 | 773 | 911 | 741 | 0 | 1351 |
| Polynucleobacter sp. MWH-UH25E | 11 | 52 | 30 | 0 | 59 |
| Polynucleobacter sp. MWH-UH2A | 4 | 28 | 16 | 0 | 26 |
| Polynucleobacter sp. MWH-UH35A | 37 | 36 | 35 | 6 | 80 |
| Polynucleobacter sp. UB-Siik-W21 | 3 | 19 | 16 | 0 | 26 |
| Polynucleobacter sp. es-EL-1 | 3 | 30 | 30 | 6 | 62 |
| Macrococcus canis | 26 | 127 | 28 | 0 | 11 |
| Polynucleobacter sp. MWH-Aus1W21 | 16 | 126 | 14 | 2 | 44 |
| Polynucleobacter sp. MWH-Braz-FAM2G | 12 | 36 | 50 | 0 | 52 |
| Polynucleobacter paludilacus | 2 | 26 | 27 | 0 | 28 |
| Polynucleobacter sp. MWH-Svant-W18 | 36 | 41 | 43 | 1 | 58 |
| Polynucleobacter sp. MWH-S4W17 | 4 | 39 | 39 | 0 | 33 |
| Luteitalea pratensis | 23675 | 58767 | 39280 | 226 | 42026 |
| Pseudomonas sp. TCU-HL1 | 413 | 668 | 632 | 29 | 1342 |
| Acidovorax sp. T1 | 1812 | 3587 | 3934 | 22 | 5299 |
| Alteromonas pelagimontana | 130 | 78 | 68 | 5 | 73 |
| Floricoccus penangensis | 0 | 0 | 0 | 0 | 0 |
| Halothiobacillus sp. LS2 | 0 | 0 | 0 | 0 | 0 |
| Nisaea acidiphila | 2121 | 1894 | 2128 | 16 | 3306 |
| Corynebacterium choanae | 23 | 102 | 181 | 7 | 299 |
| Sphingopyxis sp. MG | 243 | 863 | 636 | 27 | 700 |
| Hyphococcus flavus | 460 | 403 | 387 | 27 | 505 |
| Synechococcus sp. A18-25c | 51 | 109 | 115 | 0 | 161 |
| Bosea sp. Tri-49 | 4426 | 4259 | 4235 | 70 | 4898 |
| Phyllobacterium zundukense | 34120 | 17268 | 7937 | 77 | 15142 |
| Legionella clemsonensis | 57 | 55 | 41 | 2 | 55 |
| Enterobacter sp. HK169 | 0 | 57 | 83 | 0 | 96 |
| Prosthecochloris sp. CIB 2401 | 68 | 209 | 217 | 3 | 501 |
| Phreatobacter cathodiphilus | 6567 | 4035 | 4402 | 82 | 5292 |
| Jeotgalibaca porci | 0 | 0 | 0 | 0 | 0 |
| Jeotgalibaca arthritidis | 0 | 0 | 0 | 0 | 0 |
| Rhizobium sp. S41 | 0 | 743 | 1656 | 0 | 309 |
| Nostoc sp. CENA543 | 0 | 0 | 0 | 0 | 0 |
| Polaromonas sp. | 9 | 40 | 42 | 0 | 59 |
| Paenibacillus sp. BIHB4019 | 56 | 383 | 230 | 1 | 603 |
| Paenibacillus ihbetae | 138 | 353 | 376 | 1 | 377 |
| Arabiibacter massiliensis | 478 | 861 | 935 | 30 | 1417 |
| Parolsenella massiliensis | 273 | 816 | 872 | 2 | 860 |
| Ndongobacter massiliensis | 102 | 149 | 155 | 3 | 390 |
| Chryseobacterium sp. | 41 | 96 | 107 | 1 | 126 |
| Janthinobacterium sp. | 0 | 0 | 0 | 0 | 2 |
| Flavobacterium azooxidireducens | 18 | 105 | 48 | 0 | 64 |
| Acinetobacter defluvii | 0 | 0 | 0 | 0 | 0 |
| Salinicoccus sp. | 4 | 0 | 0 | 0 | 0 |
| Paenibacillus tritici | 133 | 277 | 220 | 25 | 381 |
| Hyphomonas sp. CACIAM 19H1 | 0 | 0 | 0 | 0 | 0 |
| Sphingopyxis sp. EG6 | 807 | 1278 | 928 | 0 | 744 |
| Marinobacter salinus | 0 | 0 | 0 | 0 | 0 |
| Gluconobacter sp. | 0 | 0 | 0 | 0 | 1 |
| Candidatus Fukatsuia symbiotica | 0 | 0 | 0 | 0 | 0 |
| Acinetobacter sp. WCHAc010034 | 195 | 392 | 342 | 0 | 353 |
| Acinetobacter wuhouensis | 0 | 0 | 0 | 0 | 0 |
| Pseudomonas sivasensis | 0 | 506 | 0 | 0 | 12949 |
| Achromobacter sp. MFA1 R4 | 792 | 1099 | 1283 | 0 | 1468 |
| Pseudomonas sp. 7SR1 | 371 | 593 | 574 | 17 | 848 |
| Streptomyces sp. 2114.2 | 477 | 587 | 824 | 0 | 2287 |
| Stappia sp. ES.058 | 1817 | 1446 | 1162 | 13 | 1386 |
| Borrelia sp. HM | 1 | 10 | 11 | 0 | 13 |
| Microvirga ossetica | 4965 | 4925 | 3899 | 70 | 5237 |
| Afipia sp. GAS231 | 14449 | 10620 | 7310 | 31 | 6046 |
| Opitutus sp. GAS368 | 2181 | 5331 | 6534 | 39 | 6262 |
| Streptomyces sp. 3214.6 | 2465 | 1875 | 3358 | 267 | 5519 |
| Verrucomicrobium sp. GAS474 | 1561 | 3752 | 3274 | 24 | 3844 |
| Pajaroellobacter abortibovis | 67 | 141 | 161 | 2 | 157 |
| Pistricoccus aurantiacus | 0 | 0 | 0 | 0 | 0 |
| Halomonas sp. 1513 | 477 | 692 | 789 | 13 | 1042 |
| Selenomonas sp. oral taxon 920 | 212 | 418 | 344 | 3 | 567 |
| Candidatus Nanopelagicus limnes | 8 | 21 | 11 | 2 | 49 |
| Candidatus Planktophila sulfonica | 0 | 0 | 0 | 0 | 0 |
| Candidatus Planktophila versatilis | 0 | 0 | 0 | 0 | 0 |
| Candidatus Planktophila vernalis | 17 | 39 | 33 | 3 | 53 |
| Candidatus Planktophila lacus | 104 | 291 | 120 | 4 | 233 |
| Candidatus Planktophila dulcis | 20 | 66 | 118 | 0 | 165 |
| Candidatus Nanopelagicus hibericus | 53 | 24 | 54 | 2 | 77 |
| Candidatus Nanopelagicus abundans | 20 | 24 | 5 | 0 | 28 |
| Pseudomonas sp. TMW 2.1634 | 0 | 0 | 0 | 0 | 0 |
| Streptococcus himalayensis | 17 | 11 | 4 | 0 | 2 |
| Cellulophaga omnivescoria | 9 | 12 | 13 | 0 | 20 |
| Polaribacter haliotis | 1 | 13 | 12 | 0 | 12 |
| Deinococcus rubellus | 344 | 753 | 747 | 7 | 962 |
| Bacillus wiedmannii | 41 | 93 | 35 | 0 | 22 |
| Pandoraea fibrosis | 696 | 943 | 1033 | 7 | 1402 |
| Vibrio aphrogenes | 34 | 69 | 89 | 6 | 67 |
| Thaumasiovibrio subtropicus | 9 | 68 | 55 | 1 | 89 |
| Thalassospira indica | 508 | 390 | 288 | 8 | 526 |
| Epidermidibacterium keratini | 2349 | 3050 | 5883 | 297 | 6525 |
| Pantoea alhagi | 86 | 171 | 197 | 0 | 231 |
| Roseobacter ponti | 451 | 510 | 360 | 4 | 507 |
| Fuerstiella marisgermanici | 656 | 2775 | 1698 | 21 | 2708 |
| Bacillus sp. ABP14 | 0 | 6 | 27 | 0 | 14 |
| Sphingosinicella sp. BN140058 | 1679 | 2600 | 2106 | 54 | 9073 |
| Luteimonas sp. JM171 | 1312 | 1123 | 1562 | 21 | 2298 |
| Polaribacter sp. KT 15 | 26 | 25 | 5 | 0 | 7 |
| Porphyrobacter sp. LM 6 | 0 | 0 | 0 | 0 | 0 |
| Amycolatopsis sp. AA4 | 9147 | 2724 | 8655 | 279 | 7546 |
| Cryobacterium sp. SO1 | 490 | 880 | 1277 | 44 | 1624 |
| Halomonas aestuarii | 680 | 991 | 902 | 8 | 1414 |
| Macrococcus sp. IME1552 | 0 | 8 | 0 | 0 | 0 |
| Pseudomonas sp. LPH1 | 104 | 139 | 207 | 24 | 307 |
| Streptococcus sp. NPS 308 | 0 | 0 | 0 | 0 | 0 |
| Nakamurella antarctica | 439 | 429 | 844 | 34 | 692 |
| Macrococcus epidermidis | 12 | 4 | 6 | 0 | 4 |
| Macrococcus bohemicus | 0 | 4 | 0 | 0 | 4 |
| Brachybacterium sp. P6-10-X1 | 816 | 793 | 1356 | 52 | 1087 |
| Jeotgalibaca sp. PTS2502 | 0 | 0 | 0 | 0 | 0 |
| Oceanisphaera avium | 12 | 48 | 43 | 10 | 46 |
| Tumebacillus avium | 443 | 1807 | 1734 | 66 | 1614 |
| Streptomyces fodineus | 2200 | 1618 | 2805 | 153 | 4415 |
| Stenotrophomonas sp. LM091 | 521 | 1031 | 991 | 0 | 1314 |
| Klebsiella sp. LTGPAF-6F | 0 | 42 | 157 | 0 | 68 |
| Pectobacterium parmentieri | 0 | 0 | 0 | 0 | 0 |
| Curtobacterium sp. BH-2-1-1 | 808 | 744 | 1135 | 16 | 1167 |
| Paenibacillus sp. JZ16 | 117 | 184 | 198 | 1 | 233 |
| Cellulosimicrobium sp. JZ28 | 507 | 922 | 977 | 9 | 1630 |
| Microbacterium sp. JZ31 | 1331 | 1435 | 2265 | 43 | 2048 |
| Cronobacter sp. JZ38 | 66 | 84 | 161 | 0 | 109 |
| Hyphomonas sp. Mor2 | 0 | 0 | 0 | 0 | 0 |
| Jeongeupia sp. USM3 | 1274 | 1771 | 1774 | 39 | 2635 |
| Microbacterium sp. BH-3-3-3 | 759 | 1089 | 12928 | 4 | 7176 |
| Rodentibacter heylii | 0 | 22 | 14 | 0 | 17 |
| Jatrophihabitans sp. GAS493 | 1612 | 1592 | 3203 | 92 | 4238 |
| Raoultibacter timonensis | 303 | 600 | 500 | 0 | 872 |
| Salinivibrio kushneri | 141 | 265 | 164 | 16 | 167 |
| Polaribacter litorisediminis | 7 | 23 | 22 | 0 | 7 |
| Nonomuraea sp. ATCC 55076 | 9217 | 5797 | 41906 | 7487 | 120543 |
| Desulfobulbus oligotrophicus | 123 | 497 | 302 | 0 | 349 |
| Tessaracoccus sp. T2.5-30 | 0 | 6762 | 0 | 0 | 0 |
| Amycolatopsis sp. BJA-103 | 18193 | 2460 | 8545 | 391 | 8797 |
| Marinilactibacillus sp. 15R | 10 | 12 | 22 | 0 | 12 |
| Virgibacillus sp. 6R | 19 | 99 | 21 | 0 | 0 |
| Staphylococcus sp. MI 10-1553 | 0 | 6 | 2 | 0 | 2 |
| Boudabousia tangfeifanii | 53 | 46 | 76 | 16 | 138 |
| Malaciobacter canalis | 2 | 1 | 0 | 0 | 2 |
| Blautia argi | 0 | 29 | 65 | 0 | 57 |
| Gramella salexigens | 13 | 73 | 25 | 2 | 23 |
| Sphingorhabdus lutea | 32 | 41 | 46 | 2 | 82 |
| Micromonospora terminaliae | 3077 | 2553 | 7714 | 398 | 8962 |
| Sulfuritortus calidifontis | 1674 | 2502 | 2693 | 99 | 3815 |
| Rhizobium sp. Y9 | 187 | 95 | 128 | 0 | 76 |
| Enterobacter sp. SA187 | 0 | 0 | 0 | 0 | 0 |
| Dolichospermum sp. UHCC 0315A | 0 | 0 | 0 | 0 | 0 |
| Thioclava nitratireducens | 0 | 0 | 0 | 0 | 0 |
| Silvanigrella aquatica | 12 | 136 | 27 | 1 | 54 |
| Enterobacter cloacae complex sp. ECNIH7 | 12 | 110 | 137 | 6 | 118 |
| Synechococcus sp. SynAce01 | 223 | 514 | 527 | 2 | 712 |
| Alteromonas sp. RW2A1 | 93 | 144 | 57 | 0 | 81 |
| Ketobacter alkanivorans | 0 | 0 | 0 | 0 | 0 |
| Streptococcus ruminantium | 3 | 2 | 2 | 0 | 2 |
| Sulfitobacter alexandrii | 1154 | 1562 | 1595 | 6 | 2068 |
| Leptospira kobayashii | 0 | 0 | 0 | 0 | 0 |
| Vibrio spartinae | 30 | 129 | 71 | 0 | 46 |
| Vibrio palustris | 24 | 42 | 30 | 1 | 51 |
| Aeromonas sp. ASNIH7 | 0 | 0 | 198 | 0 | 611 |
| Citrobacter sp. CFNIH10 | 17 | 15 | 19 | 0 | 28 |
| Leclercia sp. LSNIH1 | 63 | 53 | 203 | 0 | 55 |
| Mycobacterium sp. WY10 | 3457 | 5972 | 4997 | 0 | 8395 |
| Mariprofundus aestuarium | 58 | 184 | 227 | 2 | 208 |
| Mariprofundus ferrinatatus | 124 | 236 | 310 | 0 | 331 |
| Geobacillus genomosp. 3 | 0 | 0 | 0 | 0 | 0 |
| Tardibacter chloracetimidivorans | 1967 | 2636 | 1557 | 44 | 3176 |
| Proteus sp. CD3 | 1 | 5 | 6 | 0 | 22 |
| Bacillus haynesii | 39 | 10 | 7 | 2 | 2 |
| Candidatus Fonsibacter ubiquis | 3 | 15 | 17 | 0 | 95 |
| Rhodococcus sp. M8 | 908 | 875 | 1204 | 10 | 1786 |
| Cryobacterium sp. | 27 | 108 | 39 | 0 | 48 |
| Paraburkholderia sp. SOS3 | 1420 | 1758 | 1673 | 2 | 2292 |
| Acidovorax monticola | 874 | 1280 | 1358 | 0 | 1708 |
| Izhakiella australiensis | 0 | 0 | 0 | 0 | 12 |
| Candidatus Enterovibrio luxaltus | 9 | 17 | 13 | 0 | 20 |
| Sodalis endosymbiont of Henestaris halophilus | 5 | 46 | 7 | 0 | 14 |
| Streptomyces solisilvae | 7194 | 0 | 2549 | 660 | 6377 |
| Methylomusa anaerophila | 89 | 130 | 122 | 0 | 194 |
| Rubripirellula lacrimiformis | 931 | 2867 | 1891 | 52 | 3129 |
| Roseimaritima multifibrata | 433 | 1482 | 884 | 11 | 1480 |
| Adhaeretor mobilis | 1900 | 3389 | 2415 | 46 | 3714 |
| Rosistilla ulvae | 1048 | 2530 | 1734 | 28 | 3062 |
| Pseudomonas sp. CC6-YY-74 | 0 | 0 | 0 | 0 | 0 |
| Sporosarcina sp. P37 | 0 | 25 | 45 | 0 | 45 |
| Shewanella sp. FDAARGOS_354 | 242 | 15 | 45 | 0 | 92 |
| Candidatus Velamenicoccus archaeovorus | 0 | 0 | 0 | 0 | 0 |
| Sporosarcina sp. P33 | 30 | 88 | 8 | 0 | 28 |
| Neisseria dumasiana | 44 | 110 | 70 | 0 | 149 |
| Chryseobacterium sp. JV274 | 6 | 42 | 45 | 0 | 40 |
| Edaphobacter flagellatus | 1021 | 2980 | 2138 | 34 | 2305 |
| Sphingobacterium sp. B29 | 0 | 0 | 0 | 0 | 0 |
| Glutamicibacter halophytocola | 247 | 422 | 610 | 37 | 1216 |
| Bartonella sp. WD16.2 | 1 | 13 | 14 | 0 | 13 |
| Bartonella sp. JB15 | 0 | 0 | 123 | 0 | 0 |
| Bartonella sp. 11B | 195 | 0 | 0 | 0 | 0 |
| Bartonella sp. A1379B | 0 | 127 | 0 | 0 | 0 |
| Synechocystis sp. CACIAM 05 | 55 | 20 | 22 | 0 | 46 |
| [Arcobacter] porcinus | 1 | 0 | 2 | 0 | 0 |
| Leucobacter muris | 1034 | 1368 | 2108 | 93 | 2691 |
| Photobacterium toruni | 5 | 24 | 20 | 54 | 44 |
| Anaerohalosphaera lusitana | 235 | 632 | 488 | 2 | 712 |
| Thermosulfuriphilus ammonigenes | 0 | 0 | 0 | 0 | 0 |
| Mycobacterium sp. MS1601 | 1531 | 4417 | 3196 | 16 | 6583 |
| Winogradskyella sp. J14-2 | 12 | 48 | 126 | 3 | 85 |
| Seonamhaeicola sp. S2-3 | 0 | 0 | 0 | 0 | 0 |
| Nitrospira sp. KM1 | 1812 | 3626 | 3863 | 24 | 3421 |
| Microbacterium sp. TPU 3598 | 500 | 870 | 1222 | 7 | 1304 |
| Aquaspirillum sp. LM1 | 321 | 623 | 537 | 9 | 974 |
| Brevundimonas sp. LM2 | 535 | 996 | 881 | 18 | 1043 |
| Janthinobacterium sp. LM6 | 496 | 827 | 968 | 9 | 4931 |
| Sphingomonas sp. LM7 | 727 | 809 | 653 | 0 | 806 |
| Serratia sp. JKS000199 | 0 | 64 | 0 | 0 | 91 |
| Streptomyces sp. 2323.1 | 1034 | 547 | 1413 | 35 | 1465 |
| Streptococcus sp. DAT741 | 0 | 3 | 0 | 0 | 2 |
| Phreatobacter stygius | 5938 | 2981 | 2992 | 0 | 2478 |
| Pigmentiphaga aceris | 565 | 955 | 936 | 0 | 1270 |
| Pseudomonas shirazica | 38 | 169 | 299 | 11 | 297 |
| Sedimentisphaera cyanobacteriorum | 153 | 651 | 245 | 17 | 236 |
| Bacillus sp. 275 | 0 | 7320 | 130 | 0 | 596 |
| Sedimentisphaera salicampi | 92 | 183 | 97 | 0 | 134 |
| Cellulophaga sp. HaHaR_3_176 | 36 | 20 | 25 | 0 | 13 |
| Ponticoccus alexandrii | 0 | 0 | 0 | 0 | 0 |
| Phoenicibacter congonensis | 0 | 0 | 0 | 0 | 0 |
| blood disease bacterium A2-HR MARDI | 159 | 609 | 512 | 0 | 761 |
| Cellvibrio sp. PSBB023 | 62 | 207 | 146 | 8 | 136 |
| Capnocytophaga sp. H2931 | 0 | 0 | 30 | 0 | 5 |
| Capnocytophaga sp. H4358 | 0 | 343 | 0 | 0 | 0 |
| Paracoccus contaminans | 0 | 0 | 0 | 0 | 0 |
| Calothrix sp. NIES-2098 | 0 | 0 | 0 | 0 | 0 |
| Calothrix sp. NIES-2100 | 224 | 605 | 291 | 1 | 701 |
| Staphylococcus edaphicus | 0 | 7 | 10 | 0 | 10 |
| Actinomyces gaoshouyii | 388 | 481 | 581 | 25 | 822 |
| Streptomyces sp. MOE7 | 1303 | 990 | 1792 | 143 | 3133 |
| Geobacillus sp. 46C-IIa | 110 | 186 | 185 | 4 | 190 |
| Geobacillus sp. 47C-IIb | 0 | 0 | 0 | 0 | 110 |
| Candidatus Borreliella tachyglossi | 0 | 1 | 0 | 0 | 3 |
| Streptomyces sp. 3211 | 874 | 1082 | 1626 | 87 | 2848 |
| Campylobacter pinnipediorum | 0 | 8 | 10 | 0 | 22 |
| Shewanella khirikhana | 152 | 190 | 215 | 1 | 276 |
| Cognaticolwellia beringensis | 0 | 0 | 0 | 0 | 0 |
| Microcystis sp. MC19 | 40 | 106 | 64 | 2 | 91 |
| Chlamydia serpentis | 20 | 9 | 5 | 0 | 6 |
| Chlamydia poikilotherma | 0 | 5 | 2 | 0 | 1 |
| Sulfitobacter sp. D7 | 0 | 0 | 0 | 0 | 0 |
| Synechococcus sp. PROS-9-1 | 0 | 0 | 0 | 0 | 0 |
| Mycolicibacterium sp. CBMA 213 | 19 | 112 | 58 | 3 | 89 |
| Acidibrevibacterium fodinaquatile | 1548 | 1740 | 1427 | 0 | 2369 |
| Ciceribacter thiooxidans | 1355 | 1335 | 1386 | 11 | 1838 |
| Clostridium sp. 001 | 0 | 6 | 18 | 0 | 16 |
| Halomonas sp. GT | 26 | 81 | 101 | 1 | 112 |
| Candidatus Nardonella dryophthoridicola | 15 | 0 | 4 | 0 | 0 |
| endosymbiont of Pachyrhynchus infernalis | 0 | 0 | 5 | 0 | 0 |
| Sulfurivermis fontis | 1411 | 1696 | 1918 | 10 | 1672 |
| endosymbiont of Sipalinus gigas | 2 | 9 | 11 | 1 | 10 |
| Streptomyces sp. Sge12 | 712 | 853 | 1697 | 98 | 3222 |
| Agrobacterium rosae | 109 | 15 | 9 | 0 | 18 |
| Nostoc sp. NIES-2111 | 38 | 371 | 164 | 0 | 137 |
| Calothrix parasitica | 80 | 421 | 157 | 0 | 250 |
| Prosthecochloris sp. HL-130-GSB | 78 | 191 | 147 | 0 | 192 |
| Streptomyces ficellus | 2371 | 2011 | 3623 | 257 | 6780 |
| Candidatus Pelagibacter sp. RS39 | 2 | 12 | 11 | 0 | 16 |
| Candidatus Pelagibacter sp. RS40 | 0 | 0 | 0 | 0 | 0 |
| Cryobacterium sp. LW097 | 830 | 927 | 1341 | 62 | 1442 |
| Flavobacterium sp. MDT1-60 | 93 | 4148 | 807 | 0 | 744 |
| Cellulosimicrobium sp. TH-20 | 739 | 672 | 857 | 5 | 829 |
| Paenibacillus sp. Cedars | 260 | 183 | 187 | 0 | 424 |
| Rhizobium sp. NXC14 | 788 | 774 | 693 | 21 | 925 |
| Pseudomonas sp. M30-35 | 0 | 0 | 0 | 0 | 0 |
| Monoglobus pectinilyticus | 8 | 66 | 41 | 0 | 24 |
| Denitratisoma sp. DHT3 | 1839 | 2893 | 3490 | 20 | 4822 |
| Tsuneonella mangrovi | 0 | 0 | 0 | 0 | 0 |
| Faecalibacillus intestinalis | 0 | 0 | 0 | 0 | 0 |
| Advenella sp. S44 | 0 | 0 | 46 | 3 | 73 |
| Psychrobacter sp. L7 | 6 | 0 | 0 | 0 | 0 |
| Bacillus shivajii | 1 | 16 | 9 | 0 | 23 |
| Sphingobium sp. LB126 | 70 | 62 | 64 | 0 | 157 |
| Streptomyces sp. CLI2509 | 495 | 675 | 1108 | 90 | 1473 |
| Xanthomonas phaseoli | 1274 | 436 | 803 | 0 | 591 |
| Sulfuriferula sp. AH1 | 435 | 749 | 801 | 19 | 1075 |
| Desulfobulbus oralis | 281 | 676 | 555 | 8 | 931 |
| Brevefilum fermentans | 85 | 245 | 234 | 18 | 385 |
| Aurantimicrobium photophilum | 59 | 97 | 128 | 0 | 141 |
| Proteus columbae | 20 | 7 | 13 | 0 | 3 |
| Shewanella carassii | 42 | 55 | 47 | 25 | 52 |
| Cellvibrio sp. PSBB006 | 163 | 399 | 231 | 14 | 298 |
| Candidatus Profftia lariciata | 4 | 29 | 0 | 0 | 6 |
| Rhodococcus oxybenzonivorans | 452 | 1156 | 1190 | 29 | 1822 |
| Microbacterium sp. AISO3 | 0 | 126 | 33 | 0 | 54 |
| Sphingobacterium sp. G1-14 | 0 | 0 | 0 | 0 | 0 |
| Parolsenella catena | 345 | 577 | 699 | 6 | 976 |
| Porphyrobacter sp. CACIAM 03H1 | 702 | 923 | 1047 | 26 | 1318 |
| Pseudoalteromonas sp. GCY | 4 | 18 | 20 | 0 | 36 |
| Salinivibrio sp. YCSC6 | 0 | 55 | 18 | 0 | 32 |
| Cellulomonas sp. PSBB021 | 1924 | 2269 | 3644 | 116 | 5778 |
| Acinetobacter sp. WCHAc010052 | 1 | 28 | 0 | 0 | 4 |
| Acinetobacter chinensis | 0 | 0 | 0 | 0 | 0 |
| Stenotrophomonas sp. WZN-1 | 223 | 225 | 240 | 9 | 296 |
| Legionella endosymbiont of Polyplax serrata | 19 | 2 | 5 | 0 | 15 |
| Pseudomonas sp. RU47 | 62 | 296 | 378 | 0 | 934 |
| Fischerella sp. NIES-4106 | 75 | 231 | 109 | 4 | 410 |
| Nostoc sp. NIES-4103 | 149 | 552 | 331 | 15 | 709 |
| Chondrocystis sp. NIES-4102 | 34 | 194 | 157 | 1 | 177 |
| Calothrix sp. NIES-3974 | 21 | 191 | 136 | 0 | 136 |
| Scytonema sp. NIES-4073 | 268 | 745 | 592 | 4 | 950 |
| Calothrix sp. NIES-4071 | 0 | 0 | 378 | 0 | 0 |
| Enterococcus wangshanyuanii | 0 | 0 | 0 | 0 | 0 |
| Thauera sp. K11 | 2862 | 3837 | 4735 | 22 | 6634 |
| Streptomyces sp. S063 | 1093 | 1036 | 1566 | 141 | 2999 |
| Luteimonas chenhongjianii | 1295 | 1015 | 879 | 17 | 645 |
| Acinetobacter piscicola | 16 | 28 | 28 | 0 | 34 |
| Francisella adeliensis | 5 | 2 | 6 | 0 | 6 |
| Sagittula sp. P11 | 2314 | 2258 | 2290 | 38 | 3641 |
| Bacillus sp. MD-5 | 82 | 22 | 15 | 0 | 0 |
| Aerosticca soli | 3473 | 4849 | 5387 | 59 | 5758 |
| Caulobacter rhizosphaerae | 1176 | 2744 | 1103 | 0 | 636 |
| Pseudomonas laurylsulfatiphila | 330 | 514 | 321 | 6 | 690 |
| Lactobacillus panisapium | 1 | 14 | 7 | 0 | 11 |
| Bacillus sp. FJAT-42376 | 46 | 257 | 94 | 2 | 78 |
| Microbacterium sp. PM5 | 702 | 950 | 1421 | 7 | 1480 |
| Halomonas sp. N3-2A | 53 | 228 | 73 | 12 | 72 |
| Alcanivorax sp. N3-2A | 1422 | 1586 | 1571 | 40 | 1558 |
| Vibrio tarriae | 0 | 9 | 23 | 0 | 199 |
| Herbaspirillum robiniae | 1116 | 1541 | 1597 | 14 | 2235 |
| Streptomyces capitiformicae | 2050 | 1980 | 3349 | 220 | 6456 |
| Chryseobacterium sp. T16E-39 | 17 | 27 | 29 | 0 | 23 |
| Bosea sp. AS-1 | 3531 | 2734 | 2553 | 39 | 3419 |
| Klebsiella sp. LY | 294 | 182 | 795 | 0 | 222 |
| Leptolyngbya sp. PKUAC-SCTA174 | 77 | 279 | 251 | 6 | 370 |
| Leptolyngbya sp. PKUAC-SCTB231 | 164 | 262 | 274 | 3 | 420 |
| Hyphomonas sp. KY3 | 406 | 567 | 532 | 2 | 921 |
| Francisella opportunistica | 0 | 0 | 0 | 0 | 20 |
| Nitrogeniibacter mangrovi | 1301 | 2085 | 1948 | 26 | 2804 |
| Virgibacillus phasianinus | 0 | 20 | 26 | 0 | 23 |
| Brachybacterium vulturis | 636 | 463 | 911 | 61 | 679 |
| Brachybacterium avium | 525 | 456 | 675 | 31 | 977 |
| Nocardioides sp. S5 | 0 | 0 | 0 | 0 | 0 |
| Arthrobacter sp. PM3 | 746 | 2021 | 4218 | 18 | 5052 |
| Kitasatospora sp. MMS16-BH015 | 3433 | 2948 | 5138 | 591 | 9972 |
| Candidatus Vallotia lariciata | 50 | 36 | 95 | 0 | 141 |
| Roseomonas sp. FDAARGOS_362 | 1907 | 1350 | 1060 | 78 | 1666 |
| Shewanella bicestrii | 28 | 36 | 78 | 0 | 23 |
| Rhizobium sp. Kim5 | 847 | 427 | 352 | 16 | 433 |
| Rhizobium sp. CIAT894 | 695 | 756 | 713 | 7 | 728 |
| Rhizobium sp. TAL182 | 275 | 467 | 270 | 4 | 255 |
| Sinomonas sp. R1AF57 | 1253 | 1346 | 2149 | 44 | 3037 |
| Bosea sp. ANAM02 | 2791 | 2719 | 2052 | 8 | 1983 |
| Arthrobacter sp. YN | 188 | 503 | 12113 | 4 | 1245 |
| Paenibacillus konkukensis | 234 | 549 | 603 | 4 | 962 |
| Bifidobacterium imperatoris | 11 | 86 | 114 | 0 | 113 |
| Ahniella affigens | 731 | 1205 | 907 | 0 | 659 |
| Labrenzia sp. VG12 | 1039 | 992 | 794 | 34 | 1074 |
| Streptococcus respiraculi | 0 | 9 | 4 | 0 | 7 |
| Corynebacterium sp. LK10 | 0 | 1 | 3 | 0 | 0 |
| Porphyrobacter sp. HT-58-2 | 0 | 0 | 0 | 0 | 0 |
| Paenibacillus sp. RUD330 | 548 | 1194 | 1386 | 10 | 1561 |
| Plantactinospora sp. KBS50 | 3708 | 2865 | 8038 | 402 | 11164 |
| Tardiphaga sp. P9-11 | 0 | 3 | 1 | 1 | 5 |
| Novosphingobium sp. 9 | 789 | 1057 | 878 | 11 | 1307 |
| Pseudomonas sp. NS1(2017) | 0 | 0 | 0 | 0 | 0 |
| Vibrio qinghaiensis | 39 | 16 | 12 | 0 | 11 |
| Herbaspirillum sp. meg3 | 478 | 613 | 567 | 18 | 1169 |
| Bacillus paranthracis | 357 | 157 | 224 | 0 | 99 |
| Bacillus pacificus | 19 | 18 | 0 | 0 | 0 |
| Bacillus tropicus | 29 | 46 | 70 | 0 | 83 |
| Bacillus albus | 4 | 8 | 0 | 0 | 3 |
| Bacillus mobilis | 0 | 37 | 59 | 0 | 106 |
| Bacillus luti | 38 | 12 | 20 | 0 | 25 |
| Bacillus nitratireducens | 4 | 20 | 16 | 0 | 15 |
| Paraburkholderia aromaticivorans | 2507 | 3172 | 3409 | 41 | 4704 |
| Klebsiella quasivariicola | 0 | 0 | 0 | 0 | 0 |
| Parasphingopyxis algicola | 900 | 1227 | 862 | 10 | 2085 |
| Candidatus Bipolaricaulis anaerobius | 385 | 694 | 709 | 4 | 900 |
| Providencia huaxiensis | 0 | 0 | 37 | 0 | 0 |
| Azoarcus sp. DD4 | 2357 | 3576 | 3851 | 36 | 6092 |
| Mariniflexile sp. TRM1-10 | 22 | 80 | 86 | 1 | 91 |
| Mucilaginibacter rubeus | 193 | 468 | 435 | 0 | 724 |
| Cupriavidus sp. P-10 | 1201 | 1606 | 1784 | 3 | 2694 |
| Enterobacter cloacae complex sp. | 261 | 898 | 1630 | 21 | 2469 |
| Rhizobium sp. 11515TR | 607 | 815 | 554 | 42 | 755 |
| Pusillimonas thiosulfatoxidans | 525 | 653 | 602 | 15 | 972 |
| Marinicauda algicola | 1972 | 2494 | 2732 | 93 | 3710 |
| Shewanella sp. WE21 | 23 | 49 | 57 | 24 | 40 |
| Glaesserella sp. 15-184 | 1 | 23 | 15 | 0 | 24 |
| Aeromonas sp. CA23 | 241 | 153 | 238 | 0 | 149 |
| Aeromonas sp. CU5 | 57 | 142 | 125 | 9 | 322 |
| Chitinophaga sp. MD30 | 0 | 0 | 0 | 0 | 0 |
| Serratia sp. MYb239 | 19 | 69 | 165 | 0 | 184 |
| Actinoplanes sp. SE50 | 1193 | 0 | 2135 | 0 | 1976 |
| Rhodobacter sp. CZR27 | 1298 | 1658 | 1863 | 75 | 2290 |
| Streptomyces sp. Tue6028 | 1 | 9 | 11 | 0 | 14 |
| Suicoccus acidiformans | 0 | 0 | 0 | 0 | 0 |
| Dickeya sp. Secpp 1600 | 50 | 26 | 50 | 0 | 20 |
| Jatrophihabitans telluris | 1622 | 1678 | 3096 | 103 | 4262 |
| Micromonospora sp. WMMA2032 | 2537 | 2250 | 5969 | 233 | 6466 |
| Stenotrophomonas sp. Pemsol | 411 | 174 | 316 | 0 | 358 |
| Eubacterium maltosivorans | 6 | 104 | 221 | 0 | 111 |
| Pectobacterium polaris | 37 | 176 | 177 | 2 | 164 |
| Campylobacter blaseri | 10 | 2 | 2 | 0 | 7 |
| Paremcibacter congregatus | 0 | 0 | 0 | 0 | 0 |
| Escherichia sp. E4742 | 19 | 55 | 73 | 1 | 67 |
| Candidatus Pantoea edessiphila | 0 | 0 | 0 | 0 | 2 |
| Staphylococcus debuckii | 0 | 9 | 9 | 0 | 17 |
| Pseudomonas rhizophila | 0 | 0 | 0 | 0 | 0 |
| Massilia violaceinigra | 2039 | 5178 | 3795 | 127 | 8481 |
| Stenotrophomonas indicatrix | 453 | 382 | 460 | 26 | 476 |
| Nocardioides houyundeii | 1230 | 1661 | 2601 | 157 | 2950 |
| Thermosulfurimonas marina | 150 | 311 | 370 | 2 | 470 |
| Dongshaea marina | 118 | 170 | 165 | 4 | 248 |
| Thalassospira marina | 318 | 434 | 286 | 4 | 486 |
| Solibacillus sp. R5-41 | 0 | 0 | 0 | 0 | 0 |
| Rhizobium sp. NXC24 | 525 | 341 | 326 | 13 | 334 |
| Microbacterium sp. Y-01 | 688 | 872 | 1384 | 43 | 1545 |
| Changchengzhania lutea | 23 | 32 | 17 | 0 | 34 |
| Methylomicrobium sp. wino1 | 0 | 0 | 0 | 0 | 0 |
| Pseudomonas sp. HLS-6 | 0 | 0 | 0 | 0 | 0 |
| Streptomyces dengpaensis | 2266 | 2008 | 3392 | 209 | 6563 |
| Bacillus sp. Lzh-5 | 0 | 0 | 0 | 0 | 671 |
| Mycobacterium sp. PYR15 | 2322 | 6235 | 6473 | 51 | 11942 |
| Methylobacterium currus | 2187 | 2565 | 2603 | 26 | 2718 |
| Exiguobacterium sp. N4-1P | 0 | 0 | 0 | 0 | 0 |
| Rubrobacter indicoceani | 773 | 945 | 1291 | 11 | 1613 |
| Edaphobacter lichenicola | 1188 | 2305 | 1933 | 13 | 2149 |
| Pantoea sp. MSR2 | 45 | 185 | 147 | 3 | 288 |
| Dietzia sp. JS16-p6b | 1178 | 0 | 0 | 0 | 980 |
| Oceanobacillus zhaokaii | 2 | 27 | 12 | 0 | 22 |
| Pseudomonas sp. ACM7 | 675 | 2727 | 5205 | 5 | 8226 |
| Acinetobacter pseudolwoffii | 0 | 48 | 28 | 6 | 26 |
| Cobetia sp. ICG0124 | 0 | 0 | 54 | 0 | 0 |
| Xanthomonas prunicola | 0 | 0 | 0 | 0 | 0 |
| Candidatus Coxiella mudrowiae | 57 | 85 | 62 | 1 | 72 |
| Sphingopyxis lindanitolerans | 688 | 1212 | 532 | 17 | 507 |
| Rhodococcus sp. AQ5-07 | 238 | 192 | 187 | 0 | 320 |
| Pseudomonas sp. 02C 26 | 55 | 213 | 142 | 0 | 2392 |
| Pseudomonas sp. 09C 129 | 145 | 102 | 134 | 0 | 271 |
| Kyrpidia spormannii | 0 | 0 | 0 | 0 | 0 |
| Nocardia tengchongensis | 2307 | 1983 | 3735 | 148 | 4984 |
| Idiomarina sp. X4 | 42 | 56 | 32 | 6 | 53 |
| Corynebacterium heidelbergense | 295 | 386 | 455 | 25 | 973 |
| Pseudomonas qingdaonensis | 124 | 157 | 200 | 1 | 312 |
| Streptomyces sp. AMCC400023 | 2321 | 2003 | 3208 | 119 | 5918 |
| Spirosoma pollinicola | 132 | 514 | 391 | 7 | 414 |
| Acidipropionibacterium virtanenii | 260 | 432 | 937 | 40 | 1212 |
| Actinomyces wuliandei | 324 | 447 | 629 | 41 | 658 |
| Enterococcus sp. CR-Ec1 | 0 | 14 | 6 | 0 | 18 |
| Actinomyces sp. 432 | 0 | 0 | 0 | 0 | 0 |
| Actinomyces qiguomingii | 317 | 402 | 582 | 6 | 607 |
| Lacinutrix sp. Bg11-31 | 4 | 13 | 11 | 0 | 14 |
| Alteromonas sp. MB-3u-76 | 0 | 22 | 26 | 16 | 61 |
| Nonlabens sp. MB-3u-79 | 12 | 22 | 36 | 0 | 42 |
| Olleya sp. Bg11-27 | 7 | 11 | 14 | 0 | 21 |
| Polaribacter sp. ALD11 | 2 | 18 | 15 | 0 | 9 |
| Antarcticibacterium flavum | 0 | 0 | 0 | 0 | 0 |
| Psychrobacter sp. Sarcosine-02u-2 | 0 | 0 | 0 | 0 | 0 |
| Undibacterium sp. YM2 | 0 | 0 | 0 | 0 | 0 |
| Shewanella sp. Pdp11 | 0 | 17 | 40 | 0 | 19 |
| Chromobacterium sp. ATCC 53434 | 937 | 1422 | 1302 | 44 | 1700 |
| Gordonia sp. YC-JH1 | 2280 | 2235 | 3493 | 146 | 4726 |
| Streptomyces sp. CMB-StM0423 | 1917 | 1726 | 2738 | 248 | 6943 |
| Enterococcus sp. FDAARGOS_375 | 0 | 0 | 4 | 0 | 11 |
| Altererythrobacter sp. B11 | 0 | 0 | 0 | 0 | 0 |
| Neorhizobium sp. SOG26 | 1039 | 956 | 833 | 8 | 1119 |
| Marinomonas sp. A3A | 30 | 129 | 52 | 4 | 40 |
| Paracoccus jeotgali | 0 | 0 | 0 | 0 | 0 |
| Candidatus Cytomitobacter primus | 1 | 1 | 19 | 0 | 13 |
| Citrobacter freundii complex sp. CFNIH2 | 55 | 89 | 61 | 1 | 230 |
| Mesorhizobium japonicum | 1845 | 1465 | 1958 | 27 | 1736 |
| Marinomonas sp. CT5 | 18 | 41 | 71 | 1 | 56 |
| Candidatus Chromulinivorax destructor | 37 | 100 | 29 | 2 | 16 |
| Aquella oligotrophica | 25 | 37 | 19 | 2 | 24 |
| Altererythrobacter sp. TH136 | 0 | 0 | 0 | 0 | 0 |
| Pseudomonas sp. NC02 | 94 | 135 | 80 | 7 | 372 |
| Kosakonia sp. MUSA4 | 62 | 144 | 147 | 0 | 184 |
| Lelliottia sp. AC1 | 0 | 0 | 0 | 0 | 0 |
| Pseudazoarcus pumilus | 1439 | 1827 | 1936 | 5 | 2624 |
| Clostridium fermenticellae | 25 | 11 | 11 | 0 | 15 |
| Novisyntrophococcus fermenticellae | 36 | 100 | 24 | 0 | 86 |
| Paenibacillus sp. lzh-N1 | 0 | 0 | 40 | 0 | 89 |
| Pseudomonas sp. XWY-1 | 50 | 139 | 81 | 0 | 112 |
| Tamlana carrageenivorans | 23 | 21 | 28 | 0 | 39 |
| Curtobacterium sp. SGAir0471 | 348 | 611 | 983 | 0 | 800 |
| Agrococcus sp. SGAir0287 | 1085 | 1382 | 1961 | 26 | 2026 |
| Microbacterium sp. SGAir0570 | 367 | 622 | 1124 | 0 | 1364 |
| Sulfitobacter sp. JL08 | 0 | 0 | 0 | 0 | 0 |
| Lysinibacillus sp. SGAir0095 | 1 | 62 | 13 | 0 | 17 |
| Commensalibacter sp. ESL0284 | 0 | 5 | 9 | 0 | 34 |
| Bacillus sp. MBGLi79 | 0 | 11 | 23 | 0 | 9 |
| Sphingomonas rhizophila | 1094 | 1674 | 1144 | 0 | 1740 |
| Marinomonas sp. FW-1 | 49 | 83 | 29 | 6 | 56 |
| Plantactinospora sp. BB1 | 2575 | 2074 | 6830 | 374 | 9116 |
| Enterobacter sichuanensis | 35 | 82 | 49 | 0 | 68 |
| Lysinibacillus sp. YS11 | 0 | 10 | 9 | 0 | 3 |
| Stenotrophomonas sp. ZAC14D2_NAIMI4_7 | 257 | 347 | 399 | 0 | 590 |
| Stenotrophomonas sp. ZAC14D2_NAIMI4_6 | 89 | 145 | 251 | 0 | 300 |
| Stenotrophomonas sp. YAU14D1_LEIMI4_1 | 195 | 216 | 333 | 9 | 457 |
| Stenotrophomonas sp. YAU14A_MKIMI4_1 | 0 | 0 | 0 | 0 | 0 |
| Stenotrophomonas sp. SAU14A_NAIMI4_8 | 243 | 266 | 370 | 0 | 460 |
| Stenotrophomonas sp. ZAC14D1_NAIMI4_6 | 0 | 1192 | 0 | 0 | 0 |
| Stenotrophomonas sp. ZAC14A_NAIMI4_1 | 484 | 248 | 371 | 0 | 689 |
| Stenotrophomonas sp. SAU14A_NAIMI4_5 | 377 | 548 | 628 | 43 | 927 |
| Streptomyces sp. Go-475 | 1863 | 1728 | 2720 | 159 | 4187 |
| Pseudoduganella armeniaca | 875 | 2203 | 1697 | 0 | 3178 |
| Paenibacillus sp. PK3_47 | 73 | 208 | 291 | 6 | 254 |
| Sphingobium sp. SCG-1 | 0 | 0 | 0 | 0 | 0 |
| Pseudomonas sp. DTU12.3 | 325 | 434 | 293 | 10 | 1590 |
| Vibrio gangliei | 44 | 36 | 57 | 1 | 44 |
| Enterobacter cloacae complex sp. FDA-CDC-AR_0164 | 0 | 8 | 10 | 0 | 24 |
| Enterobacter cloacae complex sp. FDA-CDC-AR_0132 | 188 | 98 | 354 | 0 | 95 |
| Citrobacter freundii complex sp. CFNIH3 | 0 | 198 | 0 | 0 | 191 |
| Streptomyces sp. CB01881 | 3429 | 2928 | 6049 | 444 | 11906 |
| Arthrobacter sp. PGP41 | 650 | 1044 | 1717 | 58 | 3187 |
| Corynebacterium gerontici | 191 | 231 | 382 | 2 | 312 |
| Actinomyces sp. Z16 | 436 | 456 | 875 | 34 | 1283 |
| Aurantimicrobium sp. MWH-Uga1 | 19 | 83 | 60 | 12 | 90 |
| Acinetobacter sp. SWBY1 | 0 | 7 | 4 | 0 | 6 |
| Salinibacterium hongtaonis | 416 | 672 | 823 | 25 | 1008 |
| Mycetocola zhujimingii | 630 | 691 | 939 | 28 | 1183 |
| Aeromicrobium chenweiae | 7682 | 15173 | 11132 | 138 | 6088 |
| Deinococcus sp. NW-56 | 341 | 1029 | 872 | 0 | 996 |
| Agromyces badenianii | 3705 | 3086 | 6737 | 60 | 6877 |
| Corynebacterium pseudopelargi | 50 | 168 | 263 | 9 | 207 |
| Polynucleobacter sp. AP-Kolm-20A-A1 | 13 | 39 | 23 | 0 | 25 |
| Polynucleobacter corsicus | 21 | 29 | 17 | 2 | 30 |
| Polynucleobacter sp. AP-Sving-400A-A2 | 20 | 28 | 44 | 0 | 17 |
| Polynucleobacter sp. MG-5-Ahmo-C2 | 0 | 65 | 42 | 0 | 51 |
| Polynucleobacter sp. MG-Unter2-18 | 4 | 22 | 22 | 0 | 23 |
| Xanthomonas sp. MLO165 | 0 | 0 | 0 | 0 | 0 |
| Actinomyces sp. oral taxon 897 | 238 | 291 | 500 | 9 | 705 |
| Rhizobium ruizarguesonis | 814 | 1762 | 1787 | 33 | 793 |
| Polaromonas sp. Pch-P | 0 | 0 | 1395 | 0 | 2646 |
| Methylibium sp. Pch-M | 1270 | 1804 | 1754 | 34 | 3373 |
| Mesorhizobium sp. Pch-S | 0 | 0 | 0 | 0 | 0 |
| Microvirga sp. 17 mud 1-3 | 2368 | 1898 | 1907 | 34 | 2389 |
| Pseudomonas sp. SWI6 | 10 | 76 | 69 | 61 | 188 |
| Pseudomonas sp. SWI36 | 19 | 110 | 150 | 3 | 151 |
| Pseudomonas sp. SWI44 | 93 | 165 | 139 | 0 | 355 |
| Pseudomonas sp. LH1G9 | 0 | 0 | 0 | 0 | 0 |
| Streptomyces sp. CB09001 | 1234 | 1022 | 2097 | 181 | 3350 |
| Devosia sp. I507 | 1373 | 1012 | 888 | 0 | 986 |
| Adhaeribacter swui | 0 | 0 | 0 | 0 | 0 |
| Lysinibacillus timonensis | 34 | 31 | 18 | 0 | 24 |
| Massilistercora timonensis | 311 | 310 | 393 | 7 | 941 |
| Christensenella sp. Marseille-P3954 | 474 | 835 | 913 | 3 | 1344 |
| Candidatus Nanosynbacter lyticus | 26 | 34 | 26 | 0 | 123 |
| Bacillus sp. ZY-1-1 | 784 | 49 | 0 | 0 | 0 |
| Butyricimonas faecalis | 113 | 193 | 166 | 17 | 128 |
| Dysosmobacter welbionis | 241 | 542 | 736 | 9 | 874 |
| Streptomyces sp. WAC00288 | 522 | 721 | 1299 | 55 | 2273 |
| Pukyongia salina | 34 | 74 | 33 | 1 | 36 |
| Mycobacterium basiliense | 1298 | 2229 | 1621 | 29 | 3660 |
| Tolypothrix sp. PCC 7910 | 0 | 0 | 0 | 0 | 0 |
| Streptomyces sp. ICC1 | 133 | 525 | 1001 | 632 | 8614 |
| Streptomyces sp. ICC4 | 151 | 364 | 1729 | 126 | 4008 |
| Salicibibacter kimchii | 8 | 87 | 81 | 31 | 280 |
| Lactobacillus sp. CBA3605 | 24 | 0 | 8 | 0 | 5 |
| Lactobacillus sp. CBA3606 | 8 | 10 | 10 | 0 | 13 |
| Vibrio echinoideorum | 15 | 41 | 44 | 0 | 66 |
| Idiomarina sp. OT37-5b | 0 | 0 | 0 | 0 | 0 |
| Lacticaseibacillus chiayiensis | 29 | 29 | 45 | 0 | 54 |
| Aeromicrobium sp. A1-2 | 6692 | 13588 | 9771 | 164 | 5404 |
| Lactobacillus paragasseri | 0 | 0 | 3 | 0 | 4 |
| Tepiditoga spiralis | 41 | 10 | 5 | 0 | 14 |
| Pectobacterium punjabense | 0 | 0 | 0 | 0 | 0 |
| Mesorhizobium sp. DCY119 | 3377 | 3097 | 2939 | 16 | 3527 |
| Plantactinospora sp. BC1 | 3258 | 1761 | 5525 | 321 | 6818 |
| Halobacteriovorax sp. BALOs_7 | 3 | 30 | 6 | 0 | 18 |
| Streptomyces sp. SGAir0924 | 945 | 667 | 1223 | 79 | 2534 |
| Butyricicoccus sp. GAM44 | 0 | 2 | 1 | 0 | 2 |
| Melaminivora suipulveris | 1290 | 1619 | 1820 | 0 | 2290 |
| Ottowia oryzae | 0 | 0 | 0 | 0 | 0 |
| Simplicispira suum | 740 | 1311 | 1316 | 0 | 1745 |
| Lysinibacillus capsici | 0 | 0 | 0 | 0 | 0 |
| Candidatus Phycorickettsia trachydisci | 18 | 13 | 19 | 0 | 28 |
| Mycetocola sp. JXN-3 | 261 | 632 | 791 | 70 | 1016 |
| Pulveribacter suum | 615 | 830 | 773 | 0 | 906 |
| Nitrosomonas supralitoralis | 0 | 3 | 0 | 0 | 5 |
| Pseudomonas sp. LBUM920 | 0 | 0 | 0 | 0 | 0 |
| Variovorax sp. PMC12 | 0 | 0 | 0 | 0 | 0 |
| Nissabacter sp. SGAir0207 | 345 | 631 | 555 | 2 | 737 |
| Peterkaempfera bronchialis | 2543 | 2130 | 4674 | 565 | 11549 |
| Gramella fulva | 88 | 108 | 75 | 0 | 89 |
| Rhodopirellula sp. P2 | 720 | 2557 | 1594 | 9 | 2607 |
| Pseudoprevotella muciniphila | 19 | 47 | 56 | 1 | 102 |
| Bartonella kosoyi | 9 | 9 | 4 | 0 | 64 |
| Streptomyces sp. P3 | 2822 | 2035 | 3810 | 403 | 7334 |
| Halomonas sp. SF2003 | 338 | 827 | 365 | 0 | 537 |
| Streptomyces sp. So13.3 | 3262 | 2808 | 6280 | 424 | 10230 |
| Acinetobacter cumulans | 18 | 100 | 9 | 16 | 9 |
| Streptomyces sp. YIM 121038 | 2101 | 2056 | 4031 | 343 | 7404 |
| Moorella sp. Hama-1 | 251 | 466 | 485 | 4 | 755 |
| Megasphaera stantonii | 419 | 601 | 507 | 1 | 614 |
| Klebsiella huaxiensis | 0 | 0 | 0 | 0 | 0 |
| Lelliottia sp. WB101 | 0 | 0 | 0 | 0 | 0 |
| Streptomyces sp. endophyte_N2 | 688 | 623 | 1005 | 263 | 3138 |
| Kordiimonas pumila | 56 | 72 | 52 | 2 | 71 |
| Saccharospirillum mangrovi | 268 | 688 | 410 | 13 | 480 |
| Tessaracoccus timonensis | 808 | 758 | 1213 | 2 | 1314 |
| Dialister massiliensis | 0 | 83 | 86 | 0 | 141 |
| Colwellia sp. Arc7-D | 12 | 15 | 24 | 3 | 38 |
| Flavobacterium magnum | 38 | 188 | 150 | 2 | 289 |
| Orrella marina | 242 | 361 | 365 | 3 | 429 |
| Vibrio sp. dhg | 0 | 7 | 11 | 0 | 19 |
| Candidatus Deianiraea vastatrix | 3 | 14 | 4 | 0 | 14 |
| Gemmobacter aquarius | 763 | 906 | 966 | 9 | 1310 |
| Lysinibacillus sp. 2017 | 19 | 25 | 24 | 0 | 23 |
| Pseudomonas sp. SXM-1 | 0 | 0 | 0 | 0 | 0 |
| Methyloceanibacter sp. wino2 | 3081 | 2213 | 2073 | 37 | 2423 |
| Auritidibacter sp. NML130574 | 181 | 270 | 349 | 54 | 831 |
| Bradyrhizobium sp. WBOS02 | 1624 | 0 | 0 | 0 | 0 |
| Bradyrhizobium sp. WBOS04 | 1600 | 1040 | 0 | 0 | 0 |
| Bradyrhizobium sp. WBOS07 | 0 | 0 | 2814 | 0 | 1371 |
| Bradyrhizobium sp. WBOS08 | 5076 | 0 | 0 | 0 | 0 |
| Miniimonas sp. S16 | 2979 | 3145 | 5278 | 228 | 7031 |
| Profundibacter amoris | 205 | 420 | 265 | 1 | 414 |
| Flavobacterium pallidum | 31 | 249 | 190 | 0 | 212 |
| Saccharobesus litoralis | 26 | 35 | 42 | 6 | 34 |
| Limnobaculum parvum | 34 | 25 | 37 | 2 | 60 |
| Petrocella atlantisensis | 4 | 27 | 24 | 1 | 19 |
| Dokdonia sp. Dokd-P16 | 10 | 8 | 7 | 0 | 12 |
| Cellulomonas sp. WB94 | 209 | 410 | 498 | 1 | 476 |
| Streptococcus chenjunshii | 11 | 13 | 6 | 1 | 14 |
| Streptomyces tirandamycinicus | 1320 | 1513 | 2979 | 184 | 5034 |
| Flavobacterium album | 0 | 0 | 0 | 0 | 0 |
| Streptomyces sp. NHF165 | 3012 | 2297 | 4255 | 430 | 7937 |
| Erythrobacter aureus | 0 | 0 | 0 | 0 | 0 |
| Spiribacter sp. E85 | 1890 | 2410 | 2438 | 7 | 3105 |
| Saliniradius amylolyticus | 65 | 146 | 223 | 2 | 177 |
| Flavobacterium crocinum | 40 | 252 | 239 | 0 | 138 |
| Salinisphaera sp. LB1 | 1299 | 1586 | 1579 | 30 | 1760 |
| Streptomyces cadmiisoli | 2311 | 2259 | 3524 | 270 | 6920 |
| Pseudothauera hydrothermalis | 428 | 862 | 799 | 0 | 1228 |
| Hydrogenophaga sp. NH-16 | 0 | 0 | 0 | 0 | 0 |
| Acetobacterium sp. KB-1 | 101 | 209 | 103 | 1 | 352 |
| Arthrobacter sp. AQ5-05 | 89 | 284 | 642 | 27 | 1009 |
| Sphingobium sp. LF-16 | 282 | 191 | 285 | 0 | 348 |
| Flavobacterium sediminis | 0 | 0 | 0 | 0 | 0 |
| Pedobacter schmidteae | 76 | 150 | 169 | 0 | 490 |
| Phenylobacterium parvum | 1274 | 1856 | 1375 | 18 | 1947 |
| Pseudomonas sp. 31-12 | 263 | 526 | 680 | 16 | 1021 |
| Micromonospora sp. B006 | 2210 | 1570 | 5609 | 295 | 7746 |
| Streptomyces sp. NEAU-S7GS2 | 834 | 904 | 1425 | 23 | 2118 |
| Chromobacterium phragmitis | 482 | 836 | 884 | 43 | 1574 |
| Azospirillum thermophilum | 3011 | 3446 | 3531 | 21 | 5307 |
| Thermomonas aquatica | 1333 | 1806 | 1395 | 0 | 939 |
| Streptosporangium sp. 'caverna' | 8708 | 5547 | 65237 | 111090 | 1660134 |
| Deinococcus irradiatisoli | 470 | 759 | 772 | 30 | 883 |
| Zhouia spongiae | 51 | 158 | 37 | 0 | 51 |
| Methylobacterium durans | 2509 | 2852 | 2699 | 19 | 3306 |
| Methylobacterium sp. 17Sr1-1 | 2321 | 2086 | 2361 | 34 | 3174 |
| Methylobacterium terrae | 2277 | 2550 | 2420 | 48 | 3228 |
| Methylobacterium radiodurans | 2284 | 2673 | 2495 | 24 | 3330 |
| Streptomyces sp. WAC 01438 | 1868 | 1684 | 3339 | 275 | 5512 |
| Streptomyces sp. WAC 01529 | 1948 | 1404 | 2674 | 259 | 4631 |
| Streptomyces sp. WAC 06738 | 1709 | 1423 | 2407 | 57 | 2717 |
| Chitinophaga alhagiae | 116 | 226 | 213 | 15 | 416 |
| Oenococcus sicerae | 14 | 49 | 33 | 0 | 47 |
| Psychrobacter sp. YP14 | 8 | 9 | 2 | 0 | 43 |
| Pectobacterium aquaticum | 7 | 23 | 31 | 0 | 32 |
| Thiomicrorhabdus aquaedulcis | 9 | 66 | 62 | 1 | 73 |
| Acidisarcina polymorpha | 1213 | 3649 | 2710 | 7 | 3380 |
| Arthrobacter dokdonellae | 686 | 935 | 1388 | 164 | 1816 |
| Paraburkholderia dokdonella | 602 | 935 | 851 | 0 | 1107 |
| Paenibacillus sp. DCT19 | 27 | 38 | 74 | 3 | 76 |
| Clostridium sp. AWRP | 14 | 7 | 16 | 0 | 17 |
| Pseudomonas sichuanensis | 190 | 239 | 307 | 2 | 436 |
| Paraliobacillus zengyii | 11 | 20 | 24 | 0 | 14 |
| Nocardia mangyaensis | 2589 | 2266 | 3921 | 217 | 4018 |
| Planococcus lenghuensis | 0 | 0 | 0 | 0 | 0 |
| Entomomonas moraniae | 1 | 100 | 22 | 31 | 19 |
| Pseudomonas sp. SGAir0191 | 84 | 115 | 141 | 15 | 223 |
| Photorhabdus laumondii | 29 | 104 | 94 | 0 | 68 |
| Pseudomonas sp. LG1E9 | 154 | 391 | 564 | 3 | 2331 |
| Nonomuraea phyllanthi | 9216 | 5276 | 42525 | 8003 | 136888 |
| Pseudomonas asiatica | 311 | 1687 | 953 | 0 | 905 |
| Sphingomonas sp. FARSPH | 1397 | 1931 | 1876 | 80 | 3491 |
| Bdellovibrio sp. NC01 | 0 | 0 | 0 | 0 | 0 |
| Cryobacterium soli | 734 | 1081 | 1269 | 40 | 1965 |
| Indioceanicola profundi | 2010 | 2088 | 2110 | 4 | 3234 |
| Bdellovibrio sp. ZAP7 | 56 | 75 | 124 | 0 | 94 |
| Dechloromonas sp. HYN0024 | 453 | 749 | 621 | 10 | 1067 |
| Blastochloris tepida | 5459 | 3304 | 3836 | 115 | 4916 |
| Bacillus sp. COPE52 | 3 | 0 | 3 | 0 | 0 |
| Skermanella pratensis | 1683 | 2110 | 1957 | 52 | 2969 |
| Thermodesulfobacterium sp. TA1 | 30 | 51 | 17 | 0 | 18 |
| Francisella marina | 0 | 1 | 6 | 0 | 3 |
| Flavobacterium fluviale | 58 | 264 | 982 | 0 | 114 |
| Alcanivorax sp. ALC70 | 651 | 942 | 925 | 0 | 575 |
| Paracoccus suum | 571 | 879 | 861 | 5 | 1365 |
| Runella rosea | 133 | 289 | 208 | 0 | 204 |
| Xanthomonas euroxanthea | 0 | 0 | 0 | 0 | 0 |
| Oenococcus sp. UCMA 16435 | 3 | 13 | 22 | 0 | 47 |
| Raoultella sp. X13 | 0 | 56 | 0 | 0 | 37 |
| Acetobacter sp. JWB | 0 | 0 | 0 | 0 | 419 |
| Thiomicrorhabdus indica | 3 | 20 | 31 | 0 | 18 |
| Alteromonas sp. RKMC-009 | 0 | 0 | 0 | 0 | 0 |
| Bartonella krasnovii | 2 | 0 | 4 | 0 | 23 |
| Klebsiella sp. P1CD1 | 0 | 0 | 19 | 0 | 0 |
| Rhizobium oryzihabitans | 0 | 0 | 0 | 0 | 0 |
| Campylobacter novaezeelandiae | 0 | 3 | 5 | 0 | 5 |
| Ephemeroptericola cinctiostellae | 105 | 107 | 127 | 3 | 239 |
| Parasaccharibacter sp. TMW 2.1888 | 62 | 90 | 115 | 0 | 177 |
| Runella sp. SP2 | 95 | 190 | 210 | 0 | 281 |
| Polaromonas sp. SP1 | 0 | 2118 | 0 | 0 | 6400 |
| Actinokineospora sp. UTMC 2448 | 27297 | 4841 | 24076 | 508 | 14145 |
| Candidatus Pelagibacter sp. FZCC0015 | 0 | 5 | 11 | 0 | 13 |
| Microbacterium sp. ABRD_28 | 603 | 826 | 1497 | 0 | 1579 |
| Lactobacillus terrae | 11 | 3 | 4 | 0 | 5 |
| Stenotrophomonas sp. ASS1 | 314 | 214 | 209 | 72 | 290 |
| Kordia sp. SMS9 | 14 | 39 | 43 | 0 | 32 |
| Leclercia sp. W17 | 125 | 140 | 204 | 0 | 186 |
| Leclercia sp. W6 | 176 | 98 | 63 | 0 | 107 |
| Staphylococcus pseudoxylosus | 55 | 27 | 13 | 0 | 10 |
| Achromobacter sp. B7 | 488 | 855 | 724 | 24 | 985 |
| Streptomyces paludis | 3104 | 2047 | 4189 | 192 | 6493 |
| Paenibacillus sp. H1-7 | 152 | 517 | 354 | 1 | 572 |
| Sporosarcina sp. PTS2304 | 1 | 29 | 11 | 1 | 52 |
| Ornithinimicrobium avium | 814 | 1289 | 2198 | 114 | 1885 |
| Aquirhabdus parva | 29 | 155 | 84 | 2 | 170 |
| Sulfurimonas sp. CVO | 0 | 33 | 25 | 0 | 40 |
| Lysobacter sp. TY2-98 | 1940 | 2011 | 1883 | 35 | 2379 |
| Crenobacter cavernae | 1452 | 2106 | 2153 | 103 | 4617 |
| Gallaecimonas mangrovi | 53 | 102 | 100 | 16 | 154 |
| Rhizobium sp. ZX09 | 550 | 487 | 398 | 0 | 694 |
| Levilactobacillus suantsaii | 31 | 83 | 70 | 1 | 116 |
| Chromobacterium sp. Rain0013 | 394 | 418 | 519 | 25 | 506 |
| Persicimonas caeni | 1521 | 3376 | 3458 | 5 | 4313 |
| Candidatus Syntrophocurvum alkaliphilum | 4 | 10 | 21 | 0 | 20 |
| Streptomyces inhibens | 1942 | 1284 | 3253 | 366 | 7404 |
| Thermaerobacter sp. PB12/4term | 1157 | 1705 | 2105 | 30 | 2742 |
| Ruegeria sp. AD91A | 210 | 177 | 209 | 4 | 205 |
| Micromonospora craniellae | 1944 | 1716 | 3916 | 570 | 9114 |
| Flavobacterium sp. CJ74 | 0 | 0 | 0 | 0 | 0 |
| Solimonas sp. K1W22B-7 | 2592 | 3484 | 3600 | 36 | 3645 |
| Cellvibrio sp. KY-GH-1 | 75 | 315 | 158 | 16 | 155 |
| Psychrobacillus sp. AK 1817 | 0 | 17 | 15 | 0 | 16 |
| Salinimonas sediminis | 63 | 190 | 65 | 0 | 94 |
| Stenotrophomonas sp. G4 | 334 | 182 | 138 | 0 | 211 |
| Marinobacter sp. Arc7-DN-1 | 0 | 0 | 0 | 0 | 0 |
| Breoghania sp. L-A4 | 2860 | 2379 | 2269 | 75 | 3119 |
| Companilactobacillus zhachilii | 1 | 4 | 0 | 0 | 5 |
| Streptomyces sp. W1SF4 | 1827 | 1934 | 4030 | 190 | 5616 |
| Streptomyces sp. KPB2 | 579 | 634 | 899 | 247 | 1424 |
| Mucilaginibacter celer | 0 | 0 | 0 | 0 | 0 |
| Lentilitoribacter sp. Alg239-R112 | 72 | 87 | 81 | 0 | 131 |
| Streptomyces sp. CC0208 | 1398 | 742 | 1238 | 268 | 775 |
| Halomonas sp. JS92-SW72 | 0 | 0 | 0 | 0 | 0 |
| Paraflavitalea soli | 1864 | 2278 | 908 | 14 | 754 |
| Biomaibacter acetigenes | 48 | 107 | 162 | 1 | 129 |
| Hymenobacter oligotrophus | 245 | 594 | 467 | 8 | 613 |
| Arcobacter peruensis | 8 | 20 | 3 | 0 | 7 |
| Corynebacterium silvaticum | 54 | 63 | 74 | 13 | 147 |
| Paenisporosarcina cavernae | 12 | 28 | 28 | 0 | 35 |
| Pseudomonas cavernae | 681 | 1157 | 1065 | 7 | 1280 |
| Clostridium manihotivorum | 0 | 0 | 0 | 0 | 0 |
| Actinomyces lilanjuaniae | 0 | 0 | 0 | 0 | 0 |
| Chryseolinea soli | 1313 | 2060 | 1432 | 6 | 1857 |
| Serratia inhibens | 107 | 119 | 143 | 2 | 167 |
| Tsuneonella amylolytica | 0 | 0 | 0 | 0 | 0 |
| Yersinia hibernica | 13 | 56 | 64 | 12 | 94 |
| Georhizobium profundi | 1759 | 1654 | 1379 | 48 | 1969 |
| Arachidicoccus soli | 66 | 214 | 118 | 1 | 116 |
| Alteromonas sp. 76-1 | 1 | 19 | 15 | 0 | 10 |
| Tenacibaculum singaporense | 7 | 11 | 15 | 0 | 12 |
| Rhizobium sp. CCGE531 | 0 | 0 | 0 | 0 | 0 |
| Rhizobium sp. CCGE532 | 419 | 118 | 279 | 0 | 591 |
| Alistipes megaguti | 347 | 495 | 501 | 4 | 802 |
| Streptococcus koreensis | 0 | 0 | 0 | 0 | 0 |
| Nocardia yunnanensis | 1962 | 1937 | 3669 | 160 | 4435 |
| Gryllotalpicola protaetiae | 1662 | 1738 | 2311 | 33 | 2373 |
| Apilactobacillus bombintestini | 0 | 5 | 12 | 0 | 2 |
| Lactococcus allomyrinae | 16 | 14 | 14 | 0 | 14 |
| Protaetiibacter intestinalis | 1224 | 1796 | 2272 | 170 | 2730 |
| Virgibacillus sp. Bac330 | 10 | 6 | 6 | 0 | 13 |
| Virgibacillus sp. Bac332 | 2 | 42 | 18 | 0 | 29 |
| Xanthobacter sp. YC-JY1 | 2177 | 2023 | 2061 | 38 | 2656 |
| Streptomyces sp. S501 | 316 | 416 | 484 | 107 | 1003 |
| Serratia sp. FDAARGOS_506 | 0 | 0 | 0 | 0 | 0 |
| Gordonia insulae | 1751 | 1598 | 2200 | 28 | 2792 |
| Serratia sp. 1D1416 | 213 | 91 | 81 | 0 | 242 |
| Serratia sp. 3ACOL1 | 93 | 126 | 113 | 0 | 98 |
| Enterobacter oligotrophicus | 0 | 0 | 0 | 0 | 0 |
| Flavobacterium sp. 140616W15 | 0 | 0 | 0 | 0 | 0 |
| Acidovorax sp. 1608163 | 0 | 0 | 0 | 0 | 0 |
| Chryseobacterium sp. 3008163 | 6 | 580 | 398 | 1 | 460 |
| Commensalibacter sp. AMU001 | 0 | 20 | 29 | 0 | 42 |
| Buttiauxella sp. 3AFRM03 | 6 | 232 | 101 | 0 | 5058 |
| Pseudomonas sp. LTJR-52 | 0 | 0 | 0 | 0 | 0 |
| Pseudomonas sp. LTGT-11-2Z | 44 | 0 | 96 | 0 | 100 |
| Blautia sp. SC05B48 | 19 | 62 | 45 | 2 | 38 |
| Plantibacter sp. PA-3-X8 | 764 | 2001 | 3318 | 32 | 2217 |
| Pedobacter sp. G11 | 42 | 149 | 291 | 0 | 178 |
| Sphingopyxis sp. YF1 | 644 | 1385 | 774 | 12 | 1268 |
| Serratia sp. P2ACOL2 | 54 | 39 | 66 | 0 | 146 |
| Parasedimentitalea marina | 0 | 0 | 0 | 0 | 0 |
| Clostridium sp. JN-1 | 0 | 60 | 28 | 0 | 17 |
| Thermus caldilimi | 102 | 241 | 290 | 3 | 396 |
| Microbacterium sp. 10M-3C3 | 824 | 1234 | 2181 | 44 | 2336 |
| Mesorhizobium sp. NZP2234 | 1543 | 448 | 589 | 17 | 506 |
| Mesorhizobium sp. NZP2298 | 1162 | 1225 | 1206 | 32 | 1665 |
| Mesorhizobium sp. NZP2077 | 1158 | 1174 | 983 | 0 | 666 |
| Brevibacterium sp. S22 | 0 | 2 | 3 | 0 | 1 |
| Nocardioides pantholopis | 1982 | 2581 | 3692 | 95 | 2830 |
| Georgenia faecalis | 1531 | 1916 | 2889 | 65 | 3390 |
| Pectobacterium polonicum | 32 | 105 | 46 | 1 | 38 |
| Erysipelothrix piscisicarius | 0 | 15 | 4 | 0 | 4 |
| Serratia sp. LS-1 | 22 | 57 | 238 | 0 | 224 |
| Nocardioides sp. WS12 | 2178 | 2933 | 3976 | 95 | 4038 |
| Sphingopyxis sp. OPL5 | 0 | 0 | 0 | 0 | 0 |
| Rickettsiales endosymbiont of Stachyamoeba lipophora | 8 | 23 | 12 | 5 | 36 |
| Arthrobacter sulfonylureivorans | 791 | 1042 | 1197 | 56 | 1809 |
| Chryseobacterium sp. G0162 | 4 | 32 | 22 | 0 | 31 |
| Chryseobacterium sp. G0186 | 21 | 28 | 33 | 0 | 40 |
| Chryseobacterium sp. G0201 | 13 | 98 | 60 | 0 | 54 |
| Intestinibaculum porci | 30 | 30 | 35 | 0 | 38 |
| Metakosakonia sp. MRY16-398 | 0 | 0 | 0 | 0 | 43 |
| Bacillus cabrialesii | 50 | 183 | 73 | 0 | 45 |
| Mycolicibacterium nivoides | 1684 | 12283 | 2460 | 49 | 5991 |
| Shewanella psychromarinicola | 61 | 101 | 66 | 5 | 116 |
| Paenibacillus sp. M-152 | 36 | 23 | 57 | 0 | 105 |
| Coxiella endosymbiont of Amblyomma sculptum | 22 | 41 | 28 | 0 | 47 |
| Pigmentiphaga sp. H8 | 1334 | 1697 | 1889 | 31 | 2198 |
| Klebsiella sp. FDAARGOS_511 | 0 | 48 | 59 | 0 | 80 |
| Pectobacterium versatile | 140 | 122 | 164 | 19 | 149 |
| Marinobacter sp. NP-4(2019) | 286 | 495 | 446 | 20 | 379 |
| Ruminiclostridium herbifermentans | 17 | 19 | 18 | 1 | 15 |
| Corynebacterium endometrii | 181 | 230 | 349 | 9 | 541 |
| Klebsiella africana | 67 | 143 | 167 | 0 | 177 |
| Streptococcus periodonticum | 9 | 0 | 1 | 0 | 0 |
| Pseudoalteromonas sp. Xi13 | 0 | 31 | 7 | 0 | 30 |
| Pantoea sp. CCBC3-3-1 | 0 | 0 | 0 | 0 | 0 |
| Rhodococcus sp. NJ-530 | 0 | 0 | 0 | 0 | 25 |
| Staphylospora marina | 308 | 561 | 622 | 12 | 979 |
| Anabaena sp. YBS01 | 0 | 0 | 0 | 0 | 0 |
| Mycobacterium novum | 2787 | 3329 | 3954 | 105 | 6085 |
| Bradyrhizobium sp. LCT2 | 1820 | 1857 | 1411 | 11 | 1526 |
| Fluviispira sanaruensis | 49 | 40 | 57 | 1 | 52 |
| Mesorhizobium sp. M9A.F.Ca.ET.002.03.1.2 | 3171 | 2890 | 2399 | 6 | 3068 |
| Mesorhizobium sp. M1D.F.Ca.ET.043.01.1.1 | 2838 | 2543 | 2619 | 49 | 3089 |
| Mesorhizobium sp. M2A.F.Ca.ET.043.02.1.1 | 1516 | 1533 | 1251 | 18 | 1647 |
| Mesorhizobium sp. M2A.F.Ca.ET.043.05.1.1 | 1734 | 1535 | 1516 | 7 | 1858 |
| Mesorhizobium sp. M1E.F.Ca.ET.045.02.1.1 | 2880 | 2489 | 2138 | 0 | 2278 |
| Mesorhizobium sp. M1B.F.Ca.ET.045.04.1.1 | 2429 | 2585 | 2753 | 13 | 3084 |
| Mesorhizobium sp. M2A.F.Ca.ET.046.03.2.1 | 2384 | 2129 | 1677 | 0 | 2412 |
| Mesorhizobium sp. M4B.F.Ca.ET.058.02.1.1 | 2048 | 1620 | 1438 | 0 | 1204 |
| Mesorhizobium sp. M3A.F.Ca.ET.080.04.2.1 | 2233 | 1677 | 1811 | 27 | 2233 |
| Mesorhizobium sp. M6A.T.Cr.TU.016.01.1.1 | 2857 | 2539 | 2463 | 74 | 3085 |
| Mesorhizobium sp. M7D.F.Ca.US.005.01.1.1 | 2466 | 1457 | 1539 | 5 | 1855 |
| Mesorhizobium sp. M8A.F.Ca.ET.057.01.1.1 | 1540 | 1595 | 1444 | 41 | 2004 |
| Mesorhizobium sp. M1A.F.Ca.IN.022.06.1.1 | 1803 | 2058 | 1725 | 2 | 2210 |
| Mesorhizobium sp. M7A.F.Ce.TU.012.03.2.1 | 1370 | 1314 | 1267 | 65 | 1544 |
| Sutterella megalosphaeroides | 663 | 663 | 602 | 7 | 1263 |
| Flammeovirga pectinis | 6 | 15 | 14 | 0 | 16 |
| Tabrizicola piscis | 659 | 790 | 659 | 0 | 586 |
| Aequorivita sp. H23M31 | 24 | 90 | 67 | 29 | 124 |
| Chlamydia buteonis | 0 | 3 | 0 | 0 | 0 |
| Azospirillum sp. 412522 | 42 | 69 | 36 | 1 | 85 |
| Roseovarius faecimaris | 503 | 689 | 613 | 6 | 990 |
| Enterobacter chengduensis | 304 | 541 | 586 | 0 | 450 |
| Curtobacterium sp. Csp1 | 249 | 961 | 1216 | 110 | 1408 |
| Curtobacterium sp. Csp2 | 247 | 639 | 743 | 9 | 865 |
| Paenibacillus albus | 134 | 334 | 331 | 9 | 549 |
| Geobacter sp. SVR | 580 | 1436 | 1347 | 12 | 2246 |
| Actinobaculum sp. 313 | 189 | 295 | 367 | 46 | 812 |
| Hymenobacter radiodurans | 336 | 802 | 637 | 0 | 798 |
| Jeotgalibaca ciconiae | 0 | 0 | 0 | 0 | 0 |
| Iodobacter ciconiae | 62 | 131 | 92 | 0 | 235 |
| Streptomyces cyaneochromogenes | 2833 | 2080 | 4278 | 287 | 7419 |
| Brevibacillus marinus | 462 | 731 | 907 | 37 | 1295 |
| Maribacter sp. MJ134 | 52 | 41 | 22 | 1 | 34 |
| Nonlabens ponticola | 28 | 31 | 26 | 1 | 41 |
| Flaviflexus ciconiae | 0 | 0 | 0 | 0 | 0 |
| Chryseobacterium aureum | 10 | 42 | 32 | 0 | 62 |
| Cellulosilyticum sp. WCF-2 | 8 | 2 | 9 | 0 | 4 |
| Halomonas tianxiuensis | 562 | 714 | 699 | 20 | 1038 |
| Janthinobacterium sp. 17J80-10 | 678 | 974 | 859 | 3 | 1751 |
| Enterobacter chuandaensis | 256 | 198 | 176 | 0 | 138 |
| Colwellia sp. Arc7-635 | 0 | 18 | 27 | 0 | 22 |
| Jinshanibacter zhutongyuii | 0 | 0 | 0 | 0 | 0 |
| Streptomonospora litoralis | 2410 | 2068 | 3892 | 695 | 12755 |
| Agromyces sp. LHK192 | 2836 | 2787 | 4377 | 107 | 4923 |
| Pseudomonas sp. MPC6 | 379 | 618 | 587 | 20 | 1025 |
| Devosia sp. 1566 | 1119 | 1228 | 1093 | 15 | 1364 |
| Rhodococcus sp. X156 | 2606 | 1723 | 3678 | 175 | 3824 |
| Aquiluna borgnonia | 46 | 164 | 157 | 3 | 199 |
| Bacillus sp. BD59S | 2 | 18 | 42 | 0 | 17 |
| Enterobacter sp. N18-03635 | 0 | 50 | 76 | 0 | 46 |
| Pseudorhodobacter turbinis | 231 | 253 | 218 | 14 | 378 |
| Salmonella sp. SSDFZ69 | 0 | 881 | 0 | 0 | 0 |
| Nocardioides yefusunii | 1182 | 1629 | 2090 | 46 | 2017 |
| Apibacter raozihei | 14 | 23 | 31 | 0 | 36 |
| Acetobacter oryzoeni | 3 | 38 | 40 | 0 | 106 |
| Dyella sp. M7H15-1 | 0 | 0 | 0 | 0 | 0 |
| Pseudocnuella soli | 0 | 0 | 0 | 0 | 0 |
| Hymenobacter jejuensis | 184 | 843 | 569 | 0 | 721 |
| Flavobacterium cerinum | 9 | 110 | 40 | 6 | 61 |
| Salicibibacter halophilus | 74 | 104 | 95 | 0 | 183 |
| Erythrobacter sp. HKB08 | 0 | 0 | 0 | 0 | 0 |
| Pseudomonas khazarica | 131 | 213 | 163 | 0 | 296 |
| Campylobacter armoricus | 0 | 2 | 1 | 0 | 1 |
| Pseudomonas viciae | 156 | 293 | 515 | 40 | 599 |
| Periweissella cryptocerci | 11 | 48 | 82 | 0 | 53 |
| Corynebacterium suranareeae | 36 | 81 | 146 | 10 | 209 |
| Clostridium sp. JN-9 | 35 | 25 | 40 | 0 | 44 |
| Aminipila luticellarii | 32 | 62 | 49 | 0 | 62 |
| Acidilutibacter cellobiosedens | 147 | 20 | 77 | 2 | 34 |
| Caproiciproducens sp. NJN-50 | 181 | 334 | 433 | 7 | 630 |
| Muriicola soli | 28 | 61 | 100 | 0 | 73 |
| Rhodococcus sp. ABRD24 | 1442 | 1356 | 2110 | 94 | 3257 |
| Cohnella abietis | 29 | 94 | 58 | 0 | 115 |
| Luteimonas sp. YGD11-2 | 1473 | 1476 | 1444 | 24 | 1767 |
| Pandoraea commovens | 711 | 1240 | 1252 | 77 | 1688 |
| Leisingera sp. NJS201 | 0 | 0 | 0 | 0 | 0 |
| Leisingera sp. NJS204 | 137 | 376 | 315 | 19 | 319 |
| Candidatus Thioglobus sp. NP1 | 13 | 36 | 12 | 0 | 7 |
| Salinibacterium sp. UTAS2018 | 316 | 596 | 444 | 19 | 1167 |
| Ornithinimicrobium sp. HY006 | 1395 | 1523 | 2480 | 88 | 3085 |
| Sulfurimonas hydrogeniphila | 1 | 9 | 7 | 0 | 6 |
| Agromyces protaetiae | 2320 | 2029 | 3542 | 29 | 3598 |
| Paenibacillus protaetiae | 0 | 0 | 0 | 0 | 0 |
| Xylanimonas protaetiae | 1940 | 2196 | 3342 | 49 | 4090 |
| Microbacterium protaetiae | 973 | 1256 | 1901 | 62 | 2101 |
| Xylanimonas allomyrinae | 1611 | 1894 | 2683 | 119 | 3691 |
| Rhodoferax sediminis | 1015 | 1688 | 1314 | 28 | 1932 |
| Ktedonosporobacter rubrisoli | 345 | 673 | 954 | 2 | 1211 |
| Synechococcus sp. RSCCF101 | 319 | 724 | 762 | 48 | 1258 |
| Pseudolysobacter antarcticus | 1269 | 1852 | 1506 | 6 | 1798 |
| Aquirufa nivalisilvae | 25 | 44 | 46 | 0 | 72 |
| Aquirufa antheringensis | 72 | 51 | 40 | 0 | 64 |
| Campylobacter sp. CCUG 57310 | 11 | 4 | 16 | 0 | 24 |
| Psychrobacter sp. KH172YL61 | 13 | 15 | 16 | 0 | 39 |
| Lentibacillus sp. CBA3610 | 23 | 37 | 52 | 1 | 45 |
| Flavobacterium sangjuense | 0 | 0 | 0 | 0 | 0 |
| Acidovorax sp. JMULE5 | 0 | 0 | 0 | 0 | 0 |
| Nocardioides euryhalodurans | 2204 | 3165 | 4086 | 35 | 2432 |
| Nocardioides seonyuensis | 114 | 1118 | 1681 | 25 | 0 |
| Pandoraea sp. XY-2 | 290 | 765 | 808 | 10 | 1317 |
| Pseudomonas tructae | 256 | 378 | 390 | 4 | 484 |
| Oceanispirochaeta crateris | 20 | 26 | 31 | 2 | 50 |
| Duncaniella dubosii | 33 | 88 | 107 | 0 | 107 |
| Pseudoalteromonas rhizosphaerae | 0 | 0 | 0 | 0 | 0 |
| Aequoribacter fuscus | 66 | 119 | 139 | 2 | 172 |
| Spirosoma sp. KCTC 42546 | 149 | 480 | 437 | 0 | 430 |
| Shewanella maritima | 46 | 29 | 61 | 3 | 38 |
| Polynucleobacter paneuropaeus | 52 | 208 | 58 | 2 | 71 |
| Gimesia aquarii | 222 | 940 | 512 | 28 | 860 |
| Aureliella helgolandensis | 470 | 1836 | 1123 | 23 | 1806 |
| Gimesia algae | 375 | 1342 | 772 | 2 | 1249 |
| Aeoliella mucimassa | 2657 | 4748 | 3395 | 26 | 5486 |
| Gimesia alba | 228 | 1251 | 750 | 18 | 1201 |
| Tautonia plasticadhaerens | 7879 | 42700 | 19548 | 241 | 27670 |
| Gimesia fumaroli | 246 | 1247 | 737 | 28 | 1367 |
| Gimesia panareensis | 1314 | 4830 | 2928 | 93 | 4972 |
| Stieleria neptunia | 1438 | 4635 | 3232 | 75 | 5429 |
| Mucisphaera calidilacus | 905 | 2100 | 1903 | 21 | 2366 |
| Caulifigura coniformis | 6241 | 14757 | 9532 | 66 | 11236 |
| Bremerella volcania | 1772 | 6546 | 4288 | 36 | 7085 |
| Alienimonas californiensis | 2783 | 8233 | 5556 | 75 | 8418 |
| Rosistilla oblonga | 1038 | 3677 | 2254 | 8 | 4116 |
| Thalassoglobus polymorphus | 320 | 940 | 630 | 1 | 966 |
| Symmachiella dynata | 1647 | 5864 | 3665 | 46 | 6046 |
| Crateriforma conspicua | 1009 | 2257 | 1691 | 16 | 2933 |
| Polystyrenella longa | 251 | 1113 | 610 | 3 | 1083 |
| Pirellulimonas nuda | 7258 | 13133 | 10066 | 132 | 15055 |
| Lignipirellula cremea | 3478 | 11186 | 8458 | 170 | 13998 |
| Rosistilla carotiformis | 714 | 2508 | 1967 | 22 | 2754 |
| Planctopirus ephydatiae | 453 | 1755 | 987 | 20 | 1671 |
| Poriferisphaera corsica | 108 | 498 | 236 | 3 | 378 |
| Anatilimnocola aggregata | 3372 | 11852 | 9461 | 189 | 14152 |
| Botrimarina mediterranea | 6450 | 11397 | 8540 | 99 | 12662 |
| Urbifossiella limnaea | 12025 | 112854 | 46498 | 479 | 64809 |
| Lacipirellula limnantheis | 28312 | 20857 | 15608 | 375 | 16539 |
| Maioricimonas rarisocia | 3245 | 12624 | 8218 | 102 | 12837 |
| Calycomorphotria hydatis | 625 | 2728 | 1484 | 13 | 2482 |
| Eikenella exigua | 87 | 109 | 102 | 3 | 125 |
| Bacteroides sp. A1C1 | 6 | 0 | 0 | 0 | 0 |
| Zhaonella formicivorans | 38 | 113 | 132 | 2 | 176 |
| Lichenihabitans psoromatis | 1113 | 1341 | 976 | 6 | 1261 |
| Xanthobacter dioxanivorans | 6526 | 4645 | 4988 | 164 | 6156 |
| Bacillus sp. SYJ | 0 | 0 | 0 | 0 | 20 |
| Muribaculum gordoncarteri | 46 | 249 | 133 | 5 | 217 |
| Microbacterium wangchenii | 886 | 980 | 1571 | 20 | 1512 |
| Arachidicoccus sp. B3-10 | 94 | 60 | 62 | 0 | 79 |
| Rheinheimera sp. D18 | 0 | 0 | 0 | 0 | 0 |
| Acinetobacter sp. FDAARGOS_724 | 25 | 6 | 12 | 12 | 21 |
| Cedecea sp. FDAARGOS_727 | 103 | 175 | 212 | 5 | 236 |
| Pseudomonas sp. FDAARGOS_761 | 337 | 197 | 47 | 0 | 172 |
| Citrobacter arsenatis | 29 | 63 | 51 | 2 | 66 |
| Thermaerobacter sp. FW80 | 1313 | 2277 | 2604 | 31 | 3328 |
| Gloeothece citriformis | 32 | 76 | 67 | 0 | 120 |
| Gloeothece verrucosa | 16 | 120 | 89 | 2 | 86 |
| Crocosphaera subtropica | 38 | 164 | 101 | 1 | 89 |
| Rippkaea orientalis | 44 | 99 | 99 | 0 | 169 |
| Flavobacterium nackdongense | 81 | 194 | 98 | 2 | 122 |
| Paraburkholderia pallida | 1880 | 2296 | 2600 | 67 | 3846 |
| Marinobacter sp. JH2 | 93 | 169 | 142 | 9 | 174 |
| Chryseobacterium salivictor | 0 | 0 | 0 | 0 | 0 |
| Erwinia sp. QL-Z3 | 63 | 759 | 100 | 2 | 112 |
| Parashewanella tropica | 8 | 23 | 14 | 6 | 23 |
| Streptomyces aquilus | 2605 | 2131 | 4080 | 303 | 6018 |
| Thalassotalea sp. HSM 43 | 21 | 48 | 37 | 1 | 23 |
| Halomonas sp. XH26 | 24 | 166 | 94 | 0 | 142 |
| Sphingobacterium sp. CZ-2 | 0 | 0 | 0 | 0 | 0 |
| Candidatus Hydrogenosomobacter endosymbioticus | 9 | 20 | 35 | 0 | 31 |
| Oecophyllibacter saccharovorans | 188 | 268 | 248 | 4 | 356 |
| Formicincola oecophyllae | 0 | 0 | 0 | 0 | 0 |
| Nocardioides cynanchi | 2805 | 3768 | 4744 | 158 | 4773 |
| Rhodocaloribacter litoris | 2036 | 5036 | 4890 | 45 | 5236 |
| Pseudomonas sp. S150 | 0 | 223 | 402 | 191 | 1516 |
| Candidatus Nitrotoga sp. AM1P | 182 | 379 | 254 | 4 | 425 |
| Actinomyces procaprae | 497 | 582 | 836 | 47 | 1743 |
| Aeromonas sp. 1805 | 49 | 84 | 116 | 0 | 228 |
| Aeromonas sp. 2692-1 | 39 | 66 | 149 | 0 | 85 |
| Paracoccus liaowanqingii | 0 | 0 | 0 | 0 | 0 |
| Terasakiella sp. SH-1 | 0 | 0 | 0 | 0 | 0 |
| Brevundimonas sp. Bb-A | 0 | 0 | 0 | 0 | 0 |
| Candidatus Campylobacter infans | 10 | 6 | 6 | 0 | 10 |
| Brevundimonas sp. MF30-B | 0 | 0 | 0 | 0 | 0 |
| Halomonas binhaiensis | 0 | 0 | 0 | 0 | 0 |
| Pseudolabrys sp. FHR47 | 11771 | 4339 | 4705 | 206 | 8149 |
| Constantimarinum furrinae | 33 | 136 | 45 | 0 | 59 |
| Citrobacter tructae | 37 | 114 | 108 | 2 | 127 |
| Vagococcus xieshaowenii | 0 | 5 | 4 | 0 | 4 |
| Myroides albus | 16 | 30 | 12 | 0 | 22 |
| Streptomyces sp. SS52 | 925 | 758 | 1143 | 30 | 1803 |
| Treponema sp. OMZ 906 | 52 | 27 | 0 | 0 | 0 |
| Treponema sp. OMZ 788 | 0 | 0 | 3 | 0 | 1 |
| Treponema sp. OMZ 791 | 0 | 0 | 0 | 0 | 132 |
| Treponema sp. OMZ 799 | 0 | 18 | 5 | 0 | 13 |
| Treponema sp. OMZ 787 | 8 | 4 | 0 | 0 | 1 |
| Candidatus Pelagibacter giovannonii | 3 | 36 | 7 | 0 | 2 |
| Acinetobacter sp. 10FS3-1 | 0 | 0 | 0 | 0 | 0 |
| Leptospira tipperaryensis | 60 | 206 | 82 | 1 | 85 |
| Ruminococcus bovis | 4 | 11 | 3 | 0 | 7 |
| Arthrobacter sp. PAMC25564 | 600 | 1476 | 2560 | 85 | 4119 |
| Psychrobacter sp. PraFG1 | 0 | 12 | 16 | 0 | 17 |
| Sphingomonas sp. PAMC26645 | 1095 | 4500 | 3621 | 2 | 4258 |
| Sphingopyxis sp. PAMC25046 | 790 | 1192 | 665 | 0 | 722 |
| Hydrogenophaga sp. PAMC20947 | 480 | 710 | 737 | 24 | 1060 |
| Rhodococcus sp. PAMC28707 | 0 | 1993 | 0 | 0 | 2069 |
| Luteimonas yindakuii | 1468 | 1278 | 1290 | 3 | 1697 |
| Enterobacter sp. 18A13 | 0 | 24 | 48 | 0 | 14 |
| Paenibacillus algicola | 49 | 280 | 184 | 1 | 248 |
| Cellulomonas shaoxiangyii | 1968 | 2093 | 2913 | 55 | 3324 |
| Citricoccus sp. SGAir0253 | 666 | 833 | 1427 | 0 | 696 |
| Rhodococcus sp. SGAir0479 | 2439 | 2005 | 3743 | 287 | 4848 |
| Polyangium aurulentum | 5855 | 13638 | 15214 | 198 | 13737 |
| Microbacterium sp. 4R-513 | 1317 | 1689 | 2506 | 64 | 3071 |
| Pedobacter sp. BS3 | 7 | 72 | 32 | 1 | 45 |
| Metabacillus sediminilitoris | 0 | 0 | 0 | 0 | 0 |
| Desulfosediminicola ganghwensis | 51 | 201 | 275 | 6 | 411 |
| Desulfosediminicola flagellatus | 30 | 101 | 63 | 0 | 120 |
| Thauera sp. 2A1 | 706 | 279 | 629 | 0 | 373 |
| Phreatobacter sp. NMCR1094 | 4812 | 2542 | 2248 | 55 | 3045 |
| Psychroserpens sp. NJDZ02 | 35 | 25 | 22 | 1 | 18 |
| Brachybacterium sp. SGAir0954 | 0 | 0 | 0 | 0 | 0 |
| Pontibacter sp. SGAir0037 | 0 | 0 | 0 | 0 | 0 |
| Pseudoxanthomonas sp. X-1 | 3122 | 3052 | 3172 | 3 | 3492 |
| Thermosipho ferrireducens | 2 | 116 | 41 | 0 | 33 |
| Leuconostoc sp. LN180020 | 2 | 0 | 6 | 0 | 0 |
| Burkholderia sp. DHOD12 | 1189 | 1772 | 2021 | 39 | 1818 |
| Paraburkholderia acidiphila | 1193 | 1840 | 1914 | 0 | 1998 |
| Paraburkholderia acidisoli | 1134 | 1902 | 1757 | 0 | 2224 |
| Novosphingobium sp. EMRT-2 | 0 | 0 | 0 | 0 | 0 |
| Vagococcus zengguangii | 2 | 60 | 15 | 0 | 5 |
| Candidatus Nanosynbacter featherlites | 64 | 33 | 31 | 0 | 66 |
| Nostoc sp. TCL240-02 | 43 | 93 | 90 | 4 | 105 |
| Salinimonas iocasae | 49 | 223 | 67 | 1 | 76 |
| Myroides fluvii | 24 | 43 | 34 | 0 | 25 |
| Vibrio taketomensis | 34 | 28 | 38 | 16 | 25 |
| Shewanella sp. MEBiC00475 | 16 | 15 | 4 | 15 | 0 |
| Arthrobacter sp. 24S4-2 | 1166 | 2875 | 5399 | 30 | 13370 |
| Pantoea sp. SO10 | 0 | 0 | 0 | 0 | 0 |
| Nocardioides jishulii | 1516 | 2103 | 2470 | 222 | 3314 |
| Elizabethkingia sp. 2-6 | 0 | 0 | 0 | 0 | 0 |
| Brevibacterium sp. CS2 | 593 | 547 | 1443 | 0 | 0 |
| Caloramator sp. E03 | 0 | 0 | 0 | 0 | 0 |
| Streptococcus sp. 1643 | 0 | 0 | 6 | 0 | 2 |
| Caproicibacter fermentans | 122 | 328 | 222 | 2 | 297 |
| Pseudarthrobacter sp. NamE5 | 2 | 6 | 21 | 0 | 35 |
| Nibricoccus aquaticus | 923 | 2729 | 2142 | 9 | 2611 |
| Nostoc sp. C052 | 0 | 0 | 0 | 0 | 0 |
| Nostoc sp. C057 | 65 | 212 | 287 | 0 | 501 |
| Nostoc sp. TCL26-01 | 20 | 344 | 256 | 11 | 472 |
| Polynucleobacter sp. AP-Nino-20-G2 | 70 | 147 | 51 | 4 | 61 |
| Polynucleobacter sp. JS-JIR-II-50 | 12 | 38 | 15 | 3 | 48 |
| Polynucleobacter sp. JS-Mosq-20-D10 | 8 | 24 | 26 | 0 | 43 |
| Polynucleobacter sp. AP-Titi-500A-B4 | 8 | 29 | 30 | 0 | 38 |
| Polynucleobacter sp. AP-Ainpum-60-G11 | 19 | 30 | 35 | 6 | 113 |
| Polynucleobacter sp. AP-Jannik-300A-C4 | 54 | 53 | 17 | 2 | 25 |
| Polynucleobacter sp. AP-Elch-400A-B2 | 1 | 41 | 28 | 1 | 31 |
| Polynucleobacter sp. MG-6-Vaara-E2 | 2 | 23 | 24 | 1 | 32 |
| Pedobacter sp. KBS0701 | 82 | 200 | 310 | 0 | 303 |
| Arthrobacter sp. KBS0702 | 920 | 1346 | 2319 | 67 | 3147 |
| Pseudomonas sp. KBS0707 | 0 | 73 | 90 | 0 | 110 |
| Bradyrhizobium sp. KBS0725 | 0 | 37031 | 0 | 0 | 0 |
| Bradyrhizobium sp. KBS0727 | 7944 | 0 | 0 | 0 | 5740 |
| Agrobacterium sp. CGMCC 11546 | 136 | 150 | 175 | 0 | 189 |
| Nautilia sp. PV-1 | 0 | 104 | 19 | 0 | 33 |
| Synechococcus sp. PCC 11901 | 12 | 92 | 138 | 0 | 105 |
| Jejubacter calystegiae | 0 | 0 | 0 | 0 | 0 |
| Paroceanicella profunda | 2225 | 2653 | 2616 | 26 | 3831 |
| Thiomicrorhabdus sediminis | 73 | 39 | 48 | 23 | 54 |
| Agrobacterium sp. T29 | 473 | 348 | 297 | 12 | 405 |
| Dialister hominis | 0 | 52 | 0 | 0 | 0 |
| Enterococcus sp. M190262 | 0 | 0 | 0 | 0 | 17 |
| Streptomyces sp. SUK 48 | 1226 | 1190 | 1846 | 63 | 2989 |
| Nocardioides sp. S-1144 | 2403 | 4241 | 5315 | 81 | 4356 |
| Luteithermobacter gelatinilyticus | 0 | 0 | 0 | 0 | 0 |
| Alcaligenes ammonioxydans | 0 | 0 | 0 | 0 | 0 |
| Rhizobium indicum | 1141 | 1169 | 887 | 8 | 787 |
| Micromonospora sp. HM134 | 2515 | 1952 | 5999 | 263 | 6773 |
| Saccharibacillus brassicae | 495 | 859 | 959 | 7 | 1082 |
| Amedibacterium intestinale | 20 | 21 | 83 | 0 | 61 |
| Thermosynechococcus sp. CL-1 | 9 | 74 | 88 | 0 | 72 |
| Martelella lutilitoris | 1456 | 995 | 1017 | 8 | 1649 |
| Aggregatimonas sangjinii | 47 | 55 | 82 | 0 | 90 |
| Colwellia sp. M166 | 26 | 238 | 30 | 0 | 61 |
| Plantibacter sp. M259 | 533 | 1858 | 2021 | 55 | 2108 |
| Salidesulfovibrio onnuriiensis | 347 | 756 | 808 | 14 | 1019 |
| Elizabethkingia sp. JS20170427COW | 13 | 36 | 15 | 0 | 15 |
| Staphylococcus sp. 17KM0847 | 0 | 17 | 20 | 0 | 5 |
| Aureibaculum algae | 22 | 20 | 14 | 0 | 20 |
| Mesorhizobium sp. 8 | 2597 | 2889 | 2897 | 58 | 3875 |
| Sutterella faecalis | 221 | 419 | 478 | 13 | 870 |
| Alistipes communis | 540 | 1204 | 945 | 10 | 1248 |
| Alistipes dispar | 322 | 636 | 557 | 15 | 712 |
| Georgenia wutianyii | 1620 | 2031 | 2945 | 135 | 4338 |
| Streptomyces sp. C8S0 | 1885 | 1693 | 2949 | 199 | 4741 |
| Agromyces laixinhei | 2454 | 2223 | 4474 | 20 | 4631 |
| Antarcticibacterium arcticum | 11 | 44 | 63 | 0 | 54 |
| Oceanicola sp. D3 | 576 | 610 | 801 | 9 | 1095 |
| Klebsiella pasteurii | 27 | 34 | 10 | 0 | 58 |
| Pseudomonas sp. SWI7 | 85 | 43 | 89 | 0 | 170 |
| Cellulosimicrobium protaetiae | 1583 | 1623 | 2475 | 51 | 2952 |
| Maribius sp. THAF1 | 0 | 0 | 0 | 0 | 0 |
| Pseudoalteromonas sp. THAF3 | 15 | 13 | 27 | 0 | 11 |
| Halomonas sp. THAF5a | 657 | 1045 | 1222 | 20 | 1712 |
| Roseovarius sp. THAF8 | 552 | 558 | 514 | 5 | 683 |
| Roseovarius sp. THAF9 | 729 | 709 | 676 | 12 | 950 |
| Bacillus sp. THAF10 | 88 | 101 | 32 | 0 | 27 |
| Halomonas sp. THAF12 | 483 | 934 | 1152 | 18 | 1022 |
| Roseovarius sp. THAF27 | 0 | 0 | 0 | 0 | 0 |
| Roseivivax sp. THAF30 | 962 | 775 | 890 | 28 | 1156 |
| Ruegeria sp. THAF33 | 0 | 0 | 0 | 0 | 0 |
| Sulfitobacter sp. THAF37 | 947 | 1011 | 908 | 47 | 1455 |
| Microbulbifer sp. THAF38 | 82 | 144 | 157 | 0 | 233 |
| Roseivivax sp. THAF40 | 431 | 309 | 313 | 0 | 316 |
| Vibrio sp. THAF64 | 0 | 0 | 873 | 0 | 0 |
| Vibrio aquimaris | 51 | 17 | 14 | 15 | 65 |
| Pseudomonas sp. THAF187a | 0 | 0 | 10991 | 0 | 0 |
| Vibrio sp. THAF190c | 22 | 29 | 31 | 0 | 49 |
| Roseivivax sp. THAF197b | 55 | 339 | 397 | 0 | 469 |
| Shewanella polaris | 4 | 4 | 4 | 0 | 13 |
| Methylophilus medardicus | 64 | 174 | 125 | 4 | 163 |
| Candidatus Methylopumilus rimovensis | 1 | 42 | 43 | 0 | 47 |
| Candidatus Methylopumilus universalis | 45 | 65 | 45 | 1 | 79 |
| Pelagovum pacificum | 1096 | 1361 | 1547 | 33 | 1971 |
| Curtobacterium sp. csp3 | 1702 | 418 | 278 | 0 | 934 |
| Nocardioides sambongensis | 2675 | 3621 | 4576 | 200 | 5258 |
| Luteibacter pinisoli | 1287 | 1498 | 1336 | 34 | 1352 |
| Shewanella sp. LC6 | 0 | 22 | 0 | 0 | 59 |
| Georgenia yuyongxinii | 1920 | 2163 | 3376 | 104 | 3976 |
| Bacillus sp. Cs-700 | 4 | 29 | 20 | 0 | 13 |
| Mesorhizobium sp. B4-1-4 | 1736 | 1582 | 1504 | 17 | 1875 |
| Mesorhizobium sp. B2-8-5 | 1798 | 1618 | 1634 | 0 | 1616 |
| Mesorhizobium sp. B2-1-8 | 1505 | 1661 | 1341 | 0 | 1595 |
| Mesorhizobium sp. B2-1-1 | 1692 | 1358 | 1143 | 0 | 702 |
| Mesorhizobium sp. B1-1-8 | 2266 | 2070 | 2021 | 5 | 2602 |
| Francisella sp. Scap27 | 0 | 3 | 3 | 0 | 2 |
| Shewanella sp. Scap07 | 27 | 33 | 39 | 1 | 45 |
| Vibrio sp. Scap24 | 12 | 115 | 19 | 0 | 23 |
| Lysinibacillus agricola | 0 | 0 | 0 | 0 | 0 |
| Shewanella sp. SNU WT4 | 12 | 62 | 46 | 30 | 40 |
| Labrenzia sp. PHM005 | 748 | 593 | 528 | 0 | 634 |
| Sulfurimonas sediminis | 3 | 15 | 2 | 0 | 27 |
| Sulfurimonas xiamenensis | 10 | 0 | 14 | 0 | 5 |
| Sulfurimonas lithotrophica | 0 | 7 | 7 | 0 | 10 |
| Sulfurimonas marina | 8 | 4 | 8 | 0 | 7 |
| Bradyrhizobium sp. I71 | 2434 | 1591 | 1569 | 0 | 100 |
| Pseudarthrobacter sp. NIBRBAC000502771 | 236 | 931 | 1316 | 35 | 1887 |
| Pseudarthrobacter sp. NIBRBAC000502772 | 955 | 5860 | 5227 | 49 | 7775 |
| Pseudomonas sp. NIBRBAC000502773 | 133 | 210 | 243 | 4 | 670 |
| Rhizobium sp. NIBRBAC000502774 | 364 | 468 | 313 | 4 | 411 |
| Pseudarthrobacter sp. NIBRBAC000502770 | 327 | 879 | 1093 | 115 | 2219 |
| Janthinobacterium tructae | 177 | 407 | 328 | 0 | 3546 |
| Actinomadura sp. WMMA1423 | 4602 | 3959 | 8678 | 1262 | 23784 |
| Casimicrobium huifangae | 1783 | 2641 | 2735 | 11 | 4222 |
| Cellulomonas sp. Y8 | 1425 | 1970 | 2947 | 0 | 1500 |
| Brevundimonas sp. M20 | 0 | 0 | 0 | 0 | 0 |
| Microbulbifer sp. GL-2 | 71 | 128 | 119 | 11 | 200 |
| Lysobacter alkalisoli | 1381 | 1371 | 1328 | 0 | 1301 |
| Echinicola soli | 58 | 68 | 43 | 1 | 88 |
| Pontibacter sp. XAAS-72 | 0 | 0 | 0 | 0 | 0 |
| Nocardioides sp. JQ2195 | 2066 | 2862 | 3424 | 60 | 3026 |
| Olivibacter sp. LS-1 | 23 | 138 | 123 | 0 | 260 |
| Paenalkalicoccus suaedae | 92 | 125 | 94 | 0 | 64 |
| Brucella sp. 2280 | 25 | 46 | 58 | 0 | 113 |
| Agromyces intestinalis | 2584 | 2379 | 4358 | 89 | 4982 |
| Lactococcus protaetiae | 0 | 10 | 5 | 0 | 20 |
| Protaetiibacter larvae | 885 | 1534 | 1755 | 78 | 2305 |
| Acetobacter vaccinii | 181 | 231 | 205 | 17 | 386 |
| Tardiphaga sp. vice154 | 2129 | 903 | 963 | 7 | 955 |
| Tardiphaga sp. vice278 | 1363 | 3357 | 2408 | 198 | 4733 |
| Tardiphaga sp. vice352 | 3062 | 576 | 790 | 0 | 282 |
| Tardiphaga sp. vice304 | 139 | 306 | 387 | 0 | 542 |
| Mangrovibacillus cuniculi | 0 | 0 | 0 | 0 | 0 |
| Kaustia mangrovi | 4532 | 3446 | 3659 | 61 | 5401 |
| Shewanella psychropiezotolerans | 27 | 64 | 51 | 2 | 79 |
| Arcobacter sp. FWKO B | 11 | 5 | 12 | 0 | 5 |
| Ornithinimicrobium pratense | 578 | 998 | 1537 | 120 | 3094 |
| Ferrovibrio terrae | 3161 | 2268 | 2303 | 38 | 3632 |
| Formosa sediminum | 12 | 52 | 19 | 0 | 22 |
| Thalassotalea sp. PS06 | 17 | 54 | 47 | 0 | 108 |
| Rhodococcus sp. WB9 | 928 | 1081 | 1693 | 5 | 2249 |
| Ornithinimicrobium ciconiae | 917 | 1400 | 2049 | 58 | 2850 |
| Chryseobacterium sp. SNU WT5 | 13 | 27 | 23 | 0 | 44 |
| Streptomyces sp. RLB1-8 | 0 | 11502 | 0 | 0 | 0 |
| Streptomyces sp. RLB1-9 | 281 | 636 | 286 | 23 | 921 |
| Streptomyces sp. RLB3-17 | 1161 | 931 | 1656 | 23 | 1543 |
| Streptomyces sp. RLB3-5 | 5308 | 0 | 1117 | 1110 | 33484 |
| Streptomyces sp. RLB3-6 | 1619 | 1032 | 1639 | 40 | 2033 |
| Streptomyces sp. S1A1-7 | 1399 | 922 | 1543 | 13 | 1487 |
| Streptomyces sp. S1A1-8 | 3485 | 3272 | 0 | 0 | 4286 |
| Streptomyces sp. S1D4-14 | 1100 | 659 | 906 | 0 | 755 |
| Streptomyces sp. S1D4-20 | 80 | 515 | 768 | 190 | 1679 |
| Streptomyces sp. S1D4-23 | 1962 | 747 | 1425 | 979 | 8736 |
| Sphingomonas xanthus | 1372 | 989 | 581 | 16 | 988 |
| Lactobacillus sp. PV012 | 0 | 0 | 7 | 0 | 0 |
| Lactobacillus sp. PV034 | 4 | 4 | 2 | 0 | 5 |
| Chitinimonas arctica | 705 | 922 | 1019 | 15 | 1213 |
| Radiobacillus deserti | 20 | 16 | 11 | 0 | 12 |
| Microlunatus elymi | 1734 | 2220 | 3215 | 170 | 5522 |
| Candidatus Uabimicrobium amorphum | 81 | 339 | 134 | 22 | 282 |
| Tolypothrix sp. PCC 7712 | 0 | 105 | 91 | 0 | 194 |
| Pseudarthrobacter sp. NBSH8 | 581 | 2224 | 2152 | 24 | 3757 |
| Glaciihabitans sp. INWT7 | 681 | 1462 | 1291 | 122 | 1943 |
| Sphingomonas sp. NBWT7 | 1005 | 1302 | 1087 | 0 | 874 |
| Hymenobacter sp. NBH84 | 0 | 0 | 0 | 0 | 0 |
| Frigoribacterium sp. NBH87 | 1073 | 1654 | 2168 | 102 | 2417 |
| Enterobacter sp. E76 | 6 | 185 | 347 | 33 | 224 |
| Bradyrhizobium sp. SEMIA | 598 | 913 | 934 | 0 | 960 |
| Thalassolituus sp. C2-1 | 0 | 0 | 0 | 0 | 0 |
| Gordonia zhaorongruii | 841 | 812 | 1204 | 8 | 1370 |
| Tomitella fengzijianii | 1614 | 1730 | 2512 | 183 | 3427 |
| Comamonas sp. NLF-7-7 | 587 | 1261 | 1254 | 39 | 1877 |
| Serratia rhizosphaerae | 59 | 89 | 89 | 17 | 195 |
| Geobacter sp. FeAm09 | 826 | 1926 | 1728 | 29 | 2154 |
| Pseudomonas sp. BJP69 | 0 | 0 | 0 | 0 | 0 |
| Streptococcus sp. 116-D4 | 0 | 0 | 0 | 0 | 0 |
| Streptococcus sp. KS 6 | 0 | 2 | 0 | 1 | 0 |
| Cohnella cholangitidis | 171 | 402 | 418 | 4 | 473 |
| Limnoglobus roseus | 7419 | 55601 | 26448 | 134 | 42925 |
| Alkalihalobacillus miscanthi | 0 | 12 | 18 | 0 | 37 |
| Humibacter ginsenosidimutans | 962 | 1485 | 2219 | 53 | 2458 |
| Qingshengfaniella alkalisoli | 0 | 0 | 0 | 0 | 0 |
| Sphingomonas suaedae | 623 | 879 | 704 | 13 | 963 |
| Sneathiella aquimaris | 67 | 164 | 121 | 0 | 122 |
| Stenotrophomonas sp. SBJS02 | 368 | 311 | 155 | 0 | 232 |
| Streptomyces qinzhouensis | 1397 | 1335 | 2448 | 460 | 4584 |
| Pseudomonas eucalypticola | 0 | 0 | 0 | 0 | 0 |
| Nitratireductor sp. SY7 | 2912 | 2675 | 2678 | 31 | 2880 |
| Bosea sp. F3-2 | 4086 | 3899 | 3298 | 44 | 4314 |
| Corynebacterium sp. sy039 | 8 | 22 | 63 | 1 | 79 |
| Bradyrhizobium sp. TM102 | 1562 | 1312 | 1568 | 28 | 1603 |
| Francisella salimarina | 0 | 1 | 0 | 0 | 2 |
| Arthrobacter sp. UKPF54-2 | 609 | 1235 | 2355 | 36 | 3814 |
| Microvenator marinus | 264 | 797 | 723 | 15 | 835 |
| Nesterenkonia sp. NBAIMH1 | 423 | 538 | 655 | 49 | 1316 |
| Acinetobacter sp. YH12138 | 4 | 12 | 15 | 0 | 7 |
| Candidatus Cytomitobacter indipagum | 9 | 35 | 27 | 0 | 23 |
| Candidatus Sneabacter namystus | 49 | 8 | 16 | 0 | 22 |
| Candidatus Nesciobacter abundans | 6 | 7 | 4 | 0 | 12 |
| Tepidiforma bonchosmolovskayae | 1337 | 2160 | 2795 | 15 | 2969 |
| Halioglobus maricola | 0 | 0 | 0 | 0 | 0 |
| Noviherbaspirillum sp. UKPF54 | 757 | 1876 | 1436 | 0 | 2246 |
| Hypericibacter terrae | 4463 | 4446 | 5055 | 54 | 10768 |
| Hypericibacter adhaerens | 5407 | 5538 | 6606 | 118 | 13092 |
| Granulicella sp. WH15 | 1570 | 4346 | 3965 | 16 | 3511 |
| Flavobacterium alkalisoli | 18 | 75 | 53 | 0 | 54 |
| Ruania zhangjianzhongii | 1385 | 1168 | 2234 | 71 | 2657 |
| Marinobacter fonticola | 292 | 487 | 494 | 4 | 528 |
| Methylobacterium sp. WL1 | 1663 | 2000 | 1860 | 45 | 2090 |
| Rhizobium sp. WL3 | 981 | 959 | 870 | 15 | 888 |
| Salinibacterium sp. dk2585 | 611 | 1257 | 1339 | 46 | 2051 |
| Microbacterium sp. CBA3102 | 0 | 0 | 0 | 0 | 0 |
| Paraburkholderia dioscoreae | 866 | 1004 | 968 | 43 | 1744 |
| Altererythrobacter sp. BO-6 | 0 | 0 | 0 | 0 | 0 |
| Pseudomonas lalkuanensis | 786 | 925 | 896 | 0 | 1020 |
| Pseudomonas sp. C27(2019) | 0 | 0 | 3419 | 11815 | 1986 |
| Pseudomonas sp. J380 | 64 | 118 | 119 | 0 | 320 |
| Streptomyces sp. BSE6.1 | 887 | 621 | 780 | 27 | 1267 |
| Pedobacter aquae | 23 | 66 | 69 | 0 | 70 |
| Chromobacterium paludis | 499 | 591 | 705 | 31 | 1001 |
| Pukyongiella litopenaei | 856 | 1105 | 1114 | 12 | 1899 |
| Gimesia chilikensis | 912 | 4195 | 2119 | 34 | 3940 |
| Alicyclobacillus sp. TC | 49 | 77 | 65 | 0 | 55 |
| Microbacterium sp. 1S1 | 0 | 0 | 0 | 0 | 0 |
| Sodaliphilus pleomorphus | 213 | 442 | 404 | 5 | 667 |
| Rhizobium flavescens | 0 | 0 | 0 | 0 | 1 |
| Hymenobacter busanensis | 435 | 815 | 764 | 25 | 1307 |
| Yersinia canariae | 9 | 30 | 55 | 19 | 60 |
| Streptomyces sp. INR7 | 1594 | 1394 | 3017 | 299 | 6916 |
| Paenibacillus sp. 37 | 16 | 45 | 0 | 0 | 78 |
| Borrelia sp. A-FGy1 | 1 | 0 | 1 | 0 | 2 |
| Nonlabens sp. Ci31 | 1 | 13 | 21 | 3 | 49 |
| Cellulomonas palmilytica | 1886 | 1720 | 2751 | 70 | 2819 |
| Methylomonas rhizoryzae | 269 | 610 | 402 | 9 | 620 |
| Gimesia benthica | 388 | 1706 | 1130 | 1 | 1865 |
| Cupriavidus cauae | 552 | 1170 | 1353 | 0 | 1187 |
| Rathayibacter sp. VKM Ac-2754 | 0 | 0 | 31 | 0 | 19 |
| Rathayibacter sp. VKM Ac-2759 | 1236 | 1723 | 2896 | 46 | 3114 |
| Rathayibacter sp. VKM Ac-2760 | 1510 | 1607 | 2575 | 120 | 3029 |
| Rathayibacter sp. VKM Ac-2762 | 402 | 692 | 922 | 51 | 1430 |
| Rathayibacter sp. VKM Ac-2801 | 720 | 895 | 1252 | 10 | 1318 |
| Rathayibacter sp. VKM Ac-2803 | 42 | 19 | 131 | 0 | 60 |
| Rathayibacter sp. VKM Ac-2804 | 1049 | 1482 | 2546 | 88 | 2424 |
| Rathayibacter sp. VKM Ac-2805 | 770 | 700 | 1273 | 4 | 1273 |
| Cobetia sp. cqz5-12 | 0 | 0 | 0 | 0 | 0 |
| Pseudomonas sp. KUIN-1 | 8 | 40 | 112 | 0 | 57 |
| Halomonas piezotolerans | 321 | 423 | 383 | 1 | 721 |
| Acinetobacter sp. C16S1 | 10 | 12 | 20 | 0 | 5 |
| Streptomyces sp. LBUM 1475 | 1827 | 1287 | 3641 | 216 | 9371 |
| Streptomyces sp. LBUM 1480 | 2640 | 1858 | 4267 | 167 | 8069 |
| Streptomyces sp. LBUM 1482 | 4704 | 1537 | 8722 | 0 | 4342 |
| Flintibacter sp. KGMB00164 | 141 | 282 | 308 | 6 | 423 |
| Streptococcus sp. LPB0220 | 0 | 0 | 0 | 0 | 0 |
| Anoxybacillus sediminis | 328 | 1023 | 786 | 2 | 1079 |
| Rhizobium sp. BG4 | 1426 | 1064 | 1005 | 30 | 1418 |
| Rhizobium sp. BG6 | 0 | 0 | 272 | 0 | 126 |
| Sinorhizobium sp. BG8 | 1440 | 1557 | 1409 | 18 | 2234 |
| Pseudopuniceibacterium antarcticum | 0 | 0 | 0 | 0 | 0 |
| Flavobacterium sp. LPB0248 | 10 | 162 | 185 | 0 | 73 |
| Pseudomonas sp. LPB0260 | 327 | 670 | 593 | 37 | 1075 |
| Microbacterium caowuchunii | 973 | 1050 | 1715 | 48 | 2204 |
| Microbacterium lushaniae | 685 | 1038 | 1930 | 74 | 2029 |
| Nitrincola iocasae | 0 | 0 | 0 | 0 | 0 |
| Acidithiobacillus sp. 'AMD consortium' | 80 | 87 | 253 | 0 | 160 |
| Hymenobacter baengnokdamensis | 0 | 0 | 0 | 0 | 0 |
| Burkholderia perseverans | 928 | 821 | 1299 | 16 | 1456 |
| Bacteroides zhangwenhongii | 0 | 0 | 0 | 0 | 0 |
| Bacteroides luhongzhouii | 18 | 42 | 24 | 11 | 34 |
| Lacipirellula parvula | 30647 | 18225 | 16630 | 414 | 16233 |
| Pseudomonas sp. CFA | 91 | 116 | 93 | 0 | 73 |
| Arthrobacter sp. CDRTa11 | 546 | 1597 | 2108 | 0 | 4433 |
| Pradoshia sp. D12 | 5 | 30 | 18 | 0 | 3 |
| Microvirga thermotolerans | 2628 | 2190 | 2375 | 25 | 2400 |
| Mycoavidus sp. B2-EB | 11 | 110 | 63 | 0 | 104 |
| Hydrogenophaga sp. BPS33 | 2741 | 4292 | 4769 | 84 | 6506 |
| Pelistega ratti | 2 | 24 | 17 | 5 | 14 |
| Deinococcus sp. AJ005 | 430 | 714 | 686 | 7 | 743 |
| Agarivorans sp. B2Z047 | 26 | 35 | 9 | 2 | 18 |
| Candidatus Azoamicus ciliaticola | 0 | 2 | 3 | 2 | 6 |
| Chroococcidiopsis sp. CCNUC1 | 0 | 0 | 0 | 0 | 0 |
| Streptomyces phaeolivaceus | 2724 | 1666 | 3173 | 165 | 5581 |
| Sphingomonas sp. CL5.1 | 1470 | 1230 | 1070 | 0 | 1101 |
| Erythrobacter sp. 3-20A1M | 895 | 1021 | 780 | 0 | 1297 |
| Rubrobacter tropicus | 2107 | 3759 | 4344 | 44 | 5317 |
| Rubrobacter marinus | 2126 | 2925 | 4313 | 61 | 5175 |
| Pseudomonas sp. SCB32 | 425 | 699 | 638 | 26 | 956 |
| Amycolatopsis sp. YIM 10 | 22094 | 4350 | 17974 | 278 | 13107 |
| Cellulomonas sp. JZ18 | 1701 | 2051 | 3173 | 55 | 3629 |
| Dechloromonas sp. TW-R-39-2 | 299 | 429 | 472 | 3 | 573 |
| Pseudomonas sp. DTU12.1 | 166 | 180 | 67 | 0 | 459 |
| Mesorhizobium sp. INR15 | 1903 | 1759 | 1704 | 47 | 2012 |
| Mesorhizobium sp. NBSH29 | 1130 | 948 | 795 | 0 | 1084 |
| Paracoccus sp. SMMA_5 | 0 | 0 | 0 | 0 | 746 |
| Methylocystis sp. MJC1 | 1873 | 1497 | 1452 | 27 | 1960 |
| Flavobacterium sp. xlx-214 | 6 | 43 | 30 | 0 | 66 |
| Pseudactinotalea sp. HY158 | 1171 | 1487 | 2077 | 41 | 2254 |
| Flagellimonas sp. CMM7 | 10 | 37 | 29 | 6 | 86 |
| Flavobacterium panici | 0 | 0 | 278 | 0 | 0 |
| Paraburkholderia atlantica | 1056 | 1280 | 1441 | 11 | 1896 |
| Apibacter sp. B3706 | 0 | 13 | 16 | 0 | 0 |
| Apibacter sp. B2966 | 19 | 0 | 6 | 0 | 0 |
| Devosia beringensis | 1562 | 1382 | 1236 | 4 | 1806 |
| Paenibacillus sp. B01 | 862 | 1878 | 1562 | 15 | 2047 |
| Cobetia sp. AM6 | 252 | 261 | 392 | 0 | 388 |
| Candidatus Profftella armatura (Diaphorina cf. continua) | 0 | 10 | 3 | 0 | 3 |
| Candidatus Carsonella ruddii (Diaphorina cf. continua) | 0 | 0 | 0 | 0 | 2 |
| Venatorbacter cucullus | 60 | 233 | 259 | 9 | 287 |
| Vibrio sp. THAF191c | 0 | 0 | 0 | 0 | 419 |
| Aeromicrobium yanjiei | 7286 | 12532 | 10060 | 147 | 5875 |
| Pseudomonas sp. NY5710 | 150 | 213 | 306 | 0 | 306 |
| Pseudomonas sp. 13159349 | 0 | 0 | 108 | 0 | 489 |
| Streptomyces sp. SYP-A7193 | 1536 | 1038 | 1955 | 125 | 3284 |
| Raineyella fluvialis | 924 | 1302 | 2017 | 79 | 1775 |
| Vibrio algicola | 13 | 110 | 49 | 1 | 71 |
| Nocardioides sp. dk884 | 2208 | 3406 | 4571 | 200 | 5044 |
| Acinetobacter wanghuae | 0 | 0 | 0 | 0 | 0 |
| Alistipes sp. dk3624 | 231 | 433 | 363 | 3 | 618 |
| Sphingobacterium sp. dk4302 | 0 | 0 | 0 | 0 | 0 |
| Streptomyces fagopyri | 1840 | 1485 | 2851 | 78 | 4056 |
| Legionella sp. MW5194 | 174 | 215 | 126 | 10 | 149 |
| Gracilibacillus salitolerans | 38 | 19 | 18 | 0 | 32 |
| Rhodococcus sp. WAY2 | 1551 | 1716 | 2474 | 136 | 3397 |
| Serratia sp. HRI | 0 | 145 | 102 | 0 | 125 |
| Nocardioides sp. zg-579 | 3102 | 4864 | 5818 | 255 | 5751 |
| Aeromicrobium sp. zg-629 | 3815 | 7162 | 6105 | 87 | 3660 |
| Streptococcus sp. zg-86 | 8 | 2 | 0 | 0 | 0 |
| Agrobacterium sp. MA01 | 0 | 0 | 0 | 0 | 0 |
| Pseudomonas sp. CFSAN084952 | 4 | 70 | 62 | 0 | 217 |
| Candidatus Mycosynbacter amalyticus | 14 | 43 | 44 | 0 | 73 |
| Bacillus sp. AM1(2019) | 0 | 18 | 0 | 0 | 0 |
| Allosaccharopolyspora coralli | 4265 | 1697 | 4929 | 148 | 4553 |
| Gordonia mangrovi | 1119 | 1531 | 2116 | 59 | 3447 |
| Flavobacterium sp. SLB02 | 23 | 910 | 301 | 0 | 211 |
| Alicyclobacillus sp. SO9 | 42 | 230 | 135 | 11 | 192 |
| Spirosoma endbachense | 280 | 505 | 442 | 1 | 514 |
| Neisseria brasiliensis | 83 | 173 | 97 | 3 | 170 |
| Bacillus sp. N3536 | 46 | 10 | 0 | 0 | 3 |
| Desulfovibrio sp. 86 | 241 | 375 | 389 | 33 | 403 |
| Caproicibacterium lactatifermentans | 59 | 110 | 59 | 0 | 83 |
| Aminobacter sp. MDW-2 | 2688 | 2262 | 2664 | 11 | 4138 |
| Pseudomonas juntendi | 447 | 434 | 496 | 72 | 1574 |
| Spiribacter sp. 2438 | 581 | 741 | 693 | 10 | 890 |
| Marinobacterium sp. LSUCC0821 | 13 | 37 | 43 | 3 | 67 |
| Bacillus aquiflavi | 0 | 0 | 0 | 0 | 0 |
| Microlunatus sp. Gsoil 973 | 1288 | 1619 | 2782 | 138 | 3485 |
| Sulfurimonas sp. H1576 | 16 | 10 | 4 | 0 | 11 |
| Cohnella candidum | 473 | 983 | 1051 | 4 | 1533 |
| Tellurirhabdus rosea | 563 | 1296 | 1028 | 38 | 1604 |
| Thiosulfativibrio zosterae | 14 | 40 | 27 | 4 | 39 |
| Thiosulfatimonas sediminis | 27 | 111 | 74 | 6 | 83 |
| Lysobacter caseinilyticus | 0 | 0 | 16714 | 0 | 10764 |
| Corynebacterium comes | 0 | 0 | 0 | 0 | 0 |
| Micromonospora sp. WMMC415 | 2877 | 2829 | 7551 | 403 | 9725 |
| Novosphingobium sp. Gsoil 351 | 840 | 1112 | 1056 | 25 | 1584 |
| Sulfuriferula nivalis | 61 | 159 | 96 | 0 | 132 |
| Bizionia sp. M204 | 6 | 24 | 18 | 0 | 36 |
| Erwinia sp. E602 | 0 | 0 | 0 | 0 | 0 |
| Mycobacterium sp. DL440 | 981 | 3141 | 1915 | 130 | 4604 |
| Mycobacterium sp. DL592 | 1960 | 4524 | 3737 | 100 | 7323 |
| Aeromonas sp. WP2-W18-CRE-05 | 53 | 49 | 247 | 0 | 177 |
| Klebsiella sp. WP3-W18-ESBL-02 | 0 | 107 | 304 | 0 | 0 |
| Klebsiella sp. WP3-S18-ESBL-05 | 0 | 0 | 0 | 0 | 266 |
| Klebsiella sp. WP4-W18-ESBL-05 | 189 | 89 | 0 | 0 | 0 |
| Acinetobacter sp. BEC1-S18-ESBL-01 | 0 | 0 | 0 | 0 | 0 |
| Occultella kanbiaonis | 1833 | 1983 | 3317 | 73 | 3424 |
| Enterobacteriaceae endosymbiont of Donacia bicoloricornis | 0 | 0 | 5 | 0 | 6 |
| Enterobacteriaceae endosymbiont of Donacia cincticornis | 2 | 3 | 3 | 0 | 1 |
| Enterobacteriaceae endosymbiont of Donacia cinerea | 0 | 1 | 2 | 0 | 1 |
| Enterobacteriaceae endosymbiont of Donacia clavipes | 0 | 2 | 2 | 0 | 4 |
| Enterobacteriaceae endosymbiont of Donacia crassipes | 0 | 0 | 0 | 0 | 1 |
| Enterobacteriaceae endosymbiont of Donacia dentata | 0 | 0 | 0 | 0 | 8 |
| Enterobacteriaceae endosymbiont of Donacia fulgens | 0 | 1 | 0 | 0 | 6 |
| Enterobacteriaceae endosymbiont of Donacia piscatrix | 0 | 1 | 0 | 0 | 0 |
| Enterobacteriaceae endosymbiont of Donacia provostii | 17 | 0 | 1 | 0 | 0 |
| Enterobacteriaceae endosymbiont of Donacia proxima | 0 | 1 | 8 | 0 | 1 |
| Enterobacteriaceae endosymbiont of Donacia semicuprea | 0 | 1 | 0 | 0 | 0 |
| Enterobacteriaceae endosymbiont of Donacia simplex | 0 | 7 | 4 | 0 | 2 |
| Enterobacteriaceae endosymbiont of Donacia sparganii | 0 | 0 | 3 | 0 | 0 |
| Enterobacteriaceae endosymbiont of Donacia thalassina | 0 | 0 | 1 | 0 | 0 |
| Enterobacteriaceae endosymbiont of Donacia tomentosa | 1 | 3 | 2 | 0 | 5 |
| Enterobacteriaceae endosymbiont of Donacia versicolorea | 5 | 0 | 9 | 0 | 0 |
| Enterobacteriaceae endosymbiont of Donacia vulgaris | 0 | 3 | 3 | 0 | 0 |
| Enterobacteriaceae endosymbiont of Macroplea appendiculata | 0 | 0 | 0 | 0 | 0 |
| Enterobacteriaceae endosymbiont of Macroplea mutica | 1 | 1 | 1 | 0 | 0 |
| Enterobacteriaceae endosymbiont of Neohaemonia nigricornis | 8 | 10 | 5 | 0 | 2 |
| Enterobacteriaceae endosymbiont of Plateumaris braccata | 1 | 6 | 1 | 0 | 3 |
| Enterobacteriaceae endosymbiont of Plateumaris consimilis | 0 | 0 | 1 | 0 | 4 |
| Enterobacteriaceae endosymbiont of Plateumaris rustica | 0 | 0 | 0 | 0 | 0 |
| Enterobacteriaceae endosymbiont of Plateumaris sericea | 0 | 0 | 3 | 0 | 6 |
| Hymenobacter sp. BRD67 | 0 | 0 | 0 | 0 | 0 |
| Hymenobacter sp. BRD128 | 461 | 870 | 790 | 3 | 957 |
| Sphingobium sp. CAP-1 | 733 | 940 | 686 | 0 | 909 |
| Gordonia sp. 135 | 250 | 741 | 1002 | 7 | 1006 |
| Phnomibacter ginsenosidimutans | 0 | 0 | 0 | 0 | 0 |
| Streptomyces sp. Tu 2975 | 1480 | 1435 | 2898 | 174 | 5183 |
| Pseudomonas sp. St386 | 27 | 83 | 61 | 0 | 121 |
| Pseudomonas sp. St316 | 202 | 193 | 202 | 0 | 337 |
| Pseudomonas sp. Cab53 | 0 | 0 | 0 | 0 | 0 |
| Pseudomonas sp. Seg1 | 192 | 347 | 425 | 28 | 847 |
| Pseudomonas sp. Ost2 | 505 | 648 | 635 | 5 | 922 |
| Pseudomonas sp. Pc102 | 246 | 370 | 438 | 6 | 611 |
| Pseudodesulfovibrio cashew | 504 | 717 | 799 | 12 | 921 |
| Mannheimia ovis | 27 | 0 | 2 | 0 | 2 |
| Mannheimia sp. ZY171111 | 0 | 0 | 16 | 0 | 18 |
| Clostridium sp. 16K-1-R1 | 0 | 59 | 50 | 0 | 374 |
| Leclercia sp. J807 | 61 | 47 | 88 | 0 | 150 |
| Leclercia sp. 119287 | 44 | 97 | 114 | 0 | 209 |
| Leclercia sp. Colony189 | 78 | 215 | 242 | 0 | 205 |
| Methylococcus geothermalis | 913 | 1158 | 1171 | 15 | 1404 |
| Chlamydiifrater phoenicopteri | 30 | 10 | 6 | 0 | 23 |
| Chlamydiifrater volucris | 7 | 6 | 13 | 0 | 15 |
| Microbulbifer sp. SH-1 | 421 | 543 | 485 | 4 | 649 |
| Sphingomonas profundi | 1744 | 2227 | 1917 | 60 | 3831 |
| Variovorax sp. RKNM96 | 0 | 0 | 0 | 0 | 0 |
| Maribellus comscasis | 51 | 124 | 46 | 0 | 67 |
| Sporofaciens musculi | 0 | 0 | 1 | 0 | 0 |
| Pseudomonas bijieensis | 70 | 188 | 252 | 0 | 591 |
| Blochmannia endosymbiont of Camponotus nipponensis | 4 | 3 | 6 | 0 | 0 |
| Blochmannia endosymbiont of Colobopsis nipponica | 11 | 21 | 11 | 0 | 22 |
| Mucilaginibacter ginkgonis | 106 | 132 | 179 | 3 | 177 |
| Veillonella sp. S12025-13 | 0 | 0 | 0 | 0 | 0 |
| Bacillus sp. N1-1 | 0 | 51 | 25 | 0 | 75 |
| Xiamenia xianingshaonis | 218 | 707 | 554 | 19 | 777 |
| Iocasia fonsfrigidae | 10 | 50 | 46 | 0 | 70 |
| Brevibacillus sp. 7WMA2 | 0 | 22 | 10 | 0 | 16 |
| Spartinivicinus ruber | 30 | 39 | 33 | 13 | 40 |
| Diaminobutyricimonas sp. LJ205 | 854 | 1302 | 1624 | 29 | 1786 |
| Rhodococcus sp. 21391 | 589 | 958 | 1116 | 165 | 1480 |
| Bacillus sp. ms-22 | 3 | 35 | 0 | 0 | 0 |
| Citrobacter sp. 172116965 | 0 | 0 | 14 | 0 | 12 |
| Mycoplasma sp. NEAQ87857 | 16 | 6 | 0 | 0 | 1 |
| Pseudomonas izuensis | 0 | 0 | 0 | 0 | 0 |
| Corynebacterium poyangense | 37 | 129 | 149 | 0 | 125 |
| Streptomyces sp. QHH-9511 | 1831 | 1784 | 3493 | 123 | 5240 |
| Streptomyces sp. SCUT-3 | 2300 | 2101 | 4212 | 651 | 11619 |
| Streptococcus sp. CNU 77-61 | 0 | 0 | 0 | 0 | 5 |
| Nocardioides ochotonae | 2828 | 3792 | 4671 | 201 | 5661 |
| Neorickettsia findlayensis | 21 | 20 | 14 | 0 | 26 |
| Winogradskyella forsetii | 0 | 0 | 0 | 0 | 0 |
| Winogradskyella schleiferi | 34 | 24 | 25 | 0 | 31 |
| Terricaulis silvestris | 1529 | 2110 | 1568 | 7 | 2178 |
| Streptococcus sp. CNU G2 | 0 | 0 | 0 | 0 | 9 |
| Streptomyces broussonetiae | 1801 | 1580 | 2924 | 188 | 5105 |
| Paraglaciecola sp. L3A3 | 0 | 0 | 0 | 0 | 0 |
| Paraglaciecola sp. L1A13 | 19 | 48 | 34 | 18 | 51 |
| Cobetia sp. L2A1 | 214 | 406 | 313 | 0 | 369 |
| Polaribacter sp. L3A8 | 10 | 14 | 11 | 14 | 7 |
| Cellulophaga sp. L1A9 | 14 | 15 | 9 | 0 | 20 |
| Formosa sp. L2A11 | 31 | 13 | 34 | 0 | 15 |
| Algibacter sp. L1A34 | 0 | 0 | 0 | 0 | 0 |
| Algibacter sp. L3A6 | 8 | 33 | 24 | 0 | 27 |
| Polynucleobacter sp. AM-7D1 | 48 | 26 | 22 | 0 | 21 |
| Polynucleobacter sp. MWH-CaK5 | 32 | 49 | 45 | 1 | 85 |
| Polynucleobacter sp. MWH-Spelu-300-X4 | 17 | 50 | 30 | 0 | 21 |
| Polynucleobacter sp. MWH-UH24A | 16 | 58 | 65 | 2 | 206 |
| Colwellia sp. 20A7 | 11 | 8 | 15 | 3 | 12 |
| Eggerthella guodeyinii | 567 | 910 | 1038 | 62 | 1631 |
| Stenotrophomonas sp. 364 | 0 | 0 | 0 | 0 | 0 |
| Prevotella sp. Rep29 | 35 | 127 | 111 | 0 | 156 |
| Brucella sp. BO3 | 41 | 69 | 64 | 0 | 72 |
| Nanchangia anserum | 475 | 571 | 710 | 31 | 1029 |
| Spirosoma aureum | 0 | 0 | 0 | 0 | 0 |
| Streptomyces sp. HF10 | 971 | 909 | 1836 | 98 | 2018 |
| Streptomyces sp. GS7 | 1888 | 1522 | 3086 | 286 | 5075 |
| Streptomyces sp. GF20 | 1300 | 1330 | 2314 | 96 | 4082 |
| Falsirhodobacter sp. PG104 | 0 | 0 | 0 | 0 | 0 |
| Pontibacillus sp. HMF3514 | 17 | 18 | 26 | 0 | 57 |
| Pseudoalteromonas sp. M8 | 7 | 13 | 25 | 0 | 0 |
| Pontibacter russatus | 313 | 898 | 697 | 3 | 993 |
| Pontibacter pudoricolor | 0 | 0 | 0 | 0 | 0 |
| Streptomyces sp. HM190 | 1490 | 1498 | 2590 | 142 | 4546 |
| Lactobacillus sp. 3B(2020) | 15 | 7 | 42 | 1 | 14 |
| Anaerocolumna sedimenticola | 6 | 44 | 23 | 0 | 29 |
| Aquirufa lenticrescens | 68 | 103 | 124 | 0 | 134 |
| Winogradskyella helgolandensis | 7 | 29 | 15 | 5 | 27 |
| Proteus sp. ZN5 | 3 | 21 | 71 | 0 | 20 |
| Pseudomonas sp. AN-B15 | 0 | 0 | 0 | 0 | 0 |
| Sodalis ligni | 0 | 0 | 0 | 0 | 0 |
| Aminipila terrae | 9 | 164 | 63 | 0 | 92 |
| Xylophilus rhododendri | 1760 | 2074 | 2138 | 52 | 3798 |
| Aristophania vespae | 10 | 33 | 31 | 0 | 29 |
| Tichowtungia aerotolerans | 165 | 436 | 542 | 0 | 645 |
| Acidocella sp. MX-AZ03 | 1031 | 1120 | 1032 | 28 | 1597 |
| Citrobacter sp. LUTT5 | 13 | 0 | 27 | 0 | 0 |
| Klebsiella sp. MPUS7 | 8 | 8 | 19 | 0 | 21 |
| Brevibacterium atlanticum | 501 | 714 | 848 | 15 | 1064 |
| Brevibacterium limosum | 283 | 506 | 658 | 25 | 986 |
| Pseudarthrobacter psychrotolerans | 1210 | 3278 | 3455 | 36 | 5401 |
| Nibribacter ruber | 147 | 289 | 233 | 4 | 363 |
| Muricauda oceani | 52 | 125 | 130 | 0 | 131 |
| Novosphingobium decolorationis | 1574 | 1822 | 1838 | 28 | 2358 |
| Sphingomonas changnyeongensis | 745 | 940 | 597 | 2 | 1465 |
| Lysobacter oculi | 799 | 796 | 855 | 0 | 778 |
| Leuconostoc sp. MTCC 10508 | 17 | 22 | 23 | 0 | 65 |
| Sinimarinibacterium sp. NLF-5-8 | 800 | 1303 | 1065 | 7 | 1018 |
| Shewanella sp. Arc9-LZ | 25 | 33 | 27 | 0 | 33 |
| Streptococcus sp. CNU G3 | 0 | 30 | 8 | 0 | 15 |
| Methylosinus sp. C49 | 2346 | 2008 | 1730 | 35 | 2531 |
| Pseudoalteromonas sp. APM04 | 0 | 7 | 12 | 0 | 3 |
| Caldichromatium japonicum | 591 | 995 | 955 | 13 | 1216 |
| Acidovorax sp. 210-6 | 15 | 13 | 0 | 0 | 34 |
| Staphylococcus roterodami | 19 | 3 | 0 | 1 | 3 |
| Virgibacillus sp. MSP4-1 | 39 | 76 | 28 | 3 | 36 |
| Edaphobacter sp. 12200R-103 | 989 | 2353 | 2471 | 13 | 2773 |
| Mucilaginibacter sp. 14171R-50 | 0 | 0 | 0 | 0 | 0 |
| Rahnella aceris | 0 | 0 | 0 | 0 | 0 |
| Flavobacterium bizetiae | 31 | 405 | 191 | 1 | 157 |
| Paenibacillus lycopersici | 504 | 1095 | 1019 | 42 | 1493 |
| Paenibacillus rhizovicinus | 283 | 744 | 685 | 19 | 802 |
| Rhodocytophaga rosea | 0 | 0 | 0 | 0 | 0 |
| Cellulomonas sp. H30R-01 | 2262 | 2175 | 3582 | 57 | 3454 |
| Streptacidiphilus sp. P02-A3a | 3639 | 2826 | 5684 | 641 | 13286 |
| Apibacter sp. ESL0404 | 2 | 11 | 9 | 0 | 32 |
| Apibacter sp. ESL0432 | 0 | 16 | 6 | 0 | 12 |
| Gilliamella sp. ESL0405 | 9 | 27 | 15 | 0 | 24 |
| Gilliamella sp. ESL0441 | 0 | 11 | 13 | 0 | 20 |
| Gilliamella sp. ESL0443 | 6 | 7 | 18 | 0 | 5 |
| Streptacidiphilus sp. PB12-B1b | 2703 | 2831 | 4634 | 428 | 7445 |
| Streptomyces sp. S4.7 | 1484 | 1223 | 2631 | 167 | 3710 |
| Enterobacter sp. SES19 | 9 | 33 | 36 | 0 | 42 |
| Gramella sp. MT6 | 32 | 49 | 26 | 0 | 38 |
| Pseudomonas sp. MTM4 | 720 | 570 | 653 | 46 | 560 |
| Algoriphagus sp. NBT04N3 | 63 | 59 | 43 | 5 | 52 |
| Caballeronia sp. SBC2 | 409 | 976 | 859 | 37 | 1512 |
| Caballeronia sp. SBC1 | 911 | 1299 | 1114 | 0 | 1914 |
| Nitrosophilus labii | 69 | 73 | 40 | 0 | 35 |
| Draconibacterium halophilum | 34 | 59 | 73 | 0 | 82 |
| Paenibacillus lutimineralis | 59 | 282 | 200 | 0 | 190 |
| Adlercreutzia hattorii | 371 | 545 | 675 | 10 | 900 |
| Sulfuriroseicoccus oceanibius | 346 | 1052 | 742 | 29 | 1064 |
| Legionella antarctica | 37 | 37 | 35 | 1 | 19 |
| Microbacterium fandaimingii | 413 | 563 | 742 | 48 | 910 |
| Mumia sp. ZJ1417 | 3574 | 4855 | 5519 | 241 | 5610 |
| Salinibacterium sp. ZJ70 | 799 | 1205 | 1545 | 73 | 2091 |
| Mesoflavibacter profundi | 0 | 0 | 13 | 0 | 19 |
| Capnocytophaga endodontalis | 13 | 29 | 13 | 1 | 11 |
| Kineobactrum salinum | 627 | 794 | 759 | 9 | 1212 |
| Salinibacterium sp. ZJ450 | 1006 | 1722 | 1735 | 78 | 2010 |
| Actinomyces sp. zg-332 | 2 | 4 | 13 | 3 | 63 |
| Acinetobacter sp. WY4 | 35 | 19 | 22 | 12 | 15 |
| Psychrobacter sp. WY6 | 0 | 0 | 0 | 0 | 0 |
| Caulobacter soli | 1007 | 2651 | 1279 | 0 | 645 |
| Massilia sp. Dwa41.01b | 998 | 1346 | 1627 | 0 | 1537 |
| Massilia sp. Se16.2.3 | 228 | 872 | 496 | 0 | 1449 |
| Microbacterium sp. Se63.02b | 386 | 723 | 837 | 109 | 2020 |
| Starkeya sp. ORNL1 | 5430 | 4321 | 3965 | 111 | 6233 |
| Bacteroides sp. ZJ-18 | 0 | 0 | 26 | 0 | 14 |
| Corynebacterium lizhenjunii | 204 | 187 | 226 | 11 | 458 |
| Neisseria sp. ZJ785 | 21 | 70 | 50 | 0 | 101 |
| Schaalia sp. ZJ405 | 117 | 141 | 210 | 23 | 402 |
| Bacillus sp. KH172YL63 | 56 | 122 | 198 | 7 | 98 |
| Spirosoma sp. KCTC 72228 | 0 | 0 | 0 | 0 | 0 |
| Streptomyces bathyalis | 2885 | 2316 | 4567 | 291 | 6292 |
| Phototrophicus methaneseepsis | 0 | 0 | 0 | 0 | 0 |
| Rhizorhabdus phycosphaerae | 1241 | 1291 | 1069 | 11 | 1782 |
| Sphingosinithalassobacter tenebrarum | 873 | 976 | 798 | 0 | 1381 |
| Vibrio ziniensis | 4 | 26 | 23 | 2 | 84 |
| Roseimicrobium sp. ORNL1 | 2047 | 5155 | 4471 | 18 | 3907 |
| Kocuria sp. TGY1127_2 | 381 | 485 | 785 | 41 | 786 |
| Citrobacter sp. SX212 | 0 | 167 | 0 | 0 | 0 |
| Nordella sp. HKS 07 | 9376 | 5719 | 7668 | 219 | 7480 |
| Nocardioides anomalus | 3934 | 5482 | 7191 | 202 | 8545 |
| Salaquimonas pukyongi | 0 | 0 | 0 | 0 | 0 |
| Chryseobacterium sp. POL2 | 0 | 0 | 0 | 0 | 0 |
| Companilactobacillus pabuli | 3 | 3 | 8 | 0 | 15 |
| Acinetobacter sp. Marseille-Q1620 | 29 | 7 | 24 | 10 | 16 |
| Vescimonas fastidiosa | 279 | 627 | 563 | 4 | 593 |
| Vescimonas coprocola | 182 | 504 | 554 | 10 | 642 |
| Pusillibacter faecalis | 184 | 262 | 348 | 1 | 367 |
| Streptomyces sp. JB150 | 1425 | 1366 | 2615 | 266 | 4278 |
| Nitrosophilus alvini | 2 | 20 | 24 | 0 | 78 |
| Acidovorax sp. HDW3 | 0 | 0 | 0 | 0 | 0 |
| Diaphorobacter sp. HDW4A | 1770 | 2731 | 2694 | 41 | 3346 |
| Diaphorobacter sp. HDW4B | 706 | 868 | 775 | 0 | 1297 |
| Dysgonomonas sp. HDW5A | 0 | 6 | 8 | 0 | 33 |
| Dysgonomonas sp. HDW5B | 4 | 21 | 8 | 0 | 9 |
| Erysipelothrix sp. HDW6B | 0 | 0 | 0 | 0 | 0 |
| Erysipelothrix sp. HDW6C | 33 | 40 | 4 | 0 | 3 |
| Sanguibacter sp. HDW7 | 641 | 946 | 1463 | 24 | 560 |
| Hymenobacter sp. HDW8 | 380 | 1200 | 755 | 0 | 1084 |
| Leucobacter coleopterorum | 0 | 0 | 0 | 0 | 0 |
| Leucobacter viscericola | 398 | 390 | 502 | 7 | 667 |
| Lysobacter sp. HDW10 | 0 | 0 | 0 | 0 | 0 |
| Propioniciclava coleopterorum | 2019 | 2634 | 4235 | 197 | 4622 |
| Nocardioides piscis | 1306 | 2902 | 3707 | 122 | 4534 |
| Nocardioides sp. HDW12B | 2637 | 4667 | 5331 | 248 | 5323 |
| Pedobacter sp. HDW13 | 9 | 133 | 211 | 3 | 178 |
| Phycicoccus sp. HDW14 | 1811 | 2122 | 3346 | 87 | 3159 |
| Sphingomonas sp. HDW15A | 980 | 970 | 823 | 8 | 1236 |
| Sphingomonas piscis | 885 | 991 | 707 | 0 | 1454 |
| Sphingomonas sinipercae | 1012 | 1183 | 833 | 24 | 1837 |
| Thermomonas sp. HDW16 | 1091 | 944 | 859 | 48 | 913 |
| Vagococcus coleopterorum | 0 | 0 | 0 | 0 | 0 |
| Vagococcus hydrophili | 1 | 10 | 13 | 0 | 5 |
| Vibrio sp. HDW18 | 14 | 47 | 56 | 0 | 59 |
| Weissella coleopterorum | 3 | 19 | 4 | 0 | 4 |
| Leclercia sp. 29361 | 0 | 0 | 0 | 0 | 0 |
| Mangrovivirga cuniculi | 34 | 58 | 60 | 0 | 85 |
| Acinetobacter lanii | 0 | 0 | 0 | 0 | 0 |
| Acinetobacter shaoyimingii | 0 | 0 | 0 | 0 | 0 |
| Bacillus rugosus | 38 | 54 | 48 | 0 | 17 |
| Bacteroides faecium | 48 | 55 | 50 | 0 | 70 |
| Blattabacterium sp. DPU | 6 | 0 | 0 | 0 | 1 |
| Sulfurimicrobium lacus | 884 | 1579 | 1577 | 30 | 2770 |
| Dissulfurispira thermophila | 90 | 364 | 200 | 0 | 642 |
| Candidatus Chazhemtobacterium aquaticus | 20 | 51 | 43 | 2 | 116 |
| Shinella sp. PSBB067 | 1842 | 2381 | 2078 | 17 | 3083 |
| Hydrogenophaga crocea | 1888 | 2301 | 2653 | 0 | 3839 |
| Pusillimonas sp. DMV24BSW_D | 88 | 207 | 195 | 21 | 321 |
| Gephyromycinifex aptenodytis | 773 | 865 | 1512 | 158 | 2490 |
| Candidatus Frankia datiscae | 2430 | 2266 | 3829 | 225 | 6562 |
| Citrobacter sp. Y3 | 33 | 0 | 0 | 0 | 87 |
| Streptomyces liangshanensis | 2174 | 1954 | 3972 | 175 | 5925 |
| Desulfovibrio subterraneus | 100 | 480 | 394 | 13 | 632 |
| Methylobacterium sp. SyP6R | 282 | 387 | 356 | 0 | 451 |
| Phyllobacterium sp. 628 | 911 | 673 | 573 | 11 | 679 |
| Saccharopolyspora sp. ASAGF58 | 4908 | 2109 | 5714 | 370 | 7253 |
| Nonlabens sp. SY33080 | 3 | 11 | 7 | 0 | 38 |
| Leeuwenhoekiella sp. ZYFB001 | 24 | 44 | 64 | 0 | 95 |
| Entomospira culicis | 17 | 22 | 23 | 1 | 57 |
| Enterobacter sp. DNB-S2 | 18 | 32 | 71 | 0 | 37 |
| Streptomyces sp. 891-h | 2123 | 1915 | 3232 | 214 | 5710 |
| Candidatus Rhabdochlamydia oedothoracis | 0 | 70 | 27 | 0 | 49 |
| Streptomyces sp. DSM 40868 | 2026 | 1564 | 2777 | 121 | 4277 |
| Streptomyces sp. RLB1-33 | 2710 | 2423 | 4918 | 311 | 5643 |
| Streptomyces sp. RPA4-2 | 2250 | 1796 | 3300 | 172 | 4969 |
| Streptomyces sp. RPA4-5 | 1301 | 635 | 1760 | 22 | 1853 |
| Streptomyces sp. S1D4-11 | 2541 | 2133 | 4228 | 231 | 6717 |
| Marinagarivorans cellulosilyticus | 56 | 88 | 56 | 2 | 88 |
| Arcanobacterium buesumense | 17 | 210 | 58 | 2 | 47 |
| Iamia sp. SCSIO 61187 | 7447 | 5696 | 14398 | 79 | 10985 |
| Brenneria izadpanahii | 228 | 312 | 273 | 25 | 711 |
| Rhodococcus sp. DMU1 | 629 | 769 | 716 | 0 | 1308 |
| Xanthomonas sp. GW | 505 | 678 | 573 | 24 | 851 |
| Xanthomonas sp. SS | 0 | 0 | 0 | 0 | 0 |
| Xanthomonas sp. SI | 733 | 404 | 575 | 21 | 598 |
| Flavobacterium sp. CLA17 | 0 | 0 | 0 | 0 | 0 |
| Romboutsia sp. CE17 | 20 | 35 | 66 | 5 | 30 |
| Ferrimonas lipolytica | 66 | 55 | 71 | 0 | 106 |
| Serratia sp. JUb9 | 9 | 8 | 0 | 0 | 35 |
| Pseudomonas sp. BIGb0427 | 21 | 99 | 101 | 0 | 127 |
| Parasphingopyxis sp. CP4 | 0 | 0 | 0 | 0 | 0 |
| Nitratiruptor sp. YY09-18 | 8 | 93 | 35 | 0 | 125 |
| Leifsonia sp. PS1209 | 1533 | 1568 | 2172 | 22 | 2095 |
| Pseudodesulfovibrio sp. zrk46 | 102 | 323 | 422 | 3 | 371 |
| Pseudomonas tohonis | 398 | 666 | 857 | 0 | 809 |
| Kosakonia sp. SMBL-WEM22 | 189 | 214 | 240 | 0 | 389 |
| Brevibacterium sp. 'Marine' | 538 | 471 | 598 | 40 | 1017 |
| Acinetobacter sp. NEB149 | 0 | 28 | 29 | 0 | 42 |
| Mycoplasma sp. 1654_15 | 0 | 1 | 7 | 0 | 2 |
| Mycoplasma phocoeninasale | 5 | 10 | 12 | 0 | 7 |
| Streptomyces sp. Jing01 | 1395 | 1279 | 2147 | 121 | 3418 |
| Marinomonas profundi | 46 | 55 | 43 | 7 | 53 |
| Paenibacillus albicereus | 744 | 1492 | 1709 | 32 | 2358 |
| Novosphingobium terrae | 253 | 334 | 304 | 0 | 522 |
| Sarcina sp. JB2 | 39 | 17 | 20 | 0 | 21 |
| Massilia forsythiae | 977 | 2100 | 2254 | 0 | 3195 |
| Duganella dendranthematis | 1053 | 1996 | 2246 | 0 | 9285 |
| Mucilaginibacter robiniae | 81 | 98 | 78 | 0 | 109 |
| Cohnella herbarum | 140 | 332 | 364 | 2 | 436 |
| Spirosoma rhododendri | 226 | 747 | 624 | 6 | 902 |
| Catellatospora sp. IY07-71 | 8013 | 6212 | 43218 | 404 | 23003 |
| Luteolibacter luteus | 867 | 3609 | 2110 | 8 | 2800 |
| Bacillus fonticola | 1 | 34 | 53 | 0 | 42 |
| Cellulomonas taurus | 1246 | 1399 | 2234 | 71 | 2877 |
| Sediminibacillus dalangtanensis | 39 | 56 | 65 | 0 | 57 |
| Pseudomonas sp. SK | 182 | 179 | 185 | 13 | 350 |
| Methylacidimicrobium sp. AP8 | 611 | 1329 | 1113 | 17 | 1427 |
| Halomonas sp. PGE1 | 560 | 749 | 754 | 0 | 503 |
| Pseudomonas sp. ADAK2 | 3381 | 0 | 0 | 0 | 4117 |
| Pseudomonas sp. ADAK13 | 0 | 0 | 0 | 0 | 0 |
| Pseudomonas sp. ADAK18 | 0 | 0 | 0 | 0 | 0 |
| Pseudomonas sp. ADAK20 | 0 | 1419 | 0 | 0 | 0 |
| Pseudomonas sp. ADAK22 | 110 | 142 | 102 | 0 | 363 |
| Halomonas sp. TA6 | 0 | 0 | 0 | 0 | 2614 |
| Streptomyces sp. Z423-1 | 2294 | 1776 | 3456 | 155 | 5574 |
| Rhizobium sp. NZLR1 | 1006 | 1018 | 900 | 12 | 998 |
| Rhizobium sp. NLR16a | 782 | 853 | 638 | 4 | 788 |
| Alkaliphilus sp. B6464 | 125 | 36 | 56 | 0 | 25 |
| Alkalicella caledoniensis | 49 | 19 | 121 | 0 | 31 |
| Pseudomonas sp. Sm006 | 303 | 513 | 477 | 19 | 837 |
| Cellulosimicrobium sp. 72-3 | 823 | 570 | 894 | 15 | 1139 |
| Pseudomonas campi | 0 | 0 | 0 | 0 | 0 |
| Teredinibacter haidensis | 29 | 118 | 74 | 2 | 106 |
| Teredinibacter purpureus | 70 | 46 | 67 | 7 | 89 |
| Usitatibacter rugosus | 5062 | 6237 | 6353 | 151 | 11536 |
| Gemmatimonas groenlandica | 2829 | 9263 | 7577 | 105 | 28399 |
| Usitatibacter palustris | 3618 | 4888 | 4941 | 135 | 9619 |
| Synechocystis sp. PCC 7338 | 0 | 107 | 86 | 0 | 199 |
| Candidatus Vesicomyosocius sp. SY067_SCS001 | 12 | 17 | 27 | 0 | 17 |
| Wolbachia endosymbiont of Ctenocephalides felis wCfeT | 3 | 5 | 14 | 0 | 11 |
| Wolbachia endosymbiont of Ctenocephalides felis wCfeJ | 8 | 4 | 9 | 0 | 8 |
| Halomonas sulfidoxydans | 782 | 1122 | 879 | 0 | 854 |
| Halomonas sp. MCCC 1A13316 | 378 | 625 | 703 | 5 | 848 |
| Halomonas sulfidivorans | 431 | 616 | 626 | 11 | 917 |
| Trueperella pecoris | 530 | 870 | 1024 | 31 | 1291 |
| Microbacterium sp. NIBRBAC000506063 | 1062 | 1633 | 2561 | 96 | 2595 |
| Berryella wangjianweii | 0 | 0 | 0 | 0 | 0 |
| Herbiconiux sp. SALV-R1 | 1495 | 1923 | 2274 | 43 | 2703 |
| Sphingomonas sp. AP4-R1 | 1558 | 1622 | 1347 | 9 | 2525 |
| Paenarthrobacter sp. YJN-5 | 401 | 487 | 986 | 9 | 1450 |
| Paenarthrobacter sp. YJN-D | 0 | 0 | 0 | 0 | 0 |
| Hymenobacter sp. TS19 | 0 | 423 | 0 | 0 | 0 |
| Paraburkholderia sp. PGU16 | 580 | 1040 | 1153 | 0 | 842 |
| Paraburkholderia sp. PGU19 | 701 | 1643 | 1289 | 16 | 1903 |
| Mariniplasma anaerobium | 0 | 29 | 36 | 0 | 48 |
| Agrobacterium vaccinii | 982 | 869 | 763 | 4 | 1597 |
| Candidatus Absconditicoccus praedator | 34 | 57 | 53 | 0 | 79 |
| Thiothrix subterranea | 241 | 241 | 226 | 0 | 341 |
| Turicibacter bilis | 0 | 33 | 11 | 0 | 22 |
| Chromobacterium sp. Beijing | 385 | 603 | 441 | 14 | 794 |
| Exiguobacterium sp. Helios | 58 | 73 | 97 | 1 | 137 |
| Spirosoma taeanense | 147 | 675 | 555 | 6 | 518 |
| Aquimarina sp. TRL1 | 1 | 26 | 32 | 0 | 23 |
| Pseudonocardia broussonetiae | 6786 | 5208 | 14622 | 358 | 11825 |
| Mycolicibacterium mengxianglii | 1869 | 4968 | 3512 | 78 | 7696 |
| Nocardioides campestrisoli | 1703 | 2407 | 3374 | 84 | 3532 |
| Bradyrhizobium sp. 14AB | 2801 | 2157 | 1191 | 0 | 397 |
| Deinococcus sp. TS-293 | 540 | 1058 | 1026 | 40 | 1705 |
| Actinomyces marmotae | 0 | 0 | 0 | 0 | 0 |
| Massilia sp. erpn | 361 | 921 | 870 | 33 | 2001 |
| Mycobacterium ostraviense | 1743 | 3570 | 2388 | 68 | 4361 |
| Streptomyces sp. A1-5 | 927 | 1225 | 1836 | 20 | 3644 |
| Acidovorax sp. YS12 | 1075 | 1737 | 1523 | 0 | 1351 |
| Pseudomonas sp. B14-6 | 149 | 104 | 103 | 48 | 330 |
| Polaribacter pectinis | 2 | 15 | 10 | 0 | 9 |
| Candidatus Ruthia endofausta | 11 | 29 | 26 | 0 | 63 |
| Brevibacillus sp. DP1.3A | 99 | 82 | 61 | 0 | 101 |
| Candidatus Reidiella endopervernicosa | 0 | 0 | 0 | 0 | 0 |
| Brevibacillus sp. HD3.3A | 87 | 405 | 166 | 0 | 137 |
| Pectobacterium sp. PL64 | 56 | 34 | 56 | 0 | 55 |
| Deefgea piscis | 106 | 602 | 382 | 19 | 433 |
| Flavobacterium sp. M31R6 | 0 | 0 | 0 | 0 | 0 |
| Thiomicrorhabdus xiamenensis | 47 | 177 | 121 | 5 | 145 |
| Erythrobacter mangrovi | 0 | 0 | 0 | 0 | 0 |
| Chitinibacter bivalviorum | 87 | 485 | 450 | 4 | 403 |
| Geomonas paludis | 1120 | 2260 | 2060 | 36 | 2616 |
| Martelella sp. NC18 | 1109 | 0 | 565 | 0 | 0 |
| Martelella sp. NC20 | 0 | 1137 | 625 | 0 | 1573 |
| Candidatus Sulfurimonas baltica | 11 | 8 | 11 | 0 | 2 |
| Candidatus Sulfurimonas marisnigri | 29 | 31 | 8 | 0 | 4 |
| Mucilaginibacter mali | 127 | 273 | 243 | 4 | 289 |
| Hydrogenimonas urashimensis | 162 | 319 | 298 | 1 | 297 |
| Microvirga sp. R24 | 2919 | 2456 | 2219 | 96 | 3257 |
| Aquisalimonas sp. 2447 | 1035 | 1307 | 1357 | 11 | 1844 |
| Acaryochloris sp. 'Moss Beach' | 10 | 126 | 129 | 0 | 112 |
| Paenibacillus sp. URB8-2 | 177 | 402 | 320 | 0 | 401 |
| Serratia surfactantfaciens | 151 | 221 | 145 | 40 | 159 |
| Rhodoferax sp. BAB1 | 787 | 1181 | 1122 | 21 | 1669 |
| Synechococcus sp. LA31 | 151 | 286 | 363 | 10 | 400 |
| Actinomadura sp. NAK00032 | 6077 | 4735 | 10711 | 1464 | 30165 |
| Stenotrophomonas sp. NA06056 | 0 | 0 | 0 | 0 | 0 |
| Amycolatopsis sp. Hca4 | 17672 | 4694 | 16518 | 407 | 14813 |
| Verrucosispora sp. NA02020 | 1694 | 1710 | 4729 | 318 | 5720 |
| Streptomyces sp. NA02536 | 1122 | 927 | 1821 | 88 | 3320 |
| Streptomyces sp. NA03103 | 309 | 522 | 803 | 136 | 1430 |
| Kitasatospora sp. NA04385 | 3084 | 2289 | 4181 | 291 | 6879 |
| Streptomyces sp. NA04227 | 2396 | 1683 | 3625 | 338 | 6467 |
| Streptomyces sp. NA02950 | 2642 | 1951 | 3979 | 321 | 7994 |
| Oricola thermophila | 1263 | 1225 | 1218 | 35 | 1749 |
| Mycoplasma sp. OR1901 | 0 | 2 | 0 | 0 | 5 |
| Methyloligella sp. GL2 | 3518 | 2248 | 2558 | 38 | 2389 |
| Limosilactobacillus portuensis | 0 | 7 | 12 | 0 | 8 |
| Rhodococcus sp. W8901 | 2452 | 1825 | 3320 | 41 | 3278 |
| Citrobacter sp. RHB25-C09 | 0 | 0 | 0 | 0 | 0 |
| Citrobacter sp. RHB35-C17 | 7 | 5 | 28 | 0 | 0 |
| Citrobacter sp. RHB35-C21 | 88 | 70 | 44 | 0 | 74 |
| Citrobacter sp. RHB36-C18 | 10 | 15 | 0 | 0 | 0 |
| Enterobacter sp. RHBSTW-00175 | 0 | 0 | 0 | 0 | 0 |
| Citrobacter sp. RHBSTW-00229 | 0 | 0 | 0 | 0 | 107 |
| Enterobacter sp. RHBSTW-00422 | 0 | 0 | 0 | 0 | 131 |
| Citrobacter sp. RHBSTW-00424 | 0 | 0 | 654 | 0 | 0 |
| Citrobacter sp. RHBSTW-00524 | 0 | 0 | 0 | 0 | 9 |
| Citrobacter sp. RHBSTW-00570 | 36 | 0 | 0 | 0 | 106 |
| Citrobacter sp. RHBSTW-00599 | 0 | 27 | 43 | 0 | 0 |
| Citrobacter sp. RHBSTW-00944 | 0 | 0 | 76 | 0 | 0 |
| Enterobacter sp. RHBSTW-00975 | 0 | 28 | 107 | 0 | 37 |
| Enterobacter sp. RHBSTW-00994 | 113 | 96 | 150 | 0 | 143 |
| Citrobacter sp. RHBSTW-01013 | 0 | 0 | 52 | 0 | 102 |
| Citrobacter sp. RHBSTW-01044 | 0 | 0 | 40 | 0 | 0 |
| Natranaerofaba carboxydovora | 71 | 36 | 66 | 0 | 66 |
| Thalassolituus hydrocarbonoclasticus | 0 | 0 | 0 | 0 | 0 |
| Salicibibacter cibarius | 59 | 87 | 77 | 0 | 118 |
| Salicibibacter cibi | 27 | 87 | 100 | 9 | 107 |
| Acidovorax antarcticus | 1469 | 2312 | 2356 | 57 | 3444 |
| Acinetobacter sp. NEB 394 | 0 | 0 | 0 | 0 | 0 |
| Effusibacillus dendaii | 47 | 195 | 156 | 1 | 178 |
| Pseudomonas sp. 43A | 0 | 0 | 0 | 0 | 1763 |
| Rasiella rasia | 16 | 20 | 23 | 4 | 25 |
| Streptomyces sp. KMM 9044 | 1440 | 1130 | 2038 | 226 | 4386 |
| Flocculibacter collagenilyticus | 16 | 29 | 23 | 4 | 28 |
| Mesorhizobium sp. 113-1-2 | 1928 | 1654 | 1274 | 65 | 1808 |
| Mesorhizobium sp. 113-3-3 | 1513 | 1311 | 1398 | 0 | 1254 |
| Mesorhizobium sp. 113-3-9 | 1509 | 1068 | 1034 | 62 | 978 |
| Mesorhizobium sp. 131-2-1 | 3065 | 2746 | 2511 | 56 | 3355 |
| Mesorhizobium sp. 131-2-5 | 1550 | 1436 | 1423 | 0 | 810 |
| Mesorhizobium sp. 131-3-5 | 0 | 0 | 0 | 0 | 0 |
| Mesorhizobium sp. L-2-11 | 3460 | 5243 | 5160 | 22 | 10644 |
| Mesorhizobium sp. L-8-3 | 3049 | 2790 | 2838 | 2 | 2645 |
| Mesorhizobium sp. L-8-10 | 3848 | 4873 | 4750 | 74 | 5752 |
| Actinomyces respiraculi | 470 | 777 | 855 | 26 | 1335 |
| Amycolatopsis sp. CA-230715 | 15579 | 4227 | 14818 | 285 | 10975 |
| Metabacillus sp. KUDC1714 | 52 | 123 | 70 | 0 | 73 |
| Microbulbifer sp. YPW1 | 297 | 440 | 426 | 9 | 492 |
| Pseudomonas kermanshahensis | 191 | 34 | 69 | 0 | 16 |
| Pseudomonas xantholysinigenes | 0 | 0 | 0 | 0 | 0 |
| Pseudomonas vanderleydeniana | 414 | 686 | 683 | 12 | 1077 |
| Pseudomonas zarinae | 156 | 151 | 143 | 3 | 258 |
| Pseudomonas iranensis | 0 | 0 | 0 | 0 | 0 |
| Pseudomonas hamedanensis | 252 | 425 | 302 | 2 | 570 |
| Pseudomonas monsensis | 68 | 205 | 160 | 17 | 406 |
| Pseudomonas zeae | 316 | 659 | 275 | 5 | 1452 |
| Pseudomonas tensinigenes | 131 | 298 | 344 | 1 | 1241 |
| Pseudomonas shahriarae | 61 | 142 | 181 | 0 | 130 |
| Pseudomonas salmasensis | 0 | 0 | 0 | 0 | 0 |
| Pseudomonas tritici | 183 | 259 | 289 | 15 | 828 |
| Pseudomonas sp. OE 28.3 | 0 | 0 | 0 | 0 | 0 |
| Cellulophaga sp. HaHa_2_95 | 0 | 0 | 5 | 0 | 0 |
| Olleya sp. HaHaR_3_96 | 2 | 37 | 14 | 0 | 15 |
| Polaribacter sp. HaHaR_3_91 | 0 | 14 | 15 | 0 | 0 |
| Polaribacter sp. AHE13PA | 0 | 6 | 0 | 0 | 11 |
| Polaribacter sp. R2A056_3_33 | 0 | 0 | 9 | 0 | 0 |
| Salmonella sp. SJTUF14178 | 0 | 0 | 1289 | 0 | 0 |
| Spirosoma sp. KUDC1026 | 137 | 538 | 423 | 2 | 475 |
| Legionella sp. PC1000 | 24 | 23 | 25 | 5 | 19 |
| Flavobacterium sp. K5-23 | 18 | 160 | 77 | 2 | 89 |
| Moritella sp. 24 | 22 | 38 | 29 | 11 | 42 |
| Moritella sp. 5 | 1 | 21 | 12 | 0 | 13 |
| Moritella sp. 28 | 0 | 12 | 18 | 0 | 0 |
| Moritella sp. 36 | 11 | 6 | 0 | 0 | 38 |
| Pseudomonas phenolilytica | 0 | 0 | 0 | 0 | 0 |
| Shewanella sp. MTB7 | 28 | 57 | 53 | 11 | 103 |
| Dyadobacter sandarakinus | 164 | 386 | 359 | 0 | 327 |
| Enterobacter sp. DSM 30060 | 0 | 24 | 47 | 0 | 68 |
| Pseudomonas sp. ABC1 | 0 | 0 | 0 | 0 | 0 |
| Pikeienuella piscinae | 1618 | 1904 | 1789 | 0 | 2424 |
| Flavobacterium sp. I3-2 | 49 | 96 | 42 | 6 | 65 |
| Flavobacterium inviolabile | 48 | 139 | 50 | 0 | 74 |
| Bradyrhizobium quebecense | 4637 | 4100 | 2163 | 0 | 468 |
| Paenibacillus sp. E222 | 1 | 93 | 61 | 0 | 134 |
| Bdellovibrio sp. KM01 | 0 | 0 | 0 | 0 | 0 |
| Wolbachia endosymbiont of Anopheles demeilloni | 0 | 1 | 0 | 0 | 1 |
| Streptomyces sp. CB04723 | 877 | 835 | 1124 | 171 | 2850 |
| Halomonas sp. SH5A2 | 155 | 314 | 342 | 4 | 292 |
| Treponema sp. B152 | 81 | 7 | 4 | 0 | 15 |
| Pseudomonas sp. Y39-6 | 0 | 0 | 0 | 0 | 0 |
| Lysinibacillus sp. JK80 | 0 | 3 | 0 | 0 | 0 |
| Micromonospora ferruginea | 3278 | 2456 | 5855 | 245 | 6007 |
| Rhodococcus sp. ZPP | 400 | 838 | 1199 | 39 | 1797 |
| Lactococcus carnosus | 8 | 0 | 0 | 0 | 0 |
| Nocardia gipuzkoensis | 4370 | 2480 | 5158 | 188 | 5940 |
| Cellulophaga sp. HaHa_2_1 | 0 | 12 | 0 | 0 | 10 |
| Winogradskyella sp. HaHa_3_26 | 15 | 28 | 27 | 3 | 18 |
| Coxiella endosymbiont of Amblyomma nuttalli | 11 | 17 | 32 | 1 | 14 |
| Streptomyces sp. Rer75 | 2563 | 1513 | 3270 | 161 | 4724 |
| Streptomyces sp. CB00271 | 1001 | 934 | 1670 | 45 | 2328 |
| Chryseoglobus indicus | 588 | 845 | 866 | 24 | 895 |
| Xanthomonas sp. CPBF 426 | 0 | 0 | 0 | 0 | 102 |
| Actinomadura graeca | 4531 | 3723 | 8013 | 1480 | 24836 |
| Bartonella apihabitans | 160 | 242 | 90 | 5 | 120 |
| Bartonella choladocola | 0 | 0 | 0 | 0 | 0 |
| Synechococcus sp. LTW-R | 195 | 401 | 441 | 9 | 363 |
| Synechococcus sp. HK01-R | 92 | 348 | 243 | 0 | 276 |
| Vibrio sp. B1ASS3 | 0 | 28 | 25 | 0 | 17 |
| Vibrio sp. B1FIG11 | 0 | 231 | 64 | 0 | 171 |
| Vibrio sp. B1FLJ16 | 27 | 42 | 64 | 3 | 71 |
| Vibrio sp. B1REV9 | 1 | 18 | 7 | 0 | 22 |
| Streptomyces sp. NEAU-sy36 | 1765 | 1685 | 3002 | 137 | 5086 |
| Koleobacter methoxysyntrophicus | 0 | 0 | 0 | 0 | 0 |
| Rhodoferax sp. AJA081-3 | 0 | 0 | 0 | 0 | 0 |
| Pseudoalteromonas sp. JSTW | 0 | 0 | 0 | 0 | 22 |
| Brevundimonas sp. AJA228-03 | 0 | 0 | 0 | 0 | 0 |
| Rhizobacter sp. AJA081-3 | 2155 | 3545 | 3127 | 0 | 5427 |
| Corynebacterium incognita | 0 | 0 | 0 | 0 | 0 |
| Nocardia huaxiensis | 2348 | 2133 | 4199 | 136 | 4625 |
| Bacteroides sp. CACC 737 | 0 | 26 | 76 | 0 | 120 |
| Actinomyces sp. MAS-1 | 450 | 502 | 691 | 29 | 1185 |
| Mycolicibacterium sp. TY66 | 0 | 2452 | 0 | 0 | 3027 |
| Legionella sp. PC997 | 27 | 45 | 42 | 0 | 29 |
| Borrelia puertoricensis | 5 | 4 | 0 | 0 | 1 |
| Achromobacter sp. 77 | 641 | 546 | 616 | 8 | 850 |
| Chryseobacterium sp. CX-624 | 0 | 0 | 0 | 0 | 0 |
| Pontibrevibacter nitratireducens | 610 | 699 | 593 | 13 | 911 |
| Aureimonas mangrovi | 1962 | 1866 | 1772 | 45 | 2656 |
| Actinotalea sp. JY-7876 | 2113 | 2487 | 3473 | 117 | 4960 |
| Paenibacillus sp. 19GGS1-52 | 72 | 199 | 155 | 1 | 191 |
| Schaalia sp. JY-X169 | 118 | 224 | 227 | 6 | 376 |
| Flaviflexus equikiangi | 0 | 0 | 0 | 0 | 0 |
| Gordonia jinghuaiqii | 1160 | 1276 | 1776 | 93 | 3035 |
| Cobetia sp. 4B | 545 | 232 | 476 | 0 | 431 |
| Brachybacterium sp. Z12 | 698 | 666 | 1096 | 26 | 1534 |
| Streptomyces sp. M54 | 448 | 899 | 1434 | 70 | 2685 |
| Chryseobacterium capnotolerans | 19 | 43 | 21 | 1 | 22 |
| Bartonella sp. HY038 | 0 | 0 | 0 | 0 | 0 |
| Mycolicibacterium sp. TY81 | 1443 | 0 | 0 | 0 | 1083 |
| Streptococcus sp. Marseille-Q3533 | 0 | 0 | 0 | 0 | 0 |
| Pseudoalteromonas sp. MT33b | 0 | 11 | 0 | 0 | 5 |
| Sandaracinobacteroides saxicola | 964 | 1444 | 1357 | 20 | 2214 |
| Protaetiibacter sp. SSC-01 | 1044 | 1768 | 2253 | 28 | 2200 |
| Devosia sp. MC521 | 0 | 0 | 0 | 0 | 0 |
| Tomitella gaofuii | 998 | 1261 | 2184 | 5 | 1984 |
| Nocardioides dongkuii | 2203 | 3625 | 4749 | 89 | 4571 |
| Paracoccus sp. MC1862 | 0 | 0 | 0 | 0 | 0 |
| Propioniciclava sp. MC1595 | 1727 | 2628 | 3577 | 105 | 4174 |
| Tessaracoccus sp. MC1865 | 0 | 0 | 0 | 0 | 0 |
| Nocardioides sp. InS609-2 | 1894 | 3728 | 5052 | 139 | 5089 |
| Lacibacter sp. S13-6-22 | 0 | 0 | 0 | 0 | 0 |
| Luteimonas sp. MC1825 | 1994 | 1488 | 1495 | 19 | 1798 |
| Borrelia maritima | 14 | 4 | 5 | 0 | 8 |
| Mycolicibacterium baixiangningiae | 2340 | 6753 | 4779 | 54 | 10304 |
| Hymenobacter sp. S2-20-2 | 187 | 369 | 329 | 0 | 437 |
| Croceimicrobium hydrocarbonivorans | 8 | 62 | 85 | 0 | 181 |
| Rickettsia tillamookensis | 0 | 19 | 0 | 0 | 8 |
| Entomobacter blattae | 17 | 36 | 24 | 0 | 41 |
| Paenibacillus sp. PAMC21692 | 161 | 389 | 275 | 0 | 413 |
| Variovorax sp. PAMC26660 | 0 | 0 | 0 | 0 | 0 |
| Variovorax sp. PAMC28562 | 1223 | 1615 | 1065 | 8 | 1455 |
| Nakamurella sp. PAMC28650 | 1603 | 1852 | 2952 | 75 | 3763 |
| Bacillus sp. PAMC28748 | 0 | 60 | 501 | 0 | 0 |
| Bacillus sp. PAMC26543 | 0 | 19 | 7 | 0 | 0 |
| Dermacoccus sp. PAMC28757 | 487 | 727 | 1245 | 0 | 131 |
| Streptomyces sp. TYQ1024 | 0 | 0 | 0 | 0 | 6893 |
| Sporosarcina sp. resist | 25 | 114 | 28 | 117 | 199 |
| Pseudomonas kielensis | 73 | 93 | 83 | 0 | 247 |
| Lysobacter sp. CW239 | 1391 | 1506 | 1480 | 0 | 1541 |
| Novosphingopyxis iocasae | 1063 | 1053 | 907 | 0 | 1347 |
| Sphingosinithalassobacter sp. CS137 | 980 | 1107 | 932 | 7 | 1774 |
| Pseudomonas sp. MPDS | 49 | 228 | 139 | 4 | 160 |
| Streptomyces buecherae | 3420 | 2688 | 5720 | 571 | 11779 |
| Nocardioides sp. zg-1228 | 0 | 0 | 0 | 0 | 0 |
| Bacteroides sp. M10 | 9 | 46 | 27 | 0 | 19 |
| Oscillibacter hominis | 204 | 466 | 385 | 16 | 677 |
| Roseburia sp. NSJ-69 | 1 | 48 | 4 | 0 | 13 |
| Streptococcus sp. NSJ-72 | 0 | 0 | 0 | 0 | 0 |
| Edaphobacter sp. 4G125 | 668 | 1855 | 1531 | 17 | 1628 |
| Alloacidobacterium dinghuense | 1397 | 4319 | 3296 | 28 | 3864 |
| Microbacterium sp. YJN-G | 1099 | 1365 | 2267 | 54 | 2437 |
| Lysobacter solisilvae | 1973 | 1994 | 1808 | 0 | 1354 |
| Dyella telluris | 1009 | 1241 | 1107 | 33 | 1130 |
| Actinomyces trachealis | 241 | 244 | 405 | 35 | 936 |
| Wansuia hejianensis | 3 | 127 | 99 | 1 | 161 |
| Qiania dongpingensis | 98 | 164 | 126 | 7 | 191 |
| Wujia chipingensis | 15 | 56 | 72 | 0 | 65 |
| Simiaoa sunii | 0 | 0 | 0 | 0 | 0 |
| Lysobacter sp. CJ11 | 369 | 558 | 450 | 1 | 481 |
| Sphingomonas sabuli | 1536 | 1514 | 1105 | 45 | 2329 |
| Eubacterium hominis | 2 | 20 | 17 | 0 | 11 |
| Fusobacterium hominis | 4 | 40 | 107 | 1 | 76 |
| Sideroxyarcus emersonii | 652 | 1160 | 999 | 4 | 1813 |
| Nostoc sp. MS1 | 55 | 200 | 127 | 10 | 161 |
| Olivibacter sp. SDN3 | 124 | 104 | 93 | 0 | 90 |
| Massilia antarctica | 688 | 2141 | 1726 | 0 | 3099 |
| Leuconostoc falkenbergense | 6 | 10 | 6 | 0 | 19 |
| Caproicibacterium amylolyticum | 53 | 109 | 135 | 2 | 161 |
| Treponema sp. Marseille-Q4132 | 61 | 72 | 68 | 0 | 58 |
| Vibrio sp. sp1 | 0 | 28 | 0 | 0 | 0 |
| Microbacterium sp. Nx66 | 354 | 496 | 959 | 21 | 646 |
| Chlamydia crocodili | 4 | 6 | 4 | 0 | 8 |
| Amniculibacterium sp. G2-70 | 13 | 58 | 39 | 0 | 63 |
| Streptomyces roseirectus | 3038 | 2376 | 4384 | 195 | 5222 |
| Streptomyces genisteinicus | 2425 | 2240 | 4086 | 197 | 5617 |
| Streptomyces xanthii | 2512 | 2246 | 4530 | 256 | 6983 |
| Clavibacter zhangzhiyongii | 743 | 961 | 1193 | 69 | 1280 |
| Roseomonas marmotae | 1798 | 1614 | 1716 | 63 | 2111 |
| Roseomonas haemaphysalidis | 1819 | 1942 | 2034 | 48 | 3032 |
| Pantoea sp. MT58 | 73 | 42 | 44 | 0 | 128 |
| Microbacterium chengjingii | 0 | 0 | 0 | 0 | 0 |
| Brucella sp. 6810 | 84 | 76 | 54 | 0 | 79 |
| Marinobacter sp. LPB0319 | 302 | 457 | 438 | 9 | 530 |
| Thalassotalea sp. LPB0316 | 9 | 28 | 18 | 9 | 34 |
| Massilia sp. LPB0304 | 0 | 0 | 0 | 0 | 0 |
| Polynucleobacter sp. Adler-ghost | 17 | 191 | 27 | 0 | 40 |
| Stenotrophomonas sp. 169 | 616 | 1220 | 904 | 3 | 1308 |
| Yimella sp. cx-51 | 826 | 988 | 1706 | 121 | 2196 |
| Mannheimia bovis | 0 | 0 | 0 | 0 | 0 |
| Clostridioides sp. ES-S-0054-01 | 12 | 20 | 4 | 0 | 26 |
| Clostridioides sp. ES-W-0016-02 | 24 | 0 | 1 | 0 | 6 |
| Wenzhouxiangella sp. AB-CW3 | 886 | 1195 | 1226 | 10 | 1693 |
| Cupriavidus sp. ISTL7 | 418 | 811 | 810 | 128 | 1388 |
| Roseococcus microcysteis | 1758 | 1942 | 1858 | 6 | 2620 |
| Sphingosinicella flava | 661 | 1221 | 805 | 21 | 3531 |
| Thermomonas sp. XSG | 1983 | 1615 | 1351 | 8 | 1684 |
| Chryseoglobus sp. 28M-23 | 479 | 665 | 1037 | 9 | 938 |
| Psychrobacter sp. 28M-43 | 49 | 24 | 12 | 10 | 7 |
| Pseudarthrobacter sp. BIM B-2242 | 540 | 1421 | 1860 | 34 | 3283 |
| Streptomyces sp. KY70 | 0 | 0 | 1826 | 0 | 0 |
| Streptomyces sp. KY75 | 0 | 480 | 0 | 0 | 176 |
| Algoriphagus sp. Y33 | 51 | 66 | 111 | 0 | 114 |
| Desulfomarina profundi | 55 | 99 | 112 | 0 | 129 |
| Serratia sp. Tan611 | 117 | 22 | 48 | 0 | 47 |
| Agreia sp. COWG | 891 | 970 | 1254 | 66 | 1656 |
| Desulfolutivibrio sulfoxidireducens | 587 | 1388 | 1148 | 13 | 1725 |
| Halomonas sp. YLGW01 | 0 | 0 | 0 | 0 | 0 |
| Marinomonas algicola | 38 | 32 | 52 | 5 | 32 |
| Acinetobacter sp. ASP199 | 17 | 24 | 16 | 5 | 16 |
| Pectobacterium quasiaquaticum | 137 | 48 | 44 | 4 | 66 |
| Kluyvera genomosp. 3 | 2 | 72 | 62 | 0 | 80 |
| Devosia rhizoryzae | 936 | 916 | 745 | 0 | 676 |
| Frigoriglobus tundricola | 9952 | 108282 | 42482 | 397 | 50878 |
| Actinobacillus sp. GY-402 | 0 | 0 | 0 | 0 | 0 |
| Brevundimonas pondensis | 0 | 0 | 0 | 0 | 0 |
| Brevundimonas goettingensis | 700 | 1202 | 1020 | 0 | 1317 |
| Pseudomonas sp. IzPS59 | 94 | 162 | 195 | 2 | 296 |
| Pseudomonas allokribbensis | 272 | 310 | 139 | 0 | 378 |
| Pseudomonas gozinkensis | 196 | 243 | 206 | 15 | 372 |
| Aminobacter sp. SR38 | 8053 | 4786 | 4887 | 25 | 4131 |
| Pseudomonas sp. ADPe | 524 | 670 | 1016 | 8 | 824 |
| Variovorax sp. 38R | 967 | 2699 | 1264 | 0 | 769 |
| Aestuariispira ectoiniformans | 525 | 615 | 523 | 3 | 837 |
| Xanthomonas hydrangeae | 0 | 0 | 0 | 0 | 0 |
| Shewanella sp. WPAGA9 | 10 | 10 | 9 | 0 | 10 |
| Flavobacterium sp. CS20 | 18 | 33 | 36 | 0 | 38 |
| Rhizobium sp. TH2 | 1865 | 2183 | 1764 | 37 | 2471 |
| Mesorhizobium onobrychidis | 3959 | 4199 | 3928 | 28 | 6295 |
| Skermanella sp. TT6 | 4148 | 5475 | 4859 | 135 | 9561 |
| Mycobacterium senriense | 1299 | 2920 | 2241 | 50 | 4724 |
| Stenotrophomonas sp. CW117 | 468 | 1041 | 2228 | 0 | 1887 |
| Xanthomonas sp. WG16 | 0 | 1713 | 0 | 0 | 0 |
| Pontibacillus sp. ALD_SL1 | 5 | 40 | 37 | 0 | 32 |
| Psychroflexus sp. ALD_RP9 | 3 | 17 | 17 | 1 | 26 |
| Pseudomonas sp. OST1909 | 82 | 103 | 180 | 3 | 29293 |
| Mesorhizobium sp. J8 | 2007 | 2265 | 2136 | 32 | 2265 |
| Brevibacterium sp. SMBL_HHYL_HB1 | 568 | 455 | 868 | 63 | 1222 |
| Vibrio bathopelagicus | 2 | 14 | 9 | 1 | 11 |
| Paenibacillus sp. JNUCC-31 | 84 | 106 | 125 | 0 | 147 |
| Paenibacillus sp. JNUCC-32 | 0 | 0 | 0 | 0 | 0 |
| Aeromonas sp. FDAARGOS 1402 | 63 | 161 | 233 | 0 | 77 |
| Aeromonas sp. FDAARGOS 1403 | 43 | 142 | 65 | 0 | 102 |
| Aeromonas sp. FDAARGOS 1404 | 4 | 72 | 32 | 0 | 104 |
| Aeromonas sp. FDAARGOS 1405 | 5 | 67 | 80 | 1 | 50 |
| Aeromonas sp. FDAARGOS 1406 | 0 | 36 | 115 | 0 | 81 |
| Aeromonas sp. FDAARGOS 1407 | 164 | 172 | 243 | 0 | 154 |
| Aeromonas sp. FDAARGOS 1408 | 45 | 50 | 50 | 0 | 131 |
| Aeromonas sp. FDAARGOS 1409 | 261 | 324 | 400 | 9 | 330 |
| Aeromonas sp. FDAARGOS 1410 | 56 | 50 | 83 | 87 | 51 |
| Aeromonas sp. FDAARGOS 1411 | 31 | 76 | 69 | 0 | 54 |
| Aeromonas sp. FDAARGOS 1414 | 8 | 45 | 24 | 0 | 19 |
| Aeromonas sp. FDAARGOS 1415 | 189 | 357 | 376 | 13 | 551 |
| Aeromonas sp. FDAARGOS 1416 | 0 | 27 | 46 | 0 | 68 |
| Aeromonas sp. FDAARGOS 1417 | 83 | 156 | 87 | 20 | 61 |
| Aeromonas sp. FDAARGOS 1418 | 346 | 61 | 103 | 0 | 22 |
| Aeromonas sp. FDAARGOS 1419 | 0 | 131 | 56 | 0 | 95 |
| Amycolatopsis sp. FDAARGOS 1241 | 11591 | 3625 | 11579 | 240 | 9036 |
| Rhodanobacter sp. FDAARGOS 1247 | 1274 | 1809 | 1987 | 92 | 2194 |
| Pectobacterium parvum | 41 | 47 | 39 | 0 | 85 |
| Methyloradius palustris | 31 | 160 | 89 | 5 | 136 |
| Mucilaginibacter sp. 21P | 0 | 0 | 0 | 0 | 0 |
| Rodentibacter haemolyticus | 0 | 0 | 0 | 0 | 0 |
| Zunongwangia sp. SCSIO 43204 | 42 | 50 | 83 | 4 | 33 |
| Marixanthomonas sp. SCSIO 43207 | 19 | 27 | 22 | 1 | 25 |
| Erythrobacter sp. SCSIO 43205 | 306 | 303 | 412 | 0 | 327 |
| Mesoflavibacter sp. SCSIO 43206 | 17 | 39 | 5 | 1 | 13 |
| Vibrio sp. SCSIO 43132 | 12 | 37 | 33 | 0 | 58 |
| Clostridium sp. 'deep sea' | 31 | 36 | 39 | 0 | 71 |
| Blautia liquoris | 16 | 76 | 43 | 0 | 57 |
| Thermophilibacter immobilis | 552 | 661 | 924 | 3 | 988 |
| Viridibacillus sp. JNUCC-6 | 11 | 40 | 25 | 0 | 17 |
| Sulfurovum indicum | 7 | 45 | 57 | 0 | 71 |
| Streptomyces sp. EAS-AB2608 | 986 | 885 | 1229 | 192 | 2294 |
| Ruania alkalisoli | 1037 | 978 | 1719 | 70 | 2189 |
| Luteibacter flocculans | 886 | 1106 | 1099 | 23 | 1266 |
| Solibaculum mannosilyticum | 112 | 420 | 415 | 1 | 495 |
| Lysobacter sp. H21R4 | 0 | 0 | 0 | 0 | 0 |
| Lysobacter ciconiae | 0 | 0 | 0 | 0 | 0 |
| Lysobacter avium | 491 | 582 | 322 | 97 | 345 |
| Lysobacter sp. H23M47 | 709 | 836 | 387 | 51 | 543 |
| Tenacibaculum finnmarkense | 9 | 44 | 24 | 0 | 15 |
| Streptococcus oriscaviae | 1 | 14 | 13 | 0 | 7 |
| Streptomyces sp. A2-16 | 2426 | 1757 | 3630 | 110 | 5056 |
| Saccharothrix sp. 6-C | 39196 | 9883 | 23281 | 705 | 17320 |
| Staphylococcus lloydii | 1 | 2 | 2 | 0 | 3 |
| Citrobacter sp. BDA59-3 | 0 | 0 | 0 | 0 | 0 |
| Echinicola sp. 20G | 0 | 0 | 0 | 0 | 0 |
| Agromyces archimandritae | 1432 | 1947 | 2953 | 92 | 3680 |
| Leifsonia sp. ZF2019 | 1132 | 1556 | 2090 | 70 | 2071 |
| Streptomyces sp. XC 2026 | 1043 | 832 | 1502 | 182 | 3484 |
| Mongoliitalea daihaiensis | 53 | 66 | 61 | 0 | 99 |
| Streptomyces sp. A10(2020) | 494 | 590 | 1436 | 258 | 8099 |
| Xianfuyuplasma coldseepsis | 0 | 0 | 0 | 0 | 0 |
| Microbacterium sp. A18JL241 | 1003 | 839 | 1544 | 28 | 1580 |
| Microbacterium luteum | 1099 | 1711 | 2677 | 25 | 2210 |
| Microbacterium atlanticum | 1114 | 1462 | 2204 | 26 | 2301 |
| Microbacterium cremeum | 1446 | 1672 | 2640 | 42 | 2915 |
| Orrella sp. f23 | 93 | 216 | 170 | 2 | 369 |
| Synechocystis sp. PCC 7339 | 0 | 34 | 55 | 0 | 12 |
| Bradyrhizobium sp. 131 | 784 | 889 | 1220 | 0 | 0 |
| Bradyrhizobium sp. 155 | 0 | 0 | 0 | 0 | 0 |
| Bradyrhizobium sp. 170 | 123552 | 127162 | 60877 | 698 | 64167 |
| Bradyrhizobium sp. 172 | 1139 | 1556 | 2060 | 0 | 289 |
| Bradyrhizobium sp. 183 | 0 | 0 | 0 | 0 | 8374 |
| Bradyrhizobium sp. 184 | 21713 | 0 | 0 | 0 | 18288 |
| Bradyrhizobium sp. 186 | 8819 | 7833 | 5798 | 55 | 4826 |
| Bradyrhizobium sp. 187 | 0 | 0 | 0 | 0 | 0 |
| Bradyrhizobium sp. 191 | 2493 | 1582 | 1207 | 0 | 0 |
| Bradyrhizobium sp. 192 | 2425 | 2349 | 1800 | 34 | 2276 |
| Bradyrhizobium sp. 195 | 3832 | 3230 | 2431 | 47 | 2313 |
| Bradyrhizobium sp. 200 | 86280 | 74762 | 39647 | 485 | 41668 |
| Bradyrhizobium sp. 40 | 2293 | 877 | 1602 | 0 | 0 |
| Bradyrhizobium sp. 4 | 2658 | 1259 | 1595 | 0 | 501 |
| Bradyrhizobium sp. CW1 | 1884 | 3144 | 2454 | 153 | 2582 |
| Anaeromicropila herbilytica | 21 | 13 | 14 | 0 | 3 |
| Rhizobium sp. 007 | 717 | 1069 | 592 | 0 | 137 |
| Vibrio sp. ED002 | 0 | 2 | 1 | 0 | 9 |
| Vibrio sp. ED004 | 4 | 26 | 14 | 0 | 23 |
| Acinetobacter sp. SK-43 | 0 | 0 | 0 | 0 | 1 |
| Entomomonas asaccharolytica | 0 | 0 | 0 | 0 | 0 |
| Bacteroides sp. HF-162 | 0 | 0 | 14 | 0 | 0 |
| Serratia sp. CMO1 | 22 | 18 | 0 | 0 | 40 |
| Vibrio sp. VB16 | 3 | 30 | 14 | 2 | 15 |
| Methylomonas sp. LL1 | 309 | 430 | 467 | 15 | 592 |
| Erythrobacter sp. A30-3 | 0 | 0 | 0 | 0 | 0 |
| Thalassospira sp. A40-3 | 137 | 207 | 175 | 8 | 366 |
| Halomonas sp. A40-4 | 286 | 235 | 251 | 18 | 271 |
| Pseudoalteromonas sp. A41-2 | 39 | 9 | 8 | 0 | 35 |
| Thalassospira sp. B30-1 | 211 | 261 | 253 | 0 | 481 |
| Sulfitobacter sp. B30-2 | 56 | 64 | 86 | 0 | 109 |
| Alteromonas sp. B31-7 | 0 | 16 | 0 | 0 | 20 |
| Salinimonas marina | 30 | 66 | 145 | 2 | 122 |
| Treponema peruense | 26 | 17 | 32 | 0 | 36 |
| Pantoea sp. SM3640 | 0 | 113 | 70 | 0 | 81 |
| Klebsiella sp. BDA134-6 | 5 | 14 | 0 | 0 | 4 |
| Labrys sp. KNU-23 | 2883 | 2543 | 2358 | 25 | 3166 |
| Sphingobium sp. Cam5-1 | 565 | 824 | 708 | 0 | 909 |
| Rhodococcus sp. USK10 | 1336 | 2036 | 2182 | 97 | 2987 |
| Pedobacter endophyticus | 30 | 144 | 200 | 6 | 358 |
| Actinomarinicola tropica | 8754 | 6558 | 14888 | 56 | 11765 |
| Pontivivens ytuae | 1145 | 1313 | 1514 | 11 | 1578 |
| Synechococcus sp. CBW1107 | 455 | 907 | 878 | 20 | 972 |
| Thiomicrorhabdus immobilis | 5 | 21 | 24 | 2 | 38 |
| Pseudonocardia abyssalis | 5527 | 4238 | 10476 | 160 | 9304 |
| Pseudonocardia oceani | 18 | 49 | 80 | 0 | 48 |
| Spiroplasma endosymbiont of 'Nebria riversi' | 4 | 0 | 3 | 0 | 3 |
| Diaphorobacter sp. JS3051 | 0 | 0 | 0 | 0 | 0 |
| Acidihalobacter aeolianus | 972 | 1570 | 1574 | 2 | 1874 |
| Bacteroides humanifaecis | 0 | 20 | 16 | 0 | 58 |
| Gardnerella piotii | 0 | 0 | 0 | 0 | 0 |
| Gardnerella leopoldii | 2 | 8 | 7 | 0 | 20 |
| Gardnerella swidsinskii | 13 | 17 | 13 | 0 | 29 |
| Ruegeria sp. SCSIO 43209 | 235 | 255 | 303 | 8 | 358 |
| Pseudomonas sp. IAC-BECa141 | 45 | 69 | 87 | 2 | 177 |
| Schaalia sp. 19OD2882 | 337 | 550 | 766 | 13 | 882 |
| Spongiibacter nanhainus | 162 | 318 | 314 | 3 | 376 |
| Rhodococcus sp. P-2 | 132 | 220 | 316 | 0 | 465 |
| Brachybacterium halotolerans | 996 | 999 | 1708 | 7 | 1552 |
| Rhizobium sp. AB2/73 | 951 | 826 | 663 | 0 | 695 |
| Desulfolithobacter dissulfuricans | 0 | 0 | 0 | 0 | 0 |
| Methylacidiphilum sp. IT5 | 6 | 142 | 42 | 0 | 50 |
| Methylacidiphilum sp. IT6 | 45 | 28 | 30 | 0 | 43 |
| Lysobacter arenosi | 1904 | 2084 | 1722 | 30 | 1642 |
| Denitromonas sp. IR12 | 25 | 14 | 19 | 0 | 7 |
| Curtobacterium sp. YC1 | 747 | 940 | 1339 | 79 | 1534 |
| Thiohalobacter sp. COW1 | 552 | 1343 | 1125 | 24 | 1681 |
| Kaistia sp. 32K | 5150 | 4695 | 4176 | 111 | 5678 |
| Pseudomonas sp. MPFS | 175 | 132 | 111 | 0 | 225 |
| Sphingobacterium sp. UDSM-2020 | 20 | 112 | 64 | 12 | 139 |
| Stieleria maiorica | 1919 | 5480 | 3819 | 78 | 6573 |
| Methylacidimicrobium sp. B4 | 608 | 1501 | 1036 | 11 | 1382 |
| Elizabethkingia sp. M8 | 0 | 0 | 0 | 0 | 3 |
| Bacillus sp. HNR-4 | 0 | 10 | 0 | 0 | 8 |
| Alicyclobacillus sp. ALC3 | 262 | 517 | 504 | 3 | 673 |
| Comamonas fluminis | 0 | 0 | 0 | 0 | 0 |
| Brevibacillus composti | 230 | 563 | 605 | 12 | 712 |
| Gelria sp. Kuro-4 | 563 | 821 | 1069 | 5 | 1170 |
| Streptomyces sp. HSG2 | 1606 | 1427 | 2476 | 118 | 4787 |
| Streptomyces liliifuscus | 4792 | 3791 | 6059 | 320 | 7562 |
| Breznakiella homolactica | 100 | 166 | 211 | 1 | 232 |
| Croceicoccus sp. YJ47 | 889 | 1126 | 1149 | 11 | 1259 |
| Rhodoferax sp. MIZ03 | 0 | 0 | 0 | 0 | 0 |
| Acinetobacter sp. CS-2 | 42 | 81 | 56 | 0 | 134 |
| Pseudomonas sp. Eqa60 | 19 | 111 | 95 | 12 | 170 |
| Luteimonas sp. MC1572 | 1943 | 1604 | 1539 | 22 | 1679 |
| Luteimonas sp. MC1750 | 1816 | 1457 | 1600 | 0 | 1366 |
| Raoultella sp. XY-1 | 0 | 0 | 339 | 0 | 0 |
| Pseudoalteromonas sp. LC2018020214 | 2 | 2 | 0 | 0 | 10 |
| Staphylococcus sp. 11-B-312 | 0 | 0 | 0 | 0 | 0 |
| Staphylococcus sp. T93 | 3002 | 0 | 0 | 0 | 0 |
| Carnobacterium sp. CS13 | 1 | 12 | 2 | 0 | 2 |
| Brevundimonas vitisensis | 0 | 0 | 0 | 0 | 0 |
| Streptomyces sp. CA-210063 | 2982 | 2172 | 3271 | 538 | 12721 |
| Streptomyces sp. DSM 40750 | 1917 | 2034 | 3165 | 124 | 5481 |
| Streptomyces sp. CA-256286 | 268 | 434 | 857 | 15 | 2199 |
| Devosia oryziradicis | 1301 | 1167 | 975 | 0 | 1444 |
| Mycoplasma sp. E35C | 0 | 1 | 7 | 0 | 20 |
| Vibrio sp. SCSIO 43133 | 0 | 10 | 2 | 0 | 7 |
| Kutzneria sp. CA-103260 | 20474 | 5660 | 18838 | 610 | 18166 |
| Luteitalea sp. TBR-22 | 23239 | 62112 | 40878 | 270 | 40800 |
| Desulfuromonas versatilis | 1356 | 2626 | 2753 | 20 | 3282 |
| Leptolyngbya sp. Cla-17 | 8 | 57 | 28 | 0 | 76 |
| Nocardioides sp. zg-536 | 2270 | 3007 | 3722 | 96 | 3731 |
| Shewanella sp. KX20019 | 7 | 35 | 41 | 8 | 44 |
| Curtobacterium sp. 24E2 | 555 | 763 | 1092 | 61 | 1385 |
| Shewanella sp. LZH-2 | 189 | 5 | 14 | 0 | 8 |
| Caldicellulosiruptor diazotrophicus | 2 | 3 | 25 | 0 | 16 |
| Flagellatimonas centrodinii | 1042 | 1346 | 1202 | 10 | 1524 |
| Hydrogenophaga sp. YM1 | 1621 | 1777 | 1965 | 9 | 2866 |
| Paludibacterium sp. B53371 | 367 | 591 | 560 | 0 | 1013 |
| Rhizobium sp. SL42 | 891 | 973 | 806 | 22 | 1179 |
| Hydrogenophaga sp. SL48 | 0 | 0 | 0 | 0 | 0 |
| Devosia sp. SL43 | 1365 | 1399 | 1156 | 15 | 1661 |
| Rhodococcus sp. USK13 | 868 | 984 | 1415 | 22 | 1670 |
| Turicibacter sp. TJ11 | 22 | 2 | 8 | 0 | 11 |
| Polaribacter sp. Q13 | 57 | 54 | 41 | 0 | 36 |
| Bulleidia sp. zg-1006 | 22 | 55 | 94 | 0 | 93 |
| Candidatus Gromoviella agglomerans | 23 | 24 | 38 | 1 | 49 |
| Metabacillus sp. cB07 | 69 | 166 | 94 | 0 | 262 |
| Neotabrizicola shimadae | 984 | 1263 | 1330 | 3 | 1684 |
| Microvirga sp. VF16 | 3343 | 3210 | 2961 | 42 | 4638 |
| Humisphaera borealis | 2966 | 10084 | 7022 | 89 | 10123 |
| Pseudomonas sp. MIL9 | 15 | 13 | 11 | 0 | 35 |
| Glaciimonas sp. PAMC28666 | 183 | 380 | 282 | 0 | 508 |
| Polaribacter batillariae | 1 | 15 | 17 | 1 | 21 |
| Paralysiella testudinis | 63 | 193 | 167 | 4 | 287 |
| Bacillus sp. LJBS06 | 0 | 25 | 0 | 0 | 0 |
| Salinimicrobium sp. HN-2-9-2 | 44 | 61 | 104 | 0 | 148 |
| Microbacterium sp. EF45047 | 571 | 1067 | 1763 | 76 | 1612 |
| Acinetobacter sp. Colony158 | 0 | 4 | 24 | 0 | 4 |
| Paenibacillus tianjinensis | 50 | 173 | 158 | 4 | 277 |
| Mycoplasma sp. SG1 | 3 | 0 | 10 | 0 | 21 |
| Fulvivirga lutea | 29 | 101 | 44 | 0 | 35 |
| Agrococcus sp. Marseille-Q4369 | 739 | 1096 | 1415 | 0 | 813 |
| Sporosarcina sp. Marseille-Q4063 | 13 | 180 | 52 | 0 | 77 |
| Rothia sp. ZJ932 | 78 | 101 | 150 | 12 | 174 |
| Microbacterium sp. EF45044 | 935 | 822 | 1518 | 0 | 1549 |
| Desulfoluna sp. ASN36 | 354 | 1000 | 759 | 2 | 906 |
| Pseudodesulfovibrio sediminis | 311 | 373 | 376 | 8 | 603 |
| Natronoglycomyces albus | 381 | 494 | 1218 | 34 | 1496 |
| Natronosporangium hydrolyticum | 2530 | 1991 | 5888 | 175 | 6707 |
| Niveibacterium microcysteis | 1104 | 1327 | 1595 | 8 | 2243 |
| Rhodococcus pseudokoreensis | 1193 | 1729 | 2192 | 76 | 2736 |
| Pseudomonas sp. PDNC002 | 0 | 0 | 0 | 0 | 0 |
| Ensifer sp. PDNC004 | 1361 | 1286 | 1248 | 47 | 1339 |
| Gordonia sp. PDNC005 | 1270 | 1067 | 1616 | 14 | 1754 |
| Variovorax sp. PDNC026 | 1419 | 1443 | 1858 | 0 | 2815 |
| Thermoleptolyngbya sp. PKUAC-SCTB121 | 24 | 299 | 290 | 0 | 195 |
| Fenollaria sporofastidiosus | 0 | 0 | 0 | 0 | 0 |
| Burkholderia sp. MS455 | 0 | 0 | 0 | 0 | 0 |
| Burkholderia sp. MS389 | 309 | 167 | 374 | 0 | 548 |
| Pseudomonas sp. SDM007 | 0 | 0 | 0 | 0 | 0 |
| Conexibacter sp. SYSU D00693 | 10318 | 8294 | 21581 | 155 | 23796 |
| Methylomonas sp. EFPC1 | 164 | 299 | 280 | 27 | 330 |
| Methylococcus sp. EFPC2 | 806 | 1828 | 1341 | 75 | 1815 |
| Bacillus sp. PDNC022 | 0 | 0 | 6 | 0 | 0 |
| Rhodococcus sp. PSBB049 | 108 | 347 | 786 | 14 | 605 |
| Rhodococcus sp. PSBB066 | 0 | 0 | 6530 | 0 | 0 |
| Leptolyngbya sp. 7M | 1099 | 5999 | 4741 | 53 | 4400 |
| Terrisporobacter hibernicus | 35 | 30 | 11 | 0 | 11 |
| Myxococcus sp. SCHIC003 | 1234 | 2322 | 2244 | 10 | 2691 |
| Pyxidicoccus sp. SCPEA002 | 3475 | 7862 | 6291 | 71 | 7383 |
| Thermosynechococcus sp. TA-1 | 28 | 87 | 81 | 0 | 196 |
| Leucobacter sp. CX169 | 718 | 836 | 1199 | 29 | 1471 |
| Methylobacterium sp. OT2 | 0 | 0 | 0 | 0 | 0 |
| Pseudomonas sp. SORT22 | 113 | 191 | 166 | 8 | 319 |
| Burkholderia sp. LAS2 | 0 | 0 | 0 | 0 | 0 |
| Shewanella cyperi | 105 | 323 | 165 | 2 | 197 |
| Shewanella sedimentimangrovi | 29 | 116 | 144 | 6 | 154 |
| Shewanella avicenniae | 27 | 97 | 91 | 9 | 92 |
| Caballeronia sp. M1242 | 585 | 797 | 858 | 5 | 1045 |
| Nostoc sp. UHCC 0702 | 169 | 473 | 321 | 2 | 690 |
| Geobacter benzoatilyticus | 436 | 944 | 744 | 14 | 1047 |
| Nitrogeniibacter aestuarii | 773 | 932 | 966 | 1 | 1270 |
| Corynebacterium sp. CNCTC7651 | 0 | 0 | 0 | 0 | 0 |
| Alkalibacter rhizosphaerae | 25 | 91 | 101 | 4 | 104 |
| Pseudomonas germanica | 120 | 91 | 108 | 0 | 487 |
| Pseudomonas sp. PP3 | 0 | 0 | 0 | 0 | 0 |
| Flavobacterium endoglycinae | 37 | 231 | 209 | 0 | 91 |
| Aureimonas sp. OT7 | 2854 | 2960 | 2961 | 93 | 3355 |
| Arthrobacter sunyaminii | 497 | 581 | 853 | 8 | 1067 |
| Sedimentibacter sp. zth1 | 74 | 36 | 41 | 0 | 45 |
| Lactococcus sp. LG606 | 0 | 0 | 0 | 0 | 0 |
| Ottowia testudinis | 667 | 887 | 922 | 0 | 1236 |
| Cellulomonas wangleii | 1442 | 2120 | 2675 | 49 | 3194 |
| Arthrobacter jiangjiafuii | 459 | 584 | 898 | 46 | 1182 |
| Marinobacter sp. CA1 | 393 | 450 | 520 | 12 | 787 |
| Gilliamella sp. B3022 | 0 | 6 | 3 | 9 | 4 |
| Sulfidibacter corallicola | 1406 | 3395 | 3296 | 28 | 3332 |
| Sporosarcina sp. Te-1 | 21 | 53 | 58 | 0 | 99 |
| Polaribacter cellanae | 1 | 28 | 15 | 1 | 38 |
| Vibrio sp. SCSIO 43135 | 49 | 88 | 45 | 15 | 77 |
| Vibrio sp. SCSIO 43153 | 2 | 25 | 9 | 1 | 8 |
| Vibrio sp. SCSIO 43155 | 4 | 4 | 6 | 0 | 4 |
| Vibrio sp. SCSIO 43140 | 7 | 32 | 21 | 0 | 51 |
| Vibrio sp. SCSIO 43136 | 25 | 43 | 65 | 0 | 48 |
| Vibrio sp. SCSIO 43009 | 0 | 24 | 0 | 0 | 0 |
| Vibrio sp. SCSIO 43097 | 0 | 0 | 0 | 0 | 24 |
| Pengzhenrongella sicca | 2257 | 2849 | 4040 | 198 | 5579 |
| Acidihalobacter yilgarnensis | 641 | 1082 | 894 | 18 | 1181 |
| Streptococcus toyakuensis | 0 | 0 | 0 | 0 | 0 |
| Cellulomonas fengjieae | 1765 | 2213 | 3112 | 102 | 3673 |
| Cellulomonas dongxiuzhuiae | 1201 | 1501 | 2262 | 95 | 2408 |
| Rhizobium sp. B21/90 | 31 | 155 | 153 | 0 | 114 |
| Rhizobium sp. B230/85 | 0 | 0 | 0 | 0 | 0 |
| Rhizobium sp. K15/93 | 0 | 0 | 2303 | 0 | 0 |
| Rhizobium sp. L51/94 | 821 | 471 | 414 | 11 | 544 |
| Agrobacterium sp. S7/73 | 0 | 0 | 0 | 0 | 0 |
| Candidatus Kaistella beijingensis | 29 | 52 | 22 | 0 | 28 |
| Aquabacter sp. L1I39 | 2345 | 1194 | 1401 | 0 | 1060 |
| Candidatus Arcticimaribacter forsetii | 0 | 0 | 0 | 0 | 0 |
| Psychrosphaera ytuae | 3 | 22 | 39 | 5 | 45 |
| Lysobacter sp. K5869 | 1167 | 1601 | 1309 | 24 | 1491 |
| Aggregatibacter sp. 2125159857 | 158 | 36 | 40 | 4 | 14 |
| Spiractinospora alimapuensis | 1275 | 1500 | 2981 | 331 | 6664 |
| Brucella sp. 458 | 20 | 67 | 59 | 0 | 88 |
| Bacillus sp. LJBV19 | 0 | 0 | 0 | 0 | 16 |
| Chloracidobacterium sp. D | 193 | 350 | 365 | 0 | 1115 |
| Chloracidobacterium validum | 480 | 2300 | 1437 | 43 | 1758 |
| Pseudomonas sp. IsoF | 0 | 0 | 0 | 0 | 0 |
| Lysobacter luteus | 1737 | 1665 | 1581 | 0 | 1534 |
| Rhodoferax sp. PAMC 29310 | 352 | 795 | 590 | 11 | 918 |
| Methylovirgula sp. HY1 | 1442 | 1310 | 1039 | 0 | 1534 |
| Pseudoalteromonas sp. SCSIO 43201 | 18 | 121 | 60 | 3 | 54 |
| Ferrimonas sp. SCSIO 43195 | 0 | 0 | 0 | 0 | 0 |
| Pseudoalteromonas sp. SCSIO 43088 | 12 | 19 | 16 | 0 | 34 |
| Pseudoalteromonas sp. SCSIO 43101 | 1 | 35 | 27 | 3 | 37 |
| Roseovarius sp. SCSIO 43702 | 926 | 1090 | 983 | 20 | 1177 |
| Streptomyces liliiviolaceus | 137 | 34 | 154 | 0 | 88 |
| Faecalicatena sp. Marseille-Q4148 | 23 | 22 | 49 | 0 | 37 |
| Phascolarctobacterium sp. Marseille-Q4147 | 0 | 0 | 0 | 0 | 0 |
| Brevibacterium sp. W7.2 | 1485 | 1142 | 2176 | 57 | 2682 |
| Caulobacter sp. S6 | 2033 | 2964 | 2471 | 17 | 3381 |
| Pseudomonas sp. Tri1 | 0 | 0 | 0 | 0 | 0 |
| Candidatus Thiothrix anitrata | 35 | 192 | 137 | 11 | 212 |
| Paenibacillus sp. S02 | 93 | 45 | 66 | 0 | 63 |
| Paenibacillus sp. S25 | 0 | 62 | 17 | 0 | 57 |
| Luteolibacter ambystomatis | 998 | 3049 | 2275 | 27 | 2877 |
| Streptomyces sp. Go40/10 | 2263 | 1764 | 3158 | 141 | 3578 |
| Chryseobacterium sp. PCH239 | 7 | 23 | 22 | 0 | 37 |
| Pseudomonas sp. SCA2728.1_7 | 0 | 0 | 0 | 0 | 0 |
| Aureimonas sp. SA4125 | 3092 | 2713 | 2648 | 57 | 3427 |
| Pasteurella atlantica | 15 | 7 | 2 | 0 | 7 |
| Thermospira aquatica | 24 | 115 | 93 | 0 | 139 |
| Providencia sp. R33 | 8 | 41 | 111 | 9 | 109 |
| Arthrobacter sp. Helios | 647 | 618 | 986 | 34 | 1549 |
| Psychrobacillus sp. INOP01 | 14 | 91 | 30 | 1 | 35 |
| Pseudomonas sp. JS425 | 0 | 45 | 169 | 0 | 319 |
| Tessaracoccus palaemonis | 1080 | 1234 | 1627 | 54 | 1956 |
| Erythrobacter sp. JK5 | 791 | 700 | 987 | 49 | 1161 |
| Photobacterium sp. GJ3 | 0 | 0 | 0 | 0 | 0 |
| Bacterioplanoides sp. SCSIO 12839 | 49 | 87 | 60 | 4 | 82 |
| Kordiimonas sp. SCSIO 12603 | 34 | 181 | 72 | 0 | 206 |
| Kordiimonas sp. SCSIO 12610 | 29 | 88 | 44 | 1 | 95 |
| Parvicella tangerina | 36 | 22 | 18 | 0 | 36 |
| Shewanella yunxiaonensis | 35 | 91 | 83 | 3 | 206 |
| Proteiniphilum propionicum | 35 | 32 | 29 | 2 | 88 |
| Sphingopyxis sp. USTB-05 | 799 | 1644 | 1079 | 1 | 1226 |
| Dysosmobacter sp. Marseille-Q4140 | 345 | 679 | 770 | 4 | 779 |
| Flavobacterium sp. KK2020170 | 17 | 62 | 33 | 1 | 35 |
| Pseudomonas nanhaiensis | 506 | 701 | 623 | 17 | 798 |
| Austwickia sp. TVS 96-490-7B | 0 | 0 | 2 | 0 | 0 |
| Arthrobacter sp. NtRootA1 | 315 | 779 | 1249 | 39 | 2264 |
| Arthrobacter sp. StoSoilA2 | 167 | 662 | 1065 | 197 | 2806 |
| Arthrobacter sp. StoSoilB5 | 638 | 956 | 1525 | 21 | 2451 |
| Arthrobacter sp. StoSoilB13 | 359 | 602 | 836 | 76 | 2024 |
| Arthrobacter sp. StoSoilB19 | 900 | 920 | 1478 | 0 | 1873 |
| Arthrobacter sp. StoSoilB20 | 311 | 541 | 2516 | 69 | 1597 |
| Arthrobacter sp. NicSoilB4 | 821 | 1847 | 3065 | 74 | 4431 |
| Arthrobacter sp. NicSoilB8 | 1263 | 4194 | 10108 | 22 | 9754 |
| Arthrobacter sp. NicSoilB11 | 128 | 737 | 1006 | 0 | 1231 |
| Arthrobacter sp. NicSoilC5 | 484 | 706 | 1285 | 52 | 2564 |
| Paraneptunicella aestuarii | 58 | 143 | 60 | 6 | 77 |
| Streptomyces sp. V17-9 | 689 | 549 | 848 | 12 | 836 |
| Gordonia pseudamarae | 1046 | 1415 | 1832 | 90 | 3457 |
| Virgibacillus sp. NKC19-16 | 21 | 41 | 25 | 0 | 31 |
| Enterobacter sp. JBIWA003 | 10 | 69 | 87 | 0 | 83 |
| Enterobacter sp. JBIWA005 | 9 | 48 | 110 | 0 | 74 |
| Enterobacter sp. JBIWA008 | 0 | 0 | 29 | 0 | 46 |
| Serratia sp. JSRIV001 | 58 | 62 | 26 | 0 | 46 |
| Serratia sp. JSRIV002 | 9 | 64 | 151 | 2 | 117 |
| Serratia sp. JSRIV004 | 19 | 25 | 46 | 0 | 33 |
| Serratia sp. JSRIV006 | 18 | 29 | 100 | 0 | 37 |
| Nocardiopsis sp. HDS12 | 1222 | 1102 | 5767 | 863 | 15816 |
| Nocardiopsis sp. Mg02 | 2298 | 1201 | 0 | 0 | 0 |
| Halomonas sp. FeN2 | 0 | 0 | 0 | 0 | 0 |
| Streptomyces sp. MST-110588 | 2013 | 1681 | 3472 | 241 | 6125 |
| Pseudomonas sp. RC3H12 | 221 | 264 | 221 | 0 | 291 |
| Allobaculum mucilyticum | 66 | 74 | 80 | 0 | 84 |
| Pseudochrobactrum algeriensis | 0 | 0 | 0 | 0 | 0 |
| Klebsiella sp. A52 | 0 | 0 | 0 | 0 | 0 |
| Bdellovibrio reynosensis | 0 | 0 | 0 | 0 | 0 |
| Streptomyces sp. EMB24 | 638 | 692 | 1343 | 86 | 2339 |
| Hymenobacter sp. BT18 | 192 | 470 | 383 | 14 | 642 |
| Polymorphobacter megasporae | 725 | 1032 | 929 | 0 | 1035 |
| Paenibacillus sp. 481 | 64 | 117 | 159 | 0 | 168 |
| Enterobacter sp. SGAir0187 | 94 | 88 | 80 | 0 | 34 |
| Acinetobacter sp. BHS4 | 0 | 2 | 6 | 0 | 0 |
| Actinoplanes sp. L3-i22 | 6177 | 4216 | 10401 | 404 | 12062 |
| Streptomyces sp. FIT100 | 1663 | 1459 | 3284 | 301 | 6756 |
| Pseudomonas fitomaticsae | 171 | 111 | 143 | 4 | 218 |
| Streptomyces sp. MG62 | 6429 | 2983 | 51235 | 0 | 9627 |
| Flavihumibacter rivuli | 186 | 319 | 327 | 11 | 423 |
| Flavihumibacter fluminis (ex Park et al. 2022) | 351 | 726 | 422 | 5 | 520 |
| Streptomyces sp. 135 | 1671 | 1600 | 2704 | 117 | 4394 |
| Chryseobacterium sp. ZHDP1 | 0 | 0 | 0 | 0 | 0 |
| Methylobacter sp. S3L5C | 76 | 204 | 173 | 2 | 236 |
| Pseudoalteromonas sp. SiA1 | 0 | 4 | 42 | 0 | 26 |
| Enterococcus innesii | 29 | 4 | 6 | 0 | 1 |
| Cupriavidus sp. EM10 | 1354 | 2208 | 2595 | 46 | 3618 |
| Campylobacter sp. 19-13652 | 1 | 29 | 12 | 0 | 9 |
| Gemella sp. zg-570 | 11 | 21 | 7 | 1 | 18 |
| Streptomyces sp. YPW6 | 972 | 1039 | 1916 | 198 | 4323 |
| Brucella sp. BTU1 | 206 | 223 | 176 | 43 | 197 |
| Nocardioides sp. LMS-CY | 3368 | 5742 | 7089 | 244 | 8126 |
| Mycoavidus sp. HKI | 71 | 102 | 151 | 3 | 141 |
| Asticcacaulis sp. AND118 | 673 | 805 | 783 | 26 | 1082 |
| Bradyrhizobium sediminis | 27298 | 25751 | 15580 | 0 | 14121 |
| Gemmobacter fulva | 766 | 604 | 819 | 14 | 1221 |
| Eubacterium sp. c-25 | 15 | 61 | 34 | 0 | 33 |
| Pseudomonas sp. SK2 | 27 | 154 | 42 | 0 | 193 |
| Pseudomonas sp. SK3(2021) | 251 | 273 | 241 | 0 | 383 |
| Candidatus Minimicrobia vallesae | 6 | 41 | 28 | 0 | 49 |
| Eubacterium sp. MSJ-33 | 5 | 29 | 24 | 0 | 44 |
| Curtobacterium sp. L6-1 | 1035 | 1306 | 1781 | 65 | 2207 |
| Bacillus sp. ZHX3 | 0 | 0 | 0 | 0 | 938 |
| Streptomyces sp. G11C(2021) | 421 | 872 | 1611 | 26 | 1187 |
| Vibrio ostreae | 116 | 125 | 119 | 0 | 164 |
| Streptomyces sp. 4503 | 47 | 7 | 54 | 0 | 75 |
| Pseudomonas siliginis | 0 | 0 | 0 | 0 | 0 |
| Pseudomonas alvandae | 201 | 145 | 133 | 11 | 344 |
| Pseudomonas asgharzadehiana | 0 | 0 | 0 | 0 | 0 |
| Pseudomonas azerbaijanoriens | 262 | 198 | 268 | 0 | 559 |
| Pseudomonas maumuensis | 221 | 383 | 235 | 0 | 425 |
| Pseudomonas fakonensis | 313 | 288 | 400 | 2 | 433 |
| Pseudomonas xanthosomae | 346 | 327 | 368 | 0 | 446 |
| Pseudomonas muyukensis | 0 | 0 | 0 | 0 | 0 |
| Amycolatopsis aidingensis | 11983 | 2876 | 11165 | 313 | 9801 |
| Bacillus sp. JNUCC-22 | 0 | 0 | 0 | 0 | 0 |
| Miltoncostaea marina | 6874 | 7084 | 12042 | 153 | 17330 |
| Miltoncostaea oceani | 5909 | 6111 | 10371 | 84 | 14872 |
| Clostridium sp. CF011 | 6 | 12 | 0 | 0 | 0 |
| Elioraea tepida | 2230 | 2441 | 2367 | 109 | 4039 |
| Radiobacillus kanasensis | 30 | 29 | 26 | 0 | 24 |
| Collinsella sp. zg1085 | 7 | 52 | 50 | 0 | 72 |
| Flavobacterium sp. CECT 9288 | 6 | 253 | 100 | 0 | 53 |
| Subtercola sp. PAMC28395 | 526 | 611 | 919 | 10 | 743 |
| Bacillus sp. NP247 | 20 | 16 | 0 | 0 | 8 |
| Geoalkalibacter halelectricus | 533 | 1298 | 1398 | 5 | 1543 |
| Bacteroides sp. DH3716P | 73 | 182 | 102 | 0 | 85 |
| Atribacter laminatus | 56 | 89 | 96 | 9 | 118 |
| Geomonas subterranea | 785 | 1959 | 1791 | 37 | 2112 |
| Geomonas nitrogeniifigens | 932 | 2161 | 2149 | 44 | 2611 |
| Geomonas oryzisoli | 986 | 1780 | 2028 | 7 | 2928 |
| Rhizobium sp. WYJ-E13 | 1090 | 1151 | 936 | 46 | 1399 |
| Nocardioides panacis | 4075 | 6045 | 7710 | 254 | 8429 |
| Helicobacter sp. NHP19-0003 | 6 | 15 | 24 | 0 | 33 |
| Helicobacter sp. NHP19-0012 | 26 | 47 | 121 | 0 | 81 |
| Chitinophaga sp. MD30B | 187 | 183 | 0 | 0 | 307 |
| Aquihabitans sp. G128 | 9413 | 6827 | 16748 | 137 | 12454 |
| Clostridium sp. CM027 | 0 | 7 | 0 | 0 | 0 |
| Terribacillus sp. DMT04 | 17 | 20 | 15 | 0 | 17 |
| Enterobacter cloacae complex sp. ECL78 | 0 | 0 | 0 | 0 | 0 |
| Enterobacter cloacae complex sp. ECL112 | 0 | 1651 | 613 | 0 | 0 |
| Pseudomonas sp. HS6 | 149 | 274 | 256 | 2 | 455 |
| Streptomyces sp. HNA39 | 781 | 703 | 1424 | 66 | 2065 |
| Bacillus sp. 7D3 | 0 | 0 | 0 | 0 | 48 |
| Klebsiella sp. PL-2018 | 0 | 268 | 23 | 0 | 31 |
| Modestobacter sp. L9-4 | 2158 | 2716 | 4264 | 125 | 5642 |
| Pseudarthrobacter sp. L1SW | 446 | 792 | 1319 | 27 | 2262 |
| Microbacterium sp. KSW4-10 | 232 | 319 | 760 | 17 | 607 |
| Microbacterium sp. SSW1-36 | 593 | 1077 | 1519 | 43 | 1970 |
| Microbacterium sp. SSW1-51 | 1212 | 1069 | 1900 | 17 | 1907 |
| Chloracidobacterium aggregatum | 1628 | 2790 | 2782 | 32 | 3827 |
| Massilia sp. HC52 | 1512 | 4475 | 3801 | 0 | 11760 |
| Halomonas profundi | 0 | 0 | 0 | 0 | 0 |
| Acinetobacter sp. F9 | 0 | 17 | 5 | 0 | 1 |
| Pseudomonas pergaminensis | 56 | 98 | 128 | 0 | 432 |
| Comamonas sp. NLF-1-9 | 649 | 1036 | 802 | 14 | 1708 |
| Calothrix sp. PCC 7716 | 166 | 569 | 293 | 8 | 798 |
| Comamonas sp. Y33R10-2 | 152 | 325 | 193 | 2 | 283 |
| Paeniglutamicibacter sp. Y32M11 | 0 | 0 | 0 | 0 | 0 |
| Allobaculum sp. Allo2 | 40 | 110 | 101 | 10 | 107 |
| Sphingobacterium sp. E70 | 0 | 0 | 0 | 0 | 0 |
| Pseudomonas sp. PD9R | 0 | 0 | 0 | 0 | 2 |
| Nitratireductor rhodophyticola | 0 | 0 | 0 | 0 | 0 |
| Halomonas sp. SS10-MC5 | 0 | 0 | 0 | 0 | 0 |
| Porphyrobacter sp. ULC335 | 657 | 583 | 574 | 9 | 1120 |
| Burkholderia sp. EMB26 | 264 | 68 | 334 | 26 | 248 |
| Rhodococcus sp. 11-3 | 1203 | 514 | 606 | 177 | 3237 |
| Crassaminicella sp. 143-21 | 37 | 32 | 20 | 0 | 35 |
| Pseudomonas sp. AO-1 | 307 | 281 | 226 | 0 | 412 |
| Halomonas sp. 18071143 | 0 | 0 | 0 | 0 | 0 |
| Mycolicibacter sp. MYC123 | 677 | 1451 | 1135 | 102 | 2032 |
| Mycolicibacter sp. MYC340 | 1258 | 2385 | 1981 | 117 | 3669 |
| Rahnella sp. PD12R | 9 | 2 | 0 | 0 | 0 |
| Streptomyces sp. WY228 | 1358 | 1365 | 1995 | 118 | 3357 |
| Polaribacter sp. NJDZ03 | 8 | 6 | 10 | 0 | 22 |
| Bacillus sp. FDAARGOS_1420 | 16 | 1 | 0 | 0 | 1 |
| Flavobacterium litorale | 20 | 77 | 20 | 0 | 24 |
| Chryseobacterium sp. D764 | 59 | 25 | 47 | 0 | 19 |
| Pedobacter sp. D749 | 0 | 0 | 0 | 0 | 0 |
| Pseudomonas sp. HD6515 | 21 | 316 | 416 | 0 | 493 |
| Rickettsiella endosymbiont of Dermanyssus gallinae | 28 | 101 | 67 | 0 | 22 |
| Gymnodinialimonas ceratoperidinii | 668 | 802 | 814 | 80 | 1003 |
| Rhodococcus sp. LW-XY12 | 476 | 488 | 468 | 0 | 812 |
| Mycobacterium sp. SMC-2 | 1817 | 2948 | 2222 | 13 | 4556 |
| Mycobacterium sp. SMC-4 | 2051 | 5903 | 4518 | 45 | 9867 |
| Mycobacterium sp. SMC-8 | 2683 | 6704 | 5339 | 184 | 12371 |
| Pseudemcibacter aquimaris | 0 | 0 | 0 | 0 | 0 |
| Anthocerotibacter panamensis | 156 | 441 | 563 | 0 | 839 |
| Pseudomonas canavaninivorans | 0 | 0 | 0 | 0 | 0 |
| Mycetocola spongiae | 350 | 653 | 762 | 39 | 1110 |
| Bacillus sp. LJBS17 | 0 | 13 | 0 | 0 | 10 |
| Paenibacillus sp. R14(2021) | 170 | 556 | 466 | 34 | 798 |
| Echinicola marina | 30 | 58 | 83 | 0 | 108 |
| Methylococcus sp. Mc7 | 927 | 1520 | 1464 | 25 | 2024 |
| Clavibacter sp. A6099 | 554 | 631 | 959 | 0 | 601 |
| Achromobacter sp. ES-001 | 694 | 726 | 731 | 0 | 1084 |
| Devosia salina | 1534 | 1336 | 1037 | 2 | 1472 |
| Arthrobacter sp. PAMC25284 | 483 | 911 | 1800 | 28 | 2802 |
| Microbacterium sp. PAMC21962 | 0 | 870 | 482 | 0 | 1467 |
| Massilia sp. PAMC28688 | 662 | 2149 | 1497 | 23 | 3161 |
| Janthinobacterium sp. PAMC25594 | 233 | 496 | 556 | 12 | 3970 |
| Brevundimonas sp. PAMC22021 | 0 | 0 | 0 | 0 | 0 |
| Cryobacterium sp. PAMC25264 | 670 | 823 | 1055 | 60 | 1905 |
| Amycolatopsis sp. TNS106 | 11003 | 2538 | 7330 | 122 | 6327 |
| Dyadobacter sp. NIV53 | 107 | 286 | 271 | 0 | 188 |
| Pseudomonas sp. YeP6b | 69 | 112 | 119 | 0 | 214 |
| Paraburkholderia edwinii | 1017 | 1654 | 1423 | 0 | 1766 |
| Pseudomonas sp. Colony2 | 9 | 88 | 58 | 8 | 202 |
| Citrobacter sp. Colony475 | 0 | 0 | 53 | 0 | 10 |
| Citrobacter sp. Colony322 | 0 | 7 | 19 | 0 | 19 |
| Citrobacter sp. Colony219 | 0 | 12 | 16 | 0 | 30 |
| Polymorphobacter sp. PAMC 29334 | 929 | 1157 | 1108 | 0 | 1320 |
| Paracrocinitomix mangrovi | 36 | 29 | 21 | 0 | 29 |
| Horticoccus luteus | 1423 | 3184 | 2529 | 16 | 3261 |
| Curtobacterium sp. TC1 | 432 | 967 | 1171 | 58 | 1959 |
| Pseudomonas sp. ArH3a | 0 | 0 | 0 | 0 | 0 |
| Chryseobacterium sp. LJ668 | 7 | 81 | 113 | 1 | 72 |
| Pseudomonas sp. So3.2b | 56 | 231 | 180 | 7 | 237 |
| Pseudochrobactrum sp. Wa41.01b-1 | 0 | 0 | 0 | 0 | 0 |
| Microbacterium sp. Se5.02b | 1290 | 651 | 1371 | 0 | 850 |
| Aureispira sp. EL160426 | 0 | 0 | 0 | 0 | 0 |
| Shewanella sp. FJAT-52076 | 115 | 99 | 91 | 0 | 107 |
| Shewanella sp. FJAT-53532 | 13 | 124 | 57 | 1 | 58 |
| Shewanella sp. FJAT-53555 | 124 | 89 | 96 | 0 | 107 |
| Shewanella sp. FJAT-53681 | 78 | 149 | 77 | 4 | 80 |
| Shewanella sp. FJAT-53749 | 10 | 25 | 16 | 3 | 41 |
| Shewanella sp. FJAT-53764 | 13 | 176 | 544 | 0 | 129 |
| Shewanella sp. FJAT-53870 | 6 | 33 | 25 | 2 | 29 |
| Shewanella sp. FJAT-54031 | 80 | 114 | 91 | 0 | 89 |
| Shewanella sp. FJAT-51649 | 13 | 28 | 22 | 36 | 13 |
| Shewanella sp. FJAT-51754 | 4 | 46 | 17 | 0 | 47 |
| Shewanella sp. FJAT-51860 | 65 | 61 | 67 | 49 | 101 |
| Shewanella sp. FJAT-52072 | 8 | 133 | 61 | 2 | 78 |
| Candidatus Vallotia sp. (ex Adelges kitamiensis) | 35 | 33 | 49 | 0 | 51 |
| Candidatus Profftia sp. (ex Adelges kitamiensis) | 0 | 1 | 4 | 0 | 3 |
| Lysobacter terrestris | 2385 | 2106 | 1807 | 24 | 1538 |
| Nocardiopsis sp. MT53 | 0 | 1333 | 0 | 0 | 0 |
| Streptomyces akebiae | 1688 | 1941 | 2665 | 171 | 5454 |
| Amycolatopsis sp. DSM 110486 | 14131 | 4594 | 14824 | 280 | 11913 |
| Pseudonocardia sp. DSM 110487 | 15105 | 14717 | 34733 | 379 | 44060 |
| Mesorhizobium sp. AR02 | 2220 | 1738 | 1668 | 32 | 2113 |
| Mesorhizobium sp. AR07 | 2658 | 2320 | 2162 | 72 | 2560 |
| Mesorhizobium sp. AR10 | 3456 | 3484 | 2930 | 74 | 4104 |
| Deefgea tanakiae | 24 | 188 | 156 | 2 | 113 |
| Enterobacter sp. Colony194 | 0 | 0 | 0 | 0 | 0 |
| Halomonas qaidamensis | 8 | 97 | 131 | 19 | 92 |
| Pseudomonas sp. S07E 245 | 98 | 394 | 141 | 0 | 2728 |
| Pseudomonas sp. PS1(2021) | 0 | 0 | 0 | 0 | 0 |
| Ruficoccus sp. ZRK36 | 426 | 1165 | 1043 | 8 | 1153 |
| Pseudomonas sp. ERGC3:05 | 0 | 0 | 0 | 0 | 0 |
| Aggregatibacter sp. Marseille-P9115 | 0 | 0 | 0 | 0 | 0 |
| Pseudomonas sp. Marseille-Q3773 | 226 | 363 | 322 | 2 | 356 |
| Pseudomonas sp. 2hn | 228 | 244 | 321 | 1 | 351 |
| Hydrogenibacillus sp. N12 | 929 | 1614 | 1708 | 19 | 2113 |
| Streptomyces sp. BHT-5-2 | 1472 | 1722 | 2726 | 226 | 5085 |
| Pseudomonas sp. MM213 | 261 | 556 | 582 | 6 | 1044 |
| Paenibacillus sp. PSB04 | 355 | 388 | 310 | 0 | 476 |
| Mycobacterium sp. IDR2000157661 | 2882 | 9830 | 6249 | 34 | 16533 |
| Aliiroseovarius sp. M344 | 0 | 0 | 0 | 0 | 0 |
| Jannaschia sp. M317 | 586 | 651 | 582 | 5 | 708 |
| Jannaschia sp. W003 | 1399 | 1561 | 1922 | 58 | 2386 |
| Leisingera sp. M523 | 247 | 274 | 179 | 1 | 317 |
| Leisingera sp. M527 | 0 | 0 | 0 | 0 | 0 |
| Leisingera sp. M658 | 0 | 0 | 0 | 0 | 0 |
| Leisingera sp. S132 | 487 | 412 | 524 | 15 | 792 |
| Ruegeria sp. B32 | 0 | 0 | 0 | 0 | 0 |
| Sulfitobacter sp. M368 | 168 | 148 | 125 | 0 | 205 |
| Sulfitobacter sp. S190 | 481 | 561 | 450 | 8 | 582 |
| Sulfitobacter sp. S223 | 239 | 177 | 171 | 0 | 183 |
| Sulfitobacter sp. W002 | 114 | 233 | 206 | 2 | 308 |
| Sulfitobacter sp. W027 | 286 | 271 | 201 | 20 | 282 |
| Sulfitobacter sp. W074 | 179 | 113 | 134 | 0 | 236 |
[truncated: 48,568 more chars]
